# Dietary Strategies for Complementary Feeding between 6 and 24 Months of Age: The Evidence

**DOI:** 10.3390/nu15133041

**Published:** 2023-07-05

**Authors:** Leila Harrison, Zahra Padhani, Rehana Salam, Christina Oh, Komal Rahim, Maria Maqsood, Anna Ali, Kimberly Charbonneau, Emily C. Keats, Zohra S. Lassi, Aamer Imdad, Aatekah Owais, Jai Das, Zulfiqar A. Bhutta

**Affiliations:** 1 Centre for Global Child Health, Hospital for Sick Children, Toronto, ON M5G 0A4, Canada; 2 Robinson Research Institute, University of Adelaide, Adelaide, SA 5005, Australia; 3 The Daffodil Centre—A Joint Venture of Cancer Council and The University of Sydney, Sydney, NSW 2006, Australia; 4 Internal Medicine, Aga Khan University Hospital, Karachi 74800, Pakistan; 5 Adelaide Medical School, Faculty of Health and Medical Sciences, The University of Adelaide, Adelaide, SA 5005, Australia; 6 Division of Pediatric Gastroenterology, Hepatology and Nutrition, Department of Pediatrics, SUNY Upstate Medical University, Syracuse, NY 13210, USA; 7 Division of Women & Child Health, Aga Khan University, Karachi 74800, Pakistan

**Keywords:** complementary feeding, infants, young child

## Abstract

Suboptimal complementary feeding practices remain highly prevent. This review aims to comprehensively synthesize new emerging evidence on a set of topics related to the selection and consumption of complementary foods. We synthesized evidence related to five key topics focused on nutritional interventions that target the complementary feeding period, based on four systematic reviews that include updated evidence to February 2022. While there have been many studies examining interventions during the complementary feeding period, there is an overall lack of relevant information through which to draw conclusions on the ideal feeding schedule by food type. Similarly, few studies have examined the effects of animal milk versus infant formula for non-breastfed infants (6–11 months), though those that did found a greater risk of anemia among infants who were provided cow’s milk. This review highlights a number of interventions that are successful at improving micronutrient status and anthropometry during the complementary feeding period, including fortified blended foods, locally and commercially produced supplementary foods, and small-quantity lipid-based nutrient supplements. Complementary feeding education for caregivers can also be used to improve nutrition outcomes among infants in both food secure and insecure populations.

## 1. Introduction

When an infant reaches around the age of 6 months old, their energy and nutrient needs expand beyond what is provided from consumption of breast milk. During weaning, infants transition gradually from breastmilk alone to eating semi-solid, solid, or soft ordinary family foods [1,2], referred to as complementary foods, to meet these additional energy and nutrient needs. This period is referred to as the complementary feeding (CF) period and is typically between 6–24 months of age. Complementary foods should be timely, adequate, safe, and properly fed to meet the infants’ nutritional needs, otherwise growth faltering may occur [3]. Over the past decade, recommendations, guidelines, and political commitment has led to improved breastfeeding practices in many countries. While the numerous benefits of exclusive breastfeeding until 6 months of age and continued breastfeeding (>6 months) are well understood, suboptimal CF practices remain highly prevalent, especially in low- and middle-income countries (LMICs) [4,5,6,7,8]. Suboptimal CF practices include provision of foods to infants that are inadequate in nutrient quality, provided too early or too late, or that are provided in quantities that are too small or infrequent. Suboptimal CF practices persist, in part, due to the unavailability or inaccessibility of nutritious foods and dietary inadequacy. However, another cause of suboptimal CF practices stems from unclear guidelines on how to execute and achieve CF in practice. For example, when to begin CF, in what order to introduce foods, how much of each food group to consume, and more.

It is well documented in the literature that the first two years of life are a critical period for optimal growth and development. Suboptimal CF, characterized by inadequate quality or quantity of foods, poor feeding practices, and increased rates of infection [2], during this window can lead to morbidity, mortality, delayed motor and mental development, and loss of human capital later in life [1,2,9,10,11]. Malnutrition, comprised of both under- and overnutrition as well as diet-related noncommunicable diseases, occurs when the body is deprived of the essential nutrients, vitamins, and minerals required for healthy tissue and organ function [12]. As of 2020, there were 149 million children under the age of five who were stunted, 45 million wasted, and 38.9 million who were overweight or obese globally [12]. With disruptions to food systems and gaps in coverage of essential health and nutrition services caused by the COVID-19 pandemic, these numbers are sure to be further exacerbated in the coming years [13]. 

**Figure 1:**
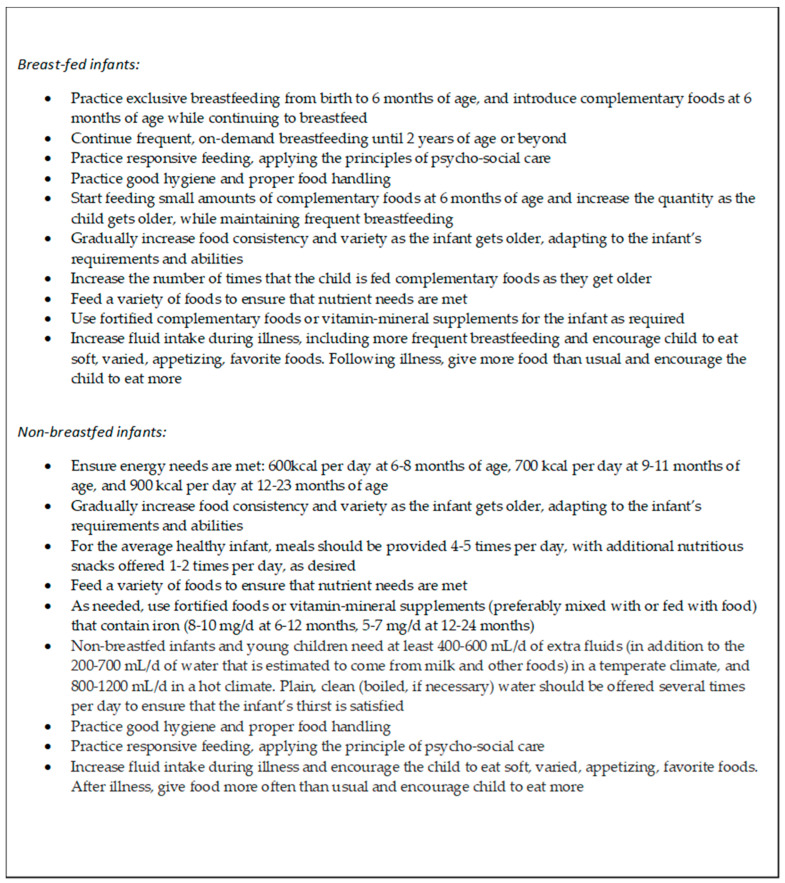
Key Recommendations for Complementary Feeding [10,11].

In 2003 and 2005, the World Health Organization (WHO) put forward guidelines on CF for the breastfed and non-breastfed child, respectively (see Figure 1 key recommendations below) [10,11]. However, there is a need to update this evidence with the more refined research conducted over recent years. Thus, the objective of this review is to comprehensively synthesize new emerging evidence on a set of topics related to selection and consumption of complementary foods. Granted, CF incorporates much more than just what an infant is fed (e.g., how the child is fed is equally important). This review will focus on a subset of topics related to adequate intake of breastmilk and breastmilk-substitutes, solids, and micronutrients during CF and how they influence the growth, morbidity, mortality, and developmental outcomes of the child. Although these elements have been reviewed separately in various recent reviews [14,15,16,17,18], there is a need to consider the evidence in its totality, and to include additional recent information on small-quantity lipid-based nutrient supplements (SQ-LNS) and fortified foods [19]. This review will therefore aim to cover the evidence related to the following dietary components and interventions: (1) continued breastmilk feeding during the CF period; (2) animal milk and infant formula feeding; (3) frequency, types, and amounts of home available complementary foods; (4) supplementation and fortification interventions: provision of complementary foods, SQ-LNS, and micronutrient powders (MNP); and (5) education interventions to promote appropriate CF, usually in food secure situations. 

## 2. Materials and Methods

### 2.1. Selection of Included Topics 

Herein we synthesize the evidence related to five key topics focused on nutritional interventions that target the CF period based on four systematic reviews that include updated evidence to February 2022 for all except one topic, with evidence relevant to April 2023 (see Supplementary Materials). Although several systematic reviews exist on some of the subsets of topics discussed in this review (i.e., SQ-LNS and MNP interventions), our descriptive review builds on them to capture updated review evidence. For all topics, we have summarized the best and most up-to-date available evidence. The need for this update arose given additional information brought to our attention since the previous syntheses for the Lancet Series on Maternal and Child Undernutrition [16], and the background work undertaken as part of an on-going update to the WHO’s infant and young child feeding (IYCF) guidelines, as well as need to summarize the evidence on selection and consumption of complementary foods in its totality for researchers and policy makers. 

A series of new systematic reviews were conducted by our group specifically to inform the update of the WHO’s IYCF guidelines. These reviews covered topics of milk feeding (animal milk versus infant formula during the CF period) (Supplementary File S1), and consumption of differing frequencies, types, and amounts of home available complementary foods (fruit and vegetables (FV); nuts, pulses, and seeds (NPS); and animal-sourced foods (ASF)) and how they influence growth, morbidity, and the developmental outcomes of the child (Supplementary File S2). ASF include many different types of food items that originate from an animal source (e.g., meat, poultry, dairy products, eggs). 

For completeness, we have also included within this review a summary of breastmilk intake during the CF period—a topic that is well established in the literature—and for this, we cite evidence from the Lancet breastfeeding series [18,20,21].

Finally, we identified the most recent and highest quality existing reviews on remaining topics of interest (provision of complementary food and educational interventions), through reference to the updated Lancet Nutrition Series (2021). Each identified review was then updated in order to report evidence relevant to February 2022, and one review had a recent search update of April 2023 (Supplementary Files S3 and S4). 

### 2.2. Methods for New Reviews and Review Updates

Database and grey literature searches were completed by topic, and all screening, data extraction, and quality assessments were conducted in duplicate for both new reviews and updates to existing reviews. All analyses included assessment of the certainty of the evidence using the Grading of Recommendations, Assessment, Development, and Evaluation (GRADE) criteria. The GRADE approach rates the quality of the best available evidence using five criteria: study limitations, consistency of effect, imprecision, indirectness, and publication bias. 

Population groups varied by systematic review, but the majority included all healthy infants aged 6–24 months. Two reviews were specific to the LMIC context. Eligible study designs, interventions, and outcomes were review specific. See Supplementary Files S1–S4 for detailed methods per systematic review topic.

## 3. Results

### 3.1. Breastmilk Feeding during the CF Period 

Global recommendations state that infants should practice introducing complementary foods at 6 months while continuing frequent, on-demand breastfeeding until 2 years of age or older [11]. These recommendations were originally based on a systematic review by Kramer and Kakuma (updated in 2012) in consultation with the WHO and has since been continuously substantiated by a plethora of evidence suggesting numerous health benefits of breastfeeding up to the second year of life for both child and mother in developing and industrialized countries [11,20]. 

Recent evidence by the Lancet Breastfeeding series reported that continued breastfeeding at 6 months of life and after was protective against infant and child mortality and morbidity in both high- and low-income settings [18]. Any breastfeeding was associated with a 52% reduction (95% CI: 40–62) in mortality when compared to no breastfeeding in children aged 6–24 months. Longer periods of breastfeeding were found to be associated with a 26% reduction (95% CI: 22–30) in likelihood of being overweight or obese, regardless of income classification [18]. Similarly, longer periods of breastfeeding were consistently found to be associated with higher performance on intelligence tests, with a pooled increase of 3.4 intelligence quotient (IQ) points (95% CI: 2.3–4.6). It should be noted that breastfeeding for longer than 12 months was associated with a double to triple increase in dental caries (OR = 2.69, 95% CI: 1.28–5.64), however, based on the many benefits associated with breastfeeding, this would suggest a need to improve oral hygiene rather than reduce breastfeeding practices [18]. 

Mothers also receive numerous benefits from breastfeeding, with the strongest evidence for prevention of breast cancer by nursing women. A robust meta-analysis of 47 studies found that for every 12-month increase in lifetime breastfeeding, there was a reduction of 4.3% (95% CI: 2.9–6.8) in incidences of invasive breast cancer [18]. Further evidence suggests a reduced risk of ovarian cancer for mothers with longer periods of breastfeeding, although more research that considers parity is needed. 

In the majority of cases, breastfeeding has a significant positive impact on the health and wellbeing of both infant and mother [21]. In order to improve breastfeeding practices at a population level, the 2023 Lancet Breastfeeding series reports that multilevel and multicomponent interventions that span the socioecological model and settings are needed [21]. 

### 3.2. Animal Milk and Infant Formula Feeding

Despite current recommendations on breastfeeding for optimal nutrition and health protection, many infants are unable to breastfeed in the early months of infancy or may stop breastfeeding before the age of 2 years due to a variety of reasons (e.g., infants born to mothers who are HIV-positive or have lactation and milk-pumping problems) [10,22]. As a substitute, alternative milk beverages (e.g., infant formula and animal milk products) may be consumed to provide the infant with essential nutrients (e.g., protein, calcium, and riboflavin) [10]. Current recommendations suggest infants aged 6–24 months consume ~200–400 mL/d of milk in combination with ASF, or ~300–500 mL/d if only consuming milk [10,11].

To update the WHO’s guidance on consumption of alternative milk beverages, a systematic review assessed the effects of the consumption of animal milk compared to infant formula in non-breastfed or mixed breastfed infants aged 6–11 months [14]. See S1.1.–S1.5. in Supplementary File S1 for more detail on review methods. 

#### 3.2.1. Animal Milk

**Figure 2:**
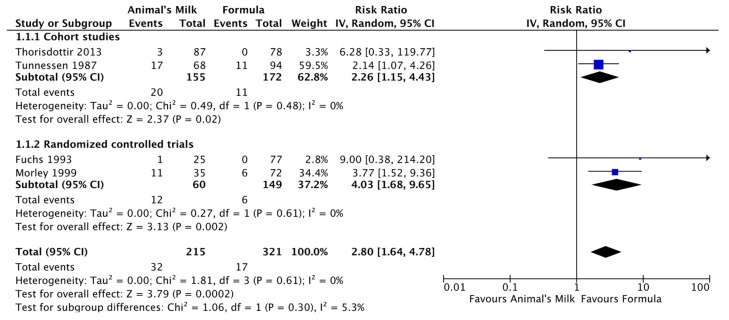
Forest plot for cow’s milk vs. infant formula on anemia.

There is discourse in the literature on the safety of feeding animal milk, such as cow’s milk, to infants under 12 months of age. This is due to the low iron content of cow’s milk, which has been associated with gastrointestinal blood loss, iron deficiency anemia, and increased solute load for kidneys [10,23,24,25]. Recent evidence continues to suggest increased risk of iron deficiency anemia from consumption of cow’s milk during infancy. Meta-analyses found that consumption of cow’s milk between 6–11 months of age in apparently healthy non-breastfed infants, compared to consumption of infant formula, seemed to increase the risk of anemia during infancy by two to four times (Figure 2) [14]. In addition, when considering duration of cow’s milk consumption, for each additional month of cow’s milk feeding, the risk of anemia increased by 23% (*p* < 0.001). See Table 1. Three of the included studies either used fortified the cow’s milk or provided additional iron through complementary foods. However, these studies were not included in every anemia-related outcome, thus, there is not enough evidence to determine whether additional supplementation or fortification of cow’s milk could potentially avert the risk of anemia. The proposed mechanisms that may cause cow’s milk to increase the risk of anemia include: the decreased amount of iron found in cow’s milk, decreased bioavailability of iron, and increased blood loss from the gastrointestinal tract [11,14]. 

#### 3.2.2. Infant Formula 

In comparison to breastmilk, infant formula has historically been composed of mainly cow’s milk with added wheat flour, malt flour, and potassium bicarbonate [26]. Following the past 20 years of innovation, infant formula has undergone many improvements, including the addition of oligosaccharides, lactoferrin, and osteopontin, as well as other beneficial micronutrients and active ingredients, in the hopes of mimicking the nutritional composition of human breast milk [26]. Although breastmilk is still regarded as optimal for nutrition and health, powdered, liquid, or ready-to-feed infant formulas provide similar nutrient profiles to breastmilk and have advantages over animal milk, especially in settings where food supplements or fortified foods are not available. The recent Dietary Guidelines for Americans recommends that infants less than 12 months old should not consume cow’s milk but, alternatively, infant formulas [27]. See S1.6.–S1.16. in Supplementary File S1 for all evidence on effects of animal milk versus infant formula. 

According to the WHO’s guiding principles for non-breastfed infants, if infant formula is available, affordable, and can be used safely (e.g., uncontaminated water is available for mixing with powdered infant formula and proper handling and storage are possible), infants at 6–12 months of age need ~280–500 mL/d if consuming other animal-source foods in their diet, and ~400–550 mL/d if not [11]. Notably, the International Code of Marketing of Breast-Milk Substitutes was enacted in 1981 by the WHO to prevent aggressive and inappropriate marketing tactics for breast-milk substitutes [28]. Under this code, breastfeeding is recommended over alternative milk feeding, due not only to the risk posed by not receiving the protective qualities of breast milk, but primarily because milk alternatives and feeding bottles carry a high risk of contamination, which can cause life threatening infections (e.g., use of non-sterile bottles or contaminated water). Furthermore, alternative milks are costly and require that mixing by the caregiver is done properly [28]. 

### 3.3. Frequency, Types, and Amount of Home Available Complementary Foods

The frequency, type, and amounts of complementary foods consumed from 6–24 months of age are critical components of ensuring appropriate CF and ensuring that the nutrient needs of the child are being met for optimal growth and development. According to the guiding principles for CF put forward in 2003, based on an average intake of breastmilk among healthy infants, the energy needs from complementary foods are estimated to be about 200 kcal per day for infants aged 6–8 months, 300 kcal per day for infants 9–11 months, and 550 kcal per day for infants 12–23 months [11]. This translates into a meal frequency of two to three times per day for the youngest infants (6–8 months) and three to four times per day through the rest of the CF period. For non-breastfed infants, these estimates increase to be about 600 kcal per day for infants of 6–8 months, 700 kcal per day for infants of 9–11 months, and 900 kcal per day for infants of 12–23 months [10]. For non-breastfed infants, the meal frequency is defined as four to five times per day throughout the entire CF period (6–24 months). Furthermore, the WHO recommends feeding a variety of complementary foods that are nutrient-dense (e.g., foods with high micronutrient content in relation to energy content). Preferably, these foods come from local sources that are rich in essential vitamins and minerals, such as ASF, FV, and NPS, in order to meet nutritional demands during this period of rapid growth and development [10,11]. Infant diets should contain adequate fat content and should avoid beverages with low nutritional value (e.g., sugary juices and tea). 

To update WHO’s guidance on the consumption of complementary foods, a systematic review assessed differing frequencies, varieties, and amounts of consumption of three recommended food groups (FV, NPS, and ASF) on the dietary and health outcomes of healthy infants aged 6–24 months. See S2.1.–S2.5. in Supplementary File S2 for more detail on review methods. 

#### 3.3.1. Fruit and Vegetables (FV) 

FV provide nutrients that are vital to health and growth, such as potassium, folate, fiber, vitamin A, vitamin C, vitamin K, and many phytochemicals [29,30]. Notably, beta-carotene-rich FV, such as orange/red, yellow, and leafy green vegetables and yellow fruits, are important for reducing vitamin A deficiencies in infants and have been found to meet many other vitamin needs [11]. For example, an infant of 6–24 months old only needs to consume one tablespoon of sweet potato to meet their vitamin A needs for one day [31]. The WHO recommends consuming FV daily, however, exclusive vegetarian diets are not advised due to their inability to provide adequate amounts of some micronutrients [11]. 

The systematic review identified 20 studies that reported on differing frequencies, varieties, or amounts of FV consumption on anthropometric outcomes, nutrient status, child development, diarrhea, and subsequent consumption of FV or food/taste preferences later in life. None of the included studies provided high certainty evidence in support of greater frequencies, varieties, or amounts of FV leading to improved dietary and health outcomes, which was mainly because most outcomes of interest only had one study contributing to the evidence base. Furthermore, almost all included studies were observational in nature (with one randomized controlled trial (RCT)), thereby limiting the ability to establish causality and, importantly, only approximately half of the studies adjusted for potential confounding variables. As a result of this, we choose to report in the main only the highest quality evidence from three observational studies and one RCT. All four studies found that greater frequency or variety of FV consumption during the CF period was associated with subsequent consumption or liking of FV items later in life. See Table 2. All other study results can be found in S2.6.–S2.7. in Supplementary File S2. 

#### 3.3.2. Nuts, Pulses, and Seeds (NPS)

NPS, such as peanuts, lentils, or pumpkin seeds, provide important macronutrients (protein, carbohydrates, and essential fats), micronutrients (e.g., iron and zinc), energy, and fiber. Iron represents the largest nutrient gap in infants at 6–24 months old, where, globally, the WHO estimates that 42% of children less than 5 years old were found to be anemic in 2022 [32]. Iron, especially bioavailable iron, is most abundant in ASF such as meat, poultry, or fish. However, NPS are another important source of iron, especially when combined with other foods rich in vitamin C that allow for improved absorption of iron by the body [33]. In addition to iron, NPS are also a good source of protein, an important macronutrient. NPS are a particularly important food group in low-income populations, as they can add nutritional value to diets when ASF, which tend to be costly, are not accessible. Their long storage life and relatively low cost make them a key source of protein, energy, and micronutrients, particularly in LMICs. 

Concerns around allergies can be a reason why commonly allergenic foods, such as nuts (e.g., peanuts), are not introduced early to a child. However, there is accumulating evidence to suggest that delays in introduction may promote the development of, versus prevent, the allergy [34,35]. Another common concern around the provision of NPS to infants includes choking, although parents are advised to puree or mash the nut or seed into a paste, thereby allowing the child to consume the food safely while receiving the nutritional value that NPS provides. 

The systematic review identified seven studies that reported on differing frequencies or amounts of NPS consumption on anthropometric outcomes, nutrient intakes, and diarrhea. Again, very limited and low certainty evidence was found, where one RCT from Nigeria demonstrated an effect between greater amounts of maize and cowpea consumption with increased length and weight measurements after 12 months of follow-up (*p* < 0.05 between groups). In addition, one RCT from Ethiopia reported an effect between greater amounts of broad bean consumption with improved protein, carbohydrate, and iron intake after 6 months of follow-up (*p* < 0.05 between groups). See Table 3. All other study results can be found in S2.6.–S2.7. in Supplementary File S2. 

#### 3.3.3. Animal-Sourced Foods (ASF)

According to the WHO’s guidelines, meat, poultry, fish, or eggs should be eaten daily, or as often as possible [11]. In comparison to plant-sourced foods, ASF are considered more nutritionally dense, with high sources of energy and readily digested protein, while also containing an array of bioavailable micronutrients (e.g., iron, zinc, calcium, vitamin A, vitamin B_12_, riboflavin, and folate) that are typically challenging to obtain in adequate amounts from foods of plant origin alone [36,37,38,39]. 

The systematic review identified 55 studies that reported on differing frequencies, varieties, or amounts of ASF consumption on a range of dietary and health outcomes. The data was highly heterogeneous, with different exposures and outcomes reported throughout. However, a few meta-analyses were possible, thus, we report pooled estimates below (see S2.8.–S2.10. in Supplementary File S2 for additional results). Like findings from the FV and NPS food groups, none of the included studies provided high certainty evidence in support of greater frequencies, varieties, or amounts of ASF leading to improved dietary and health outcomes. 

##### Eggs

**Figure 3:**
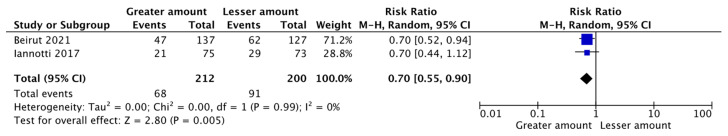
Forest plot for egg consumption vs. control on stunting.

A meta-analysis of two studies from low-resource settings found that children aged 6–9 months who consumed one egg per day over 6 months, compared to no eggs, had a borderline improved weight-for-age z-score (WAZ) by 0.15, but this had no effect on the height-for-age z-score (HAZ) or weight-for-height z-score (WHZ). WAZ, HAZ, and WHZ are indicators of malnutrition status, derived from anthropometric measurements according to the WHO’s Child Growth Standards [40]. A second meta-analysis of two studies from low-resource settings showed that children ranging from 6–12 months of age who consumed greater amounts of egg, compared to no egg, over a period of 6 to 17 months, had reduced risk of stunting by 30% (Figure 3). See Table 4. 

##### Red Meats, Chicken, Fish, and Insect-Based Food Consumption 

Although a number of RCTs and observational studies reported on red meat consumption during the CF period, studies were highly heterogeneous between exposure and outcome characteristics, thus, no meta-analyses were possible. Few studies reported on differing frequencies, varieties, or the amount of chicken, fish, or insect-based food consumption. 

##### Other ASF Products

**Figure 4:**
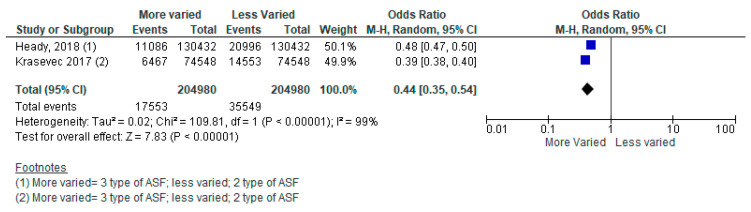
Forest plot for three types of animal-sourced foods (ASF) vs. two types of ASF on stunting.

**Figure 5:**
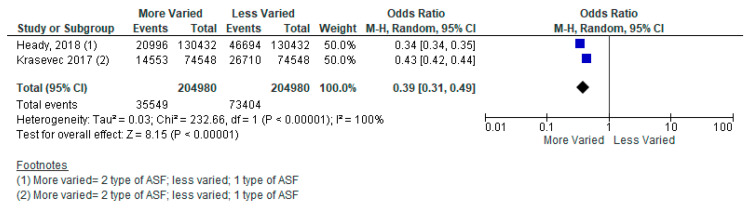
Forest plot for two types of animal-sourced foods (ASF) vs. one type of ASF on stunting.

**Figure 6:**
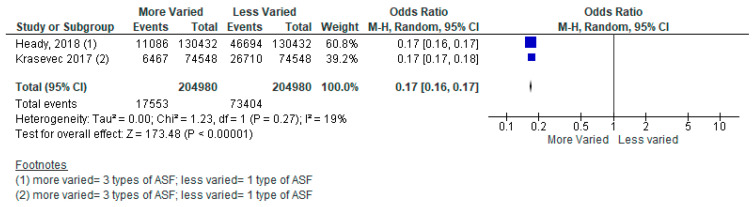
Forest plot for three types of animal-sourced foods (ASF) vs. one type of ASF on stunting.

Several included studies did not specify what type of ASF product was being consumed. For example, a meta-analysis of two studies from 49 LMICs reported on consumption of zero to three types of ASF, however, no further detail was provided on what the different types of ASF were (e.g., red meat or chicken). The pooled analysis revealed that children aged 6–24 months who consumed three types of ASF, compared to two types, had a 56% reduced likelihood of stunting (Figure 4) [41,42]. Children who consumed two types of ASF, compared to one type, had a 61% reduced likelihood of stunting (Figure 5). Lastly, children who consumed three types of ASF, compared to one type, had an 83% reduced likelihood of stunting (Figure 6). See Table 4. 

### 3.4. Provision of Complementary Food Interventions

Ideally, caregivers select foods for infants that deliver the required nutrients for optimal growth and development from home available complementary foods that are nutrient-dense, and preferably from local sources. However, in resource-poor settings, it is likely that infants do not have access to nutrient-rich diets and, thus, become deficient in macro- and micronutrients, leading to malnutrition and, in extreme cases, mortality. As such, several interventions, including use of vitamin-mineral supplements or fortified products, have been found to be effective in improving child malnutrition in vulnerable settings. These include micronutrient supplementation, SQ-LNS, large-scale fortification, targeted fortification, and point-of-use fortification with MNP [43]. As micronutrient supplementation and fortification interventions have been reviewed holistically elsewhere [43], we provide updated evidence on several key interventions in this section, with a focus on the CF period for healthy children. 

To review the highest quality and most recent evidence on preventative supplementation and fortification interventions for infants aged 6–24 months, two systematic reviews were updated. One update to the review by Lassi and colleagues [16] covers provision of complementary food interventions and SQ-LNS. In this update, 44 studies were included from LMICs (see S3.1.–S3.5. in Supplementary File S3 for more detail on review update methods). The second update to the review by Salam and colleagues [17] covers MNPs. In this update, 19 studies were included from LMICs (see S4.1.–S4.4. in Supplementary File S4 for further detail). 

To better understand how provision of complementary food interventions effects dietary and health outcomes in infants aged 6–24 months living in LMICs, in consultation with the WHO nutrition team, interventions were subdivided into those that supplied (a) fortified blended foods (i.e., corn-soy or wheat-soy blends), (b) locally produced ready-to-use supplementary foods (RUSF) (i.e., rice-lentil or chickpea-based RUSF), (c) commercially produced RUSF (i.e., Plumpy’Sup^TM^), (d) SQ-LNS (i.e., LNS that provides 100–120 kcal/day), or e) a mixed category that included any other food intervention that did not fit within the above categories, described as alternative foods (i.e., caterpillar cereal). RUSF are food supplements intended to be used to manage moderate acute malnutrition in children aged 6 months or older. Results were also grouped by food security status. For all supplementation and fortification interventions (including MNPs), it should be noted that interventions were provided preventatively, thus, children were not malnourished at baseline. Given that a plethora of meta-analyses were conducted, we have opted to report only significant findings below. All other study results can be found in S3.6. of Supplementary File S3 and S4.5.–S4.6. of Supplementary File S4. 

#### 3.4.1. Provision of Fortified Blended Foods, Locally and Commercially Produced Ready-to-Use Supplementary Foods, and Alternative Foods 

**Figure 7:**
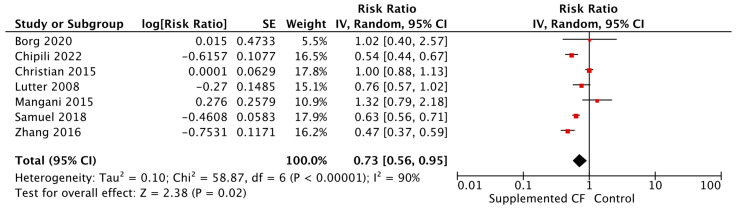
Fortified blended foods vs. control on stunting.

**Figure 8:**
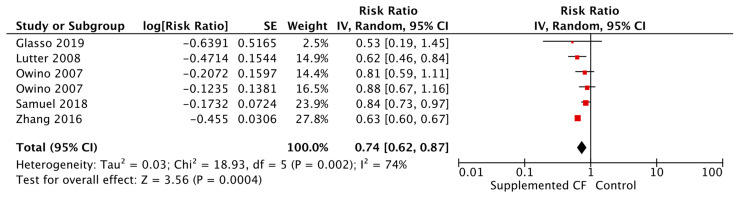
Fortified blended foods vs. control on anemia.

Updated evidence supports the use of fortified blended foods to improve dietary and health outcomes in children living in LMICs when compared with a control or standard of care. Fortified blended foods significantly improved HAZ, WAZ, WHZ, and hemoglobin levels (see Table 5). Furthermore, the risk of stunting was reduced by 27% (Figure 7) and anemia by 26% (Figure 8). 

Locally produced RUSF were found to improve HAZ, WHZ, WAZ, and changes in weight and height, when compared with a control or standard of care (Table 5). 

**Figure 9:**
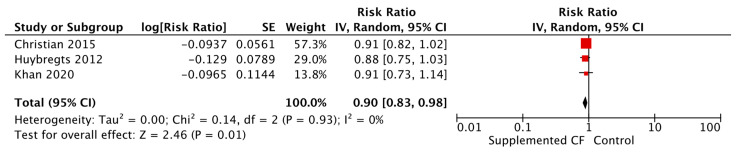
Commercially produced ready-to-use supplementary foods (RUSF) vs. control on stunting.

Commercially produced RUSF reduced the risk of stunting by 10% (Figure 9), wasting by 25%, death by 57%, and improved WHZ, WAZ, mid-upper arm circumference (MUAC), and changes in weight and height, when compared to control or standard of care (Table 5). However, it should be noted that only one study contributed to the outcome of MUAC. MUAC refers to the circumference of the upper arm, measured at the midpoint between the shoulder and elbow and is a measurement of malnourishment. 

Alternative foods were found to reduce the risk of skin conditions (i.e., rashes, bruises, scrapes, cuts) by 44%, and anemia by 48%, with improved hemoglobin levels, when compared to control or standard of care in one study. However, alternative foods (i.e., eggs) were also found to increase the risk of diarrhea in one study (Table 5).

#### 3.4.2. Lipid-Based Nutrient Supplementation

LNS are designed with the purpose of providing multiple micronutrients embedded within a food base that also provides energy, protein, and essential fatty acids, and have been found to be effective in improving malnutrition in infants [44,45,46]. They are described as ‘lipid-based’ because most of the energy from the product is derived from lipids or fats. There are three differing quantities of LNS, small quantity LNS (SQ-LNS), which provide 100–120 kcal/day, medium quantity LNS, which provide 250–500 kcal/day, and large quantity LNS, which provide ≥500 kcal/day [44]. Small and medium quantity LNS are meant to be consumed alongside household foods for the prevention of undernutrition, whereas LNS provided in large quantities were developed and have been proven effective in treatment of children with severe acute malnutrition. 

**Figure 10:**
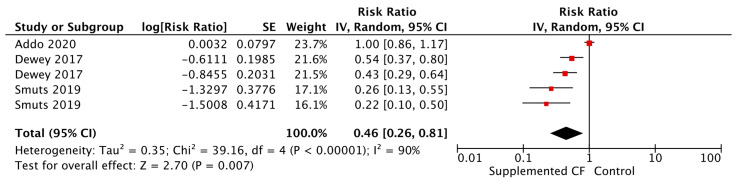
SQ-LNS vs. control on iron deficiency anemia.

Updated evidence supports the use of SQ-LNS to improve dietary and health outcomes in children living in LMICs, when compared with a control or standard of care. SQ-LNS significantly reduced the risk of wasting by 10%, iron-deficiency anemia by 54% (Figure 10), upper respiratory tract infections by 13% (only one study), and improved MUAC. SQ-LNS was also found to increase mean diarrhea episodes, however, only one study contributed to this outcome. See Table 6.

#### 3.4.3. Provision of Complementary Food Interventions by Food Security Status 

**Figure 11:**
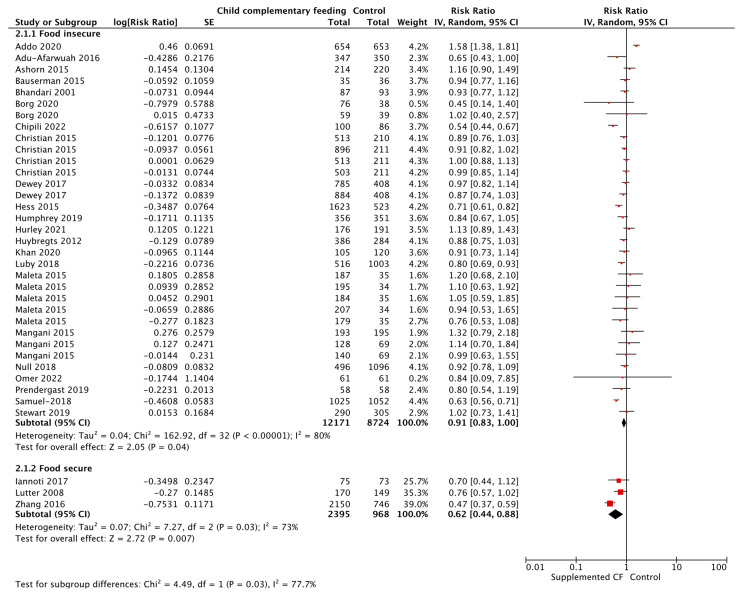
Provision of complementary foods vs. control on stunting, by food security status.

**Figure 12:**
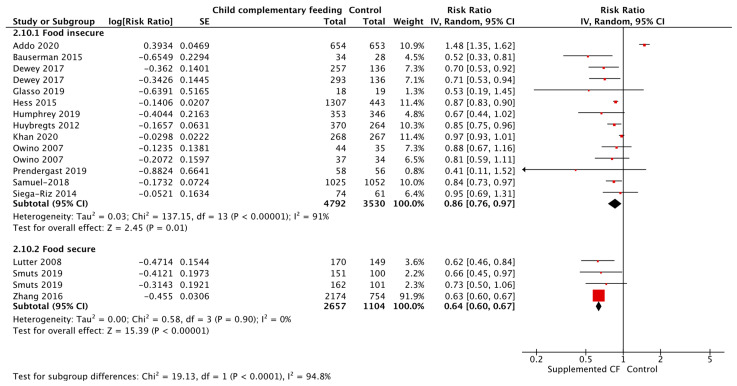
Provision of complementary foods vs. control on anemia, by food security status.

When looking at food security status (Table 5), updated evidence supports the use of complementary food provision (including SQ-LNS) to reduce the risk of iron deficiency anemia by 76%, and skin conditions by 44%, when compared to a control in food secure populations (note: only one study contributed to these outcomes). However, again we report increased risk of diarrhea in food secure groups with CF provision (i.e., eggs) in one study. Use of complementary food provision was found to reduce the risk of wasting by 13%, and improved HAZ, WAZ, MUAC, and changes in height, when compared to control in food insecure populations. For both food secure and insecure populations, hemoglobin levels were improved, the risk of stunting was reduced by 38% and 9% (Figure 11), and risk of anemia was reduced by 36% and 14% (Figure 12), respectively, when compared to a control. 

#### 3.4.4. Micronutrient Powders 

In addition to SQ-LNS, MNPs are another form of supplementation that has been found to be effective in reducing malnutrition in vulnerable groups of children [43]. MNPs are packaged in a single-serving sachet containing a dry powder mixture of 15 essential vitamins and minerals that are most commonly missing in children’s diets [43]. The powders can be sprinkled directly onto ready-to-eat semi-solid food or are added during the cooking process. The WHO recommends use of MNP for point-of-use fortification of complementary foods in infants and young children aged 6–24 months to improve iron status and reduce anemia [47]. 

Updated evidence from 19 RCTs found that MNPs consumed by apparently healthy children aged 6–24 months living in LMICs, when compared to no intervention or placebo, had improved hemoglobin levels, WAZ, and WHZ, but no effect was found for anemia, HAZ, being underweight, stunting, wasting, diarrhea, or upper respiratory illness. See Table 7. 

### 3.5. Education to Promote Complementary Feeding 

In addition to supplementation and fortification interventions, nutrition education and counselling provided to caregivers on the feeding of young children also has the potential to improve the selection of complementary foods for and ultimately the nutritional status of children in developing countries [48]. Appropriate CF is dependent on accurate information and skilled support from the family, community, and health care system [49]. It has been found that incorrect knowledge about appropriate foods and feeding practices can be a greater determinant of malnutrition than having a lack of food itself [49]. As such, several educational strategies have been used with the aim of improving CF practices. For example, nutritional counseling to caregivers to promote healthy feeding practices through local channels, one-on-one, in group meetings, or through feeding demonstrations. Nutrition messaging may be delivered through advertisements/posters, home visits, immunization clinics, sick childcare contacts/health care providers, women’s meetings, or neighborhood meetings. The counselling may include recommendations on what foods to give young children, timing of beginning complementary foods, meal frequencies, amounts of complementary foods to be fed, ways to encourage children to eat more, or continued feeding during illness. 

To review the highest quality and most recent evidence on educational interventions to promote CF practices in infants aged 6–24 months, a systematic review by Lassi and colleagues [16] was updated. In this update, 47 studies were included from LMICs (see S3.1.–S3.5. in Supplementary File S3 for more detail on review update methods). All but one study included children who were not malnourished at baseline. We separately report analyses on CF education among undernourished children based on one study [50]. Given that a plethora of meta-analyses were conducted, we have opted to report only significant findings below. All other study results can be found in S3.6. of Supplementary File S3. 

#### 3.5.1. Complementary Feeding Education Interventions

**Figure 13:**
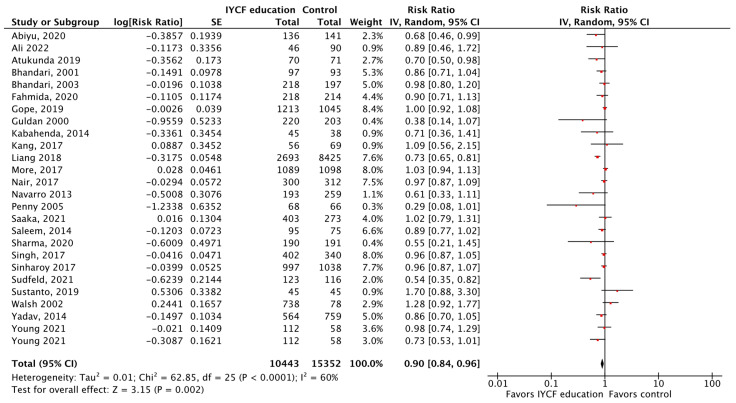
Complementary feeding education vs. control on stunting.

Updated evidence suggests that CF education versus control significantly improves HAZ, WAZ, WHZ, changes in weight, and reduces the risk of stunting by 10% (Figure 13), being underweight by 13%, being severely underweight by 61% (only one study), severe wasting by 86%, and respiratory illness by 27%, in children aged 6–24 months living in LMICs (Table 8). Lastly, CF education during pregnancy reduced the risk of low birth weight (LBW) by 53% when compared to control. LBW is defined as a baby born weighing less than 2500 g (5.5 pounds). 

#### 3.5.2. Complementary Feeding Education Interventions by Food Security Status

**Figure 14:**
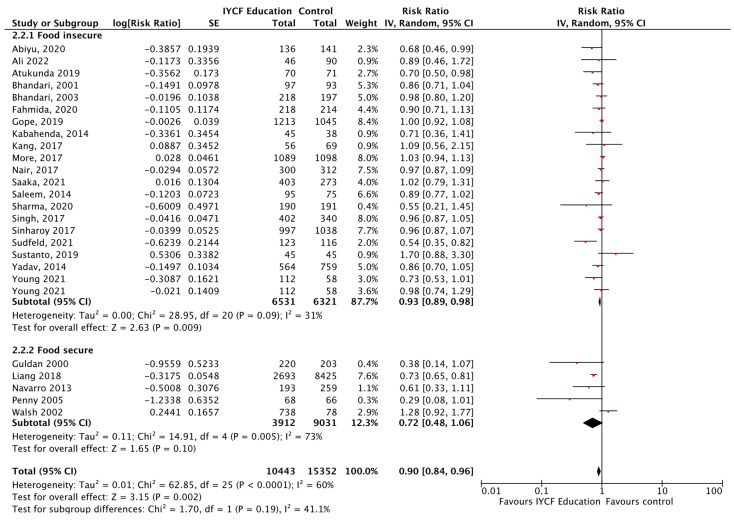
Complementary feeding education vs. control on stunting, by food security status.

**Figure 15:**
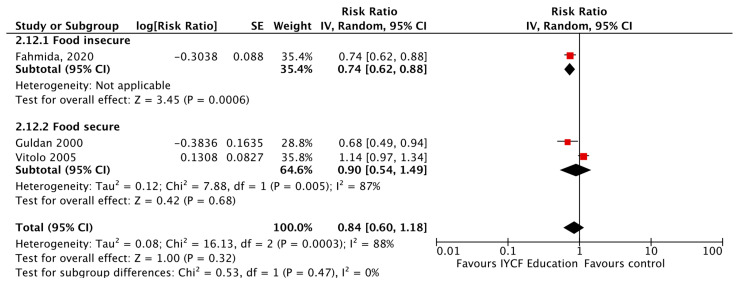
Complementary feeding education vs. control on anemia, by food security status.

Given that food insecurity plays a major role in dietary and health outcomes, a subgroup analysis was conducted between food secure versus food insecure groups. CF education, versus control, significantly reduced the risk of wasting by 22%, diarrhea by 33%, respiratory illness by 37%, and improved changes in height in food secure populations. For those who were food insecure, CF education, versus control, significantly reduced the risk of stunting by 7% (Figure 14), anemia by 26% (Figure 15), and improved changes in weight. For both food secure and insecure populations, the risk of being underweight was reduced by 29% and 10%, respectively, and HAZ and WAZ were improved when compared to a control. See Table 8. 

#### 3.5.3. Complementary Feeding Education Interventions in Undernourished Children

Lastly, one study evaluated the effect of a smartphone-based maternal education programme for CF of undernourished children in a food secure middle-income country. In this context, CF education versus control significantly improved WHZ, WAZ, and HAZ (see Table 8). 

## 4. Discussion

This descriptive review has synthesized the best and most up-to-date evidence on several aspects of CF, including the provision of breastmilk substitutes, the effects of varying frequencies, types, and amounts of key food groups, and the impacts of interventions such as nutrition education, MNPs, and supplementary foods/nutritional products. While there have been many studies examining interventions during the CF period, there is an overall lack of relevant information with which to draw conclusions on the ideal feeding schedule by food type. However, preliminary evidence has suggested that there could be improved infant anthropometric outcomes with increased frequencies, varieties, and amounts of consumption of FV, NPS, and ASF, and that greater exposure and variety of FV may lead to better uptake of these foods later in childhood. Similarly, few studies examined the effects of animal milk versus infant formula for non-breastfed infants (6–11 months), though those that did found a greater risk of anemia among infants who were provided cow’s milk. This review has highlighted a number of interventions that are successful at improving micronutrient status and anthropometry during the CF period, including fortified blended foods, locally and commercially produced supplementary foods, and SQ-LNS. CF education for caregivers can also be used to improve nutrition outcomes among infants in both food secure and insecure populations. 

When complementary foods are being introduced but breastfeeding is no longer an option, animal milk or infant formulas can be suitable alternatives. The observed association between cow’s milk and increased risk of anemia may suggest that infant formulas are more appropriate. The WHO’s guiding principles for non-breastfed infants states that feeding animal milk with appropriate complementary foods (i.e., those that are high in iron content and bioavailability) and enough fluid (adequate hydration) is considered relatively low risk, since iron deficiency provoked by gastrointestinal blood loss resolves by 12 months of age, and further effects are not seen if the milk is heat-treated [10]. Furthermore, the low iron content and bioavailability of cow’s milk could be offset by supplementing with iron (i.e., MNPs). Some benefits of feeding animal milk are that it does not require preparation with clean water, and it tends to be less expensive than infant formulas, and so is more accessible in LMICs. For example, goat milk is readily available and may be used as an alternative to cow’s milk in some cultures. Nonetheless, breastfeeding when possible is always preferred over alternative milk beverages, given the plethora of health benefits associated with breastmilk. In addition, breastmilk is economically superior to alternative milk beverages, and breastfeeding promotion has been found to be the most cost-effective strategy to ensure optimal health and growth for those who can breastfeed [51]. 

Our understanding of the optimal feeding schedule by food type remains unclear. Previous studies have reported that repeated exposure to FV during infancy was associated with better acceptance and improved intake later in childhood [52,53,54,55,56], supporting our preliminary findings. However, not a lot of research has gone into determining the frequency and amount of each food that should be offered during CF, leaving mothers and caregivers with little guidance. Notably, NPS and certain locally available ASF (i.e., caterpillars) are low cost and more readily available than other high protein foods in many low resource settings. Thus, they should be further evaluated for their nutritional value during the CF period, and represent a promising food source for improving CF practices and food choices in food insecure settings [57,58]. In addition, as we continue to observe the harmful effects of our changing climate, alternative dietary protein sources (i.e., edible insects, plant proteins, and cell-culture-based proteins) can provide an alternative to ASF with a lower environmental footprint [59]. 

The timing of the introduction of CF also remains under debate, with large global variability in patterns of initiation ranging from early initiation (4 months) to late introduction (>6 months) [60]. A recent systematic review included 268 documents, seven RCTs (from 24 papers) and 217 observational studies (from 244 papers) with the objective of determining the impact of early and late introduction of CF on infant health, nutrition, and developmental outcomes, to identify the optimal time to start CF [60]. Evidence from RCTs did not suggest a difference for early introduction, while low-certainty evidence from observational studies suggests that early introduction of CF (<6 months) might increase body mass index z-score, being overweight, and obesity [60]. The review concludes that evidence remains insufficient but does suggest increased adiposity with early introduction, thus, the current recommendation by the WHO to start introducing complementary foods at 6 months should stand. More robust studies, in particular from LMICs, are needed to inform recommendations [60]. 

In settings where nutrient-dense foods found in the traditional diet are not available/accessible, our updated analyses continue to support the literature that fortified blended foods, locally and commercially produced supplementary foods, SQ-LNS, and educational interventions all have the potential to improve nutrient intake in young children [61]. For example, we report similar findings from updated analyses on fortified blended foods, compared to a systematic review and meta-analysis that compared fortified complementary foods with an unfortified version of the same complementary product [19]. In both reviews, a reduction in the risk of anemia was found following intervention. 

A limitation of this review is the inclusion of many different interventions and, by extension, the extensive number of analyses undertaken, which led to a very large synthesis and, thus, we were unable to discuss all findings in their totality within the main paper. For some topics (i.e., animal milks and frequency, variety, and amount of FV, NPS, and ASF) we found limited evidence and, thus, report findings from observational studies, increasing the likelihood of bias. It should also be noted that all evidence synthesized herein was of low or moderate certainty, thus, no studies provided high certainty evidence for updating the WHO’s IYCF guidelines.

One key question that remains is: which intervention should be used for targeted micronutrient needs based on setting? In order for an intervention to be maximally effective, careful diagnostic assessment should occur to inform which strategies will be most beneficial for a target population [43]. For example, during the decision-making process, in addition to establishing population-level micronutrient deficiencies for a given population, ideally through biochemical data, factors such as existing supply chain infrastructure, cost of production, sustainability, and strategies for monitoring and evaluation should be examined. It is likely, especially for children living in LMICs and settings with a high prevalence of multiple micronutrient deficiencies, that several strategies implemented in conjunction with one another will have greater impact. For example, previous reviews have reported that CF education in conjunction with supplementation was more effective in improving growth outcomes than education alone [62]. Future country-specific implementation research should focus on contributing to this gap in our understanding of which interventions to use in which context. 

## 5. Conclusions

This review highlights the need for more research to be conducted examining breastmilk substitutes (i.e., animal milk and infant formulas), and various frequencies, varieties, and amounts of FV, NPS, or ASF consumption during the CF period, and the association with dietary and health outcomes. Ideally, such research would take the shape of good quality experimental and quasi-experimental studies. Findings from experimental studies continue to support the use of fortified blended foods, locally and commercially produced supplementary foods, SQ-LNS, and CF educational interventions to improve nutrition outcomes among both food secure and insecure populations. 

## Figures and Tables

**Table 1:** Summary of evidence for animal milk and infant formula feeding.

Exposure/ Intervention	Location	Evidence Reviewed	Effect
Cow’s Milk vs. Infant Formula	United Kingdom; Iceland; United States (*n* = 2)	A Meta-Analysis of Two Randomized Controlled Trials (RCTs) and Two Cohort Studies.	**Anemia** [Cohort studies: Relative Risk (RR) = 2.26, 95% Confidence Interval (CI) 1.15–4.43, two studies, *p* = 0.02, I^2^ = 0%, Grade certainty: Low; RCTs: RR = 4.03, 95% CI 1.68–9.65, two studies, *p* = 0.002, I^2^ = 0%, Grade certainty: Low]

**Table 2:** Summary of evidence for fruit and vegetable consumption.

Exposure/ Intervention	Location	Evidence Reviewed	Effect/Association
Less Frequent vs. More Frequent Fruit and Vegetables (FV)	Norway and Nepal	Two Cohort Studies Ranging from 231–9490 Participants	**Stability and change** [Overall fruit consumption at 18 months was positively associated with overall fruit consumption at 36 months (Spearman’s rho = 0.36) and at 7 years of age (Spearman’s rho = 0.23), GRADE certainty = very low]
			**Stability and tracking** [Moderate stability for the frequency of consumption of yellow fruits and vegetables and dark green leafy vegetable consumption using Generalized Estimating Equation (GEE) models (stability coefficient = 0.26, 95% CI: 0.18–0.35), GRADE certainty = very low]
Less Varied vs. More Varied FV	Germany and France	One Quasi-Experimental Study and its Associated Report with a Total of 254 Participants	**Intake of new foods** [The high vegetable variety produced the greatest increase in intake of new foods (*p* < 0.0001), GRADE certainty = very low]
			**Mean number of vegetables eaten** [At follow up three (~67 months), children who had experienced a high variety of vegetables at weaning ate more of the new vegetables and familiar vegetables than those who had experienced low or no variety (14.1 g ± 1.5 vs. 4.3 g ± 1.5 and 3.2 g ± 1.4, *p* < 0.0001 for new vegetables, respectively; and 9.6 g ± 2.0 vs. 13.1 g ± 2.0 and 13.1 g ± 1.9, *p* = 0.03 for familiar vegetables, respectively, GRADE certainty = very low]
	Australia	One Cohort Study with 333 Participants	**Vegetable and fruit intake** [A greater variety of vegetables tried at age 14 months was significantly associated with a higher fruit and vegetable intake score at age 3.7 years (Reg coefficient = 0.12, *p* = 0.05), GRADE certainty = very low]

**Table 3:** Summary of evidence for nuts, pulses, and seeds.

Exposure/ Intervention	Location	Evidence Reviewed	Effect
Greater Amount vs. Lesser Amount of Nuts, Pulses, and Seeds (NPS)	Nigeria	One Randomized Controlled Trial (RCT) with 90 Participants	**Length** [Increasing measurements for length were seen for those who consumed maize/cowpea compared to those who did not (*p* < 0.05 between groups), GRADE certainty = very low] **Weight** [Increasing measurements for weight were seen for those who consumed maize/cowpea compared to those who did not (*p* < 0.05 between groups), GRADE certainty = very low]
	Ethiopia	One RCT with 197 Participants	**Nutrient intake** [improved nutrient intakes were observed for protein, carbohydrate, and iron intake for those consuming greater amounts of broad bean (*p* < 0.05 between groups), GRADE certainty = very low]

**Table 4:** Summary of evidence for animal-sourced foods.

Exposure/ Intervention	Location	Evidence Reviewed	Effect/Association
More Varied vs. Less Varied Animal-sourced Foods (ASF)	49 Low- and Middle-Income Countries from Across World Health Organization Regions	A Meta-Analysis of Two Cross-Sectional Studies	*Three Types of ASF* vs. *Two Types* **Stunting** [Odds Ratio (OR) = 0.44, 95% Confidence Interval (CI): 0.35–0.54, 409,960 participants, I^2^ = 99%, GRADE = very low] *Two Types of ASF* vs. *One Type* **Stunting** [OR = 0.39, 95% CI: 0.31–0.49, 409,960 participants, I^2^ = 100%, GRADE = very low] *Three Types of ASF* vs. *One Type* **Stunting** [OR = 0.17, 95% CI: 0.16–0.17, 409,960 participants, I^2^ = 19%, GRADE = very low]
Greater Amount vs. Lesser Amount of ASF	Malawi and Ecuador	A Meta-Analysis of Two Randomized Controlled Trials (RCTs)	**Weight-for-age Z-score** (WAZ) [Mean Difference (MD) = 0.15, 95% CI: 0.00–0.30, 743 participants, I^2^ = 0%, GRADE = moderate] **Stunting** [Relative Risk (RR) = 0.70, 95% CI: 0.55–0.90, 412 participants, I^2^ = 0%, GRADE = low]
	Malawi (*n* = 2) and Ecuador	A Meta-Analysis of Three RCTs	**Height-for-age Z-score (HAZ)** [MD = 0.07, 95% CI: 0.07–0.20, 1017 participants, I^2^ = 0%, GRADE = low] **Weight-for-height Z-score (WHZ)** [MD = −0.09, 95% CI: 0.23–0.05, 1007 participants, I^2^ = 25%, GRADE = low]

**Table 5:** Summary of evidence for provision of complementary food interventions.

Exposure/ Intervention	Location	Evidence Reviewed	Effect/Association
Provision of Complementary Foods vs. Control, SubGroups by Type of CF Provision	Malawi (*n* = 3), Ethiopia (*n* = 3), Niger (*n* = 2), India (*n* = 2), Zambia (*n* = 2), Ecuador (*n* = 2), DRC, Cambodia, Bangladesh, Ghana, Mali, Chad, Pakistan, Nigeria, South Africa, Vietnam, Guinea-Bissau, Brazil, Honduras, China	Updated Evidence from 14 RCTs, 11 cRCTs, and Three Non-RCTs	**Height-for-age Z-score (HAZ)** *Fortified blended foods* [Mean Difference (MD) = 0.25; 95% Confidence Interval (CI): 0.09–0.41, 5820 participants, I^2^ = 93%, GRADE = very low] *Locally produced ready to use supplementary food* [MD = 0.04; 95% CI: 0.02–0.06; 2906 participants; I^2^ = 0%, GRADE = low]
			**Weight-for-height Z-score (WHZ)** *Fortified blended foods* [MD = 0.08; 95% CI: 0.01–0.15; 6966 participants, I^2^ = 56%, GRADE = very low] *Locally produced ready to use supplementary food* [MD = 0.02, 95% CI: 0.00–0.04, 2576 participants, I^2^ = 5%, GRADE = low] *Commercially produced ready to use supplementary food* [MD = 0.04, 95% CI: 0.01–0.07, 2581 participants, I^2^ = 0%, GRADE = low]
			**Weight-for-age Z-score (WAZ)** *Fortified blended foods* [MD = 0.16, 95% CI: 0.03–0.30, 5995 participants, I^2^ = 91%, GRADE = very low] *Locally produced ready to use supplementary food* [MD = 0.03, 95% CI: 0.01–0.05, 2576 participants, I^2^ = 0%, GRADE = low] *Commercially produced ready to use supplementary food* [Relative Risk (RR) = 0.90, 95% CI: 0.83–0.98, 2634 participants, I^2^ = 0%, GRADE = low]
			**Stunting** *Fortified blended foods* [RR = 0.73, 95% CI: 0.56–0.95, 7358 participants, I^2^ = 90%, GRADE = very low] *Commercially produced ready to use supplementary food* [RR = 0.75, 95% CI: 0.61–0.92, 4762 participants, I^2^ = 52%, GRADE = very low]
			**Wasting** *Commercially produced ready to use supplementary food* [RR = 0.75, 95% CI: 0.61–0.92, 4762 participants, I^2^ = 52%, GRADE = very low]
			**Change in Weight** *Locally produced ready to use supplementary food* [MD = 0.03, 95% CI: 0.01–0.05, 2576 participants, I^2^ = 0%, GRADE = low] *Commercially produced ready to use supplementary food* [MD = 0.04, 95% CI: 0.01–0.07, 1911 participants, I^2^—0%, GRADE = very low]
			**Change in Height** *Locally produced ready to use supplementary food* [MD = 0.08, 95% CI: 0.05–0.12, 2576 participants, I^2^ = 0%, GRADE = low] *Commercially produced ready to use supplementary food* [MD = 0.06, 95% CI: 0.00–0.11, 1911 participants, I^2^ = 0%, GRADE = low]
			**Mid-Upper Arm Circumference (MUAC)** *Commercially produced ready to use supplementary food* [MD = 0.20, 95% CI: 0.02–0.38, 670 participants, GRADE = low]
			**Anemia** *Alternative food* [RR = 0.52, 95% CI: 0.33–0.81, 62 participants, GRADE = very low] *Fortified blended food* [RR = 0.74, 95% CI: 0.62–0.87, 5511 participants I^2^ = 74%, GRADE = very low]
			**Hemoglobin** *Alternative food* [Standardized Mean Difference (SMD) = 0.35, 95% CI: 0.02–0.69, 62 participants, GRADE = very low] *Fortified blended food* [SMD = 0.64, 95% CI: 0.29–1.00, 2727 participants, I^2^ = 61%, GRADE = very low]
			**Skin Conditions** *Alternative food* [RR = 0.56, 95% CI: 0.32–0.98, 148 participants, GRADE = moderate]
			**Death** *Commercially produced ready to use supplementary food* [RR = 0.43, 95% CI: 0.20–0.94, 7879 participants, GRADE = very low]
Provision of Complementary Foods vs. Control, SubGroups by Food Secure vs. Insecure Status	Malawi (*n* = 3), Ethiopia (*n* = 3), Niger (*n* = 2), India (*n* = 2), Zambia (*n* = 2), Ecuador (*n* = 2), DRC, Cambodia, Bangladesh, Ghana, Mali, Chad, Pakistan, Nigeria, South Africa, Vietnam, Guinea-Bissau, Brazil, Honduras, China	Updated Evidence from 14 RCTs, 11 cRCTs, and Three Non-RCTs	**Stunting** *Food insecure* [RR = 0.91, 95% CI: 0.83–1.00, 20 895 participants, I^2^ = 80%, GRADE = very low] *Food secure* [RR = 0.62, 95% CI: 0.44–0.88, 3363 participants, I^2^ = 83%, GRADE = very low]
			**Wasting** *Food insecure* [RR = 0.87, 95% CI: 0.81–0.93, 27,987 participants, I^2^ = 12%, GRADE = very low]
			**HAZ** *Food insecure* [MD = 0.18, 95% CI: 0.03–0.33, 20,287 participants, I^2^ = 100%, GRADE = very low]
			**WAZ** *Food insecure* [MD = 0.09, 95% CI: 0.04–0.15, 22,239 participants, I^2^ = 93%, GRADE = very low]
			**Change in Height** *Food insecure* [MD = 0.21, 95% CI: 0.07–0.35, 14,390 participants, I^2^ = 97%, GRADE = very low]
			**MUAC** *Food insecure* [MD = 0.12, 95% CI: 0.05–0.18, 10,140 participants, I^2^ = 60%, GRADE = very low]
			**Hemoglobin Levels** *Food insecure* [SMD = 0.59, 95% CI: 0.04–1.15, 7901 participants, I^2^ = 99%, GRADE = very low] *Food secure* [SMD = 0.49, 95% CI: 0.28–0.71, 863 participants, I^2^ = 0%, GRADE = low]
			**Anemia** *Food insecure* [RR = 0.86, 95% CI: 0.76–0.97, 8322 participants, I^2^ = 91%, GRADE = very low] *Food secure* [RR = 0.64, 95% CI: 0.60–0.67, 3761 participants, I^2^ = 0%, GRADE = very low]
			**Iron Deficiency Anemia** *Food secure* [RR = 0.24, 95% CI: 0.14–0.42, 514 participants, GRADE = low]
			**Diarrhea** *Food secure* [RR = 2.04, 95% CI: 1.07–3.87, 148 participants, GRADE = moderate]
			**Skin Conditions** *Food secure* [RR = 0.56, 95% CI: 0.32–0.98, 148 participants, GRADE = moderate]

**Table 6:** Summary of evidence for small-quantity lipid-based nutrient supplementation.

Exposure/ Intervention	Location	Evidence Reviewed	Effect/Association
Small-quantity lipid-based nutrient supplementation (SQ-LNS) vs. Control	Malawi (*n* = 3), Ghana (*n* = 2), Burkina Faso (*n* = 2), Bangladesh *(n* = 2), Republic of Congo, Madagascar, Zimbabwe, Haiti, Indonesia, Kenya, South Africa	Updated Evidence from Nine Cluster Randomized Controlled Trials (RCTs), Four RCTs, and Three Non-RCTs	**Wasting** [Relative Risk (RR): 0.90, 95% Confidence Interval (CI): 0.82–0.98, 16,976 participants, I^2^ = 0%, GRADE = very low]
			**Mid-upper Arm Circumference** [Mean Difference (MD) = 0.10, 95% CI: 0.03–0.17, 9411 participants, I^2^ = 62% GRADE = very low]
			**Iron Deficiency Anemia** [RR = 0.46, 95% CI: 0.26–0.81, 2643 participants, I^2^ = 90%, GRADE = very low]
			**Mean Diarrhea Episodes** [MD = 0.05, 95% CI: 0.04–0.05, 2556 participants, GRADE = very low]
			**Upper Respiratory Tract Infection** [RR = 0.87, 95% CI: 0.77–0.98, 2556 participants, GRADE = very low]

**Table 7:** Summary of evidence for micronutrient powders.

Exposure/ Intervention	Location	Evidence Reviewed	Effect
Micronutrient Powders (MNP) vs. No Intervention or Placebo	Bangladesh, Brazil, Burkina Faso, Cambodia, China, Colombia, Ethiopia, Ghana, Haiti, India, Indonesia, Kenya, Kyrgyzstan, Lao People’s Democratic Republic, Mali, Pakistan, Philippines, Uganda, Nepal	Updated Evidence from 19 Randomized Controlled Trials	**Hemoglobin** [Standardized Mean Difference (SMD) = 0.72, 95% Confidence Interval (CI): 0.22–1.22, 15 studies, 9089 participants, I^2^ = 99%, GRADE = low]
			**Weight-for-age Z-score** [Mean Difference (MD) = 0.11, 95% CI: 0.02–0.20, 10 studies, 8253 participants, I^2^ = 86%, GRADE = low]
			**Weight-for-height Z-score** [MD = 0.08, 95% CI: 0.03–0.14, 9 studies, 8065 participants, I^2^ = 67%, GRADE = low]

**Table 8:** Summary of evidence for complementary feeding education interventions.

Exposure/ Intervention	Location	Evidence Reviewed	Effect
Complementary Feeding (CF) Education vs. Control, Healthy Children	India (*n* = 11), Bangladesh (*n* = 5), Ethiopia (*n* = 4), Indonesia (*n* = 3), Nepal (*n* = 3), Pakistan (*n* = 3), China (*n* = 2), Kenya (*n* = 2), Uganda (*n* = 2), Brazil (*n* = 2), Cambodia (*n* = 2), Malawi, Guatemala, Dominican Republic, Colombia, Peru, Iran, Somalia	Updated Evidence from 22 Cluster Randomized Controlled Trials (RCTs), 14 Non-RCTs, Nine RCTs, and One Quasi- RCT	**Height-for-age Z-score (HAZ)** [Mean Difference (MD) = 0.20, 95% Confidence Interval (CI): 0.12–0.28, 7457 participants, I^2^ = 85%, GRADE = very low]
			**Weight-for-age Z-score (WAZ)** [MD = 0.18, 95% CI: 0.10–0.27, 5856 participants, I^2^ = 84%, GRADE = very low]
			**Weight-for-height Z-score (WHZ)** [MD = 0.09, 95% CI: 0.01–0.17, 5260 participants, I^2^ = 84%, GRADE = very low]
			**Stunting** [Relative Risk (RR) = 0.90, 95% CI: 0.84–0.96, 25,795 participants, I^2^ = 60%, GRADE = very low]
			**Underweight** [RR = 0.87, 95% CI: 0.78–0.97, 23,176 participants, I^2^ = 73%, GRADE = very low]
			**Severe Underweight** [RR = 0.39, 95% CI: 0.16–0.93, 816 participants, 1 study, GRADE = very low]
			**Severe Wasting** [RR = 0.14, 95% CI: 0.03–0.74, 906 participants, I^2^ = 0%, GRADE = very low]
			**Change in Weight** [MD =0.20, 95% CI: 0.07–0.34, 4176 participants, GRADE = very low]
			**Respiratory Illness** [RR = 0.73, 95% CI: 0.60–0.90, 1588 participants, I^2^ = 17%, GRADE = very low]
			**Low Birth Weight** [RR = 0.47, 95% CI: 0.26–0.85, 1049 participants, I^2^ = 0%, GRADE = very low]
CF Education vs. Control, Healthy Children, SubGroups by Food Secure vs. Insecure Status	India (*n* = 11), Bangladesh (*n* = 5), Ethiopia (*n* = 4), Indonesia (*n* = 3), Nepal (*n* = 3), Pakistan (*n* = 3), China (*n* = 2), Kenya (*n* = 2), Uganda (*n* = 2), Brazil (*n* = 2), Cambodia (*n* = 2), Malawi, Guatemala, Dominican Republic, Colombia, Peru, Iran, Somalia	Updated Evidence from 22 cRCTs, 14 Non-RCTs, Nine RCTs, and One Quasi- RCT	**HAZ** *Food secure* [MD = 0.39, 95% CI: 0.09–0.68, 828 participants, I^2^ = 79%, GRADE = very low] *Food insecure* [MD = 0.16, 95% CI: 0.08–0.24, 6629 participants, I^2^ = 84%, GRADE = very low]
			**WAZ** *Food secure* [MD = 0.24, 95% CI: 0.09–0.38, 376 participants, I^2^ = 0%, GRADE = moderate] *Food insecure* [MD = 0.17, 95% CI: 0.09–0.26, 5480 participants, I^2^ = 86%, GRADE = very low]
			**Wasting** *Food secure* [RR = 0.78, 95% CI: 0.62–0.98, 12,386 participants, I^2^ = 0%, GRADE = very low]
			**Stunting** *Food insecure* [RR = 0.93, 95% CI: 0.89–0.98, 12,852 participants, I^2^ = 31%, GRADE = very low]
			**Underweight** *Food insecure* [RR = 0.90, 95% CI: 0.81–1.00, 10,819 participants, I^2^ = 71%, GRADE = very low] *Food secure* [RR = 0.71, 95% CI: 0.53–0.95, 12,357 particpants, I^2^ = 30%, GRADE = very low]
			**Change in Weight** *Food insecure* [MD = 0.20, 95% CI: 0.05–0.35, 3872 participants, I^2^ = 91%, GRADE = very low]
			**Change in Height** *Food secure* [MD = 0.90, 95% CI: 0.26–1.55, 304 participants, I^2^ = 0%, GRADE = very low]
			**Anemia** *Food insecure* [RR = 0.74, 95% CI: 0.62–0.88, 432 participants, GRADE = very low]
			**Diarrhea** *Food secure* [RR = 0.67, 95% CI: 0.51–0.90, 397 participants, GRADE = very low]
			**Respiratory Illness** *Food secure* [RR = 0.63, 95% CI: 0.46–0.85, 397 participants, GRADE = very low]
CF Education vs. Control, Undernourished Children	Iran	RCT	**WHZ** [MD = 0.34, 95% CI: 0.27–0.41, 100 participants, GRADE = very low]
			**WAZ** [MD = 0.35, 95% CI: 0.29–0.41, 100 participants, GRADE = very low]
			**HAZ** [RR = 0.35, 95% CI: 0.29–0.41, 100 participants, GRADE = very low]

## Data Availability

The data presented in this study are available in Supplementary Documents.

## Supplementary Materials

The following supporting information can be downloaded at: https://www.mdpi.com/article/10.3390/nu15133041/s1, Supplementary File S1: S1.1. Objective; S1.2. Methods; S1.3. Search Strategies; S1.4. Prisma Diagram; S1.5. List of Included Publications; S1.6. Table of Primary Outcome Results; S1.7.–S1.15. Forest Plots; S1.16. GRADE Assessments. Supplementary File S2: S2.1. Objective; S2.2. Methods; S2.3. Medline Search Strategy; S2.4. Prisma Diagram; S2.5. List of Included Publications; S2.6. Summary of Narrative Results for FV and NPS; S2.7. GRADE Assessments for FV and NPS; S2.8. Summary of Key Findings for ASF; S2.9. Forest Plots for ASF; S2.10. GRADE Assessments for ASF. Supplementary File S3: S3.1. Objective; S3.2. Methods; S3.3. Prisma Diagram; S3.4. List of Included Studies; S3.5. GRADE Assessments; S3.6. Forest Plots. Supplementary File S4: S4.1. Objective; S4.2. Methods; S4.3. Prisma Diagram; S4.4. List of Included Publications; S4.5. Summary of Findings Tables; S4.6. Forest Plots.
