# Supplementary material for: Dietary Strategies for Complementary Feeding between 6 and 24 Months of Age: The Evidence
Source: Nutrients. 2023 Jul 5;15(13):3041. doi: 10.3390/nu15133041 (PMC10346638; doi:10.3390/nu15133041)
Supplement: Supplementary file 1 [file nutrients-15-03041-s001.zip › Supplementary File S1.pdf]

SUPPLEMENTARY FILE S1 – The Effect of Consumption of Animal Milk Compared to Infant Formula for Non-Breastfed/Mixed-Fed Infants 6-11 Months of Age: A Systematic Review and Meta-Analysis

Reference: Ehrlich, J.M.; Catania, J.; Zaman, M.; Smith, E.T.; Smith, A.; Tsistinas, O.; Bhutta, Z.A.; Imdad, A. The Effect of Consumption of Animal Milk Compared to Infant Formula for Non-Breastfed/Mixed-Fed Infants 6–11 Months of Age: A Systematic Review and Meta-Analysis. Nutrients 2022, 14, 488. https://doi.org/10.3390/nu14030488

Table of Contents

S1.1. Objective ***.......... 2***

S1.2. Methods ***.......... 2***

S1.3. Search Strategies ***.......... 4***

S1.4. Prisma Diagram ***.......... 7***

S1.5. List of publications of included studies ***.......... 8***

S1.6. Table of primary outcome results at different time points ***.......... 10***

S1.7. Forest plot for effect of animal milk vs formula milk intake in infants 6-11 months of age on anemia ***.......... 10***

S1.8. Forest plot for effect of animal milk vs formula milk intake in infants 6-11 months of age on gastrointestinal blood loss ***.......... 11***

S1.9. Forest plot for effect of animal milk vs formula milk intake in infants 6-11 months of age on weight for age ***.......... 11***

S1.10. Forest plot for effect of animal milk vs formula milk intake in infants 6-11 months of age on length for age ***.......... 12***

S1.11. Table of secondary outcome results ***.......... 12***

S1.12. Forest plot for effect of animal milk vs formula milk intake in infants 6-11 months of age on blood hemoglobin levels ***.......... 13***

S1.13. Forest plot for effect of animal milk vs formula milk intake in infants 6-11 months of age on iron deficiency anemia ***.......... 13***

S1.14. Forest plot for effect of animal milk vs formula milk intake in infants 6-11 months of age on serum ferritin levels ***.......... 14***

S1.15. Forest plot for effect of animal milk vs formula milk intake in infants 6-11 months of age on hemoglobin concentration in the stool ***.......... 14***

S1.16. GRADE Evidence Profiles for Certainty Assessment of primary outcomes and selected secondary outcomes ***.......... 15***

S1.1. Objective

For non-breastfed or mixed-fed (breastmilk and formula) infants 6–11months of age, is the consumption of animal milk, compared to infant formula, associated with beneficial or adverse outcomes for health and development?

S1.2. Methods

S1.2.1. Criteria for considering studies for this review

We included individual and cluster randomized trials, quasi-randomized experimental design studies, and prospective and retrospective cohort studies with a control group. The study population was apparently healthy infants 6–11 months of age who were non-breastfed or mixed fed (breast milk and formula) irrespective of gestational age and birth weight. We excluded studies with participants who have chronic diseases such as bronchopulmonary dysplasia, genetic disorders, aerodigestive problems, or congenital anomalies.

The intervention of interest was the use of animal milk in infants 6–11 months of age. We included studies in which animal milk was the main milk drink as defined by study authors, or more than 50% of the infant's milk intake was animal milk. We included studies irrespective of whether the animal milk was boiled, pasteurized, or unpasteurized, or if the animal milk was full-fat, reduced-fat, or skim milk. The comparison group in the included studies was formula feeding or mixed feeding (i.e., breastfeeding and formula feeding). We included studies irrespective of the type of formula used; this could include cow's milk-based formula, partially or extensively hydrolyzed formula, or plant-based formulas such as soy formula. The Food and Drug Administration (FDA) of the United States Federal Food, Drug, and Cosmetic Act (FFDCA) defines infant formula as "a food which purports to be or is represented for special dietary use solely as a food for infants by reason of its simulation of human milk or its suitability as a complete or partial substitute for human milk".

The primary outcomes of interest were: any anemia (dichotomous, as defined by authors); gastrointestinal blood loss (dichotomous, based on stool occult testing); weight for- age (continuous, kg or Z scores); length-for-age (continuous, cm or Z scores); and weight-for-length Z score. The secondary outcomes were: iron deficiency anemia (dichotomous); serum iron level (continuous); serum ferritin level (continuous); stool hemoglobin concentration (continuous); blood hemoglobin concentration (continuous); serum triglycerides (continuous); diarrhea (dichotomous, defined as >3 loose stools per day); constipation (dichotomous, defined <3 bowel movements per week); pneumonia (dichotomous as defined by authors); allergy (dichotomous IgE-Mediated and non-IgE Mediated and mixed); obesity (dichotomous); overweight (dichotomous); and neurodevelopmental outcomes (continuous). We considered the time of follow-up for these outcomes at 7 months, 9 months, and 12 months as the longest follow up.

S1.2.2. Search methods for identification of studies

We conducted systematic electronic searches on multiple databases, including PubMed, EMBASE, the Cochrane Central Register for Controlled Trials,Web of Science, CINHAL, Scopus, and WHO Global Index Medicus. There were no restrictions applied to the searches based on outcomes, study design, publication status, publication date, or language. The last date of the search for publication was 17 November 2021. We conducted an update to the search 1 April 2022. The search strategy used can be found in section A.3.

Searches from all the databases were combined in bibliographic software (EndNote), and duplicates were removed. Two authors (either JE, AI, JC, or MZ) screened the titles using the software Covidence. Two authors (JE, JC, or MZ) independently extracted the data from the included studies and compared their findings. Any conflict was resolved by discussion and with the help of the senior author (AI), if needed. The risk of bias was assessed using the Cochrane risk of bias tool-2 (ROB 2.0) for RCTs and using the Cochrane risk of bias in non-randomized studies (ROBINS-I) tool for non-randomized studies. The risk of bias was assessed by two authors for each study included in a pooled analysis.

S1.2.3. Data collection and analysis

Meta-analyses were conducted when data was available from more than one study and clinical and methodological homogeneity was present in the included studies. Dichotomous outcomes were pooled to obtain an average relative risk (RR). For continuous outcomes, we pooled the data to obtain a standardized mean difference (SMD). All study-level and average effect sizes are reported alongside their 95% confidence intervals (CIs). We used the generic inverse variance weighting method for meta-analysis. We used a random-effects model for meta-analysis, given that there might be heterogeneity in effects due to variability in the study populations and interventions used. We analyzed randomized controlled trials and cohort studies separately. We used RevMan software for the statistical analysis.

Statistical heterogeneity in the pooled analysis was assessed using Tau^2, X^2, and I^2 statistics, and it was considered substantial if the P-value for the Chi^2 test was less than 0.10, the I^2 value exceeded 50%, and inspection of forest plots showed substantial variability in the effect of the intervention.

We aimed to assess small study and publication bias using funnel plots and regression tests. However, the number of included studies in the meta-analysis was less than ten, so no testing was performed for publication and small study bias (per the analysis protocol).

We assessed the overall certainty of evidence for the effect of the intervention on each primary outcome and select secondary outcomes using the Grading of Recommendations Assessment, Development, and Evaluation (GRADE) using the software GradePro. We rated the overall body of evidence to certainty level as very low (we have very little confidence in the effect estimate), low (we have limited confidence in the effect estimate), moderate (we have moderate confidence in the effect estimate; the true effect is likely close to the estimate of the effect), or

high (we have high confidence that the true effect lies close to that of the estimate of the effect). We present the results of the GRADE assessment in the form of GRADE Evidence Profiles for the primary outcomes and the following secondary outcomes: blood hemoglobin concentration, iron deficiency anemia, constipation, diarrhea, and neurodevelopmental outcomes.

We aimed to compare effects for the following subgroups when possible: age group: age of initiation at 7 months vs. 9 months; country, low- and middle-income country vs. high-income country; type of Feeding: non-breastfeeding vs. mixed feeding; and type of animal milk, cow, goat, buffalo, camel, or sheep. Finally, we considered the following sensitivity analyses: studies with a high overall risk of bias excluded; type of model: and random vs. fixed-effect meta-analysis model.

S1.3. Search Strategies

Text S1: Search Strategy for electronic data bases
Complementary Feeding Strategies

PubMed
((("cow milk*"[tiab] OR "cow's milk*"[tiab] OR "cows milk*"[tiab] OR "bovine milk*"[tiab] OR "bovine’s milk*"[tiab] OR "goat milk*"[tiab] OR "goat's milk*"[tiab] OR "goats milk*"[tiab] OR "caprine milk*"[tiab] OR "buffalo milk*"[tiab] OR "buffalo's milk*"[tiab] OR "camel milk*"[tiab] OR "camel’s milk*"[tiab]OR "camels milk*"[tiab] OR "sheep milk*"[tiab] OR "sheep’s milk*"[tiab] OR "ewe milk*"[tiab] OR "ewe’s milk*"[tiab] OR "ewes milk*"[tiab] OR "ovine milk*"[tiab]) AND ("Infant Formula"[Mesh] OR formula*[tiab])) AND ("Infant"[Mesh] OR infant*[tiab] OR infancy[tiab] OR baby[tiab] OR babies[tiab] OR neonat*[tiab] OR "neo nat*"[tiab] OR newborn*[tiab] OR "new born*"[tiab] OR "newly born*"[tiab] OR "Child"[Mesh] OR child*[tiab] OR youth[tiab] OR juvenile*[tiab])) NOT ("Animals"[Mesh] NOT ("Animals"[Mesh] AND "Humans"[Mesh]))

CINAHL

(TI ("cow milk*" OR "cow's milk*" OR "cows milk*" OR "bovine milk*" OR "bovine's milk*" OR "goat milk*" OR "goat's milk*" OR "goats milk*" OR "caprine milk*" OR "buffalo milk*" OR "buffalo's milk*" OR "camel milk*" OR "camel’s milk*" OR "camels milk*" OR "sheep milk*" OR "sheep’s milk*" OR "ewe milk*" OR "ewe’s milk*" OR "ewes milk*" OR "ovine milk*") OR AB ("cow milk*" OR "cow's milk*" OR "cows milk*" OR "bovine milk*" OR "bovine's milk*" OR "goat milk*" OR "goat's milk*" OR "goats milk*" OR "caprine milk*" OR "buffalo milk*" OR "buffalo's milk*" OR "camel milk*" OR "camel’s milk*" OR "camels milk*" OR "sheep milk*" OR "sheep’s milk*"OR "ewe milk*" OR "ewe’s milk*" OR "ewes milk*" OR "ovine milk*")) AND ((MH "Infant Formula") OR (TI formula* OR AB formula*)) AND (((MH "Child") OR (MH "Infant+")) OR (TI (infant* OR infancy OR baby OR babies OR neonat* OR "neo nat*" OR newborn* OR "new born*" OR "newly born*" OR child* OR youth OR juvenile*) OR AB (infant* OR infancy OR baby OR babies OR neonat* OR "neo nat*" OR newborn* OR "new born*" OR

"newly born*" OR child* OR youth OR juvenile*))) NOT (((MH "Animals+") OR (MH "Animal Studies") OR (TI "animal model*")) NOT (MH "human"))

Scopus

((TITLE-ABS ("cow milk*" OR "cows milk*" OR "bovine milk*" OR "bovine's milk*" OR "goat milk*" OR "goats milk*" OR "caprine milk*" OR "buffalo milk*" OR "buffalo's milk*" OR "camel milk*" OR "camels milk*" OR "sheep milk*" OR "sheep's milk*" OR "ewe milk*" OR "ewes milk*" OR "ovine milk*")) AND (TITLE-ABS (formula*)) AND (TITLEABS (infant* OR infancy OR baby OR babies OR neonat* OR "neonat*" OR newborn* OR "new born*" OR "newly born*" OR child* OR youth OR juvenile*)) AND NOT INDEX (medline))

Embase

1 'cow milk'/exp OR 'goat milk'/exp OR 'buffalo milk'/exp OR 'camel milk'/exp OR 'sheep milk'/exp
2 'cow milk*':ti,ab OR 'cows milk*':ti,ab OR 'cow s milk*':ti,ab OR 'bovine milk*':ti,ab OR 'bovine s milk*':ti,ab OR 'goat milk*':ti,ab OR 'goat s milk*':ti,ab OR 'goats milk*':ti,ab OR 'caprine milk*':ti,ab OR 'buffalo milk*':ti,ab OR 'buffalo s milk*':ti,ab OR 'camel milk*':ti,ab OR 'camel s milk*':ti,ab OR 'camels milk*':ti,ab OR 'sheep milk*':ti,ab OR 'sheep s milk*':ti,ab OR 'ewe milk*':ti,ab OR 'ewes milk*':ti,ab OR 'ewe s milk*':ti,ab OR 'ovine milk*':ti,ab
3 #1 OR #2
4 'artificial milk'/exp
5 formula*:ti,ab
6 #4 OR #5
7 'infant'/exp OR 'infancy'/exp OR 'baby'/exp OR 'newborn'/exp OR 'child'/exp OR 'juvenile'/exp
8 infant*:ti,ab OR infancy:ti,ab OR baby:ti,ab OR babies:ti,ab OR neonat*:ti,ab OR 'neo nat*':ti,ab OR newborn*:ti,ab OR 'new born*':ti,ab OR 'newly born*':ti,ab OR child*:ti,ab OR youth:ti,ab OR juvenile*:ti,ab
9 #7 OR #8
10 #3 AND #6 AND #9
11 #10 NOT ([animals]/lim NOT [humans]/lim)

Cochrane CENTRAL

1 cow* NEXT milk*:ti,ab OR bovine* NEXT milk*:ti,ab OR goat* NEXT milk*:ti,ab OR caprine NEXT milk*:ti,ab OR buffalo* NEXT milk*:ti,ab OR camel* NEXT milk*:ti,ab OR sheep* NEXT milk*:ti,ab OR ewe* NEXT milk*:ti,ab OR ovine NEXT milk*:ti,ab
2 MeSH descriptor: [Infant Formula] explode all trees
3 formula*:ti,ab
4 #2 OR #3
5 MeSH descriptor: [Child] explode all trees
6 MeSH descriptor: [Infant] explode all trees

7 infant*:ti,ab OR infancy:ti,ab OR baby:ti,ab OR babies:ti,ab OR neonat*:ti,ab OR neo NEXT nat*:ti,ab OR newborn*:ti,ab OR new NEXT born*:ti,ab OR newly NEXT born:ti,ab OR child*:ti,ab OR youth:ti,ab OR juvenile*:ti,ab
8 #5 OR #6 OR #7
9 MeSH descriptor: [Animals] explode all treMeSH descriptor: [Infant] explode all trees
10 MeSH descriptor: [Humans] explode all trees
11 (#9 NOT(#9 AND #10))
12 #1 AND #4 AND #8
13 #12 NOT #11
14 "accession number" near pubmed
15 #13 NOT #14

Web of Science: 89 results

1 TI=(“cow* milk*" OR "bovine* milk*" OR "goat* milk*" OR "caprine milk*" OR "buffalo* milk*" OR “camel* milk*" OR "sheep* milk*" OR "ovine milk*")
2 AB=(“cow* milk*" OR "bovine* milk*" OR "goat* milk*" OR "caprine milk*" OR "buffalo* milk*" "camel* milk*" OR "sheep* milk*" OR "ovine milk*")
3 #2 OR #1
4 TI=(formula*)
5 AB=(formula*)
6 #5 OR #4
7 TI=(infant* OR infancy OR baby OR babies OR neonat* OR "neo nat*" OR newborn* OR "new* born*" OR child* OR youth OR juvenile*)
8 AB=(infant* OR infancy OR baby OR babies OR neonat* OR "neo nat*" OR newborn* OR "new* born*" OR child* OR youth OR juvenile*)
9 #8 OR #7
10 #9 AND #6 AND #3
11 #9 AND #6 AND #3 Exclude Medline

LILACS

ti:("cow milk*" OR "cow's milk*" OR "cows milk*" OR "bovine milk*" OR "bovine's milk*" OR "goat milk*" OR "goat's milk*" OR "goats milk*" OR "caprine milk*" OR "buffalo milk*" OR "buffalo's milk*" OR "camel milk*" OR "camel’s milk*" OR "camels milk*" OR "sheep milk*" OR "sheep’s milk*" OR "ewe milk*" OR "ewe’s milk*" OR "ewes milk*" OR "ovine milk*") OR (ab:("cow milk*" OR "cow's milk*" OR "cows milk*" OR "bovine milk*" OR "bovine's milk*" OR "goat milk*" OR "goat's milk*" OR "goats milk*" OR "caprine milk*" OR "buffalo milk*" OR "buffalo's milk*" OR "camel milk*" OR "camel’s milk*" OR "camels milk*" OR "sheep milk*" OR "sheep’s milk*" OR "ewe milk*" OR "ewe’s milk*" OR "ewes milk*" OR "ovine milk*")) AND (((ti:(formula*)) OR (ab:(formula*))) OR ((mh:("infant formula")))) AND (((ti:(infant* OR infancy OR baby OR babies OR neonat* OR "neo nat*" OR newborn* OR "new born*" OR "newly born*" OR child* OR youth OR juvenile*)) OR (ab:(infant* OR infancy OR baby OR babies OR neonat* OR "neo nat*" OR newborn* OR "new born*" OR "newly born*" OR child* OR youth

OR juvenile*))) OR ((mh:("infant")) OR ((mh:("child"))))) AND NOT ((mh:("animals")) AND NOT ((mh:("animals")) AND ((mh:("humans")))))

Global Index Medicus

ti:("cow milk*" OR "cow's milk*" OR "cows milk*" OR "bovine milk*" OR "bovine's milk*" OR "goat milk*" OR "goat's milk*" OR "goats milk*" OR "caprine milk*" OR "buffalo milk*" OR "buffalo's milk*" OR "camel milk*" OR "camel’s milk*" OR "camels milk*" OR "sheep milk*" OR "sheep’s milk*" OR "ewe milk*" OR "ewe’s milk*" OR "ewes milk*" OR "ovine milk*") OR (ab:("cow milk*" OR "cow's milk*" OR "cows milk*" OR "bovine milk*" OR "bovine's milk*" OR "goat milk*" OR "goat's milk*" OR "goats milk*" OR "caprine milk*" OR "buffalo milk*" OR "buffalo's milk*" OR "camel milk*" OR "camel’s milk*" OR "camels milk*" OR "sheep milk*" OR "sheep’s milk*" OR "ewe milk*" OR "ewe’s milk*" OR "ewes milk*" OR "ovine milk*")) AND (((ti:(formula*)) OR (ab:(formula*))) OR ((mh:("infant formula")))) AND (((ti:(infant* OR infancy OR baby OR babies OR neonat* OR "neo nat*" OR newborn* OR "new born*" OR "newly born*" OR child* OR youth OR juvenile*)) OR (ab:(infant* OR infancy OR baby OR babies OR neonat* OR "neo nat*" OR newborn* OR "new born*" OR "newly born*" OR child* OR youth OR juvenile*))) OR ((mh:("infant")) OR ((mh:("child"))))) AND NOT ((mh:("animals")) AND NOT ((mh:("animals")) AND ((mh:("humans")))))AND (db:("WPRIM" OR "IMEMR" OR "IMSEAR" OR "AIM")

S1.4. Prisma Diagram

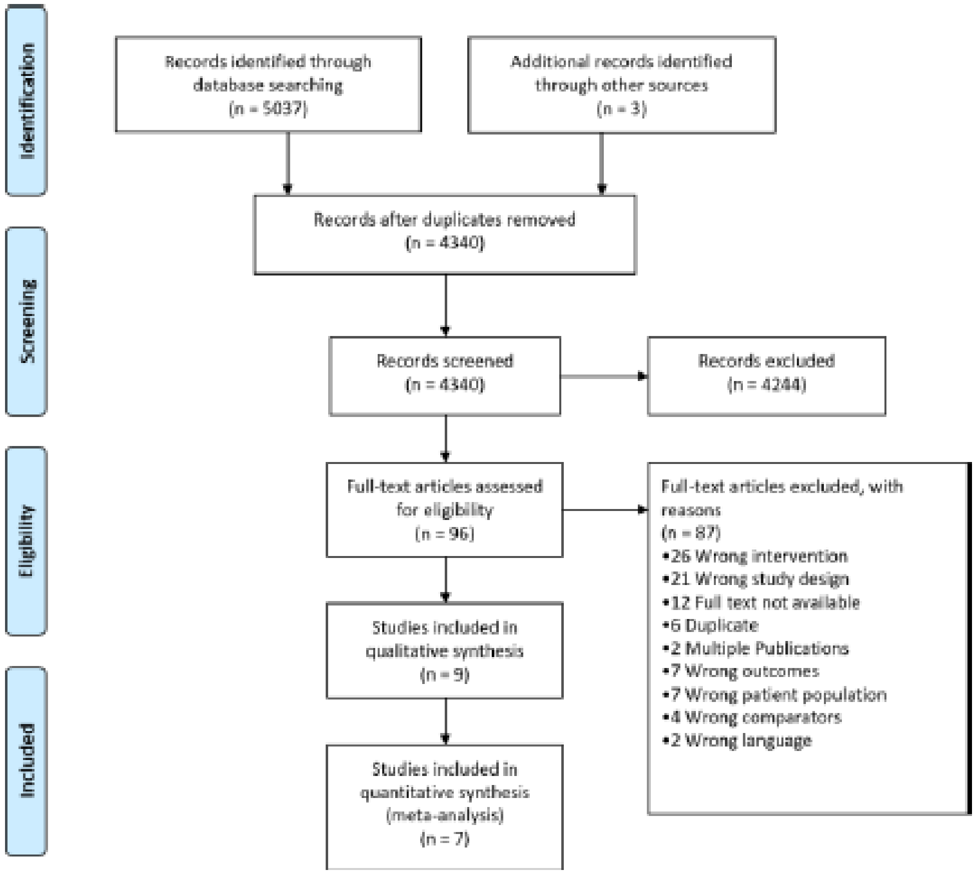

S1.5. List of publications of included studies

Title	Authors	Published Year	Merged Citation Title
Cow milk feeding in infancy: gastrointestinal blood loss and iron nutritional status.	Fomon SJ; Ziegler EE; Nelson SE; Edwards BB	1981	
Iron status and intake of older infants	Fuchs, G. J.; Farris, R. P.; DeWier, M.;	1993	Fuchs G, DeWier M, Hutchinson S, Sundeen M, Schwartz S, Suskind R. Gastrointestinal blood loss in older infants: impact of cow milk versus formula. J Pediatr Gastroenterol Nutr. 1993 Jan;16(1):4-9<br><br>Fuchs GJ, Farris RP, DeWier M, et al. Iron status and intake of older infants fed formula vs cow milk with cereal. Am J Clin Nutr 1993;58:343-8.<br><br>Fuchs, G. J. Clemens, R. A. Hutchinson, S. W. et al, Growth of older infants fed low-fat formula: Nutrition Research, Volume 16, Issue 3, 1996, Pages 391-400.
Cow milk feeding in infancy: further observations on blood loss from the gastrointestinal tract	Ziegler, E. E.; Fomon, S. J.; Nelson, S. E.; Rebouche, C. J.; Edwards, B. B.; Rogers, R. R.; Lehman, L. J.	1990	
Consequences of starting whole cow milk at 6 months of age	Tunnessen, W. W., Jr.; Oski, F. A.	1987	

Iron fortified follow on formula from 9 to 18 months improves iron status but not development or growth: a randomised trial	Morley, R.; Abbott, R.; Fairweather-Tait, S.; MacFadyen, U.; Stephenson, T.; Lucas, A.	1999	
Iron status of one-year-olds and association with breast milk, cow's milk or formula in late infancy	Thorisdottir, A. V.; Ramel, A.; Palsson, G. I.; Tomassson, H.; Thorsdottir, I.	2013	
Prevalence of iron deficiency in 12-mo-old infants from 11 European areas and influence of dietary factors on iron status (Euro-Growth study)	Male, C.; Persson, L. A.; Freeman, V.; Guerra, A.; van't Hof, M. A.; Haschke, F.	2001	
Comparative Metabolic Study of Older Infants Fed Infant Formula, Transition Formula, or Whole Cows Milk	Fuchs, G. J.; Gastanaduy, A. S.; Suskind, R. M.	1992	
Effects on childhood body habitus of feeding large volumes of cow or formula milk compared with breastfeeding in the latter part of infancy	Hopkins, D.; Steer, C. D.; Northstone, K.; Emmett, P. M.	2015	

S1.6. Table of primary outcome results at different time points

Outcome	Time Point	No. of Studies	Study Type	Relative Risk	95% CI
Anemia	9 months	1	RCT	0.59	0.03, 11.92
	12 months	1	Cohort	2.26	1.15, 4.43
		2	RCT	9.00	0.38, 214.20
Gastrointestinal Blood Loss	7 months	1	Cohort	1.52	0.73, 3.16
		1	RCT	2.78	0.83, 9.25
	9 months	1	RCT	3.14	0.98, 10.04
Weight-for-age	12 months	1	RCT	0.00	−0.45, 0.45
Length-for-age	12 months	1	RCT	−0.14	−0.59, 0.31

S1.7. Forest plot for effect of animal milk vs formula milk intake in infants 6-11 months of age on anemia

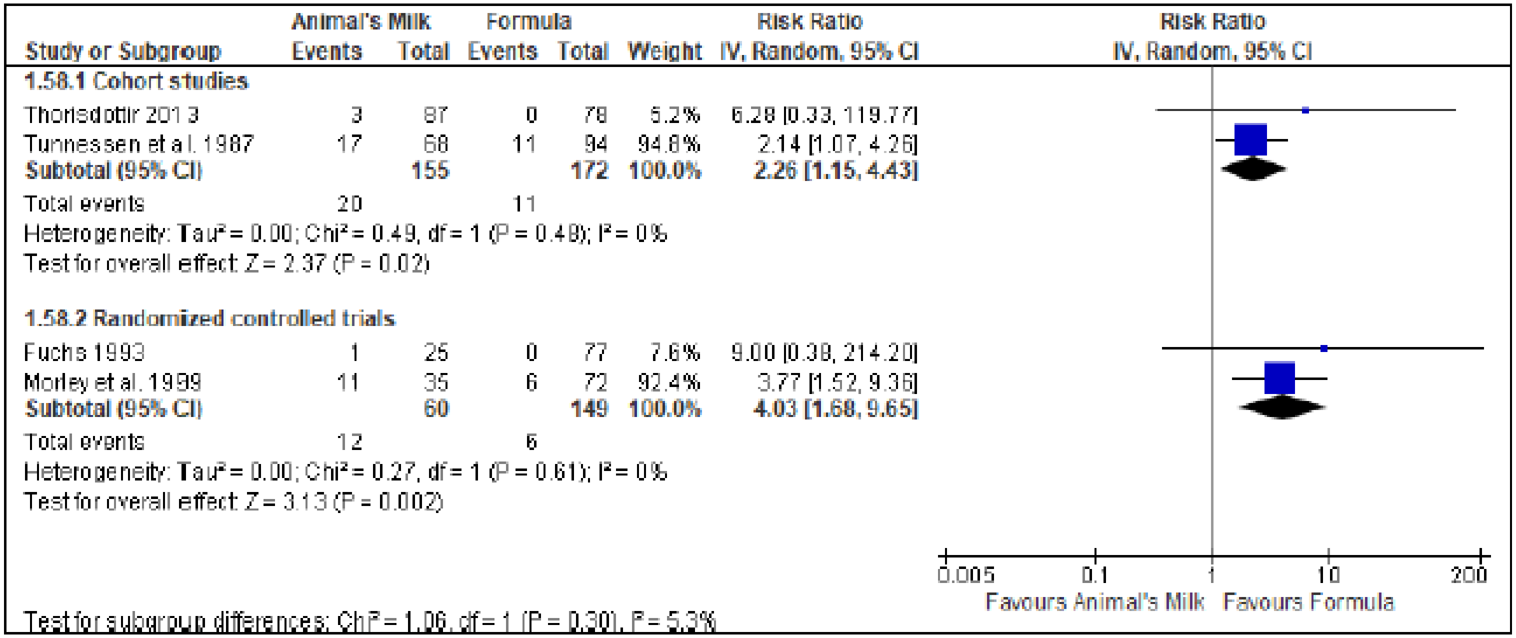

S1.8. Forest plot for effect of animal milk vs formula milk intake in infants 6-11 months of age on gastrointestinal blood loss

Study or Subgroup	Animal's Milk Events	Animal's Milk Total	Formula Events	Formula Total	Weight	Risk Ratio IV, Random, 95% CI
**1.59.1 Cohort study**						
Fomon et al. 1981	26	60	6	21	100.0%	1.52 [0.73, 3.16]
**Subtotal (95% CI)**		**60**		**21**	**100.0%**	**1.52 [0.73, 3.16]**
Total events	26		6			
Heterogeneity: Not applicable						
Test for overall effect: Z = 1.11 (P = 0.27)						
**1.59.2 Randomized trial**						
Ziegler et al. 1990	9	21	3	22	100.0%	3.14 [0.98, 10.04]
**Subtotal (95% CI)**		**21**		**22**	**100.0%**	**3.14 [0.98, 10.04]**
Total events	9		3			
Heterogeneity: Not applicable						
Test for overall effect: Z = 1.93 (P = 0.05)						

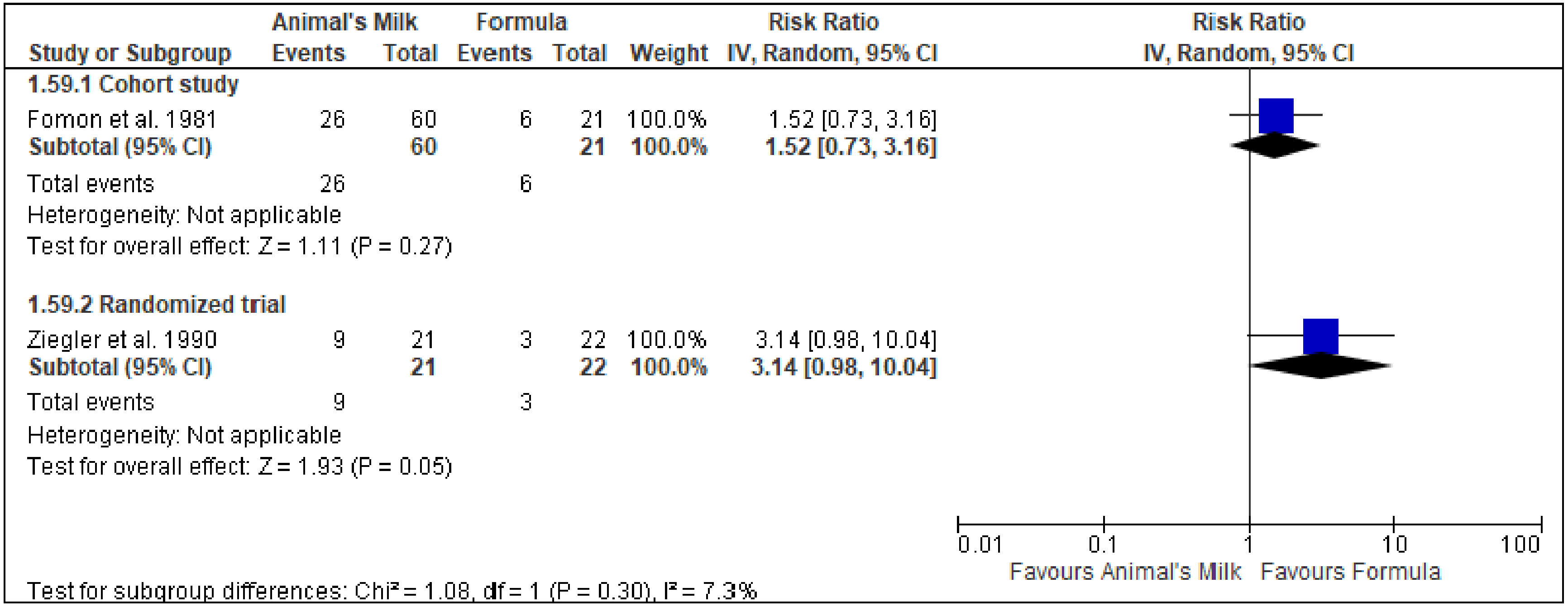

Test for subgroup differences: Chi² = 1.08, df = 1 (P = 0.30), I² = 7.3%

S1.9. Forest plot for effect of animal milk vs formula milk intake in infants 6-11 months of age on weight for age

Study or Subgroup	Std. Mean Difference	SE	Animal's Milk Total	Formula Total	Weight	Std. Mean Difference IV, Random, 95% CI
**1.60.2 Randomized trials**						
Fuchs 1993	0.0026	0.2306	25	76	22.8%	0.00 [-0.45, 0.45]
Fuchs et al. 1992	-0.6394	0.419	9	18	7.8%	-0.64 [-1.46, 0.18]
Morley et al. 1999	0.0359	0.0999	160	268	69.4%	0.04 [-0.16, 0.23]
**Subtotal (95% CI)**			**194**	**362**	**100.0%**	**-0.02 [-0.26, 0.21]**
Heterogeneity: Tau² = 0.01; Chi² = 2.46, df = 2 (P = 0.29); I² = 19%						
Test for overall effect: Z = 0.20 (P = 0.84)						
**Total (95% CI)**			**194**	**362**	**100.0%**	**-0.02 [-0.26, 0.21]**
Heterogeneity: Tau² = 0.01; Chi² = 2.46, df = 2 (P = 0.29); I² = 19%						
Test for overall effect: Z = 0.20 (P = 0.84)						
Test for subgroup differences: Not applicable						

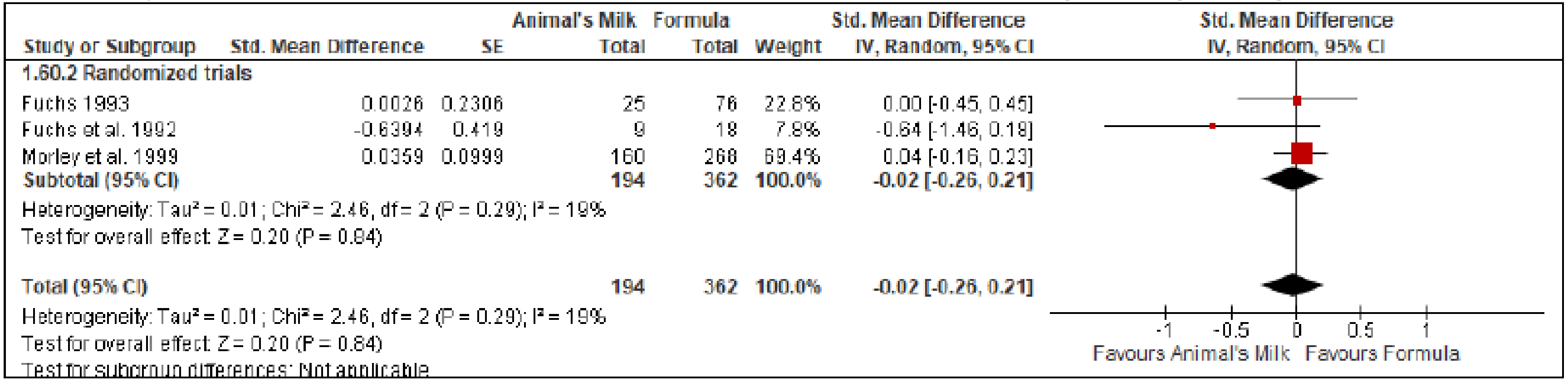

S1.10. Forest plot for effect of animal milk vs formula milk intake in infants 6-11 months of age on length for age

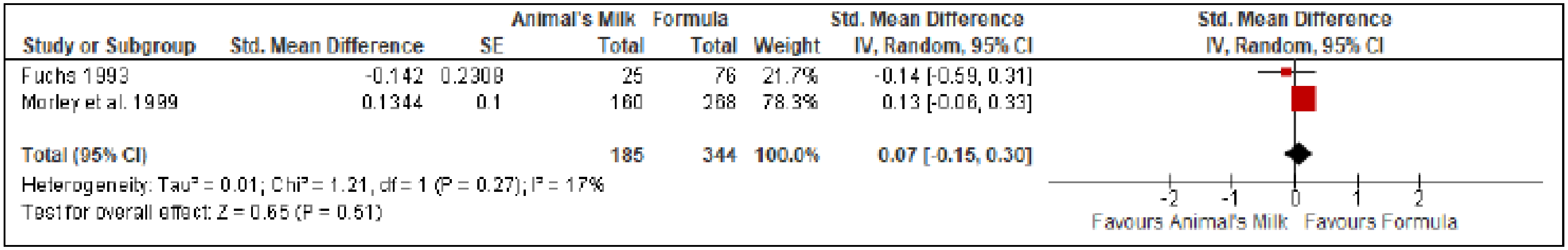

S1.11. Table of secondary outcome results

Outcome	No. of Studies	Type of Studies	Total Participants	SMD/RR (95% CI)	I^2
Iron deficiency anemia	2	Cohort	327	RR = 2.26 (1.15, 4.43)	0%
Blood ferritin at longest follow up	1	Cohort	165	SMD = −0.81 (−1.13, −0.49)	NA
	3	RCT	406	SMD = −0.30 (−0.94,0.34)	85%
Hemoglobin concentration in the stool	2	RCT	228	SMD = 0.22 (−0.16, 0.59)	41%
Hemoglobin concentration in the blood	2	Cohort	246	SMD = −0.37 (−0.78, −0.05)	0%
	3	RCT	250	SMD = −0.32 (−0.59, −0.05)	
Serum iron level	1	Cohort	43	SMD = −0.13 (−0.73, 0.46)	NA
Diarrhea	1	Cohort	167	RR = 1.86 (1.05–33.1)	NA
Constipation	1	Cohort	167	RR = 3.31 (0.89, 12.37)	NA
Neurodevelopmental outcome	1	RCT	428	SMD = 0.18 (−0.02, 0.37)	NA

Footnotes: RR, relative risk; and SMD, Standardized mean difference.

S1.12. Forest plot for effect of animal milk vs formula milk intake in infants 6-11 months of age on blood hemoglobin levels

Study or Subgroup	Std. Mean Difference	SE	Animal's Milk Total	Formula Total	Weight	Std. Mean Difference IV, Random, 95% CI
**1.61.1 Cohort studies**						
Fomon et al. 1981	-0.1006	0.2578	61	20	39.1%	-0.10 [-0.61, 0.40]
Thorisdottir 2013	-0.536	0.1588	87	78	60.9%	-0.54 [-0.85, -0.22]
**Subtotal (95% CI)**			**148**	**98**	**100.0%**	**-0.37 [-0.78, 0.05]**
Heterogeneity: Tau² = 0.05; Chi² = 2.07, df = 1 (P = 0.15); I² = 52%						
Test for overall effect: Z = 1.72 (P = 0.09)						
**1.61.2 Randomized trials**						
Fuchs 1993	-0.3889	0.2297	26	74	35.8%	-0.39 [-0.84, 0.06]
Morley et al. 1999	-0.351	0.2075	35	72	43.9%	-0.35 [-0.76, 0.06]
Ziegler et al. 1990	-0.1289	0.3054	21	22	20.3%	-0.13 [-0.73, 0.47]
**Subtotal (95% CI)**			**82**	**168**	**100.0%**	**-0.32 [-0.59, -0.05]**
Heterogeneity: Tau² = 0.00; Chi² = 0.50, df = 2 (P = 0.78); I² = 0%						
Test for overall effect: Z = 2.32 (P = 0.02)						
Test for subgroup differences: Chi² = 0.03, df = 1 (P = 0.86), I² = 0%						

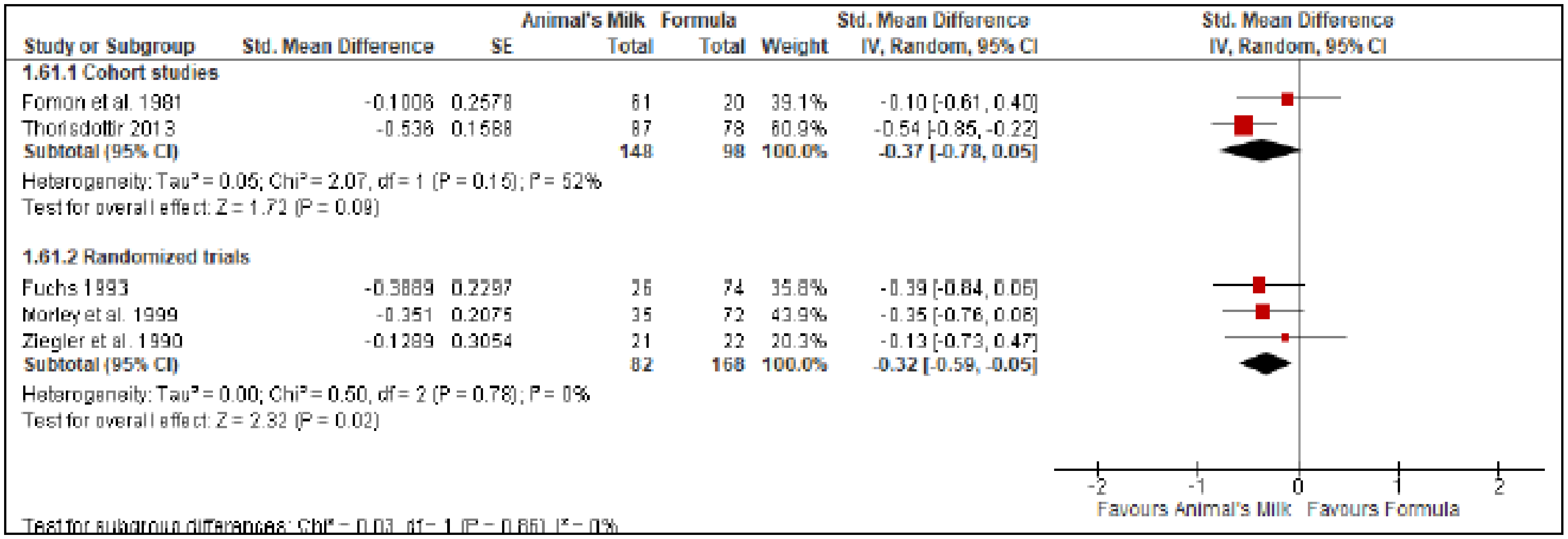

S1.13. Forest plot for effect of animal milk vs formula milk intake in infants 6-11 months of age on iron deficiency anemia

Study or Subgroup	Animal's Milk Events	Animal's Milk Total	Formula Events	Formula Total	Weight	Risk Ratio IV, Random, 95% CI
**1.63.1 Cohort studies**						
Thorisdottir 2013	3	87	0	78	5.2%	6.29 [0.33, 119.77]
Tunnessen et al. 1987	17	68	11	94	94.8%	2.14 [1.07, 4.26]
**Subtotal (95% CI)**		**155**		**172**	**100.0%**	**2.26 [1.15, 4.43]**
Total events	20		11			
Heterogeneity: Tau² = 0.00; Chi² = 0.49, df = 1 (P = 0.48); I² = 0%						
Test for overall effect: Z = 2.37 (P = 0.02)						
**Total (95% CI)**		**155**		**172**	**100.0%**	**2.26 [1.15, 4.43]**
Total events	20		11			
Heterogeneity: Tau² = 0.00; Chi² = 0.49, df = 1 (P = 0.48); I² = 0%						
Test for overall effect: Z = 2.37 (P = 0.02)						
Test for subgroup differences: Not applicable						

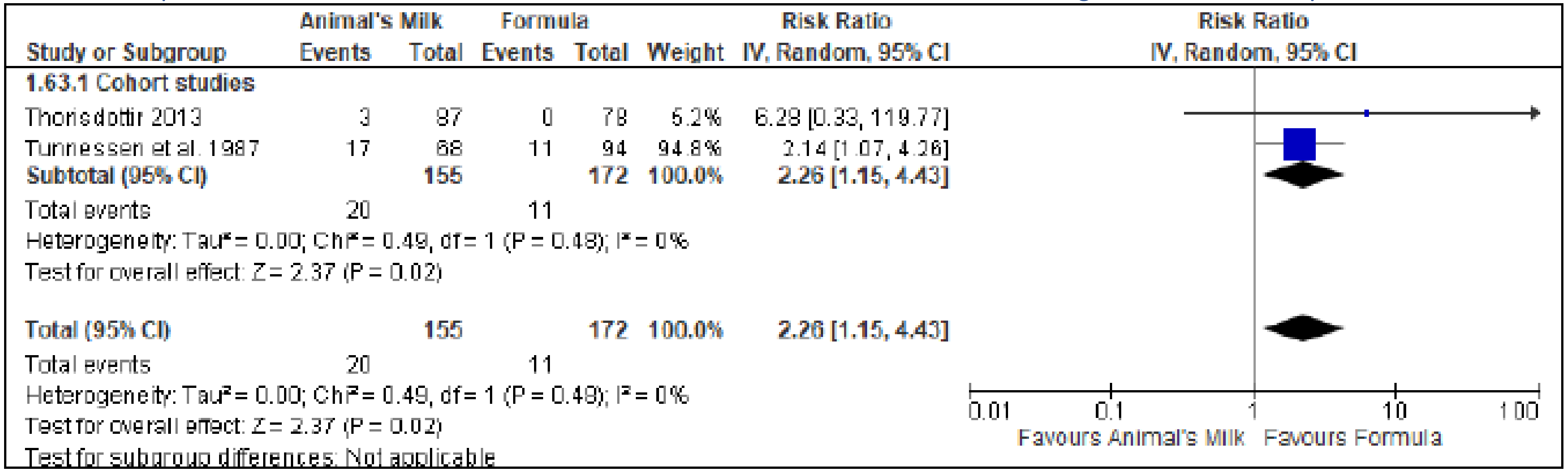

S1.14. Forest plot for effect of animal milk vs formula milk intake in infants 6-11 months of age on serum ferritin levels

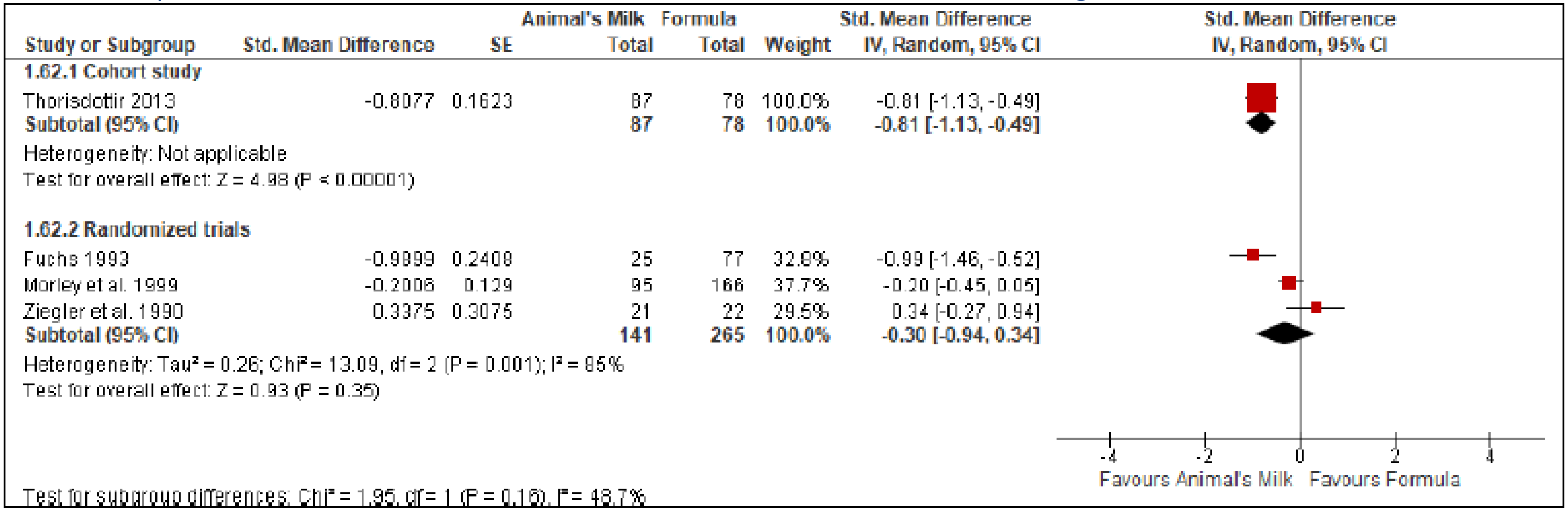

S1.15. Forest plot for effect of animal milk vs formula milk intake in infants 6-11 months of age on hemoglobin concentration in the stool

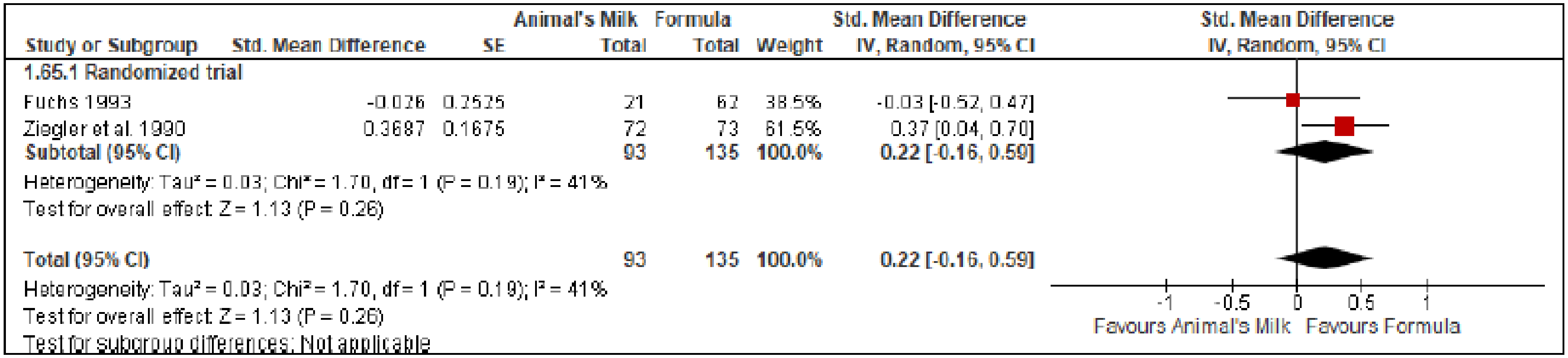

S1.16. GRADE Evidence Profiles for Certainty Assessment of primary outcomes and selected secondary outcomes

Certainty Assessment							№ of Patients		Effect		Certainty
№ of Studies	Study Design	Risk of Bias	Inconsistency	Indirectness	Imprecision	Other Considerations	Animal Milk	Infant Formula	Relative (95% CI)	Absolute (95% CI)	
Anemia at longest follow up-Randomized Controlled Trials											
2	randomised trials	serious [a]	not serious [b]	serious [c]	not serious [d]	none	12/60 (20.0%)	6/149 (4.0%)	RR 4.03 (1.68 to 9.65)	122 more per 1000 (from 27 more to 348 more)	⊕⊕○○ Low
Any anemia at the longest follow up-Cohort studies											
2	observational studies	serious [e]	not serious [f]	serious [c]	not serious	none	20/155 (12.9%)	11/172 (6.4%)	RR 2.26 (1.15 to 4.43)	81 more per 1000 (from 10 more to 219 more)	⊕⊕○○ Low
Gastrointestinal blood loss at longest follow up—Randomized Controlled Trials											
1	randomised trials	serious [g]	not serious	serious [c]	not serious [h]	none	9/21 (42.9%)	3/22 (13.6%)	RR 3.14 (0.98 to 10.04)	292 more per 1000 (from 3 fewer to 1000 more)	⊕⊕○○ Low
Gastrointestinal blood loss—Cohort study											
1	observational studies	serious [i]	not serious	serious [j]	serious [k]	none	26/60 (43.3%)	6/21 (28.6%)	RR 1.52 (0.73 to 3.16)	149 more per 1000 (from 77 fewer to 617 more)	⊕○○○ Very low
Weight-for-age at longest follow up-Randomized Controlled Trials											
3	randomised trials	serious [l]	not serious [m]	serious [n]	not serious [o]	none	194	362	-	SMD 0.02 SD lower (0.26 lower to 0.21 higher)	⊕⊕○○ Low
Height-for-age at the longest follow up-Randomized Controlled Trials											
2	randomised trials	serious [p]	not serious [q]	serious [n]	not serious [r]	none	185	344	-	SMD 0.07 SD higher (0.15 lower to 0.3 higher)	⊕⊕○○ Low
Serum hemoglobin concentration at the longest follow up—Randomized Controlled Trials											
3	randomised trials	serious [s]	not serious [b]	serious [c]	not serious	none	82	168	-	SMD 0.32 SD lower (0.59 lower to 0.05 lower)	⊕⊕○○ Low
Serum hemoglobin level—Cohort studies											
2	observational studies	serious [t]	not serious	serious [j]	not serious [u]	none	148	98	-	SMD 0.37 SD lower (0.78 lower to 0.05 higher)	⊕⊕○○ Low
Iron deficiency anemia at the longest follow up-Cohort studies											
2	observational studies	not serious	not serious [f]	serious [c]	not serious	strong association	20/155 (12.9%)	11/172 (6.4%)	RR 2.26 (1.15 to 4.43)	81 more per 1000(from 10 more to 219 more)	⊕⊕○○ Low
Constipation-Cohort study											
1	observational studies	not serious	not serious	serious [j]	serious [v]	strong association	7/69 (10.1%)	3/98 (3.1%)	RR 3.31 (0.89 to 12.37)	71 more per 1000 (from 3 fewer to 348 more)	⊕○○○ Very low

Certainty Assessment							№ of Patients		Effect		Certainty
№ of Studies	Study Design	Risk of Bias	Inconsistency	Indirectness	Imprecision	Other Considerations	Animal Milk	Infant Formula	Relative (95% CI)	Absolute (95% CI)	
Diarrhea-Cohort study											
1	observational studies	not serious	not serious	serious [j]	not serious	none	21/69 (30.4%)	16/98 (16.3%)	RR 1.86 (1.05 to 33.10)	140 more per 1000 (from 8 more to 1000 more)	⊕○○○ Very low
Neurodevelopment outcome (PDI scores) at the longest follow-Randomized Controlled Trial											
1	randomised trials	not serious	not serious	serious [j]	serious [w]	none	160	268	-	SMD 0.18 SD higher (0.02 lower to 0.37 higher)	⊕⊕○○ Low
Neurodevelopment outcome (MDI score) at the longest follow up-Randomized Controlled Trial											
1	randomised trials	not serious	not serious	serious [j]	serious [x]	none	160	268	-	SMD 0.16 SD higher (0.03 lower to 0.36 higher)	⊕⊕○○ Low

Footnotes: CI, confidence interval; RR, risk ratio; and SMD, standardized mean difference. Explanations: a One of the two randomized trial studies had "some concerns' for the risk of bias from the Cochrane risk of bias tool (2). b No statistical heterogeneity was found in the pooled data. $I^2 = 0\%$. There was clinical heterogeneity in the type of formula and animal milk use. We did

not downgrade the grade level for clinical heterogeneity as there is no consensus on the type of formula or animal milk that should be used when the breastmilk is not available and that multiple options are available for infant formula and animal milk in the community. [c] All the included studies were from high-income countries. This might limit the applicability of the results to populations from low and middle-income countries. We, however, think that the direction of effect might remain the same if there were eligible studies from low and middle-income countries and the magnitude of the effect might increase against animal milk. [d] Results were statistically significant and the confidence interval is fairly narrow around the summary estimate. [e] One cohort study had high risk of bias and the second one had some concerns for risk of bias. [f] The I2 statistics was 0%. [g] Study had "some concerns" for risk of bias based on Cochrane risk of bias tool (2). [h] Even though the confidence interval around the summary estimate included 1, the lower limit of the confidence interval was 0.98. [i] The study had "high risk of bias" from the ROBINS tool. [j] The only included study for this outcome was conducted in high-income country. [k] The confidence interval around the summary estimate included 1 and risk of increased or decreased risk cannot be excluded. [l] All three studies were randomized trials. One of the three randomized trial studies had 'high' and another has "some concerns" for the risk of bias from Cochrane risk of bias tool-2 (ROB 2). [m] The overall unexplained statistical heterogeneity based on 12 statistics was 19 %. The visual inspection of the forest plot showed that three of the included studies had an effect in the same direction and around the mean summary estimate. We did not downgrade the grade level for inconsistency for this outcome. [n] All but one of the included studies were from high-income countries. This might limit the applicability of the results to populations from low and middle-income countries. [o] The overall magnitude of the effect for the weight for age was small (SMD 0.06). This small statistical effect is not meaningful clinically. Moreover, even though the confidence interval included 0, the total sample size from the pooled studies was 1216. We think there was optimal information size (OIS) from the sample size of the pooled studies that if there was a true effect, that should have been picked up by this much of sample size. We, therefore, did not downgrade for imprecision. [p] Two studies were randomized trials. One of the two randomized trial studies had "some concerns" for the risk of bias from the Cochrane risk of bias tool-2(ROB 2). [q] Unexplained statistically heterogeneity based on 12 statistics was 17% only. [r] The overall magnitude of the effect for the weight for age was small (SMD 0.07) and the confidence interval included 0. This is a very small effect clinically. The total sample size in the analysis was 529 which should have been enough to pick a clinically meaningful effect. We, therefore, did not downgrade the level for imprecision. [s] The randomized trial studies had "some concerns" for the risk of bias from the Cochrane risk of bias tool-2. [t] One of the observational studies had 'high' risk of bias and the other had a 'moderate' risk of bias from the ROBINS-1 tool. [u] Even though the confidence interval around the summary estimate included a null effect, the upper limit was almost toward the threshold of statistical significance. The data from RCTs showed a similar direction of effect and was statistically significant. [v] The 95% CI around the summary estimate included 1. The total sample size was 167 which is not large enough to be confident about the summary estimate. [w] The overall magnitude of the effect was small (SMD 0.18) and the confidence interval included 0. [x] The overall magnitude of the effect was small (SMD 0.16) and the confidence interval included 0.
