# Supplementary material for: Dietary Strategies for Complementary Feeding between 6 and 24 Months of Age: The Evidence
Source: Nutrients. 2023 Jul 5;15(13):3041. doi: 10.3390/nu15133041 (PMC10346638; doi:10.3390/nu15133041)
Supplement: Supplementary file 1 [file nutrients-15-03041-s001.zip › Supplementary File S2.pdf]

SUPPLEMENTARY FILE S2 – The Consumption of varying frequencies, varieties, and quantities of fruits & vegetables, nuts, pulses, & seeds, and animal-sourced foods among children 6-23 months of age and their association with dietary and health outcomes: a systematic review (unpublished)

Authors: Leila Harrison, Komal Abdul Rahim, Christina Oh, Maria Maqsood, Kimberly D Charbonneau, Aatekah Owais, Emily C. Keats, Zohra Lassi, and Zulfiqar A. Bhutta

Table of Contents

S2.1. Objective **2**

S2.2. Methods **2**

S2.3. Medline Search Strategy **5**

S2.4. Prisma Diagram **8**

S2.5. List of publications included **9**

S2.6. Summary of Narrative Results for FV and NPS food groups **16**

S2.7. GRADE Assessments for FV and NPS Studies **46**

S2.8. Summary of Key Findings for ASF **78**

S2.9. Forest plots for ASF studies **89**

S2.10. GRADE Assessments for ASF Studies **92**

S2.1. Objective

The primary objectives of this systematic review are to compare: i) the effect of more frequent versus less frequent consumption of fruits, vegetables (FV), nuts, pulses, seeds (NPS), or animal-sourced foods (ASF) ii) the effect of more varied versus less varied consumption of FV, NPS, or ASF and iii) the effect of more amounts versus less amounts of FV, NPS, or ASF consumption among infants 6-23 months on dietary and health outcomes later in life. Secondary objectives of this review are to determine differences between processed/commercial versus fresh/home-prepared fruits, vegetables, nuts, pulses, seeds, or animal-sourced foods on dietary and health outcomes.

S2.2. Methods

S2.2.1. Criteria for considering studies for this review

Studies were eligible if they included healthy children aged 6-23 months, living in any low-, middle-, or high-income country. Eligible study designs included randomized controlled trials (RCTs; cluster or individually randomized), non-randomized controlled trials, cohort studies (prospective and retrospective), cross-sectional studies, and case-control studies. Qualitative studies and reviews were excluded from this review. Studies were also excluded if the infant population was considered unhealthy and infants were recruited based on this criterion. This includes, but is not limited to, infants with acute or chronic conditions/diseases, such as malnutrition, diarrhea or human immunodeficiency virus (HIV), and infants born preterm, small-for-gestational age (SGA) or low birthweight (LBW). Though our aim was to include healthy infants under two years of age, given the high burden of some of these conditions in certain populations globally, we recognize that malnourished infants and infants born preterm, SGA, or LBW are most likely included in the overall sample.

Studies were eligible for inclusion if they: i) measured and reported data on consumption practices (reported as frequency, variety, and/or amount of food consumed) during the complementary feeding period; ii) examined consumption of FV, NPS (e.g., legumes, beans, lentils, peas) or ASF; and iii) reported a relevant primary or secondary outcome. The type of milk provided during the complementary feeding period was not a consideration for inclusion (e.g., breast milk, animal milk, infant formula, or mixed).

We defined frequency, variety and amount exposures for FV, NPS, and ASF as follows:

- Frequency: none, once daily, 2-3 times per day, 3-4 times per day, >4 times per day, 1-3 times weekly, 4-6 times weekly, served either as meals or snacks.
- Variety: one type of food item per day, 2 different types per day, 3 different types per day or, >3 different types per day. These same variety exposures were evaluated on a per week basis as well.
- Amount, based on energy requirements by age:

- 6-8 months: <137 grams (g) per day, 137-187 g/day, >187 g/day, OR <200kcal/day, ≥200 kcal/day
- 9-11 months: <206 g/day, 206-281 g/day, >281 g/day, OR 9-11 months: <300 kcal/day, ≥300 kcal/day
- 12-23 months: <378 g/day, 378-515 g/day, >515 g/day, OR <550 kcal/day, ≥550 kcal/day

The comparison for frequency, variety, and amount exposures for FV, NPS, and ASF foods were as follows:

- Less frequent, varied or lower amounts of consumption of fruits and/or vegetables or nuts, pulses, and/or seeds, or animal-sourced foods

Primary outcomes included:

1. Subsequent consumption of food items across the two food groups at 1 year, 2 years, 3 years, 4 years, 5 years, and beyond 5 years of age.
2. Nutrient adequacy (e.g., protein intake and quality, micronutrient intake, choline and essential fatty acids) or nutrient excess (e.g., saturated fat, protein, sodium, fibre/phytate by type, fats), as reported by the study authors.
3. Nutrient status at study endline (blood concentration of vitamin A, vitamin C, vitamin D, vitamin E, B vitamins, zinc, iron, folate, selenium, lutein, carotenoids, iodine, fatty acids (omega 3 and 6); anemia; antioxidants.
4. Anthropometric outcomes, including stunting (height-for-age z-score < -2 SD), wasting (weight-for-age < -2 SD), overweight (weight-for-height > 2 SD), and obesity (weight-for-height > 3 SD), as defined by the WHO Growth Standards.
5. Anthropometric indices as continuous outcomes were also included and evaluated.
6. Child development (as defined by authors).
7. Contaminants within foods consumed (e.g., aflatoxins), as reported by the study authors.
8. Displacement of other foods/dietary adequacy, as reported by study authors.

Secondary outcomes included:

1. Food/taste preferences later in life.
2. Markers of lipid profiles (e.g., total cholesterol, triglyceride, low-density lipoprotein, high-density lipoprotein) at endline or latest follow up.
3. Markers of inflammation (e.g., C-reactive protein, plasminogen activator inhibitor-1) at study endline or latest follow-up.
4. Markers of gut health (e.g., Bifidobacterium, Clostridia, short chain fatty acids, environmental enteric dysfunction, microbiome) at study endline or latest follow-up.
5. Adverse effects, as reported by the study authors.
6. Morbidity (infectious).
7. Food-borne illness (not related to storage and handling).

8. Bone health, as defined by authors.
9. Oral health, as defined by authors.
10. Adverse events (e.g., choking).

S2.2.2. Search methods for identification of studies

Electronic searches were conducted in the following databases: MEDLINE, Embase, CINAHL, African Index Medicus (AIM), LILACS, The Cochrane Central Register of Controlled Trials (CENTRAL), eLENA (WHO). Searches were also conducted in non-indexed, grey literature using Google Scholar and select organizational websites including UNICEF, Nutrition International, the Global Alliance for Improved Nutrition, Helen Keller International, and the CDC. These organizations were chosen based on their relevant work in the areas of infant and child diet and nutrition. There were no restrictions on publication date or language. The date of the original search was October 7, 2020, and an updated search date of February 25, 2022. We searched the reference lists of all relevant systematic reviews captured during our electronic search for other studies that may not have been captured through this search strategy. The WHO's Guideline Development Group reviewed our list of included studies and provided suggestions for additional studies to screen.

Title, abstract, and full-text screening were managed using Covidence, a web-based software platform for systematic reviews. At both title/abstract and full-text screening stages, four review authors (CO, KC, KR, LH) independently scanned and screened all records retrieved by the searches for relevance based on selection criteria. The update search results were screened in duplicate by three review authors (CO, MM, LH). Any disagreements were resolved through discussion or by a third review author when necessary.

S2.2.3. Data collection and analysis

Four review authors (CO, KC, KR, LH) independently extracted data from each included study onto a standardized data extraction form in Excel that had been piloted. The review update was independently extracted by three authors (CO, MM, LH). All studies were matched between the review authors, and any disagreements or discrepancies were resolved through discussion, or by a third independent reviewer. The following information were extracted from each included study: source (e.g., contact details); study characteristics (e.g., study design, location of study, years of data collection, etc.); population characteristics (e.g., age, sample size, nutritional information at baseline, breastfeeding status, etc.); intervention/comparison characteristics (e.g., exposure, tools used for measurement, etc.); outcomes (e.g., food frequency data, methods/tools used, age at outcome assessment, etc.); data analysis methods; control of confounding; funding obtained, and any conflict of interests.

Non-English language studies that were included in this review were translated from Portuguese (n=1) and Chinese (n=2) to English. Where any information was unclear, seemed incorrect, or missing, we contacted the authors for missing details (n=5).

Quality assessments of included studies were conducted independently by five review authors (CO, KC, KR, MM LH). Any discrepancies between reviewers were resolved through discussion or by a third reviewer. Individual studies were critically appraised using the Cochrane Risk of Bias-2 (ROB-2) tool for randomized controlled trials and cluster-randomized controlled trials, the NIH tool for observational cohort and cross-sectional studies, and the ROBINS-I Tool for non-randomized studies. Using the NIH tool, observational studies were rated good, fair, or poor, based on fourteen criteria covering the research question, participant population, analyses, timeframe, independent and dependent variables, attrition, and control of confounding variables. The Cochrane Risk of Bias-2 tool assesses RCT studies for risk of bias in the following domains: randomization process, deviations from the intended interventions, missing outcome data, outcome measurement, and the selection of the reported results. An overall risk of bias judgement was given to each study (low, high, some concerns). The ROBINS-I tool assessed non-randomized trials in the following domains: bias due to confounding, bias in selection of study participants, bias in classification of interventions, bias due to deviations from intended interventions, bias due to missing data, bias in measurement of outcomes, and bias in selection of the reported results. Each study was given an overall risk of bias judgement (low, moderate, serious, critical). The GRADE tool was used to assess the certainty of evidence for all reported outcomes for FV and NPS food groups. For ASF, GRADE assessments were conducted only for 6 critical outcomes: weight-for-age Z-score (WAZ), height-for-age Z-score (HAZ), and weight-for-height Z-score (WHZ). The certainty of evidence was rated (very low, low, moderate, high) for each outcome in accordance with the GRADE framework. Evidence was downgraded based on five factors: risk of bias, inconsistency, indirectness, imprecision, and publication bias. The criteria and reasons for downgrading were provided in explanatory footnotes in the GRADE tables below.

All experimental and observational study data were analyzed separately. Conducting meta-analysis was possible for only ASF consumption on three outcomes, given the heterogeneity across studies, including food group items, outcome metrics, and frequency categories. The meta-analysis was done using Review Manager 5.4 with applied random effects. We planned the sub-group analysis based on the age (6-8 months, 9-11 months, and 12-23 months), the type of food consumed, and the participants' socio-economic status. However, we could not perform any sub-group analysis given the sparse and heterogeneous data that did not allow for meta-analysis. As such, a narrative synthesis was conducted for the FV and NPS food groups, and majority of outcomes for ASF.

S2.3. Medline Search Strategy

**Search Number**	**Search Terms**
**Search Set 1**	**Child Terms**

1	exp Infant, Newborn/
2	exp Infant/
3	child/ or child, preschool/
4	(infan* or babies or baby or newborn? or neonat* or toddler? or child*). tw,kf.
**5**	**or/1-4**
**Search Set 2**	**Complementary Feeding Terms**
6	Weaning/
7	Infant Nutritional Physiological Phenomena/
8	Feeding Behavior/
9	Eating/
10	complementary feed*. tw,kf.
11	(feed* or food? or diet* or eat* or wean* or consume? or consuming or consumption). tw,kf.
**12**	**or/6-11**
**Search Set 3**	**Food Terms**
13	Fruit/
14	Vegetables/
15	meat/ or meat products/ or exp meat proteins/ or exp poultry/ or exp red meat/ or exp seafood/
16	dairy products/ or exp cultured milk products/ or exp milk/
17	nuts/ or seeds/
18	Fabaceae/ or Phaseolus/
**19**	**or/13-18**
20	(frequen* or quantit* or regular* or amount* or variet* or divers* or type*).tw,kf.
**21**	**19 and 20**
22	((leafy or vitamin-A rich or vitamin A rich or nutrient dense or nutrient-dense or nutrient rich or nutrient-rich) adj5 (frequen* or quantit* or amount* or variet* or divers* or type*)).tw,kf.

23	((fruit? or vegetable? or greens or broccoli or apricot* or papaya* or avocado* or cabbage* or squash* or banana* or orange* or carrot* or mango*) adj5 (frequen* or quantit* or amount* or variet* or divers* or type*)).tw,kf.
24	((meat* or beef or pork or lamb or goat* or game or poultry or chicken* or camel? or venison or egg* or insect* or caterpillar* or spider* or beetle* or termite* or ant or ants or animal-source? or flesh-food? or flesh food?) adj5 (frequen* or quantit* or amount* or variet* or divers* or type*)).tw,kf.
25	((seafood or sea-food or shellfish* or fish* or marine*) adj5 (frequen* or quantit* or amount* or variet* or divers* or type*)).tw,kf.
26	((dairy or milk or cheese or yogurt or butter or kefir or kephir or bulgaros) adj5 (frequen* or quantit* or amount* or variet* or divers* or type*)).tw,kf.
27	((legume? or pulse? or nut? or seed? or bean? or lentil? or chickpea? or pea? or cowpea? or soybean* or chestnut*) adj5 (frequen* or quantit* or amount* or variet* or divers* or type*)).tw,kf.
**28**	**or/21-27**
**29**	**5 and 12 and 28**

S2.4. Prisma Diagram

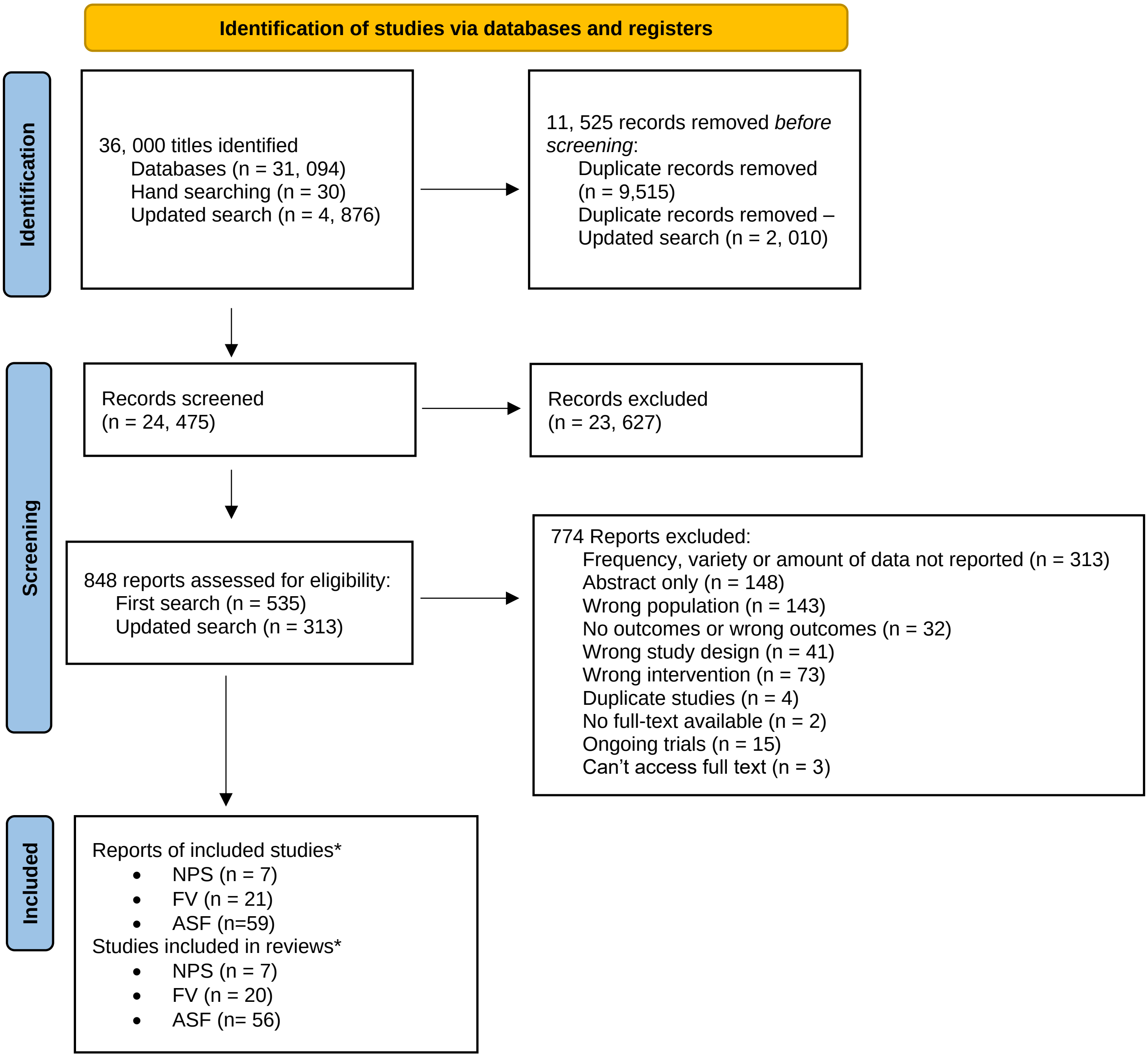

*Some studies reported data for more than one food group, and thus, were included in multiple reviews. The same is true for the number of reports of included studies.

S2.5. List of publications included

List of Included Studies for Fruits and Vegetables (n=20 studies)

#	Citation
1	A. Ahmad, S. Madanijah, C. M. Dwiriani, and R. Kolopaking, "Complementary feeding practices and nutritional status of children 6-23 months old: formative study in Aceh, Indonesia," (in eng), *Nutr Res Pract,* vol. 12, no. 6, pp. 512-520, Dec 2018, doi: 10.4162/nrp.2018.12.6.512.
2	M. Bjelland, A. L. Brantsæter, M. Haugen, H. M. Meltzer, W. Nystad, and L. F. Andersen, "Changes and tracking of fruit, vegetables and sugar-sweetened beverages intake from 18 months to 7 years in the Norwegian Mother and Child Cohort Study," (in eng), *BMC Public Health,* vol. 13, p. 793, Aug 30 2013, doi: 10.1186/1471-2458-13-793.
3	K. V. Braun *et al.*, "Dietary Intake of Protein in Early Childhood Is Associated with Growth Trajectories between 1 and 9 Years of Age," (in eng), *J Nutr,* vol. 146, no. 11, pp. 2361-2367, Nov 2016, doi: 10.3945/jn.116.237164.
4	S. Chang, C. Chen, W. He, and Y. Wang, "[Analysis on the changes of nutritional status in China--the improvement of complementary feeding among Chinese infants and young children]," (in chi), *Wei Sheng Yan Jiu,* vol. 36, no. 2, pp. 207-9, Mar 2007.
5	I. Cowin, A. Emond, P. Emmett, and A. S. Group, "Association between composition of the diet and haemoglobin and ferritin levels in 18-month-old children," (in eng), *Eur J Clin Nutr,* vol. 55, no. 4, pp. 278-86, Apr 2001, doi: 10.1038/sj.ejcn.1601155.
6	C. Darapheak, T. Takano, M. Kizuki, K. Nakamura, and K. Seino, "Consumption of animal source foods and dietary diversity reduce stunting in children in Cambodia," (in eng), *Int Arch Med,* vol. 6, p. 29, 2013, doi: 10.1186/1755-7682-6-29.
7	F. L. Garden, G. B. Marks, C. Almqvist, J. M. Simpson, and K. L. Webb, "Infant and early childhood dietary predictors of overweight at age 8 years in the CAPS population," (in eng), *Eur J Clin Nutr,* vol. 65, no. 4, pp. 454-62, Apr 2011, doi: 10.1038/ejcn.2011.7.
8	M. M. Hetherington *et al.*, "A step-by-step introduction to vegetables at the beginning of complementary feeding. The effects of early and repeated exposure," (in eng), *Appetite,* vol. 84, pp. 280-90, Jan 2015, doi: 10.1016/j.appet.2014.10.014.
9	K. Kittisakmontri *et al.*, "Comparison of 24-Hour Recall and 3-Day Food Records during the Complementary Feeding Period in Thai Infants and Evaluation of Plasma Amino Acids as Markers of Protein Intake," (in eng), *Nutrients,* vol. 13, no. 2, Feb 17 2021, doi: 10.3390/nu13020653.
10	E. Lundkvist, E. Stoltz Sjöström, R. Lundberg, S. A. Silfverdal, C. E. West, and M. Domellöf, "Fruit Pouch Consumption and Dietary Patterns Related to BMIz at 18 Months of Age," (in eng), *Nutrients,* vol. 13, no. 7, Jun 30 2021, doi: 10.3390/nu13072265.
11	a. A.S. Maier, C. Chabanet, B. Schaal, P. D. Leathwood, and S. N. Issanchou, "Breastfeeding and experience with variety early in weaning increase infants' acceptance of new foods for up to two months," (in eng), *Clin Nutr,* vol. 27, no. 6, pp. 849-57, Dec 2008, doi: 10.1016/j.clnu.2008.08.002.

	b. A. Maier-Nöth, B. Schaal, P. Leathwood, and S. Issanchou, "The Lasting Influences of Early Food-Related Variety Experience: A Longitudinal Study of Vegetable Acceptance from 5 Months to 6 Years in Two Populations," (in eng), *PLoS One,* vol. 11, no. 3, p. e0151356, 2016, doi: 10.1371/journal.pone.0151356.
12	K. M. Mallan, A. Fildes, A. M. Magarey, and L. A. Daniels, "The Relationship between Number of Fruits, Vegetables, and Noncore Foods Tried at Age 14 Months and Food Preferences, Dietary Intake Patterns, Fussy Eating Behavior, and Weight Status at Age 3.7 Years," (in eng), *J Acad Nutr Diet,* vol. 116, no. 4, pp. 630-7, Apr 2016, doi: 10.1016/j.jand.2015.06.006.
13	M. S. Morseth *et al.*, "Tracking of infant and young child feeding practices among 9- to 24-month-old children in Nepal: the MAL-ED Birth Cohort Study," (in eng), *Public Health Nutr,* vol. 21, no. 2, pp. 355-364, 02 2018, doi: 10.1017/S1368980017002294.
14	M. M. Murphy, L. M. Barraj, T. D. Brisbois, and A. M. Duncan, "Frequency of fruit juice consumption and association with nutrient intakes among Canadians," (in eng), *Nutr Health,* vol. 26, no. 4, pp. 277-283, Dec 2020, doi: 10.1177/0260106020944299.
15	B. Ntab, K. Simondon, and J. Milet, "A Young Child Feeding Index Is Not Associated with Either Height-for-Age or Height Velocity in Rural Senegalese Children," vol. 135, ed. *The Journal of Nutrition*, 2004, pp. 457-464.
16	A. M. Assis, E. N. Gaudenzi, G. Gomes, R. e. C. Ribeiro, S. C. Szarfarc, and S. B. Souza, "[Hemoglobin concentration, breastfeeding and complementary feeding in the first year of life]," (in por), *Rev Saude Publica,* vol. 38, no. 4, pp. 543-51, Aug 2004, doi: 10.1590/s0034-89102004000400010.
17	D. G. Silva, S. E. Priore, and S. o. C. Franceschini, "Risk factors for anemia in infants assisted by public health services: the importance of feeding practices and iron supplementation," (in eng), *J Pediatr (Rio J),* vol. 83, no. 2, pp. 149-56, 2007 Mar-Apr 2007, doi: 10.2223/JPED.1603.
18	A. L. Thorne-Lyman *et al.*, "Dietary Diversity and Child Development in the Far West of Nepal: A Cohort Study," (in eng), *Nutrients,* vol. 11, no. 8, Aug 03 2019, doi: 10.3390/nu11081799.
19	P. T. Olsen *et al.*, "[Iron status and weaning practices among healthy 1-year old infants]," (in nor), *Tidsskr Nor Laegeforen,* vol. 115, no. 5, pp. 612-4, Feb 20 1995.
20	N. R. Wang *et al.*, "[Prevalence and risk factors of overweight and obesity among infants in Chongqing urban area]," (in chi), *Zhongguo Dang Dai Er Ke Za Zhi,* vol. 15, no. 3, pp. 207-11, Mar 2013.

List of Included Studies for Nuts, Pulses, and Seeds (n=7 studies)

#	Citation
1	A. Ahmad, S. Madanijah, C. M. Dwiriani, and R. Kolopaking, "Complementary feeding practices and nutritional status of children 6-23 months old: formative study in Aceh, Indonesia," (in eng), *Nutr Res Pract,* vol. 12, no. 6, pp. 512-520, Dec 2018, doi: 10.4162/nrp.2018.12.6.512.

2	C. Darapheak, T. Takano, M. Kizuki, K. Nakamura, and K. Seino, "Consumption of animal source foods and dietary diversity reduce stunting in children in Cambodia," (in eng), *Int Arch Med,* vol. 6, p. 29, 2013, doi: 10.1186/1755-7682-6-29.
3	I. de Jager, K. J. Borgonjen-van den Berg, K. E. Giller, and I. D. Brouwer, "Current and potential role of grain legumes on protein and micronutrient adequacy of the diet of rural Ghanaian infants and young children: using linear programming," (in eng), *Nutr J,* vol. 18, no. 1, p. 12, 02 21 2019, doi: 10.1186/s12937-019-0435-5
4	C. Negash, T. Belachew, C. J. Henry, A. Kebebu, K. Abegaz, and S. J. Whiting, "Nutrition education and introduction of broad bean-based complementary food improves knowledge and dietary practices of caregivers and nutritional status of their young children in Hula, Ethiopia," (in eng), *Food Nutr Bull*, vol. 35, no. 4, pp. 480-6, Dec 2014, doi: 10.1177/156482651403500409.
5	V. A. Obatolu, "Growth pattern of infants fed with a mixture of extruded malted maize and cowpea," (in eng), *Nutrition*, vol. 19, no. 2, pp. 174-8, Feb 2003, doi: 10.1016/s0899-9007(02)01102-4.
6	A. M. Assis, E. N. Gaudenzi, G. Gomes, R. e. C. Ribeiro, S. C. Szarfarc, and S. B. Souza, "[Hemoglobin concentration, breastfeeding and complementary feeding in the first year of life]," (in por), *Rev Saude Publica,* vol. 38, no. 4, pp. 543-51, Aug 2004, doi: 10.1590/s0034-89102004000400010.
7	D. G. Silva, S. E. Priore, and S. o. C. Franceschini, "Risk factors for anemia in infants assisted by public health services: the importance of feeding practices and iron supplementation," (in eng), *J Pediatr (Rio J),* vol. 83, no. 2, pp. 149-56, 2007 Mar-Apr 2007, doi: 10.2223/JPED.1603.

List of Included Studies for ASF (n=56 studies)

#	Citation
1	Iannotti LL, Lutter CK, Stewart CP, Gallegos Riofrío CA, Malo C, Reinhart G, Palacios A, Karp C, Chapnick M, Cox K, Waters WF. Eggs in Early Complementary Feeding and Child Growth: A Randomized Controlled Trial. *Pediatrics.* 2017 Jul;140(1):e20163459. doi: 10.1542/peds.2016-3459.
2	Stewart CP, Caswell B, Iannotti L, Lutter C, Arnold CD, Chipatala R, Prado EL, Maleta K. The effect of eggs on early child growth in rural Malawi: the Mazira Project randomized controlled trial. *Am J Clin Nutr*. 2019 Oct 1;110(4):1026-1033. doi: 10.1093/ajcn/nqz163.
3	Ahmad A, Madanijah S, Dwiriani CM, Kolopaking R. Complementary feeding practices and nutritional status of children 6-23 months old: formative study in Aceh, Indonesia. *Nutr*. 2018;12(6):512-20.
4	Budree S, Goddard E, Brittain K, Cader S, Myer L, Zar HJ. Infant feeding practices in a South African birth cohort-A longitudinal study. *Maternal and Child Nutrition*. 2017;13(3).
5	Di Marcantonio F, Custodio E, Abukar Y. Child Dietary Diversity and Associated Factors Among Children in Somalian IDP Camps. *Food Nutr Bull*. 2020;41(1):61-76.

6	Marinda PA, Genschick S, Khayeka-Wandabwa C, Kiwanuka-Lubinda R, Thilsted SH. Dietary diversity determinants and contribution of fish to maternal and under-five nutritional status in Zambia. *PLoS ONE*. 2018;13(9):e0204009.
7	Nakamura M, Hamazaki K, Matsumura K, Kasamatsu H, Tsuchida A, Inadera H, et al. Infant dietary intake of yogurt and cheese and gastroenteritis at 1 year of age: The Japan Environment and Children's Study. *PLoS ONE*. 2019;14(10):e0223495.
8	Ntab B, Simondon KB, Milet J, Cisse B, Sokhna C, Boulanger D, et al. A young child feeding index is not associated with either height-for-age or height velocity in rural Senegalese children. *The Journal of nutrition*. 2005;135(3):457-64.
9	Kiefte-de Jong JC, de Vries JH, Franco OH, Jaddoe VW, Hofman A, Raat H, et al. Fish consumption in infancy and asthma-like symptoms at preschool age. *Pediatrics*. 2012;130(6):1060-8
10	Magnusson J, Kull I, Rosenlund H, Hakansson N, Wolk A, Melen E, et al. Fish consumption in infancy and development of allergic disease up to age 12 y. *Am J Clin Nutr*. 2013;97(6):1324-30.
11	Krebs NF, Mazariegos M, Chomba E, Sami N, Pasha O, Tshefu A, et al. Randomized controlled trial of meat compared with multimicronutrient-fortified cereal in infants and toddlers with high stunting rates in diverse settings. *Am J Clin Nutr*. 2012;96(4):840-7.
12	Libuda L, Mesch CM, Stimming M, Demmelmair H, Koletzko B, Warschburger P, et al. Fatty acid supply with complementary foods and LC-PUFA status in healthy infants: results of a randomised controlled trial. *Eur J Nutr*. 2016;55(4):1633-44.
13	Silva DG, Priore SE, Franceschini Sdo C. Risk factors for anemia in infants assisted by public health services: the importance of feeding practices and iron supplementation. *Jornal de pediatria*. 2007;83(2):149-56.
14	Zhao A, Gao H, Li B, Zhang J, Win NN, Wang P, et al. Inappropriate feeding behavior: One of the important causes of malnutrition in 6- to 36-month-old children in Myanmar. *American Journal of Tropical Medicine and Hygiene*. 2016;95(3):702-8.
15	Urkin J, Adam D, Weitzman D, Gazala E, Chamni S, Kapelushnik J. Indices of iron deficiency and anaemia in Bedouin and Jewish toddlers in southern Israel. *Acta Paediatr*. 2007;96(6):857-60
16	Shoda T, Futamura M, Yang L, Narita M, Saito H, Ohya Y. Yogurt consumption in infancy is inversely associated with atopic dermatitis and food sensitization at 5 years of age: A hospital-based birth cohort study. *J Dermatol Sci*. 2017;86(2):90-6
17	de Pee S, Bloem MW, Satoto, Yip R, Sukaton A, Tjiong R, Shrimpton R, Muhilal, Kodyat B. Impact of a social marketing campaign promoting dark-green leafy vegetables and eggs in central Java, Indonesia. Int J Vitam Nutr Res. 1998;68(6):389-98. PMID: 9857267.
18	Chang S, Chen C, He W, Wang Y. [Analysis on the changes of nutritional status in China--the improvement of complementary feeding among Chinese infants and young children]. *Wei Sheng Yan Jiu*. 2007 Mar;36(2):207-9. Chinese.
19	Thorne-Lyman AL, Shrestha M, Fawzi WW, Pasqualino M, Strand TA, Kvestad I, et al. Dietary Diversity and Child Development in the Far West of Nepal: A Cohort Study. *Nutrients*. 2019;11(8)

20	Dumas SE, Kassa L, Young SL, Travis AJ. Examining the association between livestock ownership typologies and child nutrition in the Luangwa Valley, Zambia. *PLOS ONE*. 2018;13(2):e0191339
21	Darapheak C, Takano T, Kizuki M, Nakamura K, Seino K. Consumption of animal source foods and dietary diversity reduce stunting in children in Cambodia. *International Archives of Medicine*. 2013;6.
22	Nicklaus S, Divaret-Chauveau A, Chardon ML, Roduit C, Kaulek V, Ksiazek E, et al. The protective effect of cheese consumption at 18 months on allergic diseases in the first 6 years. *Allergy*. 2019;74(4):788-98.
23	Muslimatun S, Wiradnyani LA. Dietary diversity, animal source food consumption and linear growth among children aged 1-5 years in Bandung, Indonesia: a longitudinal observational study. *The British journal of nutrition*. 2016;116 Suppl 1:S27-35.
24	Wang NR, Huang J, Li KP, Zhao Y, Wen J, Ye Y, Fan X. [Prevalence and risk factors of overweight and obesity among infants in Chongqing urban area]. Zhongguo Dang Dai Er Ke Za Zhi. 2013 Mar;15(3):207-11. *Chinese*.
25	Braun KV, Erler NS, Kiefte-de Jong JC, Jaddoe VW, van den Hooven EH, Franco OH, et al. Dietary Intake of Protein in Early Childhood Is Associated with Growth Trajectories between 1 and 9 Years of Age. *The Journal of nutrition*. 2016;146(11):2361-7
26	Chen D, McKune SL, Singh N, Yousuf Hassen J, Gebreyes W, Manary MJ, et al. Campylobacter Colonization, Environmental Enteric Dysfunction, Stunting, and Associated Risk Factors Among Young Children in Rural Ethiopia: A Cross-Sectional Study From the Campylobacter Genomics and Environmental Enteric Dysfunction (CAGED) Project. *Frontiers in public health*. 2020;8:615793.
27	Sunardi D, Bardosono S, Basrowi RW, Wasito E, Vandenplas Y. Dietary Determinants of Anemia in Children Aged 6-36 Months: A Cross-Sectional Study in Indonesia. *Nutrients*. 2021;13(7).
28	Dube K, Schwartz J, Mueller MJ, Kalhoff H, Kersting M. Complementary food with low (8%) or high (12%) meat content as source of dietary iron: a double-blinded randomized controlled trial. *European Journal of Nutrition.* 2010;49(1):11-8.
29	Lartey A, Manu A, Brown KH, Peerson JM, Dewey KG. A randomized, community-based trial of the effects of improved, centrally processed complementary foods on growth and micronutrient status of Ghanaian infants from 6 to 12 mo of age. Am J Clin Nutr. 1999 Sep;70(3):391-404. doi: 10.1093/ajcn/70.3.391. PMID: 10479202.
30	Hoffman Dr TRCCYSWDHBRGOCARMSEWLE. Maturation of visual acuity is accelerated in breast-fed term infants fed baby food containing DHA-enriched egg yolk. *The Journal of nutrition*. 2004;134(9):2307–13.
31	Tang M, Krebs NF. High protein intake from meat as complementary food increases growth but not adiposity in breastfed infants: a randomized trial. *Am J Clin Nutr*. 2014;100(5):1322-8.
32	Makrides M HJSNMAGRA. Nutritional effect of including egg yolk in the weaning diet of breast-fed and formula-fed infants: a randomized controlled trial. *Am J Clin Nutr*. 2002;75(6):1084– 92.

33	Skau JK, Touch B, Chhoun C, Chea M, Unni US, Makurat J, et al. Effects of animal source food and micronutrient fortification in complementary food products on body composition, iron status, and linear growth: a randomized trial in Cambodia. *Am J Clin Nutr*. 2015;101(4):742-51.
34	Engelmann MDM, Sandström B, Michaelsen KF. Meat Intake and Iron Status in Late Infancy: An Intervention Study. *Journal of Pediatric Gastroenterology and Nutrition*. 1998;26(1):26-33
35	Garden FL, Marks GB, Almqvist C, Simpson JM, Webb KL. Infant and early childhood dietary predictors of overweight at age 8 years in the CAPS population. *European journal of clinical nutrition.* 2011;65(4):454-62
36	Bauserman M, Lokangaka A, Gado J, Close K, Wallace D, Kodondi KK, et al. A cluster-randomized trial determining the efficacy of caterpillar cereal as a locally available and sustainable complementary food to prevent stunting and anaemia. *Public Health Nutr*. 2015;18(10):1785-92
37	Tang M, Sheng XY, Krebs NF, Hambidge KM. Meat as complementary food for older breastfed infants and toddlers: a randomized, controlled trial in rural China. *Food Nutr* Bull. 2014;35(4 Suppl):S188-92.
38	Hopkins D, Emmett P, Steer C, Rogers I, Noble S, Emond A. Infant feeding in the second 6 months of life related to iron status: an observational study. *Arch Dis Child*. 2007;92(10):850-4.
39	Thorsdottir I, Gunnarsson BS, Atladottir H, Michaelsen KF, Palsson G. Iron status at 12 months of age -- effects of body size, growth and diet in a population with high birth weight. *European journal of clinical nutrition*. 2003;57(4):505-13.
40	Nielsen G, Thomsen B, Michaelsen K. Influence of breastfeeding and complementary food on growth between 5 and 10 months. *ACTA PÆDIATRIC*. 1998;87: 911–7.
41	Oliveira MA, Osorio MM, Raposo MC. Socioeconomic and dietary risk factors for anemia in children aged 6 to 59 months. *Jornal de pediatria.* 2007;83(1):39-46.
42	Mak TN, Angeles-Agdeppa I, Tassy M, Capanzana MV, Offord EA. Contribution of Milk Beverages to Nutrient Adequacy of Young Children and Preschool Children in the Philippines. *Nutrients*. 2020;12(2)
43	Webb K, Rutishauser I, Katz T, Knezevic N, Marjaana Lahti-Koski, Peat J, et al. Meat consumption among 18-month-old children participating in the Childhood Asthma Prevention Study. *Nutrition & Dietetics*. 2005;62:12–20.
44	Taylor A, Redworth EW, Morgan JB. Influence of diet on iron, copper, and zinc status in children under 24 months of age. *Biol Trace Elem Res*. 2004;97(3):197-214.
45	Krasevec J, An X, Kumapley R, Begin F, Frongillo EA. Diet quality and risk of stunting among infants and young children in low- and middle-income countries. *Maternal & child nutrition*. 2017;13(2):10.
46	Long JK, Murphy SP, Weiss RE, Nyerere S, Bwibo NO, Neumann CG. Meat and milk intakes and toddler growth: a comparison feeding intervention of animal-source foods in rural Kenya. *Public Health Nutr*. 2012;15(6):1100-7.

47	Kaimila Y, Divala O, Agapova SE, Stephenson KB, Thakwalakwa C, Trehan I, et al. Consumption of Animal-Source Protein is Associated with Improved Height-for-Age z Scores in Rural Malawian Children Aged 12(-)36 Months. *Nutrients*. 2019;11(2).
48	Kittisakmontri K, Lanigan J, Sangcakul A, Tim-Aroon T, Meemaew P, Wangaueattachon K, et al. Comparison of 24-Hour Recall and 3-Day Food Records during the Complementary Feeding Period in Thai Infants and Evaluation of Plasma Amino Acids as Markers of Protein Intake. *Nutrients*. 2021;13(2)
49	Olaya GA, Lawson M, Fewtrell MS. Efficacy and safety of new complementary feeding guidelines with an emphasis on red meat consumption: a randomized trial in Bogota, Colombia. *Am J Clin Nutr*. 2013;98(4):983-93
50	Makrides M, Leeson R, Gibson RA, Simmer K. A randomized controlled clinical trial of increased dietary iron in breast-fed infants. *The Journal of Pediatrics*. 1998;133(4):559-62
51	Krebs NF, Westcott JE, Butler N, Robinson C, Bell M, Hambidge KM. Meat as a First Complementary Food for Breastfed Infants: Feasibility and Impact on Zinc Intake and Statusv. *Journal of Pediatric Gastroenterology and Nutrition*. 2006;42:207–14
52	Marquis GS, Colecraft EK, Kanlisi R, Aidam BA, Atuobi-Yeboah A, Pinto C, et al. An agriculture-nutrition intervention improved children's diet and growth in a randomized trial in Ghana. *Maternal & child nutrition*. 2018;14 Suppl 3:e12677.
53	Szymlek-Gay EA, Ferguson EL, Heath AL, Gray AR, Gibson RS. Food-based strategies improve iron status in toddlers: a randomized controlled trial12. *Am J Clin Nutr*. 2009;90(6):1541-51
54	Bierut T, Duckworth L, Grabowsky M, Ordiz MI, Laury ML, Callaghan-Gillespie M, et al. The effect of bovine colostrum/egg supplementation compared with corn/soy flour in young Malawian children: a randomized, controlled clinical trial. *Am J Clin Nutr.* 2021;113(2):420-7.
55	Krebs NF, Mazariegos M, Tshefu A, Bose C, Sami N, Chomba E, et al. Meat consumption is associated with less stunting among toddlers in four diverse low-income settings. *Food Nutr Bull.* 2011;32(3):185-91
56	P. T. Olsen *et al.*, "[Iron status and weaning practices among healthy 1-year old infants]," (in nor), *Tidsskr Nor Laegeforen,* vol. 115, no. 5, pp. 612-4, Feb 20 1995.

S2.6. Summary of Narrative Results for FV and NPS food groups

Table 1. Narrative Synthesis Table for Anthropometric Outcomes

Outcome	Study Design	Participants	Country	Narrative Synthesis	**Other notes** (i.e., breastfeeding status, confounders)
**Vegetables**					
Wasting	CS	392 participants aged 6-23 months	Indonesia	Frequency of eating green leafy and orange vegetables was not significantly associated with wasting prevalence (p=0.542).	
Wasting	CS	1,907 participants aged 12-59 months	Cambodia	Frequency of eating green leafy or orange color vegetables was not significantly associated with wasting prevalence (p>0.05).	Adjusted by household wealth quintile, education of mother, geographical area, and residential location.
Underweight	CS	392 participants aged 6-23 months	Indonesia	Frequency of eating green leafy and orange vegetables was not significantly associated with underweight prevalence (p=0.969).	
Underweight	CS	1,907 participants aged 12-59 months	Cambodia	Frequency of eating green leafy or orange color vegetables was not significantly associated with underweight prevalence p>0.05).	Adjusted by household wealth quintile, education of mother, geographical area, and residential location.
Stunting	CS	392 participants aged 6-23 months	Indonesia	Frequency of eating green leafy and orange vegetables was not significantly associated with stunting prevalence (p=0.491).	
Stunting	CS	1,907 participants aged 12-59 months	Cambodia	Frequency of eating green leafy or orange color vegetables was not significantly associated with stunting prevalence (p>0.05).	Adjusted by household wealth quintile, education of mother, geographical area, and residential location.

**Overweight**	Cohort	1,916 participants aged 6-18 months	China	Frequency of eating vegetables was significantly associated with overweight prevalence after 12 months of follow-up. 2.99% (n=7) and 17.84% (n=300) of those who consumed vegetables <7 times/week (n=234) and ≥ 7 times/week (n=1,682) were overweight, respectively (p<0.001 between groups).	
BMI	Cohort	362 participants aged 18 months to 8 years	Australia	Higher intake of vegetables in grams at 18 months was not significantly associated with BMI at 8 years old (p=0.05).	Model adjusted by sex, asthma study intervention group, birth weight, breastfeeding for at least 6 months, parental obesity status, ethnicity, smoking in pregnancy, father's education, and for total energy intake.<br><br>Breastfed ≥ 6 months:<br>No: 211 (58.3%)<br>Yes: 151 (41.7%)
BMI	Cohort	3,564 participants aged 1-9 years	Netherlands	Higher intake of vegetable protein in early childhood (1 year old) was not significantly associated with BMI measures in later childhood (between the ages of 1 and 9 years old) (p>0.05).	Model adjusted for child sex, ethnicity, age at dietary measurement, total energy intake at 1 y, birth weight z score, breastfeeding, playing sports, household income, maternal BMI at enrollment, education, folic acid use during pregnancy, smoking during pregnancy, diet score, and animal protein.

					Breastfeeding status:<br>Exclusively ≥4 mo: 1005 (28.2%)<br>Partially ≥4 mo: 2263 (63.5%)<br>Never: 296 (8.3%)
**Fruit**					
Wasting	CS	392 participants aged 6-23 months	Indonesia	Frequency of eating fruits was not significantly associated with wasting prevalence (p=0.356).	
Underweight	CS	392 participants aged 6-23 months	Indonesia	Frequency of eating fruits was not significantly associated with underweight prevalence (p=0.995).	
Stunting	CS	392 participants aged 6-23 months	Indonesia	Frequency of eating fruits was not significantly associated with stunting prevalence (p=0.623).	
**Overweight**	Cohort	1,916 participants aged 6-18 months	China	Frequency of eating fruit was significantly associated with overweight prevalence after 12 months of follow-up. 6.36% (n=22) and 18.15% (n=285) of those who consumed fruit <7 times/week (n=346) and ≥ 7 times/week (n=1570) were overweight, respectively (p<0.001 between groups).	
BMI	Cohort	362 participants aged 18 months to 8 years	Australia	Higher intake of fruit at 18 months was not significantly associated with BMI at 8 years old (p=0.11).	Model adjusted by sex, asthma study intervention group, birth weight, breastfeeding for at least 6 months, parental obesity status, ethnicity, smoking in pregnancy,

					father's education, and for total energy intake.<br><br>Breastfed ≥ 6 months:<br>No: 211 (58.3%)<br>Yes: 151 (41.7%)
**Fruit and vegetables combined**					
**Stunting**	CS	13,107 participants aged 6-24 months	China	Frequency of eating vegetables and fruits was significantly associated with stunting prevalence. For those who consumed vegetables and fruits weekly, monthly, and < once per month or none, compared to daily, the odds of being stunted were 1.739 (p=0.00), 1.698 (p=0.03), and 1.768 (p=0.00) times greater, respectively.	
**Underweight**	CS	13,107 participants aged 6-24 months	China	Frequency of eating vegetables and fruits was significantly associated with underweight prevalence. For those who consumed vegetables and fruits weekly, monthly, and < once per month or none, compared to daily, the odds of being underweight were 1.908 (p=0.00), 1.566 (p=0.10), and 1.478 (p=0.01) times greater, respectively.	
**Pulses, legumes, and seeds**					
Wasting	CS	392 participants aged 6-23 months	Indonesia	Frequency of eating legumes was not significantly associated with wasting (p=0.542).	

Wasting	CS	1,907 participants aged 12-59 months	Cambodia	Frequency of eating pulses was not significantly associated with wasting prevalence (p>0.05).	Adjusted by household wealth quintile, education of mother, geographical area, and residential location.
Wasting	CS	1,906 participants aged 12-59 months	Cambodia	Frequency of eating seeds was not significantly associated with wasting prevalence (p>0.05).	Adjusted by household wealth quintile, education of mother, geographical area, and residential location.
Underweight	CS	392 participants aged 6-23 months	Indonesia	Frequency of eating legumes was not significantly associated with being underweight (p=0.174).	
Underweight	CS	1,907 participants aged 12-59 months	Cambodia	Frequency of eating pulses was not significantly associated with underweight prevalence (p>0.05).	Adjusted by household wealth quintile, education of mother, geographical area, and residential location.
Underweight	CS	1,906 participants aged 12-59 months	Cambodia	Frequency of eating seeds was not significantly associated with underweight prevalence (p>0.05).	Adjusted by household wealth quintile, education of mother, geographical area, and residential location.
Stunting	CS	392 participants aged 6-23 months	Indonesia	Frequency of eating legumes was not significantly associated with stunting (p=0.618).	
Stunting	CS	1,907 participants aged 12-59 months	Cambodia	Frequency of eating pulses was not significantly associated with stunting prevalence (p>0.05).	Adjusted by household wealth quintile, education of mother, geographical area, and residential location.
Stunting	CS	1,906 participants	Cambodia	Frequency of eating seeds was not significantly associated with stunting prevalence (p>0.05).	Adjusted by household wealth quintile, education of mother,

		aged 12-59 months			geographical area, and residential location.

Note: Bolded outcomes represent statistically significant findings.

Table 2. Narrative Synthesis Table for Anthropometric Indices and Measurements

Outcome	Study Design	Participants	Country	Narrative Synthesis	**Other notes** (i.e., breastfeeding status, confounders)
**Vegetables**					
BMI Z-score	Cohort	337 participants aged 14 months to 3.7 years	Australia	Consumption of vegetables at age 14 months was not significantly associated with BMI z-score at age 3.7 years (p=0.12).	
**HAZ**	CS	165 participants aged 6-42 months	Senegal	There was a borderline difference from less frequent vegetable/leaves consumption with HAZ, where those who consumed vegetables/leaves 0-2 days/week and ≥ 3 days/week had a mean HAZ of -1.01 (p=0.052) and -0.59 (p<0.06), respectively, pointing to a trend of lower HAZ among children who consume vegetables less frequently.	Adjusted model by child age.
Waist circumference	Cohort	362 participants aged 18 months to 8 years	Australia	Higher intake of vegetables at 18 months was not significantly associated with waist circumference at 8 years old (p=0.07).	Model adjusted by total energy intake.<br><br>Breastfed ≥ 6 months:<br>No: 211 (58.3%)<br>Yes: 151 (41.7%)
**Linear Growth**	CS	165 participants	Senegal	Frequent consumption of vegetables had a statistically significant inverse	Adjusted model by child age, sex, malaria study intervention group,

		aged 6-42 months		relationship to linear growth (means: 8.3cm and 7.4cm height increment over the preceding 7 months for rare and frequent consumption, respectively, p=0.041).	maternal height, BMI, schooling, and number of children 5 y old.
Height	Cohort	3,564 participants aged 1-9 years	Netherlands	Higher intake of vegetable protein in early childhood (1 year old) was not significantly associated with height measures in later childhood (between the ages of 1 and 9 years old) (p>0.05).	Model adjusted for child sex, ethnicity, age at dietary measurement, total energy intake at 1 y, birth weight z score, breastfeeding, playing sports, household income, maternal BMI at enrollment, education, folic acid use during pregnancy, smoking during pregnancy, diet score, and animal protein.<br><br>Breastfeeding status:<br>Exclusively ≥4 mo: 1005 (28.2%)<br>Partially ≥4 mo: 2263 (63.5%)<br>Never: 296 (8.3%)
Weight	Cohort	3,564 participants aged 1-9 years	Netherlands	Higher intake of vegetable protein in early childhood (1 year old) was not significantly associated with weight measures in later childhood (between the ages of 1 and 9 years old) (p>0.05).	Model adjusted for child sex, ethnicity, age at dietary measurement, total energy intake at 1 y, birth weight z score, breastfeeding, playing sports, household income, maternal BMI at enrollment, education, folic acid use during pregnancy, smoking during pregnancy, diet score, and animal protein.

					Breastfeeding status:<br>Exclusively ≥4 mo: 1005 (28.2%)<br>Partially ≥4 mo: 2263 (63.5%)<br>Never: 296 (8.3%)
**Fruit**					
BMI Z-score	Cohort	337 participants aged 14 months to 3.7 years	Australia	Variety of fruits tried at age 14 months was not significantly associated with BMI z-score at age 3.7 years (p=0.78).	
**HAZ**	CS	165 participants aged 6-42 months	Senegal	There was a borderline difference from less frequent fruit consumption with HAZ, where those who consumed fruit 0-2 days/week and ≥ 3 days/week had a mean HAZ of -1.04 (p=0.051) and -0.71 (p=0.059), respectively,<br>pointing to a trend of lower HAZ among children who consume fruits less frequently.	Adjusted model by child age.
**Waist Circumference**	Cohort	362 participants aged 18 months to 8 years	Australia	Higher intake of fruit at 18 months was significantly associated with waist circumference at 8 years old. In the adjusted model, children who consumed increasing quintiles of fruit (0g, 17g, 45g, 80g, 124g) were significantly more likely to have a larger waist circumference by 0.63cm (Reg coefficient=0.63, 95% CI: 0.05-1.20, p=0.03).	Model adjusted by total energy intake.<br><br>Breastfed ≥ 6 months:<br>No: 211 (58.3%)<br>Yes: 151 (41.7%)

**Linear Growth**	CS	165 participants aged 6-42 months	Senegal	Fruit consumption was positively associated with linear growth in fully adjusted models (means: 7.9cm and 8.7cm height increment over the preceding 7 months for rare and frequent consumption, respectively, p=0.027).	Adjusted model by child age, sex, malaria study intervention group, maternal height, BMI, schooling, and number of children 5 y old.
**BMI Z-score**	Cohort	1,499 participants aged 18 months	Sweden	Fruit juice consumption at 18 months of age was negatively associated with BMI-z score at 18 months of age. The mean BMIz was 0.72 ± 0.92 for non-consumers, 0.61 ± 0.91 for seldom consumers and 0.45 ± 0.98 for regular consumers (p=0.003 between groups).	
**Maize/cowpea**					
**Length**	RCT	90 participants aged 6-18 months	Nigeria	In both boys and girls, amount of maize/cowpea consumption was significantly associated with mean length after 3,6,9, and 12 months of follow-up.<br><br>Boys who consumed maize/cowpea, and those who had no consumption, had mean length 70.65cm and 67.52cm at 3 months of follow-up, 74.69cm and 71.78cm at 6 months of follow-up, 76.05cm and 72.47cm at 9 months of follow-up, and 79.23cm and 74.21cm at 12 months of follow-up, respectively (p<0.05 between groups).	

				Girls who consumed maize/cowpea, and those who had no consumption, had mean length 67.51cm and 65.43cm at 3 months follow-up, 71.93cm and 67.72cm at 6 months follow-up, 76.42cm and 69.24cm at 9 months follow-up, and 78.15cm and 73.23cm at 12 months follow-up, respectively (p<0.05 between groups).	
**Weight**	RCT	90 participants aged 6-18 months	Nigeria	In both boys and girls, amount of maize/cowpea consumption was significantly associated with mean weight after 3, 6, 9, and 12 months of follow-up.<br><br>Boys who consumed maize/cowpea, and those who had no consumption, had mean weight 7.78 kg and 5.56 kg at 3 months of follow-up, 9.82kg and 5.05kg at 6 months of follow-up, 10.12kg and 6.08kg at 9 months of follow-up, and 10.51kg and 6.6kg at 12 months of follow-up, respectively (p<0.05 between groups).<br><br>Girls who consumed maize/cowpea, and those who had no consumption, had mean weight 8.2kg and 6.15kg at 3 months follow-up, 9.19kg and 6.41kg at 6 months follow-up, 10.32kg	

				and 6.53kg at 9 months follow-up, and 9.62kg and 7.07kg at 12 months follow-up, respectively ($p<0.05$ between groups).	

Note: Bolded outcomes represent statistically significant findings.

Table 3. Narrative Synthesis Table for Nutrient Status or Intake

Outcome	Study Design	Participants	Country	Narrative Synthesis	**Other notes** (i.e., breastfeeding status, confounders)
**Vegetables**					
**Iron stores (Ferritin >20µg/L)**	CS	74 participants aged 1 year old	Norway	Those who consumed vegetables once/day, compared to <once/day, were significantly more likely to have low iron stores (ferritin values <20µg/L) (Reg coefficient= -2.7, p=0.02). Although the results suggest a negative effect of feeding vegetables once/day, findings were inconsistent as this relationship was not seen for those eating vegetables more frequently (several times/day) compared to <once/day. Additionally, there was no significant relationship observed between the feeding frequency of vegetables and the likelihood of having very low iron stores (ferritin <15µg/L).	
**Hemoglobin levels**	CS	796 participants aged 18 months	United Kingdom	In children aged 18 months, mean hemoglobin levels were significantly higher in children who consumed any vegetables when compared to those	Adjusted for sex.

				who had no vegetable consumption (p=0.026). For boys, those who consumed any vegetables, and no vegetables, had a mean hemoglobin level of 11.7g/l (SD=1.0) and 11.3g/l (SD=0.9), respectively. For girls, those who consumed any vegetables, and no vegetables, had a mean hemoglobin level of 11.8g/l (SD=0.9) and 11.6g/l (SD=0.8), respectively.	
Anemia	CS	205 participants aged 6-12 months	Brazil	Frequency of eating dark green vegetables was not significantly associated with anemia prevalence (p=0.502).	Anemia= Hemoglobin <11 g/dL.<br><br>Exclusive breastfeeding time (Days):<br>≥60: 62 (anemia); 52 (non-anemia)<br><60: 56 (anemia); 35 (non-anemia)
Branched-chain amino acid levels	Cohort	54 participants aged 12 months	Thailand	There was no association between plant-based protein intake at 12 months with plasma BCAA levels at 12 months (p>0.05).	Adjusted for gender, type of milk feeding, and energy intake.<br><br>Breast milk only at 12 months: 46 (31.7%)<br>Formula/cow's milk only at 12 months: 74 (51.0%)<br>Combined at 12 months: 25 (17.2%)
Essential amino acid levels	Cohort	54 participants aged 12 months	Thailand	There was no association between plant-based protein intake at 12 months with plasma EAA levels at 12 months (p>0.05).	Adjusted for gender, type of milk feeding, and energy intake.<br><br>Breast milk only at 12 months: 46 (31.7%)<br>Formula/cow's milk only at 12 months: 74 (51.0%)<br>Combined at 12 months: 25 (17.2%)

Non-essential amino acid levels	Cohort	54 participants aged 12 months	Thailand	There was no association between plant-based protein intake at 12 months with plasma NEAA levels at 12 months (p>0.05).	Adjusted for gender, type of milk feeding, and energy intake.<br><br>Breast milk only at 12 months: 46 (31.7%)<br>Formula/cow's milk only at 12 months: 74 (51.0%)<br>Combined at 12 months: 25 (17.2%)
Total amino acid levels	Cohort	54 participants aged 12 months	Thailand	There was no association between plant-based protein intake at 12 months with total amino acid levels at 12 months (p>0.05).	Adjusted for gender, type of milk feeding, and energy intake.<br><br>Breast milk only at 12 months: 46 (31.7%)<br>Formula/cow's milk only at 12 months: 74 (51.0%)<br>Combined at 12 months: 25 (17.2%)
**Fruit**					
**Hemoglobin levels**	CS	796 participants aged 18 months	United Kingdom	In both boy and girls aged 18 months, mean hemoglobin levels were significantly higher for those who consumed any fruit when compared to those who consumed no fruit (p=0.028). For boys, those who consumed any citrus fruit, and no citrus fruit, had a mean hemoglobin level of 11.8g/l (SD=1.1) and 11.6g/l (SD=0.9), respectively. For girls, those who consumed any citrus fruit, and no citrus fruit, had a mean hemoglobin level of 11.9g/l (SD=1.0) and 11.7g/l (SD=0.9), respectively.	The models for any fruit consumption were adjusted by sex.

				For boys, those who consumed any fruit, and no fruit, had a mean hemoglobin level of 11.7g/l (SD=0.9) and 11.5g/l (SD=1.2), respectively. For girls, those who consumed any fruit, and no fruit, had a mean hemoglobin level of 11.8g/l (SD=0.9) and 11.5g/l (SD=0.8), respectively.	
**Hemoglobin levels**	CS	179 participants aged 6-12 months	Brazil	Fruit consumption was negatively associated with hemoglobin levels in the adjusted model. Children who consumed fruit, compared to no consumption, were significantly more likely to have lower hemoglobin levels by 2g/dl (Reg coefficient= -2.00, SE= 0.56, p<0.001).	Adjusted model by duration of gestation, mother's schooling, child's sex, anthropometric status based on the height-for-age indicator, and age (squared).
**Anemia**	CS	205 participants aged 6-12 months	Brazil	Fruit consumption was not significantly associated with anemia prevalence when considering consumption in the past 24 hours. When looking at daily vs < than daily frequencies, in the adjusted model, fruit consumption was significantly associated with anemia prevalence. For those who consumed fruit < daily, compared to daily, the odds were 1.88 times greater (95% CI: 1.03-3.42, p=0.003) of having anemia.	Anemia= Hemoglobin <11 g/dL.<br><br>Adjusted model by family income per capita and consumption of medicated iron supplements.<br><br>Exclusive breastfeeding time (Days) ≥60: 62 (anemia); 52 (non-anemia) <60: 56 (anemia); 35 (non-anemia).
**Vitamin C intake**	CS	2,193 participants	Canada	Mean daily vitamin C intake was significantly higher with increased frequency of 100% fruit juice	Ratio adjustment for intake day, categorized as weekend/weekday, and energy intake.

		aged 1-3 years old		consumption in boys and girls aged 1-3 years. Means of 70mg (SE=6.4), 124mg (SE=6), and 180mg (SE=6.2) for consuming fruit juice <0.5times/day, 0.5 to <1.5 times/day, and 1.5 times/day, respectively (p<0.05 between groups).	
**Magnesium intake**	CS	2,193 participants aged 1-3 years old	Canada	More frequent consumption of 100% fruit juice was associated with higher intake of magnesium and lower prevalence of inadequate magnesium intake among the most versus least frequent consumers of fruit juice in children aged 1-3 years. Means of 198mg (SE=7.3), 213mg (SE=5.2), and 221mg (SE=5.5) for consuming fruit juice <0.5times/day, 0.5 to <1.5 times/day, and 1.5 times/day, respectively (p<0.05 between groups).	Ratio adjustment for intake day, categorized as weekend/weekday, and energy intake.
Vitamin A intake	CS	2,193 participants aged 1-3 years old	Canada	There was no association between consumption of 100% fruit juice with vitamin A intake (p>0.05).	Ratio adjustment for intake day, categorized as weekend/weekday, and energy intake.
Vitamin D	CS	2,193 participants aged 1-3 years old	Canada	There was no association between consumption of 100% fruit juice with vitamin D intake (p>0.05).	Ratio adjustment for intake day, categorized as weekend/weekday, and energy intake.
Calcium	CS	2,193 participants	Canada	There was no association between consumption of 100% fruit juice with calcium intake (p>0.05).	Ratio adjustment for intake day, categorized as weekend/weekday, and energy intake.

		aged 1-3 years old			
**Legumes and beans**					
**Energy intake**	CS	337 participants aged 6-23 months	Ghana	Amount of legume consumption was significantly associated with intake of energy. Children who consumed legumes (median daily legume intake: 19.8g ± 31.3), and those who had no consumption, had a median energy intake of 893 kcal (25th, 75th percentile=726,1142) and 596 kcal (25th, 75th percentile=521,688), respectively (p<0.05 between groups).	All children were breastfed except for n=29 children aged 12-23 months old.
Energy intake	RCT	197 participants aged 6-23 months	Ethiopia	Amount of broad bean consumption was not significantly associated with intake of energy after 6 months of follow-up (p>0.05).	At baseline, 99% of control children and 91% of intervention children were currently breastfeeding.<br>At endline, 90.4% of control children and 83% of intervention children were currently breastfeeding.
**Fat intake**	CS	337 participants aged 6-23 months	Ghana	Amount of legume consumption was significantly associated with intake of fat. Children who consumed legumes (median daily legume intake: 19.8g ± 31.3), and those who had no consumption, had a median fat intake of 33g (25th, 75th percentile=28,39) and 27g (25th, 75th percentile=26,29), respectively (p<0.05 between groups).	All children were breastfed except for n=29 children aged 12-23 months old.

Fat intake	RCT	197 participants aged 6-23 months	Ethiopia	Amount of broad bean consumption was not significantly associated with intake of fat after 6 months of follow-up ($p>0.05$).	At baseline, 99% of control children and 91% of intervention children were currently breastfeeding.<br>At endline, 90.4% of control children and 83% of intervention children were currently breastfeeding.
**Protein intake**	CS	337 participants aged 6-23 months	Ghana	Amount of legume consumption was significantly associated with intake of protein. Children who consumed legumes (median daily legume intake: 19.8g ± 31.3), and those who had no consumption, had a median protein intake of 21g (25th, 75th percentile=15,28) and 11g (25th, 75th percentile=10,14), respectively ($p<0.05$ between groups).	All children were breastfed except for n=29 children aged 12-23 months old.
**Protein intake**	RCT	197 participants aged 6-23 months	Ethiopia	Amount of broad bean consumption was significantly associated with intake of protein after 6 months of follow-up. Children who consumed broad beans, and those who had no consumption, had a mean protein intake of 28.7g (SD=22.6) and 21.6g (SD= 11.3), respectively ($p<0.05$ between groups).	At baseline, 99% of control children and 91% of intervention children were currently breastfeeding.<br>At endline, 90.4% of control children and 83% of intervention children were currently breastfeeding.
**Iron intake**	CS	337 participants aged 6-23 months	Ghana	Amount of legume consumption was significantly associated with intake of iron. Children who consumed legumes (median daily legume intake: 19.8g ± 31.3), and those who had no consumption, had a median iron	All children were breastfed except for n=29 children aged 12-23 months old.

				intake of 5.6mg (25th, 75th percentile= 3.5, 8.5) and 2mg (25th, 75th percentile=1.5, 3), respectively (p<0.05 between groups).	
**Iron intake**	RCT	197 participants aged 6-23 months	Ethiopia	Amount of broad bean consumption was significantly associated with intake of iron after 6 months of follow-up. Children who consumed broad beans, and those who had no consumption, had a mean iron intake of 30.6mg (SD=21.2) and 20.9mg (SD= 13.4), respectively (p<0.05 between groups).	At baseline, 99% of control children and 91% of intervention children were currently breastfeeding.<br>At endline, 90.4% of control children and 83% of intervention children were currently breastfeeding
**Carbohydrate intake**	RCT	197 participants aged 6-23 months	Ethiopia	Amount of broad bean consumption was significantly associated with intake of carbohydrates after 6 months of follow-up. Children who consumed broad beans, and those who had no consumption, had a mean carbohydrate intake of 159g (SD=105) and 127g (SD= 78), respectively (p<0.05).	At baseline, 99% of control children and 91% of intervention children were currently breastfeeding.<br>At endline, 90.4% of control children and 83% of intervention children were currently breastfeeding
**Calcium intake**	CS	337 participants aged 6-23 months	Ghana	Amount of legume consumption was significantly associated with intake of calcium. Children who consumed legumes (median daily legume intake: 19.8g ± 31.3), and those who had no consumption, had a median calcium intake of 230mg (25th, 75th percentile=200,273) and 194mg (25th,	All children were breastfed except for n=29 children aged 12-23 months old.

				75th percentile=187,223), respectively ($p<0.05$ between groups).	
**Folate intake**	CS	337 participants aged 6-23 months	Ghana	Amount of legume consumption was significantly associated with intake of folate. Children who consumed legumes (median daily legume intake: 19.8g ± 31.3), and those who had no consumption, had a median folate intake of 91ug (25th, 75th percentile=76,121) and 63ug (25th, 75th percentile=60,69), respectively ($p<0.05$ between groups).	All children were breastfed except for n=29 children aged 12-23 months old.
**Niacin intake**	CS	337 participants aged 6-23 months	Ghana	Amount of legume consumption was significantly associated with intake of niacin. Children who consumed legumes (median daily legume intake: 19.8g ± 31.3), and those who had no consumption, had a median niacin intake of 4.7mg (25th, 75th percentile= 3.2, 6.7) and 1.9mg (25th, 75th percentile= 1.5, 2.4), respectively ($p<0.05$ between groups).	All children were breastfed except for n=29 children aged 12-23 months old.
**Zinc intake**	CS	337 participants aged 6-23 months	Ghana	Amount of legume consumption was significantly associated with intake of zinc. Children who consumed legumes (median daily legume intake: 19.8g ± 31.3), and those who had no consumption, had a median zinc intake of 3.2mg (25th, 75th percentile= 2.3, 4.5) and 1.6mg (25th, 75th	All children were breastfed except for n=29 children aged 12-23 months old.

				percentile= 1.4, 2.1), respectively (p<0.05 between groups).	
**Hemoglobin levels**	CS	179 participants aged 6-12 months	Brazil	Bean consumption was positively associated with hemoglobin levels in the adjusted model. Children who consumed beans, compared to no consumption, were significantly more likely to have higher hemoglobin levels by 0.56g/dl (Reg coefficient= 0.56, SE= 0.23, p=0.018).	Adjusted model by duration of gestation, mother's schooling, child's sex, anthropometric status based on the height-for-age indicator, and age (squared).
Anemia	CS	205 participants aged 6-12 months	Brazil	Consumption of beans was not significantly associated with anemia prevalence (p=0.550).	Anemia= Hemoglobin <11 g/dL.<br><br>Exclusive breastfeeding time (Days):<br>≥60: 62 (anemia); 52 (non-anemia)<br><60: 56 (anemia); 35 (non-anemia)

Note: Bolded outcomes represent statistically significant findings.

Table 4. Narrative Synthesis Table for Child Development

Outcome	Study Design	Participants	Country	Narrative Synthesis	**Other notes** (i.e., breastfeeding status, confounders)
**Vegetables**					
**Child development: total ASQ-3 score**	Cohort	282 participants aged 15-38 months	Nepal	Frequency of eating vegetables at ~15 months of age was significantly associated with total child development score at 23-38 months of age. In the adjusted linear model, each additional day of vegetable consumption was significantly associated with a 9.3 point higher total score on the ASQ-3 (Reg	Both models adjusted by maternal education (2 categories), wealth quintile, child age, and randomization of intervention group.

				coefficient=9.3, 95% CI: 2.4-16.3, p<0.01).<br><br>In the adjusted logistic regression model, each additional day of vegetable consumption was significantly associated with a 40% lower risk of falling into the lowest 25% group of the total ASQ-3 distribution (OR=0.6, 95% CI: 0.41-0.90, p=0.01).	
**Child development: total ASQ-3 score**	Cohort	282 participants aged 15-38 months	Nepal	Frequency of eating green leafy vegetables at ~15 months of age was significantly associated with total child development score at 23-38 months of age. In the adjusted linear model, each additional day of green leafy vegetable consumption was significantly associated with a 11.7 point higher total score on the ASQ-3 (Reg coefficient=11.7, 95% CI: 4.1-19.4, p<0.01).<br><br>In the adjusted logistic regression model, each additional day of green leafy vegetable consumption was significantly associated with a 46% lower risk of falling into the lowest 25% group of the total ASQ-3 distribution (OR=0.54, 95% CI: 0.34-0.86, p<0.01).	Both models adjusted by maternal education (2 categories), wealth quintile, child age, and randomization of intervention group.

**Child development: communication skills**	Cohort	282 participants aged 15-38 months	Nepal	Frequency of eating vegetables at ~15 months of age was significantly associated with communication score at 23-38 months of age. Each additional day of vegetable consumption was significantly associated with a 31% lower risk of a low score on the communication subscale (OR=0.69, 95% CI: 0.47-1.00, p<0.05).	Model adjusted by maternal education (2 categories), wealth quintile, child age, and randomization of intervention group.
Child development: communication skills	Cohort	282 participants aged 15-38 months	Nepal	Frequency of eating green leafy vegetables at ~15 months was not significantly associated with communication score at 23-38 months of age (p=0.16).	Model adjusted by maternal education (2 categories), wealth quintile, child age, and randomization of intervention group.
Child development: gross motor skills	Cohort	282 participants aged 15-38 months	Nepal	Frequency of eating vegetables at ~15 months old was not significantly associated with gross motor score at 23-38 months of age (p=0.76).	Model adjusted by maternal education (2 categories), wealth quintile, child age, and randomization of intervention group.
Child development: gross motor skills	Cohort	282 participants aged 15-38 months	Nepal	Frequency of eating green leafy vegetables at ~15 months old was not significantly associated with gross motor score at 23-38 months of age (p=0.48).	Model adjusted by maternal education (2 categories), wealth quintile, child age, and randomization of intervention group.
**Child development: fine motor skills**	Cohort	282 participants aged 15-38 months	Nepal	Frequency of eating vegetables at ~15 months old was significantly associated with fine motor score at 23-38 months of age. Each additional day	Model adjusted by maternal education (2 categories), wealth quintile, child age, and

				of vegetable consumption was significantly associated with a 40% lower risk of a low score on the fine motor subscale (OR=0.60, 95% CI: 0.42-0.86, p<0.01).	randomization of intervention group.
**Child development: fine motor skills**	Cohort	282 participants aged 15-38 months	Nepal	Frequency of eating green leafy vegetables at ~15 months old was significantly associated with fine motor score at 23-38 months of age. Each additional day of green leafy vegetable consumption was significantly associated with a 38% lower risk of a low score on the fine motor subscale (OR=0.62, 95% CI: 0.42-0.93, p=0.02).	Model adjusted by maternal education (2 categories), wealth quintile, child age, and randomization of intervention group.
Child development: problem-solving skills	Cohort	282 participants aged 15-38 months	Nepal	Frequency of eating vegetables at ~15 months old was not significantly associated with problem-solving score at 23-38 months of age (p=0.09).	Model adjusted by maternal education (2 categories), wealth quintile, child age, and randomization of intervention group.
**Child development: problem-solving skills**	Cohort	282 participants aged 15-38 months	Nepal	Frequency of eating green leafy vegetables at ~15 months old was significantly associated with problem-solving score at 23-38 months of age. Each additional day of green leafy vegetable consumption was significantly associated with a 36% lower risk of a low score on the problem-solving subscale (OR=0.64, 95% CI: 0.44-0.93, p=0.02).	Model adjusted by maternal education (2 categories), wealth quintile, child age, and randomization of intervention group.

**Child development: personal-social skills**	Cohort	282 participants aged 15-38 months	Nepal	Frequency of eating vegetables at ~15 months old was significantly associated with personal-social score at 23-38 months of age. Each additional day of vegetable consumption was associated with a 22% lower risk of a low score on the personal-social subscale (OR=0.78, 95% CI: 0.54-1.13, p=0.02).	Model adjusted by maternal education (2 categories), wealth quintile, child age, and randomization of intervention group.
**Child development: personal-social skills**	Cohort	282 participants aged 15-38 months	Nepal	Frequency of eating green leafy vegetables at ~15 months old was significantly associated with personal-social score at 23-38 months of age. Each additional day of green leafy vegetable consumption was associated with a 41% lower risk of a low score on the personal-social subscale (OR=0.59, 95% CI: 0.38-0.91, p=0.02).	Model adjusted by maternal education (2 categories), wealth quintile, child age, and randomization of intervention group.

Note: Bolded outcomes represent statistically significant findings.

Table 5. Narrative Synthesis Table for Subsequent consumption of food items later in life

Outcome	Study Design	Participants	Country	Narrative Synthesis	**Other notes** (i.e., breastfeeding status, confounders)
**Vegetables**					
**Stability and Change in Vegetable Consumption**	Cohort	9,490 participants aged 18 months to 7 years	Norway	In boys, overall vegetable consumption at 18 months was positively associated with overall vegetable consumption at 36 months (spearman's rho=0.36) and at 7 years of age (spearman's rho=0.28).	

				In girls, overall vegetable consumption at 18 months was positively associated with overall vegetable consumption at 36 months (spearman's rho=0.37) and at 7 years of age (spearman's rho=0.31).	
**Intake of new foods**	Quasi-experimental	147 participants aged 5-7 months	Germany and France	There was a significant association for type of variety experience with the high vegetable variety producing the greatest increase in intake of new foods (p<0.0001) at study endline (~2 months).	Breastfed infants (>30 days): n=83<br>Formula-fed infants (breastfed <15 days):<br>n=64
**Mean number of vegetables eaten by infants**	Quasi-experimental	107 participants aged 5 months to 6 years	Germany and France	At follow-up 3 (~67 months), early variety experience was significantly associated with mean number of vegetables eaten (p<0.05), but not at follow up 1 (~9 months) (p=0.20) or 2 (~31 months) (p=0.0635).<br><br>At follow up 3 (~67 months), children who had experienced a high variety of vegetables at weaning ate more of the new vegetables and familiar vegetables than those who had experienced low or no variety (14.1g ± 1.5 vs. 4.3g ± 1.5 and 3.2g ± 1.4, p<0.0001 for new vegetables, respectively; and 9.6g ± 2.0 vs. 13.1g ± 2.0 and 13.1g ± 1.9, p=0.03 for familiar vegetables, respectively).	Breastfed infants (>30 days): n=61<br>Formula-fed infants (breastfed <15 days):<br>n=45

**Vegetable and fruit intake**	Cohort	333 participants aged 14 months to 3.7 years	Australia	A greater variety of vegetables tried at age 14 months was significantly associated with a higher fruit and vegetable intake score at age 3.7 years (Reg coefficient=0.12, p=0.05).	
**Fruit**					
**Stability and Change in Fruit Consumption**	Cohort	9,490 participants aged 18 months to 7 years	Norway	In boys, overall fruit consumption at 18 months was positively associated with overall fruit consumption at 36 months (spearman's rho=0.36) and at 7 years of age (spearman's rho=0.23). In girls, overall fruit consumption at 18 months was positively associated with overall fruit consumption at 36 months (spearman's rho=0.36) and at 7 years of age (spearman's rho=0.24).	
**Fruit and vegetable intake**	Cohort	333 participants aged 14 months to 3.7 years	Australia	A greater variety of fruits tried at age 14 months was significantly associated with a higher fruit and vegetable intake score at age 3.7 years (Reg coefficient=0.19, p=0.003).	
**Fruit and vegetables combined**					
**Stability and tracking of FV patterns**	Cohort	231 participants aged 9-24 months	Nepal	There was moderate stability for the frequency of consumption of yellow fruits and vegetables and dark green leafy vegetable consumption using GEE models (stability coefficient=0.26, 95% CI: 0.18-0.35).	Models adjusted for the Water, Assets, Mother;s Education and Income (WAMI) Index, maternal age, parity and child's gender. Since these adjustments made no further changes to the estimates, only results from the unadjusted

				For intake of yellow fruits and vegetables and dark green leafy vegetables, tracking coefficients were mostly fair and decreased throughout follow-up from 0.27 for the 13–16-month time slot to 0.19 for the 21–24-month time slot (K_w=0.273 for 13-16 months; K_w=0.234 for 17-20 months; K_w=0.194 for 21-24 months).	models and those adjusted for WAMI are presented.<br><br>Breastfeeding status: 99.9% of infants were breastfeeding at baseline

Note: Bolded outcomes represent statistically significant findings.

Table 6. Narrative Synthesis Table for Morbidity: Diarrhea Prevalence

Outcome	Study Design	Participants	Country	Narrative Synthesis	**Other notes** (i.e., breastfeeding status, confounders)
			**Vegetables**		
Diarrhea	CS	4,026 participants aged 12-59 months	Cambodia	Frequency of eating green leafy or orange color vegetables was not significantly associated with diarrhea prevalence (p>0.05).	Adjusted by household wealth quintile, education of mother, geographical area, and residential location.
			**Pulses and seeds**		
Diarrhea	CS	4,026 participants aged 12-59 months	Cambodia	Frequency of eating pulses was not significantly associated with diarrhea prevalence (p>0.05).	Adjusted by household wealth quintile, education of mother, geographical area, and residential location.
Diarrhea	CS	4,027 participants aged 12-59 months	Cambodia	Frequency of eating seeds was not significantly associated with diarrhea prevalence (p>0.05).	Adjusted by household wealth quintile, education of mother, geographical area, and residential location.

Note: Bolded outcomes represent statistically significant findings.

Table 7. Narrative Synthesis Table for food/taste preferences later in life

Outcome	Study Design	Participants	Country	Narrative Synthesis	**Other notes** (i.e., breastfeeding status, confounders)
**Vegetables**					
**Infant's liking of vegetables**	Quasi-experimental	147 participants aged 5-7 months	Germany and France	There was a significant association for variety experience with high variety of vegetables producing the highest liking scores reported by both mothers and observers ($p<0.0001$) at endline (~2 months).	Breastfed infants (>30 days): n=83<br>Formula-fed infants (breastfed <15 days):<br>n=64
**Infant's liking of fruit and vegetables**	Cohort	340 participants aged 14 months to 3.7 years	Australia	A greater variety of vegetables tried at age 14 months was significantly associated with liking a greater number of fruits (Reg coefficient=0.14, p=0.022) and vegetables (Reg coefficient= 0.15, p=0.017) at age 3.7 years.	
**Mean number of vegetables liked by infants**	Quasi-experimental	107 participants aged 5 months to 6 years	Germany and France	At follow-up 3 (~67 months), early variety experience was significantly associated with mean number of vegetables liked ($p<0.05$), but not at follow-up 1 (~9 months) (p=0.20) or 2 (~31 months) (p=0.0635).<br><br>At follow-up 3 (~67 months), children who had experienced a high variety of vegetables at weaning liked the new vegetables and familiar vegetables more than those who had low or no variety (scores of 4.4 ± 0.3 vs. 2.5 ± 0.3 and 2.9± 0.3, p=0.0002 for new vegetables, respectively; and scores of	Breastfed infants (>30 days): n=61<br>Formula-fed infants (breastfed <15 days):<br>n=45

				5.1 ± 0.2 vs. 4.2 ± 0.2 and 4.4 ± 0.2, p=0.03 for familiar vegetables, respectively).	
**Infant's liking of vegetables**	RCT	36 participants aged 5-12 months	United Kingdom	At 6 months follow-up, liking for vegetables was significantly different between intervention and control children (p=0.029).<br>Children who consumed carrots more frequently (nine times over the 35-day intervention), compared to less frequent consumption (three times over the 35-day intervention), had a mean infant liking of 7.14 (SEM=0.53) and 5.69 (SEM=0.49), respectively (p=0.05).<br>Children who consumed green beans more frequently (nine times over the 35-day intervention), compared to less frequent consumption (three times over the 35-day intervention), had a mean infant liking of 6.14 (SEM=0.62) and 4.56 (SEM=0.58), respectively (p=0.07).	
**Willingness to taste**	Quasi-experimental	107 participants aged 5 months to 6 years	Germany and France	At follow-up 3 (~67 months), early variety experience was significantly associated with mean willingness to taste vegetables (p<0.05).<br>Children who had experienced a high variety of vegetables at weaning, tasted more vegetables than those who had experienced low or no variety	Breastfed infants (>30 days): n=61<br>Formula-fed infants (breastfed <15 days):<br>n=45

				(8.9 ± 0.6 vs. 5.6 ± 0.6 and 5.6 ± 0.6, p=0.0001, respectively) after 67 months of follow-up.	
**Fussiness**	Cohort	339 participants aged 14 months to 3.7 years	Australia	Having tried fewer vegetables at age 14 months was significantly associated with a higher fussiness score at age 3.7 years (Reg coefficient= -0.12, p=0.03).	Adjusted for fussiness score at age 14 months.
**Fruit**					
**Infant's liking of fruit and vegetables**	Cohort	340 participants aged 14 months to 3.7 years	Australia	A greater variety of fruits tried at 14 months was significantly associated with liking a greater number of fruits (Reg coefficient=0.16, p=0.007) at age 3.7 years. However, number of fruits tried at 14 months was not significantly associated with liking vegetables at age 3.7 years (p=0.12).	
Fussiness	Cohort	339 participants aged 14 months to 3.7 years	Australia	Consumption of fruits at age 14 months was not significantly associated with fussiness score at age 3.7 years (p=0.72).	Adjusted for fussiness score at age 14 months.

Note: Bolded outcomes represent statistically significant findings.

S2.7. GRADE Assessments for FV and NPS Studies

Table S2.7.1: GRADE table for vegetables and anthropometric outcomes

Question: Is more frequent, varied, or amounts of consumption of vegetables, compared to less, associated with beneficial anthropometric outcomes?

Certainty assessment							Impact	Certainty	Importance
№ of studies	Study design	Risk of bias	Inconsistency	Indirectness	Imprecision	Other considerations			
Wasting (Assessed with: Ahmad 2018 measured body weight using a portable Tanita digital scale and recumbent length using an infant length board. Darapheak 2013 measured weight using a lightweight electronic SECA scale and height using a board made by Shorr Productions. For both studies, wasting was defined as weight for length z-score less than 2 SD below the mean).									
2	observational studies	serious[a]	not serious	not serious	serious[b]	none	In both studies, the frequency of eating green leafy or orange vegetables was not significantly associated with wasting prevalence (p>0.05).[c]	⨁◯◯◯ Very low	CRITICAL
Underweight (Assessed with: Ahmad 2018 measured body weight using a portable Tanita digital scale and age was determined through interview with the infant's mother. Darapheak 2013 measured weight using a lightweight electronic SECA scale and age was determined through caregiver interview. For both studies, underweight was defined as weight for age z-score less than 2 SD below the mean).									
2	observational studies	serious[a]	not serious	not serious	serious[d]	none	In both studies, the frequency of eating green leafy or orange vegetables was not significantly associated with underweight prevalence (p>0.05).[c]	⨁◯◯◯ Very low	CRITICAL
Stunting (Assessed with: Ahmad 2018 measured recumbent length using an infant length board and age was determined through interview with the infant's mother. Darapheak 2013 measured height using a board made by Shorr Productions and age was determined through caregiver interview. For both studies, stunting was defined as height for age z-score less than 2 SD below the mean).									
2	observational studies	serious[a]	not serious	not serious	serious[e]	none	In both studies, the frequency of eating green leafy or orange vegetables was not significantly associated with stunting prevalence (p>0.05).[c]	⨁◯◯◯ Very low	CRITICAL
Overweight (Follow-up: 12 months; Assessed with: WHO 2006 Child Growth standards for Children 0-5 years old by calculating BMI percentiles. BMI ≥85 percentile of age was defined as overweight. Overweight prevalence was assessed at 18 months of age).									

Certainty assessment							Impact	Certainty	Importance
№ of studies	Study design	Risk of bias	Inconsistency	Indirectness	Imprecision	Other considerations			
1	observational studies	serious[f]	serious[g]	not serious	serious[h]	none	Frequency of eating vegetables was significantly associated with overweight prevalence after 12 months of follow-up. 2.99% (n=7) and 17.84% (n=300) of those who consumed vegetables <7 times/week and ≥ 7 times/week were overweight, respectively (p<0.001 between groups).[c]	⨁◯◯◯ Very low	CRITICAL
Body Mass Index (Follow-up: up to 96 months; Assessed with: Garden 2011 measured height using a portable stadiometer and weight was measured using electronic bathroom scales at 8 years of age. Braun 2016 measured height using a Harpenden stadiometer (Holtain Limited) and weight using a mechanical personal scale (SECA) at 8 different time points between the ages of 13 months and 9 years of age. For both studies, BMI was calculated in kilograms per meters squared).									
2	observational studies	serious[i]	not serious	not serious	not serious	None	For both studies, higher intake of vegetables in early childhood was not significantly associated with BMI measures in later childhood (p>0.05).[j]	⨁◯◯◯ Very low	IMPORTANT

Explanations

a. NIH tool for observational studies used. Ahmad 2018 was rated as good quality given the study had a low risk of selection bias, information bias, and measurement bias. Darapheak 2013 was rated as fair quality due to a lack of description provided for children who were included in the analysis. Both studies employed a cross-sectional design and thus lack ability to make causal conclusions.

b. Ahmad 2018 did not provide confidence intervals, limiting the ability to make a judgement about imprecision, and the sample size is relatively small (n=392). Darapheak 2013 reported a wide confidence interval around the effect (OR=1.18, 95% CI: 0.83,1.68), however the sample size is quite large (n=1,907). We have downgraded the certainty of evidence for this outcome by 1 level.

c. Study did not adjust for confounding variables.

d. Ahmad 2018 did not provide confidence intervals, limiting the ability to make a judgement about imprecision, and the sample size is relatively small (n=392). Darapheak 2013 reported a wide confidence interval around the effect (OR=0.94, 95% CI: 0.76,1.15), however the sample size is quite large (n=1,907). We have downgraded the certainty of evidence for this outcome by 1 level.

e. Ahmad 2018 did not provide confidence intervals, limiting the ability to make a judgement about imprecision, and the sample size is relatively small (n=392). Darapheak 2013 reported a wide confidence interval around the effect (OR=0.94, 95% CI: 0.78,1.13), however the sample size is quite large (n=1,907). We have downgraded the certainty of evidence for this outcome by 1 level.

f. NIH tool for observational studies used. Overall, this study presents some concerns for bias. Of particular concern is the lack of confounding variables or adjustment of analyses to control for covariates.

g. Inconsistencies cannot be determined given the lack of data (n=1 study) contributing to this outcome, thus we have downgraded the certainty of evidence for this outcome by 1 level.

h. This study did not provide confidence intervals, limiting the ability to make a judgement about imprecision. It should also be noted that the sample size is large (n=1,956). We have downgraded the certainty of evidence for this outcome by 1 level.

i. NIH tool for observational studies used. Garden 2011 was rated as good quality given the study had a low risk of selection bias, information bias, and measurement bias. Braun 2016 was rated as fair quality due to a lack of description on the study population and a relatively high loss to follow up (>20%). In addition, the outcome data was repeatedly collected over 8 years, but the exposure data was collected only at one time point instead of repeated dietary data collection. Overall, between the two studies we have downgraded the quality of the evidence by 1.

j. Garden 2011 adjusted the model by sex, asthma study intervention group, birth weight, breastfeeding for at least 6 months, parental obesity status, ethnicity, smoking in pregnancy, father's education, and for total energy intake. Braun 2016 adjusted the model by child sex, ethnicity, age at dietary measurement, total energy intake at 1 y, birth weight z score, breastfeeding, playing sports, household income, maternal BMI at enrollment, education, folic acid use during pregnancy, smoking during pregnancy, diet score, and animal protein.

Table S2.7.2: GRADE table for fruit and anthropometric outcomes

Question: Is more frequent, varied, or amounts of consumption of fruit, compared to less, associated with beneficial anthropometric outcomes?

Certainty assessment							Impact	Certainty	Importance
№ of studies	Study design	Risk of bias	Inconsistency	Indirectness	Imprecision	Other considerations			
Wasting (Assessed with: body weight was measured using a portable Tanita digital scale and recumbent length using an infant length board. Wasting was defined as weight for length z-score less than 2 SD below the mean.)									
1	observational studies	not serious	serious[a]	not serious	serious[b]	none	Frequency of eating fruits was not significantly associated with wasting prevalence (p=0.356).[c]	⊕◯◯◯ Very low	CRITICAL
Underweight (Assessed with: body weight was measured using a portable Tanita digital scale and age was determined through interview with the infant's mother. Underweight was defined as weight for age z-score less than 2 SD below the mean).									
1	observational studies	not serious	serious[a]	not serious	serious[b]	none	Frequency of eating fruits was not significantly associated with underweight prevalence (p=0.995).[c]	⊕◯◯◯ Very low	CRITICAL
Stunting (Assessed with: recumbent length was measured using an infant length board and age was determined through interview with the infant's mother. Stunting was defined as height for age z-score less than 2 SD below the mean).									
1	observational studies	not serious	serious[a]	not serious	serious[b]	none	Frequency of eating fruits was not significantly associated with stunting prevalence (p=0.623).[c]	⊕◯◯◯ Very low	CRITICAL
Overweight (Follow-up: 12 months; Assessed with: WHO 2006 Child Growth standards for Children 0-5 years old by calculating BMI percentiles. BMI ≥85 percentile of age was defined as overweight. Overweight prevalence was assessed at 18 months of age).									
1	observational studies	serious[d]	serious[a]	not serious	serious[e]	none	Frequency of eating fruit was significantly associated with overweight prevalence after 12 months of follow-up. 6.36% (n=22) and 18.15% (n=285) of those who consumed fruit <7 times/week and ≥ 7 times/week were overweight, respectively (p<0.001 between groups).[c]	⊕◯◯◯ Very low	CRITICAL
Body Mass Index (Follow-up: 78 months; Assessed with: height was measured using a portable stadiometer and weight was measured using electronic bathroom scales at 8 years of age. BMI was calculated in kilograms per meters squared).									
1	observational studies	not serious	serious[a]	not serious	serious[f]	none	Higher intake of fruit at 18 months was not significantly associated with BMI at 8 years old (p=0.11).[g]	⊕◯◯◯ Very low	IMPORTANT

Explanations

a. Inconsistencies cannot be determined given the lack of data (n=1 study) contributing to this outcome, thus we have downgraded the certainty of evidence for this outcome by 1 level.

b. This study did not provide confidence intervals, limiting the ability to make a judgement about imprecision. It should also be noted that the sample size is relatively small (n=392). We have downgraded the certainty of evidence for this outcome by 1 level.
c. Study did not adjust for confounding variables.
d. NIH tool for observational studies used. Overall, this study presents some concerns for bias. Of particular concern is the lack of confounding variables or adjustment of analyses to control for covariates.
e. This study did not provide confidence intervals, limiting the ability to make a judgement about imprecision. It should also be noted that the sample size is large (n=1,956). We have downgraded the certainty of evidence for this outcome by 1 level.
f. The confidence interval around the effect is wide (Reg coefficient=0.17, 95% CI: -0.04,0.38) and the sample size is relatively small (n=362) thus we have downgraded the certainty of evidence for this outcome by 1 level.
g. The model was adjusted by sex, asthma study intervention group, birth weight, breastfeeding for at least 6 months, parental obesity status, ethnicity, smoking in pregnancy, father's education, and for total energy intake.

Table S2.7.3: GRADE table for FV and anthropometric outcomes

Question: Is more frequent, varied, or amounts of consumption of FV, compared to less, associated with beneficial anthropometric outcomes?

Certainty assessment							Impact	Certainty	Importance
№ of studies	Study design	Risk of bias	Inconsistency	Indirectness	Imprecision	Other considerations			
Stunting (Assessed with: the China Food and Nutrition Surveillance System (CFNSS). The CFNSS uses the Z-score as recommended by WHO to evaluate the growth and development of infants and young children).									
1	observational studies	very serious[a]	serious[b]	not serious	serious[c]	none	Frequency of eating vegetables and fruits was significantly associated with stunting prevalence. For those who consumed vegetables and fruits weekly, monthly, and < once per month or none, compared to daily, the odds of being stunted were 1.739 (p=0.00), 1.698 (p=0.03), and 1.768 (p=0.00) times greater, respectively.[d]	⊕◯◯◯ Very low	CRITICAL
Underweight (Assessed with: the CFNSS which uses the Z-score as recommended by WHO to evaluate the growth and development of infants and young children).									
1	observational studies	very serious[a]	serious[b]	not serious	serious[c]	none	Frequency of eating vegetables and fruits was significantly associated with underweight prevalence. For those who consumed vegetables and fruits weekly, monthly, and < once per month or none, compared to daily, the odds of being underweight were 1.908 (p=0.00), 1.566 (p=0.10), and 1.478 (p=0.01) times greater, respectively.[d]	⊕◯◯◯ Very low	CRITICAL

Explanations

a. NIH tool for observational studies used. This study lacks internal validity. Firstly, the paper does not report a methods section. Thus, no detail is provided on the tools used to measure the exposure or outcome. The sample size is not clearly stated, and thus had to be inferred from tables. In the analysis, the reference arm is not reported within the tables, once again requiring the reader to infer this information from the text. Results should be interpreted with extreme caution.
b. Inconsistencies cannot be determined given the lack of data (n=1 study) contributing to this outcome, thus we have downgraded the certainty of evidence for this outcome by 1 level.

c. This study did not provide confidence intervals, limiting the ability to make a judgement about imprecision. It should be noted that the sample size is very large (n=13,107), however given the lack of confidence intervals provided we have downgraded the certainty of evidence for this outcome by 1 level.
d. Study did not adjust for confounding variables.

Table S2.7.4: GRADE table for NPS and anthropometric outcomes

Question: Is more frequent, varied, or amounts of consumption of NPS, compared to less, associated with beneficial anthropometric outcomes?

Certainty assessment							Impact	Certainty	Importance
№ of studies	Study design	Risk of bias	Inconsistency	Indirectness	Imprecision	Other considerations			
**Wasting** (**Assessed with**: Ahmad 2018 measured body weight using a portable Tanita digital scale and recumbent length using an infant length board. Darapheak 2013 measured weight using a lightweight electronic SECA scale and height using a board made by Shorr Productions. For both studies, wasting was defined as weight for length z-score less than 2 SD below the mean.)									
2	observational studies	serious[a]	not serious	not serious	serious[b]	none	In both studies, frequency of eating legumes, pulses, or seeds was not significantly associated with wasting prevalence (p>0.05).[c]	⨁◯◯◯ Very low	CRITICAL
**Underweight** (**Assessed with**: Ahmad 2018 measured body weight using a portable Tanita digital scale and age was determined through interview with the infant's mother. Darapheak 2013 measured weight using a lightweight electronic SECA scale and age was determined through caregiver interview. For both studies, underweight was defined as weight for age z-score less than 2 SD below the mean.)									
2	observational studies	serious[a]	not serious	not serious	serious[d]	none	In both studies, frequency of eating legumes, pulses, or seeds was not significantly associated with underweight prevalence (p>0.05).[c]	⨁◯◯◯ Very low	CRITICAL
**Stunting** (**Assessed with**: Ahmad 2018 measured recumbent length using an infant length board and age was determined through interview with the infant's mother. Darapheak 2013 measured height using a board made by Shorr Productions and age was determined through caregiver interview. For both studies, stunting was defined as height for age z-score less than 2 SD below the mean.)									
2	observational studies	serious[a]	not serious	not serious	serious[e]	none	In both studies, frequency of eating legumes, pulses, and seeds was not significantly associated with stunting prevalence (p>0.05).[c]	⨁◯◯◯ Very low	CRITICAL

Explanations

a. NIH tool for observational studies used. Ahmad 2018 was rated as good quality given the study had a low risk of selection bias, information bias, and measurement bias. Darapheak 2013 was rated as fair quality due to a lack of description provided for children who were included in the analysis.
b. Ahmad 2018 did not provide confidence intervals, limiting the ability to make a judgement about imprecision, and the sample size is relatively small (n=392). Darapheak 2013 reported a wide confidence interval around the effect for pulse consumption (OR=1.74, 95% CI: 0.96,3.15) and for seed consumption (OR=0.83, 95% CI: 0.39,1.75). We have downgraded the certainty of evidence for this outcome by 1 level.
c. Ahmad 2018 did not adjust for confounding variables. Darapheak 2013 adjusted the model by household wealth quintile, education of mother, geographical area, and residential location.
d. Ahmad 2018 did not provide confidence intervals, limiting the ability to make a judgement about imprecision, and the sample size is relatively small (n=392). Darapheak 2013 reported a wide confidence interval around the effect for pulse consumption (OR=0.88, 95% CI: 0.59,1.33) and for seed consumption (OR=0.92, 95% CI: 0.61,1.39). We have downgraded the certainty of evidence for this outcome by 1 level.

e. Ahmad 2018 did not provide confidence intervals, limiting the ability to make a judgement about imprecision, and the sample size is relatively small (n=392). Darapheak 2013 reported a wide confidence interval around the effect for pulse consumption (OR=0.84, 95% CI: 0.58,1.22) and for seed consumption (OR=1.15, 95% CI: 0.78,1.67). We have downgraded the certainty of evidence for this outcome by 1 level.

Table S2.7.5: GRADE table for vegetables and anthropometric indices

Question: Is more frequent, varied, or amounts of consumption of vegetables, compared to less, associated with beneficial anthropometric indices?

Certainty assessment							Impact	Certainty	Importance
№ of studies	Study design	Risk of bias	Inconsistency	Indirectness	Imprecision	Other considerations			
BMI Z-score (Follow-up: 29 months; Assessed with: World Health Organization Anthro-based weight and height measurements at age 3.7 years. Measurements were collected by trained study staff using a standardized protocol in which children were measured without footwear or outer clothes using standardized equipment).									
1	observational studies	not serious	serious[a]	not serious	serious[b]	none	Consumption of vegetables at age 14 months was not significantly associated with BMI z-score at age 3.7 years (p=0.12).[c]	⊕◯◯◯ Very low	CRITICAL
Height-for-age Z-score (Follow-up: 7 months; Assessed with: measurement of recumbent length to the nearest mm by trained staff using a measuring board. Recumbent length was measured in September 2002, and then again 7 months later during the survey administration. Age was determined using the IPTc trial database of the study area).									
1	observational studies	serious[d]	serious[a]	not serious	serious[e]	none	There was a borderline difference from less frequent vegetable/leaves consumption with HAZ, where those who consumed vegetables/leaves 0-2 days/week and ≥ 3 days/week had a mean HAZ of -1.01 (p=0.052) and -0.59 (p<0.06), respectively, pointing to a trend of lower HAZ among children who consume vegetables less frequently.[f]	⊕◯◯◯ Very low	CRITICAL
Waist circumference (Follow-up: 78 months; Assessed with: measured with a flexible steel tape at the level of the narrowest point (or midpoint) between the lower costal border and the iliac crest, to the nearest 0.1 cm at 8 years of age).									
1	observational studies	not serious	serious[a]	not serious	serious[g]	none	Higher intake of vegetables at 18 months was not significantly associated with waist circumference at 8 years old (p=0.07).[c]	⊕◯◯◯ Very low	NOT IMPORTANT
Linear growth (Follow up: 7 months; Assessed with: measurement of recumbent length to the nearest mm by trained staff using a measuring board in September 2002. Followed by a second measurement 7 months later during the survey administration, this time measured as standing height. Height increments were computed as the difference between measurements taken during the survey and in September 2002, divided by the precise duration and multiplied by 7 months (i.e., the average duration of the interval under study). No adjustment was made for the change in measuring technique (I.e., standing height instead of recumbent length).									

Certainty assessment							Impact	Certainty	Importance
№ of studies	Study design	Risk of bias	Inconsistency	Indirectness	Imprecision	Other considerations			
1	observational studies	serious[d]	serious[a]	not serious	serious[e]	none	Frequent consumption of vegetables had a statistically significant inverse relationship to linear growth (means: 8.3cm and 7.4cm height increment over the preceding 7 months for rare and frequent consumption, respectively, p=0.041).[h]	⨁◯◯◯ Very low	CRITICAL
Height (Follow-up: 96 months; Assessed with: height was measured using a Harpenden stadiometer (Holtain Limited) at 8 different time points between the ages of 13 months and 9 years of age).									
1	observational studies	serious[i]	serious[a]	not serious	not serious	none	Higher intake of vegetable protein in early childhood (1 year old) was not significantly associated with height measures in later childhood (between the ages of 1 and 9 years old) (p>0.05).[j]	⨁◯◯◯ Very low	CRITICAL
Weight (Follow-up: 96 months; Assessed with: weight was measured using a mechanical personal scale (SECA) at 8 different time points between the ages of 13 months and 9 years of age).									
1	observational studies	serious[i]	serious[a]	not serious	not serious	none	Higher intake of vegetable protein in early childhood (1 year old) was not significantly associated with weight measures in later childhood (between the ages of 1 and 9 years old) (p>0.05).[j]	⨁◯◯◯ Very low	CRITICAL

Explanations

a. Inconsistencies cannot be determined given the lack of data (n=1 study) contributing to this outcome, thus we have downgraded the certainty of evidence for this outcome by 1 level.
b. This study did not provide confidence intervals, limiting the ability to make a judgement about imprecision. It should also be noted that the sample size is relatively small (n=337). We have downgraded the certainty of evidence for this outcome by 1 level.
c. Study did not adjust for confounding variables.
d. NIH tool for observational studies used. This study was rated as having fair quality due to some concerns of bias. Firstly, it should be noted that data on food consumption was attained from April to May 2003, while height increment was measured in 2002 and 2003. As food consumed in 2003 does not necessarily mean the child had consumed the same food back in 2002, this may lead to inaccuracies in the association between frequency of food consumption and height increment. Secondly, 7 months may not be long enough to cause a significant change in height. Caution should be taken when interpreting results from this study.
e. This study did not provide confidence intervals, limiting the ability to make a judgement about imprecision. It should also be noted that the sample size is relatively small (n=165). We have downgraded the certainty of evidence for this outcome by 1 level.
f. Model adjusted by child age.
g. The confidence interval around the effect is wide (Reg coefficient=0.5, 95% CI: -0.04,1.04) and the sample size is relatively small (n=362) thus we have downgraded the certainty of evidence for this outcome by 1 level.
h. Model adjusted by child age, sex, malaria study intervention group, maternal height, BMI, schooling, and number of children 5 y old.
i. NIH tool for observational studies used. This study was rated as having fair quality. Some concerns include the lack of detail provided on the study population, the lack of repeated dietary data which was only conducted at one time point compared to the outcome data which was repeatedly collected over 8 years, and there was a relatively high loss to follow up (>20%). It should be noted that some of the authors have been funded by Nestle and this may cause bias.

j. Model adjusted by child sex, ethnicity, age at dietary measurement, total energy intake at 1 y, birth weight z score, breastfeeding, playing sports, household income, maternal BMI at enrollment, education, folic acid use during pregnancy, smoking during pregnancy, diet score, and animal protein.

Table S2.7.6: GRADE table for fruit and anthropometric indices

Question: Is more frequent, varied, or amounts of consumption of fruit, compared to less, associated with beneficial anthropometric indices?

Certainty assessment							Impact	Certainty	Importance
№ of studies	Study design	Risk of bias	Inconsistency	Indirectness	Imprecision	Other considerations			
BMI Z-score (Follow-up: Mallan et al. had 29 months of follow-up, but Lundkvist et al. assessed the exposure and outcome at 18 months of age; Assessed with: For Mallan et al., the World Health Organization Anthro-based weight and height measurements were assessed at age 3.7 years. Measurements were collected by trained study staff using a standardized protocol in which children were measured without footwear or outer clothes using standardized equipment. For Lundkvist et al., the WHO Anthro Survey Analyser tool was used, and measurements for weight and length were retrieved from child health records at 18 months of age).									
2	observational studies	serious[a]	serious[b]	not serious	serious[c]	none	In Mallan et al. consumption of fruits at age 14 months was not significantly associated with BMI z-score at age 3.7 years (p=0.78). In Lundkvist et al. fruit juice consumption at 18 months of age was negatively associated with BMI-z score at 18 months of age. The mean BMIz was 0.72 ± 0.92 for non-consumers, 0.61 ± 0.91 for seldom consumers and 0.45 ± 0.98 for regular consumers (p=0.003 between groups). [d]	⨁◯◯◯ Very low	CRITICAL
Height-for-age Z-score (Follow up: 7 months; Assessed with: measurement of recumbent length to the nearest mm by trained staff using a measuring board. Recumbent length was measured in September 2002, and then again 7 months later during the survey administration. Age was determined using the IPTc trial database of the study area).									
1	observational studies	serious[e]	serious[f]	not serious	serious[g]	none	There was a borderline difference from less frequent fruit consumption with HAZ, where those who consumed fruit 0-2 days/week and ≥ 3 days/week had a mean HAZ of -1.04 (p=0.051) and -0.71 (p=0.059), respectively, pointing to a trend of lower HAZ among children who consume fruits less frequently.[h]	⨁◯◯◯ Very low	CRITICAL

Waist circumference (Follow-up: 78 months; Assessed with: measured with a flexible steel tape at the level of the narrowest point (or midpoint) between the lower costal border and the iliac crest, to the nearest 0.1 cm at 8 years of age).

Certainty assessment							Impact	Certainty	Importance
№ of studies	Study design	Risk of bias	Inconsistency	Indirectness	Imprecision	Other considerations			
1	observational studies	not serious	serious[f]	not serious	serious[i]	none	Higher intake of fruit at 18 months was significantly associated with waist circumference at 8 years old. In the adjusted model, children who consumed increasing quintiles of fruit (0g, 17g, 45g, 80g, 124g) were significantly more likely to have a larger waist circumference by 0.63cm (Reg coefficient=0.63, 95% CI: 0.05-1.20, p=0.03).[j]	⨁◯◯◯ Very low	NOT IMPORTANT
Linear growth (Follow up: 7 months; Assessed with: measurement of recumbent length to the nearest mm by trained staff using a measuring board in September 2002. Followed by a second measurement 7 months later during the survey administration, this time measured as standing height. Height increments were computed as the difference between measurements taken during the survey and in September 2002, divided by the precise duration and multiplied by 7 months (I.e., the average duration of the interval under study). No adjustment was made for the change in measuring technique (I.e., standing height instead of recumbent length).									
1	observational studies	serious[e]	serious[f]	not serious	serious[g]	none	Fruit consumption was positively associated with linear growth in fully adjusted models (means: 7.9cm and 8.7cm height increment over the preceding 7 months for rare and frequent consumption, respectively, p=0.027).[k]	⨁◯◯◯ Very low	CRITICAL

Explanations

a. The NIH tool for observational studies was used. The study by Mallan et al. was rated as good quality due to a combination of data from the RCT (control and intervention groups) with this longitudinal cohort study which allowed for a satisfactory sample size and reduced type II error. The study by Lundkvist et al was rated as having fair quality due to some concerns of bias. Firstly, although the baseline characteristics of the participating mothers were reported many baseline characteristics of the children were not reported. Secondly, the number of participants recruited at baseline compared to the participants enrolled were not mentioned with their corresponding data.
b. The outcomes reported were not the same, with Mallan et al. reporting no significant association between BMI score and fruit consumption and Lundkvist et al. reporting a negative association with BMI score and fruit juice consumption, thus we have downgraded the certainty of evidence for this outcome by 1 level.
c. The Mallan et al. study or the Lundkvist et al. study did not provide confidence intervals, limiting the ability to make a judgement about imprecision. It should also be noted that the sample size is relatively small (n=337) for the Mallan et al. study. But relatively large for the Lundkvist et al. study (n=1499). We have downgraded the certainty of evidence for this outcome by 1 level.
d. The Mallan et al. study and the Lundkvist et al. study did not adjust for confounding variables.
e. NIH tool for observational studies used. This study was rated as having fair quality due to some concerns of bias. Firstly, it should be noted that data on food consumption was attained from April to May 2003, while height increment was measured in 2002 and 2003. As food consumed in 2003 does not necessarily mean the child had consumed the same food back in 2002, this may lead to inaccuracies in the association between frequency of food consumption and height increment. Secondly, 7 months may not be long enough to cause a significant change in height. Caution should be taken when interpreting results from this study.
f. Inconsistencies cannot be determined given the lack of data (n=1 study) contributing to this outcome, thus we have downgraded the certainty of evidence for this outcome by 1 level.
g. This study did not provide confidence intervals, limiting the ability to make a judgement about imprecision. It should also be noted that the sample size is relatively small (n=165). We have downgraded the certainty of evidence for this outcome by 1 level.
h. Model was adjusted by child age.
i. The confidence interval around the effect is wide (Reg coefficient=0.63, 95% CI: 0.05,1.20) and the sample size is relatively small (n=362) thus we have downgraded the certainty of evidence for this outcome by 1 level.
j. Model was adjusted by total energy intake.
k. Model was adjusted by child age, sex, malaria study intervention group, maternal height, BMI, schooling, and number of children 5 y old.

Table S2.7.7: GRADE table for NPS and anthropometric indices

Question: Is more frequent, varied, or amounts of consumption of NPS, compared to less, associated with beneficial anthropometric indices?

Certainty assessment							Impact	Certainty	Importance
№ of studies	Study design	Risk of bias	Inconsistency	Indirectness	Imprecision	Other considerations			
**Length** (**Follow-up**: 12 months; **Assessed with**: measured using a measuring board at 6, 9, 12, 15, and 18 months of age).									
1	randomised trials	serious[a]	serious[b]	not serious	serious[c]	none	In both boys and girls, amount of maize/cowpea consumption was significantly associated with mean length after 3,6,9, and 12 months of follow-up.<br><br>Boys who consumed maize/cowpea, and those who had no consumption, had mean length 70.65cm and 67.52cm at 3 months of follow-up, 74.69cm and 71.78cm at 6 months of follow-up, 76.05cm and 72.47cm at 9 months of follow-up, and 79.23cm and 74.21cm at 12 months of follow-up, respectively (p<0.05 between groups). Girls who consumed maize/cowpea, and those who had no consumption, had mean length 67.51cm and 65.43cm at 3 months follow-up, 71.93cm and 67.72cm at 6 months follow-up, 76.42cm and 69.24cm at 9 months follow-up, and 78.15cm and 73.23cm at 12 months follow-up, respectively (p<0.05 between groups).[d]	⨁◯◯◯ Very low	CRITICAL
**Weight** (**Follow-up**: 12 months; **Assessed with**: measured using a spring balance scale at 6, 9, 12, 15, and 18 months of age).									
1	randomised trials	serious[a]	serious[b]	not serious	serious[c]	none	In both boys and girls, amount of maize/cowpea consumption was significantly associated with mean weight after 3, 6, 9, and 12 months of follow-up.<br><br>Boys who consumed maize/cowpea, and those who had no consumption, had mean weight 7.78kg and 5.56kg at 3 months of follow-up, 9.82kg and 5.05kg at 6 months of follow-up, 10.12kg and 6.08kg at 9 months of follow-up, and 10.51kg and 6.6kg at 12 months of follow-up, respectively (p<0.05 between groups). Girls who consumed maize/cowpea, and those who had no consumption, had mean weight 8.2kg and 6.15kg at 3 months follow-up, 9.19kg and 6.41kg at 6 months follow-up, 10.32kg and 6.53kg at 9 months follow-up, and 9.62kg and 7.07kg at 12 months follow-up, respectively (p<0.05 between groups).[d]	⨁◯◯◯ Very low	CRITICAL

Explanations

a. RoB-2 tool for randomized trials used. This study was rated as having some concerns for bias. In particular, the methods are unclear as to allocation concealment and blinding for participants and assessors which could lend to possible selection bias.
b. Inconsistencies cannot be determined given the lack of data (n=1 study) contributing to this outcome, thus we have downgraded the certainty of evidence for this outcome by 1 level.
c. This study did not provide confidence intervals, limiting the ability to make a judgement about imprecision. It should also be noted that the sample size is very small (n=90). We have downgraded the certainty of evidence for this outcome by 1 level.
d. Study did not adjust for confounding variables.

Table S2.7.8: GRADE table for vegetables and nutrient status

Question: Is more frequent, varied, or amounts of consumption of vegetables, compared to less, associated with beneficial nutrient status at study endline?

Certainty assessment							Impact	Certainty	Importance
№ of studies	Study design	Risk of bias	Inconsistency	Indirectness	Imprecision	Other considerations			
Iron stores (ferritin <20μg/L) (Assessed with: venous blood samples that were obtained before 12am and haematological tests were carried out within 6 hours using Technicon H*1. Serum ferritin was analyzed using an immunological method (Orion Hitachi 704)).									
1	observational studies	very serious[a]	serious[b]	not serious	serious[c]	none	Those who consumed vegetables once/day, compared to <once/day, were significantly more likely to have low iron stores (ferritin values <20μg/L) (Reg coefficient= -2.7, p=0.02). Although the results suggest a negative effect of feeding vegetables once/day, findings were inconsistent as this relationship was not seen for those eating vegetables more frequently (several times/day) compared to <once/day. Additionally, there was no significant relationship observed between the feeding frequency of vegetables and the likelihood of having very low iron stores (ferritin <15μg/L).[d]	⊕◯◯◯ Very low	CRITICAL
Hemoglobin levels (Assessed with: a heel prick sample of capillary blood was taken from the children at 18 months old and collected into an EDTA capillary tube. The haemoglobin concentration was assayed using the HEMOCUE B-Hb photometer. The blood was then centrifuged, and the plasma removed and frozen. Plasma ferritin was assayed using the DELFIA time resolved uoroimmunoassay system).									

Certainty assessment							Impact	Certainty	Importance
№ of studies	Study design	Risk of bias	Inconsistency	Indirectness	Imprecision	Other considerations			
1	observational studies	serious[e]	serious[b]	not serious	serious[f]	none	In children aged 18 months, mean hemoglobin levels were significantly higher in children who consumed any vegetables when compared to those who had no vegetable consumption (p=0.026). For boys, those who consumed any vegetables, and no vegetables, had a mean hemoglobin level of 11.7g/l (SD=1.0) and 11.3g/l (SD=0.9), respectively. For girls, those who consumed any vegetables, and no vegetables, had a mean hemoglobin level of 11.8g/l (SD=0.9) and 11.6g/l (SD=0.8), respectively.[g]	⊕◯◯◯ Very low	CRITICAL
Anemia (Assessed with: blood samples obtained through heel or ring finger puncture. Capillary blood was collected in a microcube and the hemoglobin measurement was obtained by direct reading on a portable hemoglobinometer (Hemocue ®). A hemoglobin concentration of 11g/dL was adopted as the cut-off point for the diagnosis of anemia).									
1	observational studies	very serious[h]	serious[b]	not serious	serious[i]	none	Frequency of eating dark green vegetables was not significantly associated with anemia prevalence (p=0.502).[d]	⊕◯◯◯ Very low	CRITICAL
Branched-chain amino acid levels (Follow-up: Exposure and outcome both measured at 12 months of age; Assessed with: Non-fasting venous blood was collected from infants aged 12 M into EDTA tubes and centrifuged at 4 ◦C to prepare the plasma on the same day. Plasma samples were aliquoted into 1 mL and stored in a −20 ◦C freezer until analysis. Samples from fifty-four infants were selected for plasma amino acids analysis by ion exchange chromatography).									
1	observational studies	very serious[j]	serious[b]	not serious	serious[k]	none	There was no association between plant-based protein intake at 12 months with plasma BCAA levels at 12 months (p>0.05).[l]	⊕◯◯◯ Very low	CRITICAL
Essential amino acid levels (Follow-up: Exposure and outcome both measured at 12 months of age; Assessed with: Non-fasting venous blood was collected from infants aged 12 M into EDTA tubes and centrifuged at 4 ◦C to prepare the plasma on the same day. Plasma samples were aliquoted into 1 mL and stored in a −20 ◦C freezer until analysis. Samples from fifty-four infants were selected for plasma amino acids analysis by ion exchange chromatography).									
1	observational studies	very serious[j]	serious[b]	not serious	serious[k]	none	There was no association between plant-based protein intake at 12 months with plasma EAA levels at 12 months (p>0.05).[l]	⊕◯◯◯ Very low	CRITICAL

Certainty assessment							Impact	Certainty	Importance
№ of studies	Study design	Risk of bias	Inconsistency	Indirectness	Imprecision	Other considerations			
Non-essential amino acid levels (Follow-up: Exposure and outcome both measured at 12 months of age; Assessed with: Non-fasting venous blood was collected from infants aged 12 M into EDTA tubes and centrifuged at 4 ◦C to prepare the plasma on the same day. Plasma samples were aliquoted into 1 mL and stored in a −20 ◦C freezer until analysis. Samples from fifty-four infants were selected for plasma amino acids analysis by ion exchange chromatography).									
1	observational studies	very serious[j]	serious[b]	not serious	serious[k]	none	There was no association between plant-based protein intake at 12 months with plasma NEAA levels at 12 months (p>0.05).[l]	⊕◯◯◯ Very low	CRITICAL
Total amino acid levels (Follow-up: Exposure and outcome both measured at 12 months of age; Assessed with: Non-fasting venous blood was collected from infants aged 12 M into EDTA tubes and centrifuged at 4 ◦C to prepare the plasma on the same day. Plasma samples were aliquoted into 1 mL and stored in a −20 ◦C freezer until analysis. Samples from fifty-four infants were selected for plasma amino acids analysis by ion exchange chromatography).									
1	observational studies	very serious[j]	serious[b]	not serious	serious[k]	none	There was no association between plant-based protein intake at 12 months with total amino acid levels at 12 months (p>0.05).[l]	⊕◯◯◯ Very low	CRITICAL

Explanations

a. NIH tool for observational studies used. This study reported relatively little methodology and what methods are reported are unclear at times, in addition to a very small sample size. Because of this, it's extremely challenging to evaluate whether there was bias introduced or not and ultimately whether the study has internal validity.
b. Inconsistencies cannot be determined given the lack of data (n=1 study) contributing to this outcome, thus we have downgraded the certainty of evidence for this outcome by 1 level.
c. This study did not provide confidence intervals, limiting the ability to make a judgement about imprecision. It should also be noted that the sample size is very small (n=74), however the certainty of the evidence has already been rated as very low based on the very poor-quality risk of bias rating and thus could not be further downgraded.
d. Study did not adjust for confounding variables.
e. NIH tool for observational studies used. This study was rated as having fair quality. This study was downgraded as the statistical models were only adjusted by sex and no further confounding variables were considered.
f. This study did not provide confidence intervals, limiting the ability to make a judgement about imprecision. The sample size is moderate (n=796). We have downgraded the certainty of evidence for this outcome by 1 level.
g. Model was adjusted by sex.
h. NIH tool for observational studies used. Overall, this study has a poor to fair rating. Firstly, there is very limited detail provided on the methodology which makes it challenging to evaluate if there is internal validity. Some considerations to note include lack of sample size justification and no details are provided on participation rate of eligible participants. Furthermore, there is a small sample size with no details provided for baseline characteristics of study sample.
i. The confidence interval around the effect is very wide (OR=1.21; 95%CI: 0.67-2.21) and the sample size is small (n=205) thus we have downgraded the certainty of evidence for this outcome by 1 level.
j. NIH tool for observational studies used. This study was rated as having poor quality. There was no reference group used to compare the outcomes, and no details are provided on participation rate of eligible participants.
k. This study did not provide confidence intervals, limiting the ability to make a judgement about imprecision. The sample size is small (n=54). We have downgraded the certainty of evidence for this outcome by 1 level.
l. Adjusted for gender, type of milk feeding, and energy intake.

Table S2.7.9: GRADE table for fruit and nutrient status

Question: Is more frequent, varied, or amounts of consumption of fruit, compared to less, associated with beneficial nutrient status at study endline?

Certainty assessment							Impact	Certainty	Importance
№ of studies	Study design	Risk of bias	Inconsistency	Indirectness	Imprecision	Other considerations			
Hemoglobin levels (Assessed with: Cowin 2001 measured hemoglobin levels using a heel prick sample of capillary blood taken from children at 18 months old and collected into an EDTA capillary tube. The haemoglobin concentration was assayed using the HEMOCUE B-Hb photometer. The blood was then centrifuged, and the plasma removed and frozen. Plasma ferritin was assayed using the DELFIA time resolved uoroimmunoassay system. Oliveira 2004 measured hemoglobin levels using the cyanmethaemoglobin method, using the HemoCue system (WHO,19 2001). In case hemoglobin levels were below 9 g/dl of blood, a second dosage was performed and the mean of the two measurements was adopted as the final value. Blood collection was done by fingertip lancing, using disposable lancets).									
2	observational studies	very serious[a]	serious[b]	not serious	serious[c]	none	In both studies, fruit consumption was significantly associated with hemoglobin levels. However, Cowin 2001 reports a positive association, where in both boy and girls aged 18 months, mean hemoglobin levels were significantly higher for those who consumed any fruit when compared to those who consumed no fruit (p=0.028). For boys, those who consumed any citrus fruit, and no citrus fruit, had a mean hemoglobin level of 11.8g/l (SD=1.1) and 11.6g/l (SD=0.9), respectively. For girls, those who consumed any citrus fruit, and no citrus fruit, had a mean hemoglobin level of 11.9g/l (SD=1.0) and 11.7g/l (SD=0.9), respectively.<br>For boys, those who consumed any fruit, and no fruit, had a mean hemoglobin level of 11.7g/l (SD=0.9) and 11.5g/l (SD=1.2), respectively. For girls, those who consumed any fruit, and no fruit, had a mean hemoglobin level of 11.8g/l (SD=0.9) and 11.5g/l (SD=0.8), respectively.[d] Conversely, Oliveira 2004 reports a negative association, where children who consumed fruit, compared to no consumption, were significantly more likely to have lower hemoglobin levels by 2g/dl (Reg coefficient= -2.00, SE= 0.56, p<0.001).[e]	⨁◯◯◯ Very low	CRITICAL
Anemia (Assessed with: blood samples obtained through heel or ring finger puncture. Capillary blood was collected in a microcube and the hemoglobin measurement was obtained by direct reading on a portable hemoglobinometer (Hemocue ®). A hemoglobin concentration of 11g/dL was adopted as the cut-off point for the diagnosis of anemia).									

Certainty assessment							Impact	Certainty	Importance
№ of studies	Study design	Risk of bias	Inconsistency	Indirectness	Imprecision	Other considerations			
1	observational studies	very serious[f]	serious[g]	not serious	serious[h]	none	Fruit consumption was not significantly associated with anemia prevalence when considering consumption in the past 24 hours. However, when looking at daily vs < than daily frequencies, in the adjusted model, fruit consumption was significantly associated with anemia prevalence. For those who consumed fruit < daily, compared to daily, the odds were 1.88 times greater (95% CI: 1.03-3.42, p=0.003) of having anemia.[i]	⊕◯◯◯ Very low	CRITICAL
Vitamin C intake (Assessed with: The prevalence of inadequate nutrient intakes was examined using the Estimated Average Requirement (EAR) cut-point method (Institute of Medicine,2000)).									
1	observational studies	serious[j]	serious[g]	not serious	serious[k]	none	Mean daily vitamin C intake was significantly higher with increased frequency of 100% fruit juice consumption in boys and girls aged 1-3 years. Means of 70mg (SE=6.4), 124mg (SE=6), and 180mg (SE=6.2) for consuming fruit juice <0.5times/day, 0.5 to <1.5 times/day, and 1.5 times/day, respectively (p<0.05 between groups).[l]	⊕◯◯◯ Very low	CRITICAL
Magnesium Intake (Assessed with: The prevalence of inadequate nutrient intakes was examined using the Estimated Average Requirement (EAR) cut-point method (Institute of Medicine,2000)).									
1	observational studies	serious[j]	serious[g]	not serious	serious[k]	none	More frequent consumption of 100% fruit juice was associated with higher intake of magnesium and lower prevalence of inadequate magnesium intake among the most versus least frequent consumers of fruit juice in children aged 1-3 years. Means of 198mg (SE=7.3), 213mg (SE=5.2), and 221mg (SE=5.5) for consuming fruit juice <0.5times/day, 0.5 to <1.5 times/day, and 1.5 times/day, respectively (p<0.05 between groups).[l]	⊕◯◯◯ Very low	CRITICAL

Explanations

a. NIH tool for observational studies used. Cowin 2001 was rated as having fair quality because the statistical models were only adjusted by sex and no further confounding variables were considered. Oliveira 2004 was rated as having poor quality because details on methodology are lacking, which makes it challenging to determine if there is internal validity. Not

much detail is provided on eligibility criteria or a description of participants. The exposure for food consumption is not well defined, all we know is that caregivers were asked about their child's food consumption at time of the interview but there are no other details provided.
b. Both studies found a statistically significant association between fruit consumption and hemoglobin levels. However, Cowin 2001 reports a positive association and Oliveira 2004 reports a negative association. Given these inconsistencies, we have downgraded the certainty of evidence by 1 level for this outcome.
c. Both studies did not provide confidence intervals, limiting the ability to make a judgement about imprecision. It should also be noted that the sample size is small for Oliveira 2004 (n=179) and moderate for Cowin 2001 (n=796). We have downgraded the certainty of evidence for this outcome by 1 level.
d. The models for any fruit consumption were adjusted by sex.
e. Model adjusted by duration of gestation, mother's schooling, child's sex, anthropometric status based on the height-for-age indicator, and age (squared).
f. NIH tool for observational studies used. Overall, this study has a poor to fair rating. Firstly, there is very limited detail provided on the methodology which makes it challenging to evaluate if there is internal validity. Some considerations to note include lack of sample size justification and no details are provided on participation rate of eligible participants. Furthermore, there is a small sample size with no details provided for baseline characteristics of study sample.
g. Inconsistencies cannot be determined given the lack of data (n=1 study) contributing to this outcome, thus we have downgraded the certainty of evidence for this outcome by 1 level.
h. The confidence interval around the effect is very wide (OR=1.88; 95%CI: 1.03-3.42) and the sample size is small (n=205) thus we have downgraded the certainty of evidence for this outcome by 1 level.
i. Model adjusted by family income per capita and consumption of medicated iron supplements.
j. NIH tool for observational studies used. Overall, this study was rated as fair quality given there was a large sample size, however, they only looked at the association at one time point with no data shown for more recent years and models were not adjusted for confounding variables.
k. The sample size is large (n= 2193) however, this study did not provide confidence intervals, limiting the ability to make a judgement about imprecision. We have downgraded the certainty of evidence for this outcome by 1 level.
l.Ratio adjustment for intake day, categorized as weekend/weekday, and energy intake.

Table S2.7.10: GRADE table for NPS and nutrient status

Question: Is more frequent, varied, or amounts of consumption of NPS, compared to less, associated with beneficial nutrient status at study endline?

Certainty assessment							Impact	Certainty	Importance
№ of studies	Study design	Risk of bias	Inconsistency	Indirectness	Imprecision	Other considerations			
**Hemoglobin levels** (**Assessed with**: the cyanmethaemoglobin method, using the HemoCue system (WHO,19 2001). In case hemoglobin levels were below 9 g/dl of blood, a second dosage was performed and the mean of the two measurements was adopted as the final value. Blood collection was done by fingertip lancing, using disposable lancets).									
1	observational studies	very serious[a]	serious[b]	not serious	serious[c]	none	Bean consumption was positively associated with hemoglobin levels in the adjusted model. Children who consumed beans, compared to no consumption, were significantly more likely to have higher hemoglobin levels by 0.56g/dl (Reg coefficient= 0.56, SE= 0.23, p=0.018).[d]	⊕◯◯◯ Very low	CRITICAL
**Anemia** (**Assessed with**: blood samples obtained through heel or ring finger puncture. Capillary blood was collected in a microcube and the hemoglobin measurement was obtained by direct reading on a portable hemoglobinometer (Hemocue ®). A hemoglobin concentration of 11g/dL was adopted as the cut-off point for the diagnosis of anemia).									
1	observational studies	very serious[e]	serious[b]	not serious	serious[f]	none	Consumption of beans was not significantly associated with anemia prevalence (p=0.550).[g]	⊕◯◯◯ Very low	CRITICAL

Explanations

a. NIH tool for observational studies used. This study was rated as having poor quality. This is because details on methodology are lacking, which makes it challenging to determine if there is internal validity. Not much detail is provided on eligibility criteria or a description of participants. The exposure for food consumption is not well defined, all we know is that caregivers were asked about their child's food consumption at time of the interview but there are no other details provided on this.
b. Inconsistencies cannot be determined given the lack of data (n=1 study) contributing to this outcome, thus we have downgraded the certainty of evidence for this outcome by 1 level.
c. This study did not provide confidence intervals, limiting the ability to make a judgement about imprecision. It should also be noted that the sample size is small (n=179). We have downgraded the certainty of evidence for this outcome by 1 level.
d. Model adjusted by duration of gestation, mother's schooling, child's sex, anthropometric status based on the height-for-age indicator, and age (squared).
e. NIH tool for observational studies used. Overall, this study has a poor to fair rating. Firstly, there is very limited detail provided on the methodology which makes it challenging to evaluate if there is internal validity. Some considerations to note include lack of sample size justification and no details are provided on participation rate of eligible participants. Furthermore, there is a small sample size with no details provided for baseline characteristics of study sample.
f. The confidence interval around the effect is very wide (OR=0.80; 95%CI: 0.36-1.78) and the sample size is small (n=205) thus we have downgraded the certainty of evidence for this outcome by 1 level.
g. Study did not adjust for confounding variables.

Table S2.7.11: GRADE table for NPS and nutrient adequacy

Question: Is more frequent, varied, or amounts of consumption of NPS, compared to less, associated with beneficial nutrient adequacy?

Certainty assessment							Impact	Certainty	Importance
№ of studies	Study design	Risk of bias	Inconsistency	Indirectness	Imprecision	Other considerations			
**Energy intake** (**Follow-up**: 6 months; **Assessed with**: De Jager 2019 calculated energy intake using a nutrient calculation system Compl-eatTM (version 1.0, Wageningen University). Energy and nutrient intake calculations were based on a food composition table (FCT), specifically created for this study using the West African FCT as primary source, complemented with data from FCTs from Mali, the United States Department of Agriculture database, and Ghana. Negash 2014 measured energy intake using a 24-hr recall of dietary intake after 6 months of follow-up, however no further details were provided on how energy intake was assessed).									
2	1 CS and 1 RCT	serious[a]	serious[b]	not serious	serious[c]	none	For De Jager 2019, amount of legume consumption was significantly associated with intake of energy. Children who consumed legumes (median daily legume intake: 19.8g ± 31.3), and those who had no consumption, had a median energy intake of 893kcal (25th, 75th percentile=726,1142) and 596kcal (25th, 75th percentile=521,688), respectively (p<0.05 between groups).[d]<br><br>For Negash 2014, amount of broad bean consumption was not significantly associated with intake of energy after 6 months of follow-up (p>0.05).[d]	⨁◯◯◯<br>Very low	CRITICAL

Certainty assessment							Impact	Certainty	Importance
№ of studies	Study design	Risk of bias	Inconsistency	Indirectness	Imprecision	Other considerations			
**Fat intake** (**Follow-up**: 6 months; **Assessed with**: De Jager 2019 calculated fat intake using a nutrient calculation system Compl-eatTM (version 1.0, Wageningen University). Fat intake calculations were based on a food composition table (FCT), specifically created for this study using the West African FCT as primary source, complemented with data from FCTs from Mali, the United States Department of Agriculture database, and Ghana. Negash 2014 measured fat intake using a 24-hr recall of dietary intake after 6 months of follow-up, however no further details were provided on how fat intake was assessed).									
2	1 CS and 1 RCT	serious[a]	serious[e]	not serious	serious[c]	none	For De Jager 2019, amount of legume consumption was significantly associated with intake of fat. Children who consumed legumes (median daily legume intake: 19.8g ± 31.3), and those who had no consumption, had a median fat intake of 33g (25th, 75thpercentile=28,39) and 27g (25th, 75th percentile=26,29), respectively ($p<0.05$ between groups).[d]<br><br>For Negash 2014, amount of broad bean consumption was not significantly associated with intake of fat after 6 months of follow-up ($p>0.05$).[d]	⊕◯◯◯ Very low	CRITICAL
**Protein intake** (**Follow-up**: 6 months; **Assessed with**: De Jager 2019 calculated protein intake using a nutrient calculation system Compl-eatTM (version 1.0, Wageningen University). Protein intake calculations were based on a food composition table (FCT), specifically created for this study using the West African FCT as primary source, complemented with data from FCTs from Mali, the United States Department of Agriculture database, and Ghana. Negash 2014 measured protein intake using a 24-hr recall of dietary intake after 6 months of follow-up, however no further details were provided on how protein intake was assessed).									
2	1 CS and 1 RCT	serious[a]	not serious	not serious	serious[c]	none	In both studies, amount of legume and broad bean consumption was significantly associated with intake of protein. Children who consumed legumes (median daily legume intake: 19.8g ± 31.3), and those who had no consumption, had a median protein intake of 21g (25th, 75th percentile=15,28) and 11g (25th, 75th percentile=10,14), respectively ($p<0.05$ between groups).[d] Children who consumed broad beans, and those who had no consumption, had a mean protein intake of 28.7g (SD=22.6) and 21.6g (SD= 11.3), respectively after 6 months of follow-up ($p<0.05$ between groups).[d]	⊕⊕◯◯ Low	CRITICAL
**Iron intake** (**Follow-up**: 6 months; A**ssessed with**: De Jager 2019 calculated iron intake using a nutrient calculation system Compl-eatTM (version 1.0, Wageningen University). Iron intake calculations were based on a food composition table (FCT), specifically created for this study using the West African FCT as primary source, complemented with data from FCTs from Mali, the United States Department of Agriculture database, and Ghana. Negash 2014 measured iron intake using a 24-hr recall of dietary intake after 6 months of follow-up, however no further details were provided on how iron intake was assessed).									

Certainty assessment							Impact	Certainty	Importance
№ of studies	Study design	Risk of bias	Inconsistency	Indirectness	Imprecision	Other considerations			
2	1 CS and 1 RCT	serious[a]	not serious	not serious	serious[c]	none	In both studies, amount of legume and broad bean consumption was significantly associated with intake of iron. Children who consumed legumes (median daily legume intake: 19.8g ± 31.3), and those who had no consumption, had a median iron intake of 5.6mg (25th, 75th percentile= 3.5, 8.5) and 2mg (25th, 75th percentile=1.5, 3), respectively (p<0.05 between groups).[d] Children who consumed broad beans, and those who had no consumption, had a mean iron intake of 30.6mg (SD=21.2) and 20.9mg (SD= 13.4), respectively after 6 months of follow-up (p<0.05 between groups).[d]	⊕⊕◯◯ Low	IMPORTANT
**Carbohydrate intake** (**Follow-up**: 6 months; **Assessed with**: carbohydrate intake was measured using a 24-hr recall of dietary intake after 6 months of follow-up, however no further details were provided on how carbohydrate intake was assessed).									
1	randomised trials	serious[f]	serious[g]	not serious	serious[h]	none	Amount of broad bean consumption was significantly associated with intake of carbohydrates after 6 months of follow-up. Children who consumed broad beans, and those who had no consumption, had a mean carbohydrate intake of 159g (SD=105) and 127g (SD= 78), respectively (p<0.05).[d]	⊕◯◯◯ Very low	CRITICAL
**Calcium intake** (**Assessed** with: calcium intake was measured using a nutrient calculation system Compl-eatTM (version 1.0, Wageningen University). Calcium intake calculations were based on a food composition table (FCT), specifically created for this study using the West African FCT as primary source, complemented with data from FCTs from Mali, the United States Department of Agriculture database, and Ghana).									
1	observational studies	serious[i]	serious[g]	not serious	serious[j]	none	Amount of legume consumption was significantly associated with intake of calcium. Children who consumed legumes (median daily legume intake: 19.8g ± 31.3), and those who had no consumption, had a median calcium intake of 230mg (25th, 75th percentile=200,273) and 194mg (25th, 75th percentile=187,223), respectively (p<0.05 between groups).[d]	⊕◯◯◯ Very low	IMPORTANT
**Folate intake** (**Assessed with**: folate intake was measured using a nutrient calculation system Compl-eatTM (version 1.0, Wageningen University). Folate intake calculations were based on a food composition table (FCT), specifically created for this study using the West African FCT as primary source, complemented with data from FCTs from Mali, the United States Department of Agriculture database, and Ghana).									

Certainty assessment							Impact	Certainty	Importance
№ of studies	Study design	Risk of bias	Inconsistency	Indirectness	Imprecision	Other considerations			
1	observational studies	serious[i]	serious[g]	not serious	serious[j]	none	Amount of legume consumption was significantly associated with intake of folate. Children who consumed legumes (median daily legume intake: 19.8g ± 31.3), and those who had no consumption, had a median folate intake of 91ug (25th, 75th percentile=76,121) and 63ug (25th, 75th percentile=60,69), respectively (p<0.05 between groups).[d]	⨁◯◯◯ Very low	IMPORTANT
**Niacin intake** (**Assessed with**: niacin intake was measured using a nutrient calculation system Compl-eatTM (version 1.0, Wageningen University). Niacin intake calculations were based on a food composition table (FCT), specifically created for this study using the West African FCT as primary source, complemented with data from FCTs from Mali, the United States Department of Agriculture database, and Ghana).									
1	observational studies	serious[i]	serious[g]	not serious	serious[j]	none	Amount of legume consumption was significantly associated with intake of niacin. Children who consumed legumes (median daily legume intake: 19.8g ± 31.3), and those who had no consumption, had a median niacin intake of 4.7mg (25th, 75th percentile= 3.2, 6.7) and 1.9mg (25th, 75th percentile= 1.5, 2.4), respectively (p<0.05 between groups).[d]	⨁◯◯◯ Very low	IMPORTANT
**Zinc intake** (**Assessed with**: zinc intake was measured using a nutrient calculation system Compl-eatTM (version 1.0, Wageningen University). Zinc intake calculations were based on a food composition table (FCT), specifically created for this study using the West African FCT as primary source, complemented with data from FCTs from Mali, the United States Department of Agriculture database, and Ghana).									
1	observational studies	serious[i]	serious[g]	not serious	serious[j]	none	Amount of legume consumption was significantly associated with intake of zinc. Children who consumed legumes (median daily legume intake: 19.8g ± 31.3), and those who had no consumption, had a median zinc intake of 3.2mg (25th, 75th percentile= 2.3, 4.5) and 1.6mg (25th, 75th percentile= 1.4, 2.1), respectively (p<0.05 between groups).[d]	⨁◯◯◯ Very low	IMPORTANT

Explanations

a. De Jager 2019 was assessed using the NIH tool for observational studies and Negash 2014 using the RoB-2 tool for randomized studies. De Jager 2019 was rated as having fair quality because the study did not consider any confounding variables within their models. Furthermore, for the secondary recall there were only 66 responses (20%), which is a very high loss-to-follow up. Negash 2014 was rated as having some concerns for bias. This is because, the blinding and randomization processes were vaguely reported. Authors did state that the control area was far enough away to minimize contact with the intervention group (minimizing selection bias and patient awareness of allocation), however no mention of how participants were randomized and if assessors and analysts were blinded. Anthropometric data was collected by a single person. This may minimize errors in measurement but also may incite other bias if participant allocation was not concealed.

b. De Jager 2019 reported a statistically significant association between amount of legume consumption with intake of energy. Negash 2014 did not find a significant association between broad bean consumption with intake of energy. Due to these inconsistencies, we have downgraded the certainty of evidence for this outcome by 1 level.

c. Both studies did not provide confidence intervals, limiting the ability to make a judgement about imprecision. It should also be noted that the sample size is small for both studies (De Jager 2019, n=337; Negash 2014, n=197). We have downgraded the certainty of evidence for this outcome by 1 level.
d. Study did not adjust for confounding variables.
e. De Jager 2019 reported a statistically significant association between amount of legume consumption with intake of fat. Negash 2014 did not find a significant association between broad bean consumption with intake of fat. Due to these inconsistencies, we have downgraded the certainty of evidence for this outcome by 1 level.
f. RoB-2 tool for randomized trials used. This study was rated as having some concerns for bias. This is because, the blinding and randomization processes were vaguely reported. Authors did state that the control area was far enough away to minimize contact with the intervention group (minimizing selection bias and patient awareness of allocation), however no mention of how participants were randomized and if assessors and analysts were blinded. Anthropometric data was collected by a single person. This may minimize errors in measurement but also may incite other bias if participant allocation was not concealed.
g. Inconsistencies cannot be determined given the lack of data (n=1 study) contributing to this outcome, thus we have downgraded the certainty of evidence for this outcome by 1 level.
h. This study did not provide confidence intervals, limiting the ability to make a judgement about imprecision. It should also be noted that the sample size is small (n=197). We have downgraded the certainty of evidence for this outcome by 1 level.
i. NIH tool for observational studies used. This study was rated as having fair quality. It was downgraded because the study did not consider any confounding variables within their models. Furthermore, for the secondary recall there were only 66 responses (20%), which is a very high loss-to-follow up.
j. This study did not provide confidence intervals, limiting the ability to make a judgement about imprecision. It should also be noted that the sample size is relatively small (n=337). We have downgraded the certainty of evidence for this outcome by 1 level.

Table S2.7.12: GRADE table for vegetables and child development

Question: Is more frequent, varied, or amounts of consumption of vegetables, compared to less, associated with beneficial child development?

Certainty assessment							Impact	Certainty	Importance
№ of studies	Study design	Risk of bias	Inconsistency	Indirectness	Imprecision	Other considerations			
Child development: total ASQ-3 score (Follow-up: 23 months; Assessed with: The Ages and Stages Questionnaire-3 (ASQ-3): a 30-item tool that evaluates 5 different subscales of child development. The questionnaire generates an overall measure of child development as well as separate scores in gross and fine motor, personal-social, problem-solving, and communication areas. This standardized screening instrument has been used to assess child development in many global contexts including in peri-urban Nepal).									

Certainty assessment							Impact	Certainty	Importance
№ of studies	Study design	Risk of bias	Inconsistency	Indirectness	Imprecision	Other considerations			
1	observational studies	not serious	serious[a]	not serious	serious[b]	none	Frequency of eating any vegetables and green leafy vegetables at ~15 months old was significantly associated with total child development score at 23-38 months of age. In the adjusted linear model, each additional day of any vegetable consumption was significantly associated with a 9.3 point higher total score on the ASQ-3 (Reg coefficient=9.3, 95% CI: 2.4-16.3, p<0.01). In the adjusted logistic regression model, each additional day of any vegetable consumption was significantly associated with a 40% lower risk of falling into the lowest 25% group of the total ASQ-3 distribution (OR=0.6, 95% CI: 0.41-0.90, p=0.01).<br>In the adjusted linear model, each additional day of green leafy vegetable consumption was significantly associated with a 11.7 point higher total score on the ASQ-3 (Reg coefficient=11.7, 95% CI: 4.1-19.4, p<0.01). In the adjusted logistic regression model, each additional day of green leafy vegetable consumption was significantly associated with a 46% lower risk of falling into the lowest 25% group of the total ASQ-3 distribution (OR=0.54, 95% CI: 0.34-0.86, p<0.01).[c]	⨁◯◯◯ Very low	IMPORTANT
Child development: communication skills (Follow-up: 23 months; Assessed with: The Ages and Stages Questionnaire-3 (ASQ-3): a 30-item tool that evaluates 5 different subscales of child development. The questionnaire generates an overall measure of child development as well as separate scores in gross and fine motor, personal-social, problem-solving, and communication areas. This standardized screening instrument has been used to assess child development in many global contexts including in peri-urban Nepal).									
1	observational studies	not serious	serious[a]	not serious	serious[d]	none	Frequency of eating any vegetables at ~15 months old was significantly associated with communication score after 23-38 months of age. Each additional day of vegetable consumption was significantly associated with a 31% lower risk of a low score on the communication subscale (OR=0.69, 95% CI: 0.47-1.00, p<0.05). Conversely, frequency of eating green leafy vegetables was not significantly associated with communication score after 18 months of follow-up (p=0.16).[c]	⨁◯◯◯ Very low	IMPORTANT

Certainty assessment							Impact	Certainty	Importance
№ of studies	Study design	Risk of bias	Inconsistency	Indirectness	Imprecision	Other considerations			
Child development: gross motor skills (Follow-up: 23 months; Assessed with: The Ages and Stages Questionnaire-3 (ASQ-3): a 30-item tool that evaluates 5 different subscales of child development. The questionnaire generates an overall measure of child development as well as separate scores in gross and fine motor, personal-social, problem-solving, and communication areas. This standardized screening instrument has been used to assess child development in many global contexts including in peri-urban Nepal).									
1	observational studies	not serious	serious[a]	not serious	serious[e]	none	Frequency of eating any vegetables and green leafy vegetables at ~15 months old was not significantly associated with gross motor score at 23-38 months of age (p=0.76 and p=0.48, respectively).[c]	⊕◯◯◯ Very low	IMPORTANT
Child development: fine motor skills (Follow-up: 23 months; Assessed with: The Ages and Stages Questionnaire-3 (ASQ-3): a 30-item tool that evaluates 5 different subscales of child development. The questionnaire generates an overall measure of child development as well as separate scores in gross and fine motor, personal-social, problem-solving, and communication areas. This standardized screening instrument has been used to assess child development in many global contexts including in peri-urban Nepal).									
1	observational studies	not serious	serious[a]	not serious	serious[f]	none	Frequency of eating any vegetables and green leafy vegetables at ~15 months old was significantly associated with fine motor score at 23-38 months of age. Each additional day of vegetable consumption was significantly associated with a 40% lower risk of a low score on the fine motor subscale (OR=0.60, 95% CI: 0.42-0.86, p<0.01). Each additional day of green leafy vegetable consumption was significantly associated with a 38% lower risk of a low score on the fine motor subscale (OR=0.62, 95% CI: 0.42-0.93, p=0.02).[c]	⊕◯◯◯ Very low	IMPORTANT
Child development: problem-solving skills (Follow-up: 23 months; Assessed with: The Ages and Stages Questionnaire-3 (ASQ-3): a 30-item tool that evaluates 5 different subscales of child development. The questionnaire generates an overall measure of child development as well as separate scores in gross and fine motor, personal-social, problem-solving, and communication areas. This standardized screening instrument has been used to assess child development in many global contexts including in peri-urban Nepal).									
1	observational studies	not serious	serious[a]	not serious	serious[g]	none	Frequency of eating any vegetables at ~15 months old was not significantly associated with problem-solving at 23-38 months of age (p=0.09). Conversely, frequency of eating green leafy vegetables was significantly associated with problem-solving score after 18 months of follow-up. Each additional day of green leafy vegetable consumption was significantly associated with a 36% lower risk of a low score on the problem-solving subscale (OR=0.64, 95% CI: 0.44-0.93, p=0.02).[c]	⊕◯◯◯ Very low	IMPORTANT

Certainty assessment							Impact	Certainty	Importance
№ of studies	Study design	Risk of bias	Inconsistency	Indirectness	Imprecision	Other considerations			
Child development: personal-social skills (Follow-up: 23 months; Assessed with: The Ages and Stages Questionnaire-3 (ASQ-3): a 30-item tool that evaluates 5 different subscales of child development. The questionnaire generates an overall measure of child development as well as separate scores in gross and fine motor, personal-social, problem-solving, and communication areas. This standardized screening instrument has been used to assess child development in many global contexts including in peri-urban Nepal).									
1	observational studies	not serious	serious[a]	not serious	serious[h]	none	Frequency of eating any vegetables and green leafy vegetables at ~15 months old was significantly associated with personal-social score at 23-38 months of age. Each additional day of vegetable consumption was associated with a 22% lower risk of a low score on the personal-social subscale (OR=0.78, 95% CI: 0.54-1.13, p=0.02). Each additional day of green leafy vegetable consumption was associated with a 41% lower risk of a low score on the personal-social subscale (OR=0.59, 95% CI: 0.38-0.91, p=0.02).[c]	⨁◯◯◯ Very low	IMPORTANT

Explanations

a. Inconsistencies cannot be determined given the lack of data (n=1 study) contributing to this outcome, thus we have downgraded the certainty of evidence for this outcome by 1 level.
b. For both vegetable groups, the confidence interval around the effect is wide (for example for any vegetable consumption, the reg coefficient=9.3, 95% CI: 2.4,16.3) and the sample size is relatively small (n=282) thus we have downgraded the certainty of evidence for this outcome by 1 level.
c. Model was adjusted by maternal education (2 categories), wealth quintile, child age, and randomization of intervention group.
d. For both vegetable groups, the confidence interval around the effect is wide (for example for any vegetable consumption, the OR=0.69, 95% CI: 0.47,1.00) and the sample size is relatively small (n=282) thus we have downgraded the certainty of evidence for this outcome by 1 level.
e. For both vegetable groups, the confidence interval around the effect is wide (for example for any vegetable consumption, the OR=1.07, 95% CI: 0.71,1.62) and the sample size is relatively small (n=282) thus we have downgraded the certainty of evidence for this outcome by 1 level.
f. For both vegetable groups, the confidence interval around the effect is relatively wide (for example for any vegetable consumption, the OR=0.60, 95% CI: 0.42,0.86) and the sample size is relatively small (n=282) thus we have downgraded the certainty of evidence for this outcome by 1 level.
g. For both vegetable groups, the confidence interval around the effect is relatively wide (for example for green leafy vegetable consumption, the OR=0.64, 95% CI: 0.44,0.93) and the sample is relatively small (n=282) thus we have downgraded the certainty of evidence for this outcome by 1 level.
h. For both vegetable groups, the confidence interval around the effect is relatively wide (for example for any vegetable consumption, the OR=0.78, 95%CI: 0.54,1.13) and the sample size is relatively small (n=282) thus we have downgraded the certainty of evidence for this outcome by 1 level.

Table S2.7.13: GRADE table for vegetables and subsequent consumption of food item later in life

Question: Is more frequent, varied, or amounts of consumption of vegetables, compared to less, associated with subsequent consumption of food items later in life?

Certainty assessment							Impact	Certainty	Importance
№ of studies	Study design	Risk of bias	Inconsistency	Indirectness	Imprecision	Other considerations			
Stability and change in vegetable consumption (Follow-up: 66 months; Assessed with: a questionnaire at 3 time points (18 months, 36 months, and 7 years of child age), reported by a parent).									
1	observational studies	very serious[a]	serious[b]	not serious	serious[c]	none	In boys, overall vegetable consumption at 18 months was positively associated with overall vegetable consumption at 36 months (spearman's rho=0.36) and at 7 years of age (spearman's rho=0.28).<br>In girls, overall vegetable consumption at 18 months was positively associated with overall vegetable consumption at 36 months (spearman's rho=0.37) and at 7 years of age (spearman's rho=0.31).[d]	⨁◯◯◯ Very low	IMPORTANT
Intake of new foods (Follow-up: 2 months; Assessed with: an infant food diary. Throughout the study, each mother kept a diary noting the foods and drinks given to her infant).									
1	observational studies	very serious[e]	serious[b]	not serious	serious[f]	none	There was a significant association for type of variety experience with the high vegetable variety producing the greatest increase in intake of new foods (p<0.0001) at study endline (~2 months).[d]	⨁◯◯◯ Very low	IMPORTANT
Mean number of vegetables eaten by infants (Follow-up: 67 months; Assessed with: At each follow-up age (15 months, 3 and 6 years), a questionnaire was used to identify the number of vegetables offered and the number of vegetables eaten and liked by the child. At 6 years of age, consumption and liking tests in the laboratory were used to measure acceptance of new and familiar vegetables).									
1	observational studies	very serious[g]	serious[b]	not serious	serious[h]	none	At follow-up 3 (~67 months), early variety experience was significantly associated with mean number of vegetables eaten (p<0.05), but not at follow-up 1 (~9 months) (p=0.20) or 2 (~31 months) (p=0.0635). At follow-up 3 (~67 months), children who had experienced a high variety of vegetables at weaning ate more of the new vegetables and familiar vegetables than those who had experienced low or no variety (14.1g ± 1.5 vs. 4.3g ± 1.5 and 3.2g ± 1.4, p<0.0001 for new vegetables, respectively; and 9.6g ± 2.0 vs. 13.1g ± 2.0 and 13.1g ± 1.9, p=0.03 for familiar vegetables, respectively).[d]	⨁◯◯◯ Very low	IMPORTANT
Vegetable and fruit intake (Follow-up: 29 months; Assessed with: the Children's Dietary Questionnaire at age 3.7 years).									

Certainty assessment							Impact	Certainty	Importance
№ of studies	Study design	Risk of bias	Inconsistency	Indirectness	Imprecision	Other considerations			
1	observational studies	not serious	serious[b]	not serious	serious[i]	none	A greater variety of vegetables tried at age 14 months was significantly associated with a higher fruit and vegetable intake score at age 3.7 years (Reg coefficient=0.12, p=0.05).[d]	⨁◯◯◯ Very low	IMPORTANT

Explanations

a. NIH tool for observational studies used. Overall, study has a rating of poor and lacks internal validity. Hard to determine whether information bias has occurred due to missing methods and detail in the report. For example, study population, exposure measures, and outcome measures are never clearly defined. Additionally, the study does not report accounting for any confounding variables, and it appears that measurement bias could have occurred. Results should be interpreted with caution.
b. Inconsistencies cannot be determined given the lack of data (n=1 study) contributing to this outcome, thus we have downgraded the certainty of evidence for this outcome by 1 level.
c. This study did not provide confidence intervals, limiting the ability to make a judgement about imprecision. It should be noted that the sample size is large (n=9,490), however given the lack of confidence intervals provided we have downgraded the certainty of evidence for this outcome by 1 level.
d. Study did not adjust for confounding variables.
e. ROBINS-I tool for non-randomized studies used. There are major limitations to this intervention study, including but not limited to, lack of description of how infants were selected for each intervention group, mothers were not blinded to intervention food and were the ones reporting on child's liking of food which could have been biased by knowledge of food consumed, many missing details on methodology and no confounding variables considered in statistical models. Of important note, the foods provided for children were made by Nestle, the funding for the study was by Nestle, and multiple study authors work for Nestle, results from this study should be considered with caution.
f. This study did not provide confidence intervals, limiting the ability to make a judgement about imprecision. It should also be noted that the sample size is small (n=147). We have downgraded the certainty of evidence for this outcome by 1 level.
g. ROBINS-I tool for non-randomized studies used. There are major limitations to this intervention study, including but not limited to, lack of description of how infants were selected for each intervention group, mothers were not blinded to intervention food and were the ones reporting on child's liking of food which could have been biased by knowledge of food consumed, many missing details on methodology and no confounding variables considered in statistical models. Of important note, the foods provided for children were made by Nestle, the funding for the study was by Nestle, and multiple study authors work for Nestle, results from this study should be considered with caution.
h. This study did not provide confidence intervals, limiting the ability to make a judgement about imprecision. It should also be noted that the sample size is small (n=107). We have downgraded the certainty of evidence for this outcome by 1 level.
i. This study did not provide confidence intervals, limiting the ability to make a judgement about imprecision. It should also be noted that the sample size is relatively small (n=333). We have downgraded the certainty of evidence for this outcome by 1 level.

Table S2.7.14: GRADE table for fruit and subsequent consumption of food item later in life

Question: Is more frequent, varied, or amounts of consumption of fruit, compared to less, associated with subsequent consumption of food items later in life?

Certainty assessment							Impact	Certainty	Importance
№ of studies	Study design	Risk of bias	Inconsistency	Indirectness	Imprecision	Other considerations			
Stability and change in fruit consumption (Follow-up: 66 months; Assessed with: a questionnaire at 3 time points (18 months, 36 months, and 7 years of child age), reported by a parent).									

Certainty assessment							Impact	Certainty	Importance
№ of studies	Study design	Risk of bias	Inconsistency	Indirectness	Imprecision	Other considerations			
1	observational studies	very serious[a]	serious[b]	not serious	serious[c]	none	In boys, overall fruit consumption at 18 months was positively associated with overall fruit consumption at 36 months (spearman's rho=0.36) and at 7 years of age (spearman's rho=0.23). In girls, overall fruit consumption at 18 months was positively associated with overall fruit consumption at 36 months (spearman's rho=0.36) and at 7 years of age (spearman's rho=0.24).[d]	⨁◯◯◯ Very low	IMPORTANT
Fruit and vegetable intake (Follow-up: 29 months; Assessed with: the Children's Dietary Questionnaire at age 3.7 years).									
1	observational studies	not serious	serious[b]	not serious	serious[e]	none	A greater variety of fruits tried at age 14 months was significantly associated with a higher fruit and vegetable intake score at age 3.7 years (Reg coefficient=0.19, p=0.003).[d]	⨁◯◯◯ Very low	IMPORTANT

Explanations

a. NIH tool for observational studies used. Overall, study has a rating of poor and lacks internal validity. Hard to determine whether information bias has occurred due to missing methods and detail in the report. For example, study population, exposure measures, and outcome measures are never clearly defined. Additionally, the study does not report accounting for any confounding variables, and it appears that measurement bias could have occurred. Results should be interpreted with caution.
b. Inconsistencies cannot be determined given the lack of data (n=1 study) contributing to this outcome, thus we have downgraded the certainty of evidence for this outcome by 1 level.
c. This study did not provide confidence intervals, limiting the ability to make a judgement about imprecision. It should be noted that the sample size is large (n=9,490), however given the lack of confidence intervals provided we have downgraded the certainty of evidence for this outcome by 1 level.
d. Study did not adjust for confounding variables.
e. This study did not provide confidence intervals, limiting the ability to make a judgement about imprecision. It should also be noted that the sample size is relatively small (n=333). We have downgraded the certainty of evidence for this outcome by 1 level.

Table S2.7.15: GRADE table for FV and subsequent consumption of food item later in life

Question: Is more frequent, varied, or amounts of consumption of FV, compared to less, associated with subsequent consumption of food items later in life?

Certainty assessment							Impact	Certainty	Importance
№ of studies	Study design	Risk of bias	Inconsistency	Indirectness	Imprecision	Other considerations			
Stability and tracking of FV patterns (Follow-up: 15 months; Assessed with: Food intake of the children was collected by monthly 24 hr recalls where the mother or caregiver answered questions about all meals, foods and amounts consumed on the day prior to the interview).									

Certainty assessment							Impact	Certainty	Importance
№ of studies	Study design	Risk of bias	Inconsistency	Indirectness	Imprecision	Other considerations			
1	observational studies	serious[a]	serious[b]	not serious	serious[c]	none	There was moderate stability for the frequency of consumption of yellow fruits and vegetables and dark green leafy vegetable consumption using GEE models (stability coefficient=0.26, 95% CI: 0.18-0.35).<br>For intake of yellow fruits and vegetables and dark green leafy vegetables, tracking coefficients were mostly fair and decreased throughout follow-up from 0.27 for the 13–16-month time slot to 0.19 for the 21–24-month time slot (Kw=0.273 for 13-16 months; Kw=0.234 for 17-20 months; Kw=0.194 for 21-24 months).[d]	⊕◯◯◯ Very low	IMPORTANT

Explanations

a. NIH tool for observational studies used. This study was rated as having fair quality. Reasons for downgrading the certainty of evidence by 1 level include missing details on the participants included into the study and no sample size justification. It should be noted that dietary data below 9 months of age (6-9 months) was excluded due to a change in the methodology used for dietary data collection at 9 months.
b. Inconsistencies cannot be determined given the lack of data (n=1 study) contributing to this outcome, thus we have downgraded the certainty of evidence for this outcome by 1 level.
c. The confidence interval around the effect is relatively wide (stability coefficient=0.26, 95% CI: 0.18,0.35) and the sample size is small (n=231) thus we have downgraded the certainty of evidence for this outcome by 1 level.
d. Model was adjusted by the Water, Assets, Mother's education, and Income (WAMI) Index, maternal age, parity and child's gender. Since these adjustments made no further changes to the estimates, only results from the unadjusted models and those adjusted for WAMI are presented.

Table S2.7.16: GRADE table for vegetables and diarrhea

Question: Is more frequent, varied, or amounts of consumption of vegetables, compared to less, associated with decreased prevalence of diarrhea?

Certainty assessment							Impact	Certainty	Importance
№ of studies	Study design	Risk of bias	Inconsistency	Indirectness	Imprecision	Other considerations			
Diarrhea (Assessed with: Any episode of diarrhea among children in the 2 weeks preceding the survey was reported by the mothers in response to specific questions).									
1	observational studies	serious[a]	serious[b]	not serious	serious[c]	none	Frequency of eating green leafy or orange color vegetables was not significantly associated with diarrhea prevalence ($p>0.05$).[d]	⊕◯◯◯ Very low	CRITICAL

Explanations

a. NIH tool for observational studies used. This study was rated as having fair quality. This was because, this study did not discuss eligibility criteria or provide a description for participants who were included in the analysis. Additional limitations are from the nature of cross-sectional design, i.e., recall bias and lack of causality.
b. Inconsistencies cannot be determined given the lack of data (n=1 study) contributing to this outcome, thus we have downgraded the certainty of evidence for this outcome by 1 level.

c. The confidence interval around the effect is wide (OR=1.02, 95% CI: 0.87,1.19). It should be noted that the sample size is very large (n=4,026), however given the wide confidence intervals we have downgraded the certainty of evidence for this outcome by 1 level.
d. Model was adjusted by household wealth quintile, education of mother, geographical area, and residential location.

Table S2.7.17: GRADE table for NPS and diarrhea prevalence

Question: Is more frequent, varied, or amounts of consumption of NPS, compared to less, associated with decreased prevalence of diarrhea?

Certainty assessment							Impact	Certainty	Importance
№ of studies	Study design	Risk of bias	Inconsistency	Indirectness	Imprecision	Other considerations			
**Diarrhea** (**Assessed with**: Any episode of diarrhea among children in the 2 weeks preceding the survey was reported by the mothers in response to specific questions).									
1	observational studies	serious[a]	serious[b]	not serious	serious[c]	none	Frequency of eating pulses and seeds was not significantly associated with diarrhea prevalence (p>0.05 for both food items).[d]	⊕◯◯◯ Very low	CRITICAL

Explanations

a. NIH tool for observational studies used. This study was rated as having fair quality. This was because, this study did not discuss eligibility criteria or provide a description for participants who were included in the analysis. Additional limitations are from the nature of cross-sectional design, i.e., recall bias and lack of causality.
b. Inconsistencies cannot be determined given the lack of data (n=1 study) contributing to this outcome, thus we have downgraded the certainty of evidence for this outcome by 1 level.
c. For both food items, the confidence interval around the effect is wide (for example for pulses the OR=1.13, 95% CI: 0.85,1.51) and the sample size is relatively small (n=291) thus we have downgraded the certainty of evidence for this outcome by 1 level.
d. Model was adjusted by household wealth quintile, education of mother, geographical area, and residential location.

Table S2.7.18: GRADE table for vegetables and food/taste preferences later in life

Question: Is more frequent, varied, or amounts of consumption of vegetables, compared to less, associated with food/taste preferences later in life?

Certainty assessment							Impact	Certainty	Importance
№ of studies	Study design	Risk of bias	Inconsistency	Indirectness	Imprecision	Other considerations			
Infant's liking (Follow-up: up to 67 months; Assessed with: Maier 2008 measured infant's liking through mother and study observer report. The mother and/or observer rated how much they thought the infant had liked the meal using a 9-point scale anchored at 1= ‘‘did not like at all’’ to 9= ‘‘liked very much.’’ The associated report of this study (Maier-Noth 2016) measured infant's liking at each follow-up age (15 months, 3 and 6 years) using a questionnaire to identify the number of vegetables offered and the number of vegetables eaten and liked by the child. At 6 years of age, consumption and liking tests in the laboratory were used to measure acceptance of new and familiar vegetables. Acceptance was evaluated as the number of vegetables that the child “ate and liked.” Mallan 2016 measured infant's liking using the Child Food Preferences Questionnaire.									

Certainty assessment							Impact	Certainty	Importance
№ of studies	Study design	Risk of bias	Inconsistency	Indirectness	Imprecision	Other considerations			
3	observational studies	very serious[a]	not serious	not serious	serious[b]	none	For all three reports, there was a significant association between a greater variety of vegetables consumed with infant's liking of vegetables and/or fruits later in life. In Maier 2008, the high variety group had the highest liking scores reported by both mothers and observers after ~2 months of follow-up (p<0.0001).[b] In Mallan 2016, a greater variety of vegetables tried at age 14 months was significantly associated with liking a greater number of fruits (Reg coefficient=0.14, p=0.022) and vegetables (Reg coefficient= 0.15, p=0.017) at age 3.7 years as reported by caregivers.[b] In Maier-Noth 2016, children who had experienced a high variety of vegetables at weaning liked the new vegetables and familiar vegetables more than those who had low or no variety (scores of 4.4 ± 0.3 vs. 2.5 ± 0.3 and 2.9± 0.3, p=0.0002 for new vegetables, respectively; and scores of 5.1 ± 0.2 vs. 4.2 ± 0.2 and 4.4 ± 0.2, p=0.03 for familiar vegetables, respectively) after 67 months of follow-up.[c]	⨁◯◯◯ Very low	NOT IMPORTANT

Infant's liking (Follow-up: 6 months; Assessed with: A series of validated questionnaires to examine infants' feeding behaviour including their habitual vegetable intake through an adapted FFQ (Hammond et al., 1993) and Child Eating Behaviour Questionnaire (CEBQ; Wardle, Guthrie, Sanderson, & Rapoport, 2001). Intake was weighed and liking rated on days 25–26 and 33–35 after the start of complementary feeding in the laboratory, supplemented by the same data recorded at home).

Certainty assessment							Impact	Certainty	Importance
№ of studies	Study design	Risk of bias	Inconsistency	Indirectness	Imprecision	Other considerations			
1	randomised trials	very serious[d]	serious[e]	not serious	serious[f]	none	At 6 months follow-up, liking for vegetables was significantly different between intervention and control children (p=0.029).<br>Children who consumed carrots more frequently (nine times over the 35-day intervention), compared to less frequent consumption (three times over the 35-day intervention), had a mean infant liking of 7.14 (SEM=0.53) and 5.69 (SEM=0.49), respectively (p=0.05).<br>Children who consumed green beans more frequently (nine times over the 35-day intervention), compared to less frequent consumption (three times over the 35-day intervention), had a mean infant liking of 6.14 (SEM=0.62) and 4.56 (SEM=0.58), respectively (p=0.07).[c]	⊕◯◯◯ Very low	NOT IMPORTANT
Willingness to taste (follow-up: 67 months; assessed with: using a questionnaire at 15 months, 3 and 6 years to identify the number of vegetables offered and the number of vegetables eaten and liked by the child. At 6 years of age, consumption and liking tests in the laboratory were used to measure acceptance of new and familiar vegetables. Willingness to taste was assessed as the number of vegetables that were at least tasted).									
1	observational studies	very serious	serious[e]	not serious	serious[g]	none	At follow-up 3 (~67 months), early variety experience was significantly associated with mean willingness to taste vegetables (p<0.05). Children who had experienced a high variety of vegetables at weaning, tasted more vegetables than those who had experienced low or no variety (8.9 ± 0.6 vs. 5.6 ± 0.6 and 5.6 ± 0.6, p=0.0001, respectively) after 67 months of follow-up.[c]	⊕◯◯◯ Very low	NOT IMPORTANT
Fussiness (Follow-up: 29 months; Assessed with: the Fussiness subscale from the Children's Eating Behaviour Questionnaire).									
1	observational studies	not serious	serious[e]	not serious	serious[h]	none	Having tried fewer vegetables at age 14 months was significantly associated with a higher fussiness score at age 3.7 years (Reg coefficient= -0.12, p=0.03).[i]	⊕◯◯◯ Very low	NOT IMPORTANT

Explanations

a. Maier 2008 and the associated report (Maier-Noth 2016) were rated as having very poor quality using the ROBINS-I tool for non-randomized studies. There are major limitations to these intervention reports, including but not limited to, lack of description of how infants were selected for each intervention group, mothers were not blinded to intervention food and were the ones reporting on child's liking of food which could have been biased by knowledge of food consumed, many missing details on methodology and no confounding variables

considered in statistical models. Of important note, the foods provided for children were made by Nestle, the funding for the study was by Nestle, and multiple study authors work for Nestle, results from this study should be considered with caution. Mallan 2016 was rated as good quality using the NIH tool for observational studies.
b. None of the studies provided confidence intervals, limiting the ability to make a judgement about imprecision. It should also be noted that the sample size is small in all studies (ranging from n=107 to n=339). Thus, we have downgraded the certainty of evidence for this outcome by 1 level.
c. Study did not adjust for confounding variables.
d. RoB-2 tool for RCTs used. This study was rated as having a high risk of bias. This is because, there was no discussion of blinding, the methods are poorly explained and lacking detail (for example no explanation of how groups were randomized into IG vs CG), the analyses carried out were not pre-specified and did deviate from the intended plan based on lack of data collected for 18-month follow-up period. There were no confounding variables discussed or taken into account and a very high loss-to follow up (only 38% response rate at 18 months).
e. Inconsistencies cannot be determined given the lack of data (n=1 study) contributing to this outcome, thus we have downgraded the certainty of evidence for this outcome by 1 level.
f. This study did not provide confidence intervals, limiting the ability to make a judgement about imprecision. It should also be noted that the sample size is very small (n=36). We have downgraded the certainty of evidence for this outcome by 1 level.
g. This study did not provide confidence intervals, limiting the ability to make a judgement about imprecision. It should also be noted that the sample size is small (n=107). We have downgraded the certainty of evidence for this outcome by 1 level.
h. This study did not provide confidence intervals, limiting the ability to make a judgement about imprecision. It should also be noted that the sample size is relatively small (n=340). We have downgraded the certainty of evidence for this outcome by 1 level.
i. Model was adjusted by fussiness score at age 14 months.

Table S2.7.19: GRADE table for fruit and food/taste preferences later in life

Question: Is more frequent, varied, or amounts of consumption of fruit, compared to less, associated with food/taste preferences later in life?

Certainty assessment							Impact	Certainty	Importance
№ of studies	Study design	Risk of bias	Inconsistency	Indirectness	Imprecision	Other considerations			
Infant's liking of fruit and vegetables (Follow-up: 29 months; Assessed with: At each follow-up age (15 months, 3 and 6 years), a questionnaire was used to identify the number of vegetables offered and the number of vegetables eaten and liked by the child. At 6 years of age, consumption and liking tests in the laboratory were used to measure acceptance of new and familiar vegetables. Acceptance was evaluated as the number of vegetables that the child "ate and liked").									
1	observational studies	not serious	serious[a]	not serious	serious[b]	none	A greater variety of fruits tried at 14 months was significantly associated with liking a greater number of fruits (Reg coefficient=0.16, p=0.007) at age 3.7 years. However, number of fruits tried at 14 months was not significantly associated with liking vegetables at age 3.7 years (p=0.12).[c]	⊕◯◯◯ Very low	NOT IMPORTANT
Fussiness (Follow-up: 29 months; Assessed with: At each follow-up age (15 months, 3 and 6 years), a questionnaire was used to identify the number of vegetables offered and the number of vegetables eaten and liked by the child. At 6 years of age, consumption and liking tests in the laboratory were used to measure acceptance of new and familiar vegetables. Acceptance was evaluated as the number of vegetables that the child "ate and liked").									
1	observational studies	not serious	serious[a]	not serious	serious[d]	none	Variety of fruit consumption at age 14 months was not significantly associated with fussiness score at age 3.7 years (p=0.72). [e]	⊕◯◯◯ Very low	NOT IMPORTANT

Explanations

a. Inconsistencies cannot be determined given the lack of data (n=1 study) contributing to this outcome, thus we have downgraded the certainty of evidence for this outcome by 1 level.

b. This study did not provide confidence intervals, limiting the ability to make a judgement about imprecision. It should also be noted that the sample size is relatively small (n=340). We have downgraded the certainty of evidence for this outcome by 1 level.
c. Study did not adjust for confounding variables.
d. This study did not provide confidence intervals, limiting the ability to make a judgement about imprecision. It should also be noted that the sample size is relatively small (n=339). We have downgraded the certainty of evidence for this outcome by 1 level.
e. Model was adjusted by fussiness score at age 14 months.

S2.8. Summary of Key Findings for ASF

**Frequency of ASF**			
**Type of Food**	**Outcome**	**Effect Estimate**	**n; Heterogeneity I^2; p-value; GRADE certainty**
**Randomized Controlled Trials**			
Egg	Preferences	Study narratively reported “children who consumed eggs at baseline were twice more likely to consume them at endline (OR = 2.25, 95% CI 1.38, 3.66)”	
Red Meat	Haemoglobin	**5.00 [0.85, 9.15]**	n=1; NA; GRADE: very low
	Serum iron	1.00 [-1.09, 3.09]	n=1; NA
	Serum transferrin	-9.00 [-24.39, 6.39]	n=1; NA
	Serum ferritin	-0.10 [-0.28, 0.08]	n=1; NA
	Zinc status	Study narratively reported “those children who consumed red meat more frequently as compared to those who only consumed it 3 times/week had lower zinc status suggesting that more iron in red meat interferes with the zinc absorption”	
Meat	Energy (5 months)	-10.00 [-72.74, 52.74]	n=1; NA
	Weight (g) (7-12 months)	15.00 [-33.52, 63.52]	n=1; NA
	Length (cm) (7-12 months)	-0.02 [-0.15, 0.11]	n=1; NA
	Head circumference (cm) (7-12 months)	**0.07 [0.02, 0.12]**	n=1; NA
	Haemoglobin	-0.50 [-1.12, 0.12]	n=1; NA; GRADE: very low
	HCT	-0.60 [-1.71, 0.51]	n=1; NA

	Ferritin	-0.80 [-7.77, 6.17]	n=1; NA
	Zinc status	-1.10 [-37.50, 35.30]	n=1; NA
**Observational Studies**			
Meat consumption	Stunting	1.01 [0.86, 1.20]	n=1; NA; GRADE: Very Low
	Wasting	1.01 [0.63, 1.62]	n=1; NA; GRADE: Very Low
	Underweight	1.09 [0.86, 1.38]	n=1; NA; GRADE: Very Low
≥3 times/week vs. 1-2 times/week; meat consumption	Stunting	1.10 [0.61, 1.96]	n=1; NA; GRADE: Very Low
≥3 times/week vs. Never; meat consumption		1.21 [0.72, 2.04]	n=1; NA; GRADE: Very Low
1-2 times/week vs. Never; meat consumption		1.10 [0.76, 1.61]	n=1; NA; GRADE: Very Low
≥3 times/week vs. 1-2 times/week; meat consumption	Wasting	1.28 [0.64, 2.56]	n=1; NA; GRADE: Very Low
≥3 times/week vs. Never; meat consumption		1.17 [0.64, 2.14]	n=1; NA; GRADE: Very Low
1-2 times/week vs. Never; meat consumption		0.92 [0.57, 1.46]	n=1; NA; GRADE: Very Low
≥3 times/week vs. 1-2 times/week;	Underweight	1.65 [0.96, 2.83]	n=1; NA; GRADE: Very Low

meat consumption			
≥3 times/week vs. Never; meat consumption		**1.66 [1.06, 2.61]**	n=1; NA; GRADE: Very Low
1-2 times/week vs. Never; meat consumption		1.01 [0.66, 1.53]	n=1; NA; GRADE: Very Low
Red Meat (daily or weekly vs <week)	Anemia	0.80 [0.63, 1.00]	n=1; NA; GRADE: Very Low
Red meat consumption vs no consumption		**0.74 [0.59, 0.94]**	n=1; NA; GRADE: Very Low
Organ meat Liver (daily or weekly vs <week)	Anemia	0.64 [0.39, 1.05]	n=1; NA; GRADE: Very Low
Organ meat consumption vs no consumption		0.94 [0.74, 1.20]	n=1; NA; GRADE: Very Low
≥4 times/week vs. 1-3 times/week; egg consumption	Stunting	1.14 [0.79, 1.65]	n=1; NA; GRADE: Very Low
≥4 times/week vs. Never; egg consumption		1.16 [0.77, 1.74]	n=1; NA; GRADE: Very Low
1-3 times/week vs. Never; egg consumption		1.01 [0.66, 1.54]	n=1; NA; GRADE: Very Low

≥4 times/week vs. 1-3 times/week; egg consumption	Wasting	1.07 [0.70, 1.62]	n=1; NA; GRADE: Very Low
≥4 times/week vs. Never; egg consumption		1.17 [0.73, 1.90]	n=1; NA; GRADE: Very Low
1-3 times/week vs. Never; egg consumption		1.10 [0.68, 1.79]	n=1; NA; GRADE: Very Low
≥4 times/week vs. 1-3 times/week; egg consumption	Underweight	1.34 [0.91, 1.97]	n=1; NA; GRADE: Very Low
≥4 times/week vs. Never; egg consumption		1.28 [0.84, 1.94]	n=1; NA; GRADE: Very Low
1-3 times/week vs. Never; egg consumption		0.95 [0.61, 1.50]	n=1; NA; GRADE: Very Low
Eggs	Stunting	1.01 [0.87, 1.17]	n=1; NA; GRADE: Very Low
	Wasting	1.01 [0.87, 1.17]	n=1; NA; GRADE: Very Low
	Underweight	1.06 [0.86, 1.30]	n=1; NA; GRADE: Very Low
≥4 times/week vs. 1-3 times/week; fish consumption	Stunting	1.22 [0.77, 1.91]	n=1; NA; GRADE: Very Low
≥4 times/week vs. Never; fish consumption		1.17 [0.80, 1.71]	n=1; NA; GRADE: Very Low

1-3 times/week vs. Never; fish consumption		0.96 [0.57, 1.60]	n=1; NA; GRADE: Very Low
≥4 times/week vs. 1-3 times/week; fish consumption	Wasting	**0.52 [0.34, 0.80]**	n=1; NA; GRADE: Low
≥4 times/week vs. Never; fish consumption		0.73 [0.50, 1.04]	n=1; NA; GRADE: Low
1-3 times/week vs. Never; fish consumption		1.39 [0.94, 2.03]	n=1; NA; GRADE: Low
≥4 times/week vs. 1-3 times/week; fish consumption	Underweight	0.82 [0.54, 1.23]	n=1; NA; GRADE: Low
≥4 times/week vs. Never; fish consumption		1.07 [0.71, 1.60]	n=1; NA; GRADE: Low
1-3 times/week vs. Never; fish consumption		1.31 [0.81, 2.11]	n=1; NA; GRADE: Low
Any ASF	Stunting	**0.80 [0.71, 0.89]**	n=1; NA; GRADE: Low
	Wasting	1.12 [0.72, 1.74]	n=1; NA; GRADE: Low
	Underweight	**0.78 [0.66, 0.92]**	n=1; NA; GRADE: Low
Milk	Stunting	**0.72 [0.59, 0.88]**	n=1; NA; GRADE: Low
	Wasting	0.61 [0.31, 1.18]	n=1; NA; GRADE: Low
	Underweight	0.78 [0.59, 1.02]	n=1; NA; GRADE: Low
Cheese	Atopic dermatitis,	Study narratively reported "cheese consumption was significantly associated with less occurrence of atopic dermatitis (OR = 0.51 [0.29-0.90], P = 0.02) and food allergy (OR = 0.32,	

	rhinitis, and atopic sensitization	[0.15-0.71], P = 0.004), but there was no effect of it on atopic sensitization, allergic rhinitis, and asthma at 6 years"	
Cow milk formula	Anemia	**0.21 [0.13, 0.36]**	n=1; NA

**Amount of ASF**			
**Type of Food**	**Outcome**	**Effect Estimate**	**n; Heterogeneity I^2; p-value; GRADE certainty**
**Randomized Controlled Trials**			
Meat	Energy (5-7 months)	SMD -0.06 [-0.46, 0.33]	n=1; NA
	Energy (8-10 months)	SMD -0.09 [-0.48, 0.31]	n=1; NA
	Ferritin (7 months)	SMD -0.09 [-0.49, 0.31]	n=1; NA
Meat (beef, pork, lamb, turkey, and/ or cod)	Energy	SMD -0.13 [-0.74, 0.49]	n=1; NA
	Haemoglobin	SMD 0.21 [-0.40, 0.83]	n=1; NA; GRADE: Low
	Ferritin	SMD 0.01 [-1.03, 1.04]	n=1; NA
	Ferritin (infants with illness excluded)	SMD 0.01 [-1.03, 1.04]	n=1; NA
	Transferrin receptor	SMD -0.10 [-5.23, 5.03]	n=1; NA
	Change in height	MD -0.10 [-1.77, 1.57]	n=1; NA; GRADE: Very low
	Change in weight	MD 0.08 [-0.53, 0.70]	n=1; NA; GRADE: Very low
	Zinc intake	SMD -0.11 [-0.83, 0.60]	n=1; NA

	Change in Triceps skinfold	SMD -0.10 [-5.39, 5.19]	n=1; NA; GRADE: Very low
Red meat	Haemoglobin	MD -1.60 [-5.29, 2.09]	n=1; NA
	Ferritin	MD 2.90 [-7.91, 13.71]	n=1; NA
	Transferrin	MD 0.10 [-0.46, 0.66]	n=1; NA
	Body Iron	MD 0.30 [-0.95, 1.55]	n=1; NA
Lyophilized Beef	WAZ (45g of cooked meat/day VS 105Kcal/day of cereal)	**MD -0.13 [-0.25, -0.01]**	n=1; NA; GRADE: Low
	Transferrin receptors (30g of cooked meat/day VS 70Kcal/day of cereal)	**SMD: 1.60, [0.69, 2.51]**	n=1; NA
	HCAZ (45g of cooked meat/day VS 105Kcal/day of cereal)	**MD -0.19 [-0.34, -0.04]**	n=1; NA; GRADE: Low
	Vitamin B-12	Study narratively reported no significant difference	
	Zinc status	Study narratively reported no significant difference	
Pork	WAZ	**0.08 [0.01, 0.15]**	n=1; NA; GRADE: Moderate
	HAZ	**0.11 [0.03, 0.19]**	n=1; NA; GRADE: Moderate
	WHZ	0.03 [-0.06, 0.12]	n=1; NA; GRADE: Moderate
	HCAZ	-0.01 [-0.07, 0.05]	n=1; NA; GRADE: Moderate
	Change in height	**0.26 [0.05, 0.47]**	n=1; NA; GRADE: Moderate

	Change in weight	0.07 [0.00, 0.14]	n=1; NA; GRADE: Moderate
	Change in head circumference	**2.98 [2.90, 3.06]**	n=1; NA; GRADE: Moderate
Eggs	WAZ	MD 0.15[0.00, 0.30]	2 studies n= 743 participants; heterogeneity= I^2 0%, Chi^2 P 0.66; GRADE: Moderate
	HAZ	MD 0.07 [0.07, 0.20]	3 studies n= 1017 participants; heterogeneity= I^2 0%, Chi^2 P 0.43; GRADE: Low
	WHZ	MD -0.09 [0.23, 0.05]	3 studies n= 1007 participants; heterogeneity= I^2 25%, Chi^2 P 0.26; GRADE: Low
	Stunting	RR 0.70; [0.55, 0.90]	2 studies, n= 412 participants; heterogeneity= I^2 0%, Chi^2 P 0.99
Skimmed milk	Change in Triceps Skinfold (6 months)	0.00 [-0.22, 0.22]	n=1; NA; GRADE: Moderate
	Change in Triceps Skinfold (15 months)	-0.10 [-0.38, 0.18]	n=1; NA; GRADE: Moderate
	Change in head circumference (6 months)	**-0.30 [-0.58, -0.02]**	n=1; NA; GRADE: Moderate
	Change in head circumference (15 months)	**-0.40 [-0.76, -0.04]**	n=1; NA; GRADE: Moderate
Fortified milk	Haemoglobin	1.30 [-2.65, 5.25]	n=1; NA
	Ferritin	13.60 [-0.84, 28.04]	n=1; NA
	Transferrin	-0.50 [-1.12, 0.12]	n=1; NA
	Body Iron	**1.60 [0.29, 2.91]**	n=1; NA
Fish	Linoleic acid	-**0.41 [-0.80, -0.02]**	n=1; NA
	Alpha Linoleic acid	0.00 [-0.01, 0.01]	n=1; NA
	Eicosapentaenoic acid	**2.43 [1.91, 2.94]**	n=1; NA

Caterpillar	Hemoglobin	**SMD: 0.35, [0.02, 0.69]**	n=1; NA
	Serum Ferritin	**SMD: -0.39 [-0.73, -0.05]**	n=1; NA
	Transferrin receptor	SMD: -1.10 [-2.62, 0.42]	n=1; NA
**Observational Studies**			
Cow milk	Haemoglobin	0.00 [-0.52, 0.52]	n=1; NA; GRADE: Very Low
	Serum Ferritin	0.43 [-0.12, 0.97]	n=1; NA
	Transferrin Receptor	-1.20 [-11.03, 8.63]	n=1; NA
	Mean Corpuscular Volume	-4.20 [-61.10, 52.70]	n=1; NA
Meat	Q5 vs Q4; Height	**1.00 [0.61, 1.39]**	n=1; NA
	Q5 vs Q3; Height	**1.90 [1.37, 2.43]**	n=1; NA
	Q5 vs Q2; Height	**0.50 [0.12, 0.88]**	n=1; NA
	Q5 vs Q1; Height	**1.40 [1.02, 1.78]**	n=1; NA
	Q4 vs Q3; Height	**0.90 [0.36, 1.44]**	n=1; NA
	Q4 vs Q2; Height	**-0.50 [-0.89, -0.11]**	n=1; NA
	Q4 vs Q1; Height	**0.40 [0.01, 0.79]**	n=1; NA
	Q3 vs Q2; Height	**-1.40 [-1.94, -0.86]**	n=1; NA
	Q3 vs Q1; Height	-0.50 [-1.04, 0.04]	n=1; NA
	Q2 vs Q1; Height	0.90 [0.51, 1.29]	n=1; NA
	Q5 vs Q4; Weight	**0.30 [0.11, 0.49]**	n=1; NA
	Q5 vs Q3; Weight	**0.40 [0.21, 0.59]**	n=1; NA
	Q5 vs Q2; Weight	0.10 [-0.08, 0.28]	n=1; NA
	Q5 vs Q1; Weight	**0.70 [0.52, 0.88]**	n=1; NA
	Q4 vs Q3; Weight	0.10 [-0.09, 0.29]	n=1; NA
	Q4 vs Q2; Weight	**-0.20 [-0.38, -0.02]**	n=1; NA
	Q4 vs Q1; Weight	**0.40 [0.22, 0.58]**	n=1; NA

	Q3 vs Q2; Weight	**-0.30 [-0.49, -0.11]**	n=1; NA
	Q3 vs Q1; Weight	**0.30 [0.11, 0.49]**	n=1; NA
	Q2 vs Q1; Weight	**0.60 [0.43, 0.77]**	n=1; NA
	Q5 vs Q4; Energy	**560.00 [468.58, 651.42]**	n=1; NA
	Q5 vs Q3; Energy	**638.00 [555.61, 720.39]**	n=1; NA
	Q5 vs Q2; Energy	**751.00 [669.07, 832.93]**	n=1; NA
	Q5 vs Q1; Energy	**1.42 [1.27, 1.57]**	n=1; NA
	Q4 vs Q3; Energy	78.00 [-44.22, 200.22]	n=1; NA
	Q4 vs Q2; Energy	**191.00 [69.10, 312.90]**	n=1; NA
	Q4 vs Q1; Energy	**382.00 [255.65, 508.35]**	n=1; NA
	Q3 vs Q2; Energy	113.00 [-2.29, 228.29]	n=1; NA
	Q3 vs Q1; Energy	**304.00 [184.02, 423.98]**	n=1; NA
	Q2 vs. Q1; Energy	**942.00 [853.59, 1030.41]**	n=1; NA
Any type of milk	Micronutrient	The study reported higher micronutrients intake except for sodium, phosphorus, riboflavin, and selenium in YCM group as compared to other milk consumption. The non-dairy consumers had lower micronutrient and macro-nutrient adequacy except for fibre in children aged 1-2 years ($p < 0.05$)	

**Variety of ASF**			
**Type of Food**	**Outcome**	**Effect Estimate**	**n; Heterogeneity I^2; p-value; GRADE certainty**
**Non-Randomized Interventional Studies**			

Meat porridge vs milk porridge	Height	MD -0.50 [-0.74, -0.26]	n=1; NA
	HAZ	**MD -0.07 [-0.16, 0.02]**	n=1; NA
	Weight	**MD -0.07 [-0.17, 0.03]**	n=1; NA; GRADE: Low
	WAZ	**MD -0.04 [-0.12, 0.04]**	n=1; NA
	WHZ	**MD 0.08 [-0.02, 0.18]**	n=1; NA; GRADE: Low
	Head circumference	**MD -0.05 [-0.12, 0.02]**	n=1; NA; GRADE: Low
	MUAC	MD -0.17 [-0.28, -0.06]	n=1; NA; GRADE: Low
**Observational Studies**			
Not known	3 ASF versus 0 ASF; Stunting	**0.17 [0.16, 0.17]**	n=1; NA; GRADE: Low
	2 ASF versus 0 ASF; Stunting	**0.43 [0.42, 0.44]**	n=1; NA; GRADE: Low
	1 ASF versus 0 ASF; Stunting	0.99 [0.97, 1.01]	n=1; NA; GRADE: Low
Any type of ASF	3 Types versus 1 ASF; Stunting	OR 0.44 [-0.35, 0.54]	2 studies; heterogeneity= I2 99%; Chi2 P <0.00001; GRADE: Very low
	2 ASF versus 1 ASF; Stunting	OR 0.39 [0.31, 0.49]	2 studies; heterogeneity= I^2 100%; Chi^2 P <0.00001; GRADE: Very Low
	3 ASF versus 1 ASF; Stunting	OR 0.17 [0.16, 0.17]	2 studies; heterogeneity= I^2 19%; Chi^2 P 0.27; GRADE: Very Low

S2.9. Forest plots for ASF studies

Forest plot for WAZ score in children given eggs

Study or Subgroup	Greater Amount Mean	Greater Amount SD	Greater Amount Total	Lesser Amount Mean	Lesser Amount SD	Lesser Amount Total	Weight	Mean Difference IV, Random, 95% CI
#21632 Lannotti 2017	-0.34	1.06	75	-0.55	0.85	73	23.2%	0.21 [-0.10, 0.52]
Stewart 2019	-0.61	1.11	290	-0.74	1	305	76.8%	0.13 [-0.04, 0.30]
**Total (95% CI)**			**365**			**378**	**100.0%**	**0.15 [-0.00, 0.30]**

Heterogeneity: Tau² = 0.00; Chi² = 0.20, df = 1 (P = 0.66); I² = 0%

Test for overall effect: Z = 1.95 (P = 0.05)

Mean Difference
IV, Random, 95% CI

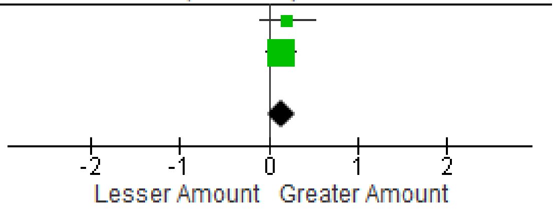

Forest plot for HAZ score in children given eggs

Study or Subgroup	Greater Amount Mean	Greater Amount SD	Greater Amount Total	Lesser Amount Mean	Lesser Amount SD	Lesser Amount Total	Weight	Mean Difference IV, Random, 95% CI
#21632 Lannotti 2017	-1.39	1.35	75	-1.71	1.39	83	9.9%	0.32 [-0.11, 0.75]
Beirut et al. 2021 (1)	-1.74	1.1	137	-1.83	1.1	127	25.6%	0.09 [-0.18, 0.36]
Stewart 2019	-1.08	1.08	290	-1.1	1	305	64.5%	0.02 [-0.15, 0.19]
**Total (95% CI)**			**502**			**515**	**100.0%**	**0.07 [-0.07, 0.20]**

Heterogeneity: Tau² = 0.00; Chi² = 1.68, df = 2 (P = 0.43); I² = 0%

Test for overall effect: Z = 0.99 (P = 0.32)

Mean Difference
IV, Random, 95% CI

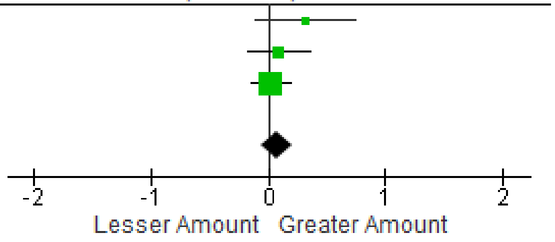

Footnotes

(1) Children aged 9-12 months; Egg powder; RCT; African Region; Follow-up 17 months

Forest plot for WHZ score in children given eggs

Study or Subgroup	Greater Amount Mean	Greater Amount SD	Greater Amount Total	Lesser Amount Mean	Lesser Amount SD	Lesser Amount Total	Weight	Mean Difference IV, Random, 95% CI
#21632 Lannotti 2017	0.45	0.84	75	0.36	0.81	73	21.7%	0.09 [-0.18, 0.36]
Beirut et al. 2021 (1)	-0.02	0.9	137	0.08	0.8	127	32.7%	-0.10 [-0.31, 0.11]
Stewart 2019	-0.3	0.97	290	-0.13	1.04	305	45.6%	-0.17 [-0.33, -0.01]
**Total (95% CI)**			**502**			**505**	**100.0%**	**-0.09 [-0.23, 0.05]**

Heterogeneity: Tau² = 0.00; Chi² = 2.68, df = 2 (P = 0.26); I² = 25%
Test for overall effect: Z = 1.30 (P = 0.19)

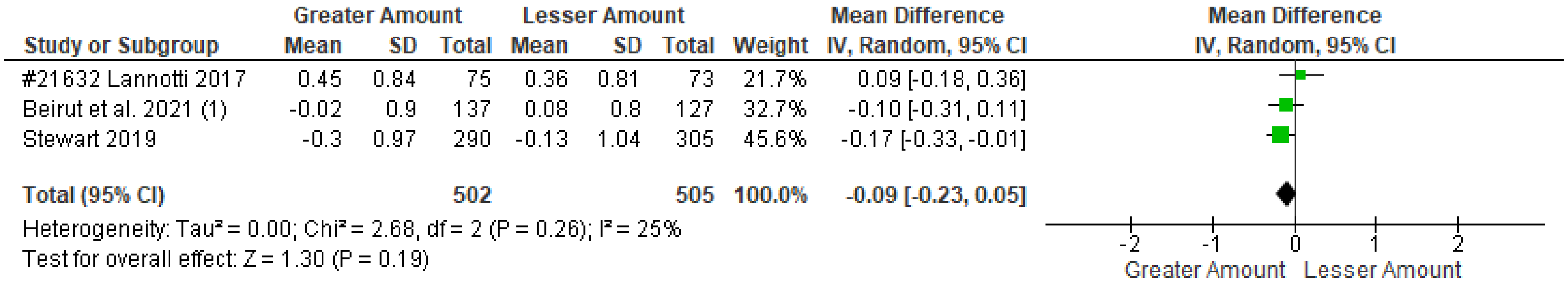

Footnotes
(1) Children aged 9-12 months; Egg powder; RCT; African Region; Follow-up 17 months

Forest plot for stunting in children given eggs/ egg powder

Study or Subgroup	Greater amount Events	Greater amount Total	None Events	None Total	Weight	Risk Ratio M-H, Random, 95% CI
#21632 Lannotti 2017 (1)	21	75	29	73	28.8%	0.70 [0.44, 1.12]
Beirut et al. 2021 (2)	47	137	62	127	71.2%	0.70 [0.52, 0.94]
**Total (95% CI)**		**212**		**200**	**100.0%**	**0.70 [0.55, 0.90]**
Total events	68		91			

Heterogeneity: Tau² = 0.00; Chi² = 0.00, df = 1 (P = 0.99); I² = 0%
Test for overall effect: Z = 2.80 (P = 0.005)

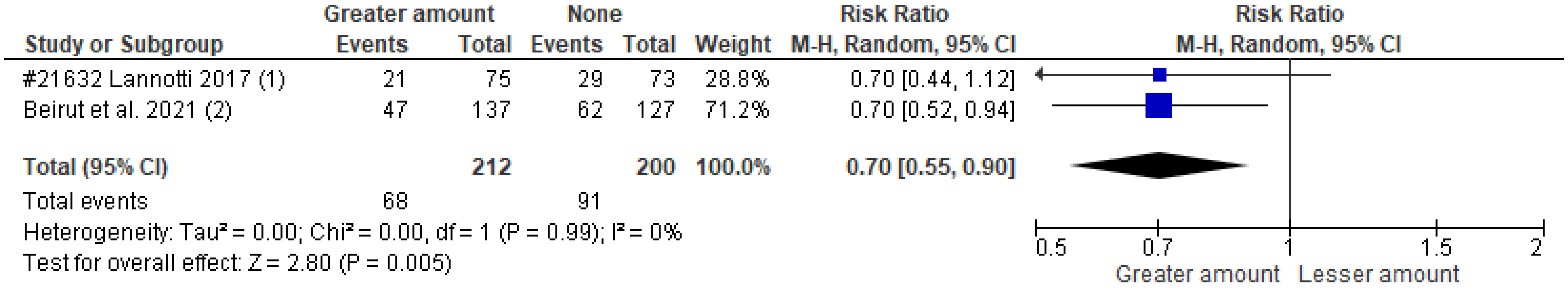

Footnotes
(1) Egg consumption; children aged 6-9.9 months; Follow-up 6 months; African region
(2) Children aged 9-12 months; Egg powder; RCT; African Region; Follow-up 17 months

Forest plot for stunting in children aged 6-23 months for varied ASF

Study or Subgroup	More varied Events	More varied Total	Less Varied Events	Less Varied Total	Weight	Odds Ratio M-H, Random, 95% CI
Heady, 2018 (1)	11086	130432	20996	130432	50.1%	0.48 [0.47, 0.50]
Krasevec 2017 (2)	6467	74548	14553	74548	49.9%	0.39 [0.38, 0.40]
**Total (95% CI)**		**204980**		**204980**	**100.0%**	**0.44 [0.35, 0.54]**
Total events	17553		35549			

Heterogeneity: Tau² = 0.02; Chi² = 109.81, df = 1 (P < 0.00001); I² = 99%
Test for overall effect: Z = 7.83 (P < 0.00001)

Odds Ratio M-H, Random, 95% CI

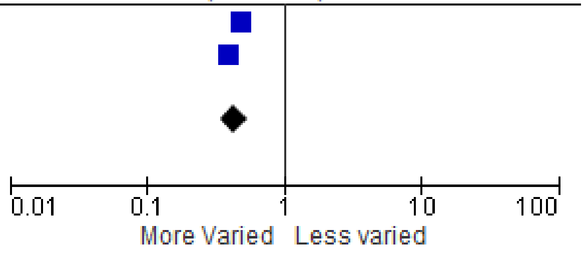

Footnotes
(1) More varied= 3 type of ASF; less varied; 2 type of ASF
(2) More varied= 3 type of ASF; less varied; 2 type of ASF

Forest plot for stunting in children aged 6-23 months for varied ASF

Study or Subgroup	More Varied Events	More Varied Total	Less Varied Events	Less Varied Total	Weight	Odds Ratio M-H, Random, 95% CI
Heady, 2018 (1)	20996	130432	46694	130432	50.0%	0.34 [0.34, 0.35]
Krasevec 2017 (2)	14553	74548	26710	74548	50.0%	0.43 [0.42, 0.44]
**Total (95% CI)**		**204980**		**204980**	**100.0%**	**0.39 [0.31, 0.49]**
Total events	35549		73404			

Heterogeneity: Tau² = 0.03; Chi² = 232.66, df = 1 (P < 0.00001); I² = 100%
Test for overall effect: Z = 8.15 (P < 0.00001)

Odds Ratio M-H, Random, 95% CI

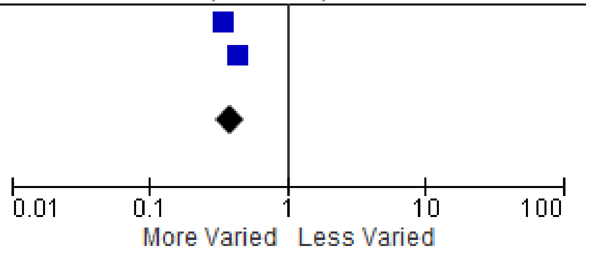

Footnotes
(1) More varied= 2 type of ASF; less varied; 1 type of ASF
(2) More varied= 2 type of ASF; less varied; 1 type of ASF

Forest plot for stunting in children aged 6-23 months for varied ASF

Study or Subgroup	More Varied Events	More Varied Total	Less Varied Events	Less Varied Total	Weight	Odds Ratio M-H, Random, 95% CI
Heady, 2018 (1)	11086	130432	46694	130432	60.8%	0.17 [0.16, 0.17]
Krasevec 2017 (2)	6467	74548	26710	74548	39.2%	0.17 [0.17, 0.18]
**Total (95% CI)**		**204980**		**204980**	**100.0%**	**0.17 [0.16, 0.17]**
Total events	17553		73404			

Heterogeneity: Tau² = 0.00; Chi² = 1.23, df = 1 (P = 0.27); I² = 19%

Test for overall effect: Z = 173.48 (P < 0.00001)

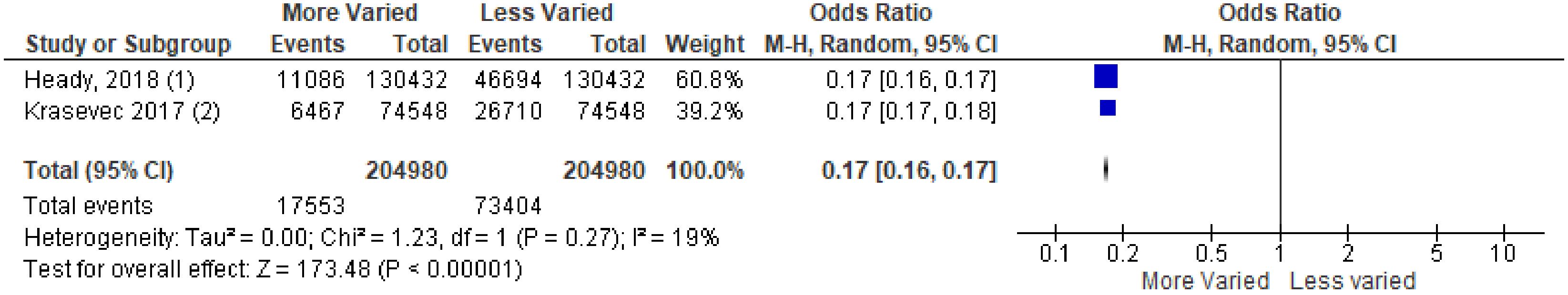

Footnotes

(1) more varied= 3 types of ASF; less varied= 1 type of ASF

(2) more varied= 3 types of ASF; less varied= 1 type of ASF

S2.10. GRADE Assessments for ASF Studies

S2.10.1. Question: Greater frequency of ASF compared to lesser frequency of ASF for 6-23 months (Observational Studies)

Certainty assessment							№ of patients		Effect		Certainty	Importance
№ of studies	Study design	Risk of bias	Inconsistency	Indirectness	Imprecision	Other considerations	greater frequency of ASF	lesser frequency of ASF	Relative (95% CI)	Absolute (95% CI)		
**Stunting in Children Consuming Meat: Assessed by Ahmad et al. 2018; Age at outcomes 6-23 months; Follow-up NA**												
1	observational studies (non-comparative cross-sectional study)	serious [a]	not serious [b]	not serious	very serious [c]	none	11/392 (2.8%)	26/392 (6.6%)	**RR 1.10** (0.61 to 1.96)	**7 more per 1,000** (from 26 fewer to 64 more)	⨁◯◯◯ VERY LOW	CRITICAL
**Stunting in Children Consuming Meat: Assessed by Zhao et al. 2016; Age at outcomes not reported; Follow-up NA**												

Certainty assessment							№ of patients		Effect		Certainty	Importance
**№ of studies**	**Study design**	**Risk of bias**	**Inconsistency**	**Indirectness**	**Imprecision**	**Other considerations**	**greater frequency of ASF**	**lesser frequency of ASF**	**Relative (95% CI)**	**Absolute (95% CI)**		
1	observational studies (cross-sectional study)	very serious [d]	not serious [b]	serious [e]	not serious	none	370/668 (55.4%)	76/139 (54.7%)	**RR 1.01** (0.86 to 1.20)	**5 more per 1,000** (from 77 fewer to 109 more)	⊕◯◯◯ VERY LOW	CRITICAL
**Wasting in Children Consuming Meat: Assessed by Ahmad et al. 2018; Age at outcomes 6-23 months; Follow-up NA**												
1	observational studies (non-comparative cross-sectional study)	serious [a]	not serious [b]	not serious	very serious [f]	none	9/392 (2.3%)	18/392 (4.6%)	**RR 1.28** (0.64 to 2.56)	**13 more per 1,000** (from 17 fewer to 72 more)	⊕◯◯◯ VERY LOW	CRITICAL
**Wasting in Children Consuming Meat: Assessed by Zhao et al. 2016; Age at outcomes not reported; Follow-up NA**												
1	observational studies (cross-sectional study)	very serious [d]	not serious [b]	serious [e]	very serious [f]	none	88/660 (13.3%)	18/136 (13.2%)	**RR 1.01** (0.63 to 1.62)	**1 more per 1,000** (from 49 fewer to 82 more)	⊕◯◯◯ VERY LOW	CRITICAL
**Underweight in Children Consuming Meat: Assessed by Ahmad et al. 2018; Age at outcomes 6-23 months; Follow-up NA**												
1	observational studies (non-comparative cross-sectional study)	serious [a]	not serious [b]	not serious	very serious [g]	none	14/392 (3.6%)	22/392 (5.6%)	**RR 1.65** (0.96 to 2.83)	**36 more per 1,000** (from 2 fewer to 103 more)	⊕◯◯◯ VERY LOW	CRITICAL
**Underweight in Children Consuming Meat: Assessed by Zhao et al. 2016; Age at outcomes not reported**												
1	observational studies (cross-sectional study)	very serious [d]	not serious [b]	serious [e]	very serious [g]	none	269/668 (40.3%)	51/138 (37.0%)	**RR 1.09** (0.86 to 1.38)	**33 more per 1,000** (from 52 fewer to 140 more)	⊕◯◯◯ VERY LOW	CRITICAL

Certainty assessment							№ of patients		Effect		Certainty	Importance
№ of studies	Study design	Risk of bias	Inconsistency	Indirectness	Imprecision	Other considerations	greater frequency of ASF	lesser frequency of ASF	Relative (95% CI)	Absolute (95% CI)		
**Anemia in Children Consuming Meat: Urkin et al. 2007; Follow up 1-2 months after enrollment, children aged 12-months were the study population**												
1	observational studies (non-comparative prospective study)	very serious [h]	not serious [b]	serious [e]	not serious	none			not estimable	not estimable	⊕◯◯◯ VERY LOW	IMPORTANT
**Anemia in Children Consuming Red Meat; Assessed by Silva et al, 2007; children aged 6-12 months were study population; Follow-up NA**												
1	observational studies (cross-sectional study)	very serious [i]	not serious [b]	not serious [j]	serious [f]	none	81/153 (52.9%)	37/52 (71.2%)	**RR 0.74** (0.59 to 0.94)	**185 fewer per 1,000** (from 292 fewer to 43 fewer)	⊕◯◯◯ VERY LOW	IMPORTANT
**Anemia in Children Consuming Organ Meat i.e.. Liver: Assessed by Silva et al, 2007; children aged 6-12 months were study population; Follow-up NA**												
1	observational studies (cross-sectional study)	very serious [i]	not serious [b]	not serious [j]	very serious [c]	none	46/83 (55.4%)	72/122 (59.0%)	**RR 0.94** (0.74 to 1.20)	**35 fewer per 1,000** (from 153 fewer to 118 more)	⊕◯◯◯ VERY LOW	IMPORTANT
**Stunting in children Consuming Eggs: Assessed by Ahmad et al. 2018; Age at outcomes 6-23 months; Follow-up NA**												
1	observational studies (non-comparative cross-sectional study)	serious [a]	not serious [b]	not serious	very serious [g]	none	43/392 (11.0%)	39/392 (9.9%)	**RR 1.14** (0.79 to 1.65)	**14 more per 1,000** (from 21 fewer to 65 more)	⊕◯◯◯ VERY LOW	CRITICAL
**Stunting in Children Consuming Eggs: Assessed by Zhao et al. 2016; Age at outcomes not reported; Follow-up NA**												

Certainty assessment							№ of patients		Effect		Certainty	Importance
**№ of studies**	**Study design**	**Risk of bias**	**Inconsistency**	**Indirectness**	**Imprecision**	**Other considerations**	**greater frequency of ASF**	**lesser frequency of ASF**	**Relative (95% CI)**	**Absolute (95% CI)**		
1	observational studies (cross-sectional study)	very serious [e]	not serious [b]	serious [e]	not serious	none	346/623 (55.5%)	100/182 (54.9%)	**RR 1.01** (0.87 to 1.17)	**5 more per 1,000** (from 71 fewer to 93 more)	⊕◯◯◯ VERY LOW	CRITICAL
**Wasting in Children Consuming Eggs: Assessed by Ahmad et al. 2018; Age at outcomes 6-23 months; Follow-up NA**												
1	observational studies (non-comparative cross-sectional study)	serious [a]	not serious [b]	not serious	very serious [g]	none	34/392 (8.7%)	33/392 (8.4%)	**RR 1.07** (0.70 to 1.62)	**6 more per 1,000** (from 25 fewer to 52 more)	⊕◯◯◯ VERY LOW	CRITICAL
**Wasting in Children Consuming Eggs: Assessed by Zhao et al. 2016; Age at outcomes not reported; Follow-up NA**												
1	observational studies (cross-sectional study)	very serious [e]	not serious [b]	serious [e]	not serious	none	346/625 (55.4%)	100/182 (54.9%)	**RR 1.01** (0.87 to 1.17)	**5 more per 1,000** (from 71 fewer to 93 more)	⊕◯◯◯ VERY LOW	CRITICAL
**Underweight in Children Consuming Eggs: Assessed by Ahmad et al. 2018; Age at outcomes 6-23 months; Follow-up NA**												
1	observational studies (non-comparative cross-sectional study)	serious [a]	not serious [b]	not serious	very serious [g]	none	44/392 (11.2%)	34/392 (8.7%)	**RR 1.34** (0.91 to 1.97)	**29 more per 1,000** (from 8 fewer to 84 more)	⊕◯◯◯ VERY LOW	CRITICAL
**Underweight in Children Consuming Eggs: Assessed by Zhao et al. 2016; Age at outcomes not reported; Follow-up NA**												
1	observational studies (cross-sectional study)	very serious [e]	not serious [b]	serious [e]	serious [k]	none	251/623 (40.3%)	69/181 (38.1%)	**RR 1.06** (0.86 to 1.30)	**23 more per 1,000** (from 53 fewer to 114 more)	⊕◯◯◯ VERY LOW	CRITICAL

Certainty assessment							№ of patients		Effect		Certainty	Importance
№ of studies	Study design	Risk of bias	Inconsistency	Indirectness	Imprecision	Other considerations	greater frequency of ASF	lesser frequency of ASF	Relative (95% CI)	Absolute (95% CI)		
**Stunting in Children Consuming Fish: Assessed by Ahmad et al. 2018; Age at outcomes 6-23 months; Follow-up NA**												
1	observational studies (non-comparative cross-sectional study)	serious [a]	not serious [b]	not serious	very serious [g]	none	63/392 (16.1%)	18/392 (4.6%)	**RR 1.22** (0.77 to 1.91)	**10 more per 1,000** (from 11 fewer to 42 more)	⨁◯◯◯ VERY LOW	CRITICAL
**Wasting in Children Consuming Fish: Assessed by Ahmad et al. 2018; Age at outcomes 6-23 months; Follow-up NA**												
1	observational studies (non-comparative cross-sectional study)	serious [a]	not serious [b]	not serious	serious [k]	none	39/392 (9.9%)	26/392 (6.6%)	**RR 0.52** (0.34 to 0.80)	**32 fewer per 1,000** (from 44 fewer to 13 fewer)	⨁⨁◯◯ LOW	CRITICAL
**Underweight in Children Consuming Fish: Assessed by Ahmad et al. 2018; Age at outcomes 6-23 months; Follow-up NA**												
1	observational studies (non-comparative cross-sectional study)	serious [a]	not serious [b]	not serious	serious [k]	none	54/392 (13.8%)	23/392 (5.9%)	**RR 0.82** (0.54 to 1.23)	**11 fewer per 1,000** (from 27 fewer to 13 more)	⨁⨁◯◯ LOW	CRITICAL

Certainty assessment							№ of patients		Effect		Certainty	Importance
№ of studies	Study design	Risk of bias	Inconsistency	Indirectness	Imprecision	Other considerations	greater frequency of ASF	lesser frequency of ASF	Relative (95% CI)	Absolute (95% CI)		
**Stunting in children consuming any ASF vs no ASF; Assessed by Darapheak et al. (2013); children aged 12-59 months; Follow-up NA**												

Certainty assessment							№ of patients		Effect		Certainty	Importance
№ of studies	Study design	Risk of bias	Inconsistency	Indirectness	Imprecision	Other considerations	greater frequency of ASF	lesser frequency of ASF	Relative (95% CI)	Absolute (95% CI)		
1	observational studies	serious[a]	not serious[b]	serious[c]	not serious	none	710/1578 (45.0%)	188/329 (57.1%)	**RR 0.80** (0.71 to 0.89)	**114 fewer per 1,000** (from 166 fewer to 63 fewer)	⨁◯◯◯ LOW	CRITICAL
**Wasting in children consuming ASF vs no ASF; Assessed by Darapheak et al. (2013); children aged 12-59 months; Follow-up NA**												
1	observational studies	serious[a]	not serious[b]	serious[c]	not serious	none	118/1578 (7.5%)	22/329 (6.7%)	**RR 1.12** (0.72 to 1.74)	**8 more per 1,000** (from 19 fewer to 49 more)	⨁◯◯◯ LOW	CRITICAL
**Underweight in children consuming ASF vs no ASF; Assessed by Darapheak et al. (2013); children aged 12-59 months; Follow-up NA**												
1	observational studies	serious[a]	not serious[b]	serious[c]	not serious	none	432/1578 (27.4%)	116/329 (35.3%)	**RR 0.78** (0.66 to 0.92)	**78 fewer per 1,000** (from 120 fewer to 28 fewer)	⨁◯◯◯ LOW	CRITICAL
**Stunting in children consuming milk products; Assessed by Darapheak et al. (2013); children aged 12-59 months; Follow-up NA**												
1	observational studies	serious[a]	not serious[b]	serious[c]	not serious	none	67/192 (34.9%)	827/1713 (48.3%)	**RR 0.72** (0.59 to 0.88)	**135 fewer per 1,000** (from 198 fewer to 58 fewer)	⨁◯◯◯ LOW	CRITICAL

Certainty assessment							№ of patients		Effect		Certainty	Importance
№ of studies	Study design	Risk of bias	Inconsistency	Indirectness	Imprecision	Other considerations	greater frequency of ASF	lesser frequency of ASF	Relative (95% CI)	Absolute (95% CI)		
**Wasting in children consuming milk products; Assessed by Darapheak et al. (2013); children aged 12-59 months; Follow-up NA**												
1	observational studies	serious[a]	not serious[b]	serious[c]	not serious	none	9/192 (4.7%)	132/1713 (7.7%)	**RR 0.61** (0.31 to 1.18)	**30 fewer per 1,000** (from 53 fewer to 14 more)	⨁◯◯◯ LOW	CRITICAL
**Underweight in children consuming milk products; Assessed by Darapheak et al. (2013); children aged 12-59 months; Follow-up NA**												
1	observational studies	serious[a]	not serious[b]	serious[c]	not serious	none	44/192 (22.9%)	504/1713 (29.4%)	**RR 0.78** (0.59 to 1.02)	**65 fewer per 1,000** (from 121 fewer to 6 more)	⨁◯◯◯ LOW	CRITICAL

CI: Confidence interval; **RR:** Risk ratio

Explanations

a. Downgraded once- The use of observational study design increases the risk of biases in the study.
b. It is a single study. Thus, inconsistency cannot be determined.
c. Downgraded twice. The sample size is very low and the number of events are also < 300.
d. Downgraded twice- Lacks the mentioning of sample size justification and the exposures were also not mentioned clearly.
e. Downgraded once. No proper sample size justification and representativeness
f. Downgraded twice. Number of events < 300.
g. Downgraded twice. CI is very broad and the number of events are < 300
h. Downgraded twice- This study lacks many details on methodology making it very hard to determine whether there is internal validity. In addition, the study is described as an interventional study when it actually appears to be observational in nature. Food exposures are assessed by a questionnaire and blood samples tested for anemia outcome/ iron stores. There is no assigning of food as an intervention but rather observation of what is eaten by questionnaire.

i. Downgraded twice- Very limited detail in the methodology makes it challenging to tell if there was internal validity of study. No sample size justification provided or mention of loss to follow up or participation rate of eligible participants. Small sample size with no information provided for baseline characteristics of study sample of children.
j. Lack of sample size justification and loss to follow-up of the participants from the baseline.
k. Downgraded once. Number of events <300

S2.10.2. Question: Greater amount of ASF compared to Lesser amount for 6-23 months (RCTs)

Certainty assessment							№ of patients		Effect		Certainty	Importance
№ of studies	Study design	Risk of bias	Inconsistency	Indirectness	Imprecision	Other considerations	greater amount of ASF	Lesser amount	Relative (95% CI)	Absolute (95% CI)		
**Change in Height in Children Consuming Meat (assessed with: Higher MD indicates improvement.): Assessed by Engelmann et al. 1998; children aged 8-10 months were study population; Follow-up at 8 and 10 months after intervention**												
1	randomised trials	serious [a]	not serious [b]	serious [c]	serious [d]	none	21	20	-	MD **0.1 SD lower** (1.77 lower to 1.57 higher)	⨁◯◯◯ VERY LOW	CRITICAL
**Change in Weight in Children Consuming Meat: (assessed with: Higher MD indicates improvement.): Assessed by Engelmann et al. 1998; children aged 8-10 months were study population; Follow-up at 8 and 10 months after intervention**												
1	randomised trials	serious [a]	not serious [b]	serious [c]	serious [e]	none	21	20	-	MD **0.08 higher** (0.53 lower to 0.7 higher)	⨁◯◯◯ VERY LOW	CRITICAL
**Change in Triceps Skinfolds in Children Consuming Meat: (assessed with: Higher MD indicates improvement.): Assessed by Engelmann et al. 1998; children aged 8-10 months were study population; Follow-up at 8 and 10 months after intervention**												
1	randomised trials	serious [a]	not serious [b]	serious [c]	serious [d]	none	21	20	-	MD **0.1 lower** (5.39 lower to 5.19 higher)	⨁◯◯◯ VERY LOW	CRITICAL
**Hemoglobin in Children Consuming Meat: (assessed with: Higher MD indicates improvement.): Assessed by Engelmann et al. 1998; children aged 8-10 months were study population; Follow-up at 8 and 10 months after intervention**												
1	randomised trials	serious [a]	not serious [b]	serious [c]	not serious	none	21	20	-	SMD **0.21 SD higher** (0.4 lower to 0.83 higher)	⨁⨁◯◯ LOW	IMPORTANT
**Stunting in Children Consuming Lyophilized Beef: Assessed by Kreb et al. 2012; children aged 6-18 months were the study population; Follow-up at 6, 9, 12 and 18 months**												

Certainty assessment							№ of patients		Effect		Certainty	Importance
№ of studies	Study design	Risk of bias	Inconsistency	Indirectness	Imprecision	Other considerations	greater amount of ASF	Lesser amount	Relative (95% CI)	Absolute (95% CI)		
1	randomised trials	serious [f]	not serious [b]	serious [g]	serious [e]	none	184/532 (34.6%)	179/530 (33.8%)	**RR 1.02** (0.87 to 1.21)	**7 more per 1,000** (from 44 fewer to 71 more)	⨁◯◯◯ VERY LOW	CRITICAL
**Wasting in Children Consuming Lyophilized Beef: Assessed by Kreb et al. 2012; children aged 6-18 months were the study population; Follow-up at 6, 9, 12 and 18 months**												
1	randomised trials	serious [f]	not serious [b]	serious [g]	very serious [d,e]	none	38/532 (7.1%)	54/530 (10.2%)	**RR 0.70** (0.47 to 1.04)	**31 fewer per 1,000** (from 54 fewer to 4 more)	⨁◯◯◯ VERY LOW	CRITICAL
**WAZ in Children Consuming Lyophilized Beef (assessed with: Higher MD indicates improvement.) : Assessed by Kreb et al. 2012; children aged 6-18 months were the study population; Follow-up at 6, 9, 12 and 18 months**												
1	randomised trials	serious [f]	not serious [b]	serious [g]	not serious	none	532	530	-	MD **0.02 lower** (0.16 lower to 0.12 higher)	⨁⨁◯◯ LOW	CRITICAL
**HAZ in Children Consuming Lyophilized Beef (assessed with: Higher MD indicates improvement.) : Assessed by Kreb et al. 2012; children aged 6-18 months were the study population; Follow-up at 6, 9, 12 and 18 months**												
1	randomised trials	serious [f]	not serious [b]	serious [g]	not serious	none	532	530	-	MD **0.06 lower** (0.23 lower to 0.11 higher)	⨁⨁◯◯ LOW	CRITICAL
**WHZ in Children Consuming Lyophilized Beef (assessed with: Higher MD indicates improvement.) : Assessed by Kreb et al. 2012; children aged 6-18 months were the study population; Follow-up at 6, 9, 12 and 18 months**												
1	randomised trials	serious [f]	not serious [b]	serious [g]	not serious	none	532	530	-	MD **0.01 SD higher** (1.04 lower to 0.16 higher)	⨁⨁◯◯ LOW	CRITICAL
**HCAZ in Children Consuming Lyophilized Beef (assessed with: Higher MD indicates improvement.) : Assessed by Kreb et al. 2012; children aged 6-18 months were the study population; Follow-up at 6, 9, 12 and 18 months**												
1	randomised trials	serious [f]	not serious [b]	serious [g]	not serious	none	532	530	-	MD **0.07 lower** (0.2 lower to 0.06 higher)	⨁⨁◯◯ LOW	CRITICAL
**WAZ in Children Consuming Pork (assessed with: Higher MD indicates improvement.): Assessed by Tang et al, 2014; children aged 6-18 months were the population; Follow-up at 6, 7, 9, 12, 15 and 18 months of age**												

Certainty assessment							№ of patients		Effect		Certainty	Importance
№ of studies	Study design	Risk of bias	Inconsistency	Indirectness	Imprecision	Other considerations	greater amount of ASF	Lesser amount	Relative (95% CI)	Absolute (95% CI)		
1	randomised trials	serious [h]	not serious [b]	not serious	not serious	none	462	856	-	MD **0.08 higher** (0.01 higher to 0.15 higher)	⊕⊕⊕◯ MODERATE	CRITICAL
**HAZ in Children consuming Pork (assessed with: Higher MD indicates improvement.): Assessed by Tang et al, 2014; children aged 6-18 months were the population; Follow-up at 6, 7, 9, 12, 15 and 18 months of age**												
1	randomised trials	serious [h]	not serious [b]	not serious	not serious	none	462	856	-	MD **0.11 higher** (0.03 higher to 0.19 higher)	⊕⊕⊕◯ MODERATE	CRITICAL
**WHZ in Children Consuming Pork (assessed with: Higher MD indicates improvement.): Assessed by Tang et al, 2014; children aged 6-18 months were the population; Follow-up at 6, 7, 9, 12, 15 and 18 months of age**												
1	randomised trials	serious [h]	not serious [b]	not serious	not serious	none	462	856	-	MD **0.03 higher** (0.06 lower to 0.12 higher)	⊕⊕⊕◯ MODERATE	CRITICAL
**HCAZ in Children Consuming Pork ((assessed with: Higher MD indicates improvement.): Assessed by Tang et al, 2014; children aged 6-18 months were the population; Follow-up at 6, 7, 9, 12, 15 and 18 months of age**												
1	randomised trials	serious [h]	not serious [b]	not serious	not serious	none	462	856	-	MD **0.01 lower** (0.07 lower to 0.05 higher)	⊕⊕⊕◯ MODERATE	CRITICAL
**Change in Height in Children Consuming Pork (assessed with: Higher MD indicates improvement.): Assessed by Tang et al, 2014; children aged 6-18 months were the population; Follow-up at 6, 7, 9, 12, 15 and 18 months of age**												
1	randomised trials	serious [h]	not serious [b]	not serious	not serious	none	462	856	-	MD **0.26 higher** (0.05 higher to 0.47 higher)	⊕⊕⊕◯ MODERATE	CRITICAL
**Change in Weight in Children Consuming Pork (assessed with: Higher MD indicates improvement.): Assessed by Tang et al, 2014; children aged 6-18 months were the population; Follow-up at 6, 7, 9, 12, 15 and 18 months of age**												
1	randomised trials	serious [h]	not serious [b]	not serious	not serious	none	462	856	-	MD **0.07 SD higher** (0 to 0.14 higher)	⊕⊕⊕◯ MODERATE	CRITICAL
**Change in Head Circumference in Children Consuming Pork (Tang) ((assessed with: Higher MD indicates improvement.): Assessed by Tang et al, 2014; children aged 6-18 months were the population; Follow-up at 6, 7, 9, 12, 15 and 18 months of age**												

Certainty assessment							№ of patients		Effect		Certainty	Importance
№ of studies	Study design	Risk of bias	Inconsistency	Indirectness	Imprecision	Other considerations	greater amount of ASF	Lesser amount	Relative (95% CI)	Absolute (95% CI)		
1	randomised trials	serious [h]	not serious [b]	not serious	not serious	none	462	856	-	MD **2.98 higher** (2.9 higher to 3.06 higher)	⊕⊕⊕◯ MODERATE	CRITICAL
**HCAZ in Children Consuming Eggs (assessed with: Higher MD indicates improvement.): Assessed by Stewart et al. 2019; children aged 6-9.9 months were the study population; Follow-up 6 months**												
1	randomised trials	serious [i]	not serious [b]	serious [j]	not serious	none	290	305	-	MD **0.23 higher** (0.05 higher to 0.41 higher)	⊕⊕◯◯ LOW	CRITICAL
**Stunting in Children Consuming Eggs: Assessed by Ianotti et al. 2017; children aged 6-9 months were the study population; Follow-up 6 months**												
1	randomised trials	serious [i]	not serious [b]	serious [k]	very serious [d,e]	none	21/75 (28.0%)	29/73 (39.7%)	**RR 0.70** (0.44 to 1.12)	**119 fewer per 1,000** (from 222 fewer to 48 more)	⊕◯◯◯ VERY LOW	CRITICAL
**Anemia in Children Consuming Eggs: Assessed by Makrides et al, 2002; children aged 6-12 months were the study population; Follow-up at 6,9 and 12 months**												
1	randomised trials	not serious	not serious [b]	not serious	very serious [d,e]	none	3/44 (6.8%)	2/23 (8.7%)	**RR 0.78** (0.14 to 4.36)	**19 fewer per 1,000** (from 75 fewer to 292 more)	⊕⊕◯◯ LOW	IMPORTANT
**Hemoglobin in Children Consuming Eggs (assessed with: Higher MD indicates improvement.): Assessed by Makrides et al, 2002; children aged 6-12 months were the study population; Follow-up at 6,9 and 12 months**												
1	randomised trials	not serious	not serious [b]	not serious	serious [e]	none	44	23	-	SMD **0.2 SD higher** (0.31 lower to 0.71 higher)	⊕⊕⊕◯ MODERATE	IMPORTANT
**Change in Triceps Skinfold in Children Consuming Skimmed Milk (assessed with: Higher MD indicates improvement.): Assessed by Skau et al, 2015; children aged 6-15 months were study population; Follow-up at 9 months of intervention [15 months]**												
1	randomised trials	not serious [l]	not serious [b]	not serious	serious [e]	none	106	102	-	MD **0.1 lower** (0.38 lower to 0.18 higher)	⊕⊕⊕◯ MODERATE	CRITICAL
**Change in Head Circumference in Children Consuming Skimmed Milk (assessed with: Higher MD indicates improvement.): Assessed by Skau et al, 2015; children aged 6-15 months were study population; Follow-up at 9 months of intervention [15 months]**												

Certainty assessment							№ of patients		Effect		Certainty	Importance
№ of studies	Study design	Risk of bias	Inconsistency	Indirectness	Imprecision	Other considerations	greater amount of ASF	Lesser amount	Relative (95% CI)	Absolute (95% CI)		
1	randomised trials	not serious [l]	not serious [b]	not serious	serious [e]	none	106	102	-	MD **0.4 lower** (0.76 lower to 0.04 higher)	⊕⊕⊕◯ MODERATE	CRITICAL
**Change in Triceps Skinfold in Children Consuming Fish and Concoction (assessed with: Higher MD indicates improvement.): Assessed by Skau et al, 2015; children aged 6-15 months were study population; Follow-up at 9 months of intervention [15 months]**												
1	randomised trials	not serious [l]	not serious [b]	not serious	serious [e]	none	85	93	-	MD **0.2 lower** (0.48 lower to 0.08 higher)	⊕⊕⊕◯ MODERATE	CRITICAL
**Change in Head Circumference in Children Consuming Fish and Concoction (assessed with: Higher MD indicates improvement.): Assessed by Skau et al, 2015; children aged 6-15 months were study population; Follow-up at 9 months of intervention [15 months]**												
1	randomised trials	not serious [l]	not serious [b]	not serious	serious [e]	none	85	93	-	MD **0.1 lower** (0.52 lower to 0.32 higher)	⊕⊕⊕◯ MODERATE	CRITICAL
**Hemoglobin in Children Consuming Fish and Concoction (assessed with: Higher MD indicates improvement.): Assessed by Skau et al, 2015; children aged 6-15 months were study population; Follow-up at 9 months of intervention [15 months]**												
1	randomised trials	not serious [l]	not serious [b]	not serious	serious [e]	none	69	73	-	SMD **0.28 SD lower** (0.58 lower to 0.02 higher)	⊕⊕⊕◯ MODERATE	IMPORTANT
**Stunting in Children Consuming Caterpillars: Assessed by Bauserman et al. 2015; children aged 6-18 months were the study population; Follow-up at 9, 12 and 18 months**												
1	randomised trials	serious [m]	not serious [b]	serious [n]	very serious [d,e]	none	54/81 (66.7%)	58/82 (70.7%)	**RR 0.94** (0.77 to 1.16)	**42 fewer per 1,000** (from 163 fewer to 113 more)	⊕◯◯◯ VERY LOW	CRITICAL
**Wasting in Children Consuming Caterpillar: Assessed by Bauserman et al. 2015; children aged 6-18 months were the study population; Follow-up at 9, 12 and 18 months**												
1	randomised trials	serious [m]	not serious [b]	serious [n]	very serious [d,e]	none	6/80 (7.5%)	8/80 (10.0%)	**RR 0.75** (0.27 to 2.06)	**25 fewer per 1,000** (from 73 fewer to 106 more)	⊕◯◯◯ VERY LOW	CRITICAL
**WAZ in Children Consuming Caterpillar (assessed with: Higher MD indicates improvement.): Assessed by Bauserman et al. 2015; children aged 6-18 months were the study population; Follow-up at 9, 12 and 18 months**												

Certainty assessment							№ of patients		Effect		Certainty	Importance
№ of studies	Study design	Risk of bias	Inconsistency	Indirectness	Imprecision	Other considerations	greater amount of ASF	Lesser amount	Relative (95% CI)	Absolute (95% CI)		
1	randomised trials	serious [m]	not serious [b]	serious [n]	very serious [d,e]	none	80	82	-	MD **0.2 lower** (0.57 lower to 0.17 higher)	⨁◯◯◯ VERY LOW	CRITICAL
**HAZ in Children Consuming Caterpillar (assessed with: Higher MD indicates improvement.): Assessed by Bauserman et al. 2015; children aged 6-18 months were the study population; Follow-up at 9, 12 and 18 months**												
1	randomised trials	serious [m]	not serious [b]	serious [n]	serious [e]	none	81	82	-	MD **0.1 higher** (0.35 lower to 0.55 higher)	⨁◯◯◯ VERY LOW	CRITICAL
**WHZ in Children Consuming Caterpillar (assessed with: Higher MD indicates improvement.): Assessed by Bauserman et al. 2015; children aged 6-18 months were the study population; Follow-up at 9, 12 and 18 months**												
1	randomised trials	serious [m]	not serious [b]	serious [n]	serious [e]	none	79	80	-	MD **0.2 lower** (0.62 lower to 0.22 higher)	⨁◯◯◯ VERY LOW	CRITICAL
**Anemia in Children Consuming Caterpillar: Assessed by Bauserman et al. 2015; children aged 6-18 months were the study population; Follow-up at 18 months**												
1	randomised trials	serious [m]	not serious [b]	serious [n]	serious [e]	none	20/77 (26.0%)	32/64 (50.0%)	**RR 0.52** (0.33 to 0.81)	**240 fewer per 1,000** (from 335 fewer to 95 fewer)	⨁◯◯◯ VERY LOW	IMPORTANT
**Hemoglobin in Children Consuming Caterpillar Assessed by Bauserman et al. 2015; children aged 6-18 months were the study population; Follow-up at 18 months**												
1	randomised trials	serious [m]	not serious [b]	serious [n]	serious [e]	none	77	64	-	SMD **0.35 SD lower** (0.02 higher to 0.69 higher)	⨁◯◯◯ VERY LOW	IMPORTANT

CI: Confidence interval; **MD:** Mean difference; **SMD:** Standardised mean difference; **RR:** Risk ratio

Explanations

a. Downgraded once- Some major concerns over bias that may have arose from using different blood collection methods. Venous blood vs capillary blood have been shown previously to have significantly different levels of hemoglobin and serum ferritin levels.

However, the number of infants who received venipuncture vs finger pricks was not significantly different (p=0.9) between the two intervention groups.
b. It is a single study. Thus, inconsistency cannot be determined.
c. Downgraded once. Inability to generalize the findings because of low sample size.
d. Downgraded once. Very broad Confidence interval
e. Downgraded once. Number of events < 300
f. Downgraded once- Allocation sequence not concealed and thus increases the likelihood of bias
g. Downgraded once. The external validity is not guaranteed since the sample size is low and the risk of bias related to poor randomisation reduces the applicability.
h. Downgraded once- Study participants were randomized to an intervention arm. There is no mention of concealment
i. Downgraded once- Lack of blinding in intervention allocation.
j. Downgraded once. The sample size is appropriate. However, lack of blinding is one factor that affects the applicability of the research findings.
k. Downgraded once. The sample size is very low to make conclusions for the entire population.
l. Participants seem to have been blinded as the food packages were identical. However, one study staff member who was responsible for distribution knew intervention type in order to distribute food appropriately. Does not appear to be any deviations from intented intervention.
m. Downgraded once- No mention of blinding towards participants or those delivering the caterpillar cereal to participants.
n. Downgraded once. Lost an undesirably high percentage of infants to follow-up because many of the participants relocated and therefore outcome data were incomplete.
n. Downgraded once. Lost an undesirably high percentage of infants to follow-up because many of the participants relocated and therefore outcome data were incomplete.

S2.10.3. Question: Greater amount of ASF compared to lesser amount of ASF for children aged 6-23 months (meta-analysis GRADE Certainty)

Certainty assessment							№ of patients		Effect		Certainty	Importance
№ of studies	Study design	Risk of bias	Inconsistency	Indirectness	Imprecision	Other considerations	greater amount of ASF	lesser amount of ASF	Relative (95% CI)	Absolute (95% CI)		
**WAZ (assessed with: Higher MD indicates improvement.): Assessed by Ianotti et al. 2017 and Stewart et al. 2019; children aged 6-9 months; Follow-up 6 months**												

Certainty assessment							№ of patients		Effect		Certainty	Importance
№ of studies	Study design	Risk of bias	Inconsistency	Indirectness	Imprecision	Other considerations	greater amount of ASF	lesser amount of ASF	Relative (95% CI)	Absolute (95% CI)		
2	randomised trials	serious [a]	not serious	not serious	not serious	none	365	378	-	MD **0.15 higher** (0 to 0.3 higher)	⊕⊕⊕◯ MODERATE	CRITICAL
**HAZ (assessed with: Higher MD indicates improvement.): Assessed by Ianotti et al. 2017 and Stewart et al. 2019; children aged 6-9 months; Follow-up 6 months**												
2	randomised trials	serious [a]	serious [b]	not serious	not serious	none	365	388	-	MD **0.06 higher** (0.1 lower to 0.22 higher)	⊕⊕◯◯ LOW	CRITICAL
**WHZ (assessed with: Higher MD indicates improvement.): Assessed by Ianotti et al. 2017 and Stewart et al. 2019; children aged 6-9 months; Follow-up 6 months**												
2	randomised trials	serious [a]	very serious [c]	not serious	not serious	none	365	378	-	MD **0.06 lower** (0.31 lower to 0.19 higher)	⊕◯◯◯ VERY LOW	CRITICAL

CI: Confidence interval; **MD:** Mean difference

Explanations

a. Downgraded once- Both the studies had some concerns in regards to the blinding.

b. Downgraded once. The studies had the heterogeneity which can be seen by I2 that is more than 30%% and the p-value which is more than 0.10.

c. Downgraded twice. The studies had the heterogeneity of 63% which can be seen by I2 which is more than 30% and the p-value which is 0.10.

S2.10.4. Question: Greater amount compared to lesser amount ASF for 6-23 months (Observational)

Certainty assessment							№ of patients		Effect		Certainty	Importance
№ of studies	Study design	Risk of bias	Inconsistency	Indirectness	Imprecision	Other considerations	greater amount	lesser amount ASF	Relative (95% CI)	Absolute (95% CI)		
**Hemoglobin in Children Consuming Cow Milk: Assessed by Thorsdottir et al. 2003; children aged 0-12 months were the study population; Follow-up at 9-12 months**												
1	observational studies (Observational cohort longitudinal study)	very serious [a]	not serious [b]	serious [c]	very serious [d]		17	80	-	**0** (0 to 0)	⨁◯◯◯ VERY LOW	IMPORTANT

CI: Confidence interval

Explanations

a. Downgraded twice- Lacked sample size justification, and power description

b. It is a single study. Thus, inconsistency cannot be determined.

c. confounders not taken into consideration, nor identified.

d. No absolute values can be calculated and the number of events are < 300

S2.10.5. Question: More varied compared to Less varied in Children aged 6-23 months (non-RCT)

Certainty assessment							№ of patients		Effect		Certainty	Importance
№ of studies	Study design	Risk of bias	Inconsistency	Indirectness	Imprecision	Other considerations	More varied	Less varied	Relative (95% CI)	Absolute (95% CI)		
**HAZ in children meat porridge vs milk porridge (assessed with: Higher MD indicates improvement); Assessed by Long et al. (2012) in children aged 11-40 months; Follow up 5 months**												
1	Non-randomised trials	not serious[a]	not serious[b]	serious[c]	serious[d]	none	81	97	-	MD **0.07 SD lower** (0.16 lower to 0.02 higher)	⨁⨁◯◯ Low	CRITICAL
**WAZ in children consuming meat porridge vs milk porridge (assessed with: Higher MD indicates improvement); Assessed by Long et al. (2012) in children aged 11-40 months; Follow up 5 months**												
1	Non-randomised trials	not serious[a]	not serious[b]	serious[c]	serious[d]	none	81	97	-	MD **0.04 SD lower** (0.12 lower to 0.04 higher)	⨁⨁◯◯ Low	CRITICAL
**WHZ in children consuming meat porridge vs milk porridge (assessed with: Higher MD indicates improvement); Assessed by Long et al. (2012) in children aged 11-40 months; Follow up 5 months**												

Certainty assessment							№ of patients		Effect		Certainty	Importance
№ of studies	Study design	Risk of bias	Inconsistency	Indirectness	Imprecision	Other considerations	More varied	Less varied	Relative (95% CI)	Absolute (95% CI)		
1	Non-randomised trials	not serious[a]	not serious[b]	serious[c]	serious[d]	none	81	97	-	MD **0.08 SD higher** (0.02 lower to 0.18 higher)	⊕⊕◯◯ Low	CRITICAL
**Head Circumference in children consuming meat porridge vs milk porridge (assessed with: Higher MD indicates improvement); Assessed by Long et al. (2012) in children aged 11-40 months; Follow up 5 months**												
1	Non-randomised trials	not serious[a]	not serious[b]	serious[c]	serious[d]	none	81	97	-	MD **0.05 SD lower** (0.12 lower to 0.02 higher)	⊕⊕◯◯ Low	CRITICAL

CI: confidence interval; **SMD:** standardised mean difference

Explanations

a. The participants were recruited from similar population and were divided into groups through the process of randomization

b. Since it is a single study, the inconsistency cannot be determined

c. Downgraded once: There are a few concerns around the representativeness of the sample.

d. Downgraded once: The sample size in each of the groups is <100 which directly affects the precision of the study

S2.10.6. Question: More varied ASF compared to less varied ASF for 6-23 months (observational studies)

Certainty assessment							№ of patients		Effect		Certainty	Importance
№ of studies	Study design	Risk of bias	Inconsistency	Indirectness	Imprecision	Other considerations	more varied ASF	less varied ASF	Relative (95% CI)	Absolute (95% CI)		
**Stunting in Children Consuming Milk/ Milk Products: Assessed by Marinda et al. 2018; children aged 6-59 months were the study population; Follow-up NA**												
1	observational studies (non-comparative cross-sectional study)	serious [d]	not serious [b]	not serious	not serious	none			not estimable		⊕⊕⊕◯ MOERATE	CRITICAL

CI: Confidence interval; **RR:** Risk ratio

Explanations
a. Downgraded once- All the participants were not selected from the same population increasing the risk of bias
b. It is a single study. Thus, inconsistency cannot be determined.
c. Downgraded once. The sample was different and not consistent; thus, it is hard to look for the sample representativeness.
d. Downgraded once- Funding for this study was provided to WorldFish by the German Federal Ministry for Economic Cooperation and Devel. Wondering if there could be some bias or advertising towards consuming fish based on the funder?

S2.10.7. Question: More varied compared to less varied for 6-23 months (meta-analysis)

Certainty assessment							№ of patients		Effect		Certainty	Importance
№ of studies	Study design	Risk of bias	Inconsistency	Indirectness	Imprecision	Other considerations	more varied	less varied	Relative (95% CI)	Absolute (95% CI)		
**Stunting (assessed with: 3 types of ASF Vs 2 types of ASF); Assessed by Krasevac et al. (2017) and Heady wt al. (2018); children aged 6-23 months**												
2	observational studies	very serious[a]	not serious	serious[b]	serious[c]	all plausible residual confounding would reduce the demonstrated effect	17553/204980 (8.6%)	35549/204980 (17.3%)	**OR 0.44** (0.35 to 0.54)	**89 fewer per 1,000** (from 105 fewer to 72 fewer)	⨁◯◯◯ Very low	
**Stunting (assessed with: 2 types of ASF Vs 1 type of ASF); Assessed by Krasevac et al. (2017) and Heady wt al. (2018); children aged 6-23 months**												
2	observational studies	very serious[a]	not serious	serious[b]	serious[c]	all plausible residual confounding would reduce the demonstrated effect	35549/204980 (17.3%)	73404/204980 (35.8%)	**OR 0.39** (0.31 to 0.49)	**179 fewer per 1,000** (from 211 fewer to 143 fewer)	⨁◯◯◯ Very low	
**Stunting (assessed with: 3 types of ASF Vs 1 type of ASF); Assessed by Krasevac et al. (2017) and Heady wt al. (2018); children aged 6-23 months**												
2	observational studies	very serious[a]	not serious	serious[b]	serious[c]	all plausible residual confounding would reduce the demonstrated effect	17553/204980 (8.6%)	73404/204980 (35.8%)	**OR 0.17** (0.16 to 0.17)	**271 fewer per 1,000** (from 276 fewer to 271 fewer)	⨁◯◯◯ Very low	

CI: confidence interval; **OR:** odds ratio

Explanations
a. Downgraded twice= The use of cross-sectional study design makes the likelihood of risk of bias higher. In addition to this, the authors have not clearly stated the recruitment, and not all participants were selected from the same population

b. Downgraded once= The sample was from different areas, and it is hard to look for sample representativeness.
c. Downgraded once= Funding for this study was provided to WorldFish by the German Federal Ministry for Economic Cooperation and Devel. Wondering if there could be some bias or advertising towards consuming fish based on the funder?
