# Supplementary material for: Dietary Strategies for Complementary Feeding between 6 and 24 Months of Age: The Evidence
Source: Nutrients. 2023 Jul 5;15(13):3041. doi: 10.3390/nu15133041 (PMC10346638; doi:10.3390/nu15133041)
Supplement: Supplementary file 1 [file nutrients-15-03041-s001.zip › Supplementary File S3.pdf]

SUPPLEMENTARY DOCUMENT S3 – Impact of Infant and Young Child Feeding (IYCF) Nutrition Interventions on Growth and Mortality in Low- and Middle-Income Countries: Systematic Review (unpublished)

Authors: Zahra Ali Padhani, Komal Abdul Rahim, Ana Ali, and Zohra Lassi

Table of Contents

S3.1. Objective ***2***

S3.2. Methods ***2***

S3.3. Prisma Diagram ***4***

S3.4. List of included studies ***5***

S3.5. GRADE assessments ***14***

S3.6. Forest Plots ***33***

Comparison 1: Complementary food provision versus control ***33***

Comparison 2: Complementary food education versus control ***54***

Comparison 3: Complementary food provision versus control (subgroup by food security status) ***65***

Comparison 4: Complementary food education versus control (subgroup by food security status) ***78***

Comparison 5: Complementary food education versus control (among malnourished children) ***89***

S3.1. Objective

To assess the effectiveness of interventions to promote appropriate complementary feeding (education or provision of complementary food) and improve child health and nutritional status in children aged 6-24 months.

S3.2. Methods

S3.2.1. Criteria for considering studies for this review

We included primary studies, including large-scale program evaluations to assess the efficacy and/or effectiveness of interventions. We included randomized controlled trials (RCTs) randomized at the individual or cluster level (i.e., in cluster randomized controlled trial), cross-over study designs. We also included non-randomized studies such as quasi-experimental designs, including controlled before-after studies (CBA), interrupted time series (ITS) studies, natural experiments and regression discontinuity designs. Non-controlled pre-post studies were not included.

Our target population was healthy children aged 6-24 months living in low- and middle-income countries (LMICs). We included studies in which the intervention arm was compared with a control/placebo arm or if both intervention and control arms received a similar secondary intervention (i.e., CF+cash vs. cash). We also included studies in which only a portion of the study sample fit our inclusion criteria, provided they were compared independently to a control group and analyzed independently. Conversely, if arms of an included study did not fit our inclusion criteria, those arms of interventions were not included in our analysis.

This review focused on the effects of the following interventions:
1. Interventions to promote dietary diversification and appropriate complementary feeding. These will be divided in interventions that provide only education and interventions in which the provision of complementary food for healthy individuals takes place regardless of education.
2. Interventions to prevent moderate and severe acute malnutrition, such as educative interventions and supplementary food for children suffering from MAM or SAM.

Interventions were supervised by healthcare professionals, community health workers and through telephone network platforms e.g., text messages, phone apps. Interventions were compared to control groups receiving no interventions or standard of care.

Primary outcomes: Infant growth (weight gain (kg), height gain (cm), Z-scores for height-for-age (HAZ), weight-for-age (WAZ), weight-for-height (WHZ), stunting (HAZ < 2), wasting (WAZ < 2), underweight (WHZ < 2)). Our secondary outcomes included neonatal mortality (death from all causes within a month of birth per total live births), infant mortality (death from all causes within 12 months of age per total live births), neonatal sepsis (the proportion of neonates dying due to possible serious bacterial infections among all neonates), incidence of acute respiratory

infections (ARI), incidence of diarrheal illness, and any adverse events including gastrointestinal symptoms. Studies were excluded if they did not report any of the outlined outcomes.

S3.2.2. Search methods for identification of studies

The literature review was conducted on the following databases: Cochrane Controlled Trials Register (CENTRAL), MEDLINE, EMBASE, CINAHL, PsycINFO, ERIC, Sociofiles, HMIS (Health Management Information Consortium), CAB Global Health (https://www.cabi.org/publishing-products/onlineinformation-resources/global-health/), the WHO nutrition databases (http://www.who.int/nutrition/databases/en/), Popline (https://www.popline.org), Epistemonikos (https://www.epistemonikos.org/en/), Social Science Citation Index, Dissertation Abstracts International, and WHO Global Health Index which covers the WHO Regional journals from Latin America (LILACS), Africa (AFRO) etc. We also searched the web sites of selected development agencies or research firms (for example, JOLIS, IDEAS, IFPRI, NBER, USAID, World Bank and Eldis.org). The trials registry Clinicaltrials.gov and WHO's ICRTP were searched for ongoing trials. Additionally, EPOC filters were used for quantitative designs (EPOC 2017b). Every effort was made to contact the relevant organization, institutions and experts for the identification of unpublished and ongoing studies. Google Scholar and the Web of Sciences were searched for citations and the references sections and annotated bibliographies were cross referenced for bonus eligible studies. The search update was originally run on July 17, 2021. The search was then updated once more for a final date of April 13, 2023.

S3.2.3. Data collection and analysis

Data collection and analysis was conducted in accordance with the Cochrane Handbook for Systematic Reviews of Interventions. The selected literature was double screened at the title and abstract level by two independent review authors. The resulting full-texts were similarly double-screened by two independent authors with reasons recorded for exclusions. Disagreements were resolved by the consultation of a third senior review author. Duplicates were excluded. Multiple reports of the same study were collated to ensure each study was the unit of interest in this review. The selection process was recorded sufficient to construct a Preferred Reporting Items for Systematic Reviews and Meta-Analyses (PRISMA) flow diagram. Two independent review authors extracted data on data extraction sheets on Microsoft Excel. Independent data extraction sheets were matched, and disagreements were resolved by discussion and third senior review author was consulted, if the need arose. We used a piloted data collection form for study characteristics and outcome data. If any information from the study was unclear or missing or could not be from the publications, the authors were contacted for further clarification.

Data was exported and analyzed on RevMan v5.4. Risk ratios (RR) were used for dichotomous outcomes, mean differences (MD) were calculated for continuous outcomes and standard mean differences were calculated for continuous outcomes reporting the same outcome but measured on a different scale within the same meta-analysis. A 95% confidence interval was used for all estimates of effect. We analyzed outcomes from studies with multiple groups in an

appropriate way to avoid double counting of participants by adding them as an appropriately duplicated entry with an added footnote specifying the differences between groups.

S3.3. Prisma Diagram

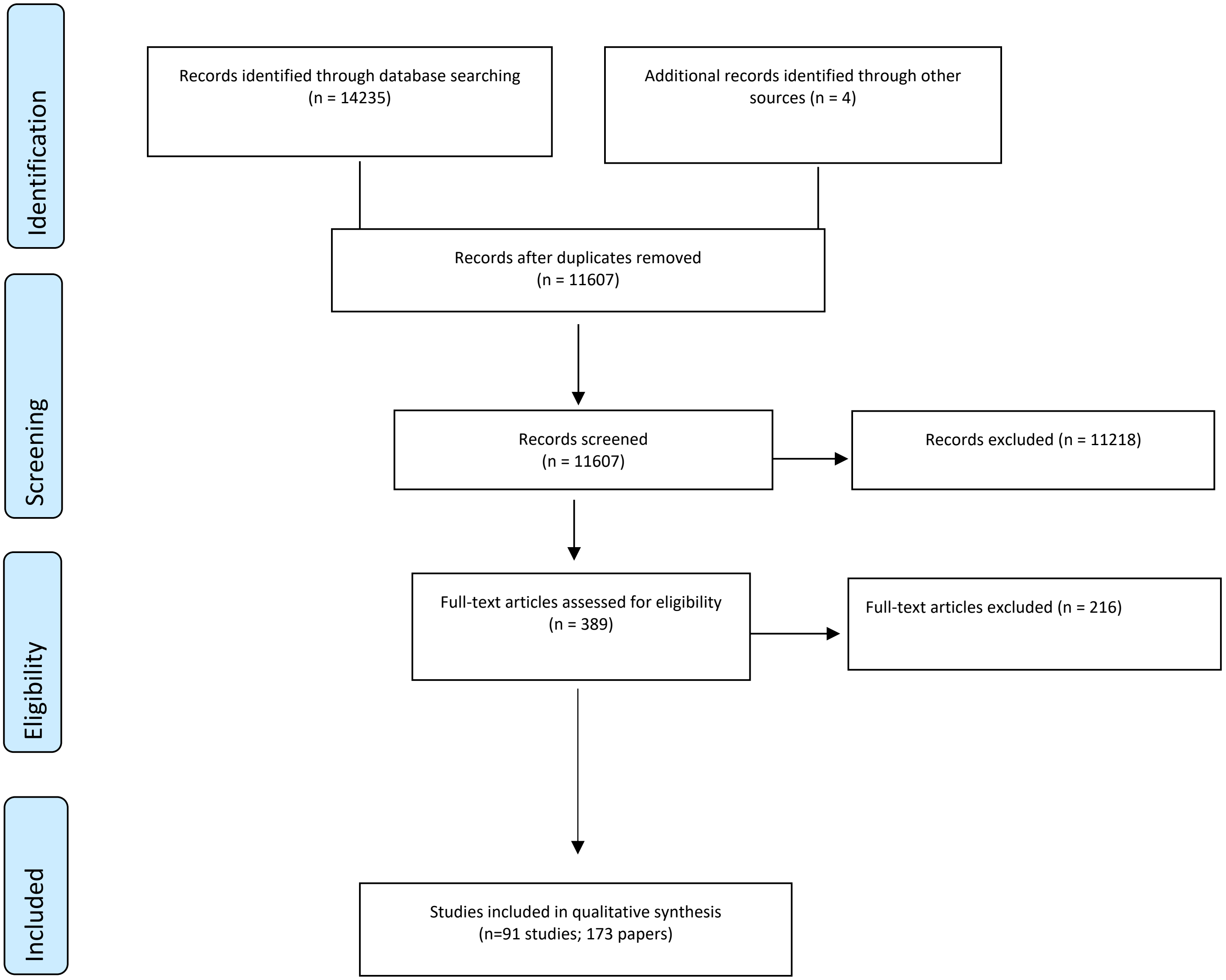

S3.4. List of included studies

Table S3.4.A. Included studies for complementary feeding provision vs control (N=43)

#	Citations
1	Addo OY, Locks LM, Jefferds ME, Nanama S, Albert B, Sandalinas F, et al. Combined infant and young child feeding with small-quantity lipid-based nutrient supplementation is associated with a reduction in anemia but no changes in anthropometric status of young children from Katanga Province of the Democratic Republic of Congo: a quasi-experimental effectiveness study. Am J Clin Nutr. 2020;112(3):683-94.
2	Adu-Afarwuah S, Lartey A, Brown KH, Zlotkin S, Briend A, Dewey KG. Randomized comparison of 3 types of micronutrient supplements for home fortification of complementary foods in Ghana: effects on growth and motor development. The American journal of clinical nutrition. 2007;86(2):412-20.
3	Adu-Afarwuah S, Lartey A, Okronipa H, Ashorn P, Peerson JM, Arimond M, et al. Small-quantity, lipid-based nutrient supplements provided to women during pregnancy and 6 mo postpartum and to their infants from 6 mo of age increase the mean attained length of 18-mo-old children in semi-urban Ghana: a randomized controlled trial. The American journal of clinical nutrition. 2016;104(3):797-808.
4	Agapova SE, Stephenson KB, Divala O, Kaimila Y, Maleta KM, Thakwalakwa C, et al. Additional common bean in the diet of Malawian children does not affect linear growth, but reduces intestinal permeability. The Journal of nutrition. 2018;148(2):267-74.
5	Akalu G, Taffesse S, Gunaratna NS, De Groote H. The effectiveness of quality protein maize in improving the nutritional status of young children in the Ethiopian highlands. Food Nutr Bull. 2010;31(3):418-30.
6	Ashorn P, Alho L, Ashorn U, Cheung YB, Dewey KG, Gondwe A, et al. Supplementation of maternal diets during pregnancy and for 6 months postpartum and infant diets thereafter with small-quantity lipid-based nutrient supplements does not promote child growth by 18 months of age in rural Malawi: a randomized controlled trial. The Journal of nutrition. 2015;145(6):1345-53.<br><br>Ashorn P, Alho L, Ashorn U, Cheung YB, Dewey KG, Harjunmaa U, et al. The impact of lipid-based nutrient supplement provision to pregnant women on newborn size in rural Malawi: a randomized controlled trial. The American journal of clinical nutrition. 2015;101(2):387-97.
7	Bauserman M, Lokangaka A, Gado J, Close K, Wallace D, Kodondi KK, et al. A cluster-randomized trial determining the efficacy of caterpillar cereal as a locally available and sustainable complementary food to prevent stunting and anaemia. Public Health Nutr. 2015;18(10):1785-92.
8	Becquey E, Huybregts L, Zongrone A, Le Port A, Leroy JL, Rawat R, et al. Impact on child acute malnutrition of integrating a preventive nutrition package into facility-based

	screening for acute malnutrition during well-baby consultation: a cluster-randomized controlled trial in Burkina Faso. PLoS medicine. 2019;16(8):e1002877.
9	Maleta KM, Phuka J, Alho L, Cheung YB, Dewey KG, Ashorn U, et al. Provision of 10-40 g/d Lipid-Based Nutrient Supplements from 6 to 18 Months of Age Does Not Prevent Linear Growth Faltering in Malawi. J Nutr. 2015;145(8):1909-15.
10	Bhandari N, Bahl R, Nayyar B, Khokhar P, Rohde JE, Bhan MK. Food supplementation with encouragement to feed it to infants from 4 to 12 months of age has a small impact on weight gain. Journal of Nutrition. 2001;131(7):1946-51.
11	Borg B, Sok D, Mihrshahi S, Griffin M, Chamnan C, Berger J, et al. Effectiveness of a locally produced ready-to-use supplementary food in preventing growth faltering for children under 2 years in Cambodia: a cluster randomised controlled trial. Maternal and Child Nutrition. 2020;16(1).
12	Chipili G, Van Graan A, Lombard CJ, Van Niekerk E. The Efficacy of Fish as an Early Complementary Food on the Linear Growth of Infants Aged 6–7 Months: A Randomised Controlled Trial. Nutrients. 2022;14(11):2191.
13	Christian P, Shaikh S, Shamim AA, Mehra S, Wu L, Merrill RD, et al. Effect of fortified complementary food supplementation on child growth in rural Bangladesh-a cluster randomized trial. FASEB Journal. 2015;29(1).
14	Dewey KG, Mridha MK, Matias SL, Arnold CD, Cummins JR, Khan MSA, et al. Lipid-based nutrient supplementation in the first 1000 d improves child growth in Bangladesh: A cluster-randomized effectiveness trial. AMERICAN JOURNAL OF CLINICAL NUTRITION. 2017;105(4):944-57.
15	Galasso E, Weber AM, Stewart CP, Ratsifandrihamanana L, Fernald LCH. Effects of nutritional supplementation and home visiting on growth and development in young children in Madagascar: a cluster-randomised controlled trial. The Lancet Global Health. 2019;7(9):e1257-e68.
16	Ghosh SA, Strutt NR, Otoo GE, Suri DJ, Ankrah J, Johnson T, et al. A macro- and micronutrient-fortified complementary food supplement reduced acute infection, improved haemoglobin and showed a dose-response effect in improving linear growth: a 12-month cluster randomised trial. Journal of nutritional science. 2019;8.
17	Hess SY, Abbeddou S, Jimenez EY, Somé JW, Vosti SA, Ouédraogo ZP, et al. Small-quantity lipid-based nutrient supplements, regardless of their zinc content, increase growth and reduce the prevalence of stunting and wasting in young burkinabe children: a cluster-randomized trial. PloS one. 2015;10(3):e0122242.
18	Hurley KM, Phuka J, Kang Y, Ruel-Bergeron J, Buckland AJ, Mitra M, et al. A longitudinal impact evaluation of a comprehensive nutrition program for reducing stunting among children aged 6-23 months in rural Malawi. Am J Clin Nutr. 2021 Jul 1;114(1):248-256.
19	Prendergast AJ, Chasekwa B, Evans C, Mutasa K, Mbuya MNN, Stoltzfus RJ, et al. Independent and combined effects of improved water, sanitation, and hygiene, and improved complementary feeding, on stunting and anaemia among HIV-exposed children in rural Zimbabwe: a cluster-randomised controlled trial. The Lancet Child and Adolescent Health. 2019;3(2):77-90.

	Humphrey JH, Mbuya MN, Ntozini R, Moulton LH, Stoltzfus RJ, Tavengwa NV, et al. Independent and combined effects of improved water, sanitation, and hygiene, and improved complementary feeding, on child stunting and anaemia in rural Zimbabwe: a cluster-randomised trial. The Lancet Global Health. 2019;7(1):e132-e47.
20	Huybregts L, Le Port A, Becquey E, Zongrone A, Barba FM, Rawat R, et al. Impact on child acute malnutrition of integrating small-quantity lipid-based nutrient supplements into community-level screening for acute malnutrition: A cluster-randomized controlled trial in Mali. PLoS medicine. 2019;16(8).
21	Huybregts L, Houngbé F, Salpéteur C, Brown R, Roberfroid D, Ait-Aissa M, et al. The Effect of Adding Ready-to-Use Supplementary Food to a General Food Distribution on Child Nutritional Status and Morbidity: A Cluster-Randomized Controlled Trial. PLoS medicine. 2012;9(9).
22	Iannotti LL, Dulience SJL, Green J, Joseph S, François J, Antenor M-L, et al. Linear growth increased in young children in an urban slum of Haiti: a randomized controlled trial of a lipid-based nutrient supplement. The American journal of clinical nutrition. 2014;99(1):198-208.
23	Iannotti LL, Lutter CK, Stewart CP, Riofrío CAG, Malo C, Reinhart G, et al. Eggs in early complementary feeding and child growth: a randomized controlled trial. Pediatrics. 2017;140(1).
24	Isanaka S, Nombela N, Djibo A, Poupard M, Van Beckhoven D, Gaboulaud V, et al. Effect of preventive supplementation with ready-to-use therapeutic food on the nutritional status, mortality, and morbidity of children aged 6 to 60 months in Niger: a cluster randomized trial. Jama. 2009;301(3):277-85.
25	Khan GN, Kureishy S, Ariff S, Rizvi A, Sajid M, Garzon C, et al. Effect of lipid-based nutrient supplement-Medium quantity on reduction of stunting in children 6-23 months of age in Sindh, Pakistan: A cluster randomized controlled trial. PloS one. 2020;15(8):e0237210.
26	Langendorf C, Roederer T, De Pee S, Brown D, Doyon S, Mamaty A, et al. Preventing acute malnutrition among children aged 6 to 23 months in Niger: Effect of supplementation and cash transfer. PLoS Med. 2014 Sep 2;11(9):e1001714.
27	Luby SP, Rahman M, Arnold BF, Unicomb L, Ashraf S, Winch PJ, et al. Effects of water quality, sanitation, handwashing, and nutritional interventions on diarrhoea and child growth in rural Bangladesh: a cluster randomised controlled trial. The Lancet Global Health. 2018;6(3):e302-e15.
28	Lutter CK, Rodríguez A, Fuenmayor G, Avila L, Sempertegui F, Escobar J. Growth and micronutrient status in children receiving a fortified complementary food. The Journal of nutrition. 2008;138(2):379-88.
29	Mangani C, Maleta K, Phuka J, Cheung YB, Thakwalakwa C, Dewey K, et al. Effect of complementary feeding with lipid-based nutrient supplements and corn-soy blend on the incidence of stunting and linear growth among 6- to 18-month-old infants and children in rural Malawi. Matern Child Nutr. 2015;11 Suppl 4(Suppl 4):132-43.
30	Muslihah N, Khomsan A, Briawan D, Riyadi H. Complementary food supplementation with a small-quantity of lipid-based nutrient supplements prevents stunting in 6-12-

	month-old infants in rural West Madura Island, Indonesia. Asia Pac J Clin Nutr. 2016 Dec;25(Suppl 1):S36-S42.
31	Null C, Stewart CP, Pickering AJ, Dentz HN, Arnold BF, Arnold CD, et al. Effects of water quality, sanitation, handwashing, and nutritional interventions on diarrhoea and child growth in rural Kenya: a cluster-randomised controlled trial. The Lancet Global Health. 2018;6(3):e316-e29.
32	Obatolu VA. Growth pattern of infants fed with a mixture of extruded malted maize and cowpea. Nutrition. 2003;19(2):174-8.
33	Oelofse A, Van Raaij JM, Benade AS, Dhansay MA, Tolboom JJ, Hautvast J. The effect of a micronutrient-fortified complementary food on micronutrient status, growth and development of 6-to 12-month-old disadvantaged urban South African infants. International journal of food sciences and nutrition. 2003;54(5):399-407.
34	Omer A, Hailu D, Whiting SJ. Effect of a Child-Owned Poultry Intervention Providing Eggs on Nutrition Status and Motor Skills of Young Children in Southern Ethiopia: A Cluster Randomized and Controlled Community Trial. International Journal of Environmental Research and Public Health. 2022;19(22):15305.
35	Owino VO, Kasonka LM, Sinkala MM, Wells JK, Eaton S, Darch T, et al. Fortified complementary foods with or without α-amylase treatment increase hemoglobin but do not reduce breast milk intake of 9-mo-old Zambian infants. The American journal of clinical nutrition. 2007;86(4):1094-103.<br><br>Owino VO, Sinkala M, Amadi B, Tomkins AM, Filteau SM. Acceptability, storage stability and costing of α-amylase-treated maize–beans–groundnuts–bambaranuts complementary blend. Journal of the Science of Food and Agriculture. 2007;87(6):1021-9.
36	Phu PV, Hoan NV, Salvignol B, Treche S, Wieringa FT, Dijkhuizen MA, et al. A Six-Month Intervention with Two Different Types of Micronutrient-Fortified Complementary Foods Had Distinct Short- and Long-Term Effects on Linear and Ponderal Growth of Vietnamese Infants. Journal of Nutrition. 2012;142(9):1735-40.
37	Roberts SB, Franceschini MA, Silver RE, Taylor SF, De Sa AB, Có R, et al. Effects of food supplementation on cognitive function, cerebral blood flow, and nutritional status in young children at risk of undernutrition: Randomized controlled trial. BMJ. 2020 Jul 22;370:m2397.
38	Samuel A, Brouwer ID, Feskens EJ, Adish A, Kebede A, De-Regil LM, et al. Effectiveness of a program intervention with reduced-iron multiple micronutrient powders on iron status, morbidity and growth in young children in Ethiopia. Nutrients. 2018;10(10):1508.
39	Santos IS, Gigante DP, Coitinho DC, Haisma H, Valle NC, Valente G. Evaluation of the impact of a nutritional program for undernourished children in Brazil. Cadernos de Saúde Pública. 2005;21:776-85.
40	Sazawal S, Dhingra P, Dhingra U, Gupta S, Iyengar V, Menon VP, et al. Compliance with home-based fortification strategies for delivery of iron and zinc: its effect on

	haematological and growth markers among 6-24 months old children in north India. Journal of health, population, and nutrition. 2014;32(2):217.
41	Siega-Riz AM, Estrada Del Campo Y, Kinlaw A, Reinhart GA, Allen LH, Shahab-Ferdows S, et al. Effect of supplementation with a lipid-based nutrient supplement on the micronutrient status of children aged 6-18 months living in the rural region of Intibuca, Honduras. Paediatr Perinat Epidemiol. 2014;28(3):245-54.
42	Smuts CM, Matsungo TM, Malan L, Kruger HS, Rothman M, Kvalsvig JD, et al. Effect of small-quantity lipid-based nutrient supplements on growth, psychomotor development, iron status, and morbidity among 6-to 12-mo-old infants in South Africa: a randomized controlled trial. The American journal of clinical nutrition. 2019;109(1):55-68.
43	Stewart CP, Caswell B, Iannotti L, Lutter C, Arnold CD, Chipatala R, et al. The effect of eggs on early child growth in rural Malawi: the Mazira Project randomized controlled trial. The American journal of clinical nutrition. 2019;110(4):1026-33.
44	Zhang Y, Wu Q, Wang W, van Velthoven MH, Chang S, Han H, et al. Effectiveness of complementary food supplements and dietary counselling on anaemia and stunting in children aged 6–23 months in poor areas of Qinghai Province, China: a controlled interventional study. BMJ open. 2016;6(10):e011234.

Table S3.4.B. Included studies for complementary feeding education vs control (N=44)

#	Citations
1	Abiyu C, Belachew T. Effect of complementary feeding behavior change communication delivered through community-level actors on the time of initiation of complementary foods in rural communities of West Gojjam zone, Northwest Ethiopia: a cluster-randomized controlled trial. BMC PEDIATRICS. 2020;20(1).
2	Ali MK, Flacking R, Sulaiman M, Osman F. Effects of Nutrition Counselling and Unconditional Cash Transfer on Child Growth and Family Food Security in Internally Displaced Person Camps in Somalia—A Quasi-Experimental Study. International Journal of Environmental Research and Public Health. 2022;19(20):13441.
3	Bhandari N, Bahl R, Nayyar B, Khokhar P, Rohde JE, Bhan MK. Food supplementation with encouragement to feed it to infants from 4 to 12 months of age has a small impact on weight gain. Journal of Nutrition. 2001;131(7):1946-51.
4	Bhandari N, Mazumder S, Bahl R, Martines J, Black RE, Bhan MK. An educational intervention to promote appropriate complementary feeding practices and physical growth in infants and young children in Rural Haryana, India. Journal of Nutrition. 2004;134(9):2342-8.
5	Brown LV, Zeitlin MF, Peterson KE, Chowdhury A, Rogers BL, Weld LH, et al. Evaluation of the impact of weaning food messages on infant feeding practices and child growth in rural Bangladesh. The American Journal of Clinical Nutrition. 1992;56(6):994-1003.
6	Fahmida U, Htet MK, Ferguson E, Do TT, Buanasita A, Titaley C, et al. Effect of an integrated package of nutrition behavior change interventions on infant and young child feeding practices and child growth from birth to 18 months: Cohort evaluation of

	the baduta cluster randomized controlled trial in east Java, Indonesia. Nutrients. 2020;12(12):1-16.
7	Gope RK, Tripathy P, Prasad V, Pradhan H, Sinha RK, Panda R, et al. Effects of participatory learning and action with women's groups, counselling through home visits and crèches on undernutrition among children under three years in eastern India: a quasi-experimental study. BMC public health. 2019;19(1):962.
8	Guldan GS, Fan HC, Ma X, Ni ZZ, Xiang X, Tang MZ. Culturally appropriate nutrition education improves infant feeding and growth in rural Sichuan, China. J Nutr. 2000;130(5):1204-11.
9	Hitachi M, Wanjihia V, Nyandieka L, Francesca C, Wekesa N, Changoma J, et al. Improvement of dietary diversity and attitude toward recommended feeding through novel community based nutritional education program in coastal kenya—an intervention study. International Journal of Environmental Research and Public Health. 2020;17(19):1-11.
10	Kabahenda MK, Andress EL, Nickols SY, Kabonesa C, Mullis RM. Promoting dietary diversity to improve child growth in less-resourced rural settings in Uganda. J Hum Nutr Diet. 2014;27 Suppl 2:143-51.
11	Kang Y, Kim S, Sinamo S, Christian P. Effectiveness of a community-based nutrition programme to improve child growth in rural Ethiopia: a cluster randomized trial. Maternal and Child Nutrition. 2017;13(1).
12	Kilaru A, Griffiths P, Ganapathy S, Shanti G. Community-based nutrition education for improving infant growth in rural Karnataka. Indian pediatrics. 2005;42(5):425.
13	Kuchenbecker J, Reinbott A, Mtimuni B, Krawinkel MB, Jordan I. Nutrition education improves dietary diversity of children 6-23 months at community-level: Results from a cluster randomized controlled trial in Malawi. PloS one. 2017;12(4).
14	Martinez B, Webb MF, Gonzalez A, Douglas K, Grazioso MD, Rohloff P. Complementary feeding intervention on stunted Guatemalan children: a randomised controlled trial. BMJ PAEDIATRICS OPEN. 2018;2(1).
15	Miller LC, Neupane S, Joshi N, Lohani M, Rogers BL, Neupane S, et al. Multisectoral community development in Nepal has greater effects on child growth and diet than nutrition education alone. Public health nutrition. 2020;23(1):146-61.
16	More NS, Das S, Bapat U, Alcock G, Manjrekar S, Kamble V, et al. Community resource centres to improve the health of women and children in informal settlements in Mumbai: a cluster-randomised, controlled trial. Lancet Glob Health. 2017;5(3):e335-e49.
17	Muhoozi GKM, Atukunda P, Diep LM, Mwadime R, Kaaya AN, Skaare AB, et al. Nutrition, hygiene, and stimulation education to improve growth, cognitive, language, and motor development among infants in Uganda: A cluster-randomized trial. Matern Child Nutr. 2018;14(2):e12527.
18	Negash C, Belachew T, Henry CJ, Kebebu A, Abegaz K, Whiting SJ. Nutrition education and introduction of broad bean—based complementary food improves knowledge and dietary practices of caregivers and nutritional status of their young children in Hula, Ethiopia. Food and Nutrition Bulletin. 2014;35(4):480-6.

19	Navarro JI, Sigulem DM, Ferraro AA, Polanco JJ, Barros AJ. The double task of preventing malnutrition and overweight: a quasi-experimental community-based trial. BMC Public Health. 2013;13:1-12.
20	Nair N, Tripathy P, Sachdev H, Pradhan H, Bhattacharyya S, Gope R, et al. Effect of participatory women's groups and counselling through home visits on children's linear growth in rural eastern India (CARING trial): a cluster-randomised controlled trial. The Lancet Global Health. 2017;5(10):e1004-e16.
21	Nayak DS, Kondagunta N, Kamath VG, Kamath A, Nair S. Impact of family level counselling on breast feeding practices and weight gain: A community based cluster randomized controlled trial. Int J Community Med Public Health. 2016;3:486-93.
22	Nyamasege C, Kimani-Murage E, Wanjohi M, Kaindi D, Ma E, Fukushige M, et al. Determinants of low birth weight in the context of maternal nutrition education in urban informal settlements, Kenya. Journal of developmental origins of health and disease. 2019;10(2):237-45.
23	Olaya GA, Lawson M, Fewtrell MS. Efficacy and safety of new complementary feeding guidelines with an emphasis on red meat consumption: a randomized trial in Bogota, Colombia. The American journal of clinical nutrition. 2013;98(4):983-93.
24	Osei AK, Pandey P, Spiro D, Adhikari D, Haselow N, De Morais C, et al. Adding multiple micronutrient powders to a homestead food production programme yields marginally significant benefit on anaemia reduction among young children in N epal. Maternal & Child Nutrition. 2015;11:188-202.
25	Owais A, Schwartz B, Kleinbaum DG, Suchdev PS, Faruque ASG, Das SK, et al. A nutrition education program in rural bangladesh was associated with improved feeding practices but not with child growth. Journal of Nutrition. 2017;147(5):948-54.
26	Penny ME, Creed-Kanashiro HM, Robert RC, Narro MR, Caulfield LE, Black RE. Effectiveness of an educational intervention delivered through the health services to improve nutrition in young children: a cluster-randomised controlled trial. The lancet. 2005;365(9474):1863-72.
27	Rachmawati Rachmawati, Rosi Novita, Erwandi Erwandi, Eva Fitriyaningsih, Rinaldi Idroes and Novi Reandy Sasmita, 2020. The Effectiveness of Counseling on Complementary Food for Mothers and Supplementary Feeding for Increasing weight of Breastfed babies (12-24 Months of Age) in the Aceh Besar District of Indonesia. Pakistan Journal of Nutrition, 19: 279-284.
28	Reinbott A, Schelling A, Kuchenbecker J, Jeremias T, Russell I, Kevanna O, et al. Nutrition education linked to agricultural interventions improved child dietary diversity in rural Cambodia. British journal of nutrition. 2016;116(8):1457-68.
29	Roy SK, Jolly SP, Shafique S, Fuchs GJ, Mahmud Z, Chakraborty B, et al. Prevention of malnutrition among young children in rural Bangladesh by a food-health-care educational intervention: A randomized, controlled trial. Food and Nutrition Bulletin. 2007;28(4):375-83.
30	Roy S, Jahan K, Alam N, Tasnim S, Rois R. Assessing acceptance and effects of child feeding counseling on nutritional status of children aged 6-23 months in a semi-urban community. British Journal of Nutrition. 2022:1-24.

31	Saleem AF, Mahmud S, Baig-Ansari N, Zaidi AK. Impact of maternal education about complementary feeding on their infants' nutritional outcomes in low- and middle-income households: a community-based randomized interventional study in Karachi, Pakistan. Journal of health, population, and nutrition. 2014;32(4):623-33.
32	Salehi M, Kimiagar SM, Shahbazi M, Mehrabi Y, Kolahi AA. Assessing the impact of nutrition education on growth indices of Iranian nomadic children: An application of a modified beliefs, attitudes, subjective-norms and enabling-factors model. British journal of nutrition. 2004;91(5):779-87.
33	Santos I, Victora CG, Martines J, Gonçalves H, Gigante DP, Valle NJ, et al. Nutrition counseling increases weight gain among Brazilian children. The Journal of nutrition. 2001;131(11):2866-73.
34	Sharma U. Utilising dais to impart health and nutrition education : impact on growth. Indian J Pediat. 1987;54:111-8.
35	Sharma N, Gupta M, Aggarwal AK, Gorle M. Effectiveness of a culturally appropriate nutrition educational intervention delivered through health services to improve growth and complementary feeding of infants: A quasi-experimental study from Chandigarh, India. PloS one. 2020;15(3).
36	Singh V, Ahmed S, Dreyfuss ML, Kiran U, Chaudhery DN, Srivastava VK, et al. An integrated nutrition and health program package on IYCN improves breastfeeding but not complementary feeding and nutritional status in rural northern India: A quasi-experimental randomized longitudinal study. PloS one. 2017;12(9).<br><br>Singh V, Ahmed S, Dreyfuss ML, Kiran U, Chaudhery DN, Srivastava VK, et al. Non-governmental organization facilitation of a community-based nutrition and health program: Effect on program exposure and associated infant feeding practices in rural India. PLoS One. 2017;12(9):e0183316.
37	Susanto T, Yunanto RA, Rasny H, Susumaningrum LA, Nur KRM. Promoting Children Growth and Development: A community-based cluster randomized controlled trial in rural areas of Indonesia. Public Health Nurs. 2019;36(4):514-24.
38	Teshome GB, Whiting SJ, Green TJ, Mulualem D, Henry CJ. Scaled-up nutrition education on pulse-cereal complementary food practice in Ethiopia: a cluster-randomized trial. BMC public health. 2020;20(1):1437.<br><br>Teshome GB, Whiting SJ, Green TJ, Mulualem D, Henry CJ. Scaled-up nutrition education on pulse-cereal complementary food practice in Ethiopia: a cluster-randomized trial. BMC public health. 2020;20(1):1-12.
39	Vazir S, Engle P, Balakrishna N, Griffiths PL, Johnson SL, Creed-Kanashiro H, et al. Cluster-randomized trial on complementary and responsive feeding education to caregivers found improved dietary intake, growth and development among rural Indian toddlers. Maternal and Child Nutrition. 2013;9(1):99-117.
40	Vitolo MR, Bortolini GA, Feldens CA, Drachler MdL. Impactos da implementação dos dez passos da alimentação saudável para crianças: ensaio de campo randomizado. Cadernos de saúde pública. 2005;21:1448-57.

41	Yadav DK, Gupta N, Shrestha N, Kumar A, Bose D. Community Based Nutrition Education for Promoting Nutritional Status of Children under Three Years of Age in Rural Areas of Mahottari District of Nepal. Journal of Nepal Paediatric Society. 2014;34(3).
42	Younes L, Houweling TA, Azad K, Kuddus A, Shaha S, Haq B, et al. The effect of participatory women's groups on infant feeding and child health knowledge, behaviour and outcomes in rural Bangladesh: a controlled before-and-after study. J Epidemiol Community Health. 2015;69(4):374-81.
43	Young MF, Baik D, Reinsma K, Gosdin L, Rogers HP, Oy S, et al. Evaluation of mobile phone-based Positive Deviance/Hearth child undernutrition program in Cambodia. Maternal & Child Nutrition. 2021;17(4):e13224.<br><br>Baik D, Reinsma K, Chhorvann C, Oy S, Heang H, Young MF. Program Impact Pathway of the Positive Deviance/Hearth Interactive Voice Calling Program in a Peri-Urban Context of Cambodia. Current Developments in Nutrition. 2022;6(5):nzac045.
44	Yousafzai AK, Rasheed MA, Rizvi A, Armstrong R, Bhutta ZA. Effect of integrated responsive stimulation and nutrition interventions in the Lady Health Worker programme in Pakistan on child development, growth, and health outcomes: a cluster-randomised factorial effectiveness trial. The Lancet. 2014;384(9950):1282-93.
45	Zaman S, Ashraf RN, Martines J. Training in complementary feeding counselling of healthcare workers and its influence on maternal behaviours and child growth: a cluster-randomized controlled trial in Lahore, Pakistan. Journal of health, population, and nutrition. 2008;26(2):210.
46	Zhang JX, Shi L, Chen DF, Wang J, Wang Y. Effectiveness of an educational intervention to improve child feeding practices and growth in rural China: updated results at 18 months of age. Maternal and Child Nutrition. 2013;9(1):118-29.
**Undernourished children**	
47	Seyyedi N, Rahimi B, Eslamlou HRF, Afshar HL, Spreco A, Timpka T. Smartphone-based maternal education for the complementary feeding of undernourished children under 3 years of age in food-secure communities: randomised controlled trial in Urmia, Iran. Nutrients. 2020;12(2):587.

S3.5. GRADE assessments

S3.5.A. GRADE tables for CF supplementation vs control

Child CF compared to control

Certainty assessment							Summary of findings				
Participants (studies) Follow-up	Risk of bias	Inconsistency	Indirectness	Imprecision	Publication bias	Overall certainty of evidence	Study event rates (%)		Relative effect (95% CI)	Anticipated absolute effects	
							With control	With Child CF		Risk with control	Risk difference with Child CF
**HAZ - Fortified Blended Food**											
5820 (11 studies)	very serious[a]	very serious[b]	not serious	serious[c]	none	⨁◯◯◯ Very low	3019	2801	-	The mean HAZ - Fortified Blended Food was **0**	MD **0.25 higher** (0.09 higher to 0.41 higher)
**HAZ - Locally Produced Ready to Use Supplementary Food**											
2907 (4 studies)	serious[d]	not serious	not serious	serious[c]	none	⨁⨁◯◯ Low	1201	1706	-	The mean HAZ - Locally Produced Ready to Use Supplementary Food was **0**	MD **0.04 higher** (0.02 higher to 0.06 higher)
**Stunting - Fortified Blended Food**											
7358 (7 studies)	very serious[e]	very serious[b]	not serious	serious[c,f]	none	⨁◯◯◯ Very low	-/3148	-/4210	**RR 0.73** (0.56 to 0.95)	0 per 1,000	**0 fewer per 1,000** (from 0 fewer to 0 fewer)

Child CF compared to control

Certainty assessment							Summary of findings				
**Stunting - Commercially Produced Ready to Use Supplementary Food**											
2634 (3 studies)	very serious[g]	not serious	not serious	not serious	none	⊕⊕◯◯ Low	-/1247	-/1387	**RR 0.90** (0.83 to 0.98)	0 per 1,000	**1 fewer per 1,000** (from 1 fewer to 1 fewer)
**Wasting - SQ LNS**											
16976 (13 studies)	very serious[h]	not serious	not serious	serious[c]	none	⊕◯◯◯ Very low	-/7534	-/9442	**RR 0.90** (0.82 to 0.98)	0 per 1,000	**0 fewer per 1,000** (from 0 fewer to 0 fewer)
**WHZ - Fortified Blended Food**											
6966 (11 studies)	very serious[i]	very serious[j]	not serious	not serious	none	⊕◯◯◯ Very low	3567	3399	-	The mean WHZ - Fortified Blended Food was **0**	MD **0.08 higher** (0.01 higher to 0.15 higher)
**WHZ - Commercially Produced Ready to Use Supplementary Food**											
2581 (3 studies)	very serious[g]	not serious	not serious	not serious	none	⊕⊕◯◯ Low	1208	1373	-	The mean WHZ - Commercially Produced Ready to Use Supplementary Food was **0**	MD **0.04 higher** (0.01 higher to 0.07 higher)
**WHZ - Locally Produced Ready to Use Supplementary Food**											

Child CF compared to control

Certainty assessment							Summary of findings				
2576 (3 studies)	very serious[k]	not serious	not serious	not serious	none	⊕⊕◯◯ Low	1105	1471	-	The mean WHZ - Locally Produced Ready to Use Supplementary Food was **0**	MD **0.02 higher** (0 to 0.04 higher)
**WAZ - Fortified Blended Food**											
5995 (12 studies)	very serious[l]	very serious[b]	not serious	not serious	none	⊕◯◯◯ Very low	3069	2926	-	The mean WAZ - Fortified Blended Food was **0**	MD **0.16 higher** (0.03 higher to 0.3 higher)
**WAZ - Locally Produced Ready to Use Supplementary Food**											
2576 (3 studies)	very serious[m]	not serious	not serious	not serious	none	⊕⊕◯◯ Low	1105	1471	-	The mean WAZ - Locally Produced Ready to Use Supplementary Food was **0**	MD **0.03 higher** (0.01 higher to 0.05 higher)
**WAZ - Commercially Produced Ready to Use Supplementary Food**											
1911 (2 studies)	very serious[g]	not serious	not serious	not serious	none	⊕⊕◯◯ Low	924	987	-	The mean WAZ - Commercially Produced Ready to Use Supplementary Food was **0**	MD **0.03 higher** (0 to 0.06 higher)

Weight - Locally Produced Ready to Use Supplementary Food

Child CF compared to control

Certainty assessment							Summary of findings				
2576 (3 studies)	very serious[m]	not serious	not serious	not serious	none	⊕⊕◯◯ Low	1105	1471	-	The mean weight - Locally Produced Ready to Use Supplementary Food was **0**	MD **0.03 higher** (0.01 higher to 0.05 higher)

Weight - Commercially Produced Ready to Use Supplementary Food

1911 (2 studies)	very serious[g]	not serious	not serious	serious[c]	none	⊕◯◯◯ Very low	924	987	-	The mean weight - Commercially Produced Ready to Use Supplementary Food was **0**	MD **0.04 higher** (0.01 higher to 0.07 higher)

Height - Locally Produced Ready to Use Supplementary Food

2576 (3 studies)	very serious[m]	not serious	not serious	not serious	none	⊕⊕◯◯ Low	1105	1471	-	The mean height - Locally Produced Ready to Use Supplementary Food was **0**	MD **0.08 higher** (0.05 higher to 0.12 higher)

Height - Commercially Produced Ready to Use Supplementary Food

1911 (2 studies)	very serious[g]	not serious	not serious	not serious	none	⊕⊕◯◯ Low	924	987	-	The mean height - Commercially Produced Ready to Use Supplementary Food was **0**	MD **0.06 higher** (0 to 0.11 higher)

MUAC (cm) - Commercially Produced Ready to Use Supplementary Food

Child CF compared to control

Certainty assessment							Summary of findings				
670<br>(1 study)	very serious[g]	not serious	not serious	not serious	none	⊕⊕◯◯<br>Low	284	386	-	The mean MUAC (cm) - Commercially Produced Ready to Use Supplementary Food was **0**	MD **0.2 higher** (0.02 higher to 0.38 higher)
**MUAC (cm) - SQ LNS**											
9411<br>(8 studies)	very serious[n]	very serious[o]	not serious	serious[c]	none	⊕◯◯◯<br>Very low	3615	5796	-	The mean MUAC (cm) - SQ LNS was **0**	MD **0.1 higher** (0.03 higher to 0.17 higher)
**Iron deficiency anemia - SQ LNS**											
2643<br>(3 studies)	serious[p]	very serious[b]	not serious	serious[f]	none	⊕◯◯◯<br>Very low	-/1126	-/1517	**RR 0.46** (0.26 to 0.81)	0 per 1,000	**0 fewer per 1,000** (from 0 fewer to 0 fewer)
**Anemia - Alternative food**											
62<br>(1 study)	serious[q]	not serious	not serious	very serious[r]	none	⊕◯◯◯<br>Very low	-/28	-/34	**RR 0.52** (0.33 to 0.81)	0 per 1,000	**0 fewer per 1,000** (from 0 fewer to 0 fewer)
**Anemia - Fortified Blended Food**											
5511<br>(5 studies)	very serious[s]	very serious[t]	not serious	serious[c,f]	none	⊕◯◯◯<br>Very low	-/2043	-/3468	**RR 0.74** (0.62 to 0.87)	0 per 1,000	**0 fewer per 1,000** (from 0 fewer to 0 fewer)
**Hb (g/dl) - Alternative food**											

Child CF compared to control

Certainty assessment							Summary of findings				
62<br>(1 study)	serious[q]	not serious	not serious	very serious[r,u]	none	⊕◯◯◯<br>Very low	28	34	-	-	SMD **0.35 higher**<br>(0.02 higher to 0.69 higher)

Hb (g/dl) - Fortified Blended Food

2727<br>(6 studies)	very serious[v]	serious[w]	not serious	not serious	none	⊕◯◯◯<br>Very low	1351	1376	-	-	SMD **0.64 higher**<br>(0.29 higher to 1 higher)

Diarrhea - Alternative food

148<br>(1 study)	not serious	not serious	not serious	serious[u,x]	none	⊕⊕⊕◯<br>Moderate	-/73	-/75	**RR 2.04**<br>(1.07 to 3.87)	0 per 1,000	**0 fewer per 1,000**<br>(from 0 fewer to 0 fewer)

Mean Diarrheal episodes - SQ LNS

2556<br>(1 study)	very serious[y]	not serious	not serious	serious[c,u]	none	⊕◯◯◯<br>Very low	838	1718	-	The mean mean Diarrheal episodes - SQ LNS was **0**	MD **0.05 higher**<br>(0.04 higher to 0.05 higher)

Skin disease - Alternative food

148<br>(1 study)	not serious	not serious	not serious	serious[u,x]	none	⊕⊕⊕◯<br>Moderate	-/73	-/75	**RR 0.56**<br>(0.32 to 0.98)	0 per 1,000	**0 fewer per 1,000**<br>(from 0 fewer to 0 fewer)

URTI - SQ-LNS

2556 (1 study)	very serious[y]	not serious	not serious	serious[c,u]	none	⊕◯◯◯ Very low	-/838	-/1718	**RR 0.87** (0.77 to 0.98)	0 per 1,000	**0 fewer per 1,000** (from 0 fewer to 0 fewer)

Death - Commercially Produced Ready to Use Supplementary Food

7879 (4 studies)	very serious[z]	not serious	not serious	serious[c,f]	none	⊕◯◯◯ Very low	-/2777	-/5102	**RR 0.43** (0.20 to 0.94)	0 per 1,000	**0 fewer per 1,000** (from 0 fewer to 0 fewer)

CI: confidence interval; **MD:** mean difference; **RR:** risk ratio; **SMD:** standardised mean difference

Explanations

a. Downgrade by two levels: among 11 studies, nine were RCTs studies of which six had an overall high risk of bias, two had unclear and one had an overall low risk of bias. Among non-RCTs, one had an overall high risk of bias and other had a low risk of bias.
b. Downgrade by two levels: high heterogeneity exists (P < 0.00001).
c. Downgrade by one level: number of participants vary between the groups.
d. Downgrade by one level: among four studies two had an overall high risk of bias and two had an overall low risk of bias.
e. Downgrade by two levels: among seven studies, five were RCTs studies of which four had an overall high risk of bias and one had an overall low risk of bias. Among non-RCTs, one had an overall high risk of bias and other had a low risk of bias.
f. Downgrade by one level: does include appreciable harm or benefit.
g. Downgrade by two levels: all the studies had an overall high risk of bias.
h. Downgrade by two levels: among 13 studies, 11 were RCTs studies of which five had an overall high risk of bias, four had unclear and two had an overall low risk of bias. Among non-RCTs, two studies had an overall low risk of bias.
i. Downgrade by two levels: among 11 studies, eight were RCTs studies of which five had an overall high risk of bias, two had unclear and one had an overall low risk of bias. Among non-RCTs, one had an overall high risk of bias and two had a low risk of bias.
j. Downgrade by two levels: high heterogeneity exists (P = 0.007).
k. Downgrade by two levels: out of three studies, two studies had an overall high risk of bias, and one had an overall low risk of bias.
l. Downgrade by two levels: among 12 studies, 10 were RCTs studies of which seven had an overall high risk of bias, two had unclear and one had an overall low risk of bias. Among non-RCTs, one had an overall high risk of bias and other had a low risk of bias.
m. Downgrade by two levels: among three studies, two had an overall high risk of bias and one had an overall low risk of bias.
n. Downgrade by two levels: among eight studies, six were RCTs studies of which four had an overall high risk of bias and two had unclear risk of bias. Among non-RCTs, one had an overall high risk of bias and other had a low risk of bias.
o. Downgrade by two levels: high heterogeneity exists (P = 0.001).
p. Downgrade by one level: among three studies, two were RCTs of which one had an overall high risk of bias, and one had an unclear risk of bias, while one was a non-RCT which had an overall low risk of bias.
q. Downgrade by one level: the study had an overall unclear risk of bias.
r. Downgrade by two levels: number of participants are <100.
s. Downgrade by two levels: among five studies, three RCTs had an overall high risk of bias, while among two non-RCTs, one had an overall high risk of bias and other had a low risk of bias.
t. Downgrade by two levels: high heterogeneity exists (P = 0.002).
u. Downgrade by one level: outcome included only one study.

v. Downgrade by two levels: among six studies, three RCTs had an overall high risk of bias and one RCT had an unclear risk of bias, while among two non-RCTs, one had an overall high risk of bias and other had a low risk of bias.
w. Downgrade by one level: high heterogeneity exists (P = 0.02).
x. Downgrade by one level: number of participants are <300.
y. Downgrade by two levels: the study had an overall high risk of bias.
z. Downgrade by two levels: among four studies, three had an overall high risk of bias and one had an overall unclear risk of bias.

S3.5.B. GRADE tables for CF supplementation vs control, by food security status

Child CF compared to control for (subgroup by food security)

Certainty assessment							Summary of findings				
Participants (studies) Follow-up	Risk of bias	Inconsistency	Indirectness	Imprecision	Publication bias	Overall certainty of evidence	Study event rates (%)		Relative effect (95% CI)	Anticipated absolute effects	
							With control	With Child CF		Risk with control	Risk difference with Child CF
**Stunting - Food insecure**											
20895 (22 Studies)	very serious[a]	very serious[b]	not serious	serious[c]	none	⊕◯◯◯ Very low	-/8724	-/12171	**RR 0.91** (0.83 to 1.00)	0 per 1,000	**0 fewer per 1,000** (from 0 fewer to 0 fewer)
**Stunting - Food secure**											
3363 (3 Studies)	very serious[d]	serious[e]	not serious	serious[c,f]	none	⊕◯◯◯ Very low	-/968	-/2395	**RR 0.62** (0.44 to 0.88)	0 per 1,000	**0 fewer per 1,000** (from 0 fewer to 0 fewer)
**Wasting - Food insecure**											
27987 (26 Studies)	very serious[g]	not serious	not serious	serious[c]	none	⊕◯◯◯ Very low	-/11453	-/16534	**RR 0.87** (0.81 to 0.93)	0 per 1,000	**0 fewer per 1,000** (from 0 fewer to 0 fewer)
**HAZ - Food insecure**											

Child CF compared to control for (subgroup by food security)

Certainty assessment							Summary of findings				
20287<br>(27 Studies)	very serious[h]	very serious[b]	not serious	serious[c]	none	⊕◯◯◯<br>Very low	7422	12865	-	The mean HAZ - Food insecure was **0**	MD **0.18 higher**<br>(0.03 higher to 0.33 higher)

WAZ - Food insecure

22239<br>(27 Studies)	very serious[i]	very serious[b]	not serious	serious[c]	none	⊕◯◯◯<br>Very low	9028	13211	-	The mean WAZ - Food insecure was **0**	MD **0.09 higher**<br>(0.04 higher to 0.15 higher)

Height - Food insecure

14390<br>(15 Studies)	very serious[j]	very serious[b]	not serious	serious[c]	none	⊕◯◯◯<br>Very low	4664	9726	-	The mean height - Food insecure was **0**	MD **0.21 higher**<br>(0.07 higher to 0.35 higher)

MUAC (cm) - Food insecure

10140<br>(11 Studies)	very serious[k]	very serious[l]	not serious	serious[c]	none	⊕◯◯◯<br>Very low	3949	6191	-	The mean MUAC (cm) - Food insecure was **0**	MD **0.12 higher**<br>(0.05 higher to 0.18 higher)

Iron deficiency anemia - Food secure

514<br>(1 Study)	serious[m]	not serious	not serious	serious[f]	none	⊕⊕◯◯<br>Low	-/201	-/313	**RR 0.24**<br>(0.14 to 0.42)	0 per 1,000	**0 fewer per 1,000**<br>(from 0 fewer to 0 fewer)

Anemia - Food insecure

Child CF compared to control for (subgroup by food security)

Certainty assessment							Summary of findings				
8322 (12 Studies)	very serious[n]	very serious[b]	not serious	not serious	none	⊕◯◯◯ Very low	-/3530	-/4792	**RR 0.86** (0.76 to 0.97)	0 per 1,000	**0 fewer per 1,000** (from 0 fewer to 0 fewer)
**Anemia - Food secure**											
3761 (3 Studies)	very serious[o]	not serious	not serious	serious[c]	none	⊕◯◯◯ Very low	-/1104	-/2657	**RR 0.64** (0.60 to 0.67)	0 per 1,000	**0 fewer per 1,000** (from 0 fewer to 0 fewer)
**Hb (g/dl) - Food insecure**											
7901 (12 Studies)	very serious[p]	very serious[b]	not serious	not serious	none	⊕◯◯◯ Very low	3311	4590	-	-	SMD **0.59 higher** (0.04 higher to 1.15 higher)
**Hb (g/dl) - Food secure**											
863 (3 Studies)	very serious[o]	not serious	not serious	not serious	none	⊕⊕◯◯ Low	364	499	-	-	SMD **0.49 higher** (0.28 higher to 0.71 higher)
**Diarrhea - Food secure**											
148 (1 Study)	not serious	not serious	not serious	serious[q,r]	none	⊕⊕⊕◯ Moderate	-/73	-/75	**RR 2.04** (1.07 to 3.87)	0 per 1,000	**0 fewer per 1,000** (from 0 fewer to 0 fewer)
**Skin disease - Food secure**											
148 (1 Study)	not serious	not serious	not serious	serious[q,r]	none	⊕⊕⊕◯ Moderate	-/73	-/75	**RR 0.56** (0.32 to 0.98)	0 per 1,000	**0 fewer per 1,000** (from 0 fewer to 0 fewer)

CI: confidence interval; **MD:** mean difference; **RR:** risk ratio; **SMD:** standardised mean difference

Explanations

a. Downgrade by two levels: Among 22 studies, 19 were RCTs of which nine had an overall high risk of bias, six had unclear and three had an overall low risk of bias. Among non-RCTs, all the three studies had an overall low risk of bias.
b. Downgrade by two levels: heterogeneity exits (P = <0.00001)
c. Downgrade by one level: number of participants vary between the groups.
d. Downgrade by two levels: among three studies, two were RCTs studies of which one had an overall high risk of bias, and one had an overall low risk of bias. Among non-RCT, the study had an overall high risk of bias.
e. Downgrade by one level: heterogeneity exits (P = 0.03)
f. Downgrade by one level: does include appreciable harm or benefit.
g. Downgrade by two levels: Among 26 studies, 23 were RCTs of which 11 had an overall high risk of bias, eight had an unclear risk of bias and four had low risk of bias. Among non-RCTs, all three studies had a low risk of bias.
h. Downgrade by two levels: Among 27 studies, 22 were RCTs of which 11 had an overall high risk of bias, eight had an unclear risk of bias and three had low risk of bias. Among non-RCTs, three studies had an overall low risk of bias, one had a high risk of bias, and one had some concerns.
i. Downgrade by two levels: Among 26 studies, 22 were RCTs of which 11 had an overall high risk of bias, eight had an unclear risk of bias and three had low risk of bias. Among non-RCTs, two studies had an overall low risk of bias, one had high risk of bias and one had some concerns.
j. Downgrade by two levels: Among 15 studies, 13 were RCTs of which eight had an overall high risk of bias, three had an unclear risk of bias and two had low risk of bias. Among non-RCTs, one study had an overall low risk of bias, and one had some concerns.
k. Downgrade by two levels: Among 11 studies, nine were RCTs of which eight had an overall high risk of bias, and one had an unclear risk of bias. Among non-RCTs, one study had an overall low risk of bias, and one had some concerns.
l. Downgrade by two levels: heterogeneity exits (P = 0.0006)
m. Downgrade by one level: The study has an overall unclear risk of bias.
n. Downgrade by two levels: Among 12 studies, 10 were RCTs of which seven had an overall high risk of bias, and three had an unclear risk of bias. Among non-RCTs, two studies had an overall low risk of bias.
o. Downgrade by two levels: among RCTs, one study had an overall high risk of bias, and one study had an overall unclear risk of bias, while the non-RCT had an overall high risk of bias.
p. Downgrade by two levels: Among 12 studies, 10 were RCTs of which six had an overall high risk of bias and four had an unclear risk of bias. Among non-RCTs, two studies had an overall low risk of bias.
q. Downgrade by one level: number of participants <300.
r. Downgrade by one level: only one study included under this outcome.

S3.5.C. GRADE tables for CF education vs control

IYCF Education compared to control

Certainty assessment							Summary of findings		
	Risk of bias	Inconsistency	Indirectness	Imprecision	Publication bias	Overall certainty	Study event rates (%)		Anticipated absolute effects

IYCF Education compared to control

Certainty assessment							Summary of findings					
**Participants (studies) Follow-up**						**of evidence**	**With placebo**	**With IYCF Education (RCTs)**	**Relative effect (95% CI)**	**Risk with placebo**	**Risk difference with IYCF Education**
**LBW**												
1049 (2 Studies)	very serious[a]	not serious	not serious	serious[b]	none	⊕◯◯◯ Very low	-/536	-/513	**RR 0.47** (0.26 to 0.85)	0 per 1,000	**0 fewer per 1,000** (from 0 fewer to 0 fewer)
**Stunting**												
25795 (25 Studies)	very serious[c]	very serious[d]	not serious	serious[e]	none	⊕◯◯◯ Very low	-/15352	-/10443	**RR 0.90** (0.84 to 0.96)	0 per 1,000	**0 fewer per 1,000** (from 0 fewer to 0 fewer)
**Prevalence of Severe Wasting**												
906 (2 Studies)	very serious[a]	not serious	not serious	serious[b,e]	none	⊕◯◯◯ Very low	-/123	-/783	**RR 0.14** (0.03 to 0.74)	0 per 1,000	**0 fewer per 1,000** (from 0 fewer to 0 fewer)
**Prevalence of Underweight**												
23176 (21 Studies)	very serious[f]	very serious[g]	not serious	serious[e]	none	⊕◯◯◯ Very low	-/14019	-/9157	**RR 0.87** (0.78 to 0.97)	0 per 1,000	**0 fewer per 1,000** (from 0 fewer to 0 fewer)
**Change in HAZ**												
7457 (20 Studies)	very serious[h]	very serious[g]	not serious	not serious	none	⊕◯◯◯ Very low	3735	3722	-	The mean change in HAZ was **0**	MD **0.2 higher** (0.12 higher to 0.28 higher)

IYCF Education compared to control

Certainty assessment							Summary of findings				
**Change in WHZ**											
5260 (16 Studies)	very serious[i]	very serious[g]	not serious	not serious	none	⨁◯◯◯ Very low	2648	2612	-	The mean change in WHZ was **0**	MD **0.09 higher** (0.01 higher to 0.17 higher)
**Change in WAZ**											
5856 (17 Studies)	very serious[j]	very serious[g]	not serious	not serious	none	⨁◯◯◯ Very low	2898	2958	-	The mean change in WAZ was **0**	MD **0.18 higher** (0.1 higher to 0.27 higher)
**Change in weight**											
4176 (14 Studies)	very serious[k]	very serious[g]	not serious	not serious	none	⨁◯◯◯ Very low	1980	2196	-	The mean change in weight was **0**	MD **0.2 higher** (0.07 higher to 0.34 higher)
**Anaemia**											
1252 (3 Studies)	very serious[l]	very serious[m]	not serious	serious[b]	none	⨁◯◯◯ Very low	-/651	-/601	**RR 0.84** (0.60 to 1.18)	0 per 1,000	**0 fewer per 1,000** (from 0 fewer to 0 fewer)
**Respiratory illness**											
1588 (5 Studies)	very serious[n]	not serious	not serious	serious[b]	none	⨁◯◯◯ Very low	-/800	-/788	**RR 0.73** (0.60 to 0.90)	0 per 1,000	**0 fewer per 1,000** (from 0 fewer to 0 fewer)

CI: confidence interval; **MD:** mean difference; **RR:** risk ratio

Explanations

a. Downgrade by two levels: both the studies had an overall high risk of bias.
b. Downgrade by one level: does include appreciable harm or benefit.

c. Downgrade by two levels: Among 25 studies, 15 were RCTs, of which nine had an overall high risk of bias, five had an unclear risk of bias and one had a low risk of bias. Among non-RCTs, five had an overall high risk of bias, four had an unclear risk of bias and one had a low risk of bias.
d. Downgrade by two levels: high heterogeneity ($P < 0.0001$).
e. Downgrade by one level: number of participants vary between the groups.
f. Downgrade by two levels: Among 21 studies, 12 were RCTs of which seven had an overall high risk of bias, four had an unclear risk of bias and one had low risk of bias. Among non-RCTs, four studies had an overall high risk of bias, one had a low risk of bias, and four studies had some concerns.
g. Downgrade by two levels: high heterogeneity ($P < 0.00001$).
h. Downgrade by two levels: Among 20 studies, 15 were RCTs of which eight had an overall high risk of bias, six had an unclear risk of bias and one had low risk of bias. Among non-RCTs, three studies had an overall high risk of bias, one had a low risk of bias, and one had some concerns.
i. Downgrade by two levels: among 16 studies, 14 were RCTs of which, seven had an overall high risk of bias, six had unclear risk of bias and one had an overall low risk of bias. Among non-RCTs, one study had a high risk of bias, and one had some concerns.
j. Downgrade by two levels: Among 17 studies, 13 were RCTs of which seven had an overall high risk of bias, one had a low risk of bias, and five had an unclear risk of bias. Among non-RCTs, two had an overall high risk of bias, one had a low risk of bias, and one had some concerns.
k. Downgrade by two levels: Among 14 studies, 10 were RCTs of which seven had an overall high risk of bias, three had an unclear risk of bias and one had low risk of bias. Among non-RCTs, two studies had an overall high risk of bias, one had a low risk of bias, and one had some concerns.
l. Downgrade by two levels: out of three studies, two RCTs have an overall high risk of bias and one non-RCT has some concerns.
m. Downgrade by two levels: high heterogeneity ($P = 0.0003$).
n. Downgrade by two levels: out of five RCTs, four have an overall high risk of bias and one have an unclear risk of bias.

S3.5.D. GRADE tables for CF education vs control, by food security status

IYCF Education compared to control (subgroup by food security)

Certainty assessment							Summary of findings				
Participants (studies) Follow-up	Risk of bias	Inconsistency	Indirectness	Imprecision	Publication bias	Overall certainty of evidence	Study event rates (%)		Relative effect (95% CI)	Anticipated absolute effects	
							With placebo	With IYCF Education (Studies)		Risk with placebo	Risk difference with IYCF Education
**Stunting - Food insecure**											
12852 (20 Studies)	very serious[a]	serious[b]	not serious	not serious	none	⨁◯◯◯ Very low	0/6321 (0.0%)	0/6531 (0.0%)	**RR 0.943** (0.890 to 0.980)	0 per 1,000	**0 fewer per 1,000** (from 0 fewer to 0 fewer)

Prevalence of Wasting - Food secure

IYCF Education compared to control (subgroup by food security)

Certainty assessment							Summary of findings				
12386 (3 Studies)	very serious[c]	not serious	not serious	serious[d,e]	none	⨁◯◯◯ Very low	0/8762 (0.0%)	0/3624 (0.0%)	**RR 0.78** (0.62 to 0.98)	0 per 1,000	**0 fewer per 1,000** (from 0 fewer to 0 fewer)

Prevalence of Underweight - Food insecure

10819 (18 Studies)	very serious[f]	very serious[g]	not serious	not serious	none	⨁◯◯◯ Very low	-/5313	-/5506	**RR 0.90** (0.81 to 1.00)	0 per 1,000	**0 fewer per 1,000** (from 0 fewer to 0 fewer)

Prevalence of Underweight - Food secure

12357 (3 Studies)	very serious[c]	not serious	not serious	serious[d,e]	none	⨁◯◯◯ Very low	0/8706 (0.0%)	0/3651 (0.0%)	**RR 0.71** (0.53 to 0.95)	0 per 1,000	**0 fewer per 1,000** (from 0 fewer to 0 fewer)

<table>
<tr><th></th><th></th><th></th><th></th><th></th><th></th><th></th><th></th><th></th><th></th><th></th><th></th></tr>
<tr><td colspan="12">Change in HAZ - Food insecure</td></tr>
<tr><td>6629 (16 Studies)</td><td>very serious[f]</td><td>very serious[g]</td><td>not serious</td><td>not serious</td><td>none</td><td>⊕◯◯◯
Very low</td><td>3291</td><td>3338</td><td>-</td><td>The mean change in HAZ - Food insecure was 0</td><td>MD 0.16 higher (0.08 higher to 0.24 higher)</td></tr>
<tr><td colspan="12">Change in HAZ - Food secure</td></tr>
<tr><td>828 (4 Studies)</td><td>very serious[h]</td><td>very serious[i]</td><td>not serious</td><td>not serious</td><td>none</td><td>⊕◯◯◯
Very low</td><td>444</td><td>384</td><td>-</td><td>The mean change in HAZ - Food secure was 0</td><td>MD 0.39 higher (0.09 higher to 0.68 higher)</td></tr>
<tr><td colspan="12">Change in WAZ - Food insecure</td></tr>
<tr><td>5480 (14 Studies)</td><td>serious[j]</td><td>very serious[g]</td><td>not serious</td><td>not serious</td><td>none</td><td>⊕◯◯◯
Very low</td><td>2713</td><td>2767</td><td>-</td><td>The mean change in WAZ - Food insecure was 0</td><td>MD 0.17 higher (0.09 higher to 0.26 higher)</td></tr>
<tr><td colspan="12">Change in WAZ - Food secure</td></tr>
<tr><td>376 (3 Studies)</td><td>serious[k]</td><td>not serious</td><td>not serious</td><td>not serious</td><td>none</td><td>⊕⊕⊕◯
Moderate</td><td>185</td><td>191</td><td>-</td><td>The mean change in WAZ - Food secure was 0</td><td>MD 0.24 higher (0.09 higher to 0.38 higher)</td></tr>
<tr><td colspan="12">Change in Height - Food secure</td></tr>
<tr><td>304 (2 Studies)</td><td>very serious[l]</td><td>not serious</td><td>not serious</td><td>serious[m]</td><td>none</td><td>⊕◯◯◯
Very low</td><td>149</td><td>155</td><td>-</td><td>The mean change in Height - Food secure was 0</td><td>MD 0.9 higher (0.26 higher to 1.55 higher)</td></tr>
<tr><td colspan="12">Change in weight - Food insecure</td></tr>
</table>

3872 (12 Studies)	very serious[n]	very serious[g]	not serious	not serious	none	⊕◯◯◯ Very low	1831	2041	-	The mean change in weight - Food insecure was **0**	MD **0.2 higher** (0.05 higher to 0.35 higher)
**Anaemia - Food insecure**											
432 (1 Study)	very serious[o]	not serious	not serious	serious[d]	none	⊕◯◯◯ Very low	0/214 (0.0%)	0/218 (0.0%)	**RR 0.74** (0.62 to 0.88)	0 per 1,000	**0 fewer per 1,000** (from 0 fewer to 0 fewer)
**Diarrhea - Food secure**											
397 (1 Study)	very serious[o]	not serious	not serious	serious[d]	none	⊕◯◯◯ Very low	0/234 (0.0%)	0/163 (0.0%)	**RR 0.67** (0.51 to 0.90)	0 per 1,000	**0 fewer per 1,000** (from 0 fewer to 0 fewer)
**Respiratory illness - Food secure**											
397 (1 Study)	very serious[o]	not serious	not serious	serious[d]	none	⊕◯◯◯ Very low	0/234 (0.0%)	0/163 (0.0%)	**RR 0.63** (0.46 to 0.85)	0 per 1,000	**0 fewer per 1,000** (from 0 fewer to 0 fewer)

CI: confidence interval; **MD:** mean difference; **RR:** risk ratio

Explanations

a. Downgrade by two levels: Among 20 studies, 14 were RCTs of which nine had an overall high risk of bias, four had an unclear risk of bias and one had low risk of bias. Among non-RCTs, one study had an overall high risk of bias, two had a low risk of bias and three had some concerns
b. Downgrade by one level: heterogeneity exists (P= 0.07)
c. Downgrade by two levels: among three non-RCTs, one study had an overall risk of bias of some concerns, while the other two studies had a high risk of bias
d. Downgrade by one level: does include appreciable harm or benefit
e. Downgrade by one level: number of participants vary between the groups
f. Downgrade by two levels: Among 18 studies, 14 were RCTs, of which nine had an overall high risk of bias, four had an unclear risk of bias and one had low risk of bias. Among non-RCTs, two studies had an overall high risk of bias, one had a low risk of bias and one had some concerns
g. Downgrade by two levels: heterogeneity exists (P <0.00001)
h. Downgrade by two levels: among four studies, three were RCTs of which one study had an overall high risk of bias, while the other two studies had an unclear risk of bias. The one non-RCT had an overall high risk of bias
i. Downgrade by two levels: heterogeneity exists (P= 0.002)
j. Downgrade by two levels: Among 14 studies, 10 were RCTs, of which six had an overall high risk of bias, three had an unclear risk of bias and one had low risk of bias. Among non-RCTs, two studies had an overall high risk of bias, one had a low risk of bias and one had some concerns
k. Downgrade by one level: among three studies one study had an overall high risk of bias, while the other two studies had an unclear risk of bias
l. Downgrade by two levels: among two studies one study had an overall high risk of bias, while the other study had an unclear risk of bias
m. Downgrade by one level: number of participants are <300

n. Downgrade by two levels: Among 12 studies, nine were RCTs, of which seven had an overall high risk of bias, and two had an unclear risk of bias. Among non-RCTs, one study had an overall high risk of bias, one had a low risk of bias and one had some concerns
o. Downgrade by two levels: the study has an overall high risk of bias

S3.5.E. Grade tables for CF education vs control, undernourished children

IYCF Education (malnourished children) compared to control

Certainty assessment							Summary of findings				
Participants (studies) Follow-up	Risk of bias	Inconsistency	Indirectness	Imprecision	Publication bias	Overall certainty of evidence	Study event rates (%)		Relative effect (95% CI)	Anticipated absolute effects	
							With placebo	With IYCF Education malnourished children		Risk with placebo	Risk difference with IYCF Education malnourished children
**Change in WHZ**											
100 (1 RCT)	serious[a]	not serious	not serious	very serious[b,c]	none	⨁◯◯◯ Very low	50	50	-	The mean change in WHZ was **0**	MD **0.34 higher** (0.27 higher to 0.41 higher)
**Change in WAZ**											
100 (1 RCT)	serious[a]	not serious	not serious	very serious[b,c]	none	⨁◯◯◯ Very low	50	50	-	The mean change in WAZ was **0**	MD **0.35 higher** (0.29 higher to 0.41 higher)
**Change in HAZ**											
100 (1 RCT)	serious[a]	not serious	not serious	very serious[b,c]	none	⨁◯◯◯ Very low	50	50	-	The mean change in HAZ was **0**	MD **0.35 higher** (0.29 higher to 0.41 higher)

CI: confidence interval; **MD:** mean difference

Explanations

a. Downgrade by one level: the study has an overall unclear risk of bias
b. Downgrade by two levels: number of participants </= 100
c. Downgrade by one level: only one study included under the outcome

S3.6. Forest Plots

Comparison 1: Complementary food provision versus control

HAZ

Study or Subgroup	Mean Difference	SE	Child complementary feeding Total	Control Total	Weight	Mean Difference IV, Random, 95% CI
**1.1.1 Alternative food**						
Bauserman 2015 (1)	0.1	0.3485	35	36	11.7%	0.10 [−0.58, 0.78]
Iannoti 2017 (2)	0.32	0.1949	75	73	24.5%	0.32 [−0.06, 0.70]
Omer 2022 (3)	0.54	0.2082	61	61	22.9%	0.54 [0.13, 0.95]
Stewart 2019 (4)	0.02	0.0854	290	305	41.0%	0.02 [−0.15, 0.19]
**Subtotal (95% CI)**			**461**	**475**	**100.0%**	**0.22 [−0.05, 0.49]**
Heterogeneity: $Tau^2 = 0.04$; $Chi^2 = 6.49$, df = 3 (P = 0.09); $I^2 = 54\%$						
Test for overall effect: Z = 1.62 (P = 0.10)						
**1.1.2 Fortified Blended Food**						
Akalu 2010 (5)	0.2	0.126	106	105	7.7%	0.20 [−0.05, 0.45]
Akalu 2010 (6)	0.01	0.2103	61	65	5.8%	0.01 [−0.40, 0.42]
Borg 2020 (7)	0.66	0.1495	59	77	7.1%	0.66 [0.37, 0.95]
Chipili 2022 (8)	1.36	0.1417	100	86	7.3%	1.36 [1.08, 1.64]
Christian 2015 (9)	0.01	0.0141	513	843	9.4%	0.01 [−0.02, 0.04]
Ghosh 2020 (10)	0.046	0.0609	260	262	8.9%	0.05 [−0.07, 0.17]
Lutter 2008 (11)	0.27	0.121	170	149	7.8%	0.27 [0.03, 0.51]
Mangani 2015 (12)	−0.07	0.073	183	185	8.7%	−0.07 [−0.21, 0.07]
Oelofse 2003 (13)	−0.22	0.3421	16	14	3.6%	−0.22 [−0.89, 0.45]
Phu 2012 (14)	0.11	0.1142	114	64	7.9%	0.11 [−0.11, 0.33]
Phu 2012 (15)	0.16	0.0931	135	64	8.4%	0.16 [−0.02, 0.34]
Samuel–2018 (16)	0.48	0.0546	1025	1052	9.0%	0.48 [0.37, 0.59]
Sazawal 2014 (17)	0.16	0.0908	59	53	8.4%	0.16 [−0.02, 0.34]
**Subtotal (95% CI)**			**2801**	**3019**	**100.0%**	**0.25 [0.09, 0.41]**
Heterogeneity: $Tau^2 = 0.07$; $Chi^2 = 182.78$, df = 12 (P < 0.00001); $I^2 = 93\%$						
Test for overall effect: Z = 3.10 (P = 0.002)						
**1.1.3 Locally Produced Ready to Use Supplementary Food**						
Agapova 2017 (18)	0.03	0.0704	119	48	1.9%	0.03 [−0.11, 0.17]
Agapova 2017 (19)	0.07	0.0689	116	48	1.9%	0.07 [−0.07, 0.21]
Borg 2020 (20)	0.13	0.133	76	77	0.5%	0.13 [−0.13, 0.39]
Christian 2015 (21)	0.04	0.0141	503	421	46.2%	0.04 [0.01, 0.07]
Christian 2015 (22)	0.03	0.0141	513	422	46.2%	0.03 [0.00, 0.06]
Mangani 2015 (23)	0.03	0.0766	188	92	1.6%	0.03 [−0.12, 0.18]
Mangani 2015 (24)	0.08	0.0723	191	93	1.8%	0.08 [−0.06, 0.22]
**Subtotal (95% CI)**			**1706**	**1201**	**100.0%**	**0.04 [0.02, 0.06]**
Heterogeneity: $Tau^2 = 0.00$; $Chi^2 = 1.38$, df = 6 (P = 0.97); $I^2 = 0\%$						
Test for overall effect: Z = 3.84 (P = 0.0001)						
**1.1.4 Commercially Produced Ready to Use Supplementary Food**						
Christian 2015 (25)	0.02	0.0141	896	843	53.3%	0.02 [−0.01, 0.05]
Huybregts 2012 (26)	0.27	0.111	386	284	27.2%	0.27 [0.05, 0.49]
Santos 2005 (27)	−0.02	0.1489	91	81	19.5%	−0.02 [−0.31, 0.27]
**Subtotal (95% CI)**			**1373**	**1208**	**100.0%**	**0.08 [−0.08, 0.24]**
Heterogeneity: $Tau^2 = 0.01$; $Chi^2 = 5.08$, df = 2 (P = 0.08); $I^2 = 61\%$						
Test for overall effect: Z = 0.98 (P = 0.33)						
**1.1.5 SQ–LNS**						
Addo 2020 (28)	−0.56	0.0036	654	653	4.7%	−0.56 [−0.57, −0.55]
Adu–afarwah 2007 (29)	0.26	0.1505	97	81	4.4%	0.26 [−0.03, 0.55]
Adu–Afarwuah 2016 (30)	0.03	0.0761	334	337	4.6%	0.03 [−0.12, 0.18]
Ashorn 2015 (31)	−0.31	0.1084	195	200	4.5%	−0.31 [−0.52, −0.10]
Dewey 2017 (32)	0.08	0.0566	884	408	4.6%	0.08 [−0.03, 0.19]
Dewey 2017 (33)	0.07	0.0472	785	408	4.6%	0.07 [−0.02, 0.16]
Galasso 2019 (34)	0.044	0.0898	139	139	4.6%	0.04 [−0.13, 0.22]
Hess 2015 (35)	0.3	0.0575	1623	523	4.6%	0.30 [0.19, 0.41]
Humphrey 2019 (36)	0.1	0.0805	356	351	4.6%	0.10 [−0.06, 0.26]
Hurley 2021 (37)	−0.19	0.1191	176	191	4.5%	−0.19 [−0.42, 0.04]
Iannotti 2014 (38)	−0.02	0.1726	150	72	4.4%	−0.02 [−0.36, 0.32]
Iannotti 2014 (39)	−0.05	0.1865	126	72	4.3%	−0.05 [−0.42, 0.32]
Maleta 2015 (40)	0.03	0.0699	245	48	4.6%	0.03 [−0.11, 0.17]
Maleta 2015 (41)	−0.93	0.0679	235	48	4.6%	−0.93 [−1.06, −0.80]
Maleta 2015 (42)	0.08	0.0692	222	48	4.6%	0.08 [−0.06, 0.22]
Maleta 2015 (43)	−0.03	0.0727	238	49	4.6%	−0.03 [−0.17, 0.11]
Maleta 2015 (44)	0.03	0.0724	242	48	4.6%	0.03 [−0.11, 0.17]
Muslihah 2016 (45)	0.41	0.0205	59	26	4.6%	0.41 [0.37, 0.45]
Muslihah 2016 (46)	0.77	0.0215	58	25	4.6%	0.77 [0.73, 0.81]
Prendergast 2019 (47)	3.73	0.2138	58	58	4.3%	3.73 [3.31, 4.15]
Smuts 2019 (48)	−0.13	0.1553	151	100	4.4%	−0.13 [−0.43, 0.17]
Smuts 2019 (49)	−0.13	0.145	162	101	4.5%	−0.13 [−0.41, 0.15]
**Subtotal (95% CI)**			**7189**	**3986**	**100.0%**	**0.15 [−0.14, 0.45]**
Heterogeneity: $Tau^2 = 0.50$; $Chi^2 = 7001.58$, df = 21 (P < 0.00001); $I^2 = 100\%$						
Test for overall effect: Z = 1.01 (P = 0.31)						

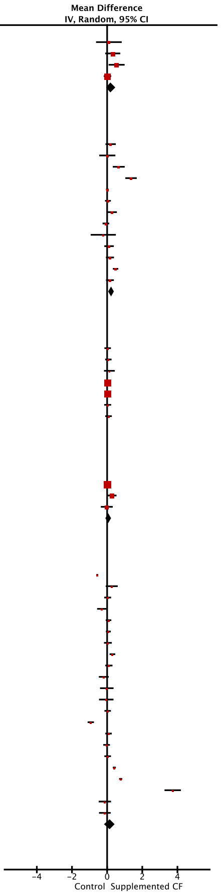

Test for subgroup differences: $Chi^2 = 9.46$, df = 4 (P = 0.05), $I^2 = 57.7\%$

Footnotes

(1) cRCT, Africa, children aged 5 months; supplementation for18 months
(2) Eggs; RCT; Africa; children aged 6–9 months; supplementation for 6 months; endline
(3) cRCT, Ethioipia, Africa, eggs
(4) RCT, Africa, 6–9.9 months age; supplementation for 6 months
(5) Sibu Siri– 13months; RCT: Ethopia; children aged 5–17 months; duration 1 year; endline HAZ
(6) cRCT: Africa; children aged 5–17 months; supplementation for 1 year; Wama Bomaya–13 months; endline HAZ
(7) CSB++ cm; cRCT, Western Pacific Region, 6months of age, supplementation for 6–12 months, change in HAZ
(8) Fish powder; RCT; Africa; children aged 6–7 months; supplementation for 6 months, endline
(9) WSB++ ; cRCT, South–East Asian Region, age–6–18 months; supplementation for 12 months; change in HAZ
(10) RCT, Africa, 6–18 months age, supplementation for intervention 6–12 months
(11) Non–RCT, Region of the Americas, 6–11 months age; supplementation for 11 months; endline
(12) CSB– RCT, Africa, age 6–18 months, suplementation for 12 month, change in HAZ
(13) RCT: South Africa; children aged 6–12 months; supplementation for 6 months; endline
(14) Food complement ; RCT, Western Pacific Region, 5–29 months age; supplementation for 11 months
(15) Fortified flour; RCT, Western Pacific Region, 5–29 months age; supplementation for 11 months
(16) Non RCT, Africa, Rural, 6–23 months age, endline
(17) RCT: India; children aged 6–24 months; supplementation for 6 months; change in HAZ
(18) RCT, cowpea, Africa, age 12–23 months, suplementation for 24 month, change in HAZ at 12 months
(19) RCT, common beans, Africa, age 12–23 months, suplementation for 24 month; change in HAZ at 12 months
(20) RUSF (fish based); cRCT, Western Pacific Region, 6months age, supplementation for 6–12 months, change in HAZ
(21) rice/lentils; cRCT; South East Asia; aged 6–18 months; supplementation for 12 months, change in HAZ
(22) cRCT, chickpeas; South East Asia; aged 6–18 months; supplementation for 12 months
(23) Soy LNS; RCT, Africa, age 6–18 months; supplementation for 12 months, change in HAZ
(24) Milk LNS; RCT, Africa, age 6–18 months; supplementation for 12 months, change in HAZ
(25) Plumpy doz– RCT, South–East Asian Region, age–6–18 months; supplementation for 12 months
(26) Plumpy doz– RCT, South–East Asian Region, age–6–18 months; supplementation for 12 months
(27) milk; non–RCT: Brazil; children aged 6–18 months; change in HAZ
(28) non–RCT; Africa; 6–18 months age, supplementation for 3 months
(29) SQ–LNS Nutributter; qRCT; African region; mothers with >20 wk of gestation; supplementation for 6 months
(30) change in HAZ, maternal and child supplementation; SQ–LNS; RCT; African region; mothers with >20 wk of gestation; supplementation for 12 months
(31) SQLNS; RCT; Malawi; children aged 6–18 months; supplementaion for 6 months, change in HAZ
(32) SQ LNS; cRCT, South–East Asia; mothers and thier children 6–24 months age, supplementation for 18 months, endline
(33) SQ LNS; cRCT, South–East Asia; children 6–24 months age, supplementation for 18 months, endline
(34) SQLNS ; RCT; Madasgar; Children <12 years; supplementation for 12 months
(35) zinc, cRCT, Africa, aged 9–18 months of age, supplementation for 9 months
(36) SQLNS 20 gm–HIV negative, cRCT, Africa, Aged 6–18 month, supplementation for 12 months, endline
(37) non–RCT, Africa; children aged 6–24 months; supplementation for 12 months
(38) LNS; RCT African region; children aged 6–11 months; supplementation for 6 months
(39) SQ LNS; RCT African region; children aged 6–11 months; supplementation for 3 months
(40) 20g no milk; RCT, Africa; aged 6 months, supplementation for 12 months, change in HAZ
(41) 20g milk; RCT, Africa; aged 6 months, supplementation for 12 months, change in HAZ
(42) 10g milk; RCT, Africa; aged 6 months, supplementation for 12 months, change in HAZ
(43) 40g no milk; RCT, Africa; aged 6 months, supplementation for 12 months, change in HAZ
(44) 40g milk; RCT, Africa; aged 6 months, supplementation for 12 months, change in HAZ
(45) Biscuits; non–RCT: Indonesia; Children aged 6–12 months; supplementation for 6 months; at endline
(46) SQ–LNS– non–RCT: Indonesia; Children aged 6–12 months; supplementation for 6 months; at endline
(47) SQLNS–20 gm–HIV positive, cRCT, Africa, Aged 6–18 month, supplementation for 12 months, endline
(48) SQ LNS; RCT, Africa; aged 6–12 months, supplementation for 6 months, endline
(49) SQ LNS plus; RCT, Africa; aged 6–12 months, supplementation for 6 months

Stunting

Study or Subgroup	log[Risk Ratio]	SE	Child complementary feeding Total	Control Total	Weight	Risk Ratio IV, Random, 95% CI
**1.2.1 Alternative Food**						
Bauserman 2015 (1)	−0.0592	0.1059	35	36	34.9%	0.94 [0.77, 1.16]
Bhandari 2001 (2)	−0.0731	0.0944	87	93	43.9%	0.93 [0.77, 1.12]
Iannoti 2017 (3)	−0.3498	0.2347	75	73	7.1%	0.70 [0.44, 1.12]
Omer 2022 (4)	−0.1744	1.1404	61	61	0.3%	0.84 [0.09, 7.85]
Stewart 2019 (5)	0.0153	0.1684	290	305	13.8%	1.02 [0.73, 1.41]
**Subtotal (95% CI)**			**548**	**568**	**100.0%**	**0.93 [0.82, 1.05]**
Heterogeneity: Tau^2 = 0.00; Chi^2 = 1.69, df = 4 (P = 0.79); I^2 = 0%						
Test for overall effect: Z = 1.22 (P = 0.22)						
**1.2.2 Fortified Blended Food**						
Borg 2020 (6)	0.015	0.4733	59	77	5.5%	1.02 [0.40, 2.57]
Chipili 2022 (7)	−0.6157	0.1077	100	86	16.5%	0.54 [0.44, 0.67]
Christian 2015 (8)	0.0001	0.0629	513	843	17.8%	1.00 [0.88, 1.13]
Lutter 2008 (9)	−0.27	0.1485	170	149	15.1%	0.76 [0.57, 1.02]
Mangani 2015 (10)	0.276	0.2579	193	195	10.9%	1.32 [0.79, 2.18]
Samuel-2018 (11)	−0.4608	0.0583	1025	1052	17.9%	0.63 [0.56, 0.71]
Zhang 2016 (12)	−0.7531	0.1171	2150	746	16.2%	0.47 [0.37, 0.59]
**Subtotal (95% CI)**			**4210**	**3148**	**100.0%**	**0.73 [0.56, 0.95]**
Heterogeneity: Tau^2 = 0.10; Chi^2 = 58.87, df = 6 (P < 0.00001); I^2 = 90%						
Test for overall effect: Z = 2.38 (P = 0.02)						
**1.2.3 Locally Produced Ready to Use Supplementary Food**						
Borg 2020 (13)	−0.7979	0.5788	76	77	0.8%	0.45 [0.14, 1.40]
Christian 2015 (14)	−0.0131	0.0744	503	422	46.9%	0.99 [0.85, 1.14]
Christian 2015 (15)	−0.1201	0.0776	513	421	43.2%	0.89 [0.76, 1.03]
Mangani 2015 (16)	−0.0144	0.231	140	69	4.9%	0.99 [0.63, 1.55]
Mangani 2015 (17)	0.127	0.2471	128	69	4.3%	1.14 [0.70, 1.84]
**Subtotal (95% CI)**			**1360**	**1058**	**100.0%**	**0.94 [0.85, 1.04]**
Heterogeneity: Tau^2 = 0.00; Chi^2 = 3.23, df = 4 (P = 0.52); I^2 = 0%						
Test for overall effect: Z = 1.17 (P = 0.24)						
**1.2.4 Commercially Produced Ready to Use Supplementary Food**						
Christian 2015 (18)	−0.0937	0.0561	896	843	57.3%	0.91 [0.82, 1.02]
Huybregts 2012 (19)	−0.129	0.0789	386	284	29.0%	0.88 [0.75, 1.03]
Khan 2020 (20)	−0.0965	0.1144	105	120	13.8%	0.91 [0.73, 1.14]
**Subtotal (95% CI)**			**1387**	**1247**	**100.0%**	**0.90 [0.83, 0.98]**
Heterogeneity: Tau^2 = 0.00; Chi^2 = 0.14, df = 2 (P = 0.93); I^2 = 0%						
Test for overall effect: Z = 2.46 (P = 0.01)						
**1.2.5 SQ LNS**						
Addo 2020 (21)	0.46	0.0691	654	653	8.1%	1.58 [1.38, 1.81]
Adu-Afarwuah 2016 (22)	−0.4286	0.2176	347	350	5.0%	0.65 [0.43, 1.00]
Ashorn 2015 (23)	0.1454	0.1304	214	220	6.9%	1.16 [0.90, 1.49]
Dewey 2017 (24)	−0.1372	0.0839	884	408	7.9%	0.87 [0.74, 1.03]
Dewey 2017 (25)	−0.0332	0.0834	785	408	7.9%	0.97 [0.82, 1.14]
Hess 2015 (26)	−0.3487	0.0764	1623	523	8.0%	0.71 [0.61, 0.82]
Humphrey 2019 (27)	−0.1711	0.1135	356	351	7.3%	0.84 [0.67, 1.05]
Hurley 2021 (28)	0.1205	0.1221	176	191	7.1%	1.13 [0.89, 1.43]
Luby 2018 (29)	−0.2216	0.0736	516	1003	8.1%	0.80 [0.69, 0.93]
Maleta 2015 (30)	−0.277	0.1823	179	35	5.7%	0.76 [0.53, 1.08]
Maleta 2015 (31)	0.1805	0.2858	187	35	3.8%	1.20 [0.68, 2.10]
Maleta 2015 (32)	0.0939	0.2852	195	34	3.8%	1.10 [0.63, 1.92]
Maleta 2015 (33)	0.0452	0.2901	184	35	3.7%	1.05 [0.59, 1.85]
Maleta 2015 (34)	−0.0659	0.2886	207	34	3.7%	0.94 [0.53, 1.65]
Null 2018 (35)	−0.0809	0.0832	496	1096	7.9%	0.92 [0.78, 1.09]
Prendergast 2019 (36)	−0.2231	0.2013	58	58	5.3%	0.80 [0.54, 1.19]
**Subtotal (95% CI)**			**7061**	**5434**	**100.0%**	**0.94 [0.81, 1.09]**
Heterogeneity: Tau^2 = 0.06; Chi^2 = 88.05, df = 15 (P < 0.00001); I^2 = 83%						
Test for overall effect: Z = 0.83 (P = 0.40)						

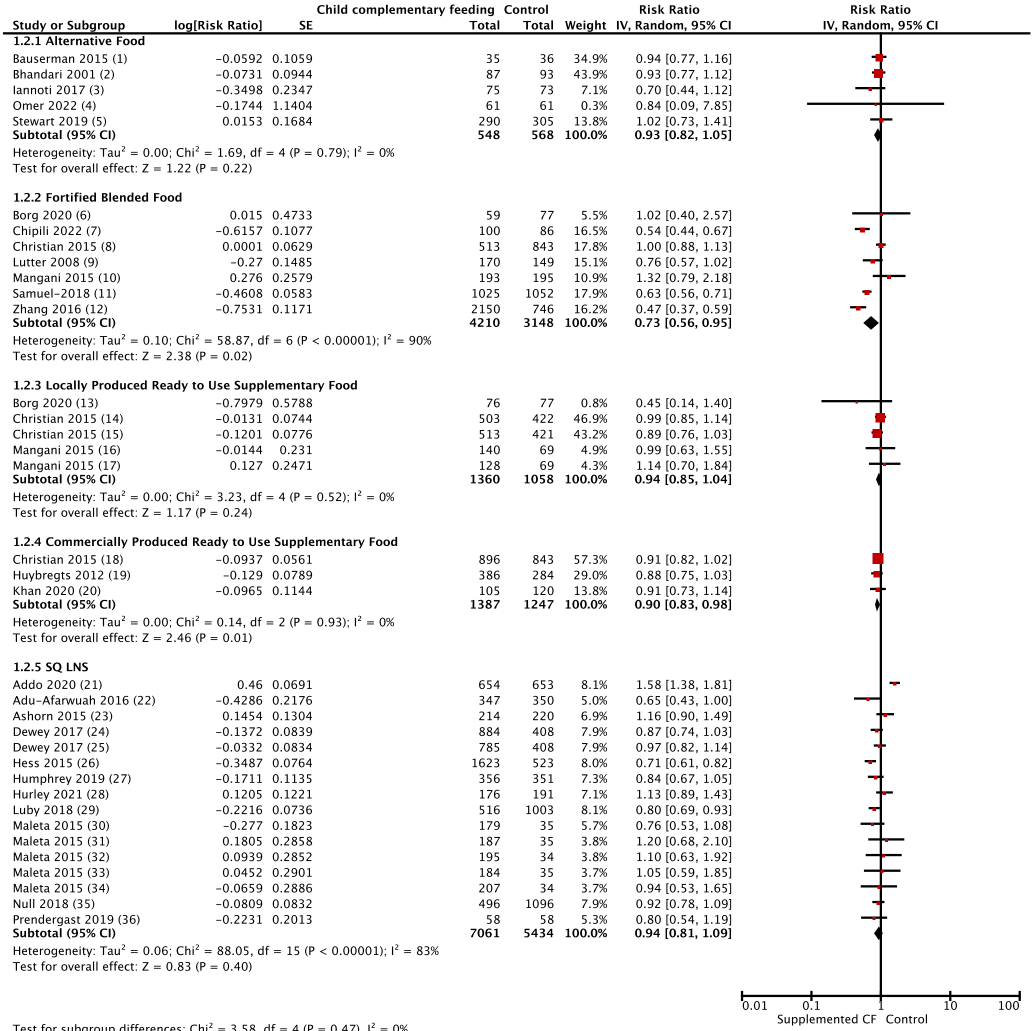

Test for subgroup differences: Chi^2 = 3.58, df = 4 (P = 0.47), I^2 = 0%

Footnotes

(1) cRCT, Africa, children aged 5 months; supplementation for 18 months,
(2) cRCT, South-East Asian Region, 4 and 12 mo of age, supplementation for 6 months intervention
(3) Eggs; RCT; Africa; children aged 6-9 months; supplementation for 6 months
(4) cRCT, Ethioipia, Africa, eggs
(5) RCT, Africa, 6-9.9 months age; supplementation for 6 months
(6) CSB++ cm; RCT, Western Pacific Region, 6months of age, supplementation for 6-12 months
(7) Fish powder; RCT; Africa; children aged 6-7 months; supplementation for 6 months, endline
(8) WSB++ ; cRCT, South-East Asian Region, age-6-18 months; supplementation for 12 months
(9) Non-RCT, Region of the Americas, 6-11 months age; supplementation for 11 months;
(10) CSB- RCT, Africa, age 6-18 months, suplementation for 12 month, severe stunting
(11) Non RCT, Africa, Rural, 6-23 months , endline
(12) RCT; Western Pacific; aged 6-23 months; supplementation for 2 years, endline
(13) RUSF (fish based); cRCT, Western Pacific Region, 6months age, supplementation for 6-12 months
(14) cRCT, rice/lentils; South East Asia; aged 6-18 months; supplementation for 12 months
(15) cRCT, chickpeas; South East Asia; aged 6-18 months; supplementation for 12 months
(16) Milk LNS; RCT, Africa, age 6-18 months; supplementation for 12 months, severe stunting
(17) Soy LNS; RCT, Africa, age 6-18 months; supplementation for 12 months, severe stunting
(18) Plumpy doz- cRCT, South-East Asian Region, age-6-18 months; supplementation for 12 months
(19) Plumpydoz Nutriset 46 g ~247Kcal/ d; RCT; Africa; age 6-36 months, supplementation for 4 months
(20) cRCT, EMRO, age 6-24 months, supplementation for 12 months-MQ LNS
(21) non-RCT; Africa; 6-18 months age, supplementation for 3 months
(22) SQ-LNS; RCT; African region; mothers with >20 wk of gestation; supplementation for 12 months
(23) SQLNS; RCT; Malawi; children aged 6-18 months; supplementaion for 6 months
(24) SQ LNS; RCT, South-East Asia; mother and thier children 6-24 months age, supplementation for 18 months
(25) SQ LNS; RCT, South-East Asia; 6-24 months age, supplementation for 18 months
(26) zinc, cRCT, Africa, aged 9-18 months of age, supplementation for 9 months
(27) SQLNS 20 gm-HIV negative, cRCT, Africa, Aged 6-18 month, supplementation for 12 months, endline
(28) Non-RCT, Africa; 6-24 months of age, Africa; supplementation for 12 months
(29) 20 g SQLNS, cRCT; Bangladesh; 6-24 months age; supplementation for 12 months
(30) 10g milk; RCT, Africa; aged 6 months, supplementation for 12 months
(31) 20g no milk; RCT, Africa; aged 6 months, supplementation for 12 months
(32) 40g no milk; RCT, Africa; aged 6 months, supplementation for 12 months
(33) 20g milk; RCT, Africa; aged 6 months, supplementation for 12 months
(34) 40g milk; RCT, Africa; aged 6 months, supplementation for 12 months
(35) SQLNS-20 gm, RCT, Africa; 6-24 month aged
(36) SQLNS-20 gm-HIV positive, cRCT, Africa, Aged 6-18 month, supplementation for 12 months, endline

Severe Stunting

Study or Subgroup	log[Risk Ratio]	SE	Child complementary feeding Total	Control Total	Weight	Risk Ratio IV, Random, 95% CI
**1.3.1 Fortified Blended Food**						
Mangani 2015 (1)	0.276	0.2579	193	195	100.0%	1.32 [0.79, 2.18]
**Subtotal (95% CI)**			**193**	**195**	**100.0%**	**1.32 [0.79, 2.18]**
Heterogeneity: Not applicable						
Test for overall effect: Z = 1.07 (P = 0.28)						
**1.3.2 Locally Produced Ready to Use Supplementary Food**						
Mangani 2015 (2)	-0.0144	0.231	140	69	53.4%	0.99 [0.63, 1.55]
Mangani 2015 (3)	0.127	0.2471	128	69	46.6%	1.14 [0.70, 1.84]
**Subtotal (95% CI)**			**268**	**138**	**100.0%**	**1.05 [0.76, 1.47]**
Heterogeneity: Tau² = 0.00; Chi² = 0.17, df = 1 (P = 0.68); I² = 0%						
Test for overall effect: Z = 0.31 (P = 0.76)						
**1.3.4 SQLNS**						
Ashorn 2015 (4)	0.0277	0.3013	214	220	6.3%	1.03 [0.57, 1.86]
Humphrey 2019 (5)	-0.048	0.2493	356	351	8.3%	0.95 [0.58, 1.55]
Hurley 2021 (6)	0.6696	0.3046	176	191	6.2%	1.95 [1.08, 3.55]
Luby 2018 (7)	-0.3015	0.1708	516	1003	13.5%	0.74 [0.53, 1.03]
Maleta 2015 (8)	-0.0328	0.2289	248	48	9.4%	0.97 [0.62, 1.52]
Maleta 2015 (9)	-0.2427	0.3935	236	48	4.0%	0.78 [0.36, 1.70]
Maleta 2015 (10)	-0.3341	0.3956	249	48	4.0%	0.72 [0.33, 1.55]
Maleta 2015 (11)	0.0078	0.4161	254	48	3.6%	1.01 [0.45, 2.28]
Maleta 2015 (12)	-0.3261	0.2465	262	48	8.5%	0.72 [0.45, 1.17]
Mangani 2015 (13)	0.2179	0.1077	126	138	20.2%	1.24 [1.01, 1.54]
Null 2018 (14)	-0.1686	0.1803	496	1096	12.7%	0.84 [0.59, 1.20]
Prendergast 2019 (15)	-0.1178	0.449	58	58	3.2%	0.89 [0.37, 2.14]
**Subtotal (95% CI)**			**3191**	**3297**	**100.0%**	**0.96 [0.82, 1.14]**
Heterogeneity: Tau² = 0.02; Chi² = 15.90, df = 11 (P = 0.15); I² = 31%						
Test for overall effect: Z = 0.43 (P = 0.67)						

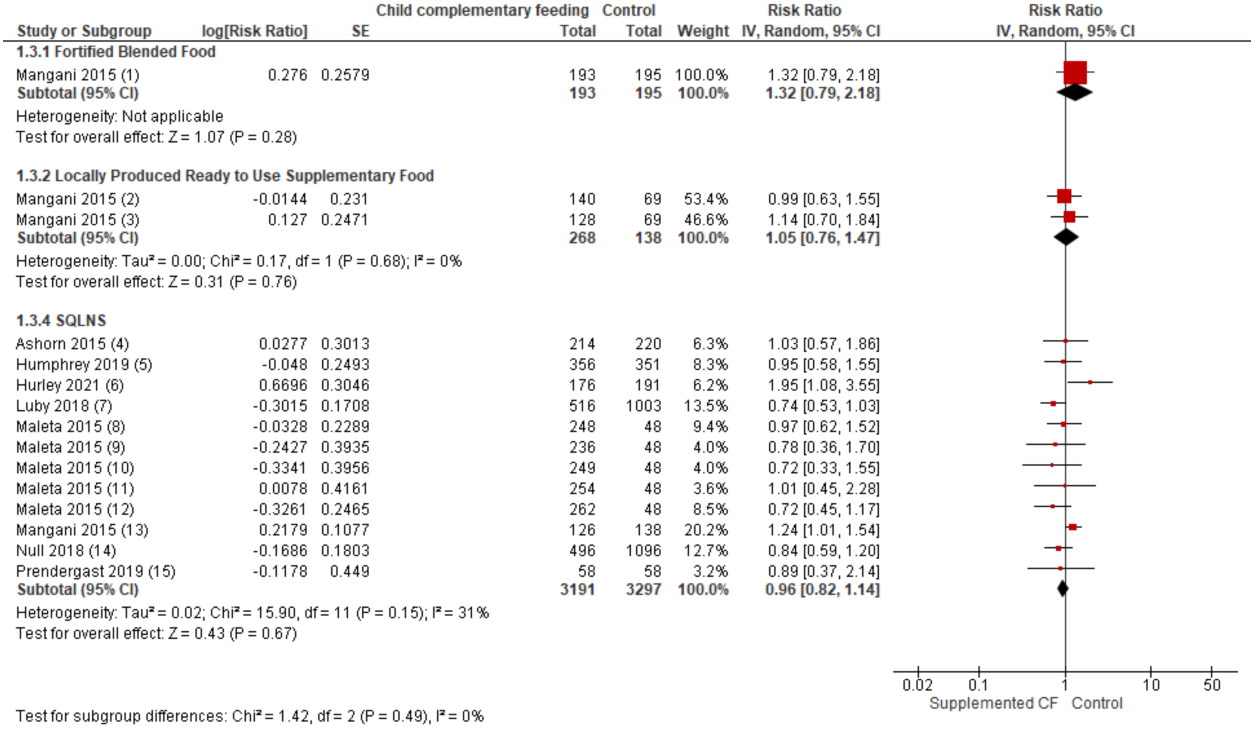

Test for subgroup differences: Chi² = 1.42, df = 2 (P = 0.49), I² = 0%

Footnotes
(1) CSB- RCT, Africa, age 6-18 months, suplementation for 12 month
(2) Milk; RCT, Africa, age 6-18 months, suplementation for 12 month
(3) Soy LNS; RCT, Africa, age 6-18 months, suplementation for 12 month
(4) SQLNS; RCT; Malawi; children aged 6-18 months; supplementaion for 6 months
(5) SQLNS 20 gm-HIV negative, cRCT, Africa, Aged 6-18 month, supplementation for 12 months, endline
(6) non-RCT, Africa; children aged 6-24 months; supplementation for 12 months
(7) 20 g SQLNS, cRCT; Bangladesh; 6-24 months age; supplementation for 12 months
(8) 20g milk; RCT, Africa; aged 6 months, supplementation for 12 months
(9) 10g milk; RCT, Africa; aged 6 months, supplementation for 12 months
(10) 20g no milk; RCT, Africa; aged 6 months, supplementation for 12 months
(11) 40g no milk; RCT, Africa; aged 6 months, supplementation for 12 months
(12) 40g milk; RCT, Africa; aged 6 months, supplementation for 12 months
(13) CSB; RCT, Africa, age 6-18 months, suplementation for 12 month
(14) SQLNS-20 gm, RCT, Africa; 6-24 month aged, duration NR
(15) SQLNS-20 gm-HIV positive, cRCT, Africa, Aged 6-18 month, supplementation for 12 months, endline

WHZ

Study or Subgroup	Mean Difference	SE	Child complementary feeding Total	Control Total	Weight	Mean Difference IV, Random, 95% CI
**1.8.1 Alternative food**						
Bauserman 2015 (1)	−0.2	0.3265	34	35	17.7%	−0.20 [−0.84, 0.44]
Iannoti 2017 (2)	−0.15	0.1356	75	73	27.0%	−0.15 [−0.42, 0.12]
Omer 2022 (3)	0.83	0.1524	61	61	26.3%	0.83 [0.53, 1.13]
Stewart 2019 (4)	0.17	0.0816	290	305	29.0%	0.17 [0.01, 0.33]
**Subtotal (95% CI)**			**460**	**474**	**100.0%**	**0.19 [−0.23, 0.61]**
Heterogeneity: $Tau^2 = 0.15$; $Chi^2 = 25.39$, df = 3 (P < 0.0001); $I^2 = 88\%$						
Test for overall effect: Z = 0.90 (P = 0.37)						
**1.8.2 Fortified Blended Food**						
Addo 2020 (5)	0.34	0.0721	654	653	11.0%	0.34 [0.20, 0.48]
Akalu 2010 (6)	0.34	0.1507	61	65	4.5%	0.34 [0.04, 0.64]
Akalu 2010 (7)	0.03	0.1338	106	105	5.3%	0.03 [−0.23, 0.29]
Borg 2020 (8)	0	0.1378	59	77	5.1%	0.00 [−0.27, 0.27]
Christian 2015 (9)	0.02	0.0141	513	843	18.7%	0.02 [−0.01, 0.05]
Ghosh 2020 (10)	0.01	0.0918	157	158	8.7%	0.01 [−0.17, 0.19]
Lutter 2008 (11)	0.15	0.1038	170	149	7.5%	0.15 [−0.05, 0.35]
Mangani 2015 (12)	−0.03	0.102	183	185	7.7%	−0.03 [−0.23, 0.17]
Oelofse 2003 (13)	−0.31	0.5084	16	14	0.5%	−0.31 [−1.31, 0.69]
Phu 2012 (14)	−0.1	0.1225	114	64	6.1%	−0.10 [−0.34, 0.14]
Phu 2012 (15)	0.02	0.1179	135	64	6.4%	0.02 [−0.21, 0.25]
Samuel-2018 (16)	0.07	0.0476	1172	1137	14.5%	0.07 [−0.02, 0.16]
Sazawal 2014 (17)	0.13	0.1578	59	53	4.2%	0.13 [−0.18, 0.44]
**Subtotal (95% CI)**			**3399**	**3567**	**100.0%**	**0.08 [0.01, 0.15]**
Heterogeneity: $Tau^2 = 0.01$; $Chi^2 = 27.49$, df = 12 (P = 0.007); $I^2 = 56\%$						
Test for overall effect: Z = 2.11 (P = 0.03)						
**1.8.3 Locally Produced Ready to Use Supplementary Food**						
Borg 2020 (18)	0.07	0.1173	76	77	0.9%	0.07 [−0.16, 0.30]
Christian 2015 (19)	0.04	0.0141	513	422	52.1%	0.04 [0.01, 0.07]
Christian 2015 (20)	0	0.0153	503	421	45.4%	0.00 [−0.03, 0.03]
Mangani 2015 (21)	0.09	0.1225	191	92	0.8%	0.09 [−0.15, 0.33]
Mangani 2015 (22)	0.04	0.1225	188	93	0.8%	0.04 [−0.20, 0.28]
**Subtotal (95% CI)**			**1471**	**1105**	**100.0%**	**0.02 [0.00, 0.04]**
Heterogeneity: $Tau^2 = 0.00$; $Chi^2 = 4.19$, df = 4 (P = 0.38); $I^2 = 5\%$						
Test for overall effect: Z = 2.03 (P = 0.04)						
**1.8.4 Commercially Produced Ready to Use Supplementary Food**						
Christian 2015 (23)	0.04	0.0141	896	843	95.5%	0.04 [0.01, 0.07]
Huybregts 2012 (24)	0.04	0.0714	386	284	3.7%	0.04 [−0.10, 0.18]
Santos 2005 (25)	0.03	0.1583	91	81	0.8%	0.03 [−0.28, 0.34]
**Subtotal (95% CI)**			**1373**	**1208**	**100.0%**	**0.04 [0.01, 0.07]**
Heterogeneity: $Tau^2 = 0.00$; $Chi^2 = 0.00$, df = 2 (P = 1.00); $I^2 = 0\%$						
Test for overall effect: Z = 2.90 (P = 0.004)						
**1.8.5 SQ-LNS**						
Addo 2020 (26)	−0.35	0.0408	654	653	6.0%	−0.35 [−0.43, −0.27]
Adu-afarwah 2007 (27)	0.31	0.1656	97	81	2.5%	0.31 [−0.01, 0.63]
Adu-Afarwuah 2016 (28)	−0.2	0.102	323	323	4.0%	−0.20 [−0.40, −0.00]
Ashorn 2015 (29)	−0.3	0.1327	195	200	3.2%	−0.30 [−0.56, −0.04]
Becquey 2019 (30)	−0.08	0.0969	885	896	4.2%	−0.08 [−0.27, 0.11]
Dewey 2017 (31)	0.08	0.0408	884	408	6.0%	0.08 [0.00, 0.16]
Dewey 2017 (32)	0.08	0.0459	785	408	5.8%	0.08 [−0.01, 0.17]
Galasso 2019 (33)	−0.1	0.075	139	139	4.9%	−0.10 [−0.25, 0.05]
Hess 2015 (34)	0.2	0.051	1623	523	5.7%	0.20 [0.10, 0.30]
Humphrey 2019 (35)	0.01	0.0816	355	350	4.7%	0.01 [−0.15, 0.17]
Hurley 2021 (36)	0.01	0.0051	176	191	6.6%	0.01 [0.00, 0.02]
Huybregts 2019 (37)	0.12	0.0408	1154	1159	6.0%	0.12 [0.04, 0.20]
Luby 2018 (38)	0.17	0.0525	516	1004	5.6%	0.17 [0.07, 0.27]
Maleta 2015 (39)	0.11	0.1378	220	49	3.1%	0.11 [−0.16, 0.38]
Maleta 2015 (40)	0.14	0.148	242	48	2.8%	0.14 [−0.15, 0.43]
Maleta 2015 (41)	0.09	0.148	238	48	2.8%	0.09 [−0.20, 0.38]
Maleta 2015 (42)	−0.01	0.148	235	48	2.8%	−0.01 [−0.30, 0.28]
Maleta 2015 (43)	0.06	0.148	245	48	2.8%	0.06 [−0.23, 0.35]
Muslihah 2016 (44)	−0.26	0.0241	58	25	6.4%	−0.26 [−0.31, −0.21]
Muslihah 2016 (45)	0.04	0.024	59	26	6.4%	0.04 [−0.01, 0.09]
Null 2018 (46)	0.03	0.051	496	1097	5.7%	0.03 [−0.07, 0.13]
Prendergast 2019 (47)	−0.11	0.199	59	58	2.0%	−0.11 [−0.50, 0.28]
**Subtotal (95% CI)**			**9638**	**7782**	**100.0%**	**−0.00 [−0.07, 0.06]**
Heterogeneity: $Tau^2 = 0.02$; $Chi^2 = 255.55$, df = 21 (P < 0.00001); $I^2 = 92\%$						
Test for overall effect: Z = 0.11 (P = 0.91)						

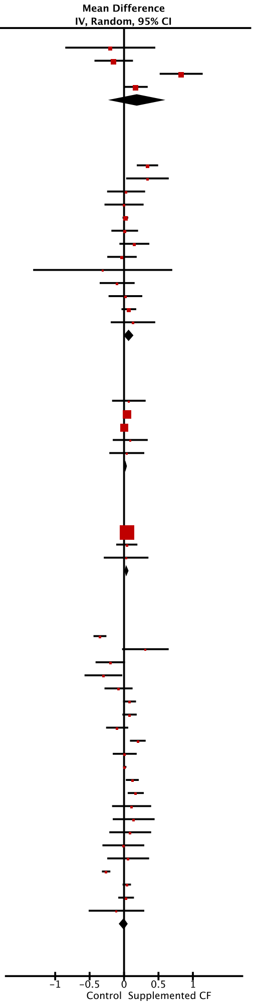

Test for subgroup differences: $Chi^2 = 4.23$, df = 4 (P = 0.38), $I^2 = 5.4\%$

Footnotes

(1) cRCT, Africa, children aged 5 months; supplementation for18 months; endline
(2) Eggs; RCT; Africa; children aged 6–9 months; supplementation for 6 months; endline
(3) cRCT, Ethioipia, Africa, eggs
(4) RCT; Africa, 6 months, egg, WHZ at 6 months
(5) non-RCT; Africa; 6–18 months age, supplementation for 3 months; endline WHZ
(6) cRCT: Africa; children aged 5–17 months; supplementation for 1 year; Wama Bomaya–13months;
(7) RCT: Africa; children aged 5–17 months; supplementation for 1 year; Sibu–Siru–13 months
(8) CSB++; cRCT, Western Pacific Region, 6months age, supplementation for 6–12 months; change in WHZ
(9) WSB++ ; cRCT, South–East Asian Region, age–6–18 months; supplementation for 12 months; change in WHZ
(10) cRCT, Africa, 6–18 months age, supplementation for intervention 6–12 months; endline WHZ
(11) Non–RCT, Region of the Americas, 6–11 months age; supplementation for 11 months
(12) CSB– RCT, Africa, age 6–18 months, suplementation for 12 month; change in WHZ
(13) RCT: South Africa; children aged 6–12 months; supplementation for 6 months; endline
(14) fortified food complement ; RCT, Western Pacific Region, 5–29 months age; supplementation for 11 months; endline WHZ
(15) fortified flour ; RCT, Western Pacific Region, 5–29 months age; supplementation for 11 months; endline WHZ
(16) Non RCT, Africa, 6–23 months age, duration NR; change in WHZ
(17) RCT: India; children aged 6–24 months; supplementation for 6 months; change in WHZ
(18) RUSF (fish based); cRCT, Western Pacific Region, 6months age, supplementation for 6–12 months; change in WHZ
(19) cRCT; chickpeas; South East Asia; aged 6–18 months; supplementation for 12 months; change in WHZ
(20) cRCT; Rice/lentils; South East Asia; aged 6–18 months; supplementation for 12 months; change in WHZ
(21) Milk LNS; RCT, Africa, age 6–18 months; supplementation for 12 months; change in WHZ
(22) Soy LNS; RCT, Africa, age 6–18 months; supplementation for 12 months; change in WHZ
(23) Plumpy doz– RCT, South–East Asian Region, age–6–18 months; supplementation for 12 months; change in WHZ
(24) Plumpydoz Nutriset 46 g ~247Kcal/d; cRCT; Africa; age 6–36 months, supplementation for 4 months; endline WHZ
(25) milk; non–RCT: Brazil; children aged 6–18 months; change in WHZ
(26) non–RCT; Africa; 6–18 months age, supplementation for 3 months
(27) SQ–LNS Nutributter; non–RCT; African region; mothers with >20 wk of gestation; supplementation for 6 months
(28) WHZ was measured at 18 months of intervention–maternal and child; SQ–LNS; RCT; African region; mothers with >20 wk of gestation; supplementation for 6 months; change in
(29) SQLNS; RCT; Malawi; children aged 6–18 months; supplementaion for 6 months; change in WHZ
(30) cRCT; Burkina GFaso, SQLNS, endline
(31) SQ LNS; cRCT, South–East Asia; mothers and thier children 6–24 months age, supplementation for 18 months; endline
(32) SQ LNS; cRCT, South–East Asia; children 6–24 months age, supplementation for 18 months; endline
(33) SQLNS ; cRCT; Madasgar; Children <12 years; supplementation for 12 months; change in WHZ
(34) zinc, cRCT, Africa, aged 9–18 months of age, supplementation for 9 months; endline
(35) SQLNS 20 gm–HIV negative, RCT, Africa, Aged 6–18 month, supplementation for 12 months; endline
(36) non–RCT, Africa; children aged 6–24 months; supplementation for 12 months; change in WHZ
(37) cRCT; Mali, SQLNS, children 6–23 months, endline
(38) 20 g SQLNS, non–RCT; Bangladesh; 6–24 months age; supplementation for 12 months; endline
(39) 10g milk; RCT, Africa; aged 6 months, supplementation for 12 months; change in WHZ
(40) 40g milk; RCT, Africa; aged 6 months, supplementation for 12 months; change in WHZ
(41) 40g no milk; RCT, Africa; aged 6 months, supplementation for 12 months; change in WHZ
(42) 20g milk; RCT, Africa; aged 6 months, supplementation for 12 months; change in WHZ
(43) 20g no milk; RCT, Africa; aged 6 months, supplementation for 12 months; change in WHZ
(44) SQ–LNS– non–RCT: Indonesia; Children aged 6–12 months; supplementation for 6 months; at endline
(45) Biscuit; non–RCT: Indonesia; Children aged 6–12 months; supplementation for 6 months; at endline
(46) SQLNS–20 gm, RCT, Africa; 6–24 month aged, endline
(47) SQLNS–20 gm–HIV positive, RCT, Africa, Aged 6–18 month, supplementation for 12 months; endline

Wasting

Study or Subgroup	log[Risk Ratio]	SE	Child complementary feeding Total	Control Total	Weight	Risk Ratio IV, Random, 95% CI
**1.4.1 Alternative food**						
Bauserman 2015 (1)	−0.2877	0.7281	35	35	12.0%	0.75 [0.18, 3.12]
Bhandari 2001 (2)	0.0677	0.3304	87	93	58.2%	1.07 [0.56, 2.04]
Omer 2022 (3)	−1.0986	1.1404	61	61	4.9%	0.33 [0.04, 3.12]
Stewart 2019 (4)	0.5596	0.5053	290	305	24.9%	1.75 [0.65, 4.71]
**Subtotal (95% CI)**			**473**	**494**	**100.0%**	**1.09 [0.67, 1.79]**
Heterogeneity: Tau² = 0.00; Chi² = 2.22, df = 3 (P = 0.53); I² = 0%						
Test for overall effect: Z = 0.36 (P = 0.72)						
**1.4.2 Fortified Blended Food**						
Borg 2020 (5)	−0.2877	0.6031	59	77	7.0%	0.75 [0.23, 2.45]
Christian 2015 (6)	0.0677	0.1235	513	843	33.7%	1.07 [0.84, 1.36]
Langendorf 2014 (7)	−0.6539	0.202	338	336	26.3%	0.52 [0.35, 0.77]
Lutter 2008 (8)	0.967	1.6195	170	149	1.1%	2.63 [0.11, 62.88]
Mangani 2015 (9)	0.4253	0.9043	180	184	3.4%	1.53 [0.26, 9.00]
Samuel−2018 (10)	−0.3011	0.18	1025	1052	28.4%	0.74 [0.52, 1.05]
**Subtotal (95% CI)**			**2285**	**2641**	**100.0%**	**0.80 [0.56, 1.12]**
Heterogeneity: Tau² = 0.08; Chi² = 10.94, df = 5 (P = 0.05); I² = 54%						
Test for overall effect: Z = 1.31 (P = 0.19)						
**1.4.3 Locally Produced Ready to Use Supplementary Food**						
Agapova 2017 (11)	−0.1305	0.4412	69	27	6.0%	0.88 [0.37, 2.08]
Agapova 2017 (12)	−0.2213	0.4535	68	28	5.7%	0.80 [0.33, 1.95]
Agapova 2017 (13)	0.4572	1.218	69	27	0.8%	1.58 [0.15, 17.19]
Agapova 2017 (14)	1.165	1.1106	68	27	0.9%	3.21 [0.36, 28.27]
Borg 2020 (15)	−0.3285	0.5605	76	77	3.7%	0.72 [0.24, 2.16]
Christian 2015 (16)	−0.0101	0.1696	513	422	40.5%	0.99 [0.71, 1.38]
Christian 2015 (17)	−0.0408	0.1685	503	421	41.0%	0.96 [0.69, 1.34]
Mangani 2015 (18)	0.384	1.1477	188	92	0.9%	1.47 [0.15, 13.92]
Mangani 2015 (19)	−1.8326	1.4146	191	92	0.6%	0.16 [0.01, 2.56]
**Subtotal (95% CI)**			**1745**	**1213**	**100.0%**	**0.96 [0.77, 1.18]**
Heterogeneity: Tau² = 0.00; Chi² = 3.58, df = 8 (P = 0.89); I² = 0%						
Test for overall effect: Z = 0.43 (P = 0.67)						
**1.4.4 Commercially Produced Ready to Use Supplementary Food**						
Christian 2015 (20)	−0.1625	0.161	896	843	16.3%	0.85 [0.62, 1.17]
Huybregts 2012 (21)	0.0198	0.0812	529	389	23.1%	1.02 [0.87, 1.20]
Isanaka 2010 (22)	−0.4005	0.2383	284	315	11.1%	0.67 [0.42, 1.07]
Khan 2020 (23)	−0.2485	0.2694	105	120	9.5%	0.78 [0.46, 1.32]
Langendorf 2014 (24)	−0.5625	0.3414	277	84	6.8%	0.57 [0.29, 1.11]
Langendorf 2014 (25)	−0.5621	0.2069	195	84	13.0%	0.57 [0.38, 0.86]
Langendorf 2014 (26)	−0.5276	0.1983	278	84	13.5%	0.59 [0.40, 0.87]
Langendorf 2014 (27)	−0.2742	0.3463	195	84	6.7%	0.76 [0.39, 1.50]
**Subtotal (95% CI)**			**2759**	**2003**	**100.0%**	**0.75 [0.61, 0.92]**
Heterogeneity: Tau² = 0.04; Chi² = 14.73, df = 7 (P = 0.04); I² = 52%						
Test for overall effect: Z = 2.82 (P = 0.005)						
**1.4.5 SQ LNS**						
Addo 2020 (28)	0.2546	0.1615	654	653	8.3%	1.29 [0.94, 1.77]
Adu−Afarwuah 2016 (29)	0.0583	0.2989	347	350	2.4%	1.06 [0.59, 1.90]
Ashorn 2015 (30)	−0.1059	0.5086	214	220	0.8%	0.90 [0.33, 2.44]
Becquey 2019 (31)	−0.0052	0.1237	885	896	14.1%	0.99 [0.78, 1.27]
Dewey 2017 (32)	−0.0943	0.1266	785	408	13.5%	0.91 [0.71, 1.17]
Dewey 2017 (33)	−0.1508	0.1198	884	408	15.1%	0.86 [0.68, 1.09]
Hess 2015 (34)	−0.4943	0.1552	1623	523	9.0%	0.61 [0.45, 0.83]
Humphrey 2019 (35)	−0.0101	0.4375	355	350	1.1%	0.99 [0.42, 2.33]
Hurley 2021 (36)	−0.7765	1.2461	165	152	0.1%	0.46 [0.04, 5.29]
Huybregts 2019 (37)	−0.0943	0.0987	1154	1159	22.2%	0.91 [0.75, 1.10]
Luby 2018 (38)	−0.1966	0.1674	516	1004	7.7%	0.82 [0.59, 1.14]
Maleta 2015 (39)	−0.0408	0.5296	265	51	0.8%	0.96 [0.34, 2.71]
Maleta 2015 (40)	−0.6931	0.4937	266	51	0.9%	0.50 [0.19, 1.32]
Maleta 2015 (41)	−0.5447	0.4946	266	51	0.9%	0.58 [0.22, 1.53]
Maleta 2015 (42)	0.2151	0.5172	252	52	0.8%	1.24 [0.45, 3.42]
Maleta 2015 (43)	−0.0408	0.4728	256	51	1.0%	0.96 [0.38, 2.43]
Null 2018 (44)	−0.1863	0.4706	496	1097	1.0%	0.83 [0.33, 2.09]
Prendergast 2019 (45)	−0.0202	0.9928	59	58	0.2%	0.98 [0.14, 6.86]
**Subtotal (95% CI)**			**9442**	**7534**	**100.0%**	**0.90 [0.82, 0.98]**
Heterogeneity: Tau² = 0.00; Chi² = 15.66, df = 17 (P = 0.55); I² = 0%						
Test for overall effect: Z = 2.32 (P = 0.02)						

Risk Ratio
IV, Random, 95% CI

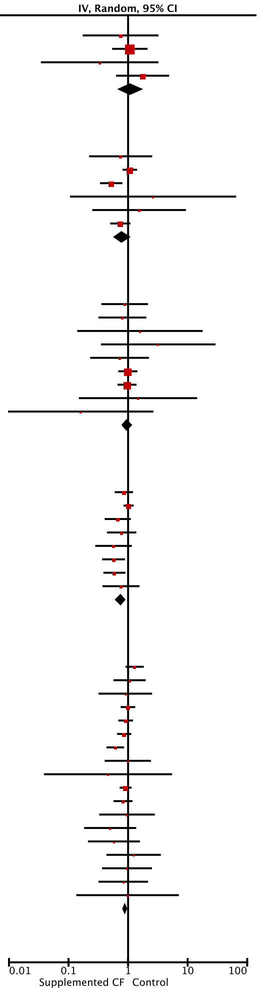

Test for subgroup differences: Chi² = 4.33, df = 4 (P = 0.36), I² = 7.6%

Footnotes

(1) cRCT, Africa, children aged 5 months; supplementation for18 months; prevalence of wasting
(2) RCT, South-East Asian Region, 4 and 12 mo of age, supplementation for 6 months intervention; prevalence of wasting
(3) cRCT, Ethioipia, Africa, eggs
(4) RCT, Africa, 6–9.9 months age; supplementation for 6 months; prevalence of wasting
(5) CSB++ cm; RCT, Western Pacific Region, 6months of age, supplementation for 6–12 months; prevalence of wasting
(6) WSB++ ; RCT, South-East Asian Region, age–6–18 months; supplementation for 12 months; prevalence of wasting
(7) SuperCereal (SC)–850 kcal; cRCT, Africa, 6–23 months age, supplementation for < 6 months, incidence of wasting
(8) Non–RCT, Region of the Americas, 6–11 months age; supplementation for 11 months; prevalence of wasting
(9) RCT; CSB–T, Africa, age 6–18 months, suplementation for 12 month; prevalence of severe wasting
(10) Non RCT, Africa, Rural, 6–23 months age; prevalence of wasting
(11) RCT, cowpea, Africa, age 12–23 months, suplementation for 24 month, incidence of MAM
(12) RCT, common beans, Africa, age 12–23 months, suplementation for 24 month; incidence of MAM
(13) RCT, cowpea, Africa, age 12–23 months, suplementation for 24 month, SAM
(14) RCT, common beans, Africa, age 12–23 months, suplementation for 24 month; SAM
(15) cRCT, RUSF (fish based); Western Pacific Region, 6months age, supplementation for 6–12 months; prevalence of wasting
(16) cRCT; chickpeas; South East Asia; aged 6–18 months; supplementation for 12 months; prevalence of wasting
(17) cRCT; rice/lentils; South East Asia; aged 6–18 months; supplementation for 12 months; prevalence of wasting
(18) Soy LNS; RCT, Africa, age 6–18 months; supplementation for 12 months; prevalence of severe wasting
(19) Milk LNS; RCT, Africa, age 6–18 months; supplementation for 12 months; prevalence of severe wasting
(20) cRCT; Plumpy doz, South–East Asian Region, age–6–18 months; supplementation for 12 months; prevalence of wasting
(21) cRCT; Plumpydoz Nutriset 46 g ~247Kcal/ d; Africa; age 6–36 months, supplementation for 4 months; incidence of wasting
(22) cRCT; Africa; RUTF (500 kcal/day); children 6–60 months; incidence of wasting
(23) cRCT, EMRO, age 6–24 months,MQLNS supplementation for 12 months; prevalence of wasting
(24) cRCT; SAM; MQ–LNS (250 kcal/day–5 months), RCT; Africa, 6–23 months age, supplementation for < 6 months, incidence of wasting
(25) cRCT; MAM; HQ–LNS (250 kcal/day–5 months), Africa, 6–23 months age, supplementation for < 6 months, incidence of wasting
(26) cRCT; MAM; MQ–LNS (250 kcal/day–5 months), Africa, 6–23 months age, supplementation for < 6 months, incidence of wasting
(27) cRCT; SAM; HQ–LNS (500 kcal/day–5 months), RCT; Africa, 6–23 months age, supplementation for < 6 months,incidence of wasting
(28) non–RCT; Africa; 6–18 months age, supplementation for 3 months; prevalence of wasting
(29) SQ–LNS; RCT; African region; mothers with >20 wk of gestation; maternal (during pregnancy) and child supplementation for 6 months; prevalence of wasting
(30) SQLNS; RCT; Malawi; children aged 6–18 months; maternal (during pregnancy) and child supplementaion for 6 months; prevalence of wasting
(31) SQLNS 20gm; cRCT: Africa; children aged 0–17 months; prevalence of wasting
(32) SQ LNS; cRCT, South–East Asia; 6–24 months age, supplementation for 18 months; prevalence of wasting
(33) SQ LNS; cRCT, South–East Asia; 6–24 months age, maternal (during pregnancy) and child supplementation for 18 months; prevalence of wasting
(34) zinc; cRCT, Africa, aged 9–18 months of age, supplementation for 9 months; prevalence of wasting
(35) cRCT, SQLNS 20 gm–HIV negative, Africa, Aged 6–18 month, supplementation for 12 months; prevalence of wasting
(36) Non–RCT, Africa; 6–24 months of age, Africa; supplementation for 12 months; prevalence of wasting
(37) cRCT; Africa; age 6–23 months, supplementation for 12 months; prevalence of wasting
(38) 20 g SQLNS, cRCT; Bangladesh; 6–24 months age; supplementation for 12 months; prevalence of wasting
(39) 20g no milk; RCT, Africa; aged 6 months, supplementation for 12 months; incidence of wasting
(40) 40g no milk; RCT, Africa; aged 6 months, supplementation for 12 months; incidence of wasting
(41) 40g milk; RCT, Africa; aged 6 months, supplementation for 12 months; incidence of wasting
(42) 10g milk; RCT, Africa; aged 6 months, supplementation for 12 months; incidence of wasting
(43) 20g milk; RCT, Africa; aged 6 months, supplementation for 12 months; incidence of wasting
(44) SQLNS–20 gm, cRCT, Africa; 6–24 month aged, duration NR; prevalence of wasting
(45) SQLNS–20 gm–HIV positive, cRCT, Africa, Aged 6–18 month, supplementation for 12 months; prevalence of wasting

Severe wasting <-3 SD

Study or Subgroup	log[Risk Ratio]	SE	Child complementary feeding Total	Control Total	Weight	Risk Ratio IV, Random, 95% CI
**1.5.1 Fortified Blended Food**						
Langendorf 2014 (1)	−0.6419	0.405	338	336	78.7%	0.53 [0.24, 1.16]
Mangani 2015 (2)	0.4253	0.9043	180	184	21.3%	1.53 [0.26, 9.00]
**Subtotal (95% CI)**			**518**	**520**	**100.0%**	**0.66 [0.28, 1.56]**
Heterogeneity: Tau² = 0.08; Chi² = 1.16, df = 1 (P = 0.28); I² = 14%						
Test for overall effect: Z = 0.95 (P = 0.34)						
**1.5.2 Locally Produced Ready to Use Supplementary Food**						
Agapova 2017 (3)	1.165	1.1106	138	54	29.5%	3.21 [0.36, 28.27]
Agapova 2017 (4)	0.4572	1.218	136	55	24.6%	1.58 [0.15, 17.19]
Mangani 2015 (5)	0.384	1.1477	188	92	27.7%	1.47 [0.15, 13.92]
Mangani 2015 (6)	−1.8326	1.4146	191	92	18.2%	0.16 [0.01, 2.56]
**Subtotal (95% CI)**			**653**	**293**	**100.0%**	**1.26 [0.39, 4.11]**
Heterogeneity: Tau² = 0.00; Chi² = 2.89, df = 3 (P = 0.41); I² = 0%						
Test for overall effect: Z = 0.38 (P = 0.70)						
**1.5.3 Commercially Produced Ready to Use Supplementary Food**						
Langendorf 2014 (7)	−0.5625	0.3414	277	84	50.7%	0.57 [0.29, 1.11]
Langendorf 2014 (8)	−0.2742	0.3463	195	84	49.3%	0.76 [0.39, 1.50]
**Subtotal (95% CI)**			**472**	**168**	**100.0%**	**0.66 [0.41, 1.06]**
Heterogeneity: Tau² = 0.00; Chi² = 0.35, df = 1 (P = 0.55); I² = 0%						
Test for overall effect: Z = 1.73 (P = 0.08)						
**1.5.4 SQ LNS**						
Ashorn 2015 (9)	−0.7022	1.221	222	220	7.6%	0.50 [0.05, 5.42]
Becquey 2019 (10)	0.0664	0.3255	885	896	31.0%	1.07 [0.56, 2.02]
Huybregts 2019 (11)	−0.5108	0.3975	101	101	27.8%	0.60 [0.28, 1.31]
Maleta 2015 (12)	2.1933	1.4881	254	52	5.4%	8.96 [0.49, 165.66]
Maleta 2015 (13)	−1.1636	1.6306	269	52	4.6%	0.31 [0.01, 7.63]
Maleta 2015 (14)	1.3063	1.1146	273	53	8.8%	3.69 [0.42, 32.81]
Maleta 2015 (15)	1.9147	1.0654	260	53	9.4%	6.78 [0.84, 54.75]
Maleta 2015 (16)	2.1398	1.4881	268	52	5.4%	8.50 [0.46, 157.03]
**Subtotal (95% CI)**			**2532**	**1479**	**100.0%**	**1.35 [0.65, 2.80]**
Heterogeneity: Tau² = 0.34; Chi² = 11.30, df = 7 (P = 0.13); I² = 38%						
Test for overall effect: Z = 0.81 (P = 0.42)						

Risk Ratio
IV, Random, 95% CI

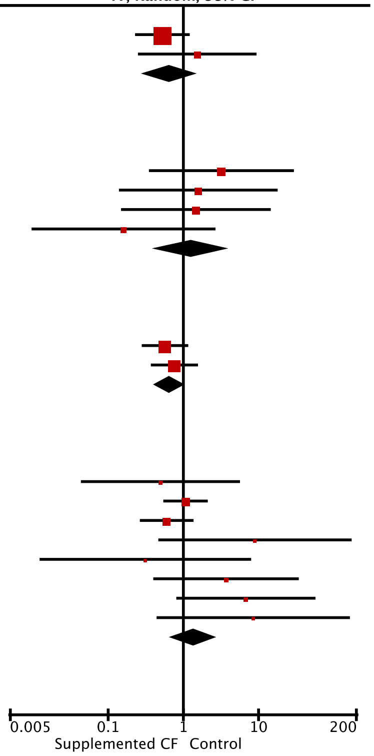

Test for subgroup differences: Chi² = 3.38, df = 3 (P = 0.34), I² = 11.2%

Footnotes
(1) SuperCereal (SC)–850 kcal; cRCT, Africa, 6–23 months age, supplementation for < 6 months, incidence of wasting
(2) RCT; CSB–T, Africa, age 6–18 months, suplementation for 12 month; prevalence of severe wasting
(3) RCT, common beans, Africa, age 12–23 months, suplementation for 24 month; SAM
(4) RCT, cowpea, Africa, age 12–23 months, suplementation for 24 month, SAM
(5) Soy LNS; RCT, Africa, age 6–18 months; supplementation for 12 months; prevalence of severe wasting
(6) Milk LNS; RCT, Africa, age 6–18 months; supplementation for 12 months; prevalence of severe wasting
(7) SAM; MQ–LNS (250 kcal/day–5 months), RCT; Africa, 6–23 months age, supplementation for < 6 months,
(8) SAM; HQ–LNS (500 kcal/day–5 months), RCT; Africa, 6–23 months age, supplementation for < 6 months,
(9) SQLNS; RCT; Malawi; children aged 6–18 months; maternal (during pregnancy) and child supplementaion for 6 months
(10) SQLNS 20gm; cRCT: Africa; children aged 0–17 months; prevalence of wasting
(11) RCT; Africa; age 6–23 months, supplementation for 12 months; prevalence of severe wasting
(12) 10g milk; RCT, Africa; aged 6 months, supplementation for 12 months; prevalence of severe wasting
(13) 40g no milk; RCT, Africa; aged 6 months, supplementation for 12 months; prevalence of severe wasting
(14) 40g milk; RCT, Africa; aged 6 months, supplementation for 12 months; prevalence of severe wasting
(15) 20g milk; RCT, Africa; aged 6 months, supplementation for 12 months; prevalence of severe wasting
(16) 20g no milk; RCT, Africa; aged 6 months, supplementation for 12 months; prevalence of severe wasting

WAZ

Study or Subgroup	Mean Difference	SE	Child complementary feeding Total	Control Total	Weight	Mean Difference IV, Random, 95% CI
**1.9.1 Alternative food**						
Bauserman 2015 (1)	−0.2	0.2857	35	36	18.8%	−0.20 [−0.76, 0.36]
Iannoti 2017 (2)	0.21	0.1577	75	73	26.2%	0.21 [−0.10, 0.52]
Omer 2022 (3)	0.89	0.1738	61	61	25.3%	0.89 [0.55, 1.23]
Stewart 2019 (4)	0.13	0.0868	290	305	29.7%	0.13 [−0.04, 0.30]
**Subtotal (95% CI)**			**461**	**475**	**100.0%**	**0.28 [−0.10, 0.66]**
Heterogeneity: $Tau^2 = 0.12$; $Chi^2 = 18.00$, df = 3 (P = 0.0004); $I^2 = 83\%$						
Test for overall effect: Z = 1.44 (P = 0.15)						
**1.9.2 Fortified Blended Food**						
Akalu 2010 (5)	0.2	0.126	106	105	6.9%	0.20 [−0.05, 0.45]
Akalu 2010 (6)	−0.26	0.1658	61	65	5.9%	−0.26 [−0.58, 0.06]
Borg 2020 (7)	0.09	0.1071	59	77	7.4%	0.09 [−0.12, 0.30]
Chipili 2022 (8)	1.05	0.0987	100	86	7.6%	1.05 [0.86, 1.24]
Christian 2015 (9)	0.02	0.0102	513	843	8.9%	0.02 [0.00, 0.04]
Ghosh 2020 (10)	0.06	0.0714	157	158	8.2%	0.06 [−0.08, 0.20]
Lutter 2008 (11)	0.26	0.1095	170	149	7.3%	0.26 [0.05, 0.47]
Mangani 2015 (12)	−0.07	0.0816	183	185	8.0%	−0.07 [−0.23, 0.09]
Oelofse 2003 (13)	−0.03	0.4941	16	14	1.6%	−0.03 [−1.00, 0.94]
Owino 2007 (14)	0.1	0.1986	44	35	5.2%	0.10 [−0.29, 0.49]
Owino 2007 (15)	0.15	0.2376	37	34	4.4%	0.15 [−0.32, 0.62]
Phu 2012 (16)	0.12	0.1326	135	64	6.8%	0.12 [−0.14, 0.38]
Phu 2012 (17)	−0.02	0.1378	114	64	6.6%	−0.02 [−0.29, 0.25]
Samuel−2018 (18)	0.3	0.0472	1172	1137	8.6%	0.30 [0.21, 0.39]
Sazawal 2014 (19)	0.2	0.14	59	53	6.6%	0.20 [−0.07, 0.47]
**Subtotal (95% CI)**			**2926**	**3069**	**100.0%**	**0.16 [0.03, 0.30]**
Heterogeneity: $Tau^2 = 0.05$; $Chi^2 = 151.90$, df = 14 (P < 0.00001); $I^2 = 91\%$						
Test for overall effect: Z = 2.33 (P = 0.02)						
**1.9.3 Locally Produced Ready to Use Supplementary Food**						
Borg 2020 (20)	0.13	0.0918	76	77	1.3%	0.13 [−0.05, 0.31]
Christian 2015 (21)	0.04	0.0153	513	421	48.1%	0.04 [0.01, 0.07]
Christian 2015 (22)	0.02	0.0153	503	422	48.1%	0.02 [−0.01, 0.05]
Mangani 2015 (23)	0.05	0.102	188	93	1.1%	0.05 [−0.15, 0.25]
Mangani 2015 (24)	0.09	0.0918	191	92	1.3%	0.09 [−0.09, 0.27]
**Subtotal (95% CI)**			**1471**	**1105**	**100.0%**	**0.03 [0.01, 0.05]**
Heterogeneity: $Tau^2 = 0.00$; $Chi^2 = 2.46$, df = 4 (P = 0.65); $I^2 = 0\%$						
Test for overall effect: Z = 3.05 (P = 0.002)						
**1.9.4 Commercially Produced Ready to Use Supplementary Food**						
Christian 2015 (25)	0.03	0.0153	896	843	98.1%	0.03 [0.00, 0.06]
Santos 2005 (26)	0.07	0.1101	91	81	1.9%	0.07 [−0.15, 0.29]
**Subtotal (95% CI)**			**987**	**924**	**100.0%**	**0.03 [0.00, 0.06]**
Heterogeneity: $Tau^2 = 0.00$; $Chi^2 = 0.13$, df = 1 (P = 0.72); $I^2 = 0\%$						
Test for overall effect: Z = 2.03 (P = 0.04)						
**1.9.5 SQ LNS**						
Addo 2020 (27)	−0.59	0.0561	654	653	5.6%	−0.59 [−0.70, −0.48]
Adu−afarwah 2007 (28)	0.34	0.1656	97	81	3.7%	0.34 [0.02, 0.66]
Adu−Afarwuah 2016 (29)	−0.14	0.0867	334	339	5.1%	−0.14 [−0.31, 0.03]
Ashorn 2015 (30)	−0.38	0.1122	195	200	4.7%	−0.38 [−0.60, −0.16]
Dewey 2017 (31)	0.1	0.0459	785	408	5.7%	0.10 [0.01, 0.19]
Dewey 2017 (32)	0.11	0.0453	884	408	5.7%	0.11 [0.02, 0.20]
Galasso 2019 (33)	−0.035	0.0796	139	139	5.2%	−0.04 [−0.19, 0.12]
Hess 2015 (34)	0.28	0.0561	1623	523	5.6%	0.28 [0.17, 0.39]
Humphrey 2019 (35)	0.06	0.0765	355	350	5.3%	0.06 [−0.09, 0.21]
Iannotti 2014 (36)	0.07	0.1476	126	72	4.0%	0.07 [−0.22, 0.36]
Iannotti 2014 (37)	0.05	0.1313	150	72	4.3%	0.05 [−0.21, 0.31]
Luby 2018 (38)	0.25	0.0559	521	1019	5.6%	0.25 [0.14, 0.36]
Maleta 2015 (39)	−0.04	0.2755	238	48	2.2%	−0.04 [−0.58, 0.50]
Maleta 2015 (40)	0	0.2755	242	48	2.2%	0.00 [−0.54, 0.54]
Maleta 2015 (41)	0.01	0.2755	220	49	2.2%	0.01 [−0.53, 0.55]
Maleta 2015 (42)	−0.12	0.2755	235	48	2.2%	−0.12 [−0.66, 0.42]
Maleta 2015 (43)	−0.04	0.2755	245	48	2.2%	−0.04 [−0.58, 0.50]
Muslihah 2016 (44)	0.25	0.0208	58	25	6.0%	0.25 [0.21, 0.29]
Muslihah 2016 (45)	0.27	0.0205	59	26	6.0%	0.27 [0.23, 0.31]
Null 2018 (46)	0.07	0.051	499	1106	5.7%	0.07 [−0.03, 0.17]
Prendergast 2019 (47)	0.03	0.1939	59	58	3.2%	0.03 [−0.35, 0.41]
Smuts 2019 (48)	0.06	0.148	162	100	4.0%	0.06 [−0.23, 0.35]
Smuts 2019 (49)	−0.14	0.1582	151	101	3.8%	−0.14 [−0.45, 0.17]
**Subtotal (95% CI)**			**8031**	**5921**	**100.0%**	**0.03 [−0.07, 0.13]**
Heterogeneity: $Tau^2 = 0.04$; $Chi^2 = 291.54$, df = 22 (P < 0.00001); $I^2 = 92\%$						
Test for overall effect: Z = 0.62 (P = 0.53)						

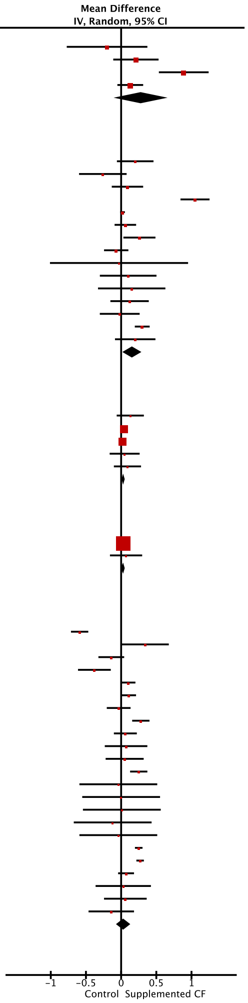

Test for subgroup differences: $Chi^2 = 5.05$, df = 4 (P = 0.28), $I^2 = 20.8\%$

Footnotes
(1) RCT, Africa, children aged 5 months; supplementation for18 months; endline
(2) Eggs; RCT; Africa; children aged 6−9 months; supplementation for 6 months; endline
(3) cRCT, Ethioipia, Africa, eggs
(4) RCT, Africa, 6−9.9 months age; supplementation for 6 months; endline
(5) RCT: Africa; children aged 5−17 months; supplementation for 1 year; Sibu−Siru−13 months;endline WAZ
(6) cRCT: Africa; children aged 5−17 months; supplementation for 1 year; Wama Bomaya−13 months; endline WAZ
(7) CSB++; cRCT, Western Pacific Region, 6months age, supplementation for 6−12 months; change in WAZ
(8) Fish powder; RCT; Africa; children aged 6−7 months; supplementation for 6 months, endline
(9) WSB++ ; cRCT, South−East Asian Region, age−6−18 months; supplementation for 12 months; change in WAZ
(10) cRCT, Africa, 6−18 months age, supplementation for intervention 6−12 months; endline
(11) Non−RCT, Region of the Americas, 6−11 months age; supplementation for 11 months; endline
(12) CSB− RCT, Africa, age 6−18 months, suplementation for 12 month; change in WAZ
(13) RCT: South Africa; children aged 6−12 months; supplementation for 6 months; endline
(14) CBMA; RCT: Africa; Children aged 6 months; supplementation for 3 months, endline
(15) CBM; RCT: Africa; Children aged 6 months; supplementation for 3 months, endline
(16) fortified flour; RCT, Western Pacific Region, 5−29 months age; supplementation for 11 months; endline
(17) Food complement ; RCT, Western Pacific Region, 5−29 months age; supplementation for 11 months; endline
(18) Non RCT, Africa, 6−23 months age, duration NR; change in WAZ
(19) RCT: India; children aged 6−24 months; supplementation for 6 months; change in WAZ
(20) RUSF (fish based); cRCT, Western Pacific Region, 6months age, supplementation for 6−12 months; change in WAZ
(21) cRCT; chickpeas; South East Asia; aged 6−18 months; supplementation for 12 months; change in WAZ
(22) cRCT; rice/lentils; South East Asia; aged 6−18 months; supplementation for 12 months; change in WAZ
(23) Soy LNS; RCT, Africa, age 6−18 months; supplementation for 12 months; change in WAZ
(24) Milk LNS; RCT, Africa, age 6−18 months; supplementation for 12 months; change in WAZ
(25) Plumpy doz, cRCT, South−East Asian Region, age−6−18 months; supplementation for 12 months; change in WAZ
(26) milk; non−RCT: Brazil; children aged 6−18 months; change in WAZ
(27) non−RCT; Africa; 6−18 months age, supplementation for 3 months
(28) SQ−LNS Nutributter; qRCT; African region; mothers with >20 wk of gestation; supplementation for 6 months
(29) chang in WAZ was measured at 18 months of intervention−maternal and child; SSQ−LNS; RCT; African region; mothers with >20 wk of gestation; supplementation for 6 months
(30) SQLNS; RCT; Malawi; children aged 6−18 months; supplementaion for 6 months, change in WAZ
(31) SQ LNS; cRCT, South−East Asia; 6−24 months age, supplementation for 18 months; endline
(32) SQ LNS; cRCT, South−East Asia; mothers and thier children 6−24 months age, supplementation for 18 months, endline
(33) SQLNS ; RCT; Madasgar; Children <12 years; supplementation for 12 months; change in WAZ
(34) zinc, cRCT, Africa, aged 9−18 months of age, supplementation for 9 months; endline
(35) SQLNS 20 gm−HIV negative, cRCT, Africa, Aged 6−18 month, supplementation for 12 months, endline
(36) SQ−LNS; RCT African region; children aged 6−11 months; supplementation for 3 months, change in WAZ at 7th visit
(37) SQ−LNS; RCT African region; children aged 6−11 months; supplementation for 6 months, change in WAZ at 7th visit
(38) 20 g SQLNS, cRCT; Bangladesh; 6−24 months age; supplementation for 12 months, endline
(39) 40g no milk; RCT, Africa; aged 6 months, supplementation for 12 months, change in WAZ
(40) 40g milk; RCT, Africa; aged 6 months, supplementation for 12 months, change in WAZ
(41) 10g milk; RCT, Africa; aged 6 months, supplementation for 12 months, change in WAZ
(42) 20g milk; RCT, Africa; aged 6 months, supplementation for 12 months, change in WAZ
(43) 20g no milk; RCT, Africa; aged 6 months, supplementation for 12 months, change in WAZ
(44) SQ−LNS− non−RCT: Indonesia; Children aged 6−12 months; supplementation for 6 months; at endline
(45) Biscuits; non−RCT: Indonesia; Children aged 6−12 months; supplementation for 6 months; at endline
(46) SQLNS−20 gm, cRCT, Africa; 6−24 month aged, endline
(47) SQLNS−20 gm−HIV positive, cRCT, Africa, Aged 6−18 month, supplementation for 12 months, endline
(48) SQ LNS plus; RCT, Africa; aged 6−12 months, supplementation for 6 months, endline
(49) SQ LNS; RCT, Africa; aged 6−12 months, supplementation for 6 months, endline

Underweight

Study or Subgroup	log[Risk Ratio]	SE	Child complementary feeding Total	Control Total	Weight	Risk Ratio IV, Random, 95% CI
**1.6.1 Alternative food**						
Iannoti 2017 (1)	−0.2502	0.6504	75	73	22.4%	0.78 [0.22, 2.79]
Omer 2022 (2)	−1.2993	0.6257	61	61	23.5%	0.27 [0.08, 0.93]
Stewart 2019 (3)	−0.0101	0.2226	290	305	54.1%	0.99 [0.64, 1.53]
**Subtotal (95% CI)**			**426**	**439**	**100.0%**	**0.69 [0.33, 1.45]**
Heterogeneity: Tau² = 0.21; Chi² = 3.79, df = 2 (P = 0.15); I² = 47%						
Test for overall effect: Z = 0.97 (P = 0.33)						
**1.6.2 Fortified Blended Food**						
Borg 2020 (4)	−0.0943	0.4592	59	77	9.0%	0.91 [0.37, 2.24]
Christian 2015 (5)	0.0296	0.0688	513	843	35.4%	1.03 [0.90, 1.18]
Lutter 2008 (6)	−1.0498	0.3994	170	149	11.0%	0.35 [0.16, 0.77]
Mangani 2015 (7)	0.1117	0.4054	200	205	10.8%	1.12 [0.51, 2.48]
Samuel-2018 (8)	−0.3288	0.0879	1025	1052	33.9%	0.72 [0.61, 0.86]
**Subtotal (95% CI)**			**1967**	**2326**	**100.0%**	**0.81 [0.59, 1.10]**
Heterogeneity: Tau² = 0.07; Chi² = 16.12, df = 4 (P = 0.003); I² = 75%						
Test for overall effect: Z = 1.35 (P = 0.18)						
**1.6.3 Locally Produced Ready to Use Supplementary Food**						
Borg 2020 (9)	−0.2107	0.4429	76	77	2.2%	0.81 [0.34, 1.93]
Christian 2015 (10)	−0.0101	0.0961	503	422	47.2%	0.99 [0.82, 1.20]
Christian 2015 (11)	−0.1278	0.0953	513	421	48.0%	0.88 [0.73, 1.06]
Mangani 2015 (12)	−1.2234	0.7202	208	102	0.8%	0.29 [0.07, 1.21]
Mangani 2015 (13)	−0.1823	0.5021	206	103	1.7%	0.83 [0.31, 2.23]
**Subtotal (95% CI)**			**1506**	**1125**	**100.0%**	**0.92 [0.81, 1.05]**
Heterogeneity: Tau² = 0.00; Chi² = 3.43, df = 4 (P = 0.49); I² = 0%						
Test for overall effect: Z = 1.28 (P = 0.20)						
**1.6.4 Commercially Produced Ready to Use Supplementary Food**						
Christian 2015 (14)	−0.1278	0.0884	843	843	73.4%	0.88 [0.74, 1.05]
Khan 2020 (15)	0	0.1468	105	120	26.6%	1.00 [0.75, 1.33]
**Subtotal (95% CI)**			**948**	**963**	**100.0%**	**0.91 [0.78, 1.06]**
Heterogeneity: Tau² = 0.00; Chi² = 0.56, df = 1 (P = 0.46); I² = 0%						
Test for overall effect: Z = 1.24 (P = 0.22)						
**1.6.5 SQ LNS**						
Addo 2020 (16)	0.7975	0.0958	654	653	9.2%	2.22 [1.84, 2.68]
Adu-Afarwuah 2016 (17)	−0.0408	0.2149	347	350	7.1%	0.96 [0.63, 1.46]
Ashorn 2015 (18)	−0.4308	0.2606	363	362	6.3%	0.65 [0.39, 1.08]
Dewey 2017 (19)	−0.1054	0.0665	785	408	9.6%	0.90 [0.79, 1.03]
Dewey 2017 (20)	−0.0619	0.0635	884	408	9.6%	0.94 [0.83, 1.06]
Hess 2015 (21)	−0.478	0.0897	1623	523	9.3%	0.62 [0.52, 0.74]
Humphrey 2019 (22)	−0.1054	0.2421	355	350	6.6%	0.90 [0.56, 1.45]
Luby 2018 (23)	−0.1676	0.0868	521	1019	9.3%	0.85 [0.71, 1.00]
Maleta 2015 (24)	−0.4954	0.4073	211	45	4.1%	0.61 [0.27, 1.35]
Maleta 2015 (25)	0.1295	0.4123	229	46	4.1%	1.14 [0.51, 2.55]
Maleta 2015 (26)	0.1496	0.3758	232	46	4.5%	1.16 [0.56, 2.43]
Maleta 2015 (27)	−0.0901	0.418	238	45	4.0%	0.91 [0.40, 2.07]
Maleta 2015 (28)	−0.1531	0.421	236	45	4.0%	0.86 [0.38, 1.96]
Null 2018 (29)	−0.1278	0.1787	499	1106	7.8%	0.88 [0.62, 1.25]
Prendergast 2019 (30)	0.077	0.3929	59	58	4.3%	1.08 [0.50, 2.33]
**Subtotal (95% CI)**			**7236**	**5464**	**100.0%**	**0.94 [0.76, 1.16]**
Heterogeneity: Tau² = 0.12; Chi² = 107.81, df = 14 (P < 0.00001); I² = 87%						
Test for overall effect: Z = 0.60 (P = 0.55)						

Risk Ratio
IV, Random, 95% CI

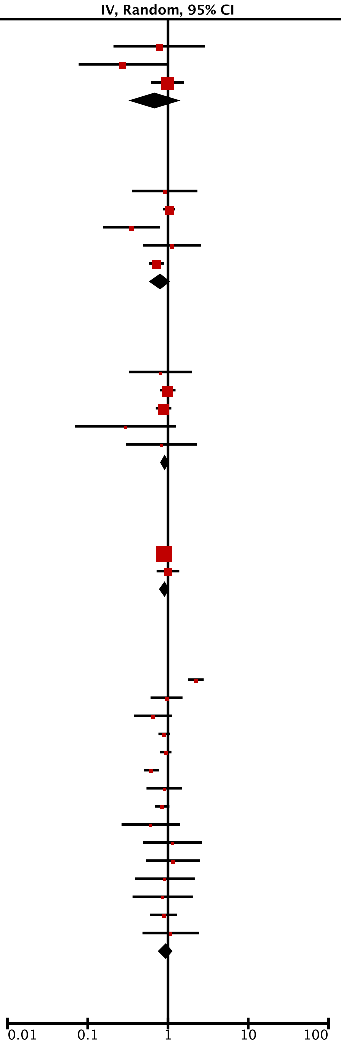

Test for subgroup differences: Chi² = 1.19, df = 4 (P = 0.88), I² = 0%

Footnotes
(1) Eggs; RCT; Africa; children aged 6-9 months; supplementation for 6 months; endline
(2) cRCT, Ethioipia, Africa, eggs
(3) RCT, Africa, 6-9.9 months age; supplementation for 6 months
(4) CSB++ cm; RCT, Western Pacific Region, 6months of age, supplementation for 6-12 months,
(5) WSB++ ; RCT, South-East Asian Region, age-6-18 months; supplementation for 12 months
(6) Non-RCT, Region of the Americas, 6-11 months age; supplementation for 11 months
(7) CSB- RCT, Africa, age 6-18 months, suplementation for 12 month
(8) Non RCT, Africa, Rural, 6-23 months age
(9) RUSF (fish based); RCT, Western Pacific Region, 6months age, supplementation for 6-12 months
(10) rice/lentils; RCT, South-East Asian Region, age-6-18 months; supplementation for 12 months
(11) Chickpeas; ; RCT, South-East Asian Region, age-6-18 months; supplementation for 12 months
(12) Milk; RCT, Africa, age 6-18 months; supplementation for 12 months
(13) Soy LNS; RCT, Africa, age 6-18 months; supplementation for 12 months
(14) Plumpy doz- RCT, South-East Asian Region, age-6-18 months; supplementation for 12 months
(15) MQ-LNS, RCT; South East Asia; aged 6-24 months, supplementation for 12 months
(16) non-RCT; Africa; 6-18 months age, maternal (during pregnancy) and child supplementation for 3 months
(17) SQ-LNS; RCT; African region; mothers with >20 wk of gestation; maternal (during pregnancy) and child supplementation for 6 months
(18) SQLNS; RCT; Malawi; children aged 6-18 months; maternal (during pregnancy) and child supplementaion for 6 months
(19) SQ LNS; cRCT, South-East Asia; 6-24 months age, supplementation for 18 months
(20) SQ LNS; cRCT, South-East Asia; mother and children 6-24 months age, maternal (during pregnancy) and child supplementation for 18 months
(21) zinc; cRCT, Africa, aged 9-18 months of age, supplementation for 9 months
(22) SQLNS 20 gm-HIV negative, cRCT, Africa, Aged 6-18 month, supplementation for 12 months
(23) 20 g SQLNS, non-RCT; Bangladesh; 6-24 months age; supplementation for 12 months
(24) 10g milk; RCT, Africa; aged 6 months, supplementation for 12 months
(25) 20g milk; RCT, Africa; aged 6 months, supplementation for 12 months
(26) 20g no milk; RCT, Africa; aged 6 months, supplementation for 12 months
(27) 40g milk; RCT, Africa; aged 6 months, supplementation for 12 months
(28) 40g no milk; RCT, Africa; aged 6 months, supplementation for 12 months
(29) SQLNS-20 gm, cRCT, Africa; 6-24 month aged, duration NR
(30) SQLNS-20 gm-HIV positive, cRCT, Africa, Aged 6-18 month, supplementation for 12 months

Severe underweight

Study or Subgroup	log[Risk Ratio]	SE	Child complementary feeding Total	Control Total	Weight	Risk Ratio IV, Random, 95% CI
**1.9.1 SQLNS**						
Ashorn 2015 (1)	0.2786	0.5316	222	220	23.1%	1.32 [0.47, 3.75]
Maleta 2015 (2)	0.0319	0.7592	247	51	11.3%	1.03 [0.23, 4.57]
Maleta 2015 (3)	-0.4637	0.64	265	50	15.9%	0.63 [0.18, 2.20]
Maleta 2015 (4)	-0.3339	0.6327	256	50	16.3%	0.72 [0.21, 2.47]
Maleta 2015 (5)	-0.0198	0.6133	255	50	17.4%	0.98 [0.29, 3.26]
Maleta 2015 (6)	-0.4447	0.6399	260	50	15.9%	0.64 [0.18, 2.25]
**Subtotal (95% CI)**			**1505**	**471**	**100.0%**	**0.87 [0.53, 1.44]**
Heterogeneity: Tau² = 0.00; Chi² = 1.29, df = 5 (P = 0.94); I² = 0%						
Test for overall effect: Z = 0.53 (P = 0.60)						
**1.9.2 Fortified Blended Foods**						
Mangani 2015 (7)	0.1117	0.4054	200	205	100.0%	1.12 [0.51, 2.48]
**Subtotal (95% CI)**			**200**	**205**	**100.0%**	**1.12 [0.51, 2.48]**
Heterogeneity: Not applicable						
Test for overall effect: Z = 0.28 (P = 0.78)						
**1.9.3 Locally Produced Ready to Use Supplementary Food**						
Mangani 2015 (8)	-0.1823	0.5021	206	103	62.3%	0.83 [0.31, 2.23]
Mangani 2015 (9)	-1.2234	0.7202	208	102	37.7%	0.29 [0.07, 1.21]
**Subtotal (95% CI)**			**414**	**205**	**100.0%**	**0.56 [0.21, 1.51]**
Heterogeneity: Tau² = 0.16; Chi² = 1.41, df = 1 (P = 0.24); I² = 29%						
Test for overall effect: Z = 1.14 (P = 0.25)						

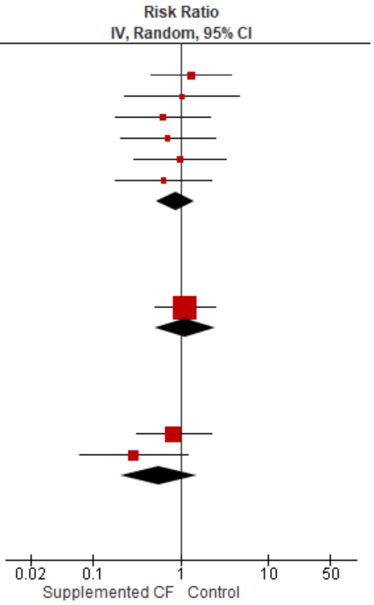

Test for subgroup differences: Chi² = 1.13, df = 2 (P = 0.57), I² = 0%

Footnotes
(1) RCT; Malawi; children aged 6-18 months; supplementaion for 6 months
(2) 10g milk LNS; RCT, Africa; aged 6 months, supplementation for 12 months
(3) 40g milk; RCT, Africa; aged 6 months, supplementation for 12 months
(4) 40g no milk; RCT, Africa; aged 6 months, supplementation for 12 months
(5) 20g milk; RCT, Africa; aged 6 months, supplementation for 12 months
(6) 20g no milk; RCT, Africa; aged 6 months, supplementation for 12 months
(7) CSB- RCT, Africa, age 6-18 months, suplementation for 12 month
(8) soy- RCT, Africa, age 6-18 months, suplementation for 12 month
(9) milk- RCT, Africa, age 6-18 months, suplementation for 12 month

Change in weight

Study or Subgroup	Mean Difference	SE	Child complementary feeding Total	Control Total	Weight	Mean Difference IV, Random, 95% CI
**1.10.1 Alternative food**						
Bhandari 2001 (1)	0.1	0.1122	87	93	100.0%	0.10 [-0.12, 0.32]
**Subtotal (95% CI)**			**87**	**93**	**100.0%**	**0.10 [-0.12, 0.32]**
Heterogeneity: Not applicable						
Test for overall effect: Z = 0.89 (P = 0.37)						
**1.10.2 Fortified Blended Food**						
Borg 2020 (2)	0.03	0.1071	59	77	12.1%	0.03 [-0.18, 0.24]
Christian 2015 (3)	0.02	0.0102	513	843	20.5%	0.02 [0.00, 0.04]
Lutter 2008 (4)	0.22	0.1389	170	149	9.5%	0.22 [-0.05, 0.49]
Mangani 2015 (5)	-0.1	0.0862	183	185	14.2%	-0.10 [-0.27, 0.07]
Obatolu 2003 (6)	2.55	3.7482	15	15	0.0%	2.55 [-4.80, 9.90]
Obatolu 2003 (7)	3.91	3.761	15	15	0.0%	3.91 [-3.46, 11.28]
Oelofse 2003 (8)	-0.2044	0.3671	16	14	2.2%	-0.20 [-0.92, 0.52]
Phu 2012 (9)	0.15	0.1457	135	64	9.0%	0.15 [-0.14, 0.44]
Phu 2012 (10)	-0.04	0.1505	114	64	8.7%	-0.04 [-0.33, 0.25]
Samuel-2018 (11)	0.3	0.0561	1025	1052	17.3%	0.30 [0.19, 0.41]
Sazawal 2014 (12)	0.2699	0.1901	59	53	6.5%	0.27 [-0.10, 0.64]
**Subtotal (95% CI)**			**2304**	**2531**	**100.0%**	**0.09 [-0.02, 0.20]**
Heterogeneity: Tau² = 0.02; Chi² = 32.67, df = 10 (P = 0.0003); I² = 69%						
Test for overall effect: Z = 1.56 (P = 0.12)						
**1.10.3 Locally Produced Ready to Use Supplementary Food**						
Borg 2020 (13)	0.07	0.0969	76	77	1.1%	0.07 [-0.12, 0.26]
Christian 2015 (14)	0.04	0.0141	513	421	52.3%	0.04 [0.01, 0.07]
Christian 2015 (15)	0.02	0.0153	503	422	44.4%	0.02 [-0.01, 0.05]
Mangani 2015 (16)	0.11	0.0969	191	92	1.1%	0.11 [-0.08, 0.30]
Mangani 2015 (17)	0.04	0.102	188	93	1.0%	0.04 [-0.16, 0.24]
**Subtotal (95% CI)**			**1471**	**1105**	**100.0%**	**0.03 [0.01, 0.05]**
Heterogeneity: Tau² = 0.00; Chi² = 1.74, df = 4 (P = 0.78); I² = 0%						
Test for overall effect: Z = 3.16 (P = 0.002)						
**1.10.4 Commercially Produced Ready to Use Supplementary Food**						
Christian 2015 (18)	0.04	0.0141	896	843	99.2%	0.04 [0.01, 0.07]
Santos 2005 (19)	-0.0128	0.1528	91	81	0.8%	-0.01 [-0.31, 0.29]
**Subtotal (95% CI)**			**987**	**924**	**100.0%**	**0.04 [0.01, 0.07]**
Heterogeneity: Tau² = 0.00; Chi² = 0.12, df = 1 (P = 0.73); I² = 0%						
Test for overall effect: Z = 2.82 (P = 0.005)						
**1.10.5 SQ LNS**						
Adu-Afarwuah 2016 (20)	0	0.0867	334	339	8.2%	0.00 [-0.17, 0.17]
Ashorn 2015 (21)	-0.3	0.102	195	200	8.1%	-0.30 [-0.50, -0.10]
Dewey 2017 (22)	-0.88	0.0102	884	408	8.3%	-0.88 [-0.90, -0.86]
Dewey 2017 (23)	0.02	0.0102	785	408	8.3%	0.02 [0.00, 0.04]
Hess 2015 (24)	0.29	0.051	1623	714	8.3%	0.29 [0.19, 0.39]
Maleta 2015 (25)	0	0.4082	242	48	5.8%	0.00 [-0.80, 0.80]
Maleta 2015 (26)	-0.1	0.4082	245	48	5.8%	-0.10 [-0.90, 0.70]
Maleta 2015 (27)	0	0.4031	220	49	5.9%	0.00 [-0.79, 0.79]
Maleta 2015 (28)	-0.1	0.4082	235	48	5.8%	-0.10 [-0.90, 0.70]
Maleta 2015 (29)	0	0.4082	238	48	5.8%	0.00 [-0.80, 0.80]
Muslihah 2016 (30)	1.6512	0.2729	58	25	7.0%	1.65 [1.12, 2.19]
Muslihah 2016 (31)	0.2234	0.236	59	26	7.3%	0.22 [-0.24, 0.69]
Smuts 2019 (32)	0.1	0.1804	162	101	7.7%	0.10 [-0.25, 0.45]
Smuts 2019 (33)	-0.2	0.1676	151	100	7.8%	-0.20 [-0.53, 0.13]
**Subtotal (95% CI)**			**5431**	**2562**	**100.0%**	**0.04 [-0.31, 0.39]**
Heterogeneity: Tau² = 0.39; Chi² = 4190.33, df = 13 (P < 0.00001); I² = 100%						
Test for overall effect: Z = 0.22 (P = 0.82)						

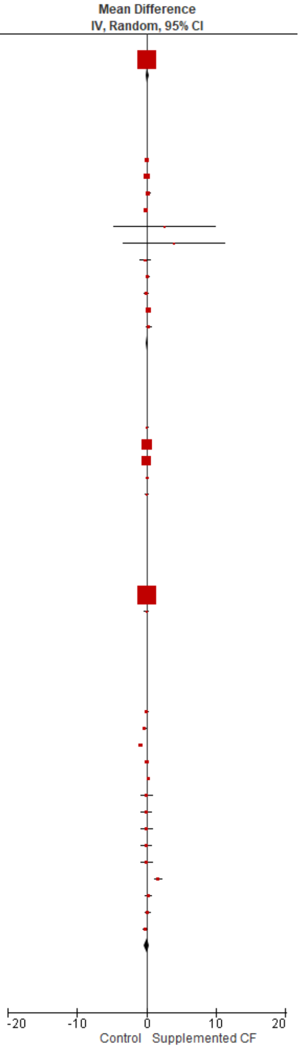

Test for subgroup differences: Chi² = 1.41, df = 4 (P = 0.84), I² = 0%

Footnotes
(1) RCT, South-East Asian Region, 4 and 12 mo of age, supplementation for 6 months intervention; actual weight
(2) CSB++ cm; cRCT, Western Pacific Region, 6months of age, supplementation for 6-12 months, change in weight
(3) WSB++ ; cRCT, South-East Asian Region, age-6-18 months; supplementation for 12 months; change in weight
(4) Non-RCT, Region of the Americas, 6-11 months age; supplementation for 11 months; weight at endline
(5) CSB- RCT, Africa, age 6-18 months, suplementation for 12 month; change in weight
(6) females 18 months-FBF
(7) males 18 months-FBF
(8) RCT: South Africa; children aged 6-12 months; supplementation for 6 months; endline
(9) fortified flour; RCT, Western Pacific Region, 5-29 months age; supplementation for 11 months, endline
(10) Food complement; RCT, Western Pacific Region, 5-29 months age; supplementation for 11 months, endline
(11) Non RCT, Africa, 6-23 months age, Duration of intervention NR; actual weight at endline
(12) RCT: India; children aged 6-24 months; supplementation for 6 months; change in weight
(13) RUSF (fish based); cRCT, Western Pacific Region, 6months age, supplementation for 6-12 months; change in weight
(14) Chickpeas; cRCT, South-East Asian Region, age-6-18 months; supplementation for 12 months; change in weight
(15) rice/lentils; cRCT, South-East Asian Region, age-6-18 months; supplementation for 12 months; change in weight
(16) Milk; RCT, Africa, age 6-18 months; supplementation for 12 months, change in weight
(17) Soy LNS; RCT, Africa, age 6-18 months; supplementation for 12 months; change in weight
(18) Plumpy doz- cRCT, South-East Asian Region, age-6-18 months; supplementation for 12 months; change in weight
(19) non-RCT: Brazil; children aged 6-18 months; change in weight
(20) change in weight was measured at 18 months of intervention-maternal and child; SQ-LNS; RCT; African region; mothers with >20 wk of gestation; supplementation for 6...
(21) SQLNS; RCT; Malawi; children aged 6-18 months; supplementaion for 6 months, change in weight
(22) SQLNS; cRCT, South-East Asian; 6-24 months age, weight gain in g/6mo; mother and child, endline
(23) SQLNS; cRCT, South-East Asian; 6-24 months age, weight gain in child g/6mo, endline
(24) cRCT, zinc, Africa, aged 9-18 months of age, supplementation for 9 months, actual weight at endline
(25) 40g milk; RCT, Africa; aged 6 months, supplementation for 12 months, change in wt
(26) 20g no milk; RCT, Africa; aged 6 months, supplementation for 12 months, change in wt
(27) 10g milk; RCT, Africa; aged 6 months, supplementation for 12 months, change in wt
(28) 20g milk; RCT, Africa; aged 6 months, supplementation for 12 months, change in wt
(29) 40g no milk; RCT, Africa; aged 6 months, supplementation for 12 months, change in wt
(30) SQ-LNS- non-RCT: Indonesia; Children aged 6-12 months; supplementation for 6 months; at endline
(31) Biscuits; non-RCT: Indonesia; Children aged 6-12 months; supplementation for 6 months; at endline
(32) SQ LNS plus; RCT, Africa; aged 6-12 months, supplementation for 6 months, endline
(33) SQ LNS; RCT, Africa; aged 6-12 months, supplementation for 6 months, endline

Change in height

Study or Subgroup	Mean Difference	SE	Child complementary feeding Total	Control Total	Weight	Mean Difference IV, Random, 95% CI
**1.11.1 Alternative food**						
Bauserman 2015 (1)	0	0.0616	34	35	97.6%	0.00 [-0.12, 0.12]
Bhandari 2001 (2)	0.1	0.3951	87	93	2.4%	0.10 [-0.67, 0.87]
**Subtotal (95% CI)**			**121**	**128**	**100.0%**	**0.00 [-0.12, 0.12]**
Heterogeneity: $Tau^2 = 0.00$; $Chi^2 = 0.06$, df = 1 (P = 0.80); $I^2 = 0\%$						
Test for overall effect: Z = 0.04 (P = 0.97)						
**1.11.2 Fortified Blended Food**						
Borg 2020 (3)	-0.3	0.3041	59	77	11.0%	-0.30 [-0.90, 0.30]
Christian 2015 (4)	0.01	0.0283	513	843	15.7%	0.01 [-0.05, 0.07]
Lutter 2008 (5)	0.3	0.4088	170	149	8.8%	0.30 [-0.50, 1.10]
Mangani 2015 (6)	-0.1	0.242	183	185	12.3%	-0.10 [-0.57, 0.37]
Obatolu 2003 (7)	4.97	19.1687	30	30	0.0%	4.97 [-32.60, 42.54]
Oelofse 2003 (8)	-0.1	0.9428	16	14	3.0%	-0.10 [-1.95, 1.75]
Phu 2012 (9)	0.5	0.2775	135	64	11.5%	0.50 [-0.04, 1.04]
Phu 2012 (10)	0.2	0.2766	114	64	11.6%	0.20 [-0.34, 0.74]
Samuel-2018 (11)	1.2	0.1733	1025	1052	13.8%	1.20 [0.86, 1.54]
Sazawal 2014 (12)	0.4	0.2457	59	53	12.3%	0.40 [-0.08, 0.88]
**Subtotal (95% CI)**			**2304**	**2531**	**100.0%**	**0.28 [-0.08, 0.63]**
Heterogeneity: $Tau^2 = 0.21$; $Chi^2 = 53.15$, df = 9 (P < 0.00001); $I^2 = 83\%$						
Test for overall effect: Z = 1.52 (P = 0.13)						
**1.11.3 Locally Produced Ready to Use Supplementary Food**						
Borg 2020 (13)	-0.1	0.2516	76	77	0.6%	-0.10 [-0.59, 0.39]
Christian 2015 (14)	0.08	0.0283	513	421	48.7%	0.08 [0.02, 0.14]
Christian 2015 (15)	0.09	0.0283	503	422	48.7%	0.09 [0.03, 0.15]
Mangani 2015 (16)	0.2	0.1917	191	92	1.1%	0.20 [-0.18, 0.58]
Mangani 2015 (17)	0	0.2071	188	93	0.9%	0.00 [-0.41, 0.41]
**Subtotal (95% CI)**			**1471**	**1105**	**100.0%**	**0.08 [0.05, 0.12]**
Heterogeneity: $Tau^2 = 0.00$; $Chi^2 = 1.13$, df = 4 (P = 0.89); $I^2 = 0\%$						
Test for overall effect: Z = 4.27 (P < 0.0001)						
**1.11.4 Commercially Produced Ready to Use Supplementary Food**						
Christian 2015 (18)	0.06	0.0283	896	843	99.6%	0.06 [0.00, 0.12]
Santos 2005 (19)	-0.23	0.4489	91	81	0.4%	-0.23 [-1.11, 0.65]
**Subtotal (95% CI)**			**987**	**924**	**100.0%**	**0.06 [0.00, 0.11]**
Heterogeneity: $Tau^2 = 0.00$; $Chi^2 = 0.42$, df = 1 (P = 0.52); $I^2 = 0\%$						
Test for overall effect: Z = 2.08 (P = 0.04)						
**1.11.5 SQ LNS**						
Adu-Afarwuah 2016 (20)	0.2	0.193	334	337	7.2%	0.20 [-0.18, 0.58]
Ashorn 2015 (21)	-0.7	0.2667	195	200	6.5%	-0.70 [-1.22, -0.18]
Dewey 2017 (22)	0.1	0.0366	785	408	8.1%	0.10 [0.03, 0.17]
Dewey 2017 (23)	0.1	0.0359	884	408	8.1%	0.10 [0.03, 0.17]
Hess 2015 (24)	0.7	0.1663	1623	523	7.4%	0.70 [0.37, 1.03]
Maleta 2015 (25)	0.2	0.1859	222	49	7.2%	0.20 [-0.16, 0.56]
Maleta 2015 (26)	0	0.1957	242	48	7.2%	0.00 [-0.38, 0.38]
Maleta 2015 (27)	0.1	0.1919	238	48	7.2%	0.10 [-0.28, 0.48]
Maleta 2015 (28)	0.1	0.1879	235	48	7.2%	0.10 [-0.27, 0.47]
Maleta 2015 (29)	0	0.1861	245	48	7.2%	0.00 [-0.36, 0.36]
Muslihah 2016 (30)	0.6	0.0504	59	26	8.0%	0.60 [0.50, 0.70]
Muslihah 2016 (31)	1.4	0.0512	58	25	8.0%	1.40 [1.30, 1.50]
Smuts 2019 (32)	-0.5	0.3976	151	100	5.2%	-0.50 [-1.28, 0.28]
Smuts 2019 (33)	-0.1	0.3783	162	101	5.4%	-0.10 [-0.84, 0.64]
**Subtotal (95% CI)**			**5433**	**2369**	**100.0%**	**0.19 [-0.11, 0.49]**
Heterogeneity: $Tau^2 = 0.29$; $Chi^2 = 575.96$, df = 13 (P < 0.00001); $I^2 = 98\%$						
Test for overall effect: Z = 1.27 (P = 0.20)						

Mean Difference IV, Random, 95% CI

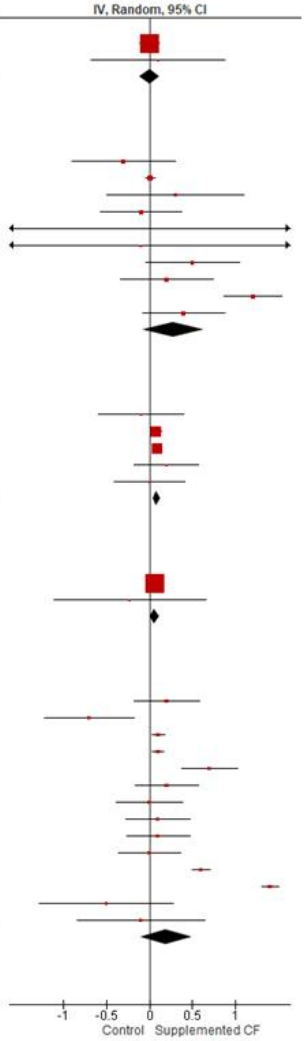

Test for subgroup differences: $Chi^2 = 3.80$, df = 4 (P = 0.43), $I^2 = 0\%$

Footnotes

(1) cRCT, Africa, children aged 5 months; supplementation for18 months, endline
(2) RCT, South-East Asian Region, 4 and 12 mo of age, supplementation for 6 months
(3) CSB++; cRCT, Western Pacific Region, 6months age, supplementation for 6-12 , change in height
(4) WSB++ ; cRCT, South-East Asian Region, age-6-18 months; supplementation for 12 months, change in height
(5) Non-RCT, Region of the Americas, 6-11 months age; supplementation for 11 months, endline
(6) CSB- RCT, Africa, age 6-18 months, suplementation for 12 month, change in height
(7) non-RCT; Africa; children aged 4 months; supplementation for 5 months
(8) RCT: South Africa; children aged 6-12 months; supplementation for 6 months; endline
(9) Fortified flour; RCT, Western Pacific Region, 5-29 months age; supplementation for 11 months, endline
(10) Food complement ; RCT, Western Pacific Region, 5-29 months age; supplementation for 11 months, endline
(11) Non RCT, Africa, Rural, 6-23 months age, endline
(12) RCT: India; children aged 6-24 months; supplementation for 6 months; change in height
(13) RUSF (fish based); cRCT, Western Pacific Region, 6months age, supplementation for 6-12 months, change in height
(14) cRCT, chickpeas; South East Asia; aged 6-18 months; supplementation for 12 months, change in height
(15) cRCT, rice/lentils;South East Asia; aged 6-18 months; supplementation for 12 months, change in height
(16) Milk LNS; RCT, Africa, age 6-18 months; supplementation for 12 months, change in height
(17) Soy LNS; RCT, Africa, age 6-18 months; supplementation for 12 months, change in height
(18) Plumpy doz- cRCT; South East Asia; aged 6-18 months; supplementation for 12 months, change in height
(19) non-RCT: Brazil; children aged 6-18 months; change in length
(20) change in height, maternal and child supplementation; SQ-LNS; RCT; African region; mothers with >20 wk of gestation; supplementation for 12 months
(21) RCT; Malawi; children aged 6-18 months; supplementaion for 6 months, change in height
(22) SQ LNS; cRCT, South-East Asia; 6-24 months age, child supplementation for 18 months, height gain
(23) SQ LNS; cRCT, South-East Asia; children 6-24 months age, maternal (during pregnancy) and child supplementation for 18 months, height gain
(24) zinc, cRCT, Africa, aged 9-18 months of age, supplementation for 9 months
(25) 10g milk; RCT, Africa; aged 6 months, supplementation for 12 months; z scores, change in height
(26) 40g no milk; RCT, Africa; aged 6 months, supplementation for 12 months, change in height
(27) 40g milk; RCT, Africa; aged 6 months, supplementation for 12 months, change in height
(28) 20g milk; RCT, Africa; aged 6 months, supplementation for 12 months; z scores, change in height
(29) 20g no milk; RCT, Africa; aged 6 months, supplementation for 12 months; z scores, change in height
(30) biscuits- non-RCT: Indonesia; Children aged 6-12 months; supplementation for 6 months; at endline
(31) SQ-LNS- non-RCT: Indonesia; Children aged 6-12 months; supplementation for 6 months; at endline
(32) SQ LNS; RCT, Africa; aged 6-12 months, supplementation for 6 months, endline
(33) SQ LNS plus; RCT, Africa; aged 6-12 months, supplementation for 6 months, endline

MUAC (cm)

Study or Subgroup	Mean Difference	SE	Child complementary feeding Total	Control Total	Weight	Mean Difference IV, Random, 95% CI
**1.12.1 Fortified Blended Food**						
Akalu 2010 (1)	0.0439	0.1377	106	105	33.8%	0.04 [-0.23, 0.31]
Borg 2020 (2)	0.2	0.1378	59	77	33.8%	0.20 [-0.07, 0.47]
Owino 2007 (3)	0	0.1929	44	35	17.2%	0.00 [-0.38, 0.38]
Owino 2007 (4)	0.3784	0.2055	37	34	15.2%	0.38 [-0.02, 0.78]
**Subtotal (95% CI)**			**246**	**251**	**100.0%**	**0.14 [-0.02, 0.30]**
Heterogeneity: Tau² = 0.00; Chi² = 2.55, df = 3 (P = 0.47); I² = 0%						
Test for overall effect: Z = 1.75 (P = 0.08)						
**1.12.2 Locally Produced Ready to Use Supplementary Food**						
Borg 2020 (5)	0.1	0.1378	76	77	100.0%	0.10 [-0.17, 0.37]
**Subtotal (95% CI)**			**76**	**77**	**100.0%**	**0.10 [-0.17, 0.37]**
Heterogeneity: Not applicable						
Test for overall effect: Z = 0.73 (P = 0.47)						
**1.12.3 Commercially Produced Ready to Use Supplementary Food**						
Huybregts 2012 (6)	0.2	0.0918	386	284	100.0%	0.20 [0.02, 0.38]
**Subtotal (95% CI)**			**386**	**284**	**100.0%**	**0.20 [0.02, 0.38]**
Heterogeneity: Not applicable						
Test for overall effect: Z = 2.18 (P = 0.03)						
**1.12.4 SQ LNS**						
Adu-Afarwuah 2016 (7)	0.1	0.0765	348	353	10.1%	0.10 [-0.05, 0.25]
Becquey 2019 (8)	0	0.0867	885	896	9.0%	0.00 [-0.17, 0.17]
Hess 2015 (9)	0.3	0.0561	1623	523	12.7%	0.30 [0.19, 0.41]
Hurley 2021 (10)	0.018	0.0042	176	191	18.1%	0.02 [0.01, 0.03]
Huybregts 2019 (11)	0.1	0.0459	1154	1159	14.1%	0.10 [0.01, 0.19]
Maleta 2015 (12)	0.2	0.148	242	48	4.6%	0.20 [-0.09, 0.49]
Maleta 2015 (13)	0.1	0.1429	235	48	4.8%	0.10 [-0.18, 0.38]
Maleta 2015 (14)	0.2	0.1429	245	48	4.8%	0.20 [-0.08, 0.48]
Maleta 2015 (15)	0.1	0.1429	220	49	4.8%	0.10 [-0.18, 0.38]
Maleta 2015 (16)	0.1	0.1429	238	48	4.8%	0.10 [-0.18, 0.38]
Muslihah 2016 (17)	0	0.2393	58	25	2.1%	0.00 [-0.47, 0.47]
Muslihah 2016 (18)	0	0.2354	59	26	2.1%	0.00 [-0.46, 0.46]
Smuts 2019 (19)	0.1	0.1627	162	101	4.0%	0.10 [-0.22, 0.42]
Smuts 2019 (20)	-0.1	0.1684	151	100	3.8%	-0.10 [-0.43, 0.23]
**Subtotal (95% CI)**			**5796**	**3615**	**100.0%**	**0.10 [0.03, 0.17]**
Heterogeneity: Tau² = 0.01; Chi² = 34.05, df = 13 (P = 0.001); I² = 62%						
Test for overall effect: Z = 2.71 (P = 0.007)						

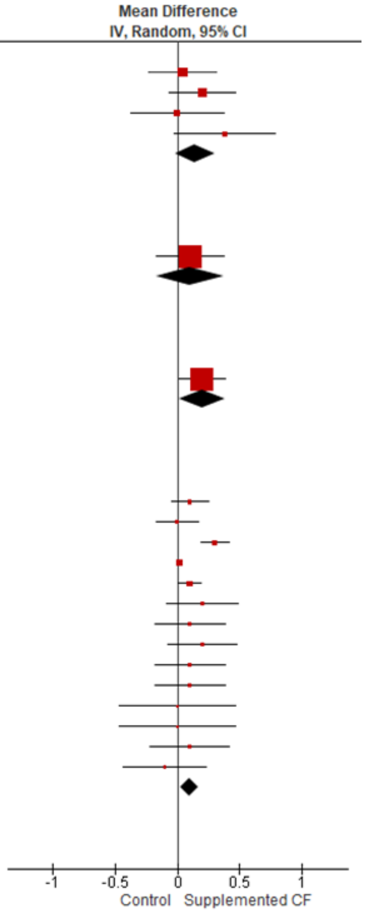

Test for subgroup differences: Chi² = 1.15, df = 3 (P = 0.77), I² = 0%

Footnotes

(1) RCT: Africa; children aged 5-17 months; supplementation for 1 year; Sibu Siri; endline MUAC
(2) CSB++ cm; RCT, Western Pacific Region, 6months of age, supplementation for 6-12 months, Change in MUAC
(3) CBMA; RCT: Africa; Children aged 6 months; supplementation for 3 months, endline
(4) CBM; RCT: Africa; Children aged 6 months; supplementation for 3 months, endline
(5) RUSF (fish based); cRCT, Western Pacific Region, 6months age, supplementation for 6-12 months, endline MUAC
(6) Plumpydoz Nutriset 46 g ~247Kcal/ d; cRCT, Africa; aged 6-36 months, supplementation for 4months; endline MUAC
(7) SQ-LNS; RCT; African region; mothers with >20 wk of gestation; supplementation for 6 months, endline
(8) SQLNS 20gm; cRCT: Africa; children aged 0-17 months; endline
(9) zinc, cRCT; Africa, aged 9-18 months of age, supplementation for 9 months, acutal MUAC at endline
(10) non-RCT, Africa; children 6-24 months of age, supplementation for 6 months; change in MUAC
(11) cRCT; Africa; age 6-23 months, supplementation for 12 months, endline
(12) 40g milk; RCT, Africa; aged 6 months, supplementation for 12 months; change in MUAC
(13) 20g milk; RCT, Africa; aged 6 months, supplementation for 12 months; death during 6-12 months of age; change in MUAC
(14) 20g no milk; RCT, Africa; aged 6 months, supplementation for 12 months; death during 6-12 months of age; change in MUAC
(15) 10g milk; RCT, Africa; aged 6 months, supplementation for 12 months; death during 6-12 months of age; change in MUAC
(16) 40g no milk; RCT, Africa; aged 6 months, supplementation for 12 months; change in MUAC
(17) SQ-LNS- non-RCT: Indonesia; Children aged 6-12 months; supplementation for 6 months; at endline
(18) biscuits- non-RCT: Indonesia; Children aged 6-12 months; supplementation for 6 months; at endline
(19) SQ LNS plus; RCT, Africa; aged 6-12 months, supplementation for 6 months, endline
(20) SQ-LNS; RCT, Africa, aged 6-12 months , supplementation for 12 months, endline

MUAC (z-score)

Study or Subgroup	Mean Difference	SE	Child complementary feeding Total	Control Total	Weight	Mean Difference IV, Random, 95% CI
**1.13.1 SQ LNS**						
Ashorn 2015 (1)	-0.06	0.0918	214	220	6.4%	-0.06 [-0.24, 0.12]
Dewey 2017 (2)	0.04	0.0408	884	408	32.3%	0.04 [-0.04, 0.12]
Dewey 2017 (3)	0.04	0.0408	785	408	32.3%	0.04 [-0.04, 0.12]
Humphrey 2019 (4)	0.02	0.0663	356	348	12.2%	0.02 [-0.11, 0.15]
Maleta 2015 (5)	0.09	0.1339	235	48	3.0%	0.09 [-0.17, 0.35]
Maleta 2015 (6)	0.14	0.1345	245	48	3.0%	0.14 [-0.12, 0.40]
Maleta 2015 (7)	0.07	0.1327	220	49	3.1%	0.07 [-0.19, 0.33]
Maleta 2015 (8)	0.05	0.1345	238	48	3.0%	0.05 [-0.21, 0.31]
Maleta 2015 (9)	0.17	0.1355	242	48	2.9%	0.17 [-0.10, 0.44]
Prendergast 2019 (10)	0	0.1684	59	58	1.9%	0.00 [-0.33, 0.33]
**Subtotal (95% CI)**			**3478**	**1683**	**100.0%**	**0.04 [-0.01, 0.09]**

Heterogeneity: Tau² = 0.00; Chi² = 3.00, df = 9 (P = 0.96); I² = 0%

Test for overall effect: Z = 1.72 (P = 0.09)

Mean Difference IV, Random, 95% CI

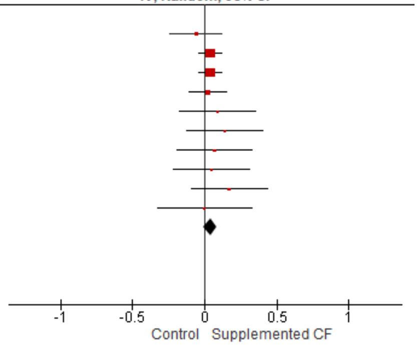

Test for subgroup differences: Not applicable

Footnotes

(1) RCT; Malawi; children aged 6-18 months; supplementaion for 6 months; endline

(2) SQ LNS; cRCT, South-East Asia; 6-24 months age, maternal (during pregnancy) and child supplementation for 18 months; actual MUAC Z score; MUAC was measured at endline

(3) SQ LNS; cRCT, South-East Asia; 6-24 months age, supplementation for 18 months; actual MUAC Z score at endline

(4) SQLNS 20 gm-HIV negative, cRCT, Africa, Aged 6-18 month, supplementation for 12 months; endline

(5) 20g milk; RCT, Africa; aged 6 months, supplementation for 12 months; death during 6-12 months of age; change in MUAC

(6) 20g no milk; RCT, Africa; aged 6 months, supplementation for 12 months; death during 6-12 months of age; change in MUAC

(7) 10g milk; RCT, Africa; aged 6 months, supplementation for 12 months; death during 6-12 months of age; change in MUAC

(8) 40g no milk; RCT, Africa; aged 6 months, supplementation for 12 months; change in MUAC

(9) 40g milk; RCT, Africa; aged 6 months, supplementation for 12 months; change in MUAC

(10) SQLNS-20 gm-HIV positive, cRCT, Africa, Aged 6-18 month, supplementation for 12 months, endline

Hb (g/dl)

Study or Subgroup	Std. Mean Difference	SE	Child complementary feeding Total	Control Total	Weight	Std. Mean Difference IV, Random, 95% CI
**1.16.1 Alternative food**						
Bauserman 2015 (1)	0.3524	0.1705	34	28	6.5%	0.35 [0.02, 0.69]
**Subtotal (95% CI)**			**34**	**28**	**6.5%**	**0.35 [0.02, 0.69]**
Heterogeneity: Not applicable						
Test for overall effect: Z = 2.07 (P = 0.04)						
**1.16.2 SQLNS**						
Addo 2020 (2)	−1	0.0042	654	653	6.7%	−1.00 [−1.01, −0.99]
Dewey 2017 (3)	0.2489	0.0873	257	136	6.7%	0.25 [0.08, 0.42]
Dewey 2017 (4)	0.3702	0.0849	293	136	6.7%	0.37 [0.20, 0.54]
Hess 2015 (5)	0.5896	0.0559	1307	443	6.7%	0.59 [0.48, 0.70]
Humphrey 2019 (6)	0.1687	0.0758	353	346	6.7%	0.17 [0.02, 0.32]
Prendergast 2019 (7)	0.1575	0.1876	58	56	6.5%	0.16 [−0.21, 0.53]
Smuts 2019 (8)	5	7.1837	162	101	0.1%	5.00 [−9.08, 19.08]
Smuts 2019 (9)	3	5.7553	151	100	0.2%	3.00 [−8.28, 14.28]
**Subtotal (95% CI)**			**3235**	**1971**	**40.3%**	**0.12 [−0.67, 0.91]**
Heterogeneity: $Tau^2 = 0.97$; $Chi^2 = 1529.71$, df = 7 (P < 0.00001); $I^2 = 100\%$						
Test for overall effect: Z = 0.29 (P = 0.77)						
**1.16.3 Fortified Blended Food**						
Galasso 2019 (10)	4	1.1348	18	19	3.0%	4.00 [1.78, 6.22]
Lutter 2008 (11)	0.513	0.1141	170	149	6.6%	0.51 [0.29, 0.74]
Oelofse 2003 (12)	0.2	0.4139	16	14	5.8%	0.20 [−0.61, 1.01]
Owino 2007 (13)	0.6	0.2876	44	35	6.2%	0.60 [0.04, 1.16]
Owino 2007 (14)	0.7	0.2674	37	34	6.3%	0.70 [0.18, 1.22]
Samuel-2018 (15)	0.06	0.4008	1025	1052	5.8%	0.06 [−0.73, 0.85]
Sazawal 2014 (16)	1.06	0.272	66	48	6.3%	1.06 [0.53, 1.59]
**Subtotal (95% CI)**			**1376**	**1351**	**40.1%**	**0.64 [0.29, 1.00]**
Heterogeneity: $Tau^2 = 0.12$; $Chi^2 = 15.27$, df = 6 (P = 0.02); $I^2 = 61\%$						
Test for overall effect: Z = 3.58 (P = 0.0003)						
**1.16.4 Commercially Produced Ready to Use Supplementary Food**						
Huybregts 2012 (17)	0.2245	0.0808	370	264	6.7%	0.22 [0.07, 0.38]
Siega-Riz 2014 (18)	2.7119	0.2408	74	61	6.4%	2.71 [2.24, 3.18]
**Subtotal (95% CI)**			**444**	**325**	**13.1%**	**1.46 [−0.98, 3.90]**
Heterogeneity: $Tau^2 = 3.06$; $Chi^2 = 95.91$, df = 1 (P < 0.00001); $I^2 = 99\%$						
Test for overall effect: Z = 1.17 (P = 0.24)						
**Total (95% CI)**			**5089**	**3675**	**100.0%**	**0.58 [0.05, 1.10]**
Heterogeneity: $Tau^2 = 1.06$; $Chi^2 = 2375.92$, df = 17 (P < 0.00001); $I^2 = 99\%$						
Test for overall effect: Z = 2.16 (P = 0.03)						
Test for subgroup differences: $Chi^2 = 2.81$, df = 3 (P = 0.42), $I^2 = 0\%$						

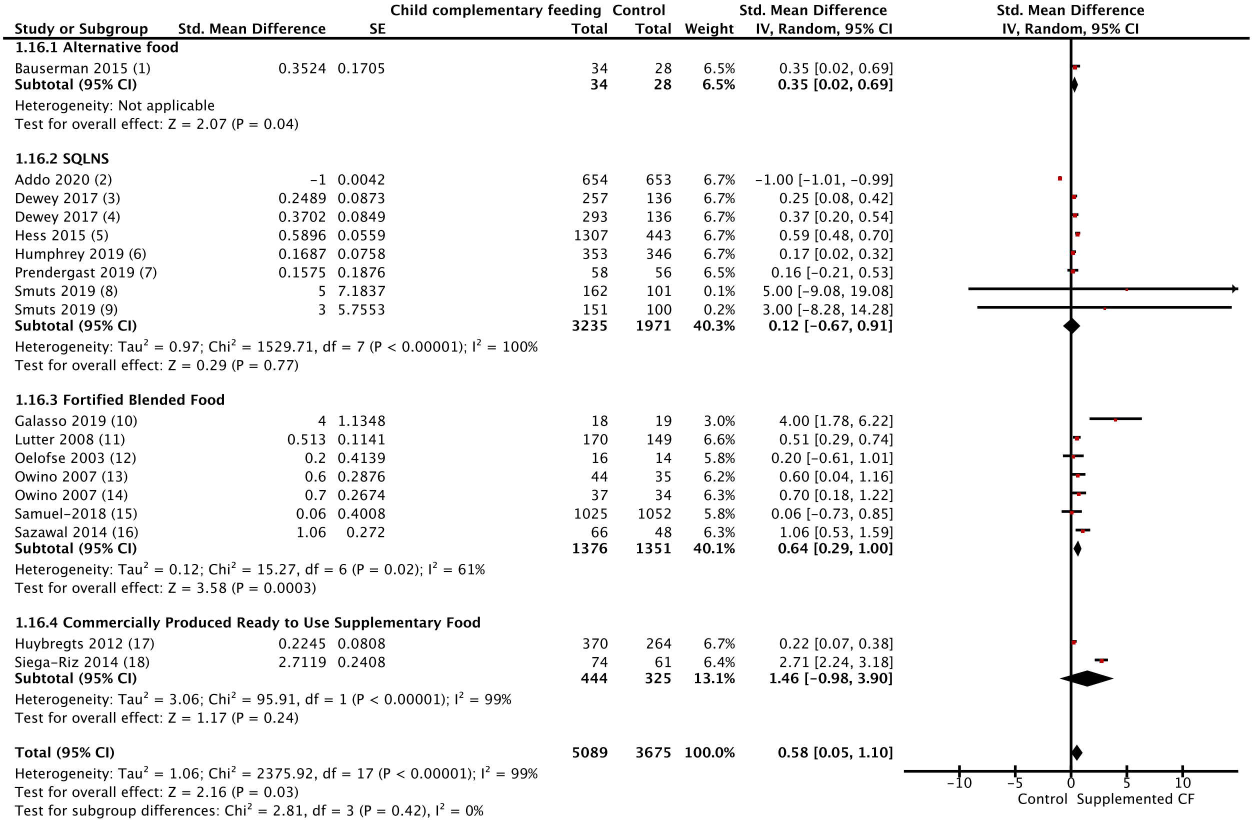

Footnotes
(1) cRCT, Africa, children aged 5 months; supplementation for18 months
(2) non-RCT; Africa; 6-18 months age, supplementation for 3 months
(3) SQ LNS; cRCT, South-East Asia; 6-24 months age and their mothers, supplementation for 18 months
(4) SQ LNS; cRCT, South-East Asia; 6-24 months age and their mothers, maternal (during pregnancy) and child supplementation for 18 months
(5) zinc, cRCT, Africa, aged 9-18 months of age, supplementation for 9 months
(6) SQLNS 20 gm-HIV negative- cRCT, Africa, Aged 6-18 month, supplementation for 12 months, endline
(7) SQLNS-20 gm-HIV positive- cRCT, Africa, Aged 6-18 month, supplementation for 12 months, endline
(8) SQ LNS; RCT, Africa; aged 6-12 months, supplementation for 6 months
(9) SQ LNS plus; RCT, Africa; aged 6-12 months, supplementation for 6 months
(10) RCT; Madasgar; Children <12 years; supplementation for 12 months
(11) Non-RCT, Region of the Americas, 6-11 months age, supplementation for 11 months, endline
(12) RCT: South Africa; children aged 6-12 months; supplementation for 6 months; endline
(13) CBMA; RCT: Africa; Children aged 6 months; supplementation for 3 months
(14) CBM; RCT: Africa; Children aged 6 months; supplementation for 3 months
(15) Non RCT, Africa, 6-23 months age
(16) RCT: India; children aged 6-24 months; supplementation for 6 months; change in Hb levels
(17) Plumpydoz Nutriset 46 g ~247Kcal/ d; RCT, Africa, aged 6-36 months, supplementation for 4 months, endline
(18) RCT; ntibucá, Honduras; children aged 6-18 months; supplementation for 12 months

Anemia

Study or Subgroup	log[Risk Ratio]	SE	Child complementary feeding Total	Control Total	Weight	Risk Ratio IV, Random, 95% CI
**1.15.1 Alternative food**						
Bauserman 2015 (1)	−0.6549	0.2294	34	28	100.0%	0.52 [0.33, 0.81]
**Subtotal (95% CI)**			**34**	**28**	**100.0%**	**0.52 [0.33, 0.81]**
Heterogeneity: Not applicable						
Test for overall effect: Z = 2.85 (P = 0.004)						
**1.15.2 Fortified Blended Food**						
Glasso 2019 (2)	−0.6391	0.5165	18	19	2.5%	0.53 [0.19, 1.45]
Owino 2007 (3)	−0.2072	0.1597	37	34	14.4%	0.81 [0.59, 1.11]
Lutter 2008 (4)	−0.4714	0.1544	170	149	14.9%	0.62 [0.46, 0.84]
Owino 2007 (5)	−0.1235	0.1381	44	35	16.5%	0.88 [0.67, 1.16]
Samuel–2018 (6)	−0.1732	0.0724	1025	1052	23.9%	0.84 [0.73, 0.97]
Zhang 2016 (7)	−0.455	0.0306	2174	754	27.8%	0.63 [0.60, 0.67]
**Subtotal (95% CI)**			**3468**	**2043**	**100.0%**	**0.74 [0.62, 0.87]**
Heterogeneity: $Tau^2 = 0.03; Chi^2 = 18.93, df = 5 (P = 0.002); I^2 = 74\%$						
Test for overall effect: Z = 3.56 (P = 0.0004)						
**1.15.3 Commercially Produced Ready to Use Supplementary Food**						
Siega–Riz 2014 (8)	−0.0521	0.1634	74	61	8.7%	0.95 [0.69, 1.31]
Huybregts 2012 (9)	−0.1657	0.0631	370	264	33.2%	0.85 [0.75, 0.96]
Khan 2020 (10)	−0.0298	0.0222	268	267	58.1%	0.97 [0.93, 1.01]
**Subtotal (95% CI)**			**712**	**592**	**100.0%**	**0.93 [0.84, 1.03]**
Heterogeneity: $Tau^2 = 0.00; Chi^2 = 4.13, df = 2 (P = 0.13); I^2 = 52\%$						
Test for overall effect: Z = 1.48 (P = 0.14)						
**1.15.4 SQ LNS**						
Prendergast 2019 (11)	−0.8824	0.6641	58	56	3.3%	0.41 [0.11, 1.52]
Humphrey 2019 (12)	−0.4044	0.2163	353	346	11.6%	0.67 [0.44, 1.02]
Smuts 2019 (13)	−0.4121	0.1973	151	100	12.2%	0.66 [0.45, 0.97]
Smuts 2019 (14)	−0.3143	0.1921	162	101	12.4%	0.73 [0.50, 1.06]
Dewey 2017 (15)	−0.3426	0.1445	293	136	13.9%	0.71 [0.53, 0.94]
Dewey 2017 (16)	−0.362	0.1401	257	136	14.0%	0.70 [0.53, 0.92]
Addo 2020 (17)	0.3934	0.0469	654	653	16.1%	1.48 [1.35, 1.62]
Hess 2015 (18)	−0.1406	0.0207	1307	443	16.4%	0.87 [0.83, 0.90]
**Subtotal (95% CI)**			**3235**	**1971**	**100.0%**	**0.80 [0.61, 1.04]**
Heterogeneity: $Tau^2 = 0.11; Chi^2 = 125.66, df = 7 (P < 0.00001); I^2 = 94\%$						
Test for overall effect: Z = 1.64 (P = 0.10)						

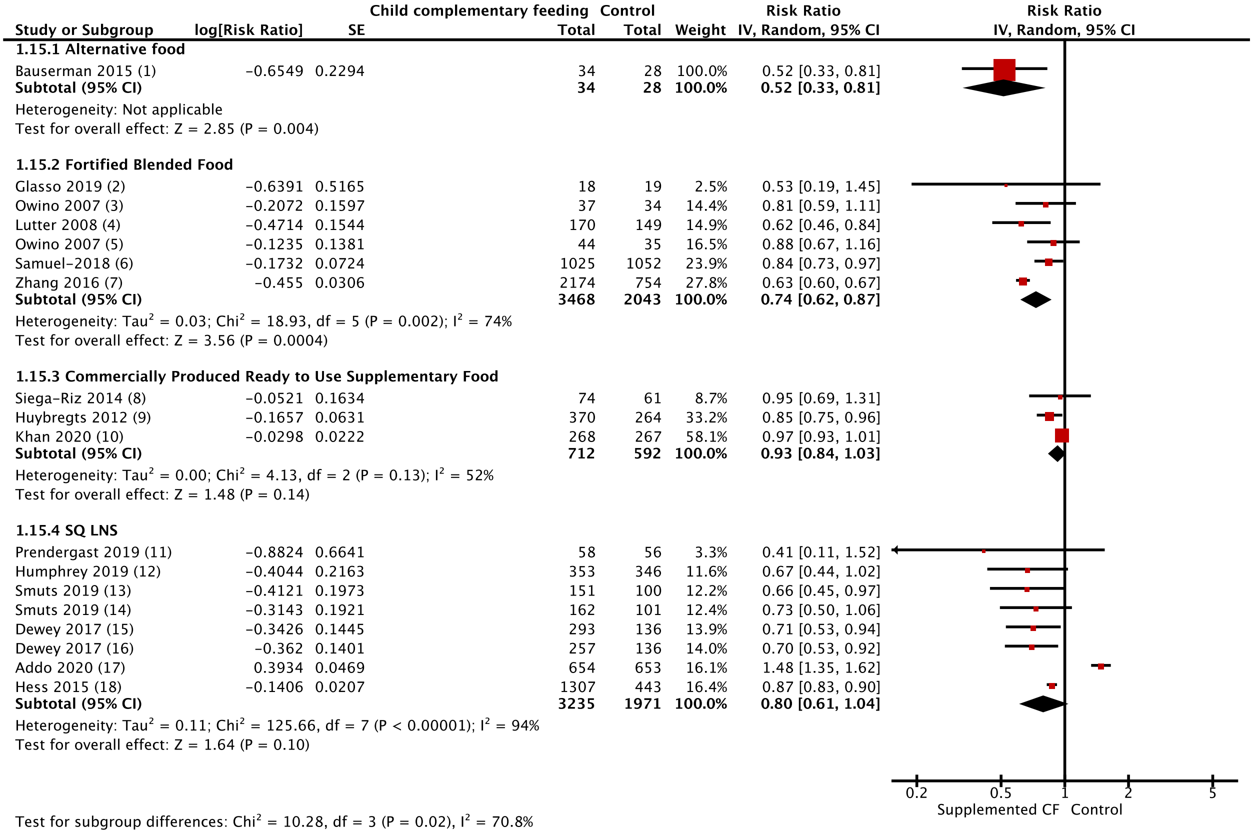

Test for subgroup differences: $Chi^2 = 10.28, df = 3 (P = 0.02), I^2 = 70.8\%$

Footnotes

(1) cRCT, Africa, aged 5 months; supplementation for 18 months; Hb <= 10g/dl
(2) RCT; Madasgar; Children <12 years; supplementation for 12 months; Hb <11g/dl
(3) CBM; RCT: Africa; Children aged 6 months; supplementation for intervention 3 months, <11g/dl, endline
(4) Non–RCT, Region of the Americas, 6–11 months age, supplementation for 11 months, endline, Hb <110g/l
(5) CBMA; RCT: Africa; Children aged 6 months; supplementation for intervention 3 months, <11g/dl, endline
(6) Non RCT, Africa, Rural, 6–23 months age
(7) RCT; Western Pacific; children aged 6–24 months; supplementation for intervention 2 years, endline
(8) RCT; ntibucá, Honduras; children aged 6–18 months; supplementation for 12 months
(9) Plumpydoz Nutriset 46 g ~247Kcal/ d; RCT, Africa, aged 6–36 months, supplementation for 4 months, endline
(10) MQ–LNS; RCT, EMRO, age 6–24 months, supplementation for 12 months; anemia
(11) SQLNS–20 gm–HIV positive– RCT, Africa, Aged 6–18 month, supplementation for 12 months, Hb <105 g/l
(12) SQLNS 20 gm–HIV negative– cRCT, Africa, Aged 6–18 month, supplementation for 12 months, Hb <105 g/l
(13) SQ LNS plus; RCT, Africa; aged 6–12 months, supplementation for 6 months
(14) SQ LNS; RCT, Africa; aged 6–12 months, supplementation for 6 months
(15) SQ LNS; cRCT, South–East Asia; 6–24 months age,maternal (during pregnancy) and child supplementation for 18 months; Hb <110g/l
(16) SQ LNS; cRCT, South–East Asia; 6–24 months age, supplementation for 18 months, Hb <110g/l
(17) Non RCT, Africa, 6–18 months age, supplementation for 3 months
(18) zinc, cRCT, Africa, aged 9–18 months of age, supplementation for 9 months, Hb <110g/l

Iron deficiency anemia

Study or Subgroup	log[Risk Ratio]	SE	Child complementary feeding Total	Control Total	Weight	Risk Ratio IV, Random, 95% CI
**1.14.1 Fortified Blended Food**						
Glasso 2019 (1)	-0.6391	0.6259	18	19	23.7%	0.53 [0.15, 1.80]
Samuel-2018 (2)	0.2097	0.1134	1025	1052	76.3%	1.23 [0.99, 1.54]
**Subtotal (95% CI)**			**1043**	**1071**	**100.0%**	**1.01 [0.50, 2.05]**
Heterogeneity: Tau² = 0.16; Chi² = 1.78, df = 1 (P = 0.18); I² = 44%						
Test for overall effect: Z = 0.02 (P = 0.98)						
**1.14.2 SQ LNS**						
Addo 2020 (3)	0.0032	0.0797	654	653	23.7%	1.00 [0.86, 1.17]
Dewey 2017 (4)	-0.8455	0.2031	293	136	21.5%	0.43 [0.29, 0.64]
Dewey 2017 (5)	-0.6111	0.1985	257	136	21.6%	0.54 [0.37, 0.80]
Smuts 2019 (6)	-1.3297	0.3776	162	100	17.1%	0.26 [0.13, 0.55]
Smuts 2019 (7)	-1.5008	0.4171	151	101	16.1%	0.22 [0.10, 0.50]
**Subtotal (95% CI)**			**1517**	**1126**	**100.0%**	**0.46 [0.26, 0.81]**
Heterogeneity: Tau² = 0.35; Chi² = 39.16, df = 4 (P < 0.00001); I² = 90%						
Test for overall effect: Z = 2.70 (P = 0.007)						

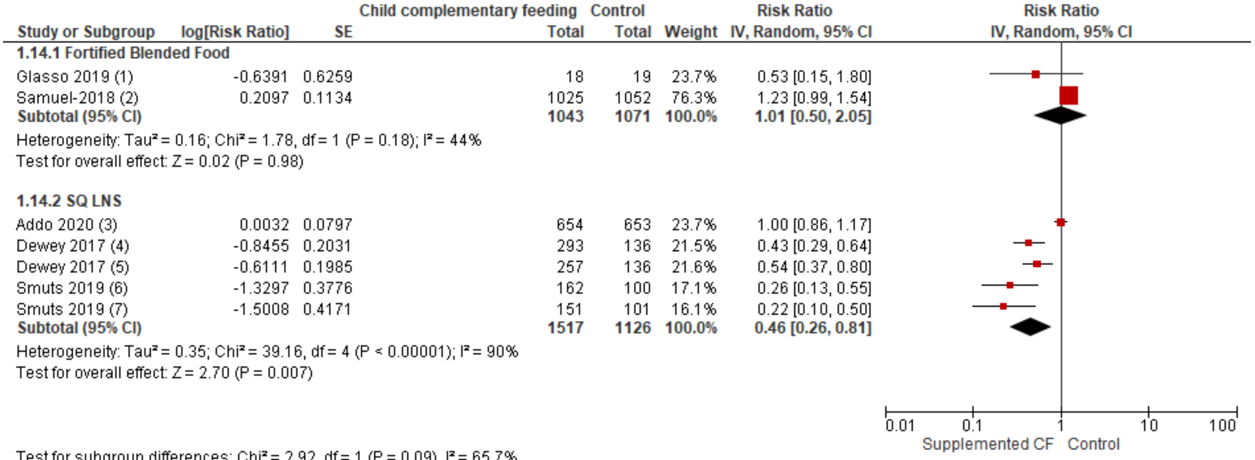

Test for subgroup differences: Chi² = 2.92, df = 1 (P = 0.09), I² = 65.7%

Footnotes

(1) RCT; Madasgar; Children <12 years; supplementation for 12 months, anaemia and iron deficient
(2) Non RCT, Africa, Rural, 6-23 months age
(3) Non RCT, Africa 6-18 months age, supplementation for 3 months
(4) SQ LNS; cRCT, South-East Asia; 6-24 months age,maternal (during pregnancy) and child supplementation for 18 months; Hb <110g/l
(5) SQ LNS; cRCT, South-East Asia; 6-24 months age, supplementation for 18 months, Hb <110g/l
(6) SQ LNS plus; RCT, Africa; aged 6-12 months, supplementation for 6 months
(7) SQ LNS; RCT, Africa; aged 6-12 months, supplementation for 6 months

Fever

Study or Subgroup	log[Risk Ratio]	SE	Child complementary feeding Total	Control Total	Weight	Risk Ratio IV, Random, 95% CI
**1.17.1 Alternative food**						
Iannoti 2017 (1)	0.2607	0.2649	75	73	100.0%	1.30 [0.77, 2.18]
**Subtotal (95% CI)**			**75**	**73**	**100.0%**	**1.30 [0.77, 2.18]**
Heterogeneity: Not applicable						
Test for overall effect: Z = 0.98 (P = 0.33)						
**1.17.2 SQ-LNS**						
Dewey 2017 (2)	0.034	0.1036	810	419	39.5%	1.03 [0.84, 1.27]
Dewey 2017 (3)	-0.1841	0.1303	908	419	36.2%	0.83 [0.64, 1.07]
Hurley 2021 (4)	0.5518	0.2326	176	191	24.3%	1.74 [1.10, 2.74]
**Subtotal (95% CI)**			**1894**	**1029**	**100.0%**	**1.08 [0.78, 1.50]**
Heterogeneity: Tau² = 0.06; Chi² = 7.69, df = 2 (P = 0.02); I² = 74%						
Test for overall effect: Z = 0.49 (P = 0.63)						

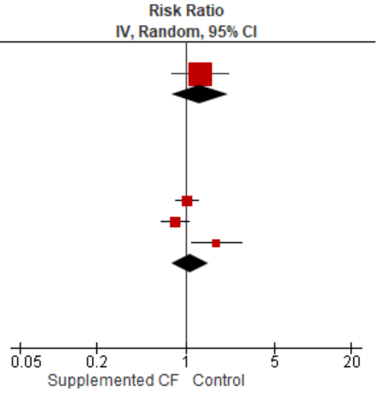

Test for subgroup differences: Chi² = 0.33, df = 1 (P = 0.56), I² = 0%

Footnotes

(1) Eggs; RCT; Ecuador; children aged 6-9 months; supplementation for 6 months; endline
(2) SQLNS; cRCT, South-East Asian; 6-24 months age, supplementation for 18 months
(3) SQLNS; cRCT, South-East Asian; 6-24 months age; mother (during pregnancy) and child supplement; measured at 18 months after intervntion
(4) non-RCT, Africa; children aged 6-24 months; supplementation for 12 months, high fever

Diarrhea

Study or Subgroup	log[Risk Ratio]	SE	Child complementary feeding Total	Control Total	Weight	Risk Ratio IV, Random, 95% CI
**1.18.1 Alternative food**						
Iannoti 2017 (1)	0.7106	0.3277	75	73	100.0%	2.04 [1.07, 3.87]
**Subtotal (95% CI)**			**75**	**73**	**100.0%**	**2.04 [1.07, 3.87]**
Heterogeneity: Not applicable						
Test for overall effect: Z = 2.17 (P = 0.03)						
**1.18.2 Fortified Blended Food**						
Chipili 2022 (2)	-0.2848	0.2508	118	117	100.0%	0.75 [0.46, 1.23]
**Subtotal (95% CI)**			**118**	**117**	**100.0%**	**0.75 [0.46, 1.23]**
Heterogeneity: Not applicable						
Test for overall effect: Z = 1.14 (P = 0.26)						
**1.18.3 SQ LNS**						
Dewey 2017 (3)	0.5539	0.3381	810	419	12.7%	1.74 [0.90, 3.38]
Dewey 2017 (4)	0.4663	0.3372	908	419	12.8%	1.59 [0.82, 3.09]
Humphrey 2019 (5)	-0.2469	0.2511	353	350	20.9%	0.78 [0.48, 1.28]
Hurley 2021 (6)	0.0958	0.1306	176	191	49.2%	1.10 [0.85, 1.42]
Prendergast 2019 (7)	-0.0171	0.6048	59	58	4.4%	0.98 [0.30, 3.22]
**Subtotal (95% CI)**			**2306**	**1437**	**100.0%**	**1.13 [0.88, 1.46]**
Heterogeneity: Tau² = 0.02; Chi² = 4.92, df = 4 (P = 0.30); I² = 19%						
Test for overall effect: Z = 0.97 (P = 0.33)						

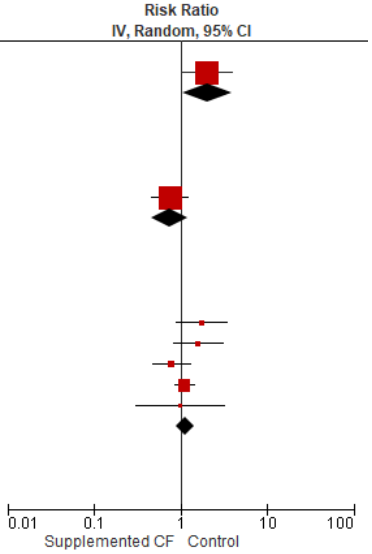

Test for subgroup differences: Chi² = 5.85, df = 2 (P = 0.05), I² = 65.8%

Footnotes

(1) Eggs; RCT; Ecuador; children aged 6-9 months; supplementation for 6 months; acute diarrhea at endline
(2) Fish powder; RCT; Africa; children aged 6-7 months; supplementation for 6 months, endline
(3) SQLNS; cRCT, South-East Asian; 6-24 months age, child supplementation for 18 months
(4) SQLNS; cRCT, South-East Asian; 6-24 months age, Diarhhoea was measured at 18 months intervention to mother (during pregnancy) and child
(5) SQLNS 20 gm-HIV negative- cRCT, Africa, Aged 6-18 month, supplementation for 12 months
(6) non-RCT, Africa; children aged 6-24 months; supplementation for 12 months
(7) SQLNS-20 gm-HIV positive- cRCT, Africa, Aged 6-18 month, supplementation for 12 months

Mean diarrhea episodes

Study or Subgroup	Mean Difference	SE	Child complementary feeding Total	Control Total	Weight	Mean Difference IV, Random, 95% CI
**1.19.1 Alternative food**						
Bhandari 2001 (1)	0.4	0.5188	74	83	100.0%	0.40 [-0.62, 1.42]
**Subtotal (95% CI)**			**74**	**83**	**100.0%**	**0.40 [-0.62, 1.42]**
Heterogeneity: Not applicable						
Test for overall effect: Z = 0.77 (P = 0.44)						
**1.19.2 SQ LNS**						
Dewey 2017 (2)	0.05	0.001	810	419	50.0%	0.05 [0.05, 0.05]
Dewey 2017 (3)	0.04	0.001	908	419	50.0%	0.04 [0.04, 0.04]
**Subtotal (95% CI)**			**1718**	**838**	**100.0%**	**0.05 [0.04, 0.05]**
Heterogeneity: Tau² = 0.00; Chi² = 50.00, df = 1 (P < 0.00001); I² = 98%						
Test for overall effect: Z = 9.00 (P < 0.00001)						

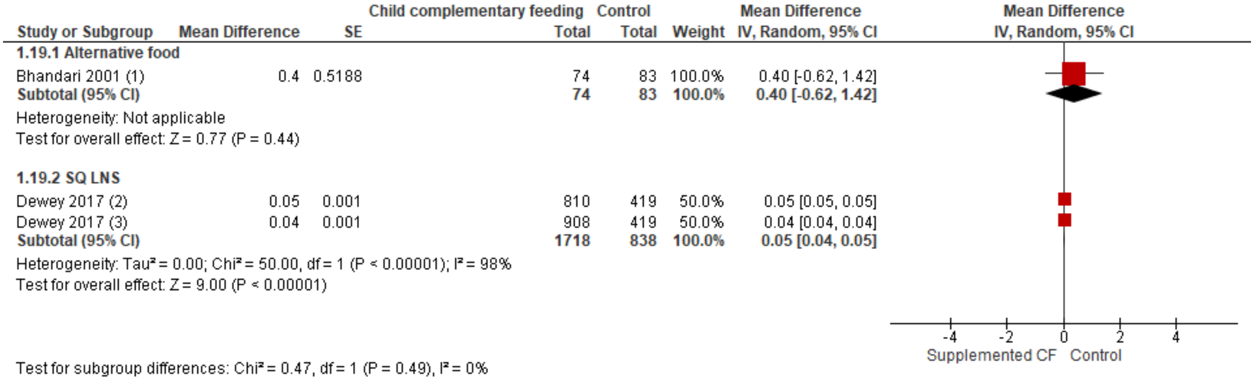

Test for subgroup differences: Chi² = 0.47, df = 1 (P = 0.49), I² = 0%

Footnotes

(1) RCT, South-East Asian Region, aged 4 and 12 months; supplementation for 6 months
(2) SQ LNS; cRCT, South-East Asia; 6-24 months age, child supplementation for 18 months
(3) SQLNS; cRCT, South-East Asian; 6-24 months age, intervention to mother (during pregnancy) and child

Respiratory illness

Study or Subgroup	log[Risk Ratio]	SE	Child complementary feeding Total	Control Total	Weight	Risk Ratio IV, Random, 95% CI
**1.20.1 Alternative food**						
Iannoti 2017 (1)	0.1622	0.2216	25	24	50.5%	1.18 [0.76, 1.82]
Iannoti 2017 (2)	0.5166	0.2465	25	24	40.8%	1.68 [1.03, 2.72]
Iannoti 2017 (3)	-0.1812	0.5315	25	25	8.8%	0.83 [0.29, 2.36]
**Subtotal (95% CI)**			**75**	**73**	**100.0%**	**1.32 [0.97, 1.80]**
Heterogeneity: $Tau^2 = 0.00; Chi^2 = 1.96, df = 2 (P = 0.38); I^2 = 0\%$						
Test for overall effect: Z = 1.76 (P = 0.08)						
**1.20.2 Fortified Blended Food**						
Chipili 2022 (4)	-0.7017	0.4129	118	117	100.0%	0.50 [0.22, 1.11]
**Subtotal (95% CI)**			**118**	**117**	**100.0%**	**0.50 [0.22, 1.11]**
Heterogeneity: Not applicable						
Test for overall effect: Z = 1.70 (P = 0.09)						
**1.20.4 SQ LNS**						
Humphrey 2019 (5)	-0.0101	0.998	352	350	10.9%	0.99 [0.14, 7.00]
Hurley 2021 (6)	0.5926	0.3499	176	191	89.1%	1.81 [0.91, 3.59]
Prendergast 2019 (7)	0	0	59	58		Not estimable
**Subtotal (95% CI)**			**587**	**599**	**100.0%**	**1.69 [0.89, 3.23]**
Heterogeneity: $Tau^2 = 0.00; Chi^2 = 0.32, df = 1 (P = 0.57); I^2 = 0\%$						
Test for overall effect: Z = 1.59 (P = 0.11)						

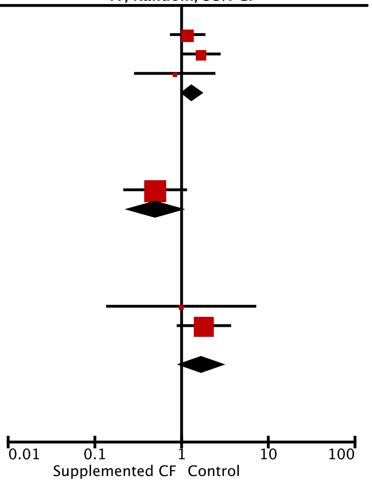

Test for subgroup differences: $Chi^2 = 5.97, df = 2 (P = 0.05), I^2 = 66.5\%$

Footnotes
(1) Eggs; RCT; Ecuador; children aged 6-9 months; supplementation for 6 months; cough
(2) Eggs; RCT; Ecuador; children aged 6-9 months; supplementation for 6 months; congestion
(3) Eggs; RCT; Ecuador; children aged 6-9 months; supplementation for 6 months; difficulty breathing
(4) Fish powder; RCT; Africa; children aged 6-7 months; supplementation for 6 months; cough, endline
(5) SQLNS 20 gm-HIV negative- cRCT, Africa, Aged 6-18 month, supplementation for 12 months
(6) non-RCT, Africa; children aged 6-24 months; supplementation for 12 months, ARI
(7) SQLNS-20 gm-HIV positive- cRCT, Africa, Aged 6-18 month, supplementation for 12 months

URTI

Study or Subgroup	log[Risk Ratio]	SE	Supplemented CF Total	Control Total	Weight	Risk Ratio IV, Random, 95% CI
**1.26.1 SQ-LNS**						
Dewey 2017 (1)	-0.1646	0.0906	810	419	48.3%	0.85 [0.71, 1.01]
Dewey 2017 (2)	-0.1177	0.0876	908	419	51.7%	0.89 [0.75, 1.06]
**Subtotal (95% CI)**			**1718**	**838**	**100.0%**	**0.87 [0.77, 0.98]**
Heterogeneity: $Tau^2 = 0.00; Chi^2 = 0.14, df = 1 (P = 0.71); I^2 = 0\%$						
Test for overall effect: Z = 2.23 (P = 0.03)						
**Total (95% CI)**			**1718**	**838**	**100.0%**	**0.87 [0.77, 0.98]**
Heterogeneity: $Tau^2 = 0.00; Chi^2 = 0.14, df = 1 (P = 0.71); I^2 = 0\%$						
Test for overall effect: Z = 2.23 (P = 0.03)						
Test for subgroup differences: Not applicable						

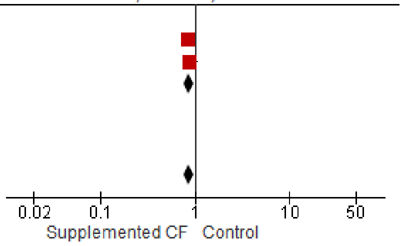

Footnotes
(1) cRCT, South-East Asia; children aged 6-24 months age, child supplementation for 18 months
(2) cRCT, South-East Asia; children aged 6-24 months age, maternal (during pregnancy) and child supplementation for 18 months

LRTI

Study or Subgroup	log[Risk Ratio]	SE	Supplemented CF Total	Control Total	Weight	Risk Ratio IV, Random, 95% CI
**1.27.1 SQ-LNS**						
Dewey 2017 (1)	0.0717	0.1854	810	419	50.6%	1.07 [0.75, 1.55]
Dewey 2017 (2)	-0.0931	0.1875	908	419	49.4%	0.91 [0.63, 1.32]
**Subtotal (95% CI)**			**1718**	**838**	**100.0%**	**0.99 [0.76, 1.28]**
Heterogeneity: $Tau^2 = 0.00; Chi^2 = 0.39, df = 1 (P = 0.53); I^2 = 0\%$						
Test for overall effect: Z = 0.07 (P = 0.94)						
**Total (95% CI)**			**1718**	**838**	**100.0%**	**0.99 [0.76, 1.28]**
Heterogeneity: $Tau^2 = 0.00; Chi^2 = 0.39, df = 1 (P = 0.53); I^2 = 0\%$						
Test for overall effect: Z = 0.07 (P = 0.94)						
Test for subgroup differences: Not applicable						

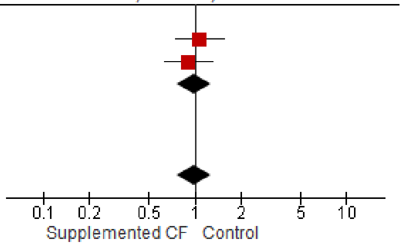

Footnotes
(1) cRCT, South-East Asia; children aged 6-24 months age, child supplementation for 18 months
(2) cRCT, South-East Asia; children aged 6-24 months age,maternal (during pregnancy) and child supplementation for 18 months

Mean respiratory episodes

Study or Subgroup	Mean Difference	SE	Child complementary feeding Total	Control Total	Weight	Mean Difference IV, Random, 95% CI
**1.21.1 Alternative food**						
Bhandari 2001 (1)	-0.09	0.1516	74	83	100.0%	-0.09 [-0.39, 0.21]
**Subtotal (95% CI)**			**74**	**83**	**100.0%**	**-0.09 [-0.39, 0.21]**
Heterogeneity: Not applicable						
Test for overall effect: Z = 0.59 (P = 0.55)						
**1.21.2 SQ LNS**						
Dewey 2017 (2)	-0.17	0.001	908	419	50.0%	-0.17 [-0.17, -0.17]
Dewey 2017 (3)	-0.01	0.001	810	419	50.0%	-0.01 [-0.01, -0.01]
**Subtotal (95% CI)**			**1718**	**838**	**100.0%**	**-0.09 [-0.25, 0.07]**
Heterogeneity: Tau² = 0.01; Chi² = 12800.00, df = 1 (P < 0.00001); I² = 100%						
Test for overall effect: Z = 1.13 (P = 0.26)						

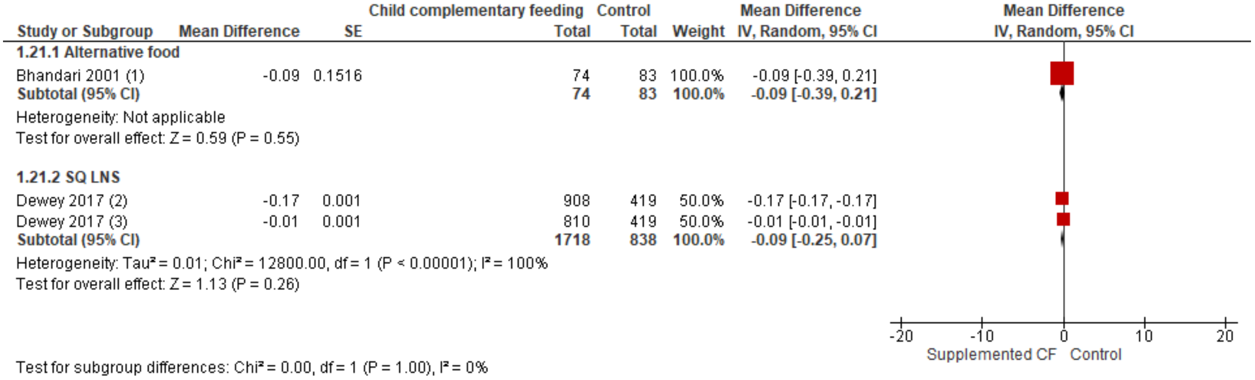

Test for subgroup differences: Chi² = 0.00, df = 1 (P = 1.00), I² = 0%

Footnotes
(1) RCT, South-East Asian Region, aged 4 and 12 months; supplementation for 6 months
(2) SQLNS; cRCT, South-East Asian; 6-24 months age, intervention to mother (during pregnancy) and child
(3) SQ LNS; cRCT, South-East Asia; 6-24 months age, supplementation for 18 months

Skin disease

Study or Subgroup	log[Risk Ratio]	SE	Child complementary feeding Total	Control Total	Weight	Risk Ratio IV, Random, 95% CI
**1.21.1 Alternative food**						
Iannoti 2017 (1)	−0.5577	0.363	37	36	63.6%	0.57 [0.28, 1.17]
Iannoti 2017 (2)	−0.6332	0.4801	38	37	36.4%	0.53 [0.21, 1.36]
**Subtotal (95% CI)**			**75**	**73**	**100.0%**	**0.56 [0.32, 0.98]**
Heterogeneity: Tau² = 0.00; Chi² = 0.02, df = 1 (P = 0.90); I² = 0%						
Test for overall effect: Z = 2.02 (P = 0.04)						
**1.21.2 Fortified Blended Food**						
Chipili 2022 (3)	−1.6179	1.5437	118	117	100.0%	0.20 [0.01, 4.09]
**Subtotal (95% CI)**			**118**	**117**	**100.0%**	**0.20 [0.01, 4.09]**
Heterogeneity: Not applicable						
Test for overall effect: Z = 1.05 (P = 0.29)						

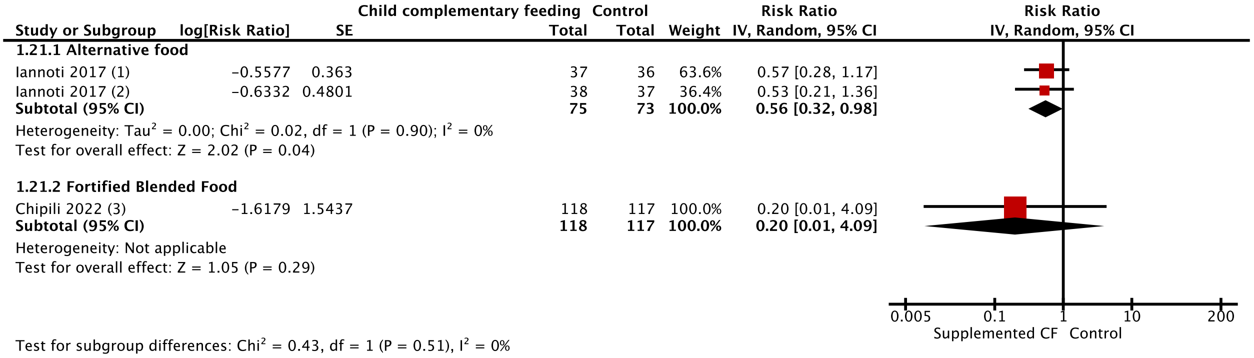

Test for subgroup differences: Chi² = 0.43, df = 1 (P = 0.51), I² = 0%

Footnotes
(1) Eggs; RCT; Ecuador; children aged 6–9 months; supplementation for 6 months; Rashes
(2) Eggs; RCT; Ecuador; children aged 6–9 months; supplementation for 6 months; bruises/scrpas/cuts
(3) Fish powder; RCT; Africa; children aged 6–7 months; supplementation for 6 months; Rashes, endline

Vomiting

Study or Subgroup	log[Risk Ratio]	SE	Child complementary feeding Total	Control Total	Weight	Risk Ratio IV, Random, 95% CI
**1.23.1 Fortified Blended Food**						
Chipili 2022 (1)	-0.0085	0.3868	118	117	100.0%	0.99 [0.46, 2.12]
**Subtotal (95% CI)**			**118**	**117**	**100.0%**	**0.99 [0.46, 2.12]**
Heterogeneity: Not applicable						
Test for overall effect: Z = 0.02 (P = 0.98)						

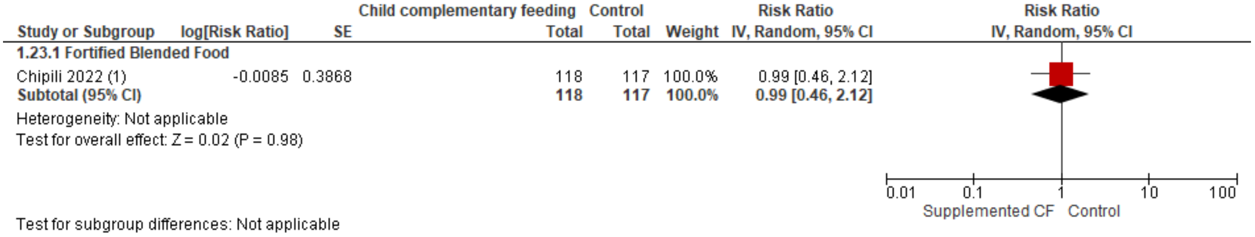

Test for subgroup differences: Not applicable

Footnotes
(1) Fish powder; RCT; Africa; children aged 6-7 months; supplementation for 6 months

Any SAE

Study or Subgroup	log[Risk Ratio]	SE	Child complementary feeding Total	Control Total	Weight	Risk Ratio IV, Random, 95% CI
**1.24.1 SQLNS**						
Adu-Afarwuah 2016 (1)	-0.0211	0.1453	409	411	35.7%	0.98 [0.74, 1.30]
Ashorn 2015 (2)	-0.015	0.113	281	288	59.0%	0.99 [0.79, 1.23]
Maleta 2015 (3)	-0.2877	0.5814	92	16	2.2%	0.75 [0.24, 2.34]
Maleta 2015 (4)	-0.539	0.6346	68	17	1.9%	0.58 [0.17, 2.02]
Maleta 2015 (5)	-0.0572	0.8145	36	17	1.1%	0.94 [0.19, 4.66]
**Subtotal (95% CI)**			**886**	**749**	**100.0%**	**0.97 [0.82, 1.15]**

Heterogeneity: $Tau^2 = 0.00$; $Chi^2 = 0.86$, $df = 4$ ($P = 0.93$); $I^2 = 0\%$
Test for overall effect: $Z = 0.39$ ($P = 0.70$)

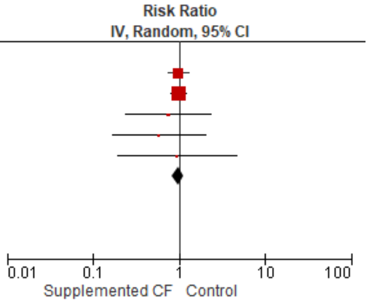

Test for subgroup differences: Not applicable

Footnotes
(1) SQ-LNS; RCT; African region; mothers with >20 wk of gestation; supplementation for 6 months
(2) RCT; Malawi; children aged 6-18 months; supplementaion for 6 months
(3) 40g milk; RCT, Africa; aged 6 months, supplementation for 12 months; death during 6-12 months of age
(4) 20g milk; RCT, Africa; aged 6 months, supplementation for 12 months; death during 6-12 months of age
(5) 10g milk; RCT, Africa; aged 6 months, supplementation for 12 months; death during 6-12 months of age

Death

Study or Subgroup	log[Risk Ratio]	SE	Child complementary feeding Total	Control Total	Weight	Risk Ratio IV, Random, 95% CI
**1.24.1 Alternative food**						
Bauserman 2015 (1)	-0.6931	1.1748	48	48	37.6%	0.50 [0.05, 5.00]
Bhandari 2001 (2)	-0.6741	1.2169	104	106	35.8%	0.51 [0.05, 5.53]
Stewart 2019 (3)	2.1917	1.4927	331	329	26.6%	8.95 [0.48, 166.89]
**Subtotal (95% CI)**			**483**	**483**	**100.0%**	**1.08 [0.19, 6.06]**
Heterogeneity: Tau^2 = 0.67; Chi^2 = 2.81, df = 2 (P = 0.25); I^2 = 29%						
Test for overall effect: Z = 0.09 (P = 0.93)						
**1.24.2 Fortified Blended Food**						
Christian 2015 (4)	0.0555	0.5676	567	959	34.7%	1.06 [0.35, 3.22]
Ghosh 2020 (5)	0.392	0.9073	194	192	13.6%	1.48 [0.25, 8.76]
Langendorf 2014 (6)	-0.9676	0.6811	1338	1189	24.1%	0.38 [0.10, 1.44]
Mangani 2015 (7)	-0.3655	0.6377	196	204	27.5%	0.69 [0.20, 2.42]
**Subtotal (95% CI)**			**2295**	**2544**	**100.0%**	**0.77 [0.40, 1.48]**
Heterogeneity: Tau^2 = 0.00; Chi^2 = 1.93, df = 3 (P = 0.59); I^2 = 0%						
Test for overall effect: Z = 0.78 (P = 0.43)						
**1.24.3 Commercially Produced Ready to Use Supplementary Food**						
Christian 2015 (8)	-0.4413	0.7042	995	959	30.9%	0.64 [0.16, 2.56]
Huybregts 2012 (9)	0.793	1.1544	386	284	11.7%	2.21 [0.23, 21.23]
Isanaka 2010 (10)	-0.9943	1.135	309	345	12.1%	0.37 [0.04, 3.42]
Langendorf 2014 (11)	-1.1087	0.7911	1353	594	24.6%	0.33 [0.07, 1.56]
Langendorf 2014 (12)	-1.9346	0.8648	2059	595	20.7%	0.14 [0.03, 0.79]
**Subtotal (95% CI)**			**5102**	**2777**	**100.0%**	**0.43 [0.20, 0.94]**
Heterogeneity: Tau^2 = 0.01; Chi^2 = 4.06, df = 4 (P = 0.40); I^2 = 1%						
Test for overall effect: Z = 2.11 (P = 0.03)						
**1.24.4 Locally Produced Ready to Use Supplementary Food**						
Christian 2015 (13)	-1.6607	1.1486	550	479	18.5%	0.19 [0.02, 1.80]
Christian 2015 (14)	0.131	0.8631	562	480	32.8%	1.14 [0.21, 6.19]
Mangani 2015 (15)	-1.0498	0.8998	196	102	30.2%	0.35 [0.06, 2.04]
Mangani 2015 (16)	-1.8207	1.1484	210	102	18.5%	0.16 [0.02, 1.54]
**Subtotal (95% CI)**			**1518**	**1163**	**100.0%**	**0.40 [0.15, 1.05]**
Heterogeneity: Tau^2 = 0.00; Chi^2 = 2.53, df = 3 (P = 0.47); I^2 = 0%						
Test for overall effect: Z = 1.86 (P = 0.06)						
**1.24.5 SQLNS**						
Adu-Afarwuah 2016 (17)	0.5365	0.4704	409	408	5.4%	1.71 [0.68, 4.30]
Ashorn 2015 (18)	-0.6172	0.3816	281	288	6.3%	0.54 [0.26, 1.14]
Becquey 2019 (19)	0.2259	0.3461	809	845	6.6%	1.25 [0.64, 2.47]
Dewey 2017 (20)	-0.1994	0.2673	847	449	7.4%	0.82 [0.49, 1.38]
Dewey 2017 (21)	-0.2578	0.2593	1047	445	7.4%	0.77 [0.46, 1.28]
Galasso 2019 (22)	0	0.621	139	139	4.2%	1.00 [0.30, 3.38]
Hess 2015 (23)	-0.8746	0.319	1623	523	6.9%	0.42 [0.22, 0.78]
Humphrey 2019 (24)	1.612	0.2341	383	384	7.7%	5.01 [3.17, 7.93]
Huybregts 2019 (25)	-0.2763	0.7486	87	88	3.4%	0.76 [0.17, 3.29]
Iannotti 2014 (26)	0.3498	1.6283	202	95	1.0%	1.42 [0.06, 34.51]
Iannotti 2014 (27)	1.0729	1.6298	196	96	1.0%	2.92 [0.12, 71.33]
Luby 2018 (28)	-0.2062	0.2418	528	1046	7.6%	0.81 [0.51, 1.31]
Maleta 2015 (29)	-0.4155	0.6353	322	64	4.1%	0.66 [0.19, 2.29]
Maleta 2015 (30)	0.1699	0.6084	324	64	4.3%	1.19 [0.36, 3.91]
Maleta 2015 (31)	-0.1462	0.6257	321	64	4.2%	0.86 [0.25, 2.95]
Maleta 2015 (32)	-0.6379	0.663	323	64	3.9%	0.53 [0.14, 1.94]
Maleta 2015 (33)	-0.0752	0.6213	322	64	4.2%	0.93 [0.27, 3.13]
Null 2018 (34)	-0.078	0.1833	480	1036	8.1%	0.92 [0.65, 1.32]
Prendergast 2019 (35)	-0.8538	0.8178	62	66	3.1%	0.43 [0.09, 2.12]
Smuts 2019 (36)	0	1.2198	250	125	1.7%	1.00 [0.09, 10.92]
Smuts 2019 (37)	-0.6931	1.41	250	125	1.3%	0.50 [0.03, 7.93]
**Subtotal (95% CI)**			**9205**	**6478**	**100.0%**	**0.94 [0.67, 1.33]**
Heterogeneity: Tau^2 = 0.35; Chi^2 = 65.05, df = 20 (P < 0.00001); I^2 = 69%						
Test for overall effect: Z = 0.35 (P = 0.73)						

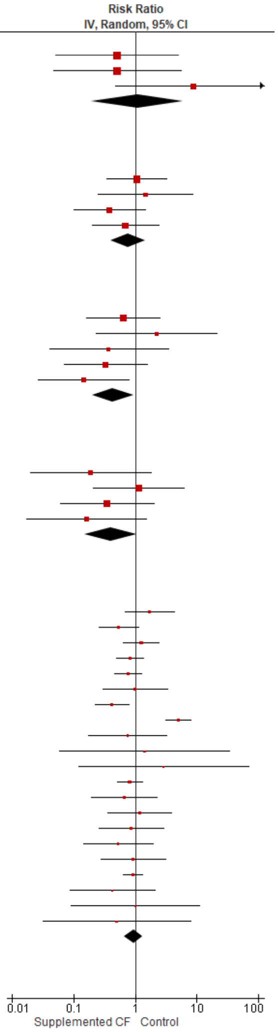

Test for subgroup differences: Chi^2 = 5.31, df = 4 (P = 0.26), I^2 = 24.7%

Footnotes
(1) cRCT, Africa, children aged 5 months; supplementation for18 months
(2) RCT, South-East Asian Region, 4 and 12 mo of age, supplementation for 6 months intervention
(3) RCT, Africa, 6-9.9 months age; supplementation for 6 month, under 5 mortality
(4) cRCT, South-East Asian Region, age-6-18 months; supplementation for 12 months
(5) cRCT, Africa, 6-18 months age, supplementation for intervention 6-12 months
(6) SC+ (820 kcal/day); RCT; Africa; aged 6-23 months, supplementation for 5 months; death at <5 yrs,
(7) CSB- RCT, Africa, age 6-18 months, suplementation for 12 month
(8) Plumpy doz- cRCT, South-East Asian Region, age-6-18 months; supplementation for 12 months
(9) Plumpydoz Nutriset 46 g ~247Kcal/d; cRCT; Africa; age 6-36 months, supplementation for 4 months
(10) cRCT; Africa; RUTF (500 kcal/day); children 6-60 months; under 5 mortality
(11) HQ LNS; cRCT, Africa, 6-23 months age, supplementation for < 6 months, death at <5 yrs
(12) MQ-LNS; cRCT; Africa; aged 6-23 months, supplementation for 5 months; death at <5 yrs,
(13) cRCT; Rice/lentils; South East Asia; aged 6-18 months; supplementation for 12 months
(14) cRCT; chickpeas; South East Asia; aged 6-18 months; supplementation for 12 months
(15) soy LNS- RCT, Africa, age 6-18 months, suplementation for 12 month
(16) milk LNS- RCT, Africa, age 6-18 months, suplementation for 12 month
(17) SQ-LNS; RCT; African region; mothers with >20 wk of gestation; supplementation for 6 months, under 5 mortality
(18) RCT; Malawi; children aged 6-18 months; supplementaion for 6 months; infant death (0-19 months)
(19) SQLNS 20gm; cRCT: Africa; children aged 0-17 months
(20) SQLNS; cRCT, South-East Asian; 6-24 months age, child supplementation for 18 months, most of these deaths occurred at <14 days postpartum
(21) SQLNS; cRCT, South-East Asian; 6-24 months age, intervention to mother and child, most of these deaths occurred at <14 days postpartum
(22) SQLNS ; cRCT; Madasgar; Children <12 years; supplementation for 12 months; infant deaths
(23) zinc; cRCT, Africa, aged 9-18 months of age, supplementation for 9 months; under 5 mortality
(24) SQLNS 20 gm-HIV negative, cRCT, Africa, Aged 6-18 month, supplementation for 12 months; under 5 mortality
(25) cRCT; Africa; age 6-23 months, supplementation for 12 months; under 24 months mortality
(26) SQ-LNS; RCT African region; children aged 6-11 months; supplementation for 6 months
(27) SQ-LNS; RCT African region; children aged 6-11 months; supplementation for 3 months
(28) 20 g SQLNS, cRCT; Bangladesh; 6-24 months age; supplementation for 12 months; under 5 mortality
(29) 20g milk; RCT, Africa; aged 6 months, supplementation for 12 months; death until 18 months of age
(30) 40g no milk; RCT, Africa; aged 6 months, supplementation for 12 months; death until 18 months of age
(31) 10g milk-death during 6-12 months of age; RCT, Africa; aged 6 months; supplementation for 12 months; death until 18 months of age
(32) 20g no milk; RCT, Africa; aged 6 months, supplementation for 12 months; death until 18 months of age
(33) 40g milk; RCT, Africa; aged 6 months, supplementation for 12 months; death until 18 months of age
(34) SQLNS-20 gm, cRCT, Africa; 6-24 month aged, under 5 mortality
(35) SQLNS-20 gm-HIV positive, cRCT, Africa, Aged 6-18 month, supplementation for 12 months, under 5 mortality
(36) SQ LNS plus; RCT, Africa; aged 6-12 months, supplementation for 6 months; infant mortality
(37) SQ LNS; RCT, Africa; aged 6-12 months, supplementation for 6 months; infant mortality

Comparison 2: Complementary food education versus control

LBW

Study or Subgroup	log[Risk Ratio]	SE	IYCF education Total	Control Total	Weight	Risk Ratio IV, Random, 95% CI
Fahmida, 2020 (1)	-0.4898	0.4396	231	230	48.7%	0.61 [0.26, 1.45]
Nyamasege, 2018 (2)	-1.0169	0.4286	282	306	51.3%	0.36 [0.16, 0.84]
**Total (95% CI)**			**513**	**536**	**100.0%**	**0.47 [0.26, 0.85]**

Heterogeneity: $Tau^2 = 0.00$; $Chi^2 = 0.74$, df = 1 (P = 0.39); $I^2 = 0\%$
Test for overall effect: Z = 2.48 (P = 0.01)

Risk Ratio IV, Random, 95% CI

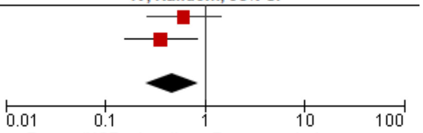

Footnotes
(1) integrated ICF; cRCT; Indonesia; children aged 0-18 months; follow-up 18 months
(2) home-based nutritional counselling; cRCT; African Region; at birth; Kenya

HAZ

Study or Subgroup	Mean Difference	SE	IYCF education Total	Control Total	Weight	Mean Difference IV, Random, 95% CI
Atukunda 2019 (1)	0.11	0.1276	69	69	4.5%	0.11 [−0.14, 0.36]
Fahmida, 2020 (2)	0.09	0.0919	218	214	5.7%	0.09 [−0.09, 0.27]
Hitachi, 2020 (3)	0.04	0.1646	67	113	3.5%	0.04 [−0.28, 0.36]
Kang, 2017 (4)	0.021	0.0066	56	69	7.9%	0.02 [0.01, 0.03]
Martinez, 2018 (5)	0.07	0.0541	145	151	7.0%	0.07 [−0.04, 0.18]
Nair, 2017 (6)	0.07	0.1135	247	246	4.9%	0.07 [−0.15, 0.29]
Navarro 2013 (7)	0.2	0.1103	193	259	5.0%	0.20 [−0.02, 0.42]
Olaya 2013 (8)	0.14	0.1459	38	38	3.9%	0.14 [−0.15, 0.43]
Owais, 2017 (9)	0.2	0.0314	351	341	7.6%	0.20 [0.14, 0.26]
Penny 2005 (10)	0.38	0.1327	75	76	4.3%	0.38 [0.12, 0.64]
Reinbott, 2016 (11)	−0.06	0.1257	175	132	4.5%	−0.06 [−0.31, 0.19]
Roy, 2007 (12)	0.23	0.0761	290	282	6.2%	0.23 [0.08, 0.38]
Roy 2022 (13)	0.61	0.2172	96	96	2.4%	0.61 [0.18, 1.04]
Salehi, 2004 (14)	0.41	0.1126	406	405	4.9%	0.41 [0.19, 0.63]
Sharma, 2020 (15)	0.33	0.0681	190	191	6.5%	0.33 [0.20, 0.46]
Singh, 2017 (16)	0.1	0.0845	402	340	5.9%	0.10 [−0.07, 0.27]
Sinharoy 2017 (17)	0.05	0.0743	408	431	6.3%	0.05 [−0.10, 0.20]
Sudfeld, 2021 (18)	0.79	0.2031	123	116	2.7%	0.79 [0.39, 1.19]
Zaman 2008 (19)	0.123	0.2061	95	95	2.6%	0.12 [−0.28, 0.53]
Zhang, 2013 (20)	0.87	0.1612	78	71	3.5%	0.87 [0.55, 1.19]
**Total (95% CI)**			**3722**	**3735**	**100.0%**	**0.20 [0.12, 0.28]**

Heterogeneity: $Tau^2 = 0.02$; $Chi^2 = 127.86$, df = 19 (P < 0.00001); $I^2 = 85\%$
Test for overall effect: Z = 4.94 (P < 0.00001)

Mean Difference IV, Random, 95% CI

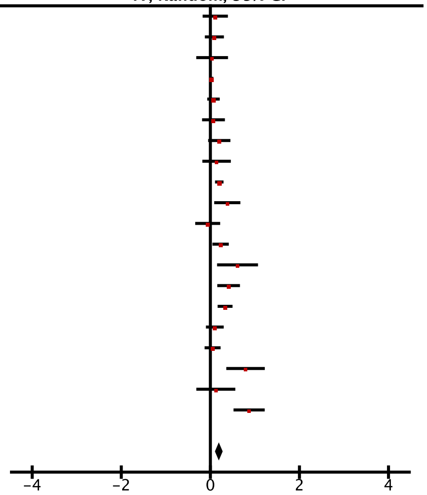

Footnotes
(1) cRCT; African Region; IYCF vs control; Children aged 6–8 months; Follow-up at 20–24 months; HAZ at endline
(2) integrated ICF; cRCT; Indonesia; children aged 0–18 months; follow-up 18 months
(3) Education on maternal and child nutrition and follow-up consultations; non-RCT; African Region; children aged 5–59 months; follow-up 12 months; change in..
(4) cRCT; African Region; IYCF [community-based participatory nutrition promotion (CPNP)'] vs control; children aged 6–12 months; intervention for 12 months
(5) RCT; Regions of America; children aged 6–24 months; Follow-up 6 months; change in HAZ
(6) change in HAZ; cRCT; South East Asia; support feeding, hygiene, care, and stimulation; vs control; children aged 0–18 months; Intervention for 18 months
(7) non-RCT, Regions of America, IYCF vs control; children <2 years; Intervention for 24 months; HAZ at endline
(8) RCT, Region of Americas, children of 6months, change in HAZ
(9) Community-based nutrition education program; RCT; Bangladesh; children aged 0–23 months; at 24 months
(10) cRCT; Peru; children aged 4 months; follow-up at 18 months, endline
(11) IYCG and agricultural activities; cRCT; Western Pacific; children aged 0–23 months; Follow-up at 2 years
(12) RCT; South East Asia; children aged 6–9 months; Follow-up 12 months; after intervention
(13) RCT, Bangladesh, Asia, 6–23 months, IYCF
(14) HAZ; RCT; EMRO; environmental health, personal hygiene and nutritional edu vs control; children aged 0–59 months; Intervention for 12 months
(15) HAZ; non-RCT; South East Asia; nutritional education arm vs control; children aged 4–6 months; Intervention for 6 months
(16) Integrated Nutrition and Health Program (INHP II); qRCT; South East Asia; children aged 6 months to 6 years; endline at 18 momths
(17) HAZ; cRCT; Western Rwanda; Classic intervention intervention vs control; children <5 years; Intervention for 2 years
(18) HAZ; cRCT; African Region;integrated health nutrition and responsive stimulation vs control; children <1 years; Intervention for 18 months
(19) cRCT; Pakistan; 6–18 months old children; duration of 180 days
(20) cRCT; Western Pacific Region; children at 6 months; at 18 months

Stunting

Study or Subgroup	log[Risk Ratio]	SE	IYCF education Total	Control Total	Weight	Risk Ratio IV, Random, 95% CI
Abiyu, 2020 (1)	−0.3857	0.1939	136	141	2.3%	0.68 [0.46, 0.99]
Ali 2022 (2)	−0.1173	0.3356	46	90	0.9%	0.89 [0.46, 1.72]
Atukunda 2019 (3)	−0.3562	0.173	70	71	2.7%	0.70 [0.50, 0.98]
Bhandari, 2001 (4)	−0.1491	0.0978	97	93	5.3%	0.86 [0.71, 1.04]
Bhandari, 2003 (5)	−0.0196	0.1038	218	197	5.0%	0.98 [0.80, 1.20]
Fahmida, 2020 (6)	−0.1105	0.1174	218	214	4.4%	0.90 [0.71, 1.13]
Gope, 2019 (7)	−0.0026	0.039	1213	1045	8.4%	1.00 [0.92, 1.08]
Guldan 2000 (8)	−0.9559	0.5233	220	203	0.4%	0.38 [0.14, 1.07]
Kabahenda, 2014 (9)	−0.3361	0.3454	45	38	0.9%	0.71 [0.36, 1.41]
Kang, 2017 (10)	0.0887	0.3452	56	69	0.9%	1.09 [0.56, 2.15]
Liang 2018 (11)	−0.3175	0.0548	2693	8425	7.6%	0.73 [0.65, 0.81]
More, 2017 (12)	0.028	0.0461	1089	1098	8.0%	1.03 [0.94, 1.13]
Nair, 2017 (13)	−0.0294	0.0572	300	312	7.5%	0.97 [0.87, 1.09]
Navarro 2013 (14)	−0.5008	0.3076	193	259	1.1%	0.61 [0.33, 1.11]
Penny 2005 (15)	−1.2338	0.6352	68	66	0.3%	0.29 [0.08, 1.01]
Saaka, 2021 (16)	0.016	0.1304	403	273	4.0%	1.02 [0.79, 1.31]
Saleem, 2014 (17)	−0.1203	0.0723	95	75	6.6%	0.89 [0.77, 1.02]
Sharma, 2020 (18)	−0.6009	0.4971	190	191	0.4%	0.55 [0.21, 1.45]
Singh, 2017 (19)	−0.0416	0.0471	402	340	8.0%	0.96 [0.87, 1.05]
Sinharoy 2017 (20)	−0.0399	0.0525	997	1038	7.7%	0.96 [0.87, 1.07]
Sudfeld, 2021 (21)	−0.6239	0.2144	123	116	2.0%	0.54 [0.35, 0.82]
Sustanto, 2019 (22)	0.5306	0.3382	45	45	0.9%	1.70 [0.88, 3.30]
Walsh 2002 (23)	0.2441	0.1657	738	78	2.9%	1.28 [0.92, 1.77]
Yadav, 2014 (24)	−0.1497	0.1034	564	759	5.0%	0.86 [0.70, 1.05]
Young 2021 (25)	−0.021	0.1409	112	58	3.6%	0.98 [0.74, 1.29]
Young 2021 (26)	−0.3087	0.1621	112	58	3.0%	0.73 [0.53, 1.01]
**Total (95% CI)**			**10443**	**15352**	**100.0%**	**0.90 [0.84, 0.96]**

Heterogeneity: $Tau^2 = 0.01; Chi^2 = 62.85, df = 25 (P < 0.0001); I^2 = 60\%$
Test for overall effect: Z = 3.15 (P = 0.002)

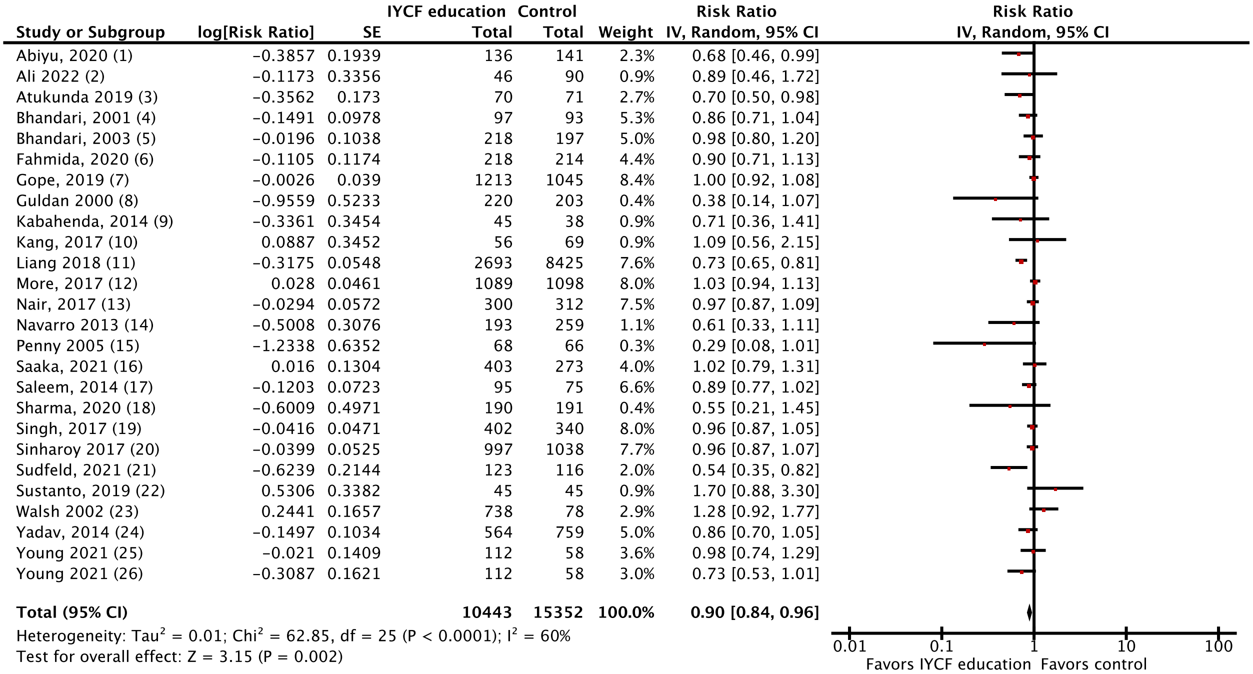

Footnotes
(1) cRCT; African Region; IYCF vs control; children aged 12-24 months; Intervention for 9 months; Prevalence of stunting at 9 months; study reported cluster...
(2) RCT, Somalia, Africa, nutrition couselling, IDP children
(3) cRCT; African Region; IYCF vs control; Children aged 6-8 months; Follow-up at 20-24 months; prevalence of stunting
(4) RCT; South East Asia; nutritional counselling vs control; children aged 4-12 months; intervention for 12 months; prevalence of stunting
(5) cRCT; South East Asia; children aged 6-18 months; Intervention for 6 months; prevalence of stunting between 12-18 months
(6) integrated ICF; cRCT; Indonesia; children aged 0-18 months; follow-up 18 months
(7) non-RCT; South-East Asia; PLA vs control; children aged 6 months to 3 years; Intervention for 24 months; prevalence of stunting
(8) non-RCT; China; 4-12 months; intervention for 1 year
(9) non-RCT; African Region; Structured nutrition education programme vs control; children aged 6-48 months; Intervention for 9 months; incidence of stunting
(10) cRCT; African Region; IYCF [community-based participatory nutrition promotion (CPNP)] vs control; children aged 6-12 months; intervention for 12 months;...
(11) non-RCT; Western Pacific region; IYCF vs control; children <3 years; Intervention for 4 years; prevalence of stunting
(12) cRCT; South East Asia; integrated activities vs control; children aged 5 years; prevalence of stunting (Endline HAZ<-2)
(13) cRCT; South East Asia; support feeding, hygiene, care, and stimulation; vs control; children aged 0-18 months; Intervention for 18 months; prevalence of...
(14) non-RCT, Regions of America, IYCF vs control; children <2 years; Intervention for 24 months; prevalence of stunting
(15) cRCT; Peru; children aged 4 months; follow-up at 18 months, endline
(16) non-RCT; African Region; IYCF vs control; children aged 6-36 months; Intervention for 12 months Prevalence of stunting
(17) cRCT; EMRO; IYCF vs control; children newborn; Intervention for 10 weeks; stunting at endline
(18) non-RCT; South East Asia; nutritional education arm vs control; children aged 4-6 months; Intervention for 6 months; prevalence of stunting
(19) Integrated Nutrition and Health Program (INHP II); qRCT; South East Asia; children aged 6 months to 6 years; endline at 18 momths
(20) cRCT; Western Rwanda; Classic intervention intervention vs control; children <5 years; Intervention for 2 years; stunting
(21) cRCT; African Region; integrated health nutrition and responsive stimulation vs control; children <1 years; Intervention for 18 months; stunting
(22) cRCT; South East Asia; growth, nutrition and development intervention vs control; children aged 0-72 months; Intervention for 12 weeks; stunting
(23) non-RCT; rural Africa; EBF vs. control for 6 months; intervention provided to mothers; stunting (HAZ <-3 to <-2) at 2 years
(24) non-RCT; South East Asia; IYCF vs control; children aged <3 years; intervention for 12 months; prevalence of stunting
(25) cRCT; Cambodia, Western Pacific; traditional Positive Deviance/Hearth with Interactive Voice Calling (PDH-IVC);prevalence at 3 months followup
(26) cRCT; Cambodia, Western Pacific; traditional Positive Deviance/Hearth programme (PDH); prevalence at 3 months followup

Severe Stunting

Study or Subgroup	log[Risk Ratio]	SE	Weight	Risk Ratio IV, Random, 95% CI
Walsh 2002 (1)	0.032	0.2657	100.0%	1.03 [0.61, 1.74]
**Total (95% CI)**			**100.0%**	**1.03 [0.61, 1.74]**

Heterogeneity: Not applicable
Test for overall effect: Z = 0.12 (P = 0.90)

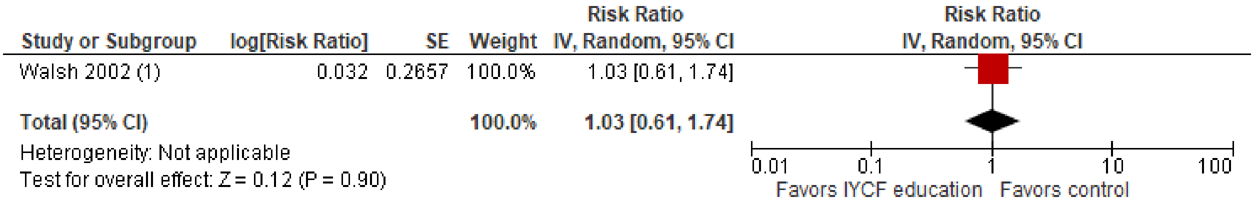

Footnotes
(1) non-RCT; rural Africa; EBF vs. control for 6 months; intervention provided to mothers; severe stunting (HAZ <-3) at 2 years

WHZ

Study or Subgroup	Mean Difference	SE	IYCF education Total	Control Total	Weight	Mean Difference IV, Random, 95% CI
Atukunda 2019 (1)	−0.19	0.1684	69	69	3.8%	−0.19 [−0.52, 0.14]
Hitachi, 2020 (2)	−0.14	0.0237	67	113	10.8%	−0.14 [−0.19, −0.09]
Kang, 2017 (3)	0.041	0.0085	56	69	11.2%	0.04 [0.02, 0.06]
Martinez, 2018 (4)	0.08	0.0808	145	151	7.7%	0.08 [−0.08, 0.24]
Nair, 2017 (5)	0	0.0841	300	312	7.5%	0.00 [−0.16, 0.16]
Olaya 2013 (6)	0.1	0.1633	38	38	3.9%	0.10 [−0.22, 0.42]
Penny 2005 (7)	0.1	0.1353	75	76	4.9%	0.10 [−0.17, 0.37]
Reinbott, 2016 (8)	0	0.1135	175	132	5.9%	0.00 [−0.22, 0.22]
Roy, 2007 (9)	0.16	0.0832	290	282	7.6%	0.16 [−0.00, 0.32]
Roy 2022 (10)	0.64	0.1826	96	96	3.4%	0.64 [0.28, 1.00]
Salehi, 2004 (11)	0.266	0.1196	406	405	5.6%	0.27 [0.03, 0.50]
Sharma, 2020 (12)	0.23	0.076	190	191	8.0%	0.23 [0.08, 0.38]
Sinharoy 2017 (13)	0.018	0.0645	409	432	8.7%	0.02 [−0.11, 0.14]
Sudfeld, 2021 (14)	−0.16	0.1838	123	116	3.4%	−0.16 [−0.52, 0.20]
Zaman 2008 (15)	0.508	0.172	95	95	3.7%	0.51 [0.17, 0.85]
Zhang, 2013 (16)	0.3	0.1671	78	71	3.8%	0.30 [−0.03, 0.63]
**Total (95% CI)**			**2612**	**2648**	**100.0%**	**0.09 [0.01, 0.17]**

Heterogeneity: Tau^2 = 0.01; Chi^2 = 91.60, df = 15 (P < 0.00001); I^2 = 84%
Test for overall effect: Z = 2.26 (P = 0.02)

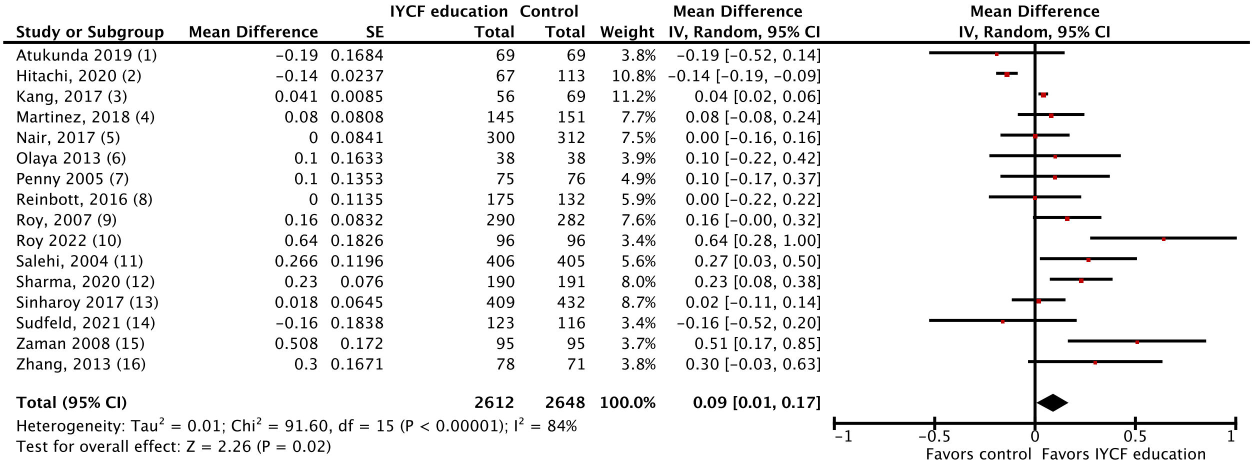

Footnotes
(1) cRCT; African Region; IYCF vs control; Children aged 6–8 months; Follow-up at 20–24 months; WHZ at endline
(2) Education on maternal and child nutrition and follow-up consultations; non-RCT; African Region; children aged 5–59 months; follow-up 12 months; change in...
(3) change in WHZ; cRCT; African Region; IYCF [community-based participatory nutrition promotion (CPNP)'] vs control; children aged 6–12 months; intervention fo
(4) RCT; Regions of America; children aged 6–24 months; Follow-up 6 months; change in WHZ
(5) cRCT; South East Asia; support feeding, hygiene, care, and stimulation; vs control; children aged 0–18 months; Intervention for 18 months; adjusted WHZ at 18...
(6) RCT, Region of Americas, children of 6months, change in WHZ
(7) cRCT; Peru; children aged 4 months; follow-up at 18 months, endline
(8) IYCG and agricultural activities; cRCT; Western Pacific; children aged 0–23 months; Follow-up at 2 years
(9) RCT; South East Asia; children aged 6–9 months; Follow-up 12 months; after intervention
(10) RCT, Bangladesh, Asia, 6–23 months, IYCF
(11) WHZ; RCT; EMRO; environmental health, personal hygiene and nutritional edu vs control; children aged 0–59 months; Intervention for 12 months
(12) WHZ; non-RCT; South East Asia; nutritional education arm vs control; children aged 4–6 months; Intervention for 6 months
(13) WHZ; cRCT; Western Rwanda; Classic intervention intervention vs control; children <5 years; Intervention for 2 years
(14) WHZ; cRCT; African Region; integrated health nutrition and responsive stimulation vs control; children <1 years; Intervention for 18 months
(15) cRCT; Pakistan; 6–18 months old children; duration of 180 days
(16) kgs; cRCT; Western Pacific Region; children at 6 months; at 18 months

Incidence of Wasting

Study or Subgroup	log[Risk Ratio]	SE	Weight	Risk Ratio IV, Random, 95% CI
Kabahenda, 2014 (1)	0.9335	1.6184	100.0%	2.54 [0.11, 60.67]
**Total (95% CI)**			**100.0%**	**2.54 [0.11, 60.67]**

Heterogeneity: Not applicable
Test for overall effect: Z = 0.58 (P = 0.56)

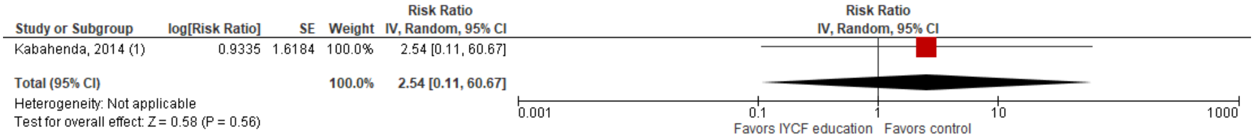

Footnotes
(1) non-RCT; African Region; Structured nutrition education programme vs control; children aged 6-48 months; Intervention for 9 months; Incidence of wasting (Endline WHZ<-2) at 9 months

Prevalence of Wasting

Study or Subgroup	log[Risk Ratio]	SE	IYCF education Total	Control Total	Weight	Risk Ratio IV, Random, 95% CI
Abiyu, 2020 (1)	−0.118	0.3746	136	141	3.1%	0.89 [0.43, 1.85]
Ali 2022 (2)	−0.1555	0.4153	46	90	2.6%	0.86 [0.38, 1.93]
Atukunda 2019 (3)	0.4196	0.8972	70	71	0.7%	1.52 [0.26, 8.83]
Bhandari, 2001 (4)	−0.2653	0.3591	97	93	3.3%	0.77 [0.38, 1.55]
Kadiyala, 2021 (5)	−0.064	0.173	421	438	7.7%	0.94 [0.67, 1.32]
Kadiyala, 2021 (6)	0.0625	0.1717	453	421	7.8%	1.06 [0.76, 1.49]
Kang, 2017 (7)	−0.0826	0.2844	48	68	4.5%	0.92 [0.53, 1.61]
Liang 2018 (8)	−0.2722	0.1196	2693	8425	9.8%	0.76 [0.60, 0.96]
More, 2017 (9)	−0.0827	0.1156	1088	1097	10.0%	0.92 [0.73, 1.15]
Nair, 2017 (10)	−0.0492	0.0978	300	312	10.7%	0.95 [0.79, 1.15]
Navarro 2013 (11)	0.5818	0.7578	193	259	0.9%	1.79 [0.41, 7.90]
Saaka, 2021 (12)	0.7806	0.2582	403	273	5.1%	2.18 [1.32, 3.62]
Saleem, 2014 (13)	−0.9628	1.2161	95	75	0.4%	0.38 [0.04, 4.14]
Sharma, 2020 (14)	−0.7569	0.307	190	191	4.1%	0.47 [0.26, 0.86]
Sinharoy 2017 (15)	0.3188	0.3288	979	1026	3.7%	1.38 [0.72, 2.62]
Sudfeld, 2021 (16)	−0.3463	0.7527	123	116	0.9%	0.71 [0.16, 3.09]
Sustanto, 2019 (17)	−0.6931	0.84	45	45	0.8%	0.50 [0.10, 2.59]
Teshome, 2020 (18)	−0.9043	0.2245	192	196	6.0%	0.40 [0.26, 0.63]
Walsh 2002 (19)	0.0041	0.7338	738	78	1.0%	1.00 [0.24, 4.23]
Yadav, 2014 (20)	−0.3071	0.1204	564	759	9.8%	0.74 [0.58, 0.93]
Young 2021 (21)	0.2174	0.3235	112	58	3.8%	1.24 [0.66, 2.34]
Young 2021 (22)	−0.0339	0.3472	112	58	3.4%	0.97 [0.49, 1.91]
**Total (95% CI)**			**9098**	**14290**	**100.0%**	**0.89 [0.77, 1.03]**

Heterogeneity: $Tau^2 = 0.04$; $Chi^2 = 39.89$, df = 21 (P = 0.008); $I^2 = 47\%$
Test for overall effect: Z = 1.61 (P = 0.11)

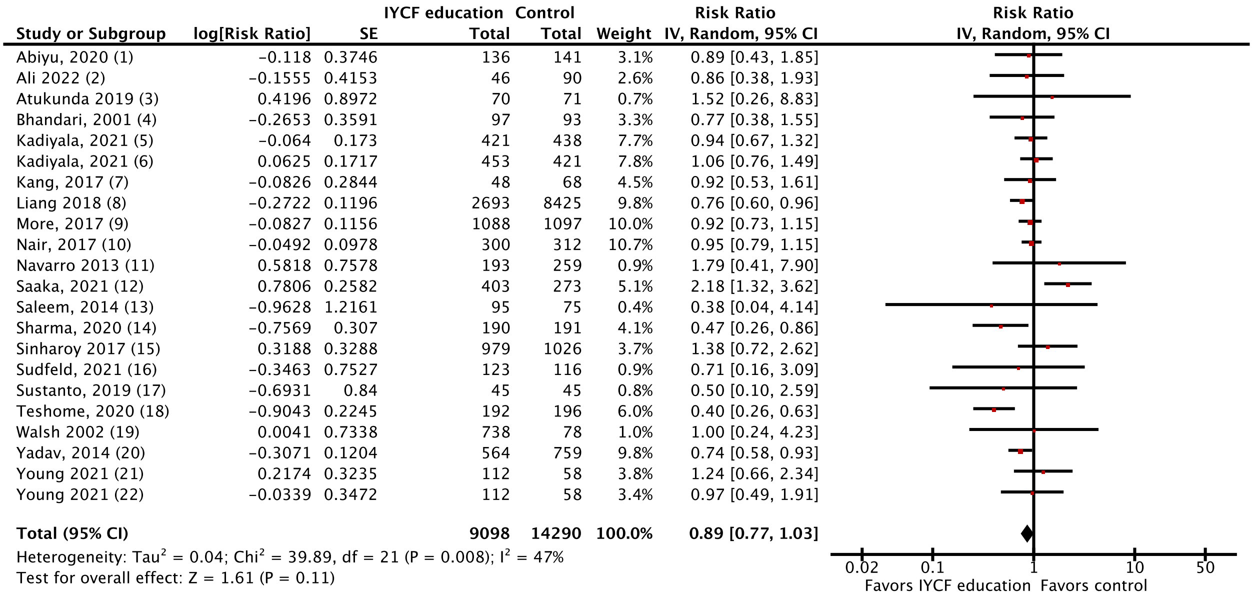

Footnotes
(1) cRCT; African Region; IYCF vs control; children aged 12−24 months; Intervention for 9 months; Prevalence of wasting (endline WHZ <−2) at 9 months; study...
(2) RCT, Somalia, Africa, nutrition couselling, IDP children
(3) cRCT; African Region; IYCF vs control; Children aged 6−8 months; Follow−up at 20−24 months; prevalence of wasting (Endline WHZ<−2)
(4) RCT; South East Asia; nutritional counselling vs control; children aged 4−12 months; intervention for 12 months; prevalence of wasting (Endline WHZ < −2)
(5) cRCT; South East Asia; Nutrition+Agriculture vs agriculture; children aged 6−23 months; Intervention for 36 months; prevalence of wasting (Endline WHZ<−2)
(6) cRCT; South East Asia; Nutrition+Agriculture+ PLA vs agriculture+Nutrition; children aged 6−23 months; Intervention for 36 months; prevalence of wasting...
(7) cRCT; African Region; IYCF (community−based participatory nutrition promotion (CPNP)) vs control; children aged 6−12 months; intervention for 12 months;...
(8) non−RCT; Western Pacific region; IYCF vs control; children <3 years; Intervention for 4 years; prevalence of wasting (Endline WHZ<−2)
(9) cRCT; South East Asia; integrated activities vs control; children aged 5 years; prevalence of wasting (Endline WHZ<−2)
(10) cRCT; South East Asia; support feeding, hygiene, care, and stimulation; vs control; children aged 0−18 months; Intervention for 18 months; prevalence of...
(11) non−RCT, Regions of America, IYCF vs control; children <2 years; Intervention for 24 months; prevalence of wasting (Endline WHZ<−2)
(12) non−RCT; African Region; IYCF vs control; children aged 6−36 months; Intervention for 12 months Prevalence of wasting (Endline WHZ < −2) after 12 months;
(13) cRCT; EMRO; IYCF vs control; children newborn; Intervention for 10 weeks; Prevalence of wasting (Endline WHZ<−2)
(14) non−RCT; South East Asia; nutritional education arm vs control; children aged 4−6 months; Intervention for 6 months; prevalence of wasting (Endline WHZ<−2
(15) cRCT; Western Rwanda; Classic intervention vs control; children <5 years; Intervention for 2 years; prevalence of wasting (Endline WHZ <−2)
(16) cRCT; African Region; integrated health nutrition and responsive stimulation vs control; children <1 years; Intervention for 18 months; Prevalence of wasting..
(17) cRCT; South East Asia; growth, nutrition and development intervention vs control; children aged 0−72 months; Intervention for 12 weeks; prevalence of...
(18) cRCT; African Region; Pulse education nutrition program vs control; children aged 6−15 months; Intervention for 9 month; prevalence of wasting (Endline...
(19) non−RCT; rural Africa; EBF vs. control for 6 months; intervention provided to mothers; prevalence of wasting (WAZ <−3 to <−2) at 2 years
(20) non−RCT; South East Asia; IYCF vs control; children aged <3 years; intervention for 12 months; prevalence of wasting (Endline WHZ<−2)
(21) cRCT; Cambodia, Western Pacific; traditional Positive Deviance/Hearth programme (PDH); prevalence at 3 months followup
(22) cRCT; Cambodia, Western Pacific; traditional Positive Deviance/Hearth with Interactive Voice Calling (PDH−IVC);prevalence at 3 months followup

Severe Wasting

Study or Subgroup	log[Risk Ratio]	SE	IYCF education Total	Control Total	Weight	Risk Ratio IV, Random, 95% CI
Sustanto, 2019 (1)	-1.7918	1.0593	45	45	63.9%	0.17 [0.02, 1.33]
Walsh 2002 (2)	-2.2472	1.4092	738	78	36.1%	0.11 [0.01, 1.67]
**Total (95% CI)**			**783**	**123**	**100.0%**	**0.14 [0.03, 0.74]**

Heterogeneity: $Tau^2 = 0.00$; $Chi^2 = 0.07$, df = 1 (P = 0.80); $I^2 = 0\%$
Test for overall effect: Z = 2.31 (P = 0.02)

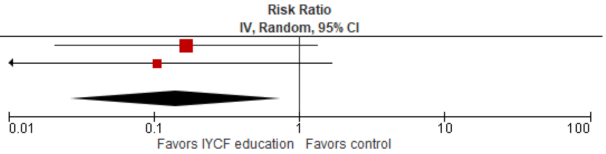

Footnotes
(1) cRCT; South East Asia; growth, nutrition and development intervention vs control; children aged 0-72 months; Intervention for 12 weeks; prevalence of severe wasting (Endline WHZ<-3)
(2) non-RCT; rural Africa; EBF vs. control for 6 months; intervention provided to mothers; severe wasting (WHZ <-3) at 2 years

WAZ

Study or Subgroup	Mean Difference	SE	IYCF education Total	Control Total	Weight	Mean Difference IV, Random, 95% CI
Atukunda 2019 (1)	0.09	0.2347	69	69	2.3%	0.09 [−0.37, 0.55]
Hitachi, 2020 (2)	−0.02	0.041	67	113	8.3%	−0.02 [−0.10, 0.06]
Kang, 2017 (3)	0.028	0.0061	56	69	9.0%	0.03 [0.02, 0.04]
Martinez, 2018 (4)	0.08	0.0523	145	151	7.9%	0.08 [−0.02, 0.18]
Nair, 2017 (5)	0.04	0.0847	302	313	6.6%	0.04 [−0.13, 0.21]
Olaya 2013 (6)	0.19	0.1021	38	38	5.8%	0.19 [−0.01, 0.39]
Owais, 2017 (7)	0	0.0806	351	341	6.7%	0.00 [−0.16, 0.16]
Penny 2005 (8)	0.29	0.1409	75	76	4.4%	0.29 [0.01, 0.57]
Reinbott, 2016 (9)	−0.02	0.1128	175	132	5.4%	−0.02 [−0.24, 0.20]
Roy, 2007 (10)	0.24	0.0697	290	282	7.2%	0.24 [0.10, 0.38]
Roy 2022 (11)	0.82	0.1523	96	96	4.1%	0.82 [0.52, 1.12]
Salehi, 2004 (12)	0.45	0.0738	406	405	7.0%	0.45 [0.31, 0.59]
Sharma, 2020 (13)	0.32	0.0754	190	191	7.0%	0.32 [0.17, 0.47]
Singh, 2017 (14)	0.1	0.081	402	340	6.7%	0.10 [−0.06, 0.26]
Sudfeld, 2021 (15)	0.28	0.1225	123	116	5.1%	0.28 [0.04, 0.52]
Zaman 2008 (16)	0.546	0.2379	95	95	2.3%	0.55 [0.08, 1.01]
Zhang, 2013 (17)	0.27	0.1502	78	71	4.1%	0.27 [−0.02, 0.56]
**Total (95% CI)**			**2958**	**2898**	**100.0%**	**0.18 [0.10, 0.27]**

Heterogeneity: $Tau^2 = 0.02$; $Chi^2 = 102.62$, df = 16 ($P < 0.00001$); $I^2 = 84\%$
Test for overall effect: Z = 4.43 ($P < 0.00001$)

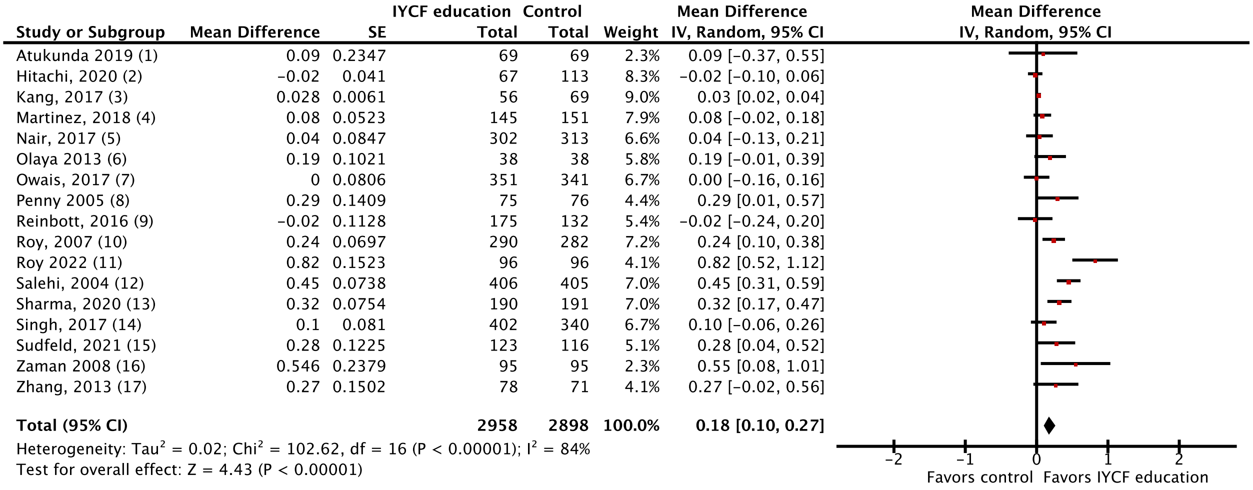

Footnotes
(1) WAZ; cRCT; African Region; IYCF vs control; Children aged 6–8 months; Follow–up at 20–24 months
(2) Education on maternal and child nutrition and follow–up consultations; non–RCT; African Region; children aged 5–59 months; follow–up 12 months; change in..
(3) change in WAZ; cRCT; African Region; IYCF [community–based participatory nutrition promotion (CPNP)'] vs control; children aged 6–12 months; intervention...
(4) RCT; Regions of America; children aged 6–24 months; Follow–up 6 months; change in WAZ
(5) cRCT; South East Asia; support feeding, hygiene, care, and stimulation; vs control; children aged 0–18 months; Intervention for 18 months; WAZ at 18 months
(6) RCT, Region of Americas, children of 6months, change in WAZ
(7) Community–based nutrition education program; RCT; Bangladesh; children aged 0–23 months; at 24 months
(8) cRCT; Peru; children aged 4 months; follow–up at 18 months, endline
(9) IYCG and agricultural activities; cRCT; Western Pacific; children aged 0–23 months; Follow–up at 2 years
(10) RCT; South East Asia; children aged 6–9 months; Follow–up 12 months; after intervention
(11) RCT, Bangladesh, Asia, 6–23 months, IYCF
(12) RCT; EMRO; environmental health, personal hygiene and nutritional edu vs control; children aged 0–59 months; Intervention for 12 months
(13) WAZ; non–RCT; South East Asia; nutritional education arm vs control; children aged 4–6 months; Intervention for 6 months
(14) Integrated Nutrition and Health Program (INHP II); qRCT; South East Asia; children aged 6 months to 6 years; endline at 18 momths
(15) WAZ; cRCT; African Region; integrated health nutrition and responsive stimulation vs control; children <1 years; Intervention for 18 months
(16) cRCT; Pakistan; 6–18 months old children; duration of 180 days
(17) cRCT; Western Pacific Region; children at 6 months; at 18 months

Prevalence of underweight

Study or Subgroup	log[Risk Ratio]	SE	IYCF education Total	Control Total	Weight	Risk Ratio IV, Random, 95% CI
Abiyu, 2020 (1)	−0.4119	0.2384	136	141	3.3%	0.66 [0.42, 1.06]
Ali 2022 (2)	−0.0455	0.195	46	90	4.3%	0.96 [0.65, 1.40]
Atukunda 2019 (3)	−0.14	0.5302	70	71	0.9%	0.87 [0.31, 2.46]
Gope, 2019 (4)	−0.1108	0.0399	1235	1100	9.4%	0.90 [0.83, 0.97]
Guldan 2000 (5)	−2.7881	1.4574	220	203	0.1%	0.06 [0.00, 1.07]
Kabahenda, 2014 (6)	0.1186	0.7313	45	38	0.5%	1.13 [0.27, 4.72]
Kang, 2017 (7)	−0.179	0.2366	56	69	3.3%	0.84 [0.53, 1.33]
Liang 2018 (8)	−0.3299	0.0875	2693	8425	7.8%	0.72 [0.61, 0.85]
More, 2017 (9)	0.021	0.0538	1088	1098	9.0%	1.02 [0.92, 1.13]
Nair, 2017 (10)	−0.0508	0.0608	302	313	8.8%	0.95 [0.84, 1.07]
Roy, 2007 (11)	0.1193	0.0443	290	282	9.2%	1.13 [1.03, 1.23]
Saaka, 2021 (12)	0.5055	0.1759	403	273	4.8%	1.66 [1.17, 2.34]
Saleem, 2014 (13)	−0.47	0.2654	95	75	2.9%	0.63 [0.37, 1.05]
Sharma, 2020 (14)	−0.6338	0.2644	190	191	2.9%	0.53 [0.32, 0.89]
Singh, 2017 (15)	0.0277	0.0741	402	340	8.3%	1.03 [0.89, 1.19]
Sudfeld, 2021 (16)	−1.157	0.8062	123	116	0.4%	0.31 [0.06, 1.53]
Sustanto, 2019 (17)	0	0.6749	45	45	0.6%	1.00 [0.27, 3.75]
Teshome, 2020 (18)	−0.9043	0.2245	192	196	3.6%	0.40 [0.26, 0.63]
Walsh 2002 (19)	−0.2934	0.2219	738	78	3.6%	0.75 [0.48, 1.15]
Yadav, 2014 (20)	−0.3459	0.1089	564	759	7.0%	0.71 [0.57, 0.88]
Young 2021 (21)	−0.0625	0.1771	112	58	4.7%	0.94 [0.66, 1.33]
Young 2021 (22)	−0.0885	0.1789	112	58	4.7%	0.92 [0.64, 1.30]
**Total (95% CI)**			**9157**	**14019**	**100.0%**	**0.87 [0.78, 0.97]**

Heterogeneity: $Tau^2 = 0.03$; $Chi^2 = 78.56$, df = 21 (P < 0.00001); $I^2 = 73\%$
Test for overall effect: Z = 2.59 (P = 0.010)

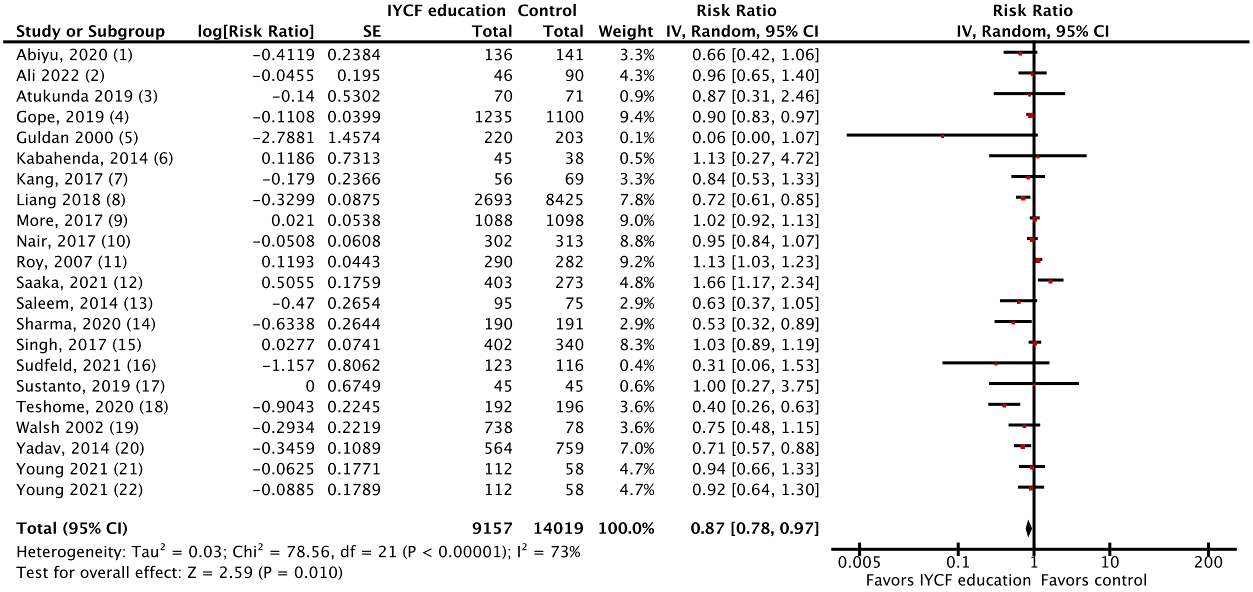

Footnotes
(1) cRCT; African Region; IYCF vs control; children aged 12–24 months; Intervention for 9 months; prevalence of underweight (WAZ<−2)
(2) RCT, Somalia, Africa, nutrition couselling, IDP children
(3) cRCT; African Region; IYCF vs control; Children aged 6–8 months; Follow-up at 20–24 months; prevalence of underweight (WAZ<−2)
(4) non-RCT; South-East Asia; PLA vs control; children aged 6 months–3 years; Intervention for 24 months; Prevalence of underweight (WAZ< −2);
(5) non-RCT; China; 4–12 months; intervention for 1 year
(6) non-RCT; African Region; Structured nutrition education programme vs control; children aged 6–48 months; Intervention for 9 months; incidence of...
(7) cRCT; African Region; IYCF [community-based participatory nutrition promotion (CPNP)’] vs control; children aged 6–12 months; intervention for 12 months;...
(8) non-RCT; Western Pacific region; IYCF vs control; children <3 years; Intervention for 4 years; prevalence of underweight (WAZ<−2)
(9) cRCT; South East Asia; integrated activities vs control; children aged 5 years; prevalence of underweight (WAZ<−2)
(10) cRCT; South East Asia; support feeding, hygiene, care, and stimulation vs control; children aged 0–18 months; Intervention for 18 months; prevalence of...
(11) RCT; South East Asia; children aged 6–9 months; Follow-up 12 months; after intervention; proportion of children WAM <25% NCHS standard
(12) non-RCT; African Region; IYCF vs control; children aged 6–36 months; Intervention for 12 months Prevalence of underweight (WAZ < −2)
(13) cRCT; EMRO; IYCF vs control; children newborn; Intervention for 10 weeks; Prevalence of underweight (WAZ<−2)
(14) non-RCT; South East Asia; nutritional education arm vs control; children aged 4–6 months; Intervention for 6 months; prevalence of underweight (WAZ<−2)
(15) Integrated Nutrition and Health Program (INHP II); qRCT; South East Asia; children aged 6 months to 6 years; endline at 18 momths
(16) cRCT; African Region; integrated health nutrition and responsive stimulation vs control; children <1 years; Intervention for 18 months; Prevalence of...
(17) cRCT; South East Asia; growth, nutrition and development intervention vs control; children aged 0–72 months; Intervention for 12 weeks; prevalence of...
(18) cRCT; African Region; Pulse education nutrition program vs control; children aged 6–15 months; Follow-up 9 month; prevalence of underweight (WAZ<−2)
(19) non-RCT; rural Africa; EBF vs. control for 6 months; intervention provided to mothers; prevalence of underweight (WAZ <−3 to <−2) at 2 years
(20) non-RCT; South East Asia; IYCF vs control; children aged <3 years; intervention for 12 months; prevalence of underweight (WAZ<−2)
(21) cRCT; Cambodia, Western Pacific; traditional Positive Deviance/Hearth with Interactive Voice Calling (PDH-IVC);prevalence at 3 months followup
(22) cRCT; Cambodia, Western Pacific; traditional Positive Deviance/Hearth programme (PDH); prevalence at 3 months followup

Severely underweight

Study or Subgroup	log[Risk Ratio]	SE	IYCF education Total	Control Total	Weight	Risk Ratio IV, Random, 95% CI
Walsh 2002 (1)	-0.948	0.4449	738	78	100.0%	0.39 [0.16, 0.93]
**Total (95% CI)**			**738**	**78**	**100.0%**	**0.39 [0.16, 0.93]**

Heterogeneity: Not applicable
Test for overall effect: Z = 2.13 (P = 0.03)

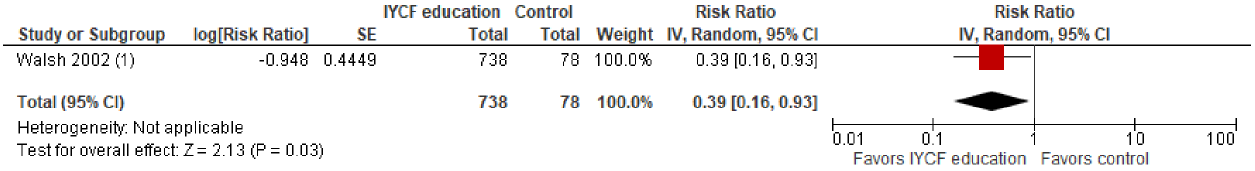

Footnotes
(1) non-RCT; rural Africa; EBF vs. control for 6 months; intervention provided to mothers; severe underweight (WAZ <-3) at 2 years

Change in weight

Study or Subgroup	Mean Difference	SE	IYCF education Total	Control Total	Weight	Mean Difference IV, Random, 95% CI
Abiyu, 2020 (1)	0.46	0.0777	136	141	8.3%	0.46 [0.31, 0.61]
Bhandari, 2001 (2)	−0.06	0.0904	97	93	8.0%	−0.06 [−0.24, 0.12]
Bhandari, 2003 (3)	−0.01	0.0639	218	197	8.5%	−0.01 [−0.14, 0.12]
Kang, 2017 (4)	0.031	0.0061	76	79	9.2%	0.03 [0.02, 0.04]
Nayak 2016 (5)	0.736	0.4804	23	23	1.7%	0.74 [−0.21, 1.68]
Penny 2005 (6)	0.29	0.2187	75	76	4.8%	0.29 [−0.14, 0.72]
Roy 2022 (7)	1.05	0.2037	96	96	5.2%	1.05 [0.65, 1.45]
Saleem, 2014 (8)	0.35	0.1527	95	75	6.4%	0.35 [0.05, 0.65]
Salehi, 2004 (9)	0.74	0.0879	406	405	8.0%	0.74 [0.57, 0.91]
Sharma, 1987 (10)	−0.19	0.1703	83	70	5.9%	−0.19 [−0.52, 0.14]
Sharma, 2020 (11)	0.16	0.083	190	191	8.1%	0.16 [−0.00, 0.32]
Singh, 2017 (12)	−0.1	0.081	402	340	8.2%	−0.10 [−0.26, 0.06]
Vazir, 2013 (13)	0.1	0.1649	117	61	6.1%	0.10 [−0.22, 0.42]
Vazir, 2013 (14)	−0.1	0.1693	102	60	6.0%	−0.10 [−0.43, 0.23]
Zhang, 2013 (15)	0.24	0.1839	80	73	5.6%	0.24 [−0.12, 0.60]
**Total (95% CI)**			**2196**	**1980**	**100.0%**	**0.20 [0.07, 0.34]**

Heterogeneity: $Tau^2 = 0.05$; $Chi^2 = 137.49$, df = 14 ($P < 0.00001$); $I^2 = 90\%$
Test for overall effect: Z = 2.93 (P = 0.003)

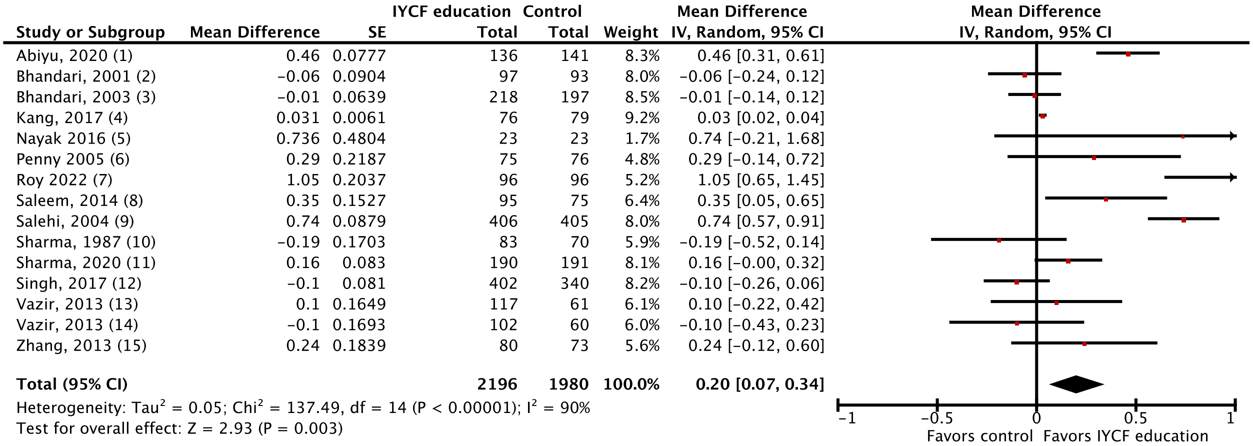

Footnotes
(1) weight gain; cRCT; African Region; IYCF vs control; children aged 12–24 months; Intervention for 9 months; study reported cluster adjusted estimates (DID)
(2) RCT; South East Asia; nutritional counselling vs control; children aged 4–12 months; Intervention for 12 months; weight change at 6 months
(3) cRCT; South East Asia; children aged 6–18 months; Intervention for 6 months; weight gain between 12–18 months
(4) change in weight/ month; cRCT; African Region; IYCF [community-based participatory nutrition promotion (CPNP)'] vs control; children aged 6–12 months;...
(5) family level counselling; cRCT; India; mother in their last trimester, follow-up at 24 months; g
(6) cRCT; Peru; children aged 4 months; follow-up at 18 months, endline
(7) RCT, Bangladesh, Asia, 6–23 months, IYCF
(8) cRCT; EMRO; IYCF vs control; children newborn; Intervention for 10 weeks; weight in kgs at endline
(9) Weight; RCT; EMRO; environmental health, personal hygiene and nutritional edu vs control; children aged 0–59 months; Intervention for 12 months
(10) nutrition edu; interventional study; weight; South East Asia; Children aged 0–12 months; 9–12 months
(11) weight; non-RCT; South East Asia; nutritional education arm vs control; children aged 4–6 months; Intervention for 6 months
(12) Integrated Nutrition and Health Program (INHP II); qRCT; South East Asia; children aged 6 months to 6 years; endline at 18 momths
(13) kgs; CF education cRCT; South East Asia; children aged 3–15 months; Follow-up 15 months
(14) kgs; CF education+PG; cRCT; South East Asia; children aged 3–15 months; Follow-up 15 months
(15) kgs; cRCT; Western Pacific Region; children at 6 months; at 18 months; change in wt

Change in height

Study or Subgroup	Mean Difference	SE	IYCF education Total	Control Total	Weight	Mean Difference IV, Random, 95% CI
Bhandari, 2001 (1)	−0.2	0.2252	97	93	2.2%	−0.20 [−0.64, 0.24]
Bhandari, 2003 (2)	−0.06	0.1924	218	197	3.0%	−0.06 [−0.44, 0.32]
Kang, 2017 (3)	0.059	0.0163	76	79	41.9%	0.06 [0.03, 0.09]
Penny 2005 (4)	1.07	0.4395	75	76	0.6%	1.07 [0.21, 1.93]
Roy 2022 (5)	2.29	0.71	96	96	0.2%	2.29 [0.90, 3.68]
Saleem, 2014 (6)	0.66	0.4997	95	75	0.5%	0.66 [−0.32, 1.64]
Salehi, 2004 (7)	0.0163	0.0035	406	405	46.0%	0.02 [0.01, 0.02]
Sharma, 2020 (8)	0.15	0.227	190	191	2.2%	0.15 [−0.29, 0.59]
Singh, 2017 (9)	−0.2	0.2586	402	340	1.7%	−0.20 [−0.71, 0.31]
Vazir, 2013 (10)	0.5	0.4228	117	61	0.6%	0.50 [−0.33, 1.33]
Vazir, 2013 (11)	0.3	0.4344	102	60	0.6%	0.30 [−0.55, 1.15]
Zhang, 2013 (12)	0.69	0.5005	80	73	0.5%	0.69 [−0.29, 1.67]
**Total (95% CI)**			**1954**	**1746**	**100.0%**	**0.05 [−0.02, 0.12]**

Heterogeneity: $Tau^2 = 0.00$; $Chi^2 = 29.85$, df = 11 ($P = 0.002$); $I^2 = 63\%$
Test for overall effect: Z = 1.43 (P = 0.15)

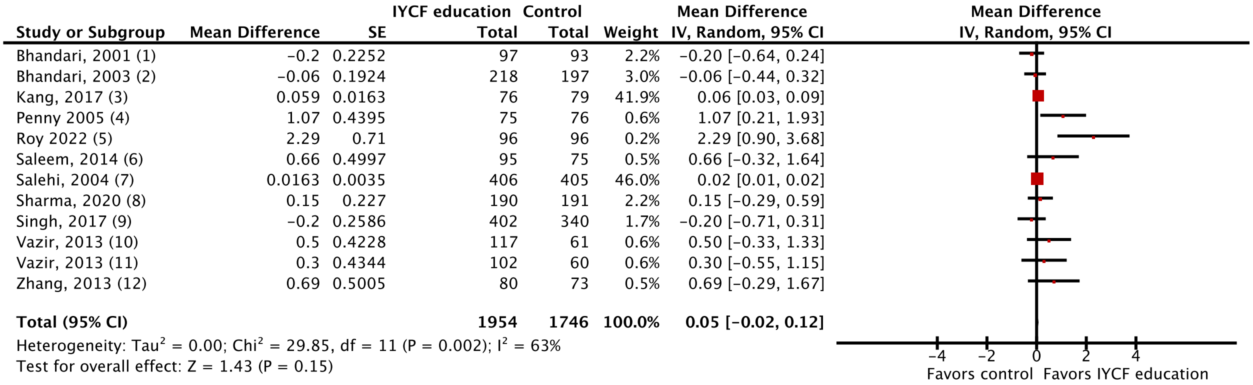

Footnotes
(1) RCT; South East Asia; nutritional counselling vs control; children aged 4–12 months; Intervention for 12 months; change in height
(2) cRCT; South East Asia; children aged 6–18 months; Intervention for 6 months; height gain at 12–18 months
(3) change in height/ month; cRCT; African Region; IYCF [community-based participatory nutrition promotion (CPNP)'] vs control; children aged 6–12 months;...
(4) cRCT; Peru; children aged 4 months; follow-up at 18 months; endline
(5) RCT, Bangladesh, Asia, 6–23 months, IYCF
(6) cRCT; EMRO; IYCF vs control; children newborn; Intervention for 10 weeks; height in cm at endline
(7) RCT; EMRO; environmental health, personal hygiene and nutritional edu vs control; children aged 0–59 months; Intervention for 12 months; Height
(8) height; non-RCT; South East Asia; nutritional education arm vs control; children aged 4–6 months; Intervention for 6 months
(9) Integrated Nutrition and Health Program (INHP II); qRCT; South East Asia; children aged 6 months to 6 years; endline at 18 momths
(10) CF education cRCT; South East Asia; children aged 3–15 months; Follow-up 15 months
(11) CF education+PG; cRCT; South East Asia; children aged 3–15 months; Follow-up 15 months
(12) kgs; cRCT; Western Pacific Region; children at 6 months; at 18 months; change in height

Change in MUAC (cm)

Study or Subgroup	Mean Difference	SE	IYCF education Total	Control Total	Weight	Mean Difference IV, Random, 95% CI
Nair, 2017 (1)	0.1	5.3109	308	320	0.0%	0.10 [−10.31, 10.51]
Roy, 2007 (2)	−0.02	0.0744	290	282	29.4%	−0.02 [−0.17, 0.13]
Roy 2022 (3)	0.6	0.1805	96	96	16.8%	0.60 [0.25, 0.95]
Saleem, 2014 (4)	0.46	0.1571	95	75	19.2%	0.46 [0.15, 0.77]
Salehi, 2004 (5)	0.005	0.001	406	405	34.7%	0.01 [0.00, 0.01]
**Total (95% CI)**			**1195**	**1178**	**100.0%**	**0.18 [−0.02, 0.39]**

Heterogeneity: $Tau^2 = 0.03$; $Chi^2 = 19.37$, df = 4 (P = 0.0007); $I^2 = 79\%$
Test for overall effect: Z = 1.80 (P = 0.07)

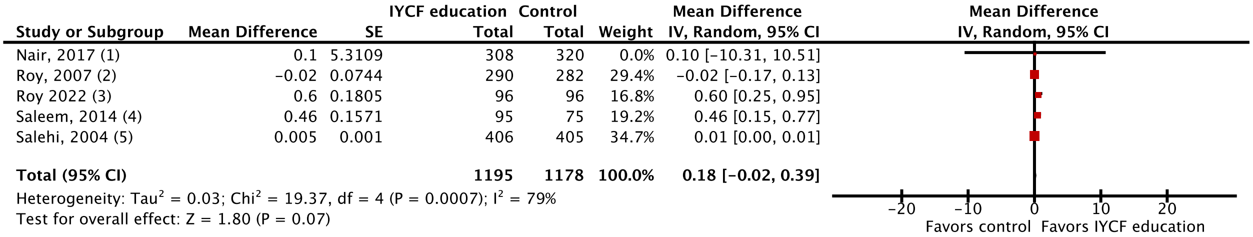

Footnotes
(1) cRCT; South East Asia; support feeding, hygiene, care, and stimulation; vs control; children aged 0–18 months; Intervention for 18 months; MUAC in cm at 18...
(2) RCT; South East Asia; children aged 6–9 months; Follow-up 12 months; after intervention
(3) RCT, Bangladesh, Asia, 6–23 months, IYCF
(4) cRCT; EMRO; IYCF vs control; children newborn; Intervention for 10 weeks; MUAC in cm at endline
(5) RCT; EMRO; environmental health, personal hygiene and nutritional edu vs control; children aged 0–59 months; Intervention for 12 months; MUAC cm

Change in MUAC (z-score)

Study or Subgroup	Mean Difference	SE	IYCF education Total	Control Total	Weight	Mean Difference IV, Random, 95% CI
Atukunda 2019 (1)	−0.01	0.1225	229	209	36.6%	−0.01 [−0.25, 0.23]
Olaya 2013 (2)	0.06	0.1617	38	38	31.0%	0.06 [−0.26, 0.38]
Roy 2022 (3)	0.47	0.1516	96	96	32.4%	0.47 [0.17, 0.77]
**Total (95% CI)**			**363**	**343**	**100.0%**	**0.17 [−0.13, 0.46]**

Heterogeneity: $Tau^2 = 0.05$; $Chi^2 = 6.47$, df = 2 (P = 0.04); $I^2 = 69\%$
Test for overall effect: Z = 1.11 (P = 0.27)

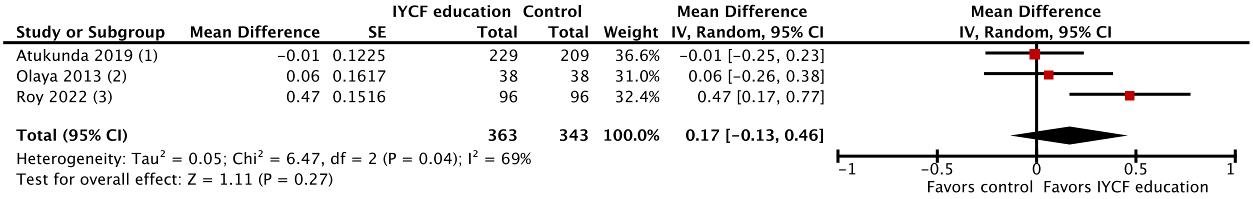

Footnotes
(1) z score; cRCT; African Region; IYCF vs control; Children aged 6–8 months; Follow-up at 20–24 months
(2) RCT, Region of Americas, children of 6months, change in MUAC–Z
(3) RCT, Bangladesh, Asia, 6–23 months, IYCF

Anemia

Study or Subgroup	log[Risk Ratio]	SE	IYCF education Total	Control Total	Weight	Risk Ratio IV, Random, 95% CI
Fahmida, 2020 (1)	-0.3038	0.088	218	214	35.4%	0.74 [0.62, 0.88]
Guldan 2000 (2)	-0.3836	0.1635	220	203	28.8%	0.68 [0.49, 0.94]
Vitolo 2005 (3)	0.1308	0.0827	163	234	35.8%	1.14 [0.97, 1.34]
**Total (95% CI)**			**601**	**651**	**100.0%**	**0.84 [0.60, 1.18]**

Heterogeneity: $Tau^2 = 0.08$; $Chi^2 = 16.13$, df = 2 (P = 0.0003); $I^2 = 88\%$
Test for overall effect: Z = 1.00 (P = 0.32)

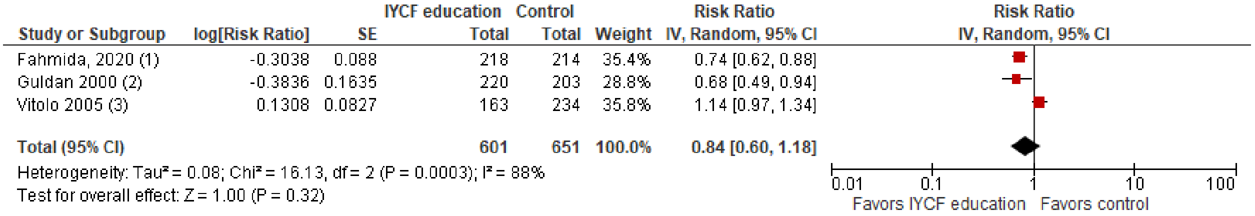

Footnotes
(1) integrated ICF; cRCT; Indonesia; children aged 0-18 months; follow-up 18 months
(2) non-RCT; China; 4-12 months; intervention for 1 year; <110g/l
(3) RCT, Region of the Americas, mothers of children 6-12 months, 2 visits for 12 months; Hb <11g/dl

Hb levels

Study or Subgroup	Mean Difference	SE	IYCF education Total	Control Total	Weight	Mean Difference IV, Random, 95% CI
Fahmida, 2020 (1)	0.09	0.1296	218	214	25.4%	0.09 [-0.16, 0.34]
Guldan 2000 (2)	3.5	1.0975	220	203	2.4%	3.50 [1.35, 5.65]
Kadiyala, 2021 (3)	-0.1	0.0983	421	438	27.0%	-0.10 [-0.29, 0.09]
Kadiyala, 2021 (4)	0.0625	0.1717	453	421	23.1%	0.06 [-0.27, 0.40]
Olaya 2013 (5)	0.6	0.1884	36	37	22.1%	0.60 [0.23, 0.97]
**Total (95% CI)**			**1348**	**1313**	**100.0%**	**0.23 [-0.12, 0.58]**

Heterogeneity: $Tau^2 = 0.11$; $Chi^2 = 20.69$, df = 4 (P = 0.0004); $I^2 = 81\%$
Test for overall effect: Z = 1.28 (P = 0.20)

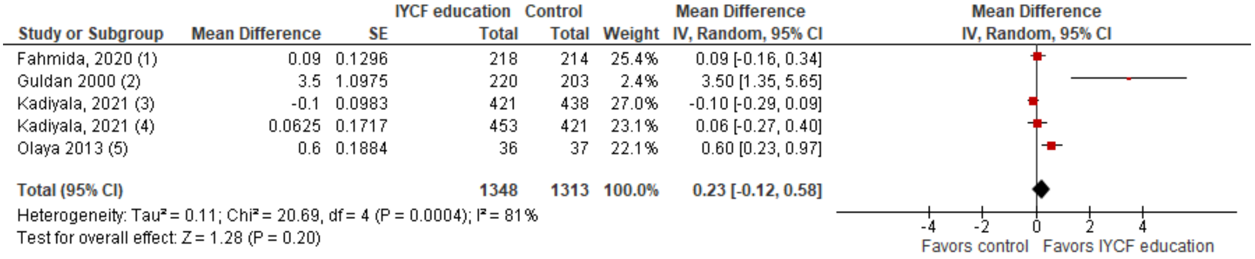

Footnotes
(1) integrated ICF; cRCT; Indonesia; children aged 0-18 months; follow-up 18 months
(2) non-RCT; China; 4-12 months; intervention for 1 year; <110g/l
(3) cRCT; South East Asia; Nutrition+Agriculture vs agriculture; children aged 6-23 months; Intervention for 36 months
(4) cRCT; South East Asia; Nutrition+Agriculture+ PLA vs agriculture+Nutrition; children aged 6-23 months; Intervention for 36 months
(5) RCT, Region of Americas, children of 6months, at endline

Diarrhea

Study or Subgroup	log[Risk Ratio]	SE	IYCF education Total	Control Total	Weight	Risk Ratio IV, Random, 95% CI
Abiyu, 2020 (1)	−0.1646	0.23	136	141	12.3%	0.85 [0.54, 1.33]
Bhandari, 2003 (2)	0.2515	0.2363	218	197	11.9%	1.29 [0.81, 2.04]
Reinbott, 2016 (3)	0.0545	0.1889	175	132	16.1%	1.06 [0.73, 1.53]
Roy 2022 (4)	−0.2744	0.268	96	96	9.8%	0.76 [0.45, 1.29]
Sinharoy 2017 (5)	0.0045	0.1042	1083	1130	28.5%	1.00 [0.82, 1.23]
Vitolo 2005 (6)	−0.3948	0.1467	163	234	21.4%	0.67 [0.51, 0.90]
**Total (95% CI)**			**1871**	**1930**	**100.0%**	**0.91 [0.76, 1.10]**

Heterogeneity: $Tau^2 = 0.02$; $Chi^2 = 8.39$, df = 5 (P = 0.14); $I^2 = 40\%$
Test for overall effect: Z = 0.96 (P = 0.34)

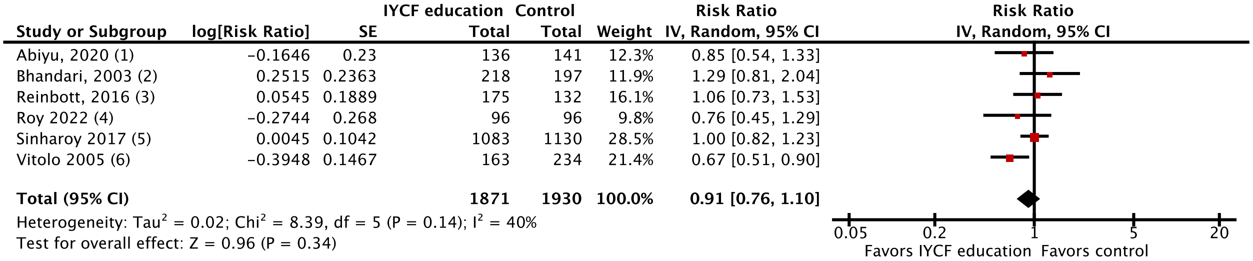

Footnotes
(1) cRCT; African Region; IYCF vs control; children aged 12–24 months; Intervention for 9 months
(2) cRCT; South East Asia; IYCF vs visitation; children aged 6–18 months; Intervention for 12 months; Diarrhea was defined as passage or 3 or more liquid stools..
(3) IYCG and agricultural activities; cRCT; Western Pacific; children aged 0–23 months; Follow-up at 2 years
(4) RCT, Bangladesh, Asia, 6–23 months, IYCF
(5) cRCT; Western Rwanda; Classic intervention intervention vs control; children <5 years; Intervention for 2 years
(6) RCT, Region of the Americas, mothers of children 6–12 months, 2 visits for 12 months

Mean diarrhea episodes

Study or Subgroup	Mean Difference	SE	IYCF education Total	Control Total	Weight	Mean Difference IV, Random, 95% CI
Bhandari, 2001 (1)	0.2	0.514	82	83	100.0%	0.20 [-0.81, 1.21]
**Total (95% CI)**			**82**	**83**	**100.0%**	**0.20 [-0.81, 1.21]**

Heterogeneity: Not applicable
Test for overall effect: Z = 0.39 (P = 0.70)

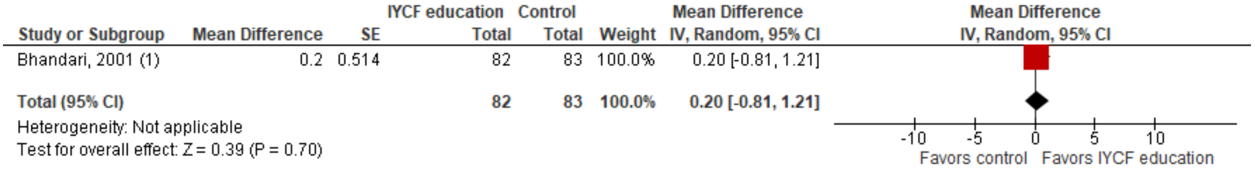

Footnotes
(1) RCT; South East Asia; nutritional counsellinh vs control; children aged 4-12 months; Intervention for 12 months; mean incidence of diarrhea

Respiratory illness

Study or Subgroup	log[Risk Ratio]	SE	IYCF education Total	Control Total	Weight	Risk Ratio IV, Random, 95% CI
Abiyu, 2020 (1)	−0.118	0.3746	136	141	7.3%	0.89 [0.43, 1.85]
Bhandari, 2003 (2)	−0.0537	0.1949	218	197	23.2%	0.95 [0.65, 1.39]
Reinbott, 2016 (3)	−0.2019	0.2003	175	132	22.2%	0.82 [0.55, 1.21]
Roy 2022 (4)	−0.6325	0.2631	96	96	14.0%	0.53 [0.32, 0.89]
Vitolo 2005 (5)	−0.4651	0.1543	163	234	33.2%	0.63 [0.46, 0.85]
**Total (95% CI)**			**788**	**800**	**100.0%**	**0.73 [0.60, 0.90]**

Heterogeneity: $Tau^2 = 0.01$; $Chi^2 = 4.80$, df = 4 (P = 0.31); $I^2 = 17\%$
Test for overall effect: Z = 2.95 (P = 0.003)

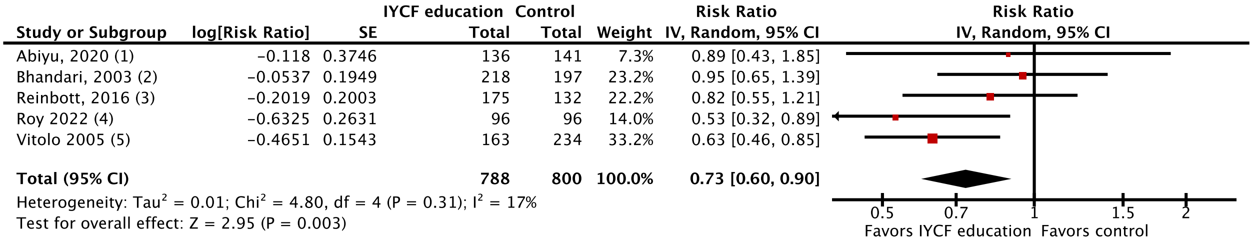

Footnotes
(1) cRCT; African Region; IYCF vs control; children aged 12–24 months; Intervention for 9 months; cough
(2) cRCT; South East Asia; IYCF vs control; children aged 6–18 months; Intervention for 12 months; cough
(3) IYCG and agricultural activities; cRCT; Western Pacific; children aged 0–23 months; Follow-up at 2 years; ARI
(4) RCT, Bangladesh, Asia, 6–23 months, IYCF, cold
(5) RCT, Region of the Americas, mothers of children 6–12 months, 2 visits for 12 months; breathing problems

Mean ALRI episodes

Study or Subgroup	Mean Difference	SE	IYCF education Total	Control Total	Weight	Mean Difference IV, Random, 95% CI
Bhandari, 2001 (1)	-0.14	0.1637	82	83	100.0%	-0.14 [-0.46, 0.18]
**Total (95% CI)**			**82**	**83**	**100.0%**	**-0.14 [-0.46, 0.18]**

Heterogeneity: Not applicable
Test for overall effect: Z = 0.86 (P = 0.39)

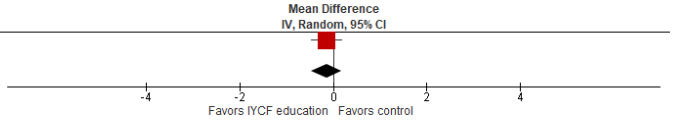

Footnotes
(1) RCT; nutritional counselling vs visitation; South East Asia; children aged 4-12 months; Intervention for 12 months; Cough with high respiratory rate (>= 50/min) or lower chest indrawingdefined an episode of acute lower...

Fever

Study or Subgroup	log[Risk Ratio]	SE	IYCF education Total	Control Total	Weight	Risk Ratio IV, Random, 95% CI
Abiyu, 2020 (1)	−0.0473	0.2627	136	141	4.1%	0.95 [0.57, 1.60]
Bhandari, 2003 (2)	0.0994	0.2402	218	197	4.9%	1.10 [0.69, 1.77]
Reinbott, 2016 (3)	−0.0867	0.0677	175	132	62.1%	0.92 [0.80, 1.05]
Roy 2022 (4)	−0.3857	0.2793	96	96	3.6%	0.68 [0.39, 1.18]
Vitolo 2005 (5)	−0.0396	0.1062	163	234	25.2%	0.96 [0.78, 1.18]
**Total (95% CI)**			**788**	**800**	**100.0%**	**0.93 [0.84, 1.03]**

Heterogeneity: Tau² = 0.00; Chi² = 1.92, df = 4 (P = 0.75); I² = 0%
Test for overall effect: Z = 1.40 (P = 0.16)

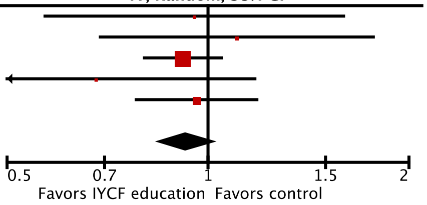

Footnotes
(1) cRCT; African Region; IYCF vs control; children aged 12–24 months; Intervention for 9 months
(2) RCT; South East Asia; IYCF vs control; children aged 6–18 months; Intervention for 12 months
(3) IYCG and agricultural activities; cRCT; Western Pacific; children aged 0–23 months; Follow-up at 2 years
(4) RCT, Bangladesh, Asia, 6–23 months, IYCF
(5) RCT, Region of the Americas, mothers of children 6–12 months, 2 visits for 12 months

Dysentery

Study or Subgroup	Mean Difference	SE	IYCF education Total	Control Total	Weight	Mean Difference IV, Random, 95% CI
Bhandari, 2001 (1)	-0.06	0.0928	82	83	100.0%	-0.06 [-0.24, 0.12]
**Total (95% CI)**			**82**	**83**	**100.0%**	**-0.06 [-0.24, 0.12]**

Heterogeneity: Not applicable
Test for overall effect: Z = 0.65 (P = 0.52)

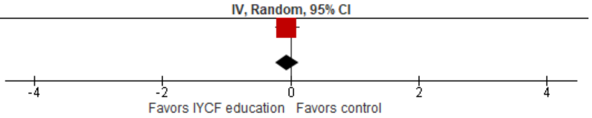

Footnotes
(1) RCT; South East Asia; IYCF vs visitation; children aged 4-12 months; Intervention for 12 months; Dysentery was defined as an episode of diarrhea in which visible blood was reported in stools

Any perceived illness (cough, diarrhea, fever)

Study or Subgroup	log[Risk Ratio]	SE	IYCF education Total	Control Total	Weight	Risk Ratio IV, Random, 95% CI
Nair, 2017 (1)	-0.027	0.1019	316	326	100.0%	0.97 [0.80, 1.19]
**Total (95% CI)**			**316**	**326**	**100.0%**	**0.97 [0.80, 1.19]**

Heterogeneity: Not applicable
Test for overall effect: Z = 0.26 (P = 0.79)

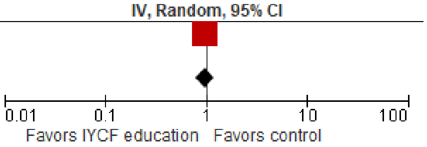

Footnotes
(1) cRCT; South East Asia; support feeding, hygiene, care, and stimulation; vs control; children aged 0-18 months; Intervention for 18 months

Mean fever episodes

Study or Subgroup	Mean Difference	SE	IYCF education Total	Control Total	Weight	Mean Difference IV, Random, 95% CI
Bhandari, 2001 (1)	0	0.3823	82	83	100.0%	0.00 [-0.75, 0.75]
**Total (95% CI)**			**82**	**83**	**100.0%**	**0.00 [-0.75, 0.75]**

Heterogeneity: Not applicable
Test for overall effect: Z = 0.00 (P = 1.00)

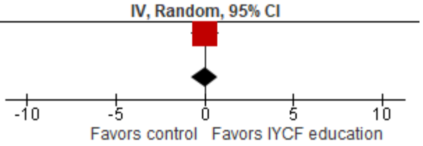

Footnotes
(1) RCT; South East Asia; nutritional counselling vs visitation; children aged 4-12 months; Intervention for 12 months, episode per child

Mortality

Study or Subgroup	log[Risk Ratio]	SE	IYCF education Total	Control Total	Weight	Risk Ratio IV, Random, 95% CI
Atukunda 2019 (1)	-0.4614	0.908	239	226	1.8%	0.63 [0.11, 3.74]
Bhandari, 2003 (2)	0.5716	0.2915	276	237	13.4%	1.77 [1.00, 3.14]
Fahmida, 2020 (3)	-0.0043	1.4111	231	230	0.8%	1.00 [0.06, 15.82]
Kang, 2017 (4)	-1.0489	1.6269	97	102	0.6%	0.35 [0.01, 8.50]
Nair, 2017 (5)	-0.2141	0.2956	370	393	13.2%	0.81 [0.45, 1.44]
Navarro 2013 (6)	-0.6797	0.8326	266	337	2.1%	0.51 [0.10, 2.59]
Penny 2005 (7)	-1.0855	1.625	75	76	0.6%	0.34 [0.01, 8.16]
Roy, 2007 (8)	-0.6964	1.2221	306	305	1.0%	0.50 [0.05, 5.47]
Sharma, 1987 (9)	0.6334	0.4158	194	172	7.6%	1.88 [0.83, 4.26]
Singh, 2017 (10)	-0.1783	0.0651	504	453	46.8%	0.84 [0.74, 0.95]
Sudfeld, 2021 (11)	-1.1291	0.8071	133	129	2.2%	0.32 [0.07, 1.57]
Yousafzai 2014 (12)	0.1046	0.3526	106	102	10.0%	1.11 [0.56, 2.22]
**Total (95% CI)**			**2797**	**2762**	**100.0%**	**0.96 [0.75, 1.22]**

Heterogeneity: Tau² = 0.03; Chi² = 13.22, df = 11 (P = 0.28); I² = 17%
Test for overall effect: Z = 0.35 (P = 0.72)

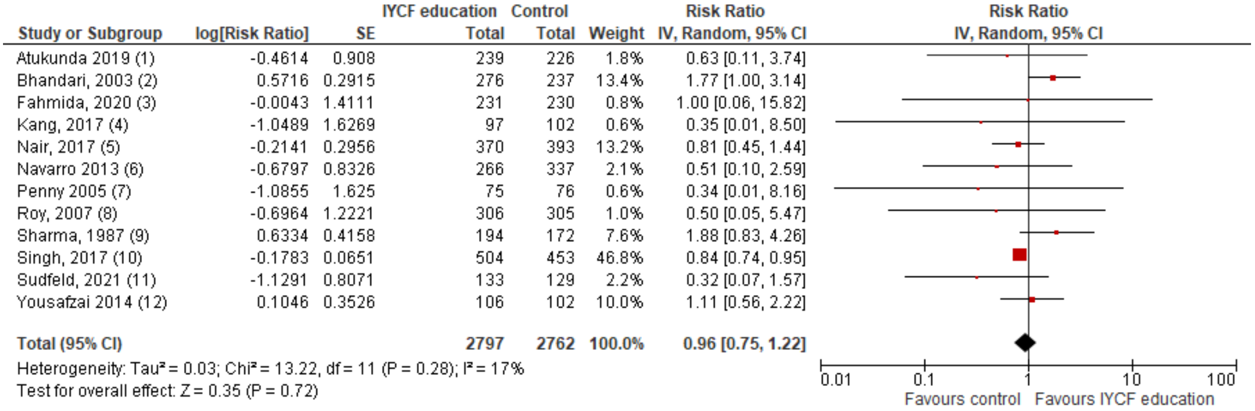

Footnotes
(1) cRCT; African Region; IYCF vs control; Children aged 6-8 months; Follow-up at 20-24 months; under 5 mortality
(2) cRCT; South East Asia; children aged 6-18 months; Intervention for 6 months; under 5 mortality
(3) integrated ICF; cRCT; Indonesia; children aged 0-18 months; follow-up 18 months; under 5 mortality
(4) cRCT; African Region; IYCF [community-based participatory nutrition promotion (CPNP)] vs control; children aged 6-12 months; under 5 mortality
(5) cRCT; South East Asia; support feeding, hygiene, care, and stimulation vs control; children aged 0-18 months; Intervention for 18 months; infant deaths
(6) non-RCT, Regions of America, IYCF vs control; children <2 years; Intervention for 24 months; under 5 mortality
(7) cRCT; Peru; children aged 4 months; follow-up at 18 months; under 5 mortality
(8) RCT; South East Asia; children aged 6-9 months; Follow-up 12 months; under 5 mortality
(9) cRCT; nutrition edu; interventional study; weight; South East Asia; Children aged 0-12 months; 9-12 months
(10) Integrated Nutrition and Health Program (INHP II); qRCT; South East Asia; children aged 6 months to 6 years; Follow-up 1 months; under 5 mortality
(11) cRCT; African Region; integrated health nutrition and responsive stimulation vs control; children <1 years; Intervention for 18 months; under 5 mortality
(12) cRCT; Pakistan, enhanced nutrition education, children upto 24 months of age; under 5 mortality

Comparison 3: Complementary food provision versus control (subgroup by food security status)

HAZ

Study or Subgroup	Mean Difference	SE	Child complementary feeding Total	Control Total	Weight	Mean Difference IV, Random, 95% CI
**2.3.1 Food insecure**						
Addo 2020 (1)	−0.56	0.0036	654	653	2.4%	−0.56 [−0.57, −0.55]
Adu-afarwah 2007 (2)	0.26	0.1505	97	81	2.2%	0.26 [−0.03, 0.55]
Adu-Afarwuah 2016 (3)	0.03	0.0761	334	337	2.4%	0.03 [−0.12, 0.18]
Agapova 2017 (4)	0.07	0.0689	116	48	2.4%	0.07 [−0.07, 0.21]
Agapova 2017 (5)	0.03	0.0704	119	48	2.4%	0.03 [−0.11, 0.17]
Akalu 2010 (6)	0.2	0.126	106	105	2.3%	0.20 [−0.05, 0.45]
Akalu 2010 (7)	0.01	0.2103	61	65	2.1%	0.01 [−0.40, 0.42]
Ashorn 2015 (8)	−0.31	0.1084	195	200	2.3%	−0.31 [−0.52, −0.10]
Bauserman 2015 (9)	0.1	0.3485	35	36	1.6%	0.10 [−0.58, 0.78]
Borg 2020 (10)	0.13	0.133	76	38	2.3%	0.13 [−0.13, 0.39]
Borg 2020 (11)	0.66	0.1495	59	39	2.2%	0.66 [0.37, 0.95]
Chipili 2022 (12)	1.36	0.1417	100	86	2.3%	1.36 [1.08, 1.64]
Christian 2015 (13)	0.04	0.0141	503	210	2.4%	0.04 [0.01, 0.07]
Christian 2015 (14)	0.03	0.0141	513	211	2.4%	0.03 [0.00, 0.06]
Christian 2015 (15)	0.02	0.0141	896	211	2.4%	0.02 [−0.01, 0.05]
Christian 2015 (16)	0.01	0.0141	513	211	2.4%	0.01 [−0.02, 0.04]
Dewey 2017 (17)	0.08	0.0566	884	408	2.4%	0.08 [−0.03, 0.19]
Dewey 2017 (18)	0.07	0.0472	785	408	2.4%	0.07 [−0.02, 0.16]
Galasso 2019 (19)	0.044	0.0898	139	139	2.4%	0.04 [−0.13, 0.22]
Ghosh 2020 (20)	0.046	0.0609	260	262	2.4%	0.05 [−0.07, 0.17]
Hess 2015 (21)	0.3	0.0575	1623	523	2.4%	0.30 [0.19, 0.41]
Humphrey 2019 (22)	0.1	0.0805	356	351	2.4%	0.10 [−0.06, 0.26]
Hurley 2021 (23)	−0.19	0.1191	176	191	2.3%	−0.19 [−0.42, 0.04]
Huybregts 2012 (24)	0.27	0.111	386	284	2.3%	0.27 [0.05, 0.49]
Iannotti 2014 (25)	−0.05	0.1865	126	72	2.1%	−0.05 [−0.42, 0.32]
Iannotti 2014 (26)	−0.02	0.1726	150	72	2.2%	−0.02 [−0.36, 0.32]
Maleta 2015 (27)	0.03	0.0724	242	48	2.4%	0.03 [−0.11, 0.17]
Maleta 2015 (28)	0.03	0.0699	245	48	2.4%	0.03 [−0.11, 0.17]
Maleta 2015 (29)	−0.93	0.0679	235	48	2.4%	−0.93 [−1.06, −0.80]
Maleta 2015 (30)	0.08	0.0692	222	49	2.4%	0.08 [−0.06, 0.22]
Maleta 2015 (31)	−0.03	0.0727	238	48	2.4%	−0.03 [−0.17, 0.11]
Mangani 2015 (32)	0.03	0.0766	188	61	2.4%	0.03 [−0.12, 0.18]
Mangani 2015 (33)	0.08	0.0723	191	61	2.4%	0.08 [−0.06, 0.22]
Mangani 2015 (34)	−0.07	0.073	183	62	2.4%	−0.07 [−0.21, 0.07]
Muslihah 2016 (35)	0.41	0.0205	59	26	2.4%	0.41 [0.37, 0.45]
Muslihah 2016 (36)	0.77	0.0215	58	25	2.4%	0.77 [0.73, 0.81]
Omer 2022 (37)	0.54	0.2082	61	61	2.1%	0.54 [0.13, 0.95]
Phu 2012 (38)	0.11	0.1142	114	64	2.3%	0.11 [−0.11, 0.33]
Phu 2012 (39)	0.16	0.0931	135	64	2.4%	0.16 [−0.02, 0.34]
Prendergast 2019 (40)	3.73	0.2138	58	58	2.1%	3.73 [3.31, 4.15]
Samuel-2018 (41)	0.48	0.0546	1025	1052	2.4%	0.48 [0.37, 0.59]
Sazawal 2014 (42)	0.16	0.0908	59	53	2.4%	0.16 [−0.02, 0.34]
Stewart 2019 (43)	0.02	0.0854	290	305	2.4%	0.02 [−0.15, 0.19]
**Subtotal (95% CI)**			**12865**	**7422**	**100.0%**	**0.18 [0.03, 0.33]**
Heterogeneity: $Tau^2 = 0.24; Chi^2 = 11984.11, df = 42 (P < 0.00001); I^2 = 100\%$						
Test for overall effect: Z = 2.37 (P = 0.02)						
**2.3.2 Food secure**						
Iannoti 2017 (44)	0.32	0.1949	75	73	14.0%	0.32 [−0.06, 0.70]
Lutter 2008 (45)	0.27	0.121	170	149	23.1%	0.27 [0.03, 0.51]
Oelofse 2003 (46)	−0.22	0.3421	16	14	6.0%	−0.22 [−0.89, 0.45]
Santos 2005 (47)	−0.02	0.1489	91	81	19.1%	−0.02 [−0.31, 0.27]
Smuts 2019 (48)	−0.13	0.1553	151	100	18.3%	−0.13 [−0.43, 0.17]
Smuts 2019 (49)	−0.13	0.145	162	101	19.6%	−0.13 [−0.41, 0.15]
**Subtotal (95% CI)**			**665**	**518**	**100.0%**	**0.04 [−0.14, 0.22]**
Heterogeneity: $Tau^2 = 0.02; Chi^2 = 8.95, df = 5 (P = 0.11); I^2 = 44\%$						
Test for overall effect: Z = 0.45 (P = 0.65)						

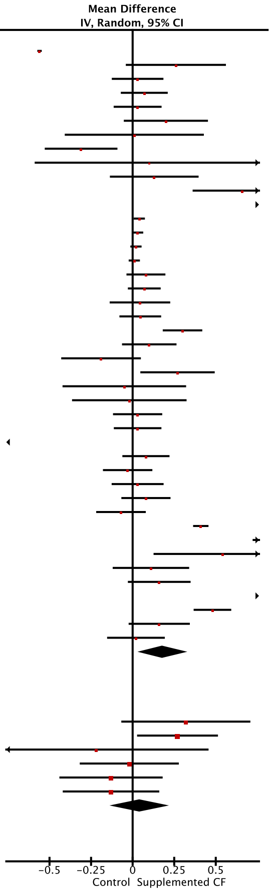

Test for subgroup differences: $Chi^2 = 1.40, df = 1 (P = 0.24), I^2 = 28.4\%$

Footnotes
(1) non-RCT; Africa; 6-18 months age, SQLNS supplementation for 3 months
(2) SQ-LNS Nutributter; qRCT; African region; mothers with >20 wk of gestation; supplementation for 6 months
(3) change in HAZ, maternal and child supplementation; SQ-LNS; RCT; African region; mothers with >20 wk of gestation; supplementation for 12 months
(4) RCT, common beans, Africa, age 12-23 months, suplementation for 24 month; change in HAZ at 12 months
(5) RCT, cowpea, Africa, age 12-23 months, suplementation for 24 month, change in HAZ at 12 months
(6) Sibu Siri- 13months; RCT: Ethopia; children aged 5-17 months; duration 1 year; endline HAZ; FBF
(7) cRCT: Africa; children aged 5-17 months; supplementation for 1 year; Wama Bomaya-13 months; endline HAZ; FBF
(8) SQLNS; RCT; Malawi; children aged 6-18 months; supplementaion for 6 months, change in HAZ
(9) cRCT, Africa, children aged 5 months; supplementation for18 months; alternative food
(10) RUSF (fish based); cRCT, Western Pacific Region, 6months age, supplementation for 6-12 months, change in HAZ
(11) CSB++ cm; cRCT, Western Pacific Region, 6months of age, supplementation for 6-12 months, change in HAZ
(12) Fish powder; RCT; Africa; children aged 6-7 months; supplementation for 6 months, endline
(13) rice/lentils; cRCT; South East Asia; aged 6-18 months; supplementation for 12 months, change in HAZ
(14) cRCT, chickpeas; South East Asia; aged 6-18 months; supplementation for 12 months
(15) Plumpy doz- RCT, South-East Asian Region, age-6-18 months; supplementation for 12 months
(16) WSB++ ; cRCT, South-East Asian Region, age-6-18 months; supplementation for 12 months; change in HAZ
(17) SQ LNS; cRCT, South-East Asia; mothers and thier children 6-24 months age, supplementation for 18 months, endline
(18) SQ LNS; cRCT, South-East Asia; children 6-24 months age, supplementation for 18 months, endline
(19) SQLNS ; RCT; Madasgar; Children <12 years; supplementation for 12 months
(20) RCT, Africa, 6-18 months age, supplementation for intervention 6-12 months; FBF
(21) zinc, cRCT, Africa, aged 9-18 months of age, SQLNS supplementation for 9 months
(22) SQLNS 20 gm-HIV negative, cRCT, Africa, Aged 6-18 month, supplementation for 12 months, endline
(23) non-RCT, Africa; children aged 6-24 months; SQLNS supplementation for 12 months
(24) Plumpy doz- RCT, South-East Asian Region, age-6-18 months; supplementation for 12 months
(25) SQ LNS; RCT African region; children aged 6-11 months; supplementation for 3 months
(26) LNS; RCT African region; children aged 6-11 months; supplementation for 6 months
(27) 40g milk; RCT, Africa; aged 6 months, supplementation for 12 months, change in HAZ
(28) 20g no milk; RCT, Africa; aged 6 months, supplementation for 12 months, change in HAZ
(29) 20g milk; RCT, Africa; aged 6 months, supplementation for 12 months, change in HAZ
(30) 10g milk; RCT, Africa; aged 6 months, supplementation for 12 months, change in HAZ
(31) 40g no milk; RCT, Africa; aged 6 months, supplementation for 12 months, change in HAZ
(32) Soy LNS; RCT, Africa, age 6-18 months; supplementation for 12 months, change in HAZ
(33) Milk LNS; RCT, Africa, age 6-18 months; supplementation for 12 months, change in HAZ
(34) CSB- RCT, Africa, age 6-18 months, suplementation for 12 month, change in HAZ
(35) Biscuits; non-RCT: Indonesia; Children aged 6-12 months; supplementation for 6 months; at endline
(36) SQ-LNS- non-RCT: Indonesia; Children aged 6-12 months; supplementation for 6 months; at endline
(37) cRCT, Ethioipia, Africa, eggs
(38) Food complement ; RCT, Western Pacific Region, 5-29 months age; supplementation for 11 months
(39) Fortified flour; RCT, Western Pacific Region, 5-29 months age; supplementation for 11 months
(40) SQLNS-20 gm-HIV positive, cRCT, Africa, Aged 6-18 month, supplementation for 12 months, endline
(41) Non RCT, Africa, Rural, 6-23 months age, endline; FBF
(42) RCT: India; children aged 6-24 months; FBF supplementation for 6 months; change in HAZ
(43) RCT, Africa, 6-9.9 months age; supplementation for 6 months; alternative food
(44) Eggs; RCT; Africa; children aged 6-9 months; supplementation for 6 months; endline
(45) Non-RCT, Region of the Americas, 6-11 months age; supplementation for 11 months; endline; FBF
(46) RCT: South Africa; children aged 6-12 months; FBF supplementation for 6 months; endline
(47) milk; non-RCT: Brazil; children aged 6-18 months; change in HAZ
(48) SQ LNS; RCT, Africa; aged 6-12 months, supplementation for 6 months, endline
(49) SQ LNS plus; RCT, Africa; aged 6-12 months, supplementation for 6 months

Stunting

Study or Subgroup	log[Risk Ratio]	SE	Child complementary feeding Total	Control Total	Weight	Risk Ratio IV, Random, 95% CI
**2.1.1 Food insecure**						
Addo 2020 (1)	0.46	0.0691	654	653	4.2%	1.58 [1.38, 1.81]
Adu-Afarwuah 2016 (2)	-0.4286	0.2176	347	350	2.3%	0.65 [0.43, 1.00]
Ashorn 2015 (3)	0.1454	0.1304	214	220	3.4%	1.16 [0.90, 1.49]
Bauserman 2015 (4)	-0.0592	0.1059	35	36	3.7%	0.94 [0.77, 1.16]
Bhandari 2001 (5)	-0.0731	0.0944	87	93	3.9%	0.93 [0.77, 1.12]
Borg 2020 (6)	-0.7979	0.5788	76	38	0.5%	0.45 [0.14, 1.40]
Borg 2020 (7)	0.015	0.4733	59	39	0.8%	1.02 [0.40, 2.57]
Chipili 2022 (8)	-0.6157	0.1077	100	86	3.7%	0.54 [0.44, 0.67]
Christian 2015 (9)	-0.1201	0.0776	513	210	4.1%	0.89 [0.76, 1.03]
Christian 2015 (10)	-0.0937	0.0561	896	211	4.4%	0.91 [0.82, 1.02]
Christian 2015 (11)	0.0001	0.0629	513	211	4.3%	1.00 [0.88, 1.13]
Christian 2015 (12)	-0.0131	0.0744	503	211	4.2%	0.99 [0.85, 1.14]
Dewey 2017 (13)	-0.0332	0.0834	785	408	4.1%	0.97 [0.82, 1.14]
Dewey 2017 (14)	-0.1372	0.0839	884	408	4.1%	0.87 [0.74, 1.03]
Hess 2015 (15)	-0.3487	0.0764	1623	523	4.2%	0.71 [0.61, 0.82]
Humphrey 2019 (16)	-0.1711	0.1135	356	351	3.6%	0.84 [0.67, 1.05]
Hurley 2021 (17)	0.1205	0.1221	176	191	3.5%	1.13 [0.89, 1.43]
Huybregts 2012 (18)	-0.129	0.0789	386	284	4.1%	0.88 [0.75, 1.03]
Khan 2020 (19)	-0.0965	0.1144	105	120	3.6%	0.91 [0.73, 1.14]
Luby 2018 (20)	-0.2216	0.0736	516	1003	4.2%	0.80 [0.69, 0.93]
Maleta 2015 (21)	0.1805	0.2858	187	35	1.6%	1.20 [0.68, 2.10]
Maleta 2015 (22)	0.0939	0.2852	195	34	1.7%	1.10 [0.63, 1.92]
Maleta 2015 (23)	0.0452	0.2901	184	35	1.6%	1.05 [0.59, 1.85]
Maleta 2015 (24)	-0.0659	0.2886	207	34	1.6%	0.94 [0.53, 1.65]
Maleta 2015 (25)	-0.277	0.1823	179	35	2.7%	0.76 [0.53, 1.08]
Mangani 2015 (26)	0.276	0.2579	193	195	1.9%	1.32 [0.79, 2.18]
Mangani 2015 (27)	0.127	0.2471	128	69	2.0%	1.14 [0.70, 1.84]
Mangani 2015 (28)	-0.0144	0.231	140	69	2.1%	0.99 [0.63, 1.55]
Null 2018 (29)	-0.0809	0.0832	496	1096	4.1%	0.92 [0.78, 1.09]
Omer 2022 (30)	-0.1744	1.1404	61	61	0.2%	0.84 [0.09, 7.85]
Prendergast 2019 (31)	-0.2231	0.2013	58	58	2.4%	0.80 [0.54, 1.19]
Samuel-2018 (32)	-0.4608	0.0583	1025	1052	4.4%	0.63 [0.56, 0.71]
Stewart 2019 (33)	0.0153	0.1684	290	305	2.9%	1.02 [0.73, 1.41]
**Subtotal (95% CI)**			**12171**	**8724**	**100.0%**	**0.91 [0.83, 1.00]**
Heterogeneity: $Tau^2 = 0.04$; $Chi^2 = 162.92$, df = 32 (P < 0.00001); $I^2 = 80\%$						
Test for overall effect: Z = 2.05 (P = 0.04)						
**2.1.2 Food secure**						
Iannoti 2017 (34)	-0.3498	0.2347	75	73	25.7%	0.70 [0.44, 1.12]
Lutter 2008 (35)	-0.27	0.1485	170	149	35.3%	0.76 [0.57, 1.02]
Zhang 2016 (36)	-0.7531	0.1171	2150	746	39.0%	0.47 [0.37, 0.59]
**Subtotal (95% CI)**			**2395**	**968**	**100.0%**	**0.62 [0.44, 0.88]**
Heterogeneity: $Tau^2 = 0.07$; $Chi^2 = 7.27$, df = 2 (P = 0.03); $I^2 = 73\%$						
Test for overall effect: Z = 2.72 (P = 0.007)						

Risk Ratio
IV, Random, 95% CI

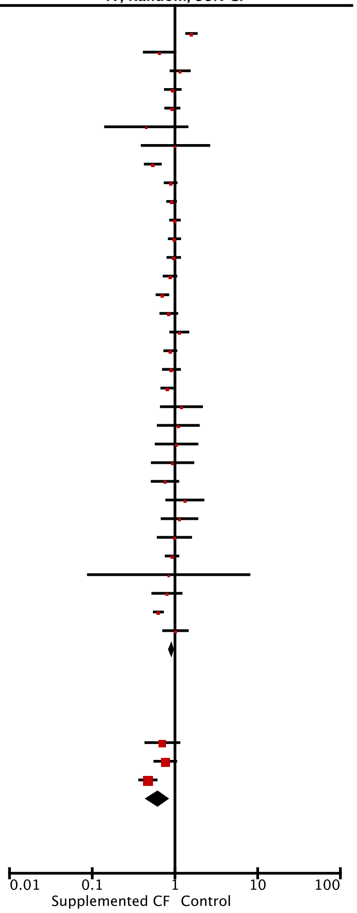

Test for subgroup differences: $Chi^2 = 4.49$, df = 1 (P = 0.03), $I^2 = 77.7\%$

Footnotes
(1) non-RCT; Africa; 6-18 months age, SQLNS supplementation for 3 months
(2) SQ-LNS; RCT; African region; mothers with >20 wk of gestation; supplementation for 12 months
(3) SQLNS; RCT; Malawi; children aged 6-18 months; supplementaion for 6 months
(4) cRCT, Africa, children aged 5 months; alternative food supplementation for 18 months
(5) cRCT, South-East Asian Region, 4 and 12 mo of age, alternative food supplementation for 6 months intervention
(6) RUSF (fish based); cRCT, Western Pacific Region, 6months age, supplementation for 6-12 months
(7) CSB++ cm; RCT, Western Pacific Region, 6months of age, supplementation for 6-12 months
(8) Fish powder; RCT; Africa; children aged 6-7 months; supplementation for 6 months, endline
(9) cRCT, chickpeas; South East Asia; aged 6-18 months; supplementation for 12 months
(10) Plumpy doz- cRCT, South-East Asian Region, age-6-18 months; supplementation for 12 months
(11) WSB++ ; cRCT, South-East Asian Region, age-6-18 months; supplementation for 12 months
(12) cRCT, rice/lentils; South East Asia; aged 6-18 months; supplementation for 12 months
(13) SQ LNS; RCT, South-East Asia; 6-24 months age, supplementation for 18 months
(14) SQ LNS; RCT, South-East Asia; mother and thier children 6-24 months age, supplementation for 18 months
(15) zinc, cRCT, Africa, aged 9-18 months of age, supplementation for 9 months
(16) SQLNS 20 gm-HIV negative, cRCT, Africa, Aged 6-18 month, supplementation for 12 months, endline
(17) Non-RCT, Africa; 6-24 months of age, SQLNS supplementation for 12 months
(18) Plumpydoz Nutriset 46 g ~247Kcal/ d; RCT; Africa; age 6-36 months, supplementation for 4 months
(19) cRCT, EMRO, age 6-24 months, commercially produced RUSF supplementation for 12 months-MQ LNS
(20) 20 g SQLNS, cRCT; Bangladesh; 6-24 months age; supplementation for 12 months
(21) 20g no milk; RCT, Africa; aged 6 months, supplementation for 12 months
(22) 40g no milk; RCT, Africa; aged 6 months, supplementation for 12 months
(23) 20g milk; RCT, Africa; aged 6 months, supplementation for 12 months
(24) 40g milk; RCT, Africa; aged 6 months, supplementation for 12 months
(25) 10g milk; RCT, Africa; aged 6 months, supplementation for 12 months
(26) CSB- RCT, Africa, age 6-18 months, suplementation for 12 month, severe stunting
(27) Soy LNS; RCT, Africa, age 6-18 months; supplementation for 12 months, severe stunting
(28) Milk LNS; RCT, Africa, age 6-18 months; supplementation for 12 months, severe stunting
(29) SQLNS-20 gm, RCT, Africa; 6-24 month aged
(30) cRCT, Ethioipia, Africa, eggs
(31) SQLNS-20 gm-HIV positive, cRCT, Africa, Aged 6-18 month, supplementation for 12 months, endline
(32) Non RCT, Africa, Rural, 6-23 months, FBF supplementation, endline
(33) RCT, Africa, 6-9.9 months age; alternative food supplementation for 6 months
(34) Eggs; RCT; Africa; children aged 6-9 months; supplementation for 6 months
(35) Non-RCT, Region of the Americas, 6-11 months age; FBF supplementation for 11 months;
(36) RCT; Western Pacific; aged 6-23 months; FBF supplementation for 2 years, endline

WHZ

Study or Subgroup	Mean Difference	SE	Child complementary feeding Total	Control Total	Weight	Mean Difference IV, Random, 95% CI
**2.4.1 Food insecure**						
Addo 2020 (1)	0.34	0.0721	654	653	2.6%	0.34 [0.20, 0.48]
Addo 2020 (2)	−0.35	0.0408	654	653	3.7%	−0.35 [−0.43, −0.27]
Adu-afarwah 2007 (3)	0.31	0.1656	97	81	0.9%	0.31 [−0.01, 0.63]
Adu-Afarwuah 2016 (4)	−0.2	0.102	323	323	1.8%	−0.20 [−0.40, −0.00]
Akalu 2010 (5)	0.34	0.1507	61	65	1.0%	0.34 [0.04, 0.64]
Akalu 2010 (6)	0.03	0.1338	106	105	1.2%	0.03 [−0.23, 0.29]
Ashorn 2015 (7)	−0.3	0.1327	195	200	1.3%	−0.30 [−0.56, −0.04]
Bauserman 2015 (8)	−0.2	0.3265	34	35	0.3%	−0.20 [−0.84, 0.44]
Becquey 2019 (9)	−0.08	0.0969	885	896	1.9%	−0.08 [−0.27, 0.11]
Borg 2020 (10)	0	0.1378	59	38	1.2%	0.00 [−0.27, 0.27]
Borg 2020 (11)	0.07	0.1173	76	39	1.5%	0.07 [−0.16, 0.30]
Christian 2015 (12)	0.02	0.0141	513	210	4.5%	0.02 [−0.01, 0.05]
Christian 2015 (13)	0	0.0153	503	211	4.5%	0.00 [−0.03, 0.03]
Christian 2015 (14)	0.04	0.0141	513	211	4.5%	0.04 [0.01, 0.07]
Christian 2015 (15)	0.04	0.0141	896	211	4.5%	0.04 [0.01, 0.07]
Dewey 2017 (16)	0.08	0.0459	785	408	3.5%	0.08 [−0.01, 0.17]
Dewey 2017 (17)	0.08	0.0408	884	408	3.7%	0.08 [0.00, 0.16]
Galasso 2019 (18)	−0.1	0.075	139	139	2.5%	−0.10 [−0.25, 0.05]
Ghosh 2020 (19)	0.01	0.0918	157	158	2.0%	0.01 [−0.17, 0.19]
Hess 2015 (20)	0.2	0.051	1623	523	3.3%	0.20 [0.10, 0.30]
Humphrey 2019 (21)	0.01	0.0816	355	350	2.3%	0.01 [−0.15, 0.17]
Hurley 2021 (22)	0.01	0.0051	176	191	4.6%	0.01 [0.00, 0.02]
Huybregts 2012 (23)	0.04	0.0714	386	284	2.6%	0.04 [−0.10, 0.18]
Huybregts 2019 (24)	0.12	0.0408	1154	1159	3.7%	0.12 [0.04, 0.20]
Luby 2018 (25)	0.17	0.0525	516	1004	3.3%	0.17 [0.07, 0.27]
Maleta 2015 (26)	0.11	0.1378	220	49	1.2%	0.11 [−0.16, 0.38]
Maleta 2015 (27)	0.14	0.148	242	48	1.1%	0.14 [−0.15, 0.43]
Maleta 2015 (28)	0.09	0.148	238	48	1.1%	0.09 [−0.20, 0.38]
Maleta 2015 (29)	−0.01	0.148	235	48	1.1%	−0.01 [−0.30, 0.28]
Maleta 2015 (30)	0.06	0.148	245	48	1.1%	0.06 [−0.23, 0.35]
Mangani 2015 (31)	−0.03	0.102	183	61	1.8%	−0.03 [−0.23, 0.17]
Mangani 2015 (32)	0.04	0.1225	188	62	1.4%	0.04 [−0.20, 0.28]
Mangani 2015 (33)	0.09	0.1225	191	62	1.4%	0.09 [−0.15, 0.33]
Muslihah 2016 (34)	0.04	0.024	59	26	4.3%	0.04 [−0.01, 0.09]
Muslihah 2016 (35)	−0.26	0.0241	58	25	4.2%	−0.26 [−0.31, −0.21]
Null 2018 (36)	0.03	0.051	496	1097	3.3%	0.03 [−0.07, 0.13]
Omer 2022 (37)	0.83	0.1524	61	61	1.0%	0.83 [0.53, 1.13]
Phu 2012 (38)	−0.1	0.1225	114	64	1.4%	−0.10 [−0.34, 0.14]
Phu 2012 (39)	0.02	0.1179	135	64	1.5%	0.02 [−0.21, 0.25]
Prendergast 2019 (40)	−0.11	0.199	59	58	0.7%	−0.11 [−0.50, 0.28]
Samuel-2018 (41)	0.07	0.0476	1172	1137	3.4%	0.07 [−0.02, 0.16]
Sazawal 2014 (42)	0.13	0.1578	59	53	1.0%	0.13 [−0.18, 0.44]
Stewart 2019 (43)	0.17	0.0816	290	305	2.3%	0.17 [0.01, 0.33]
**Subtotal (95% CI)**			**15989**	**11871**	**100.0%**	**0.03 [−0.00, 0.07]**
Heterogeneity: $Tau^2 = 0.01$; $Chi^2 = 331.77$, df = 42 (P < 0.00001); $I^2 = 87\%$						
Test for overall effect: Z = 1.83 (P = 0.07)						
**2.4.2 Food secure**						
Iannoti 2017 (44)	−0.15	0.1356	75	73	29.8%	−0.15 [−0.42, 0.12]
Lutter 2008 (45)	0.15	0.1038	170	149	44.6%	0.15 [−0.05, 0.35]
Oelofse 2003 (46)	−0.31	0.5084	16	14	2.6%	−0.31 [−1.31, 0.69]
Santos 2005 (47)	0.03	0.1583	91	81	23.0%	0.03 [−0.28, 0.34]
**Subtotal (95% CI)**			**352**	**317**	**100.0%**	**0.02 [−0.14, 0.18]**
Heterogeneity: $Tau^2 = 0.00$; $Chi^2 = 3.55$, df = 3 (P = 0.31); $I^2 = 15\%$						
Test for overall effect: Z = 0.26 (P = 0.80)						

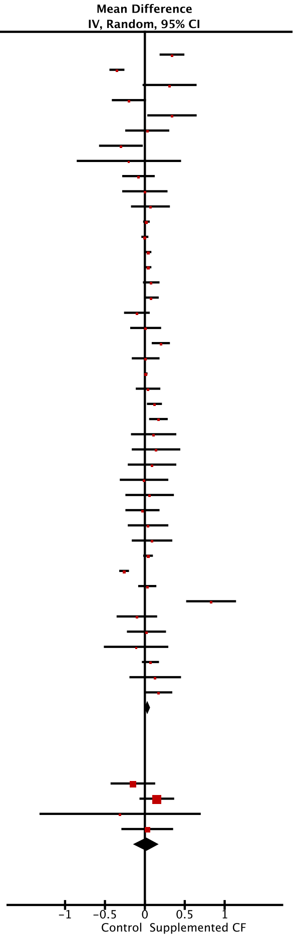

Test for subgroup differences: $Chi^2 = 0.02$, df = 1 (P = 0.90), $I^2 = 0\%$

Footnotes

(1) non-RCT; Africa; 6-18 months age, FBF supplementation for 3 months; endline WHZ
(2) non-RCT; Africa; 6-18 months age, SQLNS supplementation for 3 months
(3) SQ-LNS Nutributter; non-RCT; African region; mothers with >20 wk of gestation; supplementation for 6 months
(4) WHZ was measured at 18 months of intervention-maternal and child; SQ-LNS; RCT; African region; mothers with >20 wk of gestation; supplementation for 6 months; change in
(5) cRCT: Africa; children aged 5-17 months; FBF supplementation for 1 year; Wama Bomaya-13months;
(6) RCT: Africa; children aged 5-17 months; FBF supplementation for 1 year; Sibu-Siru-13 months
(7) SQLNS; RCT; Malawi; children aged 6-18 months; supplementaion for 6 months; change in WHZ
(8) cRCT, Africa, children aged 5 months; alternative food supplementation for18 months; endline
(9) cRCT; Burkina GFaso, SQLNS, endline
(10) CSB++; cRCT, Western Pacific Region, 6months age, supplementation for 6-12 months; change in WHZ
(11) RUSF (fish based); cRCT, Western Pacific Region, 6months age, supplementation for 6-12 months; change in WHZ
(12) WSB++ ; cRCT, South-East Asian Region, age-6-18 months; supplementation for 12 months; change in WHZ
(13) cRCT; Rice/lentils; South East Asia; aged 6-18 months; supplementation for 12 months; change in WHZ
(14) cRCT; chickpeas; South East Asia; aged 6-18 months; supplementation for 12 months; change in WHZ
(15) Plumpy doz- RCT, South-East Asian Region, age-6-18 months; supplementation for 12 months; change in WHZ
(16) SQ LNS; cRCT, South-East Asia; children 6-24 months age, supplementation for 18 months; endline
(17) SQ LNS; cRCT, South-East Asia; mothers and thier children 6-24 months age, supplementation for 18 months; endline
(18) SQLNS ; cRCT; Madasgar; Children <12 years; supplementation for 12 months; change in WHZ
(19) cRCT, Africa, 6-18 months age, FBF supplementation for intervention 6-12 months; endline WHZ
(20) zinc, cRCT, Africa, aged 9-18 months of age, supplementation for 9 months; endline
(21) SQLNS 20 gm-HIV negative, RCT, Africa, Aged 6-18 month, supplementation for 12 months; endline
(22) non-RCT, Africa; children aged 6-24 months; SQLNS supplementation for 12 months; change in WHZ
(23) Plumpydoz Nutriset 46 g ~247Kcal/d; cRCT; Africa; age 6-36 months, supplementation for 4 months; endline WHZ
(24) cRCT; Mali, SQLNS, children 6-23 months, endline
(25) 20 g SQLNS, non-RCT; Bangladesh; 6-24 months age; supplementation for 12 months; endline
(26) 10g milk; RCT, Africa; aged 6 months, supplementation for 12 months; change in WHZ
(27) 40g milk; RCT, Africa; aged 6 months, supplementation for 12 months; change in WHZ
(28) 40g no milk; RCT, Africa; aged 6 months, supplementation for 12 months; change in WHZ
(29) 20g milk; RCT, Africa; aged 6 months, supplementation for 12 months; change in WHZ
(30) 20g no milk; RCT, Africa; aged 6 months, supplementation for 12 months; change in WHZ
(31) CSB- RCT, Africa, age 6-18 months, suplementation for 12 month; change in WHZ
(32) Soy LNS; RCT, Africa, age 6-18 months; supplementation for 12 months; change in WHZ
(33) Milk LNS; RCT, Africa, age 6-18 months; supplementation for 12 months; change in WHZ
(34) Biscuit; non-RCT: Indonesia; Children aged 6-12 months; supplementation for 6 months; at endline
(35) SQ-LNS- non-RCT: Indonesia; Children aged 6-12 months; supplementation for 6 months; at endline
(36) SQLNS-20 gm, RCT, Africa; 6-24 month aged, endline
(37) cRCT, Ethioipia, Africa, eggs
(38) fortified food complement ; RCT, Western Pacific Region, 5-29 months age; supplementation for 11 months; endline WHZ
(39) fortified flour ; RCT, Western Pacific Region, 5-29 months age; supplementation for 11 months; endline WHZ
(40) SQLNS-20 gm-HIV positive, RCT, Africa, Aged 6-18 month, supplementation for 12 months; endline
(41) Non RCT, Africa, 6-23 months age, duration NR; change in WHZ; FBF supplementation
(42) RCT: India; children aged 6-24 months; FBF supplementation for 6 months; change in WHZ
(43) RCT; Africa, 6 months, egg, WHZ at 6 months
(44) Eggs; RCT; Africa; children aged 6-9 months; supplementation for 6 months; endline
(45) Non-RCT, Region of the Americas, 6-11 months age; FBF supplementation for 11 months
(46) RCT: South Africa; children aged 6-12 months; FBF supplementation for 6 months; endline
(47) milk; non-RCT: Brazil; children aged 6-18 months; change in WHZ

Wasting

Study or Subgroup	log[Risk Ratio]	SE	Child complementary feeding Total	Control Total	Weight	Risk Ratio IV, Random, 95% CI
**2.2.1 Food insecure**						
Addo 2020 (1)	0.2546	0.1615	654	653	4.3%	1.29 [0.94, 1.77]
Adu-Afarwuah 2016 (2)	0.0583	0.2989	347	350	1.5%	1.06 [0.59, 1.90]
Agapova 2017 (3)	0.4572	1.218	69	27	0.1%	1.58 [0.15, 17.19]
Agapova 2017 (4)	1.165	1.1106	68	27	0.1%	3.21 [0.36, 28.27]
Agapova 2017 (5)	−0.1305	0.4412	69	27	0.7%	0.88 [0.37, 2.08]
Agapova 2017 (6)	−0.2213	0.4535	68	28	0.7%	0.80 [0.33, 1.95]
Ashorn 2015 (7)	−0.1059	0.5086	214	220	0.5%	0.90 [0.33, 2.44]
Bauserman 2015 (8)	−0.2877	0.7281	35	35	0.3%	0.75 [0.18, 3.12]
Becquey 2019 (9)	−0.0052	0.1237	885	896	6.4%	0.99 [0.78, 1.27]
Bhandari 2001 (10)	0.0677	0.3304	87	93	1.2%	1.07 [0.56, 2.04]
Borg 2020 (11)	−0.2877	0.6031	59	38	0.4%	0.75 [0.23, 2.45]
Borg 2020 (12)	−0.3285	0.5605	76	39	0.4%	0.72 [0.24, 2.16]
Christian 2015 (13)	0.0677	0.1235	513	210	6.4%	1.07 [0.84, 1.36]
Christian 2015 (14)	−0.0101	0.1696	513	211	4.0%	0.99 [0.71, 1.38]
Christian 2015 (15)	−0.0408	0.1685	503	211	4.0%	0.96 [0.69, 1.34]
Christian 2015 (16)	−0.1625	0.161	896	211	4.3%	0.85 [0.62, 1.17]
Dewey 2017 (17)	−0.0943	0.1266	785	408	6.2%	0.91 [0.71, 1.17]
Dewey 2017 (18)	−0.1508	0.1198	884	408	6.7%	0.86 [0.68, 1.09]
Hess 2015 (19)	−0.4943	0.1552	1623	523	4.6%	0.61 [0.45, 0.83]
Humphrey 2019 (20)	−0.0101	0.4375	355	350	0.7%	0.99 [0.42, 2.33]
Hurley 2021 (21)	−0.7765	1.2461	165	152	0.1%	0.46 [0.04, 5.29]
Huybregts 2012 (22)	0.0198	0.0812	529	389	10.8%	1.02 [0.87, 1.20]
Huybregts 2019 (23)	−0.0943	0.0987	1154	1159	8.7%	0.91 [0.75, 1.10]
Isanaka 2010 (24)	−0.4005	0.2383	284	315	2.2%	0.67 [0.42, 1.07]
Khan 2020 (25)	−0.2485	0.2694	105	120	1.8%	0.78 [0.46, 1.32]
Langendorf 2014 (26)	−0.2742	0.3463	195	67	1.1%	0.76 [0.39, 1.50]
Langendorf 2014 (27)	−0.5625	0.3414	277	67	1.1%	0.57 [0.29, 1.11]
Langendorf 2014 (28)	−0.5621	0.2069	195	67	2.8%	0.57 [0.38, 0.86]
Langendorf 2014 (29)	−0.5276	0.1983	278	68	3.0%	0.59 [0.40, 0.87]
Langendorf 2014 (30)	−0.6539	0.202	338	67	2.9%	0.52 [0.35, 0.77]
Luby 2018 (31)	−0.1966	0.1674	516	1004	4.0%	0.82 [0.59, 1.14]
Maleta 2015 (32)	−0.0408	0.5296	265	51	0.5%	0.96 [0.34, 2.71]
Maleta 2015 (33)	−0.6931	0.4937	266	51	0.6%	0.50 [0.19, 1.32]
Maleta 2015 (34)	−0.5447	0.4946	266	51	0.6%	0.58 [0.22, 1.53]
Maleta 2015 (35)	0.2151	0.5172	252	52	0.5%	1.24 [0.45, 3.42]
Maleta 2015 (36)	−0.0408	0.4728	256	51	0.6%	0.96 [0.38, 2.43]
Mangani 2015 (37)	−1.8326	1.4146	191	61	0.1%	0.16 [0.01, 2.56]
Mangani 2015 (38)	0.384	1.1477	188	62	0.1%	1.47 [0.15, 13.92]
Mangani 2015 (39)	0.4253	0.9043	180	61	0.2%	1.53 [0.26, 9.00]
Null 2018 (40)	−0.1863	0.4706	496	1097	0.6%	0.83 [0.33, 2.09]
Omer 2022 (41)	−1.0986	1.1404	61	61	0.1%	0.33 [0.04, 3.12]
Prendergast 2019 (42)	−0.0202	0.9928	59	58	0.1%	0.98 [0.14, 6.86]
Samuel-2018 (43)	−0.3011	0.18	1025	1052	3.6%	0.74 [0.52, 1.05]
Stewart 2019 (44)	0.5596	0.5053	290	305	0.5%	1.75 [0.65, 4.71]
**Subtotal (95% CI)**			**16534**	**11453**	**100.0%**	**0.87 [0.81, 0.93]**
Heterogeneity: $Tau^2 = 0.01$; $Chi^2 = 48.75$, df = 43 (P = 0.25); $I^2 = 12\%$						
Test for overall effect: Z = 3.77 (P = 0.0002)						
**2.2.2 Food secure**						
Lutter 2008 (45)	0.967	1.6195	170	149	100.0%	2.63 [0.11, 62.88]
**Subtotal (95% CI)**			**170**	**149**	**100.0%**	**2.63 [0.11, 62.88]**
Heterogeneity: Not applicable						
Test for overall effect: Z = 0.60 (P = 0.55)						

Risk Ratio
IV, Random, 95% CI

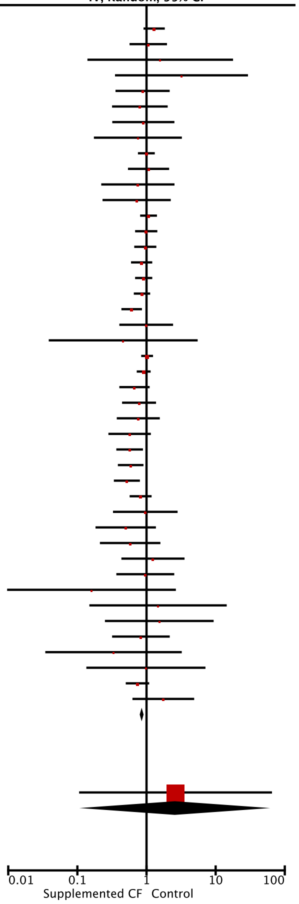

Test for subgroup differences: $Chi^2 = 0.47$, df = 1 (P = 0.49), $I^2 = 0\%$

Footnotes
(1) non-RCT; Africa; 6-18 months age, supplementation for 3 months; prevalence of wasting
(2) SQ-LNS; RCT; African region; mothers with >20 wk of gestation; maternal (during pregnancy) and child supplementation for 6 months; prevalence of wasting
(3) RCT, cowpea, Africa, age 12-23 months, suplementation for 24 month, SAM
(4) RCT, common beans, Africa, age 12-23 months, suplementation for 24 month; SAM
(5) RCT, cowpea, Africa, age 12-23 months, suplementation for 24 month, incidence of MAM
(6) RCT, common beans, Africa, age 12-23 months, suplementation for 24 month; incidence of MAM
(7) SQLNS; RCT; Malawi; children aged 6-18 months; maternal (during pregnancy) and child supplementaion for 6 months; prevalence of wasting
(8) cRCT, Africa, children aged 5 months; alternative food supplementation for18 months; prevalence of wasting
(9) SQLNS 20gm; cRCT: Africa; children aged 0-17 months; prevalence of wasting
(10) RCT, South-East Asian Region, 4 and 12 mo of age, alternative food supplementation for 6 months intervention; prevalence of wasting
(11) CSB++ cm; RCT, Western Pacific Region, 6months of age, supplementation for 6-12 months; prevalence of wasting
(12) cRCT, RUSF (fish based); Western Pacific Region, 6months age, supplementation for 6-12 months; prevalence of wasting
(13) WSB++ ; RCT, South-East Asian Region, age-6-18 months; supplementation for 12 months; prevalence of wasting
(14) cRCT; chickpeas; South East Asia; aged 6-18 months; supplementation for 12 months; prevalence of wasting
(15) cRCT; rice/lentils; South East Asia; aged 6-18 months; supplementation for 12 months; prevalence of wasting
(16) cRCT; Plumpy doz, South-East Asian Region, age-6-18 months; supplementation for 12 months; prevalence of wasting
(17) SQ LNS; cRCT, South-East Asia; 6-24 months age, supplementation for 18 months; prevalence of wasting
(18) SQ LNS; cRCT, South-East Asia; 6-24 months age, maternal (during pregnancy) and child supplementation for 18 months; prevalence of wasting
(19) zinc; cRCT, Africa, aged 9-18 months of age, supplementation for 9 months; prevalence of wasting
(20) cRCT, SQLNS 20 gm-HIV negative, Africa, Aged 6-18 month, supplementation for 12 months; prevalence of wasting
(21) Non-RCT, Africa; 6-24 months of age, Africa; supplementation for 12 months; prevalence of wasting
(22) cRCT; Plumpydoz Nutriset 46 g ~247Kcal/ d; Africa; age 6-36 months, supplementation for 4 months; incidence of wasting
(23) cRCT; Africa; age 6-23 months, supplementation for 12 months; prevalence of wasting
(24) cRCT; Africa; RUTF (500 kcal/day); children 6-60 months; incidence of wasting
(25) cRCT, EMRO, age 6-24 months,MQLNS supplementation for 12 months; prevalence of wasting
(26) cRCT; SAM; HQ-LNS (500 kcal/day-5 months), RCT; Africa, 6-23 months age, supplementation for < 6 months,incidence of wasting
(27) cRCT; SAM; MQ-LNS (250 kcal/day-5 months), RCT; Africa, 6-23 months age, supplementation for < 6 months, incidence of wasting
(28) cRCT; MAM; HQ-LNS (250 kcal/day-5 months), Africa, 6-23 months age, supplementation for < 6 months, incidence of wasting
(29) cRCT; MAM; MQ-LNS (250 kcal/day-5 months), Africa, 6-23 months age, supplementation for < 6 months, incidence of wasting
(30) SuperCereal (SC)-850 kcal; cRCT, Africa, 6-23 months age, supplementation for < 6 months, incidence of wasting
(31) 20 g SQLNS, cRCT; Bangladesh; 6-24 months age; supplementation for 12 months; prevalence of wasting
(32) 20g no milk; RCT, Africa; aged 6 months, supplementation for 12 months; incidence of wasting
(33) 40g no milk; RCT, Africa; aged 6 months, supplementation for 12 months; incidence of wasting
(34) 40g milk; RCT, Africa; aged 6 months, supplementation for 12 months; incidence of wasting
(35) 10g milk; RCT, Africa; aged 6 months, supplementation for 12 months; incidence of wasting
(36) 20g milk; RCT, Africa; aged 6 months, supplementation for 12 months; incidence of wasting
(37) Milk LNS; RCT, Africa, age 6-18 months; supplementation for 12 months; prevalence of severe wasting
(38) Soy LNS; RCT, Africa, age 6-18 months; supplementation for 12 months; prevalence of severe wasting
(39) RCT; CSB-T, Africa, age 6-18 months, suplementation for 12 month; prevalence of severe wasting
(40) SQLNS-20 gm, cRCT, Africa; 6-24 month aged, duration NR; prevalence of wasting
(41) cRCT, Ethioipia, Africa, eggs
(42) SQLNS-20 gm-HIV positive, cRCT, Africa, Aged 6-18 month, supplementation for 12 months; prevalence of wasting
(43) Non RCT, Africa, Rural, 6-23 months age; prevalence of wasting; FBF supplementation
(44) RCT, Africa, 6-9.9 months age; alernative food supplementation for 6 months; prevalence of wasting
(45) Non-RCT, Region of the Americas, 6-11 months age; FBF supplementation for 11 months; prevalence of wasting

WAZ

Study or Subgroup	Mean Difference	SE	Child complementary feeding Total	Control Total	Weight	Mean Difference IV, Random, 95% CI
**2.5.1 Food insecure**						
Addo 2020 (1)	−0.59	0.0561	654	653	3.2%	−0.59 [−0.70, −0.48]
Adu-afarwah 2007 (2)	0.34	0.1656	97	81	1.5%	0.34 [0.02, 0.66]
Adu-Afarwuah 2016 (3)	−0.14	0.0867	334	339	2.7%	−0.14 [−0.31, 0.03]
Akalu 2010 (4)	−0.26	0.1658	61	65	1.5%	−0.26 [−0.58, 0.06]
Akalu 2010 (5)	0.2	0.126	106	105	2.0%	0.20 [−0.05, 0.45]
Ashorn 2015 (6)	−0.38	0.1122	195	200	2.2%	−0.38 [−0.60, −0.16]
Bauserman 2015 (7)	−0.2	0.2857	35	36	0.7%	−0.20 [−0.76, 0.36]
Borg 2020 (8)	0.09	0.1071	59	38	2.3%	0.09 [−0.12, 0.30]
Borg 2020 (9)	0.13	0.0918	76	39	2.6%	0.13 [−0.05, 0.31]
Chipili 2022 (10)	1.05	0.0987	100	86	2.5%	1.05 [0.86, 1.24]
Christian 2015 (11)	0.03	0.0153	896	210	3.7%	0.03 [0.00, 0.06]
Christian 2015 (12)	0.02	0.0102	513	211	3.7%	0.02 [0.00, 0.04]
Christian 2015 (13)	0.02	0.0153	503	211	3.7%	0.02 [−0.01, 0.05]
Christian 2015 (14)	0.04	0.0153	513	211	3.7%	0.04 [0.01, 0.07]
Dewey 2017 (15)	0.11	0.0453	884	408	3.4%	0.11 [0.02, 0.20]
Dewey 2017 (16)	0.1	0.0459	785	408	3.3%	0.10 [0.01, 0.19]
Galasso 2019 (17)	−0.035	0.0796	139	139	2.8%	−0.04 [−0.19, 0.12]
Ghosh 2020 (18)	0.06	0.0714	157	158	2.9%	0.06 [−0.08, 0.20]
Hess 2015 (19)	0.28	0.0561	1623	523	3.2%	0.28 [0.17, 0.39]
Humphrey 2019 (20)	0.06	0.0765	355	350	2.9%	0.06 [−0.09, 0.21]
Iannotti 2014 (21)	0.05	0.1313	150	72	2.0%	0.05 [−0.21, 0.31]
Iannotti 2014 (22)	0.07	0.1476	126	72	1.7%	0.07 [−0.22, 0.36]
Luby 2018 (23)	0.25	0.0559	521	1019	3.2%	0.25 [0.14, 0.36]
Maleta 2015 (24)	−0.04	0.2755	238	48	0.8%	−0.04 [−0.58, 0.50]
Maleta 2015 (25)	0	0.2755	242	48	0.8%	0.00 [−0.54, 0.54]
Maleta 2015 (26)	0.01	0.2755	220	49	0.8%	0.01 [−0.53, 0.55]
Maleta 2015 (27)	−0.12	0.2755	235	48	0.8%	−0.12 [−0.66, 0.42]
Maleta 2015 (28)	−0.04	0.2755	245	48	0.8%	−0.04 [−0.58, 0.50]
Mangani 2015 (29)	−0.07	0.0816	183	61	2.8%	−0.07 [−0.23, 0.09]
Mangani 2015 (30)	0.09	0.0918	191	62	2.6%	0.09 [−0.09, 0.27]
Mangani 2015 (31)	0.05	0.102	188	62	2.4%	0.05 [−0.15, 0.25]
Muslihah 2016 (32)	0.25	0.0208	58	25	3.6%	0.25 [0.21, 0.29]
Muslihah 2016 (33)	0.27	0.0205	59	26	3.6%	0.27 [0.23, 0.31]
Null 2018 (34)	0.07	0.051	499	1106	3.3%	0.07 [−0.03, 0.17]
Omer 2022 (35)	0.89	0.1738	61	61	1.4%	0.89 [0.55, 1.23]
Owino 2007 (36)	0.15	0.2376	37	34	0.9%	0.15 [−0.32, 0.62]
Owino 2007 (37)	0.1	0.1986	44	35	1.2%	0.10 [−0.29, 0.49]
Phu 2012 (38)	0.12	0.1326	135	64	1.9%	0.12 [−0.14, 0.38]
Phu 2012 (39)	−0.02	0.1378	114	64	1.9%	−0.02 [−0.29, 0.25]
Prendergast 2019 (40)	0.03	0.1939	59	58	1.3%	0.03 [−0.35, 0.41]
Samuel-2018 (41)	0.3	0.0472	1172	1137	3.3%	0.30 [0.21, 0.39]
Sazawal 2014 (42)	0.2	0.14	59	53	1.8%	0.20 [−0.07, 0.47]
Stewart 2019 (43)	0.13	0.0868	290	305	2.7%	0.13 [−0.04, 0.30]
**Subtotal (95% CI)**			**13211**	**9028**	**100.0%**	**0.09 [0.04, 0.15]**
Heterogeneity: Tau² = 0.02; Chi² = 562.86, df = 42 (P < 0.00001); I² = 93%						
Test for overall effect: Z = 3.48 (P = 0.0005)						
**2.5.2 Food secure**						
Iannoti 2017 (44)	0.21	0.1577	75	73	13.7%	0.21 [−0.10, 0.52]
Lutter 2008 (45)	0.26	0.1095	170	149	28.0%	0.26 [0.05, 0.47]
Oelofse 2003 (46)	−0.03	0.4941	16	14	1.4%	−0.03 [−1.00, 0.94]
Santos 2005 (47)	0.07	0.1101	91	81	27.7%	0.07 [−0.15, 0.29]
Smuts 2019 (48)	0.06	0.148	162	100	15.6%	0.06 [−0.23, 0.35]
Smuts 2019 (49)	−0.14	0.1582	151	101	13.6%	−0.14 [−0.45, 0.17]
**Subtotal (95% CI)**			**665**	**518**	**100.0%**	**0.11 [−0.00, 0.23]**
Heterogeneity: Tau² = 0.00; Chi² = 5.10, df = 5 (P = 0.40); I² = 2%						
Test for overall effect: Z = 1.88 (P = 0.06)						

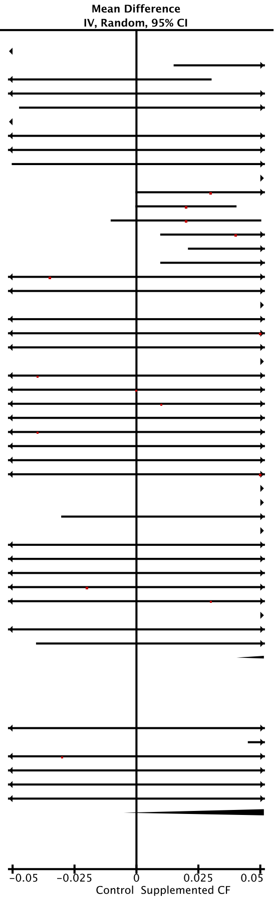

Test for subgroup differences: Chi² = 0.07, df = 1 (P = 0.79), I² = 0%

Footnotes
(1) non-RCT; Africa; 6-18 months age, SQLNS supplementation for 3 months
(2) SQ-LNS Nutributter; qRCT; African region; mothers with >20 wk of gestation; supplementation for 6 months
(3) chang in WAZ was measured at 18 months of intervention-maternal and child; SQ-LNS; RCT; African region; mothers with >20 wk of gestation; supplementation for 6 months
(4) cRCT: Africa; children aged 5-17 months; FBF supplementation for 1 year; Wama Bomaya-13 months; endline WAZ
(5) RCT: Africa; children aged 5-17 months; FBF supplementation for 1 year; Sibu-Siru-13 months;endline WAZ
(6) SQLNS; RCT; Malawi; children aged 6-18 months; supplementaion for 6 months, change in WAZ
(7) RCT, Africa, children aged 5 months; alternative food supplementation for18 months; endline
(8) CSB++; cRCT, Western Pacific Region, 6months age, supplementation for 6-12 months; change in WAZ
(9) RUSF (fish based); cRCT, Western Pacific Region, 6months age, supplementation for 6-12 months; change in WAZ
(10) Fish powder; RCT; Africa; children aged 6-7 months; supplementation for 6 months, endline
(11) Plumpy doz, cRCT, South-East Asian Region, age-6-18 months; supplementation for 12 months; change in WAZ
(12) WSB++ ; cRCT, South-East Asian Region, age-6-18 months; supplementation for 12 months; change in WAZ
(13) cRCT; rice/lentils; South East Asia; aged 6-18 months; supplementation for 12 months; change in WAZ
(14) cRCT; chickpeas; South East Asia; aged 6-18 months; supplementation for 12 months; change in WAZ
(15) SQ LNS; cRCT, South-East Asia; mothers and thier children 6-24 months age, supplementation for 18 months, endline
(16) SQ LNS; cRCT, South-East Asia; 6-24 months age, supplementation for 18 months; endline
(17) SQLNS ; RCT; Madasgar; Children <12 years; supplementation for 12 months; change in WAZ
(18) cRCT, Africa, 6-18 months age, supplementation for intervention 6-12 months; endline
(19) zinc, cRCT, Africa, aged 9-18 months of age, supplementation for 9 months; endline
(20) SQLNS 20 gm-HIV negative, cRCT, Africa, Aged 6-18 month, supplementation for 12 months, endline
(21) SQ-LNS; RCT African region; children aged 6-11 months; supplementation for 6 months, change in WAZ at 7th visit
(22) SQ-LNS; RCT African region; children aged 6-11 months; supplementation for 3 months, change in WAZ at 7th visit
(23) 20 g SQLNS, cRCT; Bangladesh; 6-24 months age; supplementation for 12 months, endline
(24) 40g no milk; RCT, Africa; aged 6 months, supplementation for 12 months, change in WAZ
(25) 40g milk; RCT, Africa; aged 6 months, supplementation for 12 months, change in WAZ
(26) 10g milk; RCT, Africa; aged 6 months, supplementation for 12 months, change in WAZ
(27) 20g milk; RCT, Africa; aged 6 months, supplementation for 12 months, change in WAZ
(28) 20g no milk; RCT, Africa; aged 6 months, supplementation for 12 months, change in WAZ
(29) CSB- RCT, Africa, age 6-18 months, suplementation for 12 month; change in WAZ
(30) Milk LNS; RCT, Africa, age 6-18 months; supplementation for 12 months; change in WAZ
(31) Soy LNS; RCT, Africa, age 6-18 months; supplementation for 12 months; change in WAZ
(32) SQ-LNS- non-RCT: Indonesia; Children aged 6-12 months; supplementation for 6 months; at endline
(33) Biscuits; non-RCT: Indonesia; Children aged 6-12 months; supplementation for 6 months; at endline
(34) SQLNS-20 gm, cRCT, Africa; 6-24 month aged, endline
(35) cRCT, Ethioipia, Africa, eggs
(36) CBM; RCT: Africa; Children aged 6 months; supplementation for 3 months, endline
(37) CBMA; RCT: Africa; Children aged 6 months; supplementation for 3 months, endline
(38) fortified flour; RCT, Western Pacific Region, 5-29 months age; supplementation for 11 months; endline
(39) Food complement ; RCT, Western Pacific Region, 5-29 months age; supplementation for 11 months; endline
(40) SQLNS-20 gm-HIV positive, cRCT, Africa, Aged 6-18 month, supplementation for 12 months, endline
(41) Non RCT, Africa, 6-23 months age, FBF supplementation; change in WAZ
(42) RCT: India; children aged 6-24 months; FBF supplementation for 6 months; change in WAZ
(43) RCT, Africa, 6-9.9 months age; alternative food supplementation for 6 months; endline
(44) Eggs; RCT; Africa; children aged 6-9 months; supplementation for 6 months; endline
(45) Non-RCT, Region of the Americas, 6-11 months age; supplementation for 11 months; endline
(46) RCT: South Africa; children aged 6-12 months; supplementation for 6 months; endline
(47) milk; non-RCT: Brazil; children aged 6-18 months; change in WAZ
(48) SQ LNS plus; RCT, Africa; aged 6-12 months, supplementation for 6 months, endline
(49) SQ LNS; RCT, Africa; aged 6-12 months, supplementation for 6 months, endline

Change in weight

Study or Subgroup	Mean Difference	SE	Child complementary feeding Total	Control Total	Weight	Mean Difference IV, Random, 95% CI
**2.6.1 Food insecure**						
Adu-Afarwuah 2016 (1)	0	0.0867	334	339	4.3%	0.00 [-0.17, 0.17]
Ashorn 2015 (2)	-0.3	0.102	195	200	4.3%	-0.30 [-0.50, -0.10]
Bhandari 2001 (3)	0.1	0.1122	87	93	4.2%	0.10 [-0.12, 0.32]
Borg 2020 (4)	0.07	0.0969	76	38	4.3%	0.07 [-0.12, 0.26]
Borg 2020 (5)	0.03	0.1071	59	39	4.2%	0.03 [-0.18, 0.24]
Christian 2015 (6)	0.02	0.0102	513	211	4.5%	0.02 [0.00, 0.04]
Christian 2015 (7)	0.02	0.0153	503	210	4.5%	0.02 [-0.01, 0.05]
Christian 2015 (8)	0.04	0.0141	513	211	4.5%	0.04 [0.01, 0.07]
Christian 2015 (9)	0.04	0.0141	896	211	4.5%	0.04 [0.01, 0.07]
Dewey 2017 (10)	-0.88	0.0102	884	408	4.5%	-0.88 [-0.90, -0.86]
Dewey 2017 (11)	0.02	0.0102	785	408	4.5%	0.02 [0.00, 0.04]
Hess 2015 (12)	0.29	0.051	1623	714	4.4%	0.29 [0.19, 0.39]
Maleta 2015 (13)	0	0.4082	238	48	2.3%	0.00 [-0.80, 0.80]
Maleta 2015 (14)	-0.1	0.4082	235	48	2.3%	-0.10 [-0.90, 0.70]
Maleta 2015 (15)	0	0.4082	242	48	2.3%	0.00 [-0.80, 0.80]
Maleta 2015 (16)	-0.1	0.4082	245	48	2.3%	-0.10 [-0.90, 0.70]
Maleta 2015 (17)	0	0.4031	220	49	2.3%	0.00 [-0.79, 0.79]
Mangani 2015 (18)	0.04	0.102	188	61	4.3%	0.04 [-0.16, 0.24]
Mangani 2015 (19)	0.11	0.0969	191	62	4.3%	0.11 [-0.08, 0.30]
Mangani 2015 (20)	-0.1	0.0862	183	62	4.3%	-0.10 [-0.27, 0.07]
Muslihah 2016 (21)	0.2234	0.236	59	26	3.4%	0.22 [-0.24, 0.69]
Muslihah 2016 (22)	1.6512	0.2729	58	25	3.1%	1.65 [1.12, 2.19]
Obatolu 2003 (23)	2.55	3.7482	15	15	0.1%	2.55 [-4.80, 9.90]
Obatolu 2003 (24)	3.91	3.761	15	15	0.1%	3.91 [-3.46, 11.28]
Phu 2012 (25)	-0.04	0.1505	114	64	4.0%	-0.04 [-0.33, 0.25]
Phu 2012 (26)	0.15	0.1457	135	64	4.0%	0.15 [-0.14, 0.44]
Samuel-2018 (27)	0.3	0.0561	1025	1052	4.4%	0.30 [0.19, 0.41]
Sazawal 2014 (28)	0.2699	0.1901	59	53	3.7%	0.27 [-0.10, 0.64]
**Subtotal (95% CI)**			**9690**	**4822**	**100.0%**	**0.06 [-0.11, 0.24]**
Heterogeneity: Tau² = 0.17; Chi² = 6381.50, df = 27 (P < 0.00001); I² = 100%						
Test for overall effect: Z = 0.72 (P = 0.47)						
**2.6.2 Food secure**						
Lutter 2008 (29)	0.22	0.1389	170	149	29.8%	0.22 [-0.05, 0.49]
Oelofse 2003 (30)	-0.2044	0.3671	16	14	4.8%	-0.20 [-0.92, 0.52]
Santos 2005 (31)	-0.0128	0.1528	91	81	25.2%	-0.01 [-0.31, 0.29]
Smuts 2019 (32)	-0.2	0.1676	151	100	21.4%	-0.20 [-0.53, 0.13]
Smuts 2019 (33)	0.1	0.1804	162	101	18.7%	0.10 [-0.25, 0.45]
**Subtotal (95% CI)**			**590**	**445**	**100.0%**	**0.03 [-0.13, 0.19]**
Heterogeneity: Tau² = 0.00; Chi² = 4.39, df = 4 (P = 0.36); I² = 9%						
Test for overall effect: Z = 0.35 (P = 0.73)						

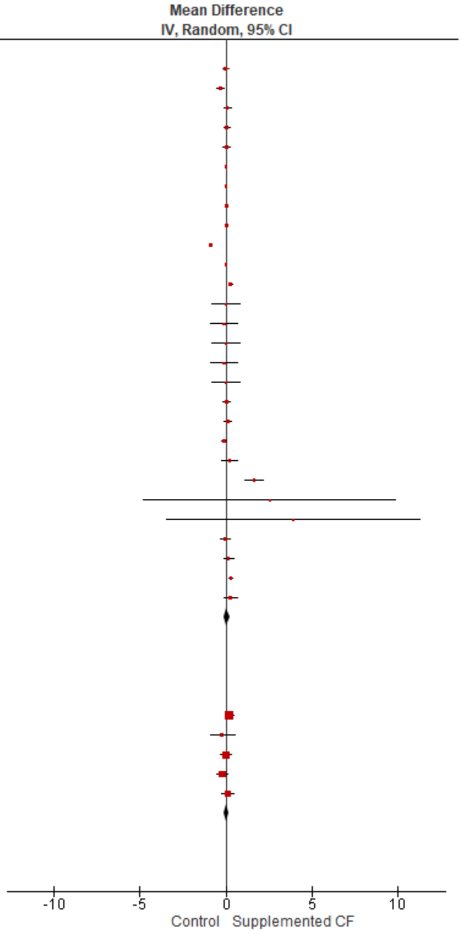

Test for subgroup differences: Chi² = 0.09, df = 1 (P = 0.77), I² = 0%

Footnotes

(1) change in weight was measured at 18 months of intervention-maternal and child; SQ-LNS; RCT; African region; mothers with >20 wk of gestation; supplementation for 6 months
(2) SQLNS; RCT; Malawi; children aged 6-18 months; supplementaion for 6 months, change in weight
(3) RCT, South-East Asian Region, 4 and 12 mo of age, alternative food supplementation for 6 months intervention; actual weight
(4) RUSF (fish based); cRCT, Western Pacific Region, 6months age, supplementation for 6-12 months; change in weight
(5) CSB++ cm; cRCT, Western Pacific Region, 6months of age, supplementation for 6-12 months, change in weight
(6) WSB++ ; cRCT, South-East Asian Region, age-6-18 months; supplementation for 12 months; change in weight
(7) rice/lentils; cRCT, South-East Asian Region, age-6-18 months; supplementation for 12 months; change in weight
(8) Chickpeas; cRCT, South-East Asian Region, age-6-18 months; supplementation for 12 months; change in weight
(9) Plumpy doz- cRCT, South-East Asian Region, age-6-18 months; supplementation for 12 months; change in weight
(10) SQLNS; cRCT, South-East Asian; 6-24 months age, weight gain in g/6mo; mother and child, endline
(11) SQLNS; cRCT, South-East Asian; 6-24 months age, weight gain in child g/6mo, endline
(12) cRCT, zinc, Africa, aged 9-18 months of age, supplementation for 9 months, actual weight at endline
(13) 40g no milk; RCT, Africa; aged 6 months, supplementation for 12 months, change in wt
(14) 20g milk; RCT, Africa; aged 6 months, supplementation for 12 months, change in wt
(15) 40g milk; RCT, Africa; aged 6 months, supplementation for 12 months, change in wt
(16) 20g no milk; RCT, Africa; aged 6 months, supplementation for 12 months, change in wt
(17) 10g milk; RCT, Africa; aged 6 months, supplementation for 12 months, change in wt
(18) Soy LNS; RCT, Africa, age 6-18 months; supplementation for 12 months; change in weight
(19) Milk; RCT, Africa, age 6-18 months; supplementation for 12 months, change in weight
(20) CSB- RCT, Africa, age 6-18 months, supplementation for 12 month; change in weight
(21) Biscuits; non-RCT: Indonesia; Children aged 6-12 months; supplementation for 6 months; at endline
(22) SQ-LNS- non-RCT: Indonesia; Children aged 6-12 months; supplementation for 6 months; at endline
(23) females 18 months-FBF
(24) males 18 months-FBF
(25) Food complement; RCT, Western Pacific Region, 5-29 months age; supplementation for 11 months, endline
(26) fortified flour; RCT, Western Pacific Region, 5-29 months age; supplementation for 11 months, endline
(27) Non RCT, Africa, 6-23 months age, FBF supplementation; actual weight at endline
(28) RCT: India; children aged 6-24 months; FBF supplementation for 6 months; change in weight
(29) Non-RCT, Region of the Americas, 6-11 months age; FBF supplementation for 11 months; weight at endline
(30) RCT: South Africa; children aged 6-12 months; FBF supplementation for 6 months; endline
(31) non-RCT: Brazil; children aged 6-18 months; change in weight; Commercially Produced Ready to Use Supplementary Food
(32) SQ LNS; RCT, Africa; aged 6-12 months, supplementation for 6 months, endline
(33) SQ LNS plus; RCT, Africa; aged 6-12 months, supplementation for 6 months, endline

Change in height

Study or Subgroup	Mean Difference	SE	Child complementary feeding Total	Control Total	Weight	Mean Difference IV, Random, 95% CI
**2.7.1 Food insecure**						
Adu-Afarwuah 2016 (1)	0.2	0.193	334	337	3.5%	0.20 [-0.18, 0.58]
Ashorn 2015 (2)	-0.7	0.2667	195	200	2.9%	-0.70 [-1.22, -0.18]
Bauserman 2015 (3)	0	0.0616	34	35	4.6%	0.00 [-0.12, 0.12]
Bhandari 2001 (4)	0.1	0.3951	87	93	2.0%	0.10 [-0.67, 0.87]
Borg 2020 (5)	-0.1	0.2516	76	38	3.0%	-0.10 [-0.59, 0.39]
Borg 2020 (6)	-0.3	0.3041	59	39	2.6%	-0.30 [-0.90, 0.30]
Christian 2015 (7)	0.09	0.0283	503	210	4.7%	0.09 [0.03, 0.15]
Christian 2015 (8)	0.08	0.0283	513	211	4.7%	0.08 [0.02, 0.14]
Christian 2015 (9)	0.06	0.0283	896	211	4.7%	0.06 [0.00, 0.12]
Christian 2015 (10)	0.01	0.0283	513	211	4.7%	0.01 [-0.05, 0.07]
Dewey 2017 (11)	0.1	0.0359	884	408	4.7%	0.10 [0.03, 0.17]
Dewey 2017 (12)	0.1	0.0366	785	408	4.7%	0.10 [0.03, 0.17]
Hess 2015 (13)	0.7	0.1663	1623	523	3.8%	0.70 [0.37, 1.03]
Maleta 2015 (14)	0	0.1861	245	48	3.6%	0.00 [-0.36, 0.36]
Maleta 2015 (15)	0.1	0.1879	235	48	3.6%	0.10 [-0.27, 0.47]
Maleta 2015 (16)	0	0.1957	242	48	3.5%	0.00 [-0.38, 0.38]
Maleta 2015 (17)	0.1	0.1919	238	48	3.5%	0.10 [-0.28, 0.48]
Maleta 2015 (18)	0.2	0.1859	222	49	3.6%	0.20 [-0.16, 0.56]
Mangani 2015 (19)	0	0.2071	188	62	3.4%	0.00 [-0.41, 0.41]
Mangani 2015 (20)	-0.1	0.242	183	62	3.1%	-0.10 [-0.57, 0.37]
Mangani 2015 (21)	0.2	0.1917	191	61	3.6%	0.20 [-0.18, 0.58]
Muslihah 2016 (22)	1.4	0.0512	58	25	4.6%	1.40 [1.30, 1.50]
Muslihah 2016 (23)	0.6	0.0504	59	26	4.6%	0.60 [0.50, 0.70]
Obatolu 2003 (24)	4.97	19.1687	30	30	0.0%	4.97 [-32.60, 42.54]
Phu 2012 (25)	0.2	0.2766	114	64	2.8%	0.20 [-0.34, 0.74]
Phu 2012 (26)	0.5	0.2775	135	64	2.8%	0.50 [-0.04, 1.04]
Samuel-2018 (27)	1.2	0.1733	1025	1052	3.7%	1.20 [0.86, 1.54]
Sazawal 2014 (28)	0.4	0.2457	59	53	3.1%	0.40 [-0.08, 0.88]
**Subtotal (95% CI)**			**9726**	**4664**	**100.0%**	**0.21 [0.07, 0.35]**
Heterogeneity: Tau² = 0.11; Chi² = 795.87, df = 27 (P < 0.00001); I² = 97%						
Test for overall effect: Z = 2.94 (P = 0.003)						
**2.7.2 Food secure**						
Lutter 2008 (29)	0.3	0.4088	170	149	23.6%	0.30 [-0.50, 1.10]
Oelofse 2003 (30)	-0.1	0.9428	16	14	4.4%	-0.10 [-1.95, 1.75]
Santos 2005 (31)	-0.23	0.4489	91	81	19.5%	-0.23 [-1.11, 0.65]
Smuts 2019 (32)	-0.1	0.3783	162	101	27.5%	-0.10 [-0.84, 0.64]
Smuts 2019 (33)	-0.5	0.3976	151	100	24.9%	-0.50 [-1.28, 0.28]
**Subtotal (95% CI)**			**590**	**445**	**100.0%**	**-0.13 [-0.52, 0.26]**
Heterogeneity: Tau² = 0.00; Chi² = 2.03, df = 4 (P = 0.73); I² = 0%						
Test for overall effect: Z = 0.66 (P = 0.51)						

Mean Difference IV, Random, 95% CI

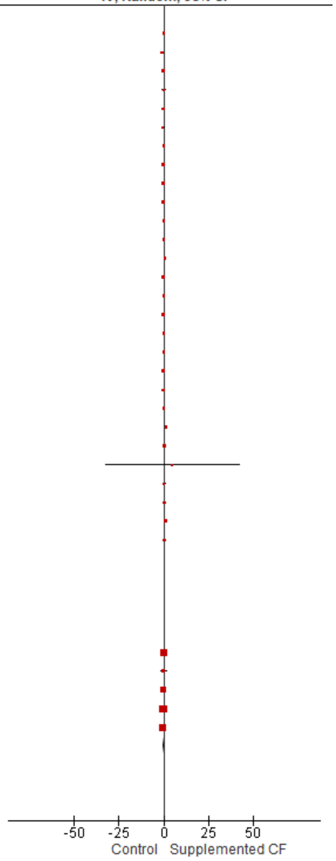

Test for subgroup differences: Chi² = 2.64, df = 1 (P = 0.10), I² = 62.2%

Footnotes
(1) change in height, maternal and child supplementation; SQ-LNS; RCT; African region; mothers with >20 wk of gestation; supplementation for 12 months
(2) RCT; Malawi; children aged 6-18 months; SQLNS supplementaion for 6 months, change in height
(3) cRCT, Africa, children aged 5 months;alternative food supplementation for18 months, endline
(4) RCT, South-East Asian Region, 4 and 12 mo of age, alternative food supplementation for 6 months
(5) RUSF (fish based); cRCT, Western Pacific Region, 6months age, supplementation for 6-12 months, change in height
(6) CSB++; cRCT, Western Pacific Region, 6months age, supplementation for 6-12 , change in height
(7) cRCT, rice/lentils;South East Asia; aged 6-18 months; supplementation for 12 months, change in height
(8) cRCT, chickpeas; South East Asia; aged 6-18 months; supplementation for 12 months, change in height
(9) Plumpy doz- cRCT; South East Asia; aged 6-18 months; supplementation for 12 months, change in height
(10) WSB++ ; cRCT, South-East Asian Region, age-6-18 months; supplementation for 12 months, change in height
(11) SQ LNS; cRCT, South-East Asia; children 6-24 months age, maternal (during pregnacy) and child supplementation for 18 months, height gain
(12) SQ LNS; cRCT, South-East Asia; 6-24 months age, child supplementation for 18 months, height gain
(13) zinc, cRCT, Africa, aged 9-18 months of age, supplementation for 9 months
(14) 20g no milk; RCT, Africa; aged 6 months, supplementation for 12 months; z scores, change in height
(15) 20g milk; RCT, Africa; aged 6 months, supplementation for 12 months; z scores, change in height
(16) 40g no milk; RCT, Africa; aged 6 months, supplementation for 12 months, change in height
(17) 40g milk; RCT, Africa; aged 6 months, supplementation for 12 months, change in height
(18) 10g milk; RCT, Africa; aged 6 months, supplementation for 12 months; z scores, change in height
(19) Soy LNS; RCT, Africa, age 6-18 months; supplementation for 12 months, change in height
(20) CSB- RCT, Africa, age 6-18 months, suplementation for 12 month, change in height
(21) Milk LNS; RCT, Africa, age 6-18 months; supplementation for 12 months, change in height
(22) SQ-LNS- non-RCT: Indonesia; Children aged 6-12 months; supplementation for 6 months; at endline
(23) biscuits- non-RCT: Indonesia; Children aged 6-12 months; supplementation for 6 months; at endline
(24) non-RCT; Africa; children aged 4 months; FBF supplementation for 5 months
(25) Food complement ; RCT, Western Pacific Region, 5-29 months age; supplementation for 11 months, endline
(26) Fortified flour; RCT, Western Pacific Region, 5-29 months age; supplementation for 11 months, endline
(27) Non RCT, Africa, Rural, 6-23 months age, endline; FBF supplementation
(28) RCT: India; children aged 6-24 months; FBF supplementation for 6 months; change in height
(29) Non-RCT, Region of the Americas, 6-11 months age; FBF supplementation for 11 months, endline
(30) RCT: South Africa; children aged 6-12 months; FBF supplementation for 6 months; endline
(31) non-RCT: Brazil; children aged 6-18 months; change in length; Commercially Produced Ready to Use Supplementary Food
(32) SQ LNS plus; RCT, Africa; aged 6-12 months, supplementation for 6 months, endline
(33) SQ LNS; RCT, Africa; aged 6-12 months, supplementation for 6 months, endline

MUAC (cm)

Study or Subgroup	Mean Difference	SE	Child complementary feeding Total	Control Total	Weight	Mean Difference IV, Random, 95% CI
**2.8.1 Food insecure**						
Adu-Afarwuah 2016 (1)	0.1	0.0765	348	353	8.3%	0.10 [-0.05, 0.25]
Akalu 2010 (2)	0.0439	0.1377	106	105	4.2%	0.04 [-0.23, 0.31]
Becquey 2019 (3)	0	0.0867	885	896	7.4%	0.00 [-0.17, 0.17]
Borg 2020 (4)	0.2	0.1378	59	38	4.2%	0.20 [-0.07, 0.47]
Borg 2020 (5)	0.1	0.1378	76	39	4.2%	0.10 [-0.17, 0.37]
Hess 2015 (6)	0.3	0.0561	1623	523	10.4%	0.30 [0.19, 0.41]
Hurley 2021 (7)	0.018	0.0042	176	191	14.8%	0.02 [0.01, 0.03]
Huybregts 2012 (8)	0.2	0.0918	386	284	7.0%	0.20 [0.02, 0.38]
Huybregts 2019 (9)	0.1	0.0459	1154	1159	11.6%	0.10 [0.01, 0.19]
Maleta 2015 (10)	0.1	0.1429	238	48	4.0%	0.10 [-0.18, 0.38]
Maleta 2015 (11)	0.1	0.1429	220	49	4.0%	0.10 [-0.18, 0.38]
Maleta 2015 (12)	0.2	0.1429	245	48	4.0%	0.20 [-0.08, 0.48]
Maleta 2015 (13)	0.1	0.1429	235	48	4.0%	0.10 [-0.18, 0.38]
Maleta 2015 (14)	0.2	0.148	242	48	3.8%	0.20 [-0.09, 0.49]
Muslihah 2016 (15)	0	0.2393	58	25	1.7%	0.00 [-0.47, 0.47]
Muslihah 2016 (16)	0	0.2354	59	26	1.8%	0.00 [-0.46, 0.46]
Owino 2007 (17)	0	0.1929	44	35	2.5%	0.00 [-0.38, 0.38]
Owino 2007 (18)	0.3784	0.2055	37	34	2.2%	0.38 [-0.02, 0.78]
**Subtotal (95% CI)**			**6191**	**3949**	**100.0%**	**0.12 [0.05, 0.18]**
Heterogeneity: Tau² = 0.01; Chi² = 42.15, df = 17 (P = 0.0006); I² = 60%						
Test for overall effect: Z = 3.54 (P = 0.0004)						
**2.8.2 Food secure**						
Smuts 2019 (19)	0.1	0.1627	162	101	51.7%	0.10 [-0.22, 0.42]
Smuts 2019 (20)	-0.1	0.1684	151	100	48.3%	-0.10 [-0.43, 0.23]
**Subtotal (95% CI)**			**313**	**201**	**100.0%**	**0.00 [-0.23, 0.23]**
Heterogeneity: Tau² = 0.00; Chi² = 0.73, df = 1 (P = 0.39); I² = 0%						
Test for overall effect: Z = 0.03 (P = 0.98)						

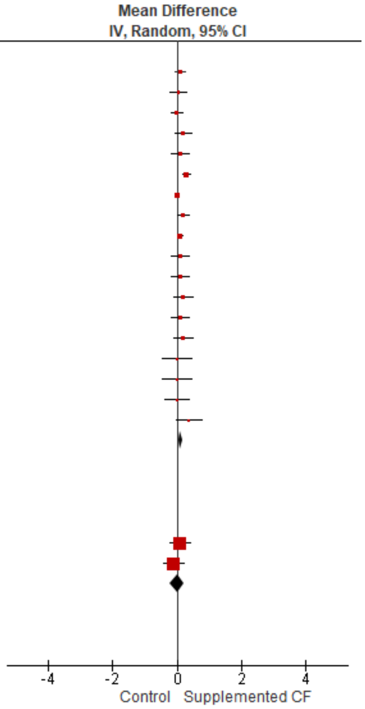

Test for subgroup differences: Chi² = 0.89, df = 1 (P = 0.35), I² = 0%

Footnotes

(1) SQ-LNS; RCT; African region; mothers with >20 wk of gestation; supplementation for 6 months, endline
(2) RCT: Africa; children aged 5-17 months; FBF supplementation for 1 year; Sibu Siri; endline MUAC
(3) SQLNS 20gm; cRCT: Africa; children aged 0-17 months; endline
(4) CSB++ cm; RCT, Western Pacific Region, 6months of age, supplementation for 6-12 months, Change in MUAC
(5) RUSF (fish based); cRCT, Western Pacific Region, 6months age, supplementation for 6-12 months, endline MUAC
(6) zinc, cRCT; Africa, aged 9-18 months of age, supplementation for 9 months, acutal MUAC at endline
(7) non-RCT, Africa; children 6-24 months of age, SQLNS supplementation for 6 months; change in MUAC
(8) Plumpydoz Nutriset 46 g ~247Kcal/ d; cRCT, Africa; aged 6-36 months, supplementation for 4months; endline MUAC
(9) cRCT; Africa; age 6-23 months, SQLNS supplementation for 12 months, endline
(10) 40g no milk; RCT, Africa; aged 6 months, supplementation for 12 months; change in MUAC
(11) 10g milk; RCT, Africa; aged 6 months, supplementation for 12 months; death during 6-12 months of age; change in MUAC
(12) 20g no milk; RCT, Africa; aged 6 months, supplementation for 12 months; death during 6-12 months of age; change in MUAC
(13) 20g milk; RCT, Africa; aged 6 months, SQLNS supplementation for 12 months; death during 6-12 months of age; change in MUAC
(14) 40g milk; RCT, Africa; aged 6 months, SQLNS supplementation for 12 months; change in MUAC
(15) SQ-LNS- non-RCT: Indonesia; Children aged 6-12 months; supplementation for 6 months; at endline
(16) biscuits- non-RCT: Indonesia; Children aged 6-12 months; supplementation for 6 months; at endline
(17) CBMA; RCT: Africa; Children aged 6 months; supplementation for 3 months, endline
(18) CBM; RCT: Africa; Children aged 6 months; supplementation for 3 months, endline
(19) SQ LNS plus; RCT, Africa; aged 6-12 months, supplementation for 6 months, endline
(20) SQ-LNS; RCT, Africa, aged 6-12 months , supplementation for 12 months, endline

Anemia

Study or Subgroup	log[Risk Ratio]	SE	Child complementary feeding Total	Control Total	Weight	Risk Ratio IV, Random, 95% CI
**2.10.1 Food insecure**						
Addo 2020 (1)	0.3934	0.0469	654	653	10.9%	1.48 [1.35, 1.62]
Bauserman 2015 (2)	−0.6549	0.2294	34	28	4.5%	0.52 [0.33, 0.81]
Dewey 2017 (3)	−0.362	0.1401	257	136	7.3%	0.70 [0.53, 0.92]
Dewey 2017 (4)	−0.3426	0.1445	293	136	7.1%	0.71 [0.53, 0.94]
Glasso 2019 (5)	−0.6391	0.5165	18	19	1.3%	0.53 [0.19, 1.45]
Hess 2015 (6)	−0.1406	0.0207	1307	443	11.4%	0.87 [0.83, 0.90]
Humphrey 2019 (7)	−0.4044	0.2163	353	346	4.8%	0.67 [0.44, 1.02]
Huybregts 2012 (8)	−0.1657	0.0631	370	264	10.3%	0.85 [0.75, 0.96]
Khan 2020 (9)	−0.0298	0.0222	268	267	11.4%	0.97 [0.93, 1.01]
Owino 2007 (10)	−0.1235	0.1381	44	35	7.3%	0.88 [0.67, 1.16]
Owino 2007 (11)	−0.2072	0.1597	37	34	6.5%	0.81 [0.59, 1.11]
Prendergast 2019 (12)	−0.8824	0.6641	58	56	0.8%	0.41 [0.11, 1.52]
Samuel-2018 (13)	−0.1732	0.0724	1025	1052	10.0%	0.84 [0.73, 0.97]
Siega-Riz 2014 (14)	−0.0521	0.1634	74	61	6.4%	0.95 [0.69, 1.31]
**Subtotal (95% CI)**			**4792**	**3530**	**100.0%**	**0.86 [0.76, 0.97]**
Heterogeneity: $Tau^2 = 0.03$; $Chi^2 = 137.15$, df = 13 (P < 0.00001); $I^2 = 91\%$						
Test for overall effect: Z = 2.45 (P = 0.01)						
**2.10.2 Food secure**						
Lutter 2008 (15)	−0.4714	0.1544	170	149	3.6%	0.62 [0.46, 0.84]
Smuts 2019 (16)	−0.3143	0.1921	162	101	2.3%	0.73 [0.50, 1.06]
Smuts 2019 (17)	−0.4121	0.1973	151	100	2.2%	0.66 [0.45, 0.97]
Zhang 2016 (18)	−0.455	0.0306	2174	754	91.9%	0.63 [0.60, 0.67]
**Subtotal (95% CI)**			**2657**	**1104**	**100.0%**	**0.64 [0.60, 0.67]**
Heterogeneity: $Tau^2 = 0.00$; $Chi^2 = 0.58$, df = 3 (P = 0.90); $I^2 = 0\%$						
Test for overall effect: Z = 15.39 (P < 0.00001)						

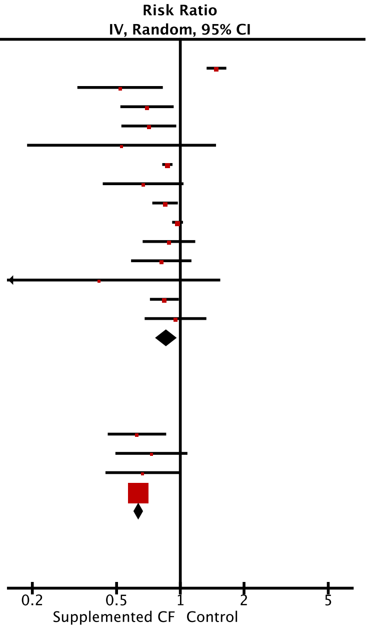

Test for subgroup differences: $Chi^2 = 19.13$, df = 1 (P < 0.0001), $I^2 = 94.8\%$

Footnotes

(1) Non RCT, Africa, 6-18 months age,SQLNS supplementation for 3 months
(2) cRCT, Africa, aged 5 months; Alternative food supplementation for 18 months; Hb <= 10g/dl
(3) SQ LNS; cRCT, South-East Asia; 6-24 months age, supplementation for 18 months, Hb <110g/l
(4) SQ LNS; cRCT, South-East Asia; 6-24 months age,maternal (during pregnancy) and child supplementation for 18 months; Hb <110g/l
(5) RCT; Madasgar; Children <12 years; supplementation for 12 months; Hb <11g/dl
(6) zinc, cRCT, Africa, aged 9-18 months of age, supplementation for 9 months, Hb <110g/l
(7) SQLNS 20 gm-HIV negative- cRCT, Africa, Aged 6-18 month, supplementation for 12 months, Hb <105 g/l
(8) Plumpydoz Nutriset 46 g ~247Kcal/ d; RCT, Africa, aged 6-36 months, supplementation for 4 months, endline
(9) MQ-LNS; RCT, EMRO, age 6-24 months, supplementation for 12 months; anemia
(10) CBMA; RCT: Africa; Children aged 6 months; supplementation for intervention 3 months, <11g/dl, endline
(11) CBM; RCT: Africa; Children aged 6 months; supplementation for intervention 3 months, <11g/dl, endline
(12) SQLNS-20 gm-HIV positive- RCT, Africa, Aged 6-18 month, supplementation for 12 months, Hb <105 g/l
(13) Non RCT, Africa, Rural, 6-23 months age; FBF supplementation
(14) RCT; ntibucá, Honduras; children aged 6-18 months; supplementation for 12 months
(15) Non-RCT, Region of the Americas, 6-11 months age, FBF supplementation for 11 months, endline, Hb <110g/l
(16) SQ LNS; RCT, Africa; aged 6-12 months, supplementation for 6 months
(17) SQ LNS plus; RCT, Africa; aged 6-12 months, supplementation for 6 months
(18) RCT; Western Pacific; children aged 6-24 months; FBF supplementation for intervention 2 years, endline

Iron deficiency anemia

Study or Subgroup	log[Risk Ratio]	SE	Weight	Risk Ratio IV, Random, 95% CI
**2.9.1 Food insecure**				
Addo 2020 (1)	0.0032	0.0797	25.8%	1.00 [0.86, 1.17]
Dewey 2017 (2)	-0.6111	0.1985	21.2%	0.54 [0.37, 0.80]
Dewey 2017 (3)	-0.8455	0.2031	21.0%	0.43 [0.29, 0.64]
Glasso 2019 (4)	-0.6391	0.6259	7.3%	0.53 [0.15, 1.80]
Samuel-2018 (5)	0.2097	0.1134	24.7%	1.23 [0.99, 1.54]
**Subtotal (95% CI)**			**100.0%**	**0.74 [0.50, 1.09]**
Heterogeneity: Tau² = 0.15; Chi² = 29.76, df = 4 (P < 0.00001); I² = 87%				
Test for overall effect: Z = 1.52 (P = 0.13)				
**2.9.2 Food secure**				
Smuts 2019 (6)	-1.3297	0.3776	55.0%	0.26 [0.13, 0.55]
Smuts 2019 (7)	-1.5008	0.4171	45.0%	0.22 [0.10, 0.50]
**Subtotal (95% CI)**			**100.0%**	**0.24 [0.14, 0.42]**
Heterogeneity: Tau² = 0.00; Chi² = 0.09, df = 1 (P = 0.76); I² = 0%				
Test for overall effect: Z = 5.03 (P < 0.00001)				

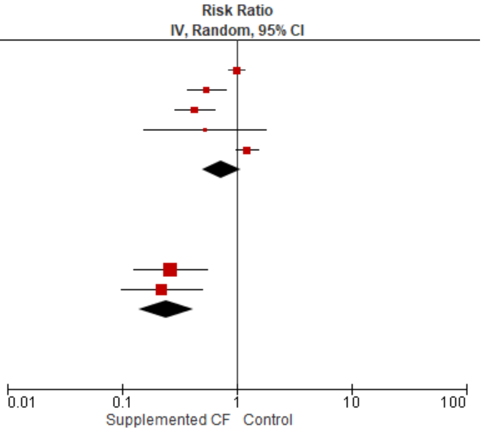

Test for subgroup differences: Chi² = 10.38, df = 1 (P = 0.001), I² = 90.4%

Footnotes
(1) Non RCT, Africa 6-18 months age, SQLNS supplementation for 3 months
(2) SQ LNS; cRCT, South-East Asia; 6-24 months age, supplementation for 18 months, Hb <110g/l
(3) SQ LNS; cRCT, South-East Asia; 6-24 months age,maternal (during pregnancy) and child supplementation for 18 months; Hb <110g/l
(4) RCT; Madasgar; Children <12 years; FBF supplementation for 12 months, anaemia and iron deficient
(5) Non RCT, Africa, Rural, 6-23 months age; FBF supplementation
(6) SQ LNS plus; RCT, Africa; aged 6-12 months, supplementation for 6 months
(7) SQ LNS; RCT, Africa; aged 6-12 months, supplementation for 6 months

Hb levels (g/dl)

Study or Subgroup	Std. Mean Difference	SE	Child complementary feeding Total	Control Total	Weight	Std. Mean Difference IV, Random, 95% CI
**2.11.1 Food insecure**						
Addo 2020 (1)	−1	0.0042	654	653	6.7%	−1.00 [−1.01, −0.99]
Bauserman 2015 (2)	0.3524	0.1705	34	28	6.5%	0.35 [0.02, 0.69]
Dewey 2017 (3)	0.3702	0.0849	293	136	6.7%	0.37 [0.20, 0.54]
Dewey 2017 (4)	0.2489	0.0873	257	136	6.7%	0.25 [0.08, 0.42]
Galasso 2019 (5)	4	1.1348	18	19	3.0%	4.00 [1.78, 6.22]
Hess 2015 (6)	0.5896	0.0559	1307	443	6.7%	0.59 [0.48, 0.70]
Humphrey 2019 (7)	0.1687	0.0758	353	346	6.7%	0.17 [0.02, 0.32]
Huybregts 2012 (8)	0.2245	0.0808	370	264	6.7%	0.22 [0.07, 0.38]
Owino 2007 (9)	0.7	0.2674	37	34	6.3%	0.70 [0.18, 1.22]
Owino 2007 (10)	0.6	0.2876	44	35	6.2%	0.60 [0.04, 1.16]
Prendergast 2019 (11)	0.1575	0.1876	58	56	6.5%	0.16 [−0.21, 0.53]
Samuel–2018 (12)	0.06	0.4008	1025	1052	5.8%	0.06 [−0.73, 0.85]
Sazawal 2014 (13)	1.06	0.272	66	48	6.3%	1.06 [0.53, 1.59]
Siega–Riz 2014 (14)	2.7119	0.2408	74	61	6.4%	2.71 [2.24, 3.18]
**Subtotal (95% CI)**			**4590**	**3311**	**87.2%**	**0.59 [0.04, 1.15]**
Heterogeneity: Tau² = 1.06; Chi² = 2197.02, df = 13 (P < 0.00001); I² = 99%						
Test for overall effect: Z = 2.08 (P = 0.04)						
**2.11.2 Food secure**						
Lutter 2008 (15)	0.513	0.1141	170	149	6.6%	0.51 [0.29, 0.74]
Oelofse 2003 (16)	0.2	0.4139	16	14	5.8%	0.20 [−0.61, 1.01]
Smuts 2019 (17)	3	5.7553	151	100	0.2%	3.00 [−8.28, 14.28]
Smuts 2019 (18)	5	7.1837	162	101	0.1%	5.00 [−9.08, 19.08]
**Subtotal (95% CI)**			**499**	**364**	**12.8%**	**0.49 [0.28, 0.71]**
Heterogeneity: Tau² = 0.00; Chi² = 1.12, df = 3 (P = 0.77); I² = 0%						
Test for overall effect: Z = 4.48 (P < 0.00001)						
**Total (95% CI)**			**5089**	**3675**	**100.0%**	**0.58 [0.05, 1.10]**
Heterogeneity: Tau² = 1.06; Chi² = 2375.92, df = 17 (P < 0.00001); I² = 99%						
Test for overall effect: Z = 2.16 (P = 0.03)						
Test for subgroup differences: Chi² = 0.11, df = 1 (P = 0.74), I² = 0%						

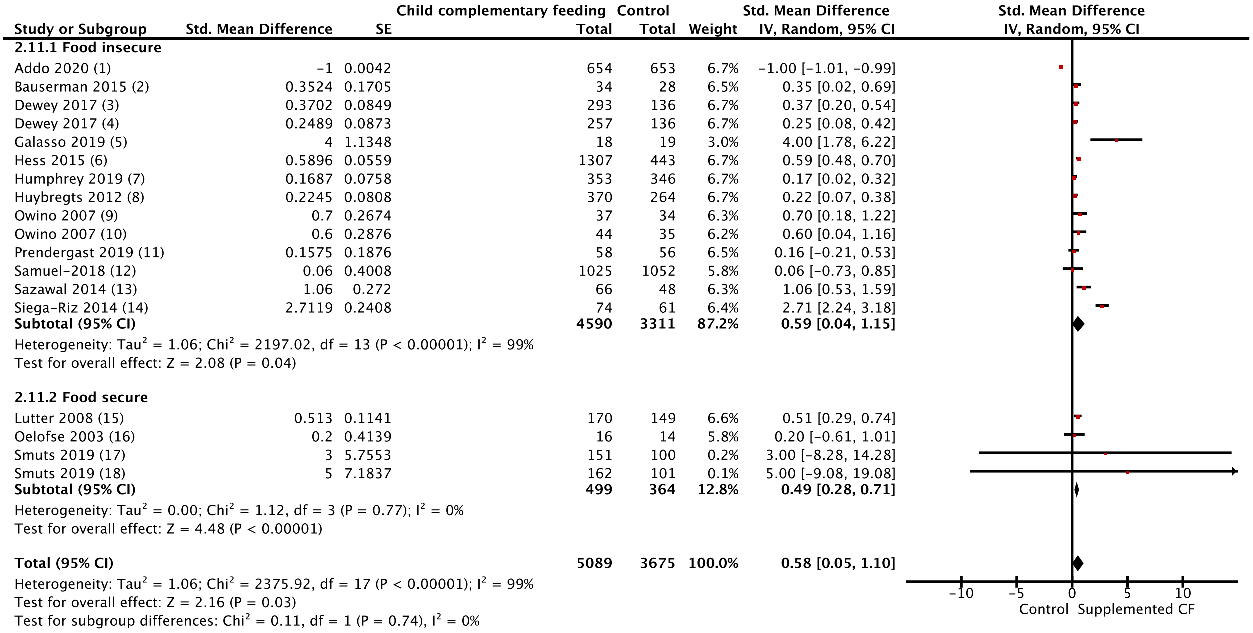

Footnotes
(1) non–RCT; Africa; 6–18 months age, SQLNS supplementation for 3 months
(2) cRCT, Africa, children aged 5 months; alternative food supplementation for18 months
(3) SQ LNS; cRCT, South–East Asia; 6–24 months age and their mothers, maternal (during pregnancy) and child supplementation for 18 months
(4) SQ LNS; cRCT, South–East Asia; 6–24 months age and their mothers, supplementation for 18 months
(5) RCT; Madasgar; Children <12 years; FBF supplementation for 12 months
(6) zinc, cRCT, Africa, aged 9–18 months of age, supplementation for 9 months
(7) SQLNS 20 gm–HIV negative– cRCT, Africa, Aged 6–18 month, supplementation for 12 months, endline
(8) Plumpydoz Nutriset 46 g ~247Kcal/ d; RCT, Africa, aged 6–36 months, supplementation for 4 months, endline
(9) CBM; RCT: Africa; Children aged 6 months; supplementation for 3 months
(10) CBMA; RCT: Africa; Children aged 6 months; supplementation for 3 months
(11) SQLNS–20 gm–HIV positive– cRCT, Africa, Aged 6–18 month, supplementation for 12 months, endline
(12) Non RCT, Africa, 6–23 months age; FBF supplementation
(13) RCT: India; children aged 6–24 months; FBF supplementation for 6 months; change in Hb levels
(14) RCT; ntibucá, Honduras; children aged 6–18 months; supplementation for 12 months
(15) Non–RCT, Region of the Americas, 6–11 months age, FBF supplementation for 11 months, endline
(16) RCT: South Africa; children aged 6–12 months; FBF supplementation for 6 months; endline
(17) SQ LNS plus; RCT, Africa; aged 6–12 months, supplementation for 6 months
(18) SQ LNS; RCT, Africa; aged 6–12 months, supplementation for 6 months

Fever

Study or Subgroup	log[Risk Ratio]	SE	Weight	Risk Ratio IV, Random, 95% CI
**2.12.1 Food insecure**				
Dewey 2017 (1)	0.034	0.1036	39.5%	1.03 [0.84, 1.27]
Dewey 2017 (2)	-0.1841	0.1303	36.2%	0.83 [0.64, 1.07]
Hurley 2021 (3)	0.5518	0.2326	24.3%	1.74 [1.10, 2.74]
**Subtotal (95% CI)**			**100.0%**	**1.08 [0.78, 1.50]**
Heterogeneity: Tau² = 0.06; Chi² = 7.69, df = 2 (P = 0.02); I² = 74%				
Test for overall effect: Z = 0.49 (P = 0.63)				
**2.12.2 Food secure**				
Iannoti 2017 (4)	0.2607	0.2649	100.0%	1.30 [0.77, 2.18]
**Subtotal (95% CI)**			**100.0%**	**1.30 [0.77, 2.18]**
Heterogeneity: Not applicable				
Test for overall effect: Z = 0.98 (P = 0.33)				

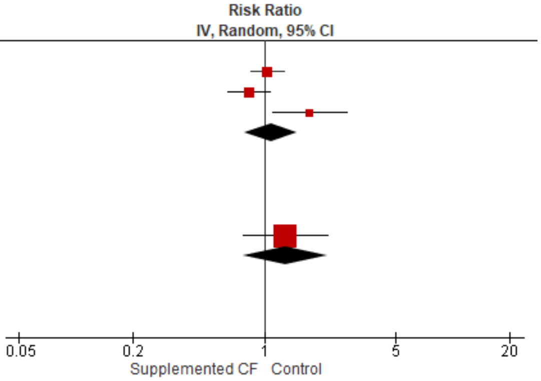

Test for subgroup differences: Chi² = 0.33, df = 1 (P = 0.56), I² = 0%

Footnotes
(1) SQLNS; cRCT, South-East Asian; 6-24 months age, supplementation for 18 months
(2) SQLNS; cRCT, South-East Asian; 6-24 months age; mother (during pregnancy) and child supplement; measured at 18 months after intervntion
(3) non-RCT, Africa; children aged 6-24 months; supplementation for 12 months, high fever
(4) Eggs; RCT; Ecuador; children aged 6-9 months; supplementation for 6 months; endline

Diarrhea

Study or Subgroup	log[Risk Ratio]	SE	Weight	Risk Ratio IV, Random, 95% CI
**2.13.1 Food insecure**				
Chipili 2022 (1)	-0.2848	0.2508	18.1%	0.75 [0.46, 1.23]
Dewey 2017 (2)	0.5539	0.3381	11.6%	1.74 [0.90, 3.38]
Dewey 2017 (3)	0.4663	0.3372	11.6%	1.59 [0.82, 3.09]
Humphrey 2019 (4)	-0.2469	0.2511	18.1%	0.78 [0.48, 1.28]
Hurley 2021 (5)	0.0958	0.1306	36.5%	1.10 [0.85, 1.42]
Prendergast 2019 (6)	-0.0171	0.6048	4.2%	0.98 [0.30, 3.22]
**Subtotal (95% CI)**			**100.0%**	**1.06 [0.82, 1.36]**
Heterogeneity: Tau² = 0.03; Chi² = 7.06, df = 5 (P = 0.22); I² = 29%				
Test for overall effect: Z = 0.44 (P = 0.66)				
**2.13.2 Food secure**				
Iannoti 2017 (7)	0.7106	0.3277	100.0%	2.04 [1.07, 3.87]
**Subtotal (95% CI)**			**100.0%**	**2.04 [1.07, 3.87]**
Heterogeneity: Not applicable				
Test for overall effect: Z = 2.17 (P = 0.03)				

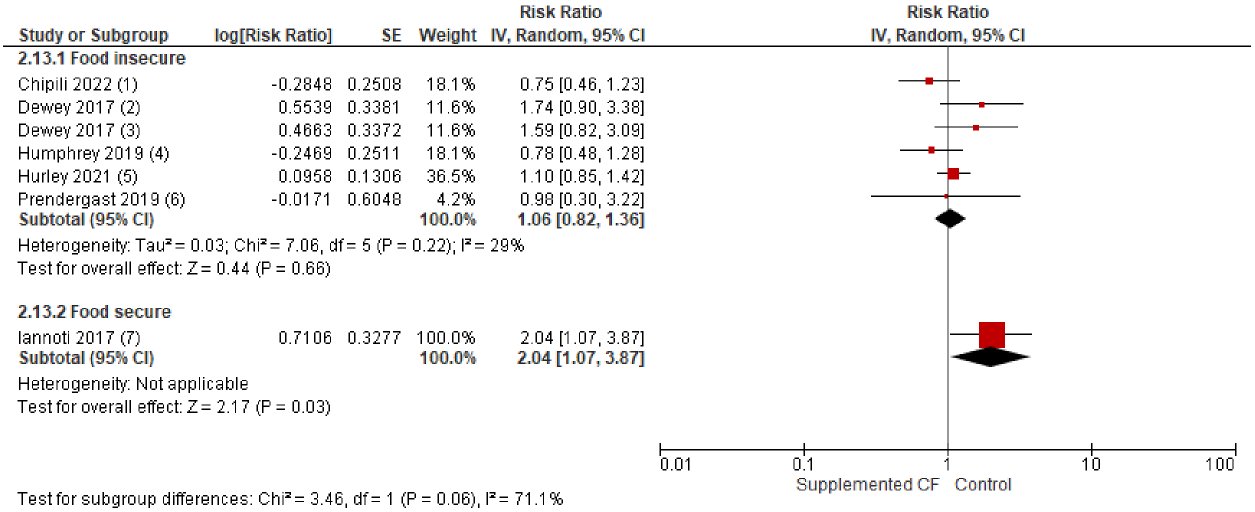

Test for subgroup differences: Chi² = 3.46, df = 1 (P = 0.06), I² = 71.1%

Footnotes
(1) Fish powder; RCT; Africa; children aged 6-7 months; supplementation for 6 months, endline
(2) SQLNS; cRCT, South-East Asian; 6-24 months age, child supplementation for 18 months
(3) SQLNS; cRCT, South-East Asian; 6-24 months age, Diarhhoea was measured at 18 months intervention to mother (during pregnancy) and child
(4) SQLNS 20 gm-HIV negative- cRCT, Africa, Aged 6-18 month, supplementation for 12 months
(5) non-RCT, Africa; children aged 6-24 months; supplementation for 12 months
(6) SQLNS-20 gm-HIV positive- cRCT, Africa, Aged 6-18 month, supplementation for 12 months
(7) Eggs; RCT; Ecuador; children aged 6-9 months; supplementation for 6 months; acute diarrhea at endline

Respiratory illness

Study or Subgroup	log[Risk Ratio]	SE	Child complementary feeding Total	Control Total	Weight	Risk Ratio IV, Random, 95% CI
**2.14.1 Food insecure**						
Chipili 2022 (1)	−0.7017	0.4129	118	117	39.8%	0.50 [0.22, 1.11]
Humphrey 2019 (2)	−0.0101	0.998	352	350	17.1%	0.99 [0.14, 7.00]
Hurley 2021 (3)	0.5926	0.3499	176	191	43.1%	1.81 [0.91, 3.59]
Prendergast 2019 (4)	0	0	59	58		Not estimable
**Subtotal (95% CI)**			**705**	**716**	**100.0%**	**0.97 [0.37, 2.58]**
Heterogeneity: $Tau^2 = 0.45$; $Chi^2 = 5.72$, df = 2 (P = 0.06); $I^2 = 65\%$						
Test for overall effect: Z = 0.05 (P = 0.96)						
**2.14.2 Food secure**						
Iannoti 2017 (5)	0.5166	0.2465	25	24	40.8%	1.68 [1.03, 2.72]
Iannoti 2017 (6)	−0.1812	0.5315	25	24	8.8%	0.83 [0.29, 2.36]
Iannoti 2017 (7)	0.1622	0.2216	25	25	50.5%	1.18 [0.76, 1.82]
**Subtotal (95% CI)**			**75**	**73**	**100.0%**	**1.32 [0.97, 1.80]**
Heterogeneity: $Tau^2 = 0.00$; $Chi^2 = 1.96$, df = 2 (P = 0.38); $I^2 = 0\%$						
Test for overall effect: Z = 1.76 (P = 0.08)						

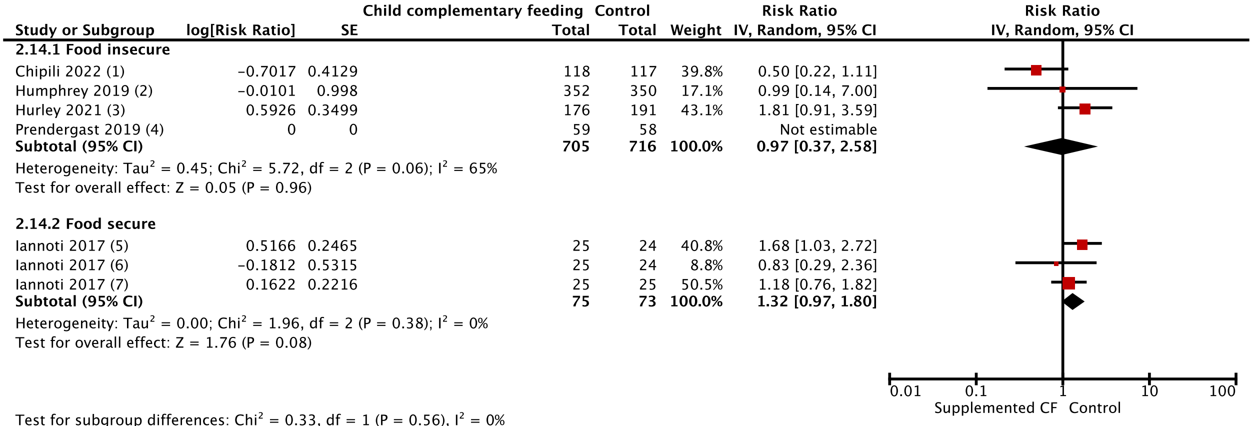

Test for subgroup differences: $Chi^2 = 0.33$, df = 1 (P = 0.56), $I^2 = 0\%$

Footnotes
(1) Fish powder; RCT; Africa; children aged 6-7 months; supplementation for 6 months; cough, endline
(2) SQLNS 20 gm-HIV negative- cRCT, Africa, Aged 6-18 month, supplementation for 12 months
(3) non-RCT, Africa; children aged 6-24 months; supplementation for 12 months, ARI
(4) SQLNS-20 gm-HIV positive- cRCT, Africa, Aged 6-18 month, supplementation for 12 months
(5) Eggs; RCT; Ecuador; children aged 6-9 months; supplementation for 6 months; congestion
(6) Eggs; RCT; Ecuador; children aged 6-9 months; supplementation for 6 months; difficulty breathing
(7) Eggs; RCT; Ecuador; children aged 6-9 months; supplementation for 6 months; cough

Skin disease

Study or Subgroup	log[Risk Ratio]	SE	Child complementary feeding Total	Control Total	Weight	Risk Ratio IV, Random, 95% CI
**2.15.1 Food insecure**						
Chipili 2022 (1)	−1.6179	1.5437	118	117	100.0%	0.20 [0.01, 4.09]
**Subtotal (95% CI)**			**118**	**117**	**100.0%**	**0.20 [0.01, 4.09]**
Heterogeneity: Not applicable						
Test for overall effect: Z = 1.05 (P = 0.29)						
**2.15.2 Food secure**						
Iannoti 2017 (2)	−0.5577	0.363	38	37	63.6%	0.57 [0.28, 1.17]
Iannoti 2017 (3)	−0.6332	0.4801	37	36	36.4%	0.53 [0.21, 1.36]
**Subtotal (95% CI)**			**75**	**73**	**100.0%**	**0.56 [0.32, 0.98]**
Heterogeneity: $Tau^2 = 0.00$; $Chi^2 = 0.02$, df = 1 (P = 0.90); $I^2 = 0\%$						
Test for overall effect: Z = 2.02 (P = 0.04)						

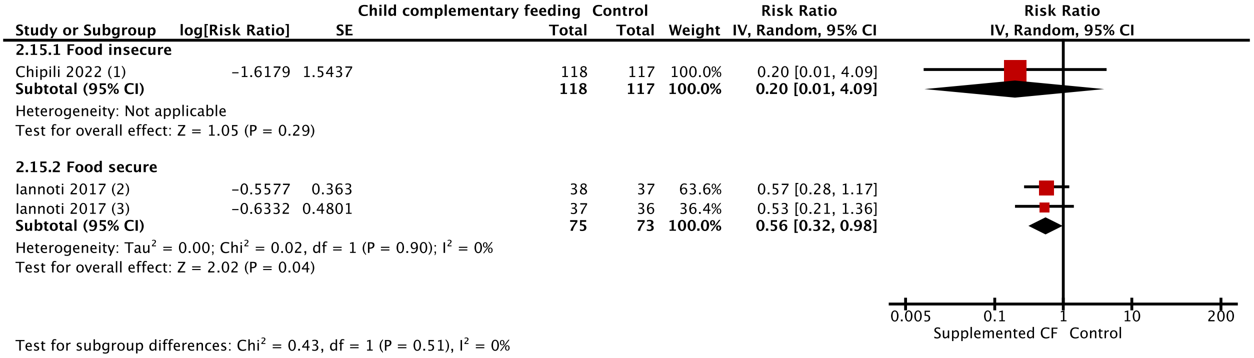

Test for subgroup differences: $Chi^2 = 0.43$, df = 1 (P = 0.51), $I^2 = 0\%$

Footnotes
(1) Fish powder; RCT; Africa; children aged 6-7 months; supplementation for 6 months; Rashes, endline
(2) Eggs; RCT; Ecuador; children aged 6-9 months; supplementation for 6 months; Rashes
(3) Eggs; RCT; Ecuador; children aged 6-9 months; supplementation for 6 months; bruises/scrpas/cuts

Mortality

Study or Subgroup	log[Risk Ratio]	SE	Child complementary feeding Total	Control Total	Weight	Risk Ratio IV, Random, 95% CI
**2.16.1 Food insecure**						
Adu-Afarwuah 2016 (1)	0.5365	0.4704	409	408	3.9%	1.71 [0.68, 4.30]
Ashorn 2015 (2)	-0.6172	0.3816	281	288	4.5%	0.54 [0.26, 1.14]
Bauserman 2015 (3)	-0.6931	1.1748	48	48	1.3%	0.50 [0.05, 5.00]
Becquey 2019 (4)	0.2259	0.3461	809	845	4.8%	1.25 [0.64, 2.47]
Bhandari 2001 (5)	-0.6741	1.2169	104	106	1.2%	0.51 [0.05, 5.53]
Christian 2015 (6)	0.0555	0.5676	567	239	3.3%	1.06 [0.35, 3.22]
Christian 2015 (7)	-0.4413	0.7042	995	239	2.6%	0.64 [0.16, 2.56]
Christian 2015 (8)	0.131	0.8631	562	239	2.0%	1.14 [0.21, 6.19]
Christian 2015 (9)	-1.6607	1.1486	550	239	1.3%	0.19 [0.02, 1.80]
Dewey 2017 (10)	-0.1994	0.2673	847	449	5.3%	0.82 [0.49, 1.38]
Dewey 2017 (11)	-0.2578	0.2593	1047	445	5.4%	0.77 [0.46, 1.28]
Galasso 2019 (12)	0	0.621	139	139	3.0%	1.00 [0.30, 3.38]
Ghosh 2020 (13)	0.392	0.9073	194	192	1.9%	1.48 [0.25, 8.76]
Hess 2015 (14)	-0.8746	0.319	1623	523	5.0%	0.42 [0.22, 0.78]
Humphrey 2019 (15)	1.612	0.2341	383	384	5.6%	5.01 [3.17, 7.93]
Huybregts 2012 (16)	0.793	1.1544	386	284	1.3%	2.21 [0.23, 21.23]
Huybregts 2019 (17)	-0.2763	0.7486	87	88	2.4%	0.76 [0.17, 3.29]
Iannotti 2014 (18)	0.3498	1.6283	202	95	0.7%	1.42 [0.06, 34.51]
Iannotti 2014 (19)	1.0729	1.6298	196	96	0.7%	2.92 [0.12, 71.33]
Isanaka 2010 (20)	-0.9943	1.135	309	345	1.3%	0.37 [0.04, 3.42]
Langendorf 2014 (21)	-0.9676	0.6811	1338	396	2.7%	0.38 [0.10, 1.44]
Langendorf 2014 (22)	-1.9346	0.8648	2059	396	2.0%	0.14 [0.03, 0.79]
Langendorf 2014 (23)	-1.1087	0.7911	1353	397	2.3%	0.33 [0.07, 1.56]
Luby 2018 (24)	-0.2062	0.2418	528	1046	5.5%	0.81 [0.51, 1.31]
Maleta 2015 (25)	-0.0752	0.6213	322	64	3.0%	0.93 [0.27, 3.13]
Maleta 2015 (26)	-0.4155	0.6353	322	64	2.9%	0.66 [0.19, 2.29]
Maleta 2015 (27)	0.1699	0.6084	324	64	3.1%	1.19 [0.36, 3.91]
Maleta 2015 (28)	-0.1462	0.6257	321	64	3.0%	0.86 [0.25, 2.95]
Maleta 2015 (29)	-0.6379	0.663	323	64	2.8%	0.53 [0.14, 1.94]
Mangani 2015 (30)	-0.3655	0.6377	196	204	2.9%	0.69 [0.20, 2.42]
Mangani 2015 (31)	-1.8207	1.1484	210	102	1.3%	0.16 [0.02, 1.54]
Mangani 2015 (32)	-1.0498	0.8998	196	102	1.9%	0.35 [0.06, 2.04]
Null 2018 (33)	-0.078	0.1833	480	1036	5.9%	0.92 [0.65, 1.32]
Prendergast 2019 (34)	-0.8538	0.8178	62	66	2.2%	0.43 [0.09, 2.12]
Stewart 2019 (35)	2.1917	1.4927	331	329	0.8%	8.95 [0.48, 166.89]
**Subtotal (95% CI)**			**18103**	**10085**	**100.0%**	**0.82 [0.61, 1.09]**
Heterogeneity: Tau² = 0.34; Chi² = 84.59, df = 34 (P < 0.00001); I² = 60%						
Test for overall effect: Z = 1.39 (P = 0.17)						
**2.16.2 Food secure**						
Smuts 2019 (36)	0	1.2198	250	125	57.2%	1.00 [0.09, 10.92]
Smuts 2019 (37)	-0.6931	1.41	250	125	42.8%	0.50 [0.03, 7.93]
**Subtotal (95% CI)**			**500**	**250**	**100.0%**	**0.74 [0.12, 4.53]**
Heterogeneity: Tau² = 0.00; Chi² = 0.14, df = 1 (P = 0.71); I² = 0%						
Test for overall effect: Z = 0.32 (P = 0.75)						

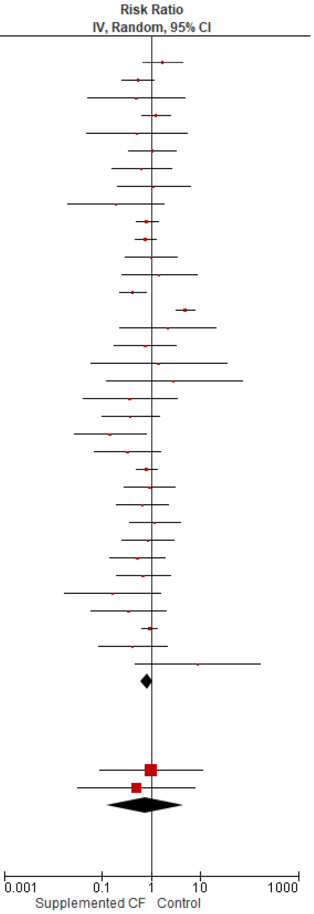

Test for subgroup differences: Chi² = 0.01, df = 1 (P = 0.92), I² = 0%

Footnotes
(1) SQ-LNS; RCT; African region; mothers with >20 wk of gestation; supplementation for 6 months, under 5 mortality
(2) RCT; Malawi; children aged 6-18 months; SQLNS supplementaion for 6 months; infant death (0-19 months)
(3) cRCT, Africa, children aged 5 months; alternative food supplementation for18 months
(4) SQLNS 20gm; cRCT: Africa; children aged 0-17 months
(5) RCT, South-East Asian Region, 4 and 12 mo of age, alternative food supplementation for 6 months intervention
(6) cRCT, South-East Asian Region, age-6-18 months; FBF supplementation for 12 months
(7) Plumpy doz- cRCT, South-East Asian Region, age-6-18 months; supplementation for 12 months
(8) cRCT; chickpeas; South East Asia; aged 6-18 months; supplementation for 12 months
(9) cRCT; Rice/lentils; South East Asia; aged 6-18 months; supplementation for 12 months
(10) SQLNS; cRCT, South-East Asian; 6-24 months age, child supplementation for 18 months, most of these deaths occurred at <14 days postpartum
(11) SQLNS; cRCT, South-East Asian; 6-24 months age, intervention to mother and child, most of these deaths occurred at <14 days postpartum
(12) SQLNS ; cRCT; Madasgar; Children <12 years; supplementation for 12 months; infant deaths
(13) cRCT, Africa, 6-18 months age, FBF supplementation for intervention 6-12 months
(14) zinc; cRCT, Africa, aged 9-18 months of age, supplementation for 9 months; under 5 mortality
(15) SQLNS 20 gm-HIV negative, cRCT, Africa, Aged 6-18 month, supplementation for 12 months; under 5 mortality
(16) Plumpydoz Nutriset 46 g ~247Kcal/d; cRCT; Africa; age 6-36 months, supplementation for 4 months
(17) cRCT; Africa; age 6-23 months, SQLNS supplementation for 12 months; under 24 months mortality
(18) SQ-LNS; RCT African region; children aged 6-11 months; SQLNS supplementation for 6 months
(19) SQ-LNS; RCT African region; children aged 6-11 months; SQLNS supplementation for 3 months
(20) cRCT; Africa; RUTF (500 kcal/day); children 6-60 months; under 5 mortality
(21) SC+ (820 kcal/day); RCT; Africa; aged 6-23 months, supplementation for 5 months; death at <5 yrs,
(22) MQ-LNS; cRCT; Africa; aged 6-23 months, supplementation for 5 months; death at <5 yrs,
(23) HQ LNS; cRCT, Africa, 6-23 months age, supplementation for < 6 months, death at <5 yrs
(24) 20 g SQLNS, cRCT; Bangladesh; 6-24 months age; supplementation for 12 months; under 5 mortality
(25) 40g milk; RCT, Africa; aged 6 months, supplementation for 12 months; death until 18 months of age
(26) 20g milk; RCT, Africa; aged 6 months, supplementation for 12 months; death until 18 months of age
(27) 40g no milk; RCT, Africa; aged 6 months, supplementation for 12 months; death until 18 months of age
(28) 10g milk-death during 6-12 months of age; RCT, Africa; aged 6 months; supplementation for 12 months; death until 18 months of age
(29) 20g no milk; RCT, Africa; aged 6 months, supplementation for 12 months; death until 18 months of age
(30) CSB- RCT, Africa, age 6-18 months, suplementation for 12 month
(31) milk LNS- RCT, Africa, age 6-18 months, suplementation for 12 month
(32) soy LNS- RCT, Africa, age 6-18 months, suplementation for 12 month
(33) SQLNS-20 gm, cRCT, Africa; 6-24 month aged, under 5 mortality
(34) SQLNS-20 gm-HIV positive, cRCT, Africa, Aged 6-18 month, supplementation for 12 months, under 5 mortality
(35) RCT, Africa, 6-9.9 months age; alternative food supplementation for 6 month, under 5 mortality
(36) SQ LNS plus; RCT, Africa; aged 6-12 months, supplementation for 6 months; infant mortality
(37) SQ LNS; RCT, Africa; aged 6-12 months, supplementation for 6 months; infant mortality

Comparison 4: Complementary food education versus control (subgroup by food security status)

HAZ

Study or Subgroup	Mean Difference	SE	IYCF Education Total	Control Total	Weight	Mean Difference IV, Random, 95% CI
**2.1.1 Food insecure**						
Atukunda 2019 (1)	0.11	0.1276	69	69	4.5%	0.11 [−0.14, 0.36]
Fahmida, 2020 (2)	0.09	0.0919	218	214	5.7%	0.09 [−0.09, 0.27]
Hitachi, 2020 (3)	0.04	0.1646	67	113	3.5%	0.04 [−0.28, 0.36]
Kang, 2017 (4)	0.021	0.0066	56	69	7.9%	0.02 [0.01, 0.03]
Martinez, 2018 (5)	0.07	0.0541	145	151	7.0%	0.07 [−0.04, 0.18]
Nair, 2017 (6)	0.07	0.1135	247	246	4.9%	0.07 [−0.15, 0.29]
Owais, 2017 (7)	0.2	0.0314	351	341	7.6%	0.20 [0.14, 0.26]
Reinbott, 2016 (8)	−0.06	0.1257	175	132	4.5%	−0.06 [−0.31, 0.19]
Roy, 2007 (9)	0.23	0.0761	290	282	6.2%	0.23 [0.08, 0.38]
Roy 2022 (10)	0.61	0.2172	96	96	2.4%	0.61 [0.18, 1.04]
Salehi, 2004 (11)	0.41	0.1126	406	405	4.9%	0.41 [0.19, 0.63]
Sharma, 2020 (12)	0.33	0.0681	190	191	6.5%	0.33 [0.20, 0.46]
Singh, 2017 (13)	0.1	0.0845	402	340	5.9%	0.10 [−0.07, 0.27]
Sinharoy 2017 (14)	0.05	0.0743	408	431	6.3%	0.05 [−0.10, 0.20]
Sudfeld, 2021 (15)	0.79	0.2031	123	116	2.7%	0.79 [0.39, 1.19]
Zaman 2008 (16)	0.123	0.2061	95	95	2.6%	0.12 [−0.28, 0.53]
**Subtotal (95% CI)**			**3338**	**3291**	**83.2%**	**0.16 [0.08, 0.24]**
Heterogeneity: $Tau^2 = 0.02$; $Chi^2 = 91.91$, df = 15 (P < 0.00001); $I^2 = 84\%$						
Test for overall effect: Z = 4.01 (P < 0.0001)						
**2.1.2 Food secure**						
Navarro 2013 (17)	0.2	0.1103	193	259	5.0%	0.20 [−0.02, 0.42]
Olaya 2013 (18)	0.14	0.1459	38	38	3.9%	0.14 [−0.15, 0.43]
Penny 2005 (19)	0.38	0.1327	75	76	4.3%	0.38 [0.12, 0.64]
Zhang, 2013 (20)	0.87	0.1612	78	71	3.5%	0.87 [0.55, 1.19]
**Subtotal (95% CI)**			**384**	**444**	**16.8%**	**0.39 [0.09, 0.68]**
Heterogeneity: $Tau^2 = 0.07$; $Chi^2 = 14.38$, df = 3 (P = 0.002); $I^2 = 79\%$						
Test for overall effect: Z = 2.60 (P = 0.009)						
**Total (95% CI)**			**3722**	**3735**	**100.0%**	**0.20 [0.12, 0.28]**
Heterogeneity: $Tau^2 = 0.02$; $Chi^2 = 127.86$, df = 19 (P < 0.00001); $I^2 = 85\%$						
Test for overall effect: Z = 4.94 (P < 0.00001)						
Test for subgroup differences: $Chi^2 = 2.10$, df = 1 (P = 0.15), $I^2 = 52.4\%$						

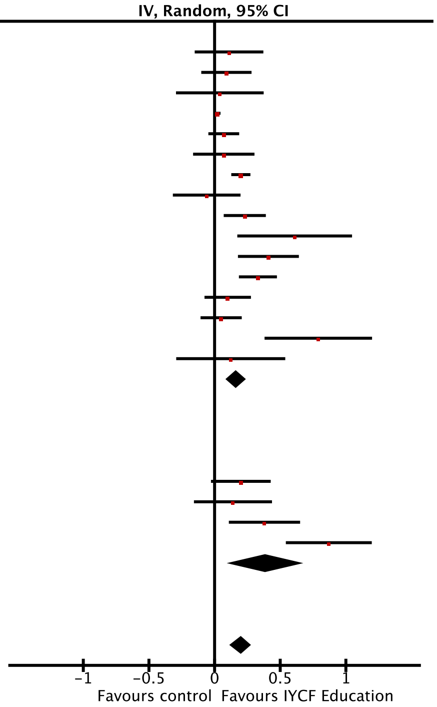

Footnotes
(1) cRCT; African Region; IYCF vs control; Children aged 6–8 months; Follow-up at 20–24 months; HAZ at endline
(2) integrated ICF; cRCT; Indonesia; children aged 0–18 months; follow-up 18 months
(3) Education on maternal and child nutrition and follow-up consultations; non-RCT; African Region; children aged 5–59 months; follow-up 12 months; change in HA
(4) cRCT; African Region; IYCF [community-based participatory nutrition promotion (CPNP)'] vs control; children aged 6–12 months; intervention for 12 months
(5) RCT; Regions of America; children aged 6–24 months; Follow-up 6 months; change in HAZ
(6) change in HAZ; cRCT; South East Asia; support feeding, hygiene, care, and stimulation; vs control; children aged 0–18 months; Intervention for 18 months
(7) Community-based nutrition education program; RCT; Bangladesh; children aged 0–23 months; at 24 months
(8) IYCG and agricultural activities; cRCT; Western Pacific; children aged 0–23 months; Follow-up at 2 years
(9) RCT; South East Asia; children aged 6–9 months; Follow-up 12 months; after intervention
(10) RCT, Bangladesh, Asia, 6–23 months, IYCF
(11) HAZ; RCT; EMRO; environmental health, personal hygiene and nutritional edu vs control; children aged 0–59 months; Intervention for 12 months
(12) HAZ; non-RCT; South East Asia; nutritional education arm vs control; children aged 4–6 months; Intervention for 6 months
(13) Integrated Nutrition and Health Program (INHP II); qRCT; South East Asia; children aged 6 months to 6 years; endline at 18 momths
(14) HAZ; cRCT; Western Rwanda; Classic intervention intervention vs control; children <5 years; Intervention for 2 years
(15) HAZ; cRCT; African Region;integrated health nutrition and responsive stimulation vs control; children <1 years; Intervention for 18 months
(16) cRCT; Pakistan; 6–18 months old children; duration of 180 days
(17) non-RCT, Regions of America, IYCF vs control; children <2 years; Intervention for 24 months; HAZ at endline
(18) RCT, Region of Americas, children of 6months, change in HAZ
(19) cRCT; Peru; children aged 4 months; follow-up at 18 months, endline
(20) cRCT; Western Pacific Region; children at 6 months; at 18 months

Stunting

Study or Subgroup	log[Risk Ratio]	SE	IYCF Education Total	Control Total	Weight	Risk Ratio IV, Random, 95% CI
**2.2.1 Food insecure**						
Abiyu, 2020 (1)	−0.3857	0.1939	136	141	2.3%	0.68 [0.46, 0.99]
Ali 2022 (2)	−0.1173	0.3356	46	90	0.9%	0.89 [0.46, 1.72]
Atukunda 2019 (3)	−0.3562	0.173	70	71	2.7%	0.70 [0.50, 0.98]
Bhandari, 2001 (4)	−0.1491	0.0978	97	93	5.3%	0.86 [0.71, 1.04]
Bhandari, 2003 (5)	−0.0196	0.1038	218	197	5.0%	0.98 [0.80, 1.20]
Fahmida, 2020 (6)	−0.1105	0.1174	218	214	4.4%	0.90 [0.71, 1.13]
Gope, 2019 (7)	−0.0026	0.039	1213	1045	8.4%	1.00 [0.92, 1.08]
Kabahenda, 2014 (8)	−0.3361	0.3454	45	38	0.9%	0.71 [0.36, 1.41]
Kang, 2017 (9)	0.0887	0.3452	56	69	0.9%	1.09 [0.56, 2.15]
More, 2017 (10)	0.028	0.0461	1089	1098	8.0%	1.03 [0.94, 1.13]
Nair, 2017 (11)	−0.0294	0.0572	300	312	7.5%	0.97 [0.87, 1.09]
Saaka, 2021 (12)	0.016	0.1304	403	273	4.0%	1.02 [0.79, 1.31]
Saleem, 2014 (13)	−0.1203	0.0723	95	75	6.6%	0.89 [0.77, 1.02]
Sharma, 2020 (14)	−0.6009	0.4971	190	191	0.4%	0.55 [0.21, 1.45]
Singh, 2017 (15)	−0.0416	0.0471	402	340	8.0%	0.96 [0.87, 1.05]
Sinharoy 2017 (16)	−0.0399	0.0525	997	1038	7.7%	0.96 [0.87, 1.07]
Sudfeld, 2021 (17)	−0.6239	0.2144	123	116	2.0%	0.54 [0.35, 0.82]
Sustanto, 2019 (18)	0.5306	0.3382	45	45	0.9%	1.70 [0.88, 3.30]
Yadav, 2014 (19)	−0.1497	0.1034	564	759	5.0%	0.86 [0.70, 1.05]
Young 2021 (20)	−0.3087	0.1621	112	58	3.0%	0.73 [0.53, 1.01]
Young 2021 (21)	−0.021	0.1409	112	58	3.6%	0.98 [0.74, 1.29]
**Subtotal (95% CI)**			**6531**	**6321**	**87.7%**	**0.93 [0.89, 0.98]**
Heterogeneity: $Tau^2 = 0.00; Chi^2 = 28.95, df = 20 (P = 0.09); I^2 = 31\%$						
Test for overall effect: Z = 2.63 (P = 0.009)						
**2.2.2 Food secure**						
Guldan 2000 (22)	−0.9559	0.5233	220	203	0.4%	0.38 [0.14, 1.07]
Liang 2018 (23)	−0.3175	0.0548	2693	8425	7.6%	0.73 [0.65, 0.81]
Navarro 2013 (24)	−0.5008	0.3076	193	259	1.1%	0.61 [0.33, 1.11]
Penny 2005 (25)	−1.2338	0.6352	68	66	0.3%	0.29 [0.08, 1.01]
Walsh 2002 (26)	0.2441	0.1657	738	78	2.9%	1.28 [0.92, 1.77]
**Subtotal (95% CI)**			**3912**	**9031**	**12.3%**	**0.72 [0.48, 1.06]**
Heterogeneity: $Tau^2 = 0.11; Chi^2 = 14.91, df = 4 (P = 0.005); I^2 = 73\%$						
Test for overall effect: Z = 1.65 (P = 0.10)						
**Total (95% CI)**			**10443**	**15352**	**100.0%**	**0.90 [0.84, 0.96]**
Heterogeneity: $Tau^2 = 0.01; Chi^2 = 62.85, df = 25 (P < 0.0001); I^2 = 60\%$						
Test for overall effect: Z = 3.15 (P = 0.002)						
Test for subgroup differences: $Chi^2 = 1.70, df = 1 (P = 0.19), I^2 = 41.1\%$						

Risk Ratio
IV, Random, 95% CI

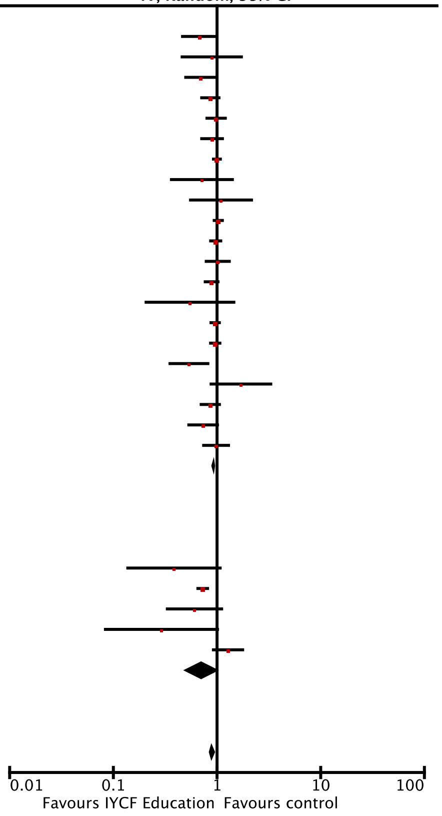

Footnotes

(1) cRCT; African Region; IYCF vs control; children aged 12–24 months; Intervention for 9 months; Prevalence of stunting at 9 months; study reported cluster...
(2) RCT, Somalia, Africa, nutrition couselling, IDP children
(3) cRCT; African Region; IYCF vs control; Children aged 6–8 months; Follow-up at 20–24 months; prevalence of stunting
(4) RCT; South East Asia; nutritional counselling vs control; children aged 4–12 months; intervention for 12 months; prevalence of stunting
(5) cRCT; South East Asia; children aged 6–18 months; Intervention for 6 months; prevalence of stunting between 12–18 months
(6) integrated ICF; cRCT; Indonesia; children aged 0–18 months; follow-up 18 months
(7) non-RCT; South-East Asia; PLA vs control; children aged 6 months to 3 years; Intervention for 24 months; prevalence of stunting
(8) non-RCT; African Region; Structured nutrition education programme vs control; children aged 6–48 months; Intervention for 9 months; incidence of stunting
(9) cRCT; African Region; IYCF [community-based participatory nutrition promotion (CPNP)] vs control; children aged 6–12 months; intervention for 12 months;...
(10) cRCT; South East Asia; integrated activities vs control; children aged 5 years; prevalence of stunting (Endline HAZ<-2)
(11) cRCT; South East Asia; support feeding, hygiene, care, and stimulation; vs control; children aged 0–18 months; Intervention for 18 months; prevalence of stuntin
(12) non-RCT; African Region; IYCF vs control; children aged 6–36 months; Intervention for 12 months Prevalence of stunting
(13) cRCT; EMRO; IYCF vs control; children newborn; Intervention for 10 weeks; stunting at endline
(14) non-RCT; South East Asia; nutritional education arm vs control; children aged 4–6 months; Intervention for 6 months; prevalence of stunting
(15) Integrated Nutrition and Health Program (INHP II); qRCT; South East Asia; children aged 6 months to 6 years; endline at 18 momths
(16) cRCT; Western Rwanda; Classic intervention intervention vs control; children <5 years; Intervention for 2 years; stunting
(17) cRCT; African Region; integrated health nutrition and responsive stimulation vs control; children <1 years; Intervention for 18 months; stunting
(18) cRCT; South East Asia; growth, nutrition and development intervention vs control; children aged 0–72 months; Intervention for 12 weeks; stunting
(19) non-RCT; South East Asia; IYCF vs control; children aged <3 years; intervention for 12 months; prevalence of stunting
(20) cRCT; Cambodia, Western Pacific; traditional Positive Deviance/Hearth programme (PDH); prevalence at 3 months followup
(21) cRCT; Cambodia, Western Pacific; traditional Positive Deviance/Hearth with Interactive Voice Calling (PDH-IVC);prevalence at 3 months followup
(22) non-RCT; China; 4–12 months; intervention for 1 year
(23) non-RCT; Western Pacific region; IYCF vs control; children <3 years; Intervention for 4 years; prevalence of stunting
(24) non-RCT, Regions of America, IYCF vs control; children <2 years; Intervention for 24 months; prevalence of stunting
(25) cRCT; Peru; children aged 4 months; follow-up at 18 months, endline
(26) non-RCT; rural Africa; EBF vs. control for 6 months; intervention provided to mothers; stunting (HAZ <-3 to <-2) at 2 years

WHZ

Study or Subgroup	Mean Difference	SE	Weight	Mean Difference IV, Random, 95% CI
**2.3.1 Food insecure**				
Atukunda 2019 (1)	−0.19	0.1684	3.8%	−0.19 [−0.52, 0.14]
Hitachi, 2020 (2)	−0.14	0.0237	10.8%	−0.14 [−0.19, −0.09]
Kang, 2017 (3)	0.041	0.0085	11.2%	0.04 [0.02, 0.06]
Martinez, 2018 (4)	0.08	0.0808	7.7%	0.08 [−0.08, 0.24]
Nair, 2017 (5)	0	0.0841	7.5%	0.00 [−0.16, 0.16]
Reinbott, 2016 (6)	0	0.1135	5.9%	0.00 [−0.22, 0.22]
Roy, 2007 (7)	0.16	0.0832	7.6%	0.16 [−0.00, 0.32]
Roy 2022 (8)	0.64	0.1826	3.4%	0.64 [0.28, 1.00]
Salehi, 2004 (9)	0.266	0.1196	5.6%	0.27 [0.03, 0.50]
Sharma, 2020 (10)	0.23	0.076	8.0%	0.23 [0.08, 0.38]
Sinharoy 2017 (11)	0.018	0.0645	8.7%	0.02 [−0.11, 0.14]
Sudfeld, 2021 (12)	−0.16	0.1838	3.4%	−0.16 [−0.52, 0.20]
Zaman 2008 (13)	0.508	0.172	3.7%	0.51 [0.17, 0.85]
**Subtotal (95% CI)**			**87.3%**	**0.08 [−0.00, 0.17]**
Heterogeneity: $Tau^2 = 0.01$; $Chi^2 = 88.44$, df = 12 (P < 0.00001); $I^2 = 86\%$				
Test for overall effect: Z = 1.87 (P = 0.06)				
**2.3.2 Food secure**				
Olaya 2013 (14)	0.1	0.1633	3.9%	0.10 [−0.22, 0.42]
Penny 2005 (15)	0.1	0.1353	4.9%	0.10 [−0.17, 0.37]
Zhang, 2013 (16)	0.3	0.1671	3.8%	0.30 [−0.03, 0.63]
**Subtotal (95% CI)**			**12.7%**	**0.16 [−0.02, 0.33]**
Heterogeneity: $Tau^2 = 0.00$; $Chi^2 = 1.03$, df = 2 (P = 0.60); $I^2 = 0\%$				
Test for overall effect: Z = 1.76 (P = 0.08)				
**Total (95% CI)**			**100.0%**	**0.09 [0.01, 0.17]**
Heterogeneity: $Tau^2 = 0.01$; $Chi^2 = 91.60$, df = 15 (P < 0.00001); $I^2 = 84\%$				
Test for overall effect: Z = 2.26 (P = 0.02)				
Test for subgroup differences: $Chi^2 = 0.57$, df = 1 (P = 0.45), $I^2 = 0\%$				

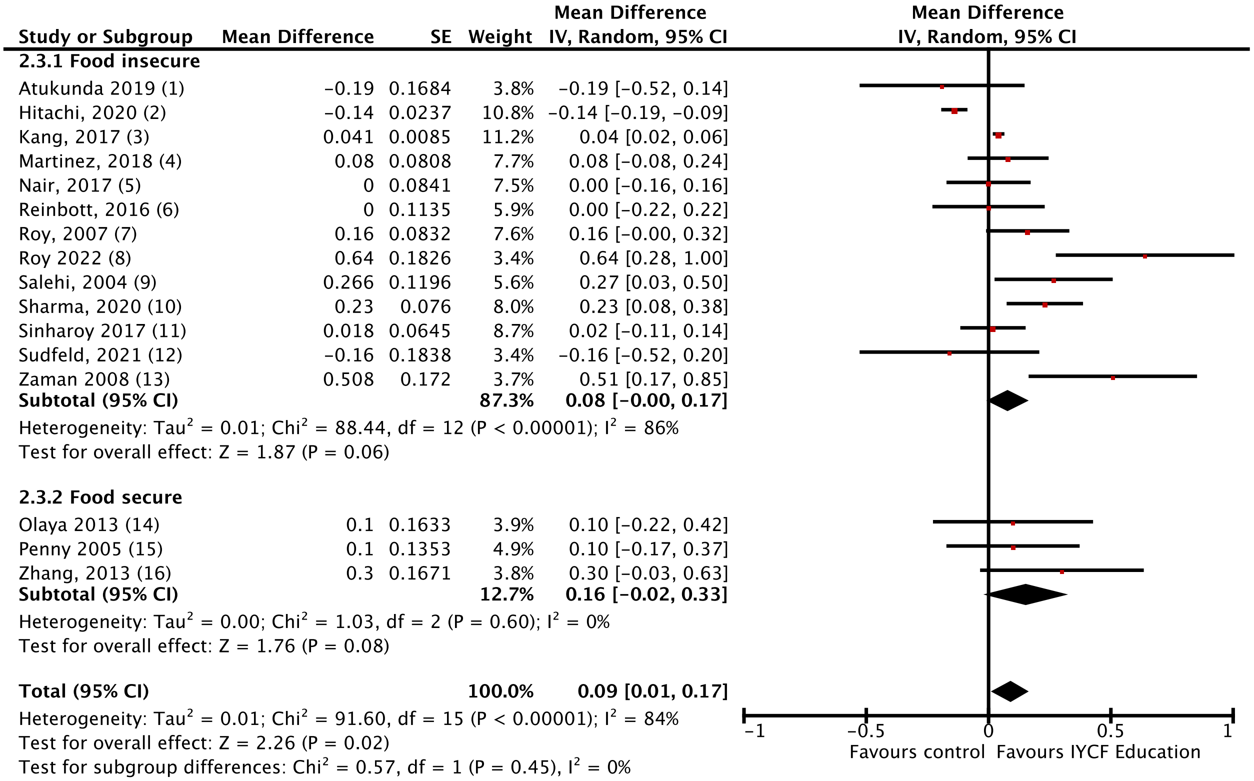

Footnotes
(1) cRCT; African Region; IYCF vs control; Children aged 6-8 months; Follow-up at 20-24 months; WHZ at endline
(2) Education on maternal and child nutrition and follow-up consultations; non-RCT; African Region; children aged 5-59 months;...
(3) change in WHZ; cRCT; African Region; IYCF [community-based participatory nutrition promotion (CPNP)'] vs control; children aged...
(4) RCT; Regions of America; children aged 6-24 months; Follow-up 6 months; change in WHZ
(5) cRCT; South East Asia; support feeding, hygiene, care, and stimulation; vs control; children aged 0-18 months; Intervention for 18...
(6) IYCG and agricultural activities; cRCT; Western Pacific; children aged 0-23 months; Follow-up at 2 years
(7) RCT; South East Asia; children aged 6-9 months; Follow-up 12 months; after intervention
(8) RCT, Bangladesh, Asia, 6-23 months, IYCF
(9) WHZ; RCT; EMRO; environmental health, personal hygiene and nutritional edu vs control; children aged 0-59 months; Intervention for...
(10) WHZ; non-RCT; South East Asia; nutritional education arm vs control; children aged 4-6 months; Intervention for 6 months
(11) WHZ; cRCT; Western Rwanda; Classic intervention intervention vs control; children <5 years; Intervention for 2 years
(12) WHZ; cRCT; African Region; integrated health nutrition and responsive stimulation vs control; children <1 years; Intervention for 18...
(13) cRCT; Pakistan; 6-18 months old children; duration of 180 days
(14) RCT, Region of Americas, children of 6months, change in WHZ
(15) cRCT; Peru; children aged 4 months; follow-up at 18 months, endline
(16) kgs; cRCT; Western Pacific Region; children at 6 months; at 18 months

Wasting

Study or Subgroup	log[Risk Ratio]	SE	IYCF Education Total	Control Total	Weight	Risk Ratio IV, Random, 95% CI
**2.4.1 Food insecure**						
Abiyu, 2020 (1)	−0.118	0.3746	136	141	3.1%	0.89 [0.43, 1.85]
Ali 2022 (2)	−0.1555	0.4153	46	90	2.6%	0.86 [0.38, 1.93]
Atukunda 2019 (3)	0.4196	0.8972	70	71	0.7%	1.52 [0.26, 8.83]
Bhandari, 2001 (4)	−0.2653	0.3591	97	93	3.3%	0.77 [0.38, 1.55]
Kadiyala, 2021 (5)	0.0625	0.1717	453	421	7.8%	1.06 [0.76, 1.49]
Kadiyala, 2021 (6)	−0.064	0.173	421	438	7.7%	0.94 [0.67, 1.32]
Kang, 2017 (7)	−0.0826	0.2844	48	68	4.5%	0.92 [0.53, 1.61]
More, 2017 (8)	−0.0827	0.1156	1088	1097	10.0%	0.92 [0.73, 1.15]
Nair, 2017 (9)	−0.0492	0.0978	300	312	10.7%	0.95 [0.79, 1.15]
Saaka, 2021 (10)	0.7806	0.2582	403	273	5.1%	2.18 [1.32, 3.62]
Saleem, 2014 (11)	−0.9628	1.2161	95	75	0.4%	0.38 [0.04, 4.14]
Sharma, 2020 (12)	−0.7569	0.307	190	191	4.1%	0.47 [0.26, 0.86]
Sinharoy 2017 (13)	0.3188	0.3288	979	1026	3.7%	1.38 [0.72, 2.62]
Sudfeld, 2021 (14)	−0.3463	0.7527	123	116	0.9%	0.71 [0.16, 3.09]
Sustanto, 2019 (15)	−0.6931	0.84	45	45	0.8%	0.50 [0.10, 2.59]
Teshome, 2020 (16)	−0.9043	0.2245	192	196	6.0%	0.40 [0.26, 0.63]
Yadav, 2014 (17)	−0.3071	0.1204	564	759	9.8%	0.74 [0.58, 0.93]
Young 2021 (18)	0.2174	0.3235	112	58	3.8%	1.24 [0.66, 2.34]
Young 2021 (19)	−0.0339	0.3472	112	58	3.4%	0.97 [0.49, 1.91]
**Subtotal (95% CI)**			**5474**	**5528**	**88.3%**	**0.89 [0.76, 1.05]**
Heterogeneity: $Tau^2 = 0.05$; $Chi^2 = 37.45$, df = 18 (P = 0.005); $I^2 = 52\%$						
Test for overall effect: Z = 1.33 (P = 0.18)						
**2.4.2 Food secure**						
Liang 2018 (20)	−0.2722	0.1196	2693	8425	9.8%	0.76 [0.60, 0.96]
Navarro 2013 (21)	0.5818	0.7578	193	259	0.9%	1.79 [0.41, 7.90]
Walsh 2002 (22)	0.0041	0.7338	738	78	1.0%	1.00 [0.24, 4.23]
**Subtotal (95% CI)**			**3624**	**8762**	**11.7%**	**0.78 [0.62, 0.98]**
Heterogeneity: $Tau^2 = 0.00$; $Chi^2 = 1.36$, df = 2 (P = 0.51); $I^2 = 0\%$						
Test for overall effect: Z = 2.10 (P = 0.04)						
**Total (95% CI)**			**9098**	**14290**	**100.0%**	**0.89 [0.77, 1.03]**

Heterogeneity: $Tau^2 = 0.04$; $Chi^2 = 39.89$, df = 21 (P = 0.008); $I^2 = 47\%$
Test for overall effect: Z = 1.61 (P = 0.11)
Test for subgroup differences: $Chi^2 = 0.85$, df = 1 (P = 0.36), $I^2 = 0\%$

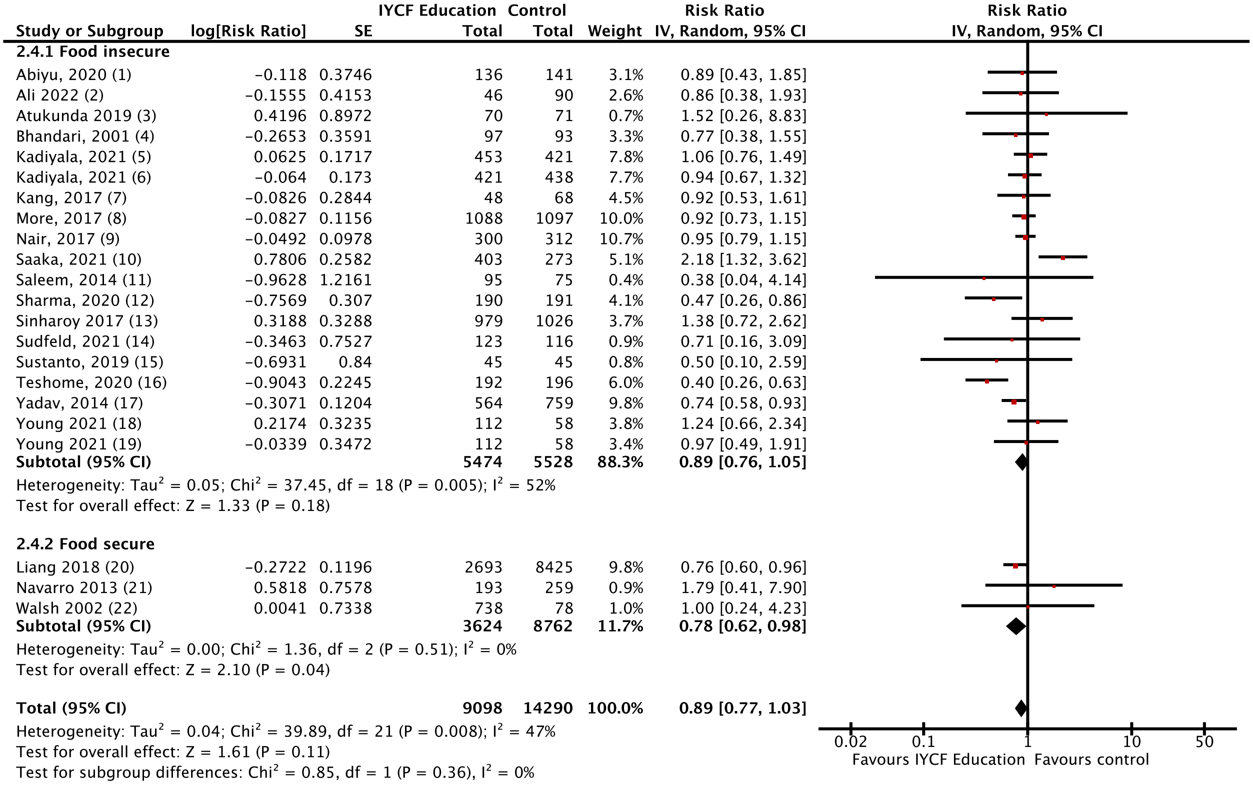

Footnotes
(1) cRCT; African Region; IYCF vs control; children aged 12-24 months; Intervention for 9 months; Prevalence of wasting (endline WHZ <-2) at 9 months; study...
(2) RCT, Somalia, Africa, nutrition couselling, IDP children
(3) cRCT; African Region; IYCF vs control; Children aged 6-8 months; Follow-up at 20-24 months; prevalence of wasting (Endline WHZ<-2)
(4) RCT; South East Asia; nutritional counselling vs control; children aged 4-12 months; intervention for 12 months; prevalence of wasting (Endline WHZ < -2)
(5) cRCT; South East Asia; Nutrition+Agriculture+ PLA vs agriculture+Nutrition; children aged 6-23 months; Intervention for 36 months; prevalence of wasting...
(6) cRCT; South East Asia; Nutrition+Agriculture vs agriculture; children aged 6-23 months; Intervention for 36 months; prevalence of wasting (Endline WHZ<-2)
(7) cRCT; African Region; IYCF (community-based participatory nutrition promotion (CPNP)) vs control; children aged 6-12 months; intervention for 12 months;...
(8) cRCT; South East Asia; integrated activities vs control; children aged 5 years; prevalence of wasting (Endline WHZ<-2)
(9) cRCT; South East Asia; support feeding, hygiene, care, and stimulation; vs control; children aged 0-18 months; Intervention for 18 months; prevalence of...
(10) non-RCT; African Region; IYCF vs control; children aged 6-36 months; Intervention for 12 months Prevalence of wasting (Endline WHZ < -2) after 12 months;
(11) cRCT; EMRO; IYCF vs control; children newborn; Intervention for 10 weeks; Prevalence of wasting (Endline WHZ<-2)
(12) non-RCT; South East Asia; nutritional education arm vs control; children aged 4-6 months; Intervention for 6 months; prevalence of wasting (Endline WHZ<-2)
(13) cRCT; Western Rwanda; Classic intervention vs control; children <5 years; Intervention for 2 years; prevalence of wasting (Endline WHZ <-2)
(14) cRCT; African Region; integrated health nutrition and responsive stimulation vs control; children <1 years; Intervention for 18 months; Prevalence of wasting...
(15) cRCT; South East Asia; growth, nutrition and development intervention vs control; children aged 0-72 months; Intervention for 12 weeks; prevalence of wasting.
(16) cRCT; African Region; Pulse education nutrition program vs control; children aged 6-15 months; Intervention for 9 month; prevalence of wasting (Endline...
(17) non-RCT; South East Asia; IYCF vs control; children aged <3 years; intervention for 12 months; prevalence of wasting (Endline WHZ<-2)
(18) cRCT; Cambodia, Western Pacific; traditional Positive Deviance/Hearth programme (PDH); prevalence at 3 months followup
(19) cRCT; Cambodia, Western Pacific; traditional Positive Deviance/Hearth with Interactive Voice Calling (PDH-IVC);prevalence at 3 months followup
(20) non-RCT; Western Pacific region; IYCF vs control; children <3 years; Intervention for 4 years; prevalence of wasting (Endline WHZ<-2)
(21) non-RCT, Regions of America, IYCF vs control; children <2 years; Intervention for 24 months; prevalence of wasting (Endline WHZ<-2)
(22) non-RCT; rural Africa; EBF vs. control for 6 months; intervention provided to mothers; prevalence of wasting (WAZ <-3 to <-2) at 2 years

Prevalence of severe wasting

Study or Subgroup	log[Risk Ratio]	SE	Weight	Risk Ratio IV, Random, 95% CI
**3.5.1 Food insecure**				
Sustanto, 2019 (1)	-1.7918	1.0593	63.9%	0.17 [0.02, 1.33]
**Subtotal (95% CI)**			**63.9%**	**0.17 [0.02, 1.33]**
Heterogeneity: Not applicable				
Test for overall effect: Z = 1.69 (P = 0.09)				
**3.5.2 Food secure**				
Walsh 2002 (2)	-2.2472	1.4092	36.1%	0.11 [0.01, 1.67]
**Subtotal (95% CI)**			**36.1%**	**0.11 [0.01, 1.67]**
Heterogeneity: Not applicable				
Test for overall effect: Z = 1.59 (P = 0.11)				
**Total (95% CI)**			**100.0%**	**0.14 [0.03, 0.74]**

Heterogeneity: Tau² = 0.00; Chi² = 0.07, df = 1 (P = 0.80); I² = 0%
Test for overall effect: Z = 2.31 (P = 0.02)
Test for subgroup differences: Chi² = 0.07, df = 1 (P = 0.80), I² = 0%

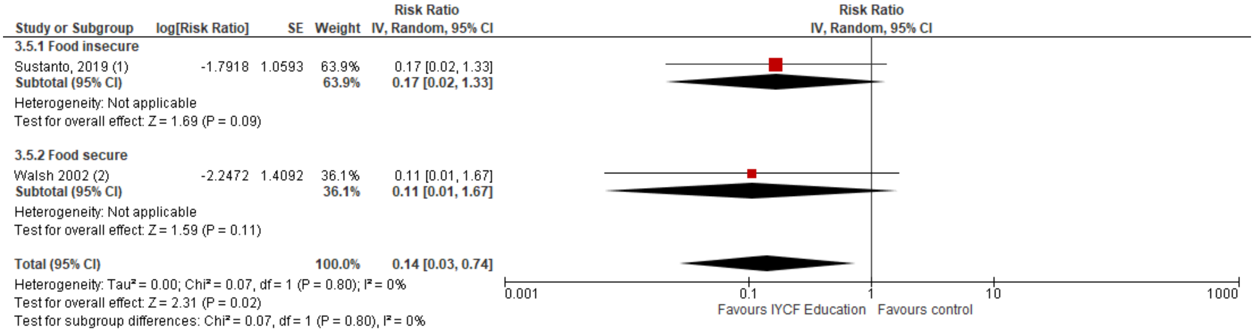

Footnotes
(1) cRCT; South East Asia; growth, nutrition and development intervention vs control; children aged 0-72 months; Intervention for 12 weeks; prevalence of severe wasting (Endline WHZ<-3)
(2) non-RCT; rural Africa; EBF vs. control for 6 months; intervention provided to mothers; severe wasting (WHZ <-3) at 2 years

WAZ

Study or Subgroup	Mean Difference	SE	IYCF Education Total	Control Total	Weight	Mean Difference IV, Random, 95% CI
**2.6.1 Food insecure**						
Atukunda 2019 (1)	0.09	0.2347	69	69	2.3%	0.09 [−0.37, 0.55]
Hitachi, 2020 (2)	−0.02	0.041	67	113	8.3%	−0.02 [−0.10, 0.06]
Kang, 2017 (3)	0.028	0.0061	56	69	9.0%	0.03 [0.02, 0.04]
Martinez, 2018 (4)	0.08	0.0523	145	151	7.9%	0.08 [−0.02, 0.18]
Nair, 2017 (5)	0.04	0.0847	302	313	6.6%	0.04 [−0.13, 0.21]
Owais, 2017 (6)	0	0.0806	351	341	6.7%	0.00 [−0.16, 0.16]
Reinbott, 2016 (7)	−0.02	0.1128	175	132	5.4%	−0.02 [−0.24, 0.20]
Roy, 2007 (8)	0.24	0.0697	290	282	7.2%	0.24 [0.10, 0.38]
Roy 2022 (9)	0.82	0.1523	96	96	4.1%	0.82 [0.52, 1.12]
Salehi, 2004 (10)	0.45	0.0738	406	405	7.0%	0.45 [0.31, 0.59]
Sharma, 2020 (11)	0.32	0.0754	190	191	7.0%	0.32 [0.17, 0.47]
Singh, 2017 (12)	0.1	0.081	402	340	6.7%	0.10 [−0.06, 0.26]
Sudfeld, 2021 (13)	0.28	0.1225	123	116	5.1%	0.28 [0.04, 0.52]
Zaman 2008 (14)	0.546	0.2379	95	95	2.3%	0.55 [0.08, 1.01]
**Subtotal (95% CI)**			**2767**	**2713**	**85.6%**	**0.17 [0.09, 0.26]**
Heterogeneity: Tau² = 0.02; Chi² = 94.70, df = 13 (P < 0.00001); I² = 86%						
Test for overall effect: Z = 3.85 (P = 0.0001)						
**2.6.2 Food secure**						
Olaya 2013 (15)	0.19	0.1021	38	38	5.8%	0.19 [−0.01, 0.39]
Penny 2005 (16)	0.29	0.1409	75	76	4.4%	0.29 [0.01, 0.57]
Zhang, 2013 (17)	0.27	0.1502	78	71	4.1%	0.27 [−0.02, 0.56]
**Subtotal (95% CI)**			**191**	**185**	**14.4%**	**0.24 [0.09, 0.38]**
Heterogeneity: Tau² = 0.00; Chi² = 0.40, df = 2 (P = 0.82); I² = 0%						
Test for overall effect: Z = 3.24 (P = 0.001)						
**Total (95% CI)**			**2958**	**2898**	**100.0%**	**0.18 [0.10, 0.27]**

Heterogeneity: Tau² = 0.02; Chi² = 102.62, df = 16 (P < 0.00001); I² = 84%
Test for overall effect: Z = 4.43 (P < 0.00001)
Test for subgroup differences: Chi² = 0.51, df = 1 (P = 0.48), I² = 0%

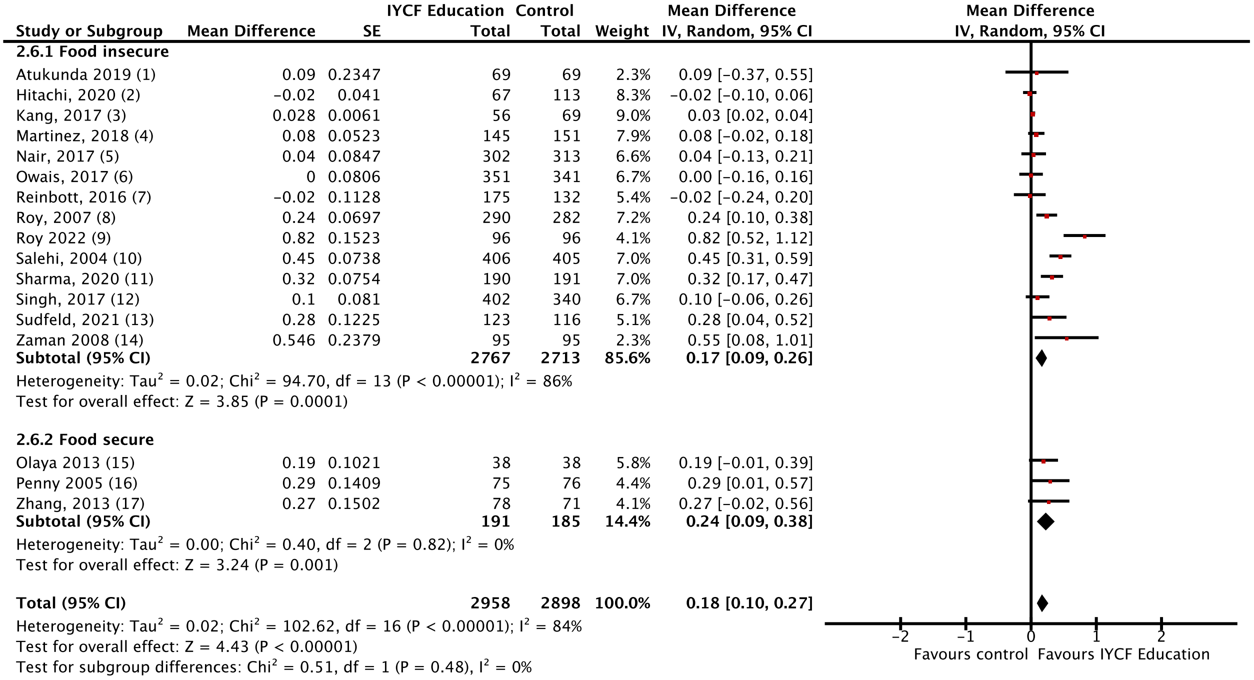

Footnotes
(1) WAZ; cRCT; African Region; IYCF vs control; Children aged 6-8 months; Follow-up at 20-24 months
(2) Education on maternal and child nutrition and follow-up consultations; non-RCT; African Region; children aged 5-59 months; follow-up 12 months; change in WA
(3) change in WAZ; cRCT; African Region; IYCF [community-based participatory nutrition promotion (CPNP)'] vs control; children aged 6-12 months; intervention for..
(4) RCT; Regions of America; children aged 6-24 months; Follow-up 6 months; change in WAZ
(5) cRCT; South East Asia; support feeding, hygiene, care, and stimulation; vs control; children aged 0-18 months; Intervention for 18 months; WAZ at 18 months
(6) Community-based nutrition education program; RCT; Bangladesh; children aged 0-23 months; at 24 months
(7) IYCG and agricultural activities; cRCT; Western Pacific; children aged 0-23 months; Follow-up at 2 years
(8) RCT; South East Asia; children aged 6-9 months; Follow-up 12 months; after intervention
(9) RCT, Bangladesh, Asia, 6-23 months, IYCF
(10) RCT; EMRO; environmental health, personal hygiene and nutritional edu vs control; children aged 0-59 months; Intervention for 12 months
(11) WAZ; non-RCT; South East Asia; nutritional education arm vs control; children aged 4-6 months; Intervention for 6 months
(12) Integrated Nutrition and Health Program (INHP II); qRCT; South East Asia; children aged 6 months to 6 years; endline at 18 momths
(13) WAZ; cRCT; African Region; integrated health nutrition and responsive stimulation vs control; children <1 years; Intervention for 18 months
(14) cRCT; Pakistan; 6-18 months old children; duration of 180 days
(15) RCT, Region of Americas, children of 6months, change in WAZ
(16) cRCT; Peru; children aged 4 months; follow-up at 18 months, endline
(17) cRCT; Western Pacific Region; children at 6 months; at 18 months

Underweight

Study or Subgroup	log[Risk Ratio]	SE	IYCF Education Total	Control Total	Weight	Risk Ratio IV, Random, 95% CI
**2.7.1 Food insecure**						
Abiyu, 2020 (1)	−0.4119	0.2384	136	141	3.3%	0.66 [0.42, 1.06]
Ali 2022 (2)	−0.0455	0.195	46	90	4.3%	0.96 [0.65, 1.40]
Atukunda 2019 (3)	−0.14	0.5302	70	71	0.9%	0.87 [0.31, 2.46]
Gope, 2019 (4)	−0.1108	0.0399	1235	1100	9.4%	0.90 [0.83, 0.97]
Kabahenda, 2014 (5)	0.1186	0.7313	45	38	0.5%	1.13 [0.27, 4.72]
Kang, 2017 (6)	−0.179	0.2366	56	69	3.3%	0.84 [0.53, 1.33]
More, 2017 (7)	0.021	0.0538	1088	1098	9.0%	1.02 [0.92, 1.13]
Nair, 2017 (8)	−0.0508	0.0608	302	313	8.8%	0.95 [0.84, 1.07]
Roy, 2007 (9)	0.1193	0.0443	290	282	9.2%	1.13 [1.03, 1.23]
Saaka, 2021 (10)	0.5055	0.1759	403	273	4.8%	1.66 [1.17, 2.34]
Saleem, 2014 (11)	−0.47	0.2654	95	75	2.9%	0.63 [0.37, 1.05]
Sharma, 2020 (12)	−0.6338	0.2644	190	191	2.9%	0.53 [0.32, 0.89]
Singh, 2017 (13)	0.0277	0.0741	402	340	8.3%	1.03 [0.89, 1.19]
Sudfeld, 2021 (14)	−1.157	0.8062	123	116	0.4%	0.31 [0.06, 1.53]
Sustanto, 2019 (15)	0	0.6749	45	45	0.6%	1.00 [0.27, 3.75]
Teshome, 2020 (16)	−0.9043	0.2245	192	196	3.6%	0.40 [0.26, 0.63]
Yadav, 2014 (17)	−0.3459	0.1089	564	759	7.0%	0.71 [0.57, 0.88]
Young 2021 (18)	−0.0885	0.1789	112	58	4.7%	0.92 [0.64, 1.30]
Young 2021 (19)	−0.0625	0.1771	112	58	4.7%	0.94 [0.66, 1.33]
**Subtotal (95% CI)**			**5506**	**5313**	**88.4%**	**0.90 [0.81, 1.00]**
Heterogeneity: Tau² = 0.02; Chi² = 63.01, df = 18 (P < 0.00001); I² = 71%						
Test for overall effect: Z = 1.97 (P = 0.05)						
**2.7.2 Food secure**						
Guldan 2000 (20)	−2.7881	1.4574	220	203	0.1%	0.06 [0.00, 1.07]
Liang 2018 (21)	−0.3299	0.0875	2693	8425	7.8%	0.72 [0.61, 0.85]
Walsh 2002 (22)	−0.2934	0.2219	738	78	3.6%	0.75 [0.48, 1.15]
**Subtotal (95% CI)**			**3651**	**8706**	**11.6%**	**0.71 [0.53, 0.95]**
Heterogeneity: Tau² = 0.02; Chi² = 2.87, df = 2 (P = 0.24); I² = 30%						
Test for overall effect: Z = 2.32 (P = 0.02)						
**Total (95% CI)**			**9157**	**14019**	**100.0%**	**0.87 [0.78, 0.97]**
Heterogeneity: Tau² = 0.03; Chi² = 78.56, df = 21 (P < 0.00001); I² = 73%						
Test for overall effect: Z = 2.59 (P = 0.010)						
Test for subgroup differences: Chi² = 2.28, df = 1 (P = 0.13), I² = 56.2%						

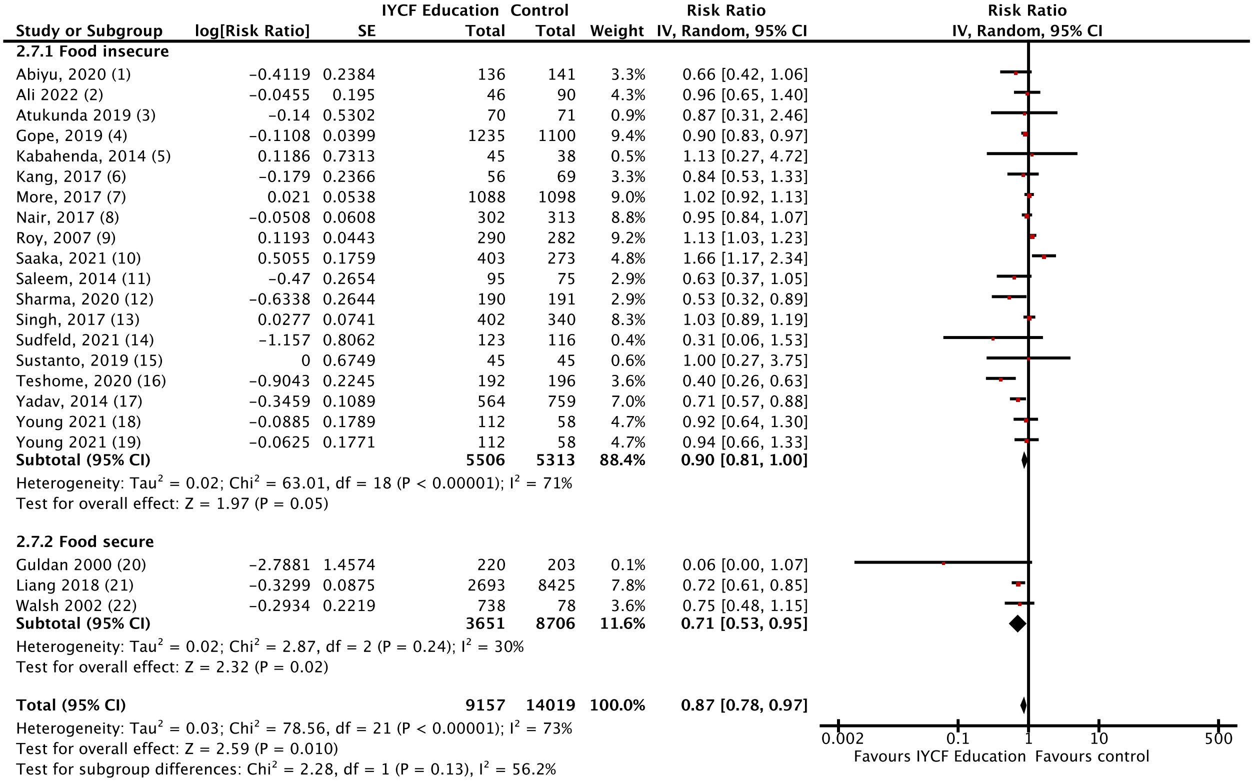

Footnotes
(1) cRCT; African Region; IYCF vs control; children aged 12–24 months; Intervention for 9 months; prevalence of underweight (WAZ<−2)
(2) RCT, Somalia, Africa, nutrition couselling, IDP children
(3) cRCT; African Region; IYCF vs control; Children aged 6–8 months; Follow-up at 20–24 months; prevalence of underweight (WAZ<−2)
(4) non–RCT; South–East Asia; PLA vs control; children aged 6 months–3 years; Intervention for 24 months; Prevalence of underweight (WAZ< −2);
(5) non–RCT; African Region; Structured nutrition education programme vs control; children aged 6–48 months; Intervention for 9 months; incidence of underweight..
(6) cRCT; African Region; IYCF [community-based participatory nutrition promotion (CPNP)'] vs control; children aged 6–12 months; intervention for 12 months;...
(7) cRCT; South East Asia; integrated activities vs control; children aged 5 years; prevalence of underweight (WAZ<−2)
(8) cRCT; South East Asia; support feeding, hygiene, care, and stimulation vs control; children aged 0–18 months; Intervention for 18 months; prevalence of...
(9) RCT; South East Asia; children aged 6–9 months; Follow-up 12 months; after intervention; proportion of children WAM <25% NCHS standard
(10) non–RCT; African Region; IYCF vs control; children aged 6–36 months; Intervention for 12 months Prevalence of underweight (WAZ < −2)
(11) cRCT; EMRO; IYCF vs control; children newborn; Intervention for 10 weeks; Prevalence of underweight (WAZ<−2)
(12) non–RCT; South East Asia; nutritional education arm vs control; children aged 4–6 months; Intervention for 6 months; prevalence of underweight (WAZ<−2)
(13) Integrated Nutrition and Health Program (INHP II); qRCT; South East Asia; children aged 6 months to 6 years; endline at 18 momths
(14) cRCT; African Region; integrated health nutrition and responsive stimulation vs control; children <1 years; Intervention for 18 months; Prevalence of underweight
(15) cRCT; South East Asia; growth, nutrition and development intervention vs control; children aged 0–72 months; Intervention for 12 weeks; prevalence of...
(16) cRCT; African Region; Pulse education nutrition program vs control; children aged 6–15 months; Follow-up 9 month; prevalence of underweight (WAZ<−2)
(17) non–RCT; South East Asia; IYCF vs control; children aged <3 years; intervention for 12 months; prevalence of underweight (WAZ<−2)
(18) cRCT; Cambodia, Western Pacific; traditional Positive Deviance/Hearth programme (PDH); prevalence at 3 months followup
(19) cRCT; Cambodia, Western Pacific; traditional Positive Deviance/Hearth with Interactive Voice Calling (PDH-IVC);prevalence at 3 months followup
(20) non–RCT; China; 4–12 months; intervention for 1 year
(21) non–RCT; Western Pacific region; IYCF vs control; children <3 years; Intervention for 4 years; prevalence of underweight (WAZ<−2)
(22) non–RCT; rural Africa; EBF vs. control for 6 months; intervention provided to mothers; prevalence of underweight (WAZ <−3 to <−2) at 2 years

Change in MUAC (z-score)

Study or Subgroup	Mean Difference	SE	Weight	Mean Difference IV, Random, 95% CI
**2.8.1 Food insecure**				
Atukunda 2019 (1)	−0.01	0.1225	36.6%	−0.01 [−0.25, 0.23]
Roy 2022 (2)	0.47	0.1516	32.4%	0.47 [0.17, 0.77]
**Subtotal (95% CI)**			**69.0%**	**0.22 [−0.25, 0.69]**
Heterogeneity: $Tau^2 = 0.10$; $Chi^2 = 6.06$, df = 1 (P = 0.01); $I^2 = 84\%$				
Test for overall effect: Z = 0.92 (P = 0.36)				
**2.8.2 Food secure**				
Olaya 2013 (3)	0.06	0.1617	31.0%	0.06 [−0.26, 0.38]
**Subtotal (95% CI)**			**31.0%**	**0.06 [−0.26, 0.38]**
Heterogeneity: Not applicable				
Test for overall effect: Z = 0.37 (P = 0.71)				
**Total (95% CI)**			**100.0%**	**0.17 [−0.13, 0.46]**
Heterogeneity: $Tau^2 = 0.05$; $Chi^2 = 6.47$, df = 2 (P = 0.04); $I^2 = 69\%$				
Test for overall effect: Z = 1.11 (P = 0.27)				
Test for subgroup differences: $Chi^2 = 0.31$, df = 1 (P = 0.58), $I^2 = 0\%$				

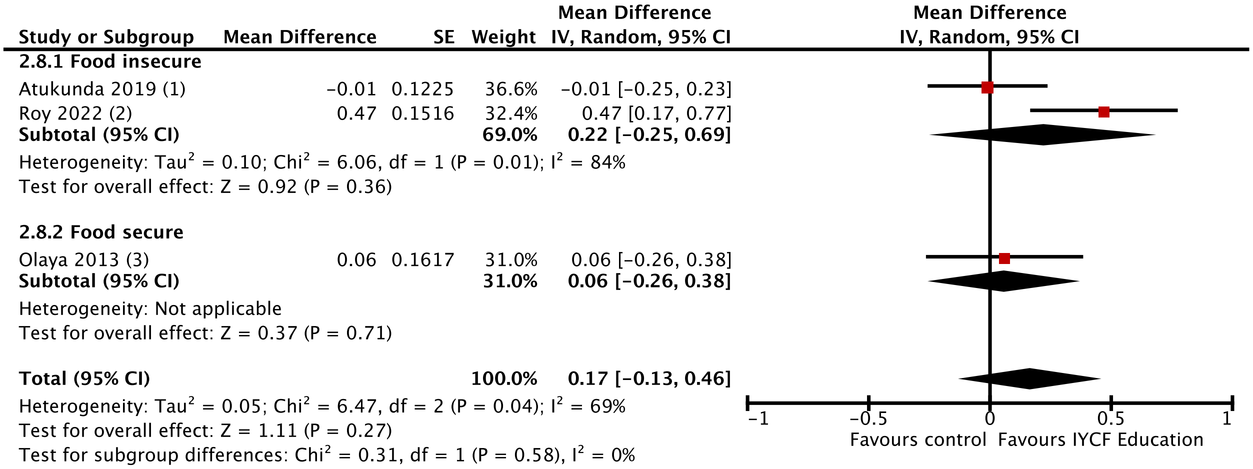

Footnotes

(1) z score; cRCT; African Region; IYCF vs control; Children aged 6–8 months; Follow-up at 20–24 months
(2) RCT, Bangladesh, Asia, 6–23 months, IYCF
(3) RCT, Region of Americas, children of 6months, change in MUAC-Z

Change in weight

Study or Subgroup	Mean Difference	SE	IYCF Education Total	Control Total	Weight	Mean Difference IV, Random, 95% CI
**2.10.1 Food insecure**						
Abiyu, 2020 (1)	0.46	0.0777	136	141	8.3%	0.46 [0.31, 0.61]
Bhandari, 2001 (2)	−0.06	0.0904	97	93	8.0%	−0.06 [−0.24, 0.12]
Bhandari, 2003 (3)	−0.01	0.0639	218	197	8.5%	−0.01 [−0.14, 0.12]
Kang, 2017 (4)	0.031	0.0061	76	79	9.2%	0.03 [0.02, 0.04]
Nayak 2016 (5)	0.736	0.4804	23	23	1.7%	0.74 [−0.21, 1.68]
Roy 2022 (6)	1.05	0.2037	96	96	5.2%	1.05 [0.65, 1.45]
Saleem, 2014 (7)	0.35	0.1527	95	75	6.4%	0.35 [0.05, 0.65]
Salehi, 2004 (8)	0.74	0.0879	406	405	8.0%	0.74 [0.57, 0.91]
Sharma, 1987 (9)	−0.19	0.1703	83	70	5.9%	−0.19 [−0.52, 0.14]
Sharma, 2020 (10)	0.16	0.083	190	191	8.1%	0.16 [−0.00, 0.32]
Singh, 2017 (11)	−0.1	0.081	402	340	8.2%	−0.10 [−0.26, 0.06]
Vazir, 2013 (12)	0.1	0.1649	117	61	6.1%	0.10 [−0.22, 0.42]
Vazir, 2013 (13)	−0.1	0.1693	102	60	6.0%	−0.10 [−0.43, 0.23]
**Subtotal (95% CI)**			**2041**	**1831**	**89.5%**	**0.20 [0.05, 0.35]**
Heterogeneity: $Tau^2 = 0.06$; $Chi^2 = 134.94$, df = 12 (P < 0.00001); $I^2 = 91\%$						
Test for overall effect: Z = 2.65 (P = 0.008)						
**2.10.2 Food secure**						
Penny 2005 (14)	0.29	0.2187	75	76	4.8%	0.29 [−0.14, 0.72]
Zhang, 2013 (15)	0.24	0.1839	80	73	5.6%	0.24 [−0.12, 0.60]
**Subtotal (95% CI)**			**155**	**149**	**10.5%**	**0.26 [−0.02, 0.54]**
Heterogeneity: $Tau^2 = 0.00$; $Chi^2 = 0.03$, df = 1 (P = 0.86); $I^2 = 0\%$						
Test for overall effect: Z = 1.85 (P = 0.06)						
**Total (95% CI)**			**2196**	**1980**	**100.0%**	**0.20 [0.07, 0.34]**
Heterogeneity: $Tau^2 = 0.05$; $Chi^2 = 137.49$, df = 14 (P < 0.00001); $I^2 = 90\%$						
Test for overall effect: Z = 2.93 (P = 0.003)						
Test for subgroup differences: $Chi^2 = 0.15$, df = 1 (P = 0.70), $I^2 = 0\%$						

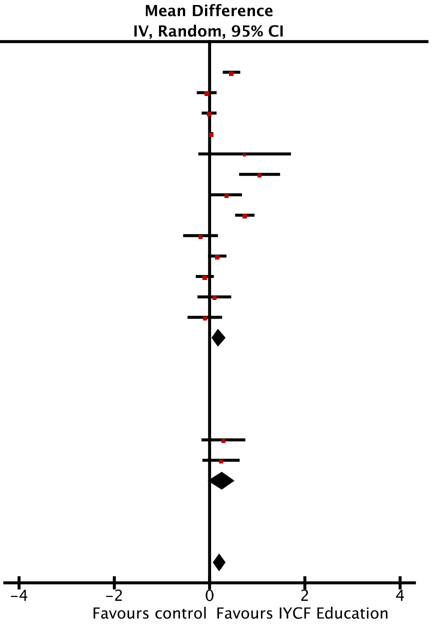

Footnotes

(1) weight gain; cRCT; African Region; IYCF vs control; children aged 12–24 months; Intervention for 9 months; study reported cluster adjusted estimates (DID)
(2) RCT; South East Asia; nutritional counselling vs control; children aged 4–12 months; Intervention for 12 months; weight change at 6 months
(3) cRCT; South East Asia; children aged 6–18 months; Intervention for 6 months; weight gain between 12–18 months
(4) change in weight/ month; cRCT; African Region; IYCF [community-based participatory nutrition promotion (CPNP)'] vs control; children aged 6–12 months;...
(5) family level counselling; cRCT; India; mother in their last trimester, follow-up at 24 months; g
(6) RCT, Bangladesh, Asia, 6–23 months, IYCF
(7) cRCT; EMRO; IYCF vs control; children newborn; Intervention for 10 weeks; weight in kgs at endline
(8) Weight; RCT; EMRO; environmental health, personal hygiene and nutritional edu vs control; children aged 0–59 months; Intervention for 12 months
(9) nutrition edu; interventional study; weight; South East Asia; Children aged 0–12 months; 9–12 months
(10) weight; non-RCT; South East Asia; nutritional education arm vs control; children aged 4–6 months; Intervention for 6 months
(11) Integrated Nutrition and Health Program (INHP II); qRCT; South East Asia; children aged 6 months to 6 years; endline at 18 momths
(12) kgs; CF education cRCT; South East Asia; children aged 3–15 months; Follow-up 15 months
(13) kgs; CF education+PG; cRCT; South East Asia; children aged 3–15 months; Follow-up 15 months
(14) cRCT; Peru; children aged 4 months; follow-up at 18 months, endline
(15) kgs; cRCT; Western Pacific Region; children at 6 months; at 18 months; change in wt

Change in height

Study or Subgroup	Mean Difference	SE	IYCF Education Total	Control Total	Weight	Mean Difference IV, Random, 95% CI
**2.9.1 Food insecure**						
Bhandari, 2001 (1)	−0.2	0.2252	97	93	2.2%	−0.20 [−0.64, 0.24]
Bhandari, 2003 (2)	−0.06	0.1924	218	197	3.0%	−0.06 [−0.44, 0.32]
Kang, 2017 (3)	0.059	0.0163	76	79	41.9%	0.06 [0.03, 0.09]
Roy 2022 (4)	2.29	0.71	96	96	0.2%	2.29 [0.90, 3.68]
Saleem, 2014 (5)	0.66	0.4997	95	75	0.5%	0.66 [−0.32, 1.64]
Salehi, 2004 (6)	0.0163	0.0035	406	405	46.0%	0.02 [0.01, 0.02]
Sharma, 2020 (7)	0.15	0.227	190	191	2.2%	0.15 [−0.29, 0.59]
Singh, 2017 (8)	−0.2	0.2586	402	340	1.7%	−0.20 [−0.71, 0.31]
Vazir, 2013 (9)	0.3	0.4344	102	60	0.6%	0.30 [−0.55, 1.15]
Vazir, 2013 (10)	0.5	0.4228	117	61	0.6%	0.50 [−0.33, 1.33]
**Subtotal (95% CI)**			**1799**	**1597**	**98.9%**	**0.04 [−0.02, 0.10]**
Heterogeneity: $Tau^2 = 0.00$; $Chi^2 = 22.32$, df = 9 (P = 0.008); $I^2 = 60\%$						
Test for overall effect: Z = 1.30 (P = 0.20)						
**2.9.2 Food secure**						
Penny 2005 (11)	1.07	0.4395	75	76	0.6%	1.07 [0.21, 1.93]
Zhang, 2013 (12)	0.69	0.5005	80	73	0.5%	0.69 [−0.29, 1.67]
**Subtotal (95% CI)**			**155**	**149**	**1.1%**	**0.90 [0.26, 1.55]**
Heterogeneity: $Tau^2 = 0.00$; $Chi^2 = 0.33$, df = 1 (P = 0.57); $I^2 = 0\%$						
Test for overall effect: Z = 2.74 (P = 0.006)						
**Total (95% CI)**			**1954**	**1746**	**100.0%**	**0.05 [−0.02, 0.12]**
Heterogeneity: $Tau^2 = 0.00$; $Chi^2 = 29.85$, df = 11 (P = 0.002); $I^2 = 63\%$						
Test for overall effect: Z = 1.43 (P = 0.15)						
Test for subgroup differences: $Chi^2 = 6.82$, df = 1 (P = 0.009), $I^2 = 85.3\%$						

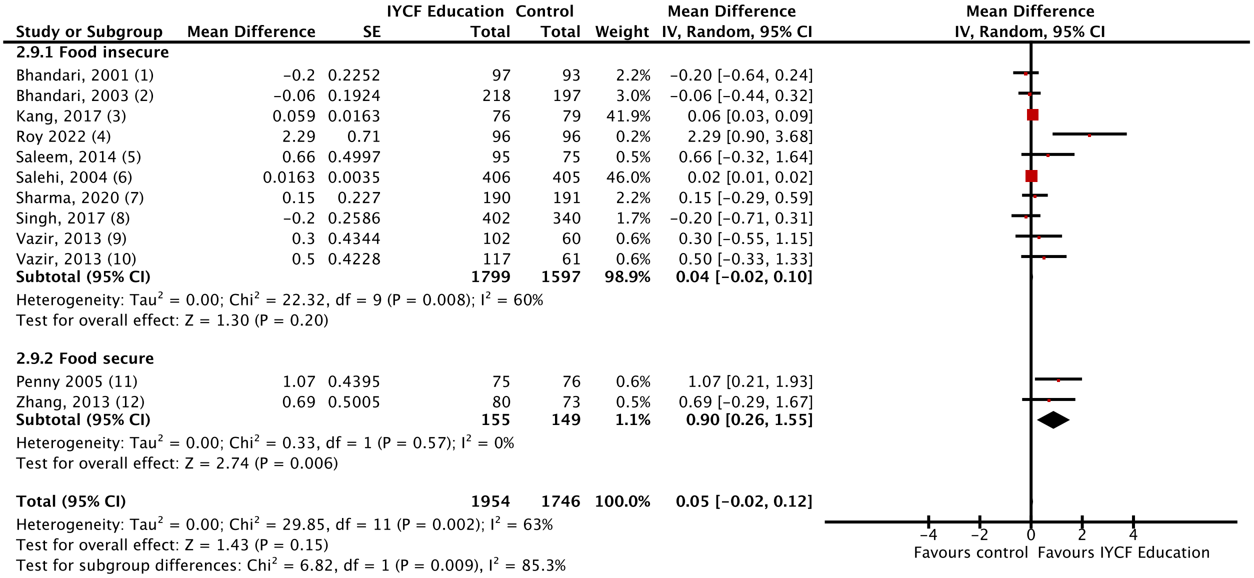

Footnotes
(1) RCT; South East Asia; nutritional counselling vs control; children aged 4–12 months; Intervention for 12 months; change in height
(2) cRCT; South East Asia; children aged 6–18 months; Intervention for 6 months; height gain at 12–18 months
(3) change in height/ month; cRCT; African Region; IYCF [community-based participatory nutrition promotion (CPNP)'] vs control; children aged 6–12 months;...
(4) RCT, Bangladesh, Asia, 6–23 months, IYCF
(5) cRCT; EMRO; IYCF vs control; children newborn; Intervention for 10 weeks; height in cm at endline
(6) RCT; EMRO; environmental health, personal hygiene and nutritional edu vs control; children aged 0–59 months; Intervention for 12 months; Height
(7) height; non-RCT; South East Asia; nutritional education arm vs control; children aged 4–6 months; Intervention for 6 months
(8) Integrated Nutrition and Health Program (INHP II); qRCT; South East Asia; children aged 6 months to 6 years; endline at 18 momths
(9) CF education+PG; cRCT; South East Asia; children aged 3–15 months; Follow-up 15 months
(10) CF education cRCT; South East Asia; children aged 3–15 months; Follow-up 15 months
(11) cRCT; Peru; children aged 4 months; follow-up at 18 months; endline
(12) kgs; cRCT; Western Pacific Region; children at 6 months; at 18 months; change in height

Hb levels

Study or Subgroup	Mean Difference	SE	Weight	Mean Difference IV, Random, 95% CI
**3.11.1 Food insecure**				
Fahmida, 2020 (1)	0.09	0.1296	25.4%	0.09 [-0.16, 0.34]
Kadiyala, 2021 (2)	-0.1	0.0983	27.0%	-0.10 [-0.29, 0.09]
Kadiyala, 2021 (3)	0.0625	0.1717	23.1%	0.06 [-0.27, 0.40]
**Subtotal (95% CI)**			**75.5%**	**-0.01 [-0.15, 0.13]**
Heterogeneity: $Tau^2 = 0.00$; $Chi^2 = 1.61$, df = 2 (P = 0.45); $I^2 = 0\%$				
Test for overall effect: Z = 0.20 (P = 0.84)				
**3.11.2 Food secure**				
Guldan 2000 (4)	3.5	1.0975	2.4%	3.50 [1.35, 5.65]
Olaya 2013 (5)	0.6	0.1884	22.1%	0.60 [0.23, 0.97]
**Subtotal (95% CI)**			**24.5%**	**1.85 [-0.97, 4.66]**
Heterogeneity: $Tau^2 = 3.58$; $Chi^2 = 6.78$, df = 1 (P = 0.009); $I^2 = 85\%$				
Test for overall effect: Z = 1.29 (P = 0.20)				
**Total (95% CI)**			**100.0%**	**0.23 [-0.12, 0.58]**
Heterogeneity: $Tau^2 = 0.11$; $Chi^2 = 20.69$, df = 4 (P = 0.0004); $I^2 = 81\%$				
Test for overall effect: Z = 1.28 (P = 0.20)				
Test for subgroup differences: $Chi^2 = 1.68$, df = 1 (P = 0.20), $I^2 = 40.4\%$				

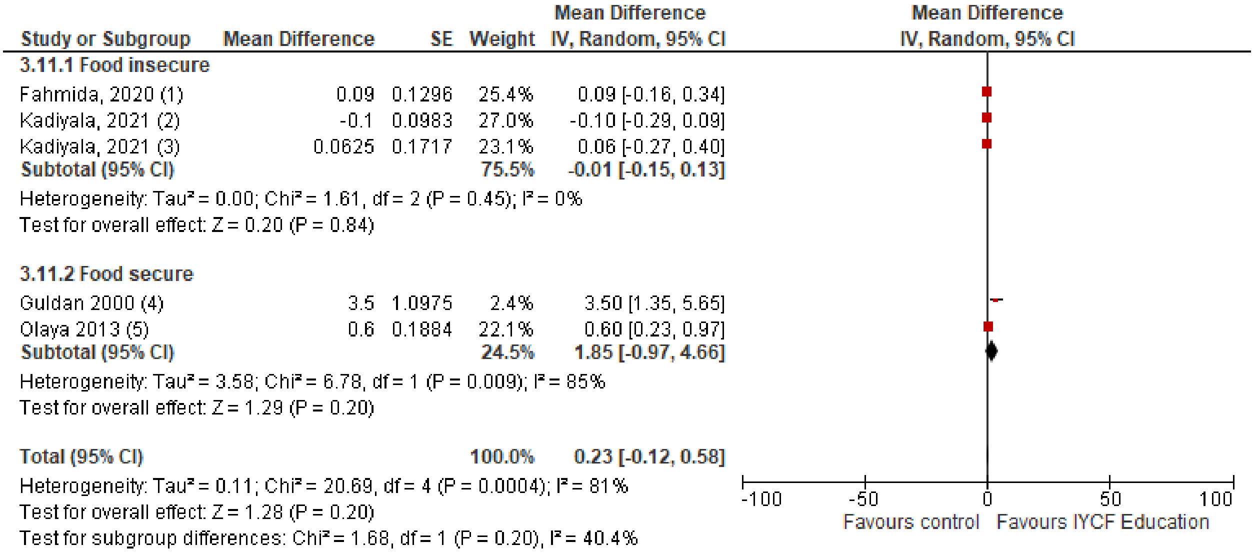

Footnotes
(1) integrated ICF; cRCT; Indonesia; children aged 0-18 months; follow-up 18 months
(2) cRCT; South East Asia; Nutrition+Agriculture vs agriculture; children aged 6-23 months; Intervention for 36 months
(3) cRCT; South East Asia; Nutrition+Agriculture+ PLA vs agriculture+Nutrition; children aged 6-23 months; Intervention for 36 months
(4) non-RCT; China; 4-12 months; intervention for 1 year; <110g/l
(5) RCT, Region of Americas, children of 6months, at endline

Anaemia

Study or Subgroup	log[Risk Ratio]	SE	Weight	Risk Ratio IV, Random, 95% CI
**3.12.1 Food insecure**				
Fahmida, 2020 (1)	-0.3038	0.088	35.4%	0.74 [0.62, 0.88]
**Subtotal (95% CI)**			**35.4%**	**0.74 [0.62, 0.88]**
Heterogeneity: Not applicable				
Test for overall effect: Z = 3.45 (P = 0.0006)				
**3.12.2 Food secure**				
Guldan 2000 (2)	-0.3836	0.1635	28.8%	0.68 [0.49, 0.94]
Vitolo 2005 (3)	0.1308	0.0827	35.8%	1.14 [0.97, 1.34]
**Subtotal (95% CI)**			**64.6%**	**0.90 [0.54, 1.49]**
Heterogeneity: Tau^2 = 0.12; Chi^2 = 7.88, df = 1 (P = 0.005); I^2 = 87%				
Test for overall effect: Z = 0.42 (P = 0.68)				
**Total (95% CI)**			**100.0%**	**0.84 [0.60, 1.18]**
Heterogeneity: Tau^2 = 0.08; Chi^2 = 16.13, df = 2 (P = 0.0003); I^2 = 88%				
Test for overall effect: Z = 1.00 (P = 0.32)				
Test for subgroup differences: Chi^2 = 0.53, df = 1 (P = 0.47), I^2 = 0%				

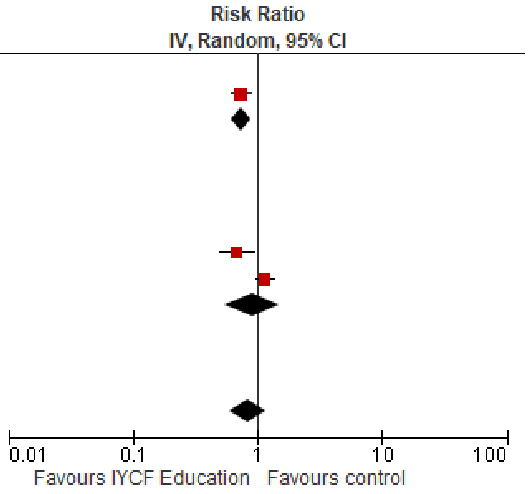

Footnotes
(1) integrated ICF; cRCT; Indonesia; children aged 0-18 months; follow-up 18 months
(2) non-RCT; China; 4-12 months; intervention for 1 year; <110g/l
(3) RCT, Region of the Americas, mothers of children 6-12 months, 2 visits for 12 months; Hb <11g/dl

Fever

Study or Subgroup	log[Risk Ratio]	SE	Weight	Risk Ratio IV, Random, 95% CI
**2.13.1 Food insecure**				
Abiyu, 2020 (1)	−0.0473	0.2627	4.1%	0.95 [0.57, 1.60]
Bhandari, 2003 (2)	0.0994	0.2402	4.9%	1.10 [0.69, 1.77]
Reinbott, 2016 (3)	−0.0867	0.0677	62.1%	0.92 [0.80, 1.05]
Roy 2022 (4)	−0.3857	0.2793	3.6%	0.68 [0.39, 1.18]
**Subtotal (95% CI)**			**74.8%**	**0.92 [0.81, 1.03]**
Heterogeneity: Tau^2 = 0.00; Chi^2 = 1.77, df = 3 (P = 0.62); I^2 = 0%				
Test for overall effect: Z = 1.41 (P = 0.16)				
**2.13.2 Food secure**				
Vitolo 2005 (5)	−0.0396	0.1062	25.2%	0.96 [0.78, 1.18]
**Subtotal (95% CI)**			**25.2%**	**0.96 [0.78, 1.18]**
Heterogeneity: Not applicable				
Test for overall effect: Z = 0.37 (P = 0.71)				
**Total (95% CI)**			**100.0%**	**0.93 [0.84, 1.03]**
Heterogeneity: Tau^2 = 0.00; Chi^2 = 1.92, df = 4 (P = 0.75); I^2 = 0%				
Test for overall effect: Z = 1.40 (P = 0.16)				
Test for subgroup differences: Chi^2 = 0.15, df = 1 (P = 0.70), I^2 = 0%				

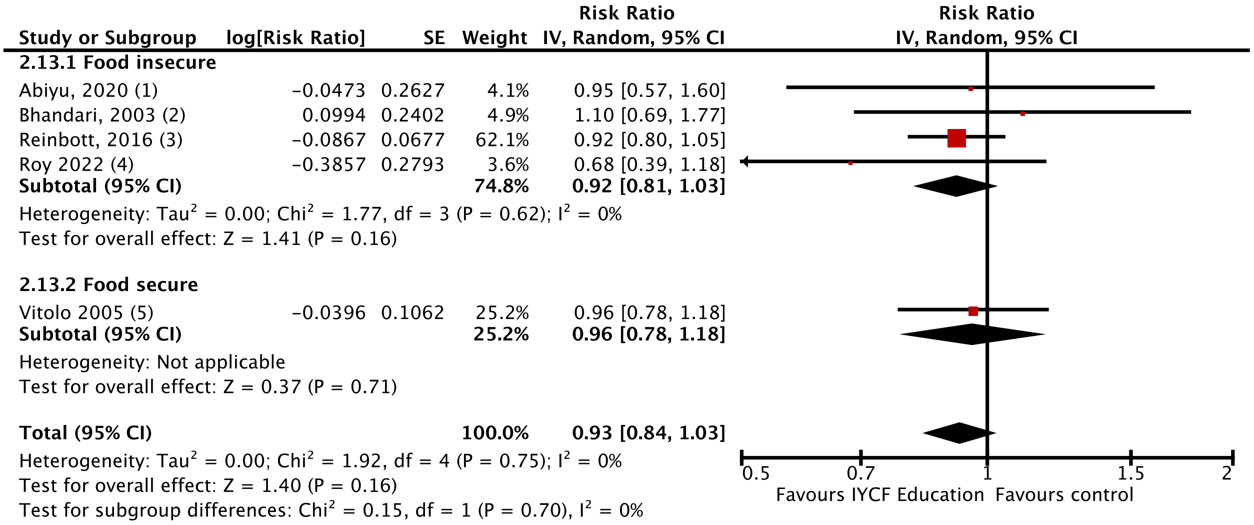

Footnotes
(1) cRCT; African Region; IYCF vs control; children aged 12–24 months; Intervention for 9 months
(2) RCT; South East Asia; IYCF vs control; children aged 6–18 months; Intervention for 12 months
(3) IYCG and agricultural activities; cRCT; Western Pacific; children aged 0–23 months; Follow-up at 2 years
(4) RCT, Bangladesh, Asia, 6–23 months, IYCF
(5) RCT, Region of the Americas, mothers of children 6–12 months, 2 visits for 12 months

Diarrhea

Study or Subgroup	log[Risk Ratio]	SE	Weight	Risk Ratio IV, Random, 95% CI	Risk Ratio IV, Random, 95% CI
**2.14.1 Food insecure**					
Abiyu, 2020 (1)	−0.1646	0.23	12.3%	0.85 [0.54, 1.33]	
Bhandari, 2003 (2)	0.2515	0.2363	11.9%	1.29 [0.81, 2.04]	
Reinbott, 2016 (3)	0.0545	0.1889	16.1%	1.06 [0.73, 1.53]	
Roy 2022 (4)	−0.2744	0.268	9.8%	0.76 [0.45, 1.29]	
Sinharoy 2017 (5)	0.0045	0.1042	28.5%	1.00 [0.82, 1.23]	
**Subtotal (95% CI)**			**78.6%**	**1.00 [0.86, 1.16]**	
Heterogeneity: $Tau^2 = 0.00$; $Chi^2 = 2.78$, df = 4 (P = 0.60); $I^2 = 0\%$					
Test for overall effect: Z = 0.04 (P = 0.97)					
**2.14.2 Food secure**					
Vitolo 2005 (6)	−0.3948	0.1467	21.4%	0.67 [0.51, 0.90]	
**Subtotal (95% CI)**			**21.4%**	**0.67 [0.51, 0.90]**	
Heterogeneity: Not applicable					
Test for overall effect: Z = 2.69 (P = 0.007)					
**Total (95% CI)**			**100.0%**	**0.91 [0.76, 1.10]**	
Heterogeneity: $Tau^2 = 0.02$; $Chi^2 = 8.39$, df = 5 (P = 0.14); $I^2 = 40\%$					
Test for overall effect: Z = 0.96 (P = 0.34)					
Test for subgroup differences: $Chi^2 = 5.61$, df = 1 (P = 0.02), $I^2 = 82.2\%$					

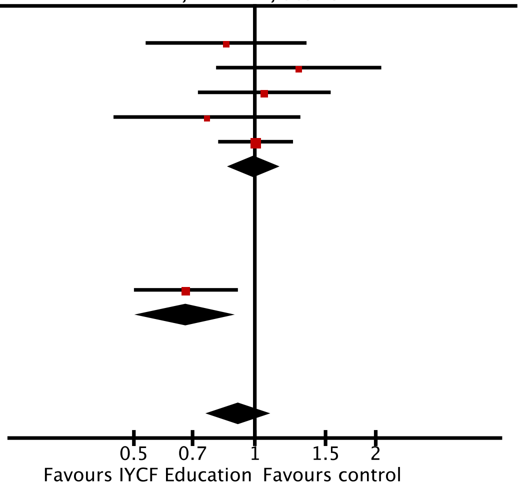

Footnotes

(1) cRCT; African Region; IYCF vs control; children aged 12–24 months; Intervention for 9 months
(2) cRCT; South East Asia; IYCF vs visitation; children aged 6–18 months; Intervention for 12 months; Diarrhea was defined as passage...
(3) IYCG and agricultural activities; cRCT; Western Pacific; children aged 0–23 months; Follow-up at 2 years
(4) RCT, Bangladesh, Asia, 6–23 months, IYCF
(5) cRCT; Western Rwanda; Classic intervention intervention vs control; children <5 years; Intervention for 2 years
(6) RCT, Region of the Americas, mothers of children 6–12 months, 2 visits for 12 months

Respiratory illness

Study or Subgroup	log[Risk Ratio]	SE	Weight	Risk Ratio IV, Random, 95% CI	Risk Ratio IV, Random, 95% CI
**2.15.1 Food insecure**					
Abiyu, 2020 (1)	−0.118	0.3746	7.3%	0.89 [0.43, 1.85]	
Bhandari, 2003 (2)	−0.0537	0.1949	23.2%	0.95 [0.65, 1.39]	
Reinbott, 2016 (3)	−0.2019	0.2003	22.2%	0.82 [0.55, 1.21]	
Roy 2022 (4)	−0.6325	0.2631	14.0%	0.53 [0.32, 0.89]	
**Subtotal (95% CI)**			**66.8%**	**0.80 [0.62, 1.01]**	
Heterogeneity: $Tau^2 = 0.01$; $Chi^2 = 3.27$, df = 3 (P = 0.35); $I^2 = 8\%$					
Test for overall effect: Z = 1.85 (P = 0.06)					
**2.15.2 Food secure**					
Vitolo 2005 (5)	−0.4651	0.1543	33.2%	0.63 [0.46, 0.85]	
**Subtotal (95% CI)**			**33.2%**	**0.63 [0.46, 0.85]**	
Heterogeneity: Not applicable					
Test for overall effect: Z = 3.01 (P = 0.003)					
**Total (95% CI)**			**100.0%**	**0.73 [0.60, 0.90]**	
Heterogeneity: $Tau^2 = 0.01$; $Chi^2 = 4.80$, df = 4 (P = 0.31); $I^2 = 17\%$					
Test for overall effect: Z = 2.95 (P = 0.003)					
Test for subgroup differences: $Chi^2 = 1.43$, df = 1 (P = 0.23), $I^2 = 29.9\%$					

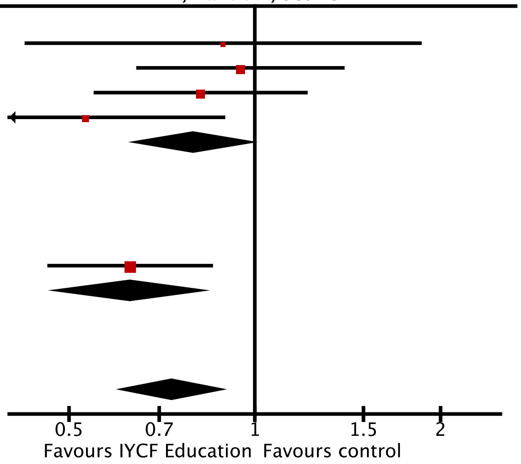

Footnotes

(1) cRCT; African Region; IYCF vs control; children aged 12–24 months; Intervention for 9 months; cough
(2) cRCT; South East Asia; IYCF vs control; children aged 6–18 months; Intervention for 12 months; cough
(3) IYCG and agricultural activities; cRCT; Western Pacific; children aged 0–23 months; Follow-up at 2 years; ARI
(4) RCT, Bangladesh, Asia, 6–23 months, IYCF, cold
(5) RCT, Region of the Americas, mothers of children 6–12 months, 2 visits for 12 months; breathing problems

Mortality

Study or Subgroup	log[Risk Ratio]	SE	Weight	Risk Ratio IV, Random, 95% CI
**3.16.1 Food insecure**				
Atukunda 2019 (1)	-0.4614	0.908	1.8%	0.63 [0.11, 3.74]
Bhandari, 2003 (2)	0.5716	0.2915	13.4%	1.77 [1.00, 3.14]
Fahmida, 2020 (3)	-0.0043	1.4111	0.8%	1.00 [0.06, 15.82]
Kang, 2017 (4)	-1.0489	1.6269	0.6%	0.35 [0.01, 8.50]
Nair, 2017 (5)	-0.2141	0.2956	13.2%	0.81 [0.45, 1.44]
Roy, 2007 (6)	-0.6964	1.2221	1.0%	0.50 [0.05, 5.47]
Sharma, 1987 (7)	0.6334	0.4158	7.6%	1.88 [0.83, 4.26]
Singh, 2017 (8)	-0.1783	0.0651	46.8%	0.84 [0.74, 0.95]
Sudfeld, 2021 (9)	-1.1291	0.8071	2.2%	0.32 [0.07, 1.57]
Yousafzai 2014 (10)	0.1046	0.3526	10.0%	1.11 [0.56, 2.22]
**Subtotal (95% CI)**			**97.3%**	**0.99 [0.75, 1.31]**
Heterogeneity: Tau² = 0.05; Chi² = 12.44, df = 9 (P = 0.19); I² = 28%				
Test for overall effect: Z = 0.04 (P = 0.97)				
**3.16.2 Food secure**				
Navarro 2013 (11)	-0.6797	0.8326	2.1%	0.51 [0.10, 2.59]
Penny 2005 (12)	-1.0855	1.625	0.6%	0.34 [0.01, 8.16]
**Subtotal (95% CI)**			**2.7%**	**0.47 [0.11, 1.99]**
Heterogeneity: Tau² = 0.00; Chi² = 0.05, df = 1 (P = 0.82); I² = 0%				
Test for overall effect: Z = 1.03 (P = 0.30)				
**Total (95% CI)**			**100.0%**	**0.96 [0.75, 1.22]**
Heterogeneity: Tau² = 0.03; Chi² = 13.22, df = 11 (P = 0.28); I² = 17%				
Test for overall effect: Z = 0.35 (P = 0.72)				
Test for subgroup differences: Chi² = 1.01, df = 1 (P = 0.31), I² = 1.0%				

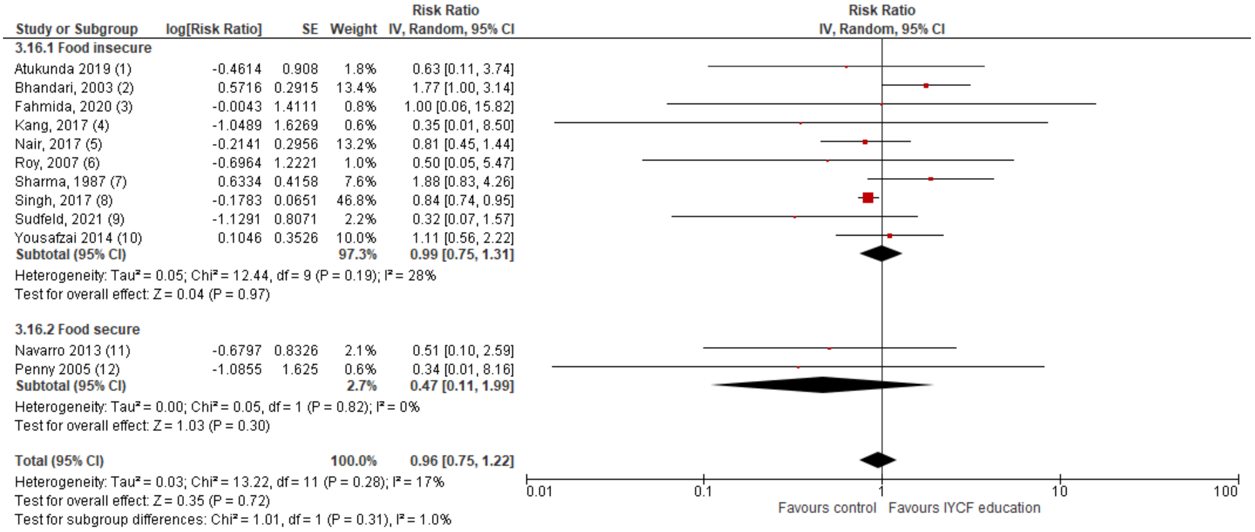

Footnotes

(1) cRCT; African Region; IYCF vs control; Children aged 6-8 months; Follow-up at 20-24 months; under 5 mortality
(2) cRCT; South East Asia; children aged 6-18 months; Intervention for 6 months; under 5 mortality
(3) integrated ICF; cRCT; Indonesia; children aged 0-18 months; follow-up 18 months; under 5 mortality
(4) cRCT; African Region; IYCF [community-based participatory nutrition promotion (CPNP)] vs control; children aged 6-12 months; under 5 mortality
(5) cRCT; South East Asia; support feeding, hygiene, care, and stimulation vs control; children aged 0-18 months; Intervention for 18 months; infant deaths
(6) RCT; South East Asia; children aged 6-9 months; Follow-up 12 months; under 5 mortality
(7) cRCT; nutrition edu; interventional study; weight; South East Asia; Children aged 0-12 months; 9-12 months
(8) Integrated Nutrition and Health Program (INHP II); qRCT; South East Asia; children aged 6 months to 6 years; Follow-up 1 months; under 5 mortality
(9) cRCT; African Region; integrated health nutrition and responsive stimulation vs control; children <1 years; Intervention for 18 months; under 5 mortality
(10) cRCT; Pakistan, enhanced nutrition education, children upto 24 months of age; under 5 mortality
(11) non-RCT, Regions of America, IYCF vs control; children <2 years; Intervention for 24 months; under 5 mortality
(12) cRCT; Peru; children aged 4 months; follow-up at 18 months; under 5 mortality

Comparison 5: Complementary food education versus control (among malnourished children)

WHZ

Study or Subgroup	Mean Difference	SE	Experimental Total	Control Total	Weight	Mean Difference IV, Random, 95% CI
Seyyedi 2020 (1)	0.34	0.0373	50	50	100.0%	0.34 [0.27, 0.41]
**Total (95% CI)**			**50**	**50**	**100.0%**	**0.34 [0.27, 0.41]**
Heterogeneity: Not applicable						
Test for overall effect: Z = 9.12 (P < 0.00001)						

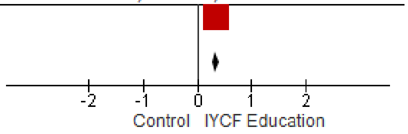

Footnotes
(1) RCT; EMRO; smartphone-based maternal nutrition vs treatment as usual; children <3 years; Intervention for 6 months; WHZ at endline

WAZ

Study or Subgroup	Mean Difference	SE	Experimental Total	Control Total	Weight	Mean Difference IV, Random, 95% CI
Seyyedi 2020 (1)	0.35	0.0281	50	50	100.0%	0.35 [0.29, 0.41]
**Total (95% CI)**			**50**	**50**	**100.0%**	**0.35 [0.29, 0.41]**
Heterogeneity: Not applicable						
Test for overall effect: Z = 12.46 (P < 0.00001)						

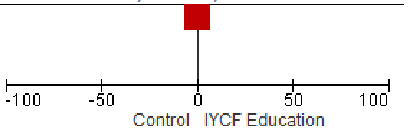

Footnotes
(1) RCT; EMRO; smartphone-based maternal nutrition vs treatment as usual; children <3 years; Intervention for 6 months; WAZ at endline

HAZ

Study or Subgroup	Mean Difference	SE	Experimental Total	Control Total	Weight	Mean Difference IV, Random, 95% CI
Seyyedi 2020 (1)	0.35	0.029	50	50	100.0%	0.35 [0.29, 0.41]
**Total (95% CI)**			**50**	**50**	**100.0%**	**0.35 [0.29, 0.41]**
Heterogeneity: Not applicable						
Test for overall effect: Z = 12.07 (P < 0.00001)						

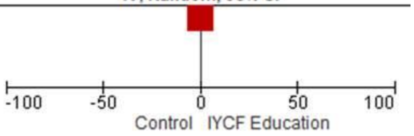

Footnotes
(1) RCT; EMRO; smartphone-based maternal nutrition vs treatment as usual; children <3 years; Intervention for 6 months; WAZ at endline
