# Supplementary material for: Dietary Strategies for Complementary Feeding between 6 and 24 Months of Age: The Evidence
Source: Nutrients. 2023 Jul 5;15(13):3041. doi: 10.3390/nu15133041 (PMC10346638; doi:10.3390/nu15133041)

SUPPLEMENTARY DOCUMENT S4 – Effectiveness of Micronutrient Powders (MNP) in children aged 6-24 months (unpublished)

Authors: Rehana A Salam, Jai K Das, Zohra S Lassi

Table of Contents

S4.1. Objective ***.......... 2***

S4.2. Methods ***.......... 2***

S4.3. Prisma diagram ***.......... 3***

S4.4. List of publications included ***.......... 4***

S4.5. Summary of findings tables ***.......... 5***

S4.6. Forest plots ***.......... 6***

S4.1. Objective

To summarize the effect of MNPs on the health outcomes of children aged 6-24 months.

S4.2. Methods

S4.2.1. Criteria for considering studies for this review

MNPs were identified as point-of-use powders with two or more micronutrients in their formulation. Studies were included that provided MNPs either in the home or at designated centers, using different multiple micronutrient formulations, with different dosages and duration. Studies that included supporting interventions such as nutrition education were included only if the supporting interventions were given to both the intervention and comparison groups, so that the difference between the two groups was solely of MNPs. Because of the unique nature of this intervention and a need to do a separate analysis specifically for this intervention, we excluded studies examining the impact of supplementary food provision, lipid-based supplements, micronutrient crushable tablets or foodlets, fortified milk or complementary foods and other fortified foods and beverages including fortified seasoning powders.

S4.2.2. Search methods for identification of studies

We systematically reviewed literature published up to November 2012 to identify studies describing the effectiveness of MNPs. An update to this search was performed on August 12, 2022. Following CHERG Systematic Review Guidelines, we searched PubMed, Cochrane Libraries, Embase, and WHO Regional Databases to identify all published and unpublished trials. Additional studies were identified by hand searching references from included studies. Search terms included combinations of Micronutrient* OR 'multiple micronutrient" OR "multi-vitamin" OR "multi-mineral" OR "micronutrient powder" OR MNP OR sprinkle AND Fortifi* OR "food fortifi*" OR "point of use" OR "home fortification". No language or date restrictions were applied in the
searches.

S4.2.3. Data collection and analysis

We abstracted data describing study identifiers and context, study design and limitations, intervention specifics and outcome effects into a standardized abstraction form for studies that met the final inclusion criteria as detailed in the CHERG Systematic Review Guidelines. Outcomes of interest included hematological; anemia, hemoglobin levels, serum micronutrient levels, anthropometric; stunting, wasting, underweight, weight for age z-score (WAZ), height for age z- score (HAZ), weight for height z-score (WHZ), head circumference and morbidity; diarrhea, upper respiratory infections (URI), fever and mortality among children aged 6-24 months. Each study was assessed and graded according to the CHERG adaptation of the GRADE technique.

We conducted a meta-analysis for individual studies and pooled statistics were reported as the relative risk (RR) for categorical variables and standard mean difference (SMD) for continuous variables between the experimental and control groups with 95% confidence intervals (CI). Mantel–Haenszel pooled RR and corresponding 95% CI were reported or the DerSimonian–Laird pooled RR and corresponding 95% CI where there was an unexplained heterogeneity. All analyses were conducted using the software Review Manager 5.1. Heterogeneity was quantified by Chi^2 and I^2, which can be interpreted as the percentage of the total variation between studies that is attributable to heterogeneity rather than to chance, a low p-value (less than 0.1) or a large chi squared statistic relative to its degree of freedom and I^2 values greater than 50% were taken as substantial and high heterogeneity. In situations of high heterogeneity, causes were explored by sensitivity analysis and random effect models were used.

We summarized the evidence by outcome, including qualitative assessments of study quality and quantitative measures, according to the standard guidelines. A grade of "high", "moderate", "low" and "very low" was used for grading the overall evidence indicating the strength of an effect on specific health outcome according to the CHERG Rules for Evidence Review.

S4.3. Prisma diagram

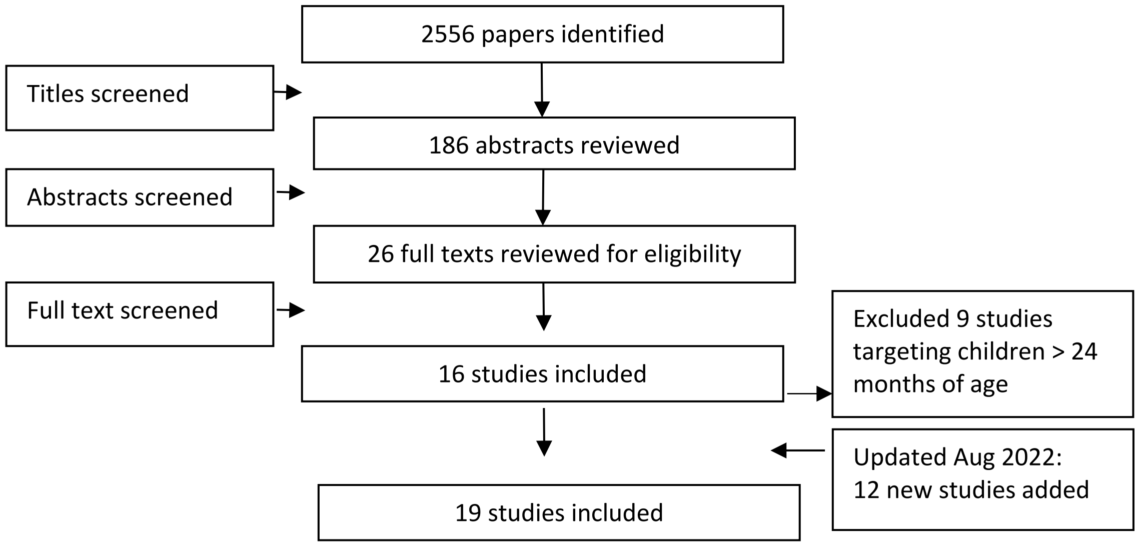

S4.4. List of publications included

#	Citation
1	Aboud FE, Akhter S. A cluster-randomized evaluation of a responsive stimulation and feeding intervention in Bangladesh. Pediatrics. 2011;127(5):e1191-e1197.
2	Albelbeisi A, Shariff ZM, Mun CY, Rahman HA, Abed Y. Multiple micronutrient supplementation improves growth and reduces the risk of anemia among infants in Gaza Strip, Palestine: a prospective randomized community trial. Nutr J. 2020;19(1):133.
3	Attanasio OP, Fernández C, Fitzsimons EO, Grantham-McGregor SM, Meghir C, Rubio-Codina M. Using the infrastructure of a conditional cash transfer program to deliver a scalable integrated early child development program in Colombia: cluster randomized controlled trial. Bmj. 2014;349.
4	Barffour MA, Hinnouho GM, Kounnavong S, et al. Effects of Daily Zinc, Daily Multiple Micronutrient Powder, or Therapeutic Zinc Supplementation for Diarrhea Prevention on Physical Growth, Anemia, and Micronutrient Status in Rural Laotian Children: A Randomized Controlled Trial. J Pediatr. 2019;207:80-89.e82.
5	Ghosh SA, Strutt NR, Otoo GE, et al. A macro- and micronutrient-fortified complementary food supplement reduced acute infection, improved haemoglobin and showed a dose-response effect in improving linear growth: a 12-month cluster randomised trial. J Nutr Sci. 2019;8:e22.
6	Inayati DA, Scherbaum V, Purwestri RC, et al. Combined intensive nutrition education and micronutrient powder supplementation improved nutritional status of mildly wasted children on Nias Island, Indonesia. Asia Pac J Clin Nutr. 2012;21(3):361-373.
7	Islam MM, Black RE, Krebs NF, et al. Different doses, forms, and frequencies of zinc supplementation for the prevention of diarrhea and promotion of linear growth among young Bangladeshi children: a six-arm, randomized, community-based efficacy trial. The Journal of Nutrition. 2022;152(5):1306-1315.
8	Larson LM, Young MF, Bauer PJ, et al. Effectiveness of a home fortification programme with multiple micronutrients on infant and young child development: a cluster-randomised trial in rural Bihar, India. Br J Nutr. 2018;120(2):176-187.
9	Matias SL, Mridha MK, Young RT, et al. Prenatal and postnatal supplementation with lipid-based nutrient supplements reduces anemia and iron deficiency in 18-month-old Bangladeshi children: a cluster-randomized effectiveness trial. The Journal of nutrition. 2018;148(7):1167-1176.
10	Mridha MK, Matias SL, Chaparro CM, et al. Lipid-based nutrient supplements for pregnant women reduce newborn stunting in a cluster-randomized controlled effectiveness trial in Bangladesh. The American journal of clinical nutrition. 2016;103(1):236-249.
11	Sazawal S, Dhingra P, Dhingra U, et al. Compliance with home-based fortification strategies for delivery of iron and zinc: its effect on haematological and growth markers

	among 6-24 months old children in north India. J Health Popul Nutr. 2014;32(2):217-226.
12	Somassè YE, Dramaix M, Traoré B, et al. The WHO recommendation of home fortification of foods with multiple-micronutrient powders in children under 2 years of age and its effectiveness on anaemia and weight: a pragmatic cluster-randomized controlled trial. Public Health Nutr. 2018;21(7):1350-1358.
13	Soofi S, Cousens S, Iqbal SP, et al. Effect of provision of daily zinc and iron with several micronutrients on growth and morbidity among young children in Pakistan: a cluster-randomised trial. Lancet. 2013;382(9886):29-40.
14	Adu-Afarwuah S, Lartey A, Brown KH, Zlotkin S, Briend A, Dewey KG. Randomized comparison of 3 types of micronutrient supplements for home fortification of complementary foods in Ghana: effects on growth and motor development. The American journal of clinical nutrition. 2007;86(2):412-420.
15	Agostoni C, Giovannini M, Sala D, et al. Double-blind, placebo-controlled trial comparing effects of supplementation of two micronutrient sprinkles on fatty acid status in Cambodian infants. Journal of pediatric gastroenterology and nutrition. 2007;44(1):136-142.
16	Giovannini M, Sala D, Usuelli M, et al. Double-blind, placebo-controlled trial comparing effects of supplementation with two different combinations of micronutrients delivered as sprinkles on growth, anemia, and iron deficiency in Cambodian infants. Journal of pediatric gastroenterology and nutrition. 2006;42(3):306-312.
17	Jack SJ, Ou K, Chea M, et al. Effect of micronutrient sprinkles on reducing anemia: a cluster-randomized effectiveness trial. Archives of pediatrics & adolescent medicine. 2012;166(9):842-850.
18	Menon P, Ruel MT, Loechl CU, et al. Micronutrient Sprinkles reduce anemia among 9-to 24-mo-old children when delivered through an integrated health and nutrition program in rural Haiti. The Journal of nutrition. 2007;137(4):1023-1030.
19	Sharieff W, Bhutta Z, Schauer C, Tomlinson G, Zlotkin S. Micronutrients (including zinc) reduce diarrhoea in children: the Pakistan Sprinkles Diarrhoea Study. Archives of disease in childhood. 2006;91(7):573-579.

S4.5. Summary of findings tables

MNPs compared to no intervention/placebo for children upto 24 months of age

Patient or population: Apparently healthy children up to 24 months of age
Setting: Bangladesh, Brazil, Burkina Faso, Cambodia, China, Colombia, Ethiopia, Ghana, Haiti, India, Indonesia, Kenya, Kyrgyzstan, Lao People's Democratic Republic, Mali, Pakistan, Philippines, Uganda, Nepal
Intervention: MNPs
Comparison: No intervention/placebo

Outcomes	Anticipated absolute effects* (95% CI)		Relative effect (95% CI)	№ of participants (studies)	Certainty of the evidence (GRADE)	Comments
	Risk with no intervention/placebo	Risk with MNPs				
Anemia (GIV)	0 per 1,000	0 per 1,000 (0 to 0)	**RR 0.80** (0.64 to 1.01)	7290 (10 RCTs)	⨁⨁⨁◯ Moderate[a]	

MNPs compared to no intervention/placebo for children upto 24 months of age

Patient or population: Apparently healthy children up to 24 months of age
Setting: Bangladesh, Brazil, Burkina Faso, Cambodia, China, Colombia, Ethiopia, Ghana, Haiti, India, Indonesia, Kenya, Kyrgyzstan, Lao People's Democratic Republic, Mali, Pakistan, Philippines, Uganda, Nepal
Intervention: MNPs
Comparison: No intervention/placebo

Outcomes	Anticipated absolute effects* (95% CI)		Relative effect (95% CI)	№ of participants (studies)	Certainty of the evidence (GRADE)	Comments
	Risk with no intervention/placebo	Risk with MNPs				
Hemoglobin	-	SMD 0.72 higher (0.22 higher to 1.22 higher)	-	9089 (15 RCTs)	⊕⊕◯◯ Low[a,b]	
WAZ	The mean WAZ was 0	MD 0.11 higher (0.02 higher to 0.2 higher)	-	8253 (10 RCTs)	⊕⊕◯◯ Low[a,b]	
HAZ	The mean HAZ was 0	MD 0.05 higher (0.01 lower to 0.11 higher)	-	8253 (10 RCTs)	⊕⊕◯◯ Low[a,b]	
WHZ	The mean WHZ was 0	MD 0.08 higher (0.03 higher to 0.14 higher)	-	8065 (9 RCTs)	⊕⊕◯◯ Low[a,b]	
Underweight (GIV)	0 per 1,000	0 per 1,000 (0 to 0)	**RR 1.00** (0.95 to 1.05)	10128 (7 RCTs)	⊕⊕⊕◯ Moderate[b]	
Stunting(GIV)	0 per 1,000	0 per 1,000 (0 to 0)	**RR 1.02** (0.97 to 1.07)	9905 (7 RCTs)	⊕⊕⊕◯ Moderate[b]	
Wasting (GIV)	0 per 1,000	0 per 1,000 (0 to 0)	**RR 0.99** (0.90 to 1.08)	9901 (7 RCTs)	⊕⊕⊕◯ Moderate[b]	
Diarrhea (GIV)	0 per 1,000	0 per 1,000 (0 to 0)	**RR 1.08** (0.95 to 1.23)	6438 (4 RCTs)	⊕⊕◯◯ Low[b,c]	
URI (GIV)	0 per 1,000	0 per 1,000 (0 to 0)	**RR 0.90** (0.78 to 1.03)	1872 (2 RCTs)	⊕⊕◯◯ Low[b,c]	

***The risk in the intervention group** (and its 95% confidence interval) is based on the assumed risk in the comparison group and the **relative effect** of the intervention (and its 95% CI).

CI: confidence interval; **MD:** mean difference; **RR:** risk ratio; **SMD:** standardised mean difference

GRADE Working Group grades of evidence
High certainty: we are very confident that the true effect lies close to that of the estimate of the effect.
Moderate certainty: we are moderately confident in the effect estimate: the true effect is likely to be close to the estimate of the effect, but there is a possibility that it is substantially different.
Low certainty: our confidence in the effect estimate is limited: the true effect may be substantially different from the estimate of the effect.
Very low certainty: we have very little confidence in the effect estimate: the true effect is likely to be substantially different from the estimate of effect.

Explanations
a. Downgraded by one level due to high heterogeneity
b. Downgraded by one level due to study limitations
c. Downgraded by one level due to small number of events

S4.6. Forest plots

1. Outcome: Anemia

Study or Subgroup	log[Risk Ratio]	SE	Experimental Total	Control Total	Weight	Risk Ratio IV, Random, 95% CI
Adu-Afarwuah 2007	-0.5642	0.2592	98	96	7.9%	0.57 [0.34, 0.95]
Albelbeisi 2020	-1.0609	0.2341	100	100	8.5%	0.35 [0.22, 0.55]
Attanasio 2014	-0.2838	0.1461	316	321	10.9%	0.75 [0.57, 1.00]
Barffour 2019	-0.0808	0.0634	689	726	12.7%	0.92 [0.81, 1.04]
Giovannini 2006	-0.6126	0.1767	65	62	10.1%	0.54 [0.38, 0.77]
Jack 2012	-0.1744	0.093	443	457	12.1%	0.84 [0.70, 1.01]
Larson 2018	0.084	0.0235	1399	1406	13.1%	1.09 [1.04, 1.14]
Matias 2018	1.0599	0.1958	299	272	9.5%	2.89 [1.97, 4.24]
Menon 2007	-0.5773	0.1424	254	161	11.0%	0.56 [0.42, 0.74]
Sharieff 2006	-0.1823	0.4613	13	13	4.2%	0.83 [0.34, 2.06]
**Total (95% CI)**			**3676**	**3614**	**100.0%**	**0.80 [0.64, 1.01]**

Heterogeneity: $Tau^2 = 0.10$; $Chi^2 = 103.85$, df = 9 (P < 0.00001); $I^2 = 91\%$
Test for overall effect: Z = 1.89 (P = 0.06)

Risk Ratio
IV, Random, 95% CI

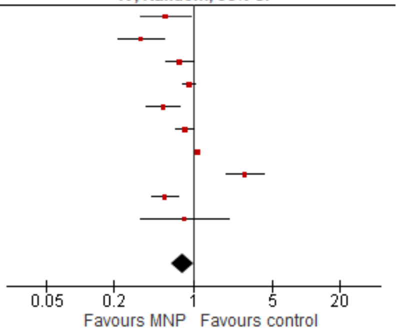

2. Outcome: Hemoglobin

Study or Subgroup	Experimental Mean	Experimental SD	Experimental Total	Control Mean	Control SD	Control Total	Weight	Std. Mean Difference IV, Random, 95% CI
Adu-Afarwuah 2007	110.1	14.3	98	105.8	14.3	96	6.7%	0.30 [0.02, 0.58]
Agostoni 2007	107.6	11	65	99.7	10.3	62	6.6%	0.74 [0.38, 1.10]
Albelbeisi 2020	11.24	0.44	100	10.81	0.59	100	6.7%	0.82 [0.53, 1.11]
Attanasio 2014	120.44	11.87	316	120.94	12.06	321	6.8%	-0.04 [-0.20, 0.11]
Barffour 2019	111.6	0.4	689	110.6	0.4	726	6.8%	2.50 [2.36, 2.64]
Ghosh 2019	107.01	46.8	307	108.66	13.89	292	6.8%	-0.05 [-0.21, 0.11]
Giovannini 2006	107.6	11	65	99.7	10.3	62	6.6%	0.74 [0.38, 1.10]
Jack 2012	10.9	0.88	443	10.79	0.88	457	6.8%	0.12 [-0.01, 0.26]
Larson 2018	9.9	2.86	1399	10.2	1.91	1406	6.8%	-0.12 [-0.20, -0.05]
Matias 2018	115.2	12.79	299	112.4	13.4	272	6.8%	0.21 [0.05, 0.38]
Menon 2007	104.4	0.8	254	100.5	1.1	161	6.6%	4.20 [3.85, 4.54]
Sazawal 2014	0.37	1.1	62	0.23	1.3	48	6.6%	0.12 [-0.26, 0.49]
Sharieff 2006	103	8	13	99	12	13	5.9%	0.38 [-0.40, 1.16]
Somasse 2018	0.5	1.22	296	0.09	1.21	246	6.8%	0.34 [0.17, 0.51]
Soofi 2013	10.1	1.47	210	9.2	1.85	211	6.8%	0.54 [0.34, 0.73]
**Total (95% CI)**			**4616**			**4473**	**100.0%**	**0.72 [0.22, 1.22]**

Heterogeneity: $Tau^2 = 0.95$; $Chi^2 = 1624.64$, df = 14 (P < 0.00001); $I^2 = 99\%$
Test for overall effect: Z = 2.81 (P = 0.005)

Std. Mean Difference
IV, Random, 95% CI

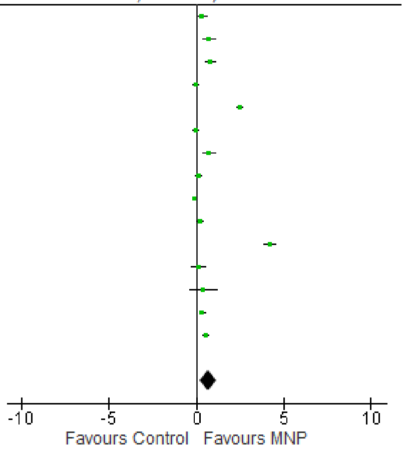

3. Outcome: WAZ

Study or Subgroup	Experimental Mean	Experimental SD	Experimental Total	Control Mean	Control SD	Control Total	Weight	Mean Difference IV, Random, 95% CI
Aboud 2011	-1.86	1	100	-2	1.1	88	5.6%	0.14 [-0.16, 0.44]
Adu-Afarwuah 2007	-0.53	1.1	96	-0.74	1.1	81	5.1%	0.21 [-0.12, 0.54]
Albelbeisi 2020	0.57	0.65	100	-0.15	0.7	100	9.1%	0.72 [0.53, 0.91]
Barffour 2019	-1.51	0.93	701	-1.52	0.9	740	13.0%	0.01 [-0.08, 0.10]
Ghosh 2019	-0.947	0.52	307	-1.05	0.68	292	12.9%	0.10 [0.01, 0.20]
Giovannini 2006	-1.25	0.69	65	-1.08	0.68	62	7.3%	-0.17 [-0.41, 0.07]
Islam 2022	-1.07	0.02	443	-1.18	0.02	453	15.2%	0.11 [0.11, 0.11]
Larson 2018	-1.8	1.9	1399	-1.8	0.95	1406	12.3%	0.00 [-0.11, 0.11]
Mridha 2016	-1.77	0.94	894	-1.75	0.93	816	13.2%	-0.02 [-0.11, 0.07]
Sazawal 2014	0.65	0.79	62	0.52	0.67	48	6.3%	0.13 [-0.14, 0.40]
**Total (95% CI)**			**4167**			**4086**	**100.0%**	**0.11 [0.02, 0.20]**

Heterogeneity: $Tau^2 = 0.01$; $Chi^2 = 62.81$, df = 9 (P < 0.00001); $I^2 = 86\%$
Test for overall effect: Z = 2.37 (P = 0.02)

Mean Difference
IV, Random, 95% CI

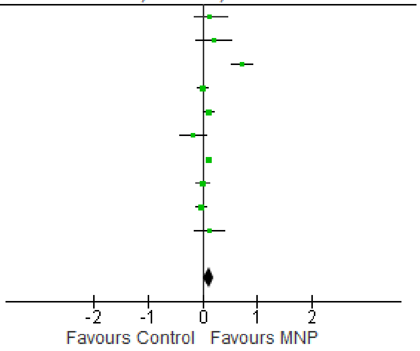

4. Outcome: HAZ

Study or Subgroup	Experimental Mean	Experimental SD	Experimental Total	Control Mean	Control SD	Control Total	Weight	Mean Difference IV, Random, 95% CI
Aboud 2011	-1.59	1.3	100	-1.82	1.1	88	2.8%	0.23 [-0.11, 0.57]
Adu-Afarwuah 2007	-0.4	1	96	-0.4	1	81	3.6%	0.00 [-0.30, 0.30]
Albelbeisi 2020	-0.21	1.33	100	-0.78	0.66	100	3.7%	0.57 [0.28, 0.86]
Barffour 2019	-1.94	1	701	-1.93	1	740	13.7%	-0.01 [-0.11, 0.09]
Ghosh 2019	-1.21	0.54	307	-1.266	0.52	292	15.7%	0.06 [-0.03, 0.14]
Giovannini 2006	-1.56	0.78	65	-1.63	0.7	62	4.6%	0.07 [-0.19, 0.33]
Islam 2022	-1.33	0.02	443	-1.41	0.02	453	22.4%	0.08 [0.08, 0.08]
Larson 2018	-1.5	1.9	1399	-1.5	1.91	1406	10.3%	0.00 [-0.14, 0.14]
Mridha 2016	-1.85	0.95	894	-1.8	0.94	816	15.2%	-0.05 [-0.14, 0.04]
Sazawal 2014	-0.22	0.46	62	-0.21	0.47	48	7.9%	-0.01 [-0.19, 0.17]
**Total (95% CI)**			**4167**			**4086**	**100.0%**	**0.05 [-0.01, 0.11]**

Heterogeneity: Tau² = 0.00; Chi² = 25.44, df = 9 (P = 0.003); I² = 65%
Test for overall effect: Z = 1.52 (P = 0.13)

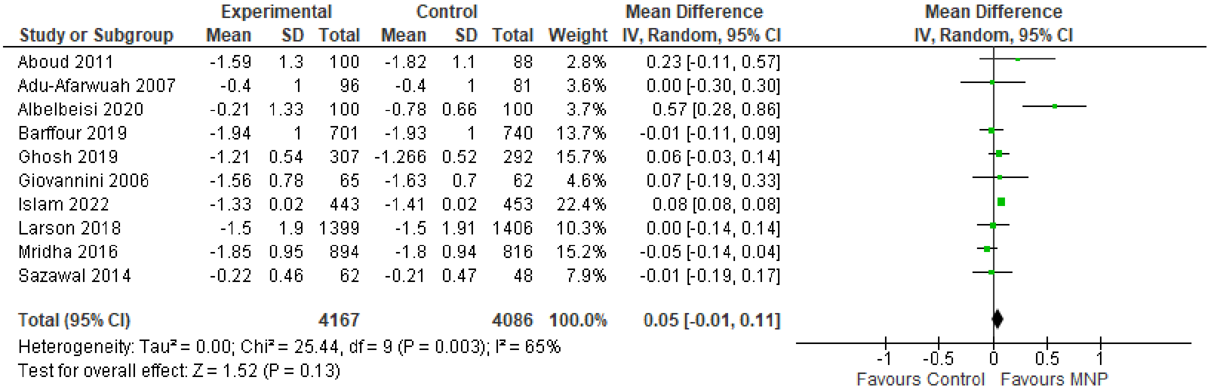

5. Outcome: WHZ

Study or Subgroup	Experimental Mean	Experimental SD	Experimental Total	Control Mean	Control SD	Control Total	Weight	Mean Difference IV, Random, 95% CI
Adu-Afarwuah 2007	-0.45	1.1	96	-0.74	1.1	81	2.6%	0.29 [-0.04, 0.62]
Albelbeisi 2020	1	1.79	100	0.31	0.88	100	1.9%	0.69 [0.30, 1.08]
Barffour 2019	-0.72	0.85	701	-0.74	0.82	740	15.6%	0.02 [-0.07, 0.11]
Ghosh 2019	-0.546	0.45	307	-0.658	0.62	292	15.5%	0.11 [0.02, 0.20]
Giovannini 2006	-0.46	0.76	65	-0.26	0.82	62	3.5%	-0.20 [-0.48, 0.08]
Islam 2022	-0.61	0.03	443	-0.71	0.03	453	24.8%	0.10 [0.10, 0.10]
Larson 2018	-1.3	0.95	1399	-1.4	0.95	1406	17.8%	0.10 [0.03, 0.17]
Mridha 2016	-1.11	0.89	894	-1.11	0.89	816	15.8%	0.00 [-0.08, 0.08]
Sazawal 2014	0.91	0.99	62	0.66	0.81	48	2.5%	0.25 [-0.09, 0.59]
**Total (95% CI)**			**4067**			**3998**	**100.0%**	**0.08 [0.03, 0.14]**

Heterogeneity: Tau² = 0.00; Chi² = 24.12, df = 8 (P = 0.002); I² = 67%
Test for overall effect: Z = 2.91 (P = 0.004)

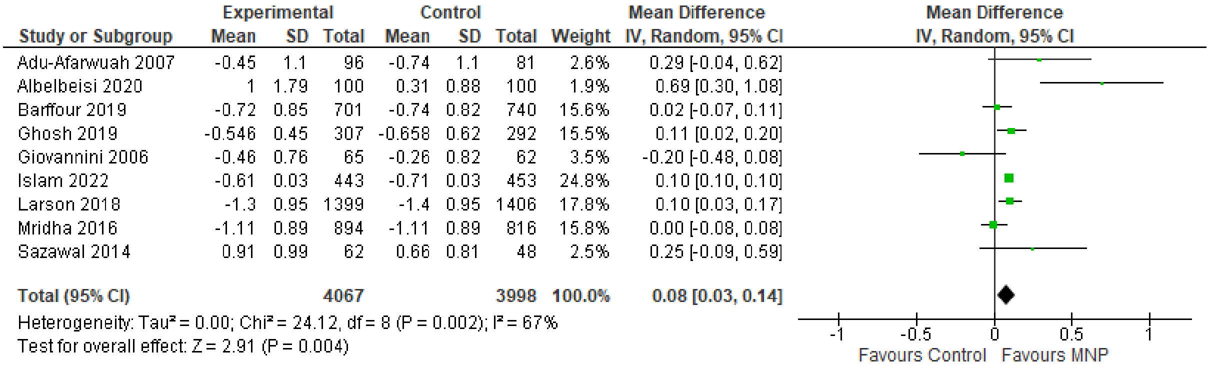

6. Outcome: Stunting

Study or Subgroup	log[Risk Ratio]	SE	Experimental Total	Control Total	Weight	Risk Ratio IV, Fixed, 95% CI
Albelbeisi 2020	-2.3979	1.0348	100	100	0.1%	0.09 [0.01, 0.69]
Barffour 2019	0.1153	0.0578	701	740	18.4%	1.12 [1.00, 1.26]
Islam 2022	-0.0556	0.1135	443	453	4.8%	0.95 [0.76, 1.18]
Jack 2012	-0.067	0.0813	490	499	9.3%	0.94 [0.80, 1.10]
Larson 2018	0.0201	0.0538	1399	1406	21.2%	1.02 [0.92, 1.13]
Mridha 2016	0.0242	0.0563	894	816	19.4%	1.02 [0.92, 1.14]
Soofi 2013	-0.01	0.0479	1239	625	26.8%	0.99 [0.90, 1.09]
**Total (95% CI)**			**5266**	**4639**	**100.0%**	**1.02 [0.97, 1.07]**

Heterogeneity: Chi² = 10.15, df = 6 (P = 0.12); I² = 41%
Test for overall effect: Z = 0.70 (P = 0.49)

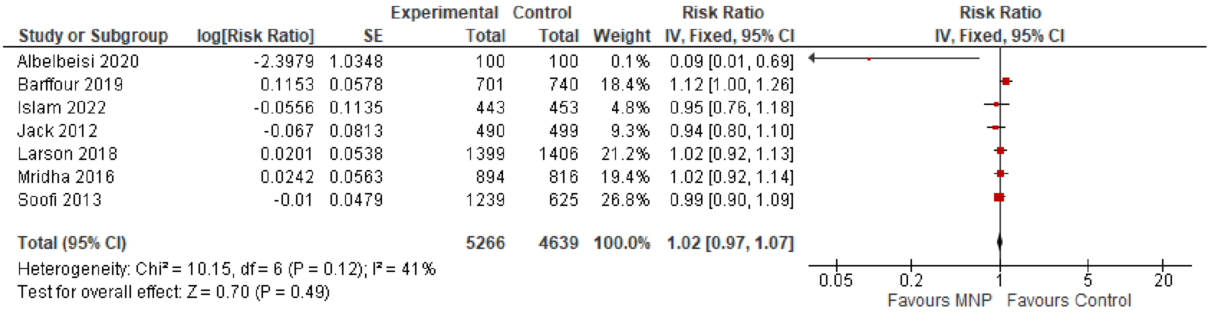

7. Outcome: Wasting

Study or Subgroup	log[Risk Ratio]	SE	Experimental Total	Control Total	Weight	Risk Ratio IV, Fixed, 95% CI
Albelbeisi 2020	-1.6094	1.5428	100	100	0.1%	0.20 [0.01, 4.11]
Barffour 2019	0.1624	0.2268	701	740	4.1%	1.18 [0.75, 1.83]
Islam 2022	-0.2564	0.2414	443	453	3.7%	0.77 [0.48, 1.24]
Jack 2012	0.174	0.271	490	499	2.9%	1.19 [0.70, 2.02]
Larson 2018	-0.0372	0.0621	1399	1406	55.2%	0.96 [0.85, 1.09]
Mridha 2016	-0.1158	0.1183	894	816	15.2%	0.89 [0.71, 1.12]
Soofi 2013	0.1221	0.1066	1236	624	18.7%	1.13 [0.92, 1.39]
**Total (95% CI)**			**5263**	**4638**	**100.0%**	**0.99 [0.90, 1.08]**

Heterogeneity: Chi² = 5.67, df = 6 (P = 0.46); I² = 0%
Test for overall effect: Z = 0.31 (P = 0.76)

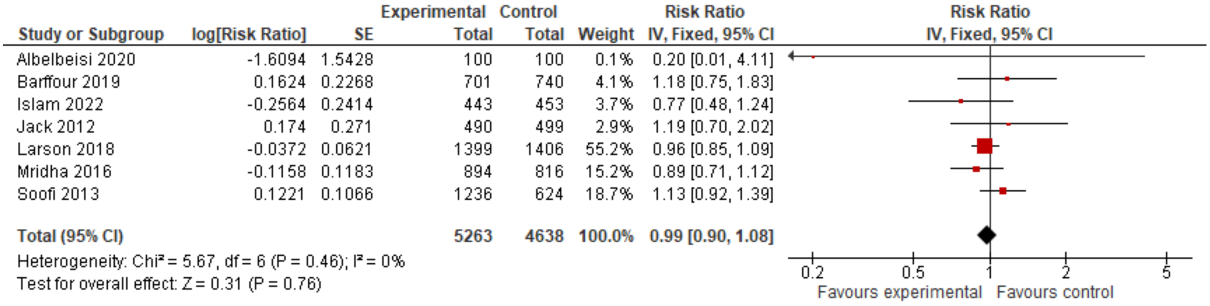

8. Outcome: Underweight

Study or Subgroup	log[Risk Ratio]	SE	Experimental Total	Control Total	Weight	Risk Ratio IV, Fixed, 95% CI
Albelbeisi 2020	-1.9459	1.5053	100	100	0.0%	0.14 [0.01, 2.73]
Barffour 2019	0.0241	0.085	701	740	10.5%	1.02 [0.87, 1.21]
Islam 2022	-0.258	0.1461	443	453	3.6%	0.77 [0.58, 1.03]
Jack 2012	-0.1627	0.1085	490	499	6.4%	0.85 [0.69, 1.05]
Larson 2018	-0.0035	0.0446	1399	1406	38.1%	1.00 [0.91, 1.09]
Mridha 2016	0.0247	0.0614	894	816	20.1%	1.03 [0.91, 1.16]
Soofi 2013	0.0626	0.0597	1378	709	21.3%	1.06 [0.95, 1.20]
**Total (95% CI)**			**5405**	**4723**	**100.0%**	**1.00 [0.95, 1.05]**

Heterogeneity: Chi² = 8.39, df = 6 (P = 0.21); I² = 28%
Test for overall effect: Z = 0.03 (P = 0.98)

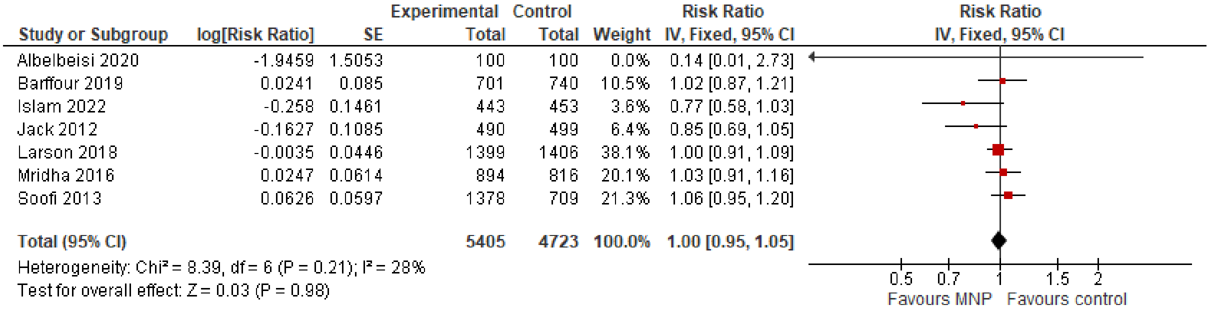

9. Outcome: Diarrhea

Study or Subgroup	log[Risk Ratio]	SE	Experimental Total	Control Total	Weight	Risk Ratio IV, Fixed, 95% CI
Giovannini 2006	0.5124	0.6011	65	62	1.2%	1.67 [0.51, 5.42]
Larson 2018	-0.0424	0.0827	2184	2176	63.6%	0.96 [0.82, 1.13]
Menon 2007	0.2875	0.1487	125	81	19.7%	1.33 [1.00, 1.78]
Mridha 2016	0.2473	0.1671	907	838	15.6%	1.28 [0.92, 1.78]
**Total (95% CI)**			**3281**	**3157**	**100.0%**	**1.08 [0.95, 1.23]**

Heterogeneity: Chi² = 5.65, df = 3 (P = 0.13); I² = 47%
Test for overall effect: Z = 1.13 (P = 0.26)

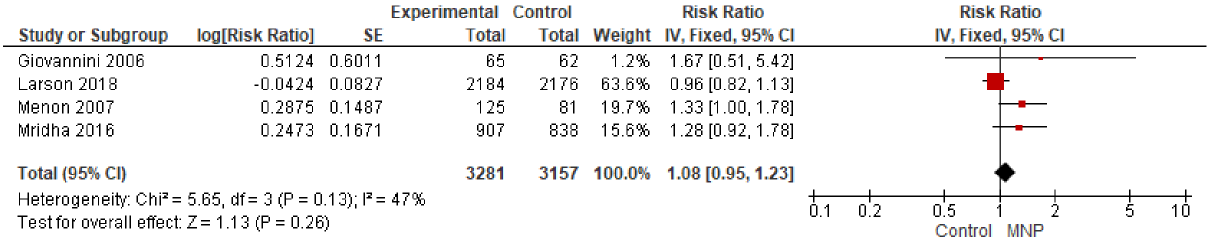

10. Outcome: URI

Study or Subgroup	log[Risk Ratio]	SE	Experimental Total	Control Total	Weight	Risk Ratio IV, Fixed, 95% CI
Giovannini 2006	0.1759	0.6469	65	62	1.1%	1.19 [0.34, 4.24]
Mridha 2016	-0.1113	0.0697	907	838	98.9%	0.89 [0.78, 1.03]
**Total (95% CI)**			**972**	**900**	**100.0%**	**0.90 [0.78, 1.03]**

Heterogeneity: Chi² = 0.19, df = 1 (P = 0.66); I² = 0%
Test for overall effect: Z = 1.56 (P = 0.12)

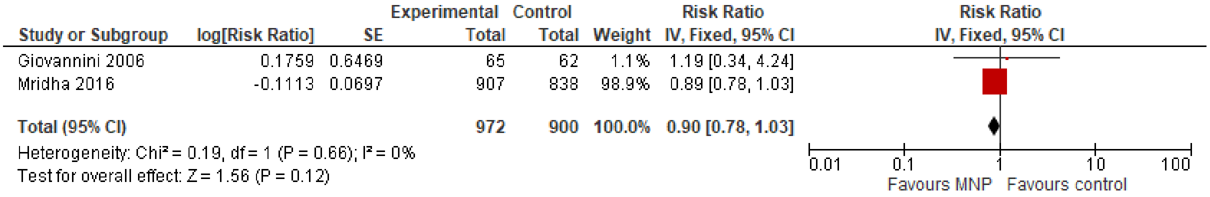

Supplement: Supplementary file 1 [file nutrients-15-03041-s001.zip › Supplementary File S4.pdf]
